# Association Between SGLT2is and Cardiovascular and Respiratory Diseases: A Meta-Analysis of Large Trials

**DOI:** 10.3389/fphar.2021.724405

**Published:** 2021-07-26

**Authors:** Dao-Gen Yin, Mei Qiu, Xue-Yan Duan

**Affiliations:** ^1^ Center of Community Health Service Management, Shenzhen Longhua District Central Hospital, Shenzhen, China; ^2^ Department of General Medicine, Shenzhen Longhua District Central Hospital, Shenzhen, China

**Keywords:** SGLT2is, atrial fibrillation, bradycardia, hypertensive emergency, chronic obstructive pulmonary disease, asthma, sleep apnoea syndrome

## Abstract

The association between sodium-glucose cotransporter 2 inhibitors (SGLT2is) and various cardiovascular and respiratory diseases is unestablished. This meta-analysis aimed to explore whether use of SGLT2is is significantly associated with the occurrences of 80 types of cardiovascular diseases and 55 types of respiratory diseases. Large randomized trials of SGLT2is were included in analysis. Meta-analysis was conducted to synthesize risk ratio (RR) and 95% confidence interval (CI). Nine large trials were included in analysis. Compared to placebo, SGLT2is were associated with the reduced risks of 9 types of cardiovascular diseases (e.g., atrial fibrillation [RR 0.78, 95% CI 0.67-0.91], bradycardia [RR 0.60, 95% CI 0.40-0.89], and hypertensive emergency [RR 0.29, 95% CI 0.12-0.72]) and 11 types of respiratory diseases (e.g., chronic obstructive pulmonary disease [RR 0.77, 95% CI 0.61-0.97], asthma [RR 0.57, 95% CI 0.35-0.95], and sleep apnoea syndrome [RR 0.36, 95% CI 0.15-0.87]). The results of random-effects meta-analysis were similar with those of fixed-effects meta-analysis. No heterogeneity or only little heterogeneity was found in most meta-analyses. No publication bias was observed in most of the meta-analyses conducted in this study. SGLT2is were not significantly associated with the other 115 cardiovascular and respiratory diseases. SGLT2is are associated with the reduced risks of 9 types of cardiovascular diseases (e.g., atrial fibrillation, bradycardia, and hypertensive emergency) and 11 types of respiratory diseases (e.g., chronic obstructive pulmonary disease, asthma, and sleep apnoea syndrome). This proposes the potential of SGLT2is to be used for prevention of these cardiovascular and respiratory diseases.

## Introduction

Sodium-glucose cotransporter 2 inhibitors (SGLT2is) have been confirmed, by large cardiovascular outcome trials, to have the obvious efficacy in reducing arteriosclerotic cardiovascular events and heart failure events. However, the impact of this drug class on other cardiovascular diseases such as arrhythmia, hypertensive emergency, and varicose vein is not established. Meanwhile, relevant animal studies (Park et al., 2019; Chowdhury et al., 2020; Lin et al., 2020) have revealed the protective effects of SGLT2is against some respiratory diseases, whereas these benefits of SGLT2is have not been confirmed by large clinical trials.

Although there are no large randomized trials which have aimed to assess the impact of SGLT2is on the occurrences of various cardiovascular and respiratory diseases, those trials focusing on the cardiorenal endpoints with SGLT2is reported in detail the occurrences of various serious adverse events (SAEs), which included the occurrences of various cardiovascular and respiratory diseases. These data of SAEs make it possible to evaluate the association between use of SGLT2is and the occurrences of various cardiovascular and respiratory diseases.

However, due to the low incidences of most SAEs in cardiorenal outcome trials of SGLT2is, individual trials are not powered to draw a definitive conclusion on whether use of SGLT2is significantly affects the incidences of various cardiovascular and respiratory SAEs. Thus, we intended to, based on the SAEs data from the large cardiorenal outcome trials of SGLT2is, conduct a meta-analysis to explore whether use of SGLT2is is significantly associated with the occurrences of various cardiovascular and respiratory diseases.

## Methods

This study is reported according to the Preferred Reporting Items for Systematic Reviews and Meta-Analyses (PRISMA) statement (Moher et al., 2009). We searched Embase, Cochrane Central Register of Controlled Trials (CENTRAL), and PubMed, to obtain relevant studies published before April 7th, 2021. The search terms included but were not limited to “SGLT2 inhibitors,” “Empagliflozin,” “Dapagliflozin,” “Canagliflozin,” “Ertugliflozin”, and “Trial.” In this meta-analysis we included those large randomized trials that compared any SGLT2i with placebo. We excluded those trials assessing sotagliflozin because it also inhibits SGLT1 besides SGLT2, and excluded those trials in which there was at least one study group with less than one thousand participants for fear of small-study effects. Different doses of SGLT2is were not separately considered in this study. Included trials were evaluated for quality according to the Cochrane risk of bias assessment tool (Higgins et al., 2011). SAEs of interest for this study consisted of 80 kinds of cardiovascular disorders (detailed in Supplementary Table S1) and 55 respiratory disorders (detailed in Supplementary Table S2). The data regarding various SAEs of interest were extracted from the ClinicalTrials.gov website or included articles. Two authors independently conducted study selection, quality assessment, and data extraction; and all the inconsistencies they encountered were solved by a third author’s arbitration.

The numbers of patients developing SAEs of interest and those of all randomly assigned patients in each group were used to perform meta-analysis to derive pooled risk ratios (RRs) and 95% confidence intervals (CIs). We conducted meta-analysis respectively using the fixed-effects model with the inverse variance method and the random-effects model with the method of DerSimonian & Laird (DerSimonian and Kacker, 2007), to evaluate the robustness of meta-analysis results. The magnitude of heterogeneity across studies was reflected by I^2^ statistic. We detected publication bias by drawing funnel plots and conducting Egger test (Egger et al., 1997). *p* < 0.05 denotes statistical significance. All statistical analyses were implemented in the Stata software (version 16.0).

## Results

After study selection (Supplementary Figure S1 in Supplementary Appendix S1), we finally included nine large trials (Zinman et al., 2015; Neal et al., 2017; McMurray et al., 2019; Perkovic et al., 2019; Wiviott et al., 2019; Cannon et al., 2020; Heerspink et al., 2020; Packer et al., 2020) for meta-analysis. Included trials consisted of six trials enrolling patients with type 2 diabetes (i.e., CREDENCE (Perkovic et al., 2019), CANVAS (Neal et al., 2017), CANVAS-R (Neal et al., 2017), DECLARE-TIMI 58 (Wiviott et al., 2019), EMPA-REG OUTCOME (Zinman et al., 2015), and VERTIS CV (Cannon et al., 2020)), two trials enrolling patients with heart failure (i.e., EMPEROR-Reduced (Packer et al., 2020), and DAPA-HF (McMurray et al., 2019)), and one trial enrolling patients with chronic kidney disease (i.e., DAPA-CKD (Heerspink et al., 2020)). Included trials involved a total of 33,124 participants taking SGLT2is and 26,568 participants taking placebo, and all the trials were with low risk of bias (Supplementary Figure S2 in Supplementary Appendix S1).

**FIGURE 1:**
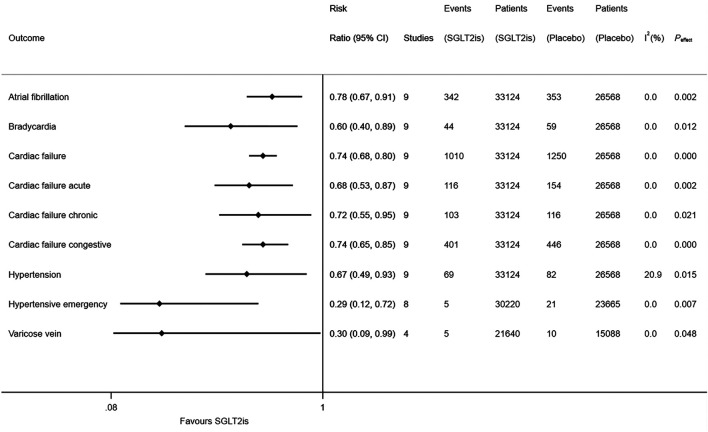
Meta-analysis of SGLT2is and nine types of cardiovascular diseases.

Compared to placebo, SGLT2is were associated with the reduced risks of atrial fibrillation (RR 0.78, 95% CI 0.67–0.91; I^2^ = 0; *P*_effect_ = 0.002), bradycardia (RR 0.60, 95% CI 0.40–0.89; I^2^ = 0; *P*_effect_ = 0.012), cardiac failure (RR 0.74, 95% CI 0.68–0.80; I^2^ = 0; *P*_effect_ < 0.001), cardiac failure acute (RR 0.68, 95% CI 0.53–0.87; I^2^ = 0; *P*_effect_ = 0.002), cardiac failure chronic (RR 0.72, 95% CI 0.55–0.95; I^2^ = 0; *P*_effect_ = 0.021), cardiac failure congestive (RR 0.74, 95% CI 0.65–0.85; I^2^ = 0; *P*_effect_ < 0.001), hypertension (RR 0.67, 95% CI 0.49–0.93; I^2^ = 20.9%; *P*_effect_ = 0.015), hypertensive emergency (RR 0.29, 95% CI 0.12–0.72; I^2^ = 0; *P*_effect_ = 0.007), and varicose vein (RR 0.30, 95% CI 0.09–0.99; I^2^ = 0; *P*_effect_ = 0.048) (Figure 1). SGLT2is were not significantly associated with the risks of 71 other cardiovascular diseases (Supplementary Table S1). The detailed results of meta-analysis of SGLT2is and 80 cardiovascular diseases are provided in Supplementary Figures S3–S82 in Supplementary Appendix S1), which suggested that the results of random-effects meta-analysis were similar with those of fixed-effects meta-analysis.

**FIGURE 2:**
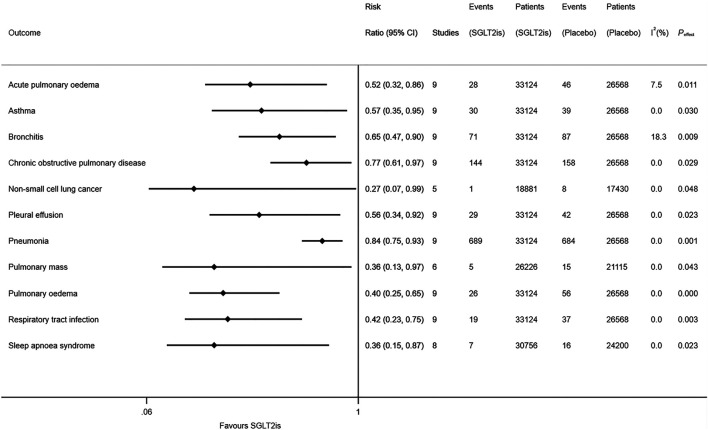
Meta-analysis of SGLT2is and 11 types of respiratory diseases.

Compared to placebo, SGLT2is were associated with the reduced risks of acute pulmonary oedema (RR 0.52, 95% CI 0.32–0.86; I^2^ = 7.5%; *P*_effect_ = 0.011), asthma (RR 0.57, 95% CI 0.35–0.95; I^2^ = 0; *P*_effect_ = 0.030), bronchitis (RR 0.65, 95% CI 0.47–0.90; I^2^ = 18.3%; *P*_effect_ = 0.009), chronic obstructive pulmonary disease (RR 0.77, 95% CI 0.61–0.97; I^2^ = 0; *P*_effect_ = 0.029), non-small cell lung cancer (RR 0.27, 95% CI 0.07–0.99; I^2^ = 0; *P*_effect_ = 0.048), pleural effusion (RR 0.56, 95% CI 0.34–0.92; I^2^ = 0; *P*_effect_ = 0.023), pneumonia (RR 0.84, 95% CI 0.75–0.93; I^2^ = 0; *P*_effect_ = 0.001), pulmonary mass (RR 0.36, 95% CI 0.13–0.97; I^2^ = 0; *P*_effect_ = 0.043), pulmonary oedema (RR 0.40, 95% CI 0.25–0.65; I^2^ = 0; *P*_effect_ < 0.001), respiratory tract infection (RR 0.42, 95% CI 0.23–0.75; I^2^ = 0; *P*_effect_ = 0.003), and sleep apnoea syndrome (RR 0.36, 95% CI 0.15–0.87; I^2^ = 0; *P*_effect_ = 0.023) (Figure 2). SGLT2is were not significantly associated with the risks of 44 other respiratory diseases (Supplementary Table S2). The detailed results of meta-analysis of SGLT2is and 55 respiratory diseases are provided in Supplementary Figures S83–S137 in Supplementary Appendix S1), which suggested that the results of random-effects meta-analysis were similar with those of fixed-effects meta-analysis.

The results of detection of publication bias are detailed in Supplementary Figures S138–S272 in Supplementary Appendix S1), suggesting that most of the meta-analyses conducted in this study had no publication bias since most of the *p* values from Egger test were greater than 0.05.

## Discussion

This is the first meta-analysis that assessed in detail the association between use of SGLT2is and the occurrence of various cardiovascular and respiratory diseases. Accordingly, it revealed that use of SGLT2is was associated with the reduced risks of nine types of cardiovascular diseases (i.e., atrial fibrillation, bradycardia, cardiac failure, cardiac failure acute, cardiac failure chronic, cardiac failure congestive, hypertension, hypertensive emergency, and varicose vein) and 11 types of respiratory diseases (i.e., acute pulmonary oedema, asthma, bronchitis, chronic obstructive pulmonary disease, non-small cell lung cancer, pleural effusion, pneumonia, pulmonary mass, pulmonary oedema, respiratory tract infection, and sleep apnoea syndrome).

Three previous meta-analyses (Li et al., 2021a; Li et al., 2021b; Zhou et al., 2021) identified SGLT2is with the reduced risk of atrial fibrillation, whereas they failed to explore the association between SGLT2is and bradycardia. Our meta-analysis further revealed the association between use of SGLT2is and a lower risk of bradycardia besides that of atrial fibrillation. Prior studies (Alqudsi et al., 2021; Hunter et al., 2021; Kario et al., 2021) showed the antihypertension effects of SGLT2is, while our meta-analysis further revealed SGLT2is with the lower incidences of hypertensive emergency and varicose vein besides hypertension. Another previous meta-analysis (Qiu et al., 2021) revealed the significant association between use of SGLT2is and the lower risks of three types of noninfectious respiratory disorders (i.e., asthma, acute pulmonary oedema, and sleep apnoea syndrome), whereas that meta-analysis (Qiu et al., 2021) failed to explore the association between SGLT2is and infectious respiratory diseases, and also failed to observe the significant association between SGLT2is and chronic obstructive pulmonary disease because it failed to incorporate the data from the two trials of VERTIS CV (Cannon et al., 2020) and EMPEROR-Reduced (Packer et al., 2020), as was stated in the Limitations section of that article (Qiu et al., 2021). In contrast, our meta-analysis additionally revealed the significant association between use of SGLT2is and the lower occurrences of three types of infectious respiratory disorders (i.e., bronchitis, pneumonia, and respiratory tract infection) and four types of noninfectious respiratory disorders (i.e., chronic obstructive pulmonary disease, non-small cell lung cancer, pleural effusion, and pulmonary mass).

In this meta-analysis SGLT2is were observed with the reduced risks of cardiac failure, cardiac failure acute, cardiac failure chronic, cardiac failure congestive, acute pulmonary oedema, and pulmonary oedema; which is consistent with the benefits of SGLT2is on heart failure endpoints observed in two heart failure trials (McMurray et al., 2019; Packer et al., 2020). The mechanisms for the anti-heart failure activity of SGLT2is are to improve myocardial efficiency and mitochondrial function, and to reduce inflammation, oxidative stress, fibrosis, and sympathetic nervous system activation (Zelniker and Braunwald, 2020). In this meta-analysis SGLT2is were observed with the reduced risks of infectious respiratory disorders, which might be associated with the glucose-lowering efficacy of SGLT2is. However, the mechanisms for the reductions SGLT2is led to in the risks of noninfectious respiratory disorders are required to be further investigated.

The strengths of this study include that the original studies included were large trials with low risk of bias and that no heterogeneity or only little heterogeneity was found in most of the meta-analyses conducted in this study. Moreover, Egger test suggested that most meta-analyses conducted in this study were not with publication bias, and the similarity between random-effects results and fixed-effects results suggested the robustness of meta-analysis results. Conversely, the main limitation of this study was that SAEs of interest for this meta-analysis were not events of special interest in the included trials. Therefore, the association between use of SGLT2is and risk reductions of some cardiovascular and pulmonary diseases revealed in this study does not definitely represent for the causal relationship. Instead, the causal relationship needs to be further confirmed. Moreover, patients the included trials enrolled were not susceptible to most of the cardiovascular and respiratory diseases assessed in this meta-analysis, which led to the low occurrences of these diseases. Thus, prospective trials enrolling patients who are susceptible to cardiovascular and respiratory diseases are warranted to confirm the protective effects of SGLT2is against cardiopulmonary disorders and whether these effects are a class effect or drug-specific effects.

In conclusion, SGLT2is are associated with the reduced risks of nine types of cardiovascular diseases (e.g., atrial fibrillation, bradycardia, and hypertensive emergency) and 11 types of respiratory diseases (e.g., chronic obstructive pulmonary disease, asthma, and sleep apnoea syndrome). This proposes the potential of SGLT2is to be used for prevention of these cardiovascular and respiratory diseases. However, due to the low incidences of these diseases among included trials, these positive findings are needed to be confirmed by prospective trials enrolling susceptible individuals.

## Abbreviations

CI, confidence interval; CENTRAL, cochrane central register of controlled trials; PRISMA, preferred reporting items for systematic reviews and meta-analyses; RR, risk ratio; SAEs, serious adverse events; SGLT2is, sodium-glucose cotransporter 2 inhibitors.
