# Supplementary material for: Association Between SGLT2is and Cardiovascular and Respiratory Diseases: A Meta-Analysis of Large Trials
Source: Front Pharmacol. 2021 Jul 26;12:724405. doi: 10.3389/fphar.2021.724405 (PMC8350118; doi:10.3389/fphar.2021.724405)
Supplement: Supplementary file 2 [file DataSheet1.PDF]

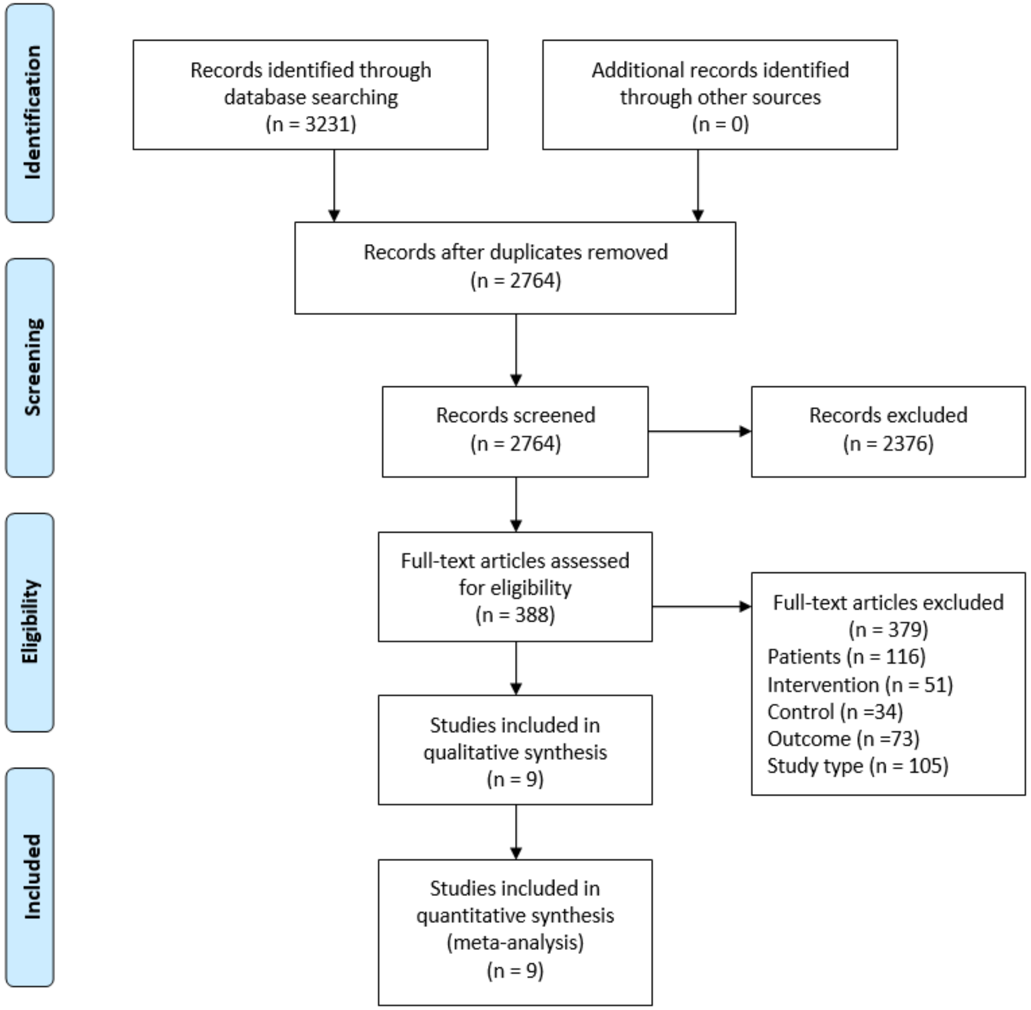

Figure S1 Flow diagram of study selection

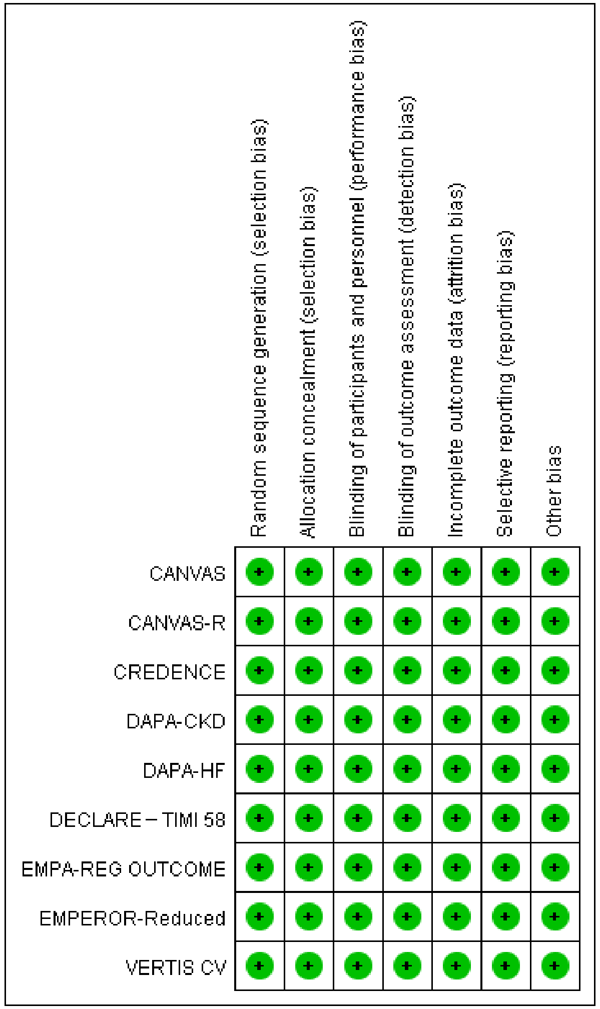

Figure S2 Risk of bias summary

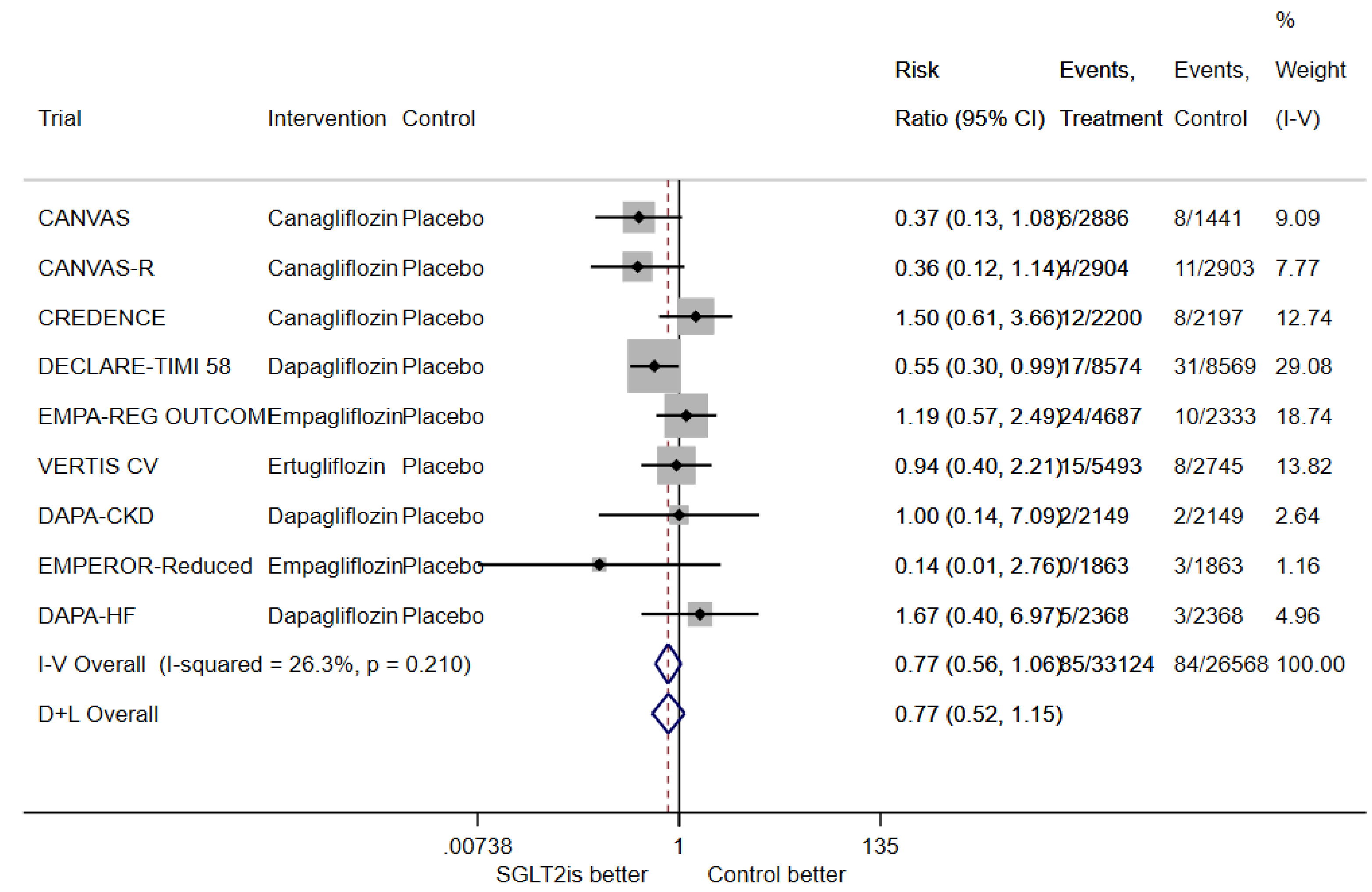

Figure S3 Meta-analysis of SGLT2is and Acute coronary syndrome

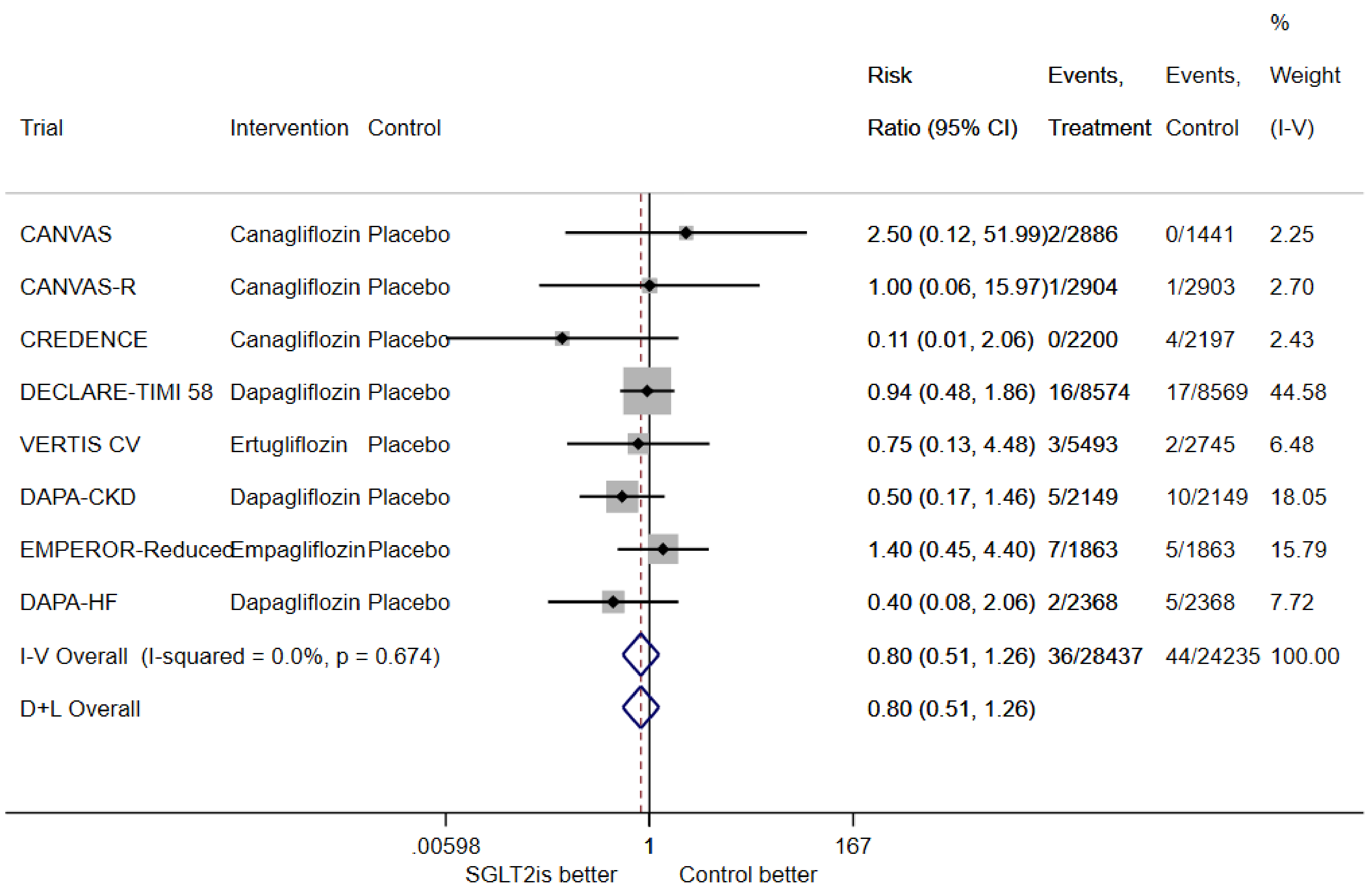

Figure S4 Meta-analysis of SGLT2is and Acute left ventricular failure

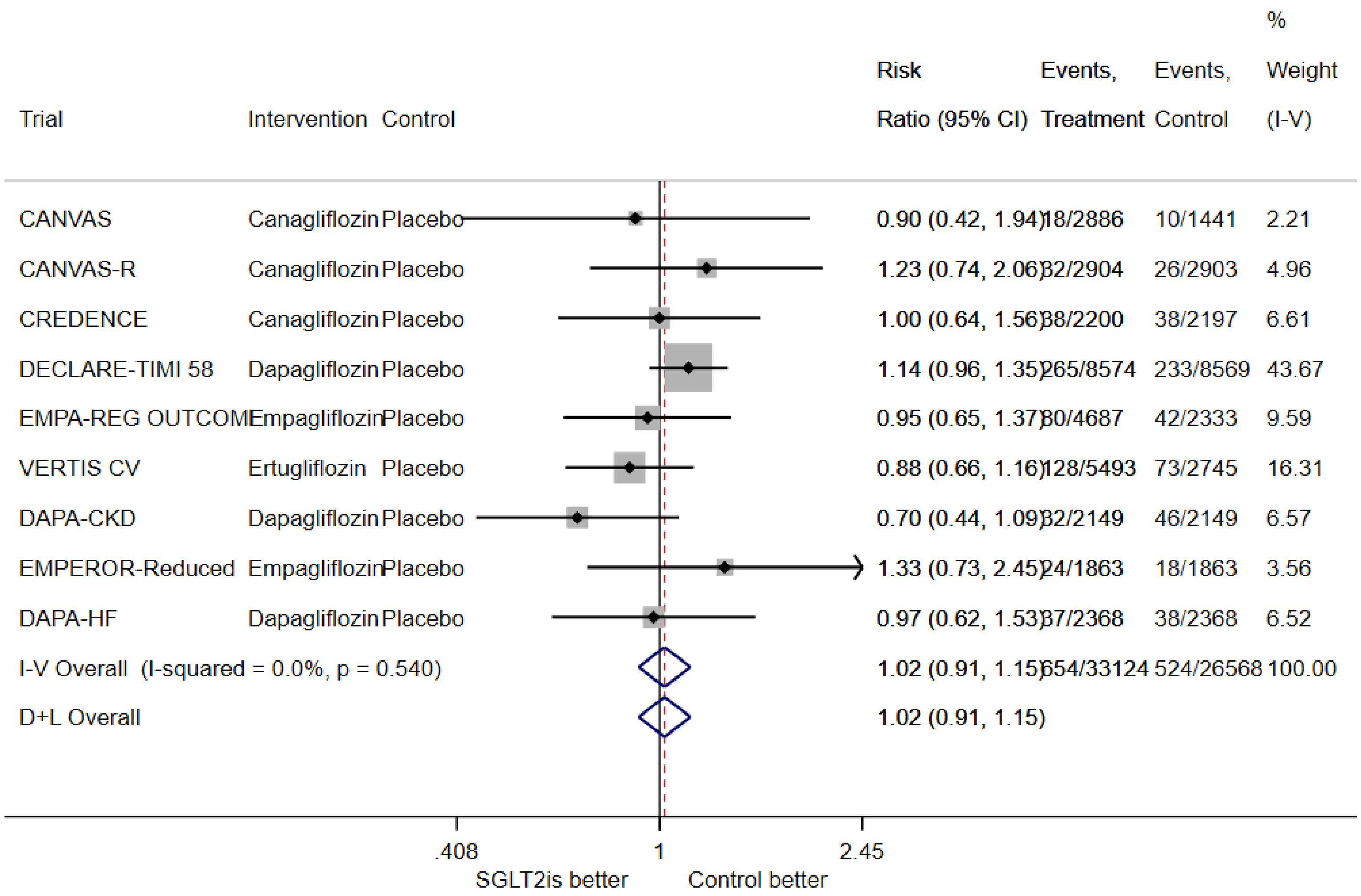

Figure S5 Meta-analysis of SGLT2is and Acute myocardial infarction

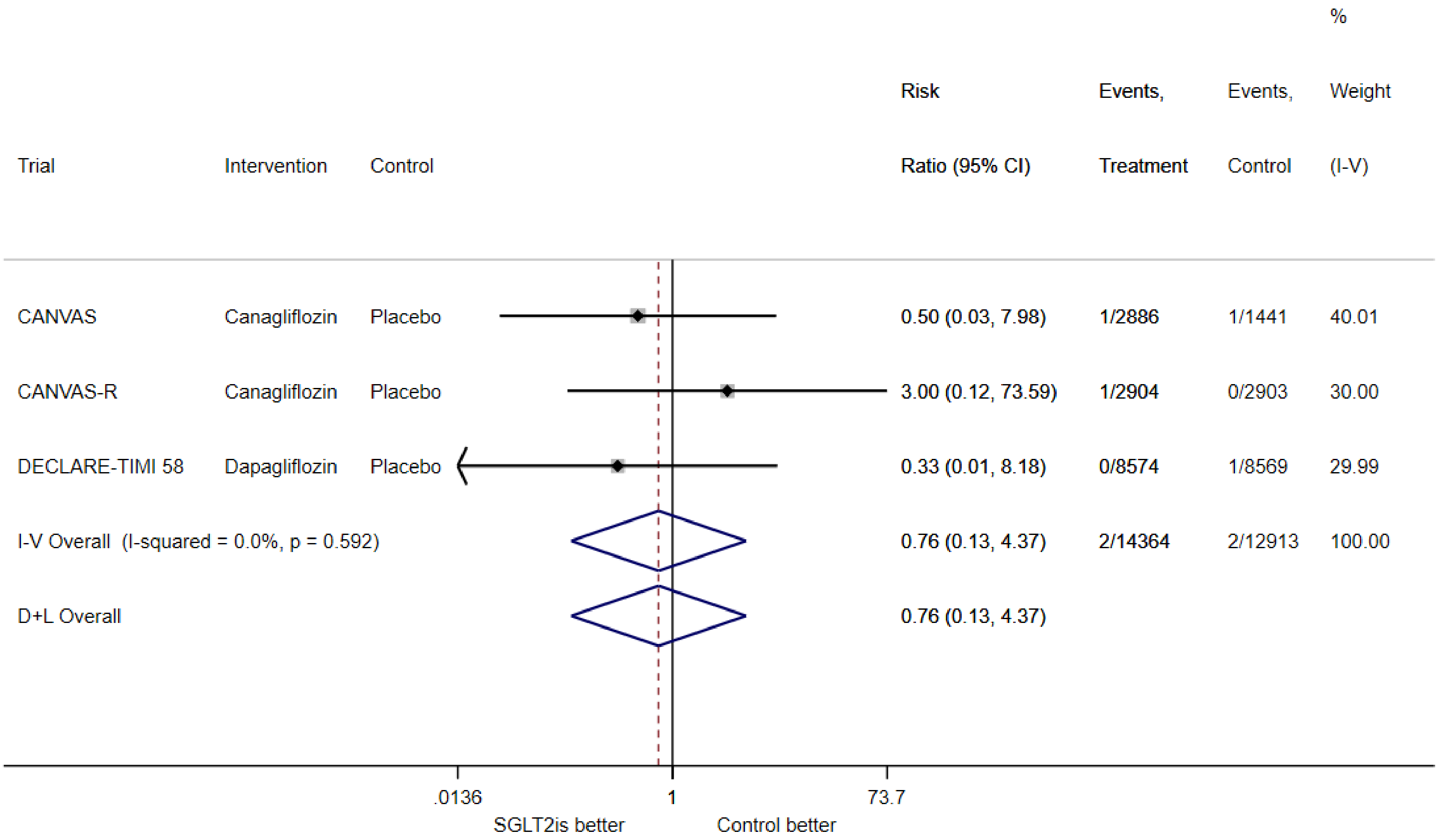

Figure S6 Meta-analysis of SGLT2is and Adams-stokes syndrome

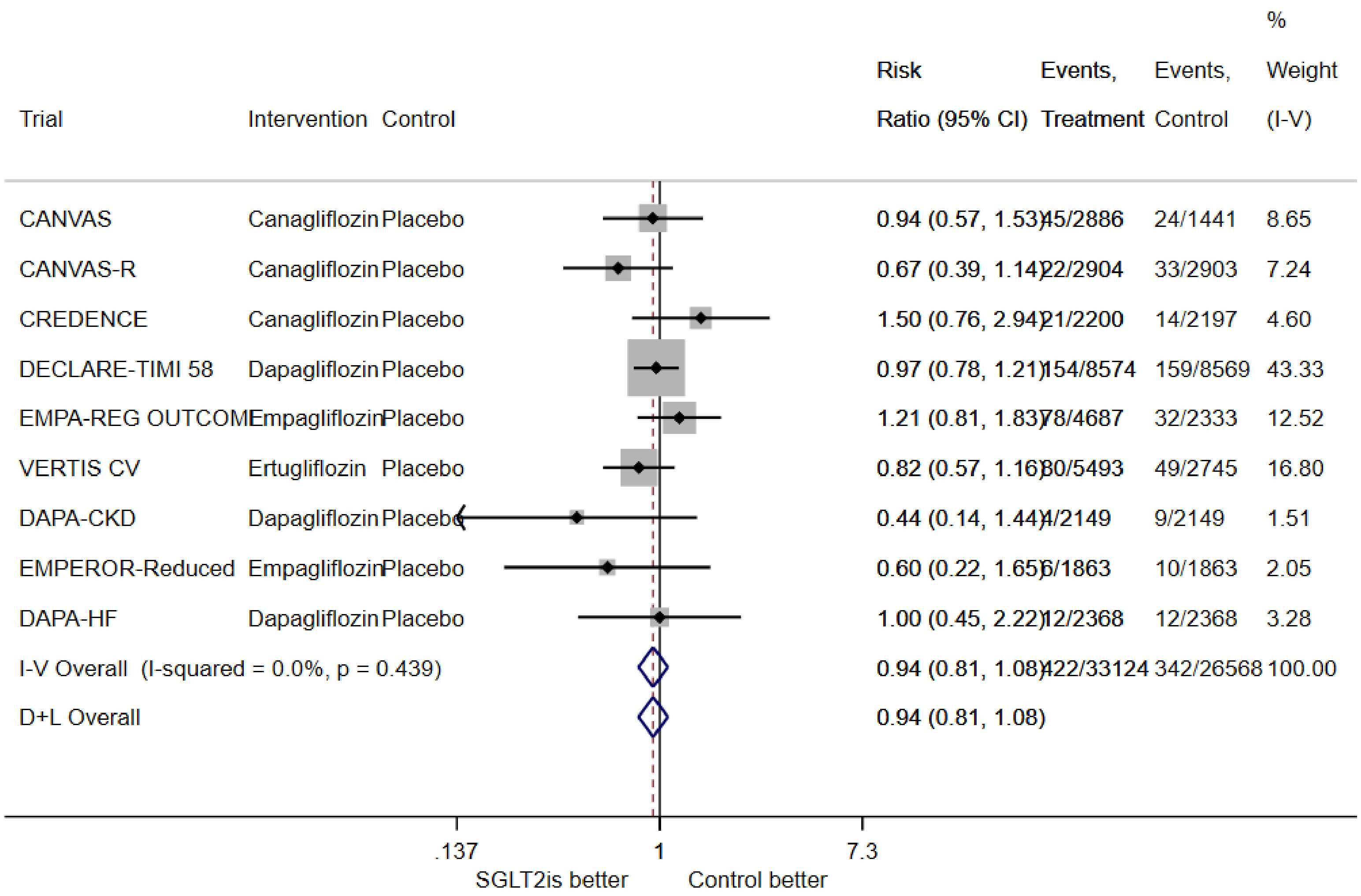

Figure S7 Meta-analysis of SGLT2is and Angina pectoris

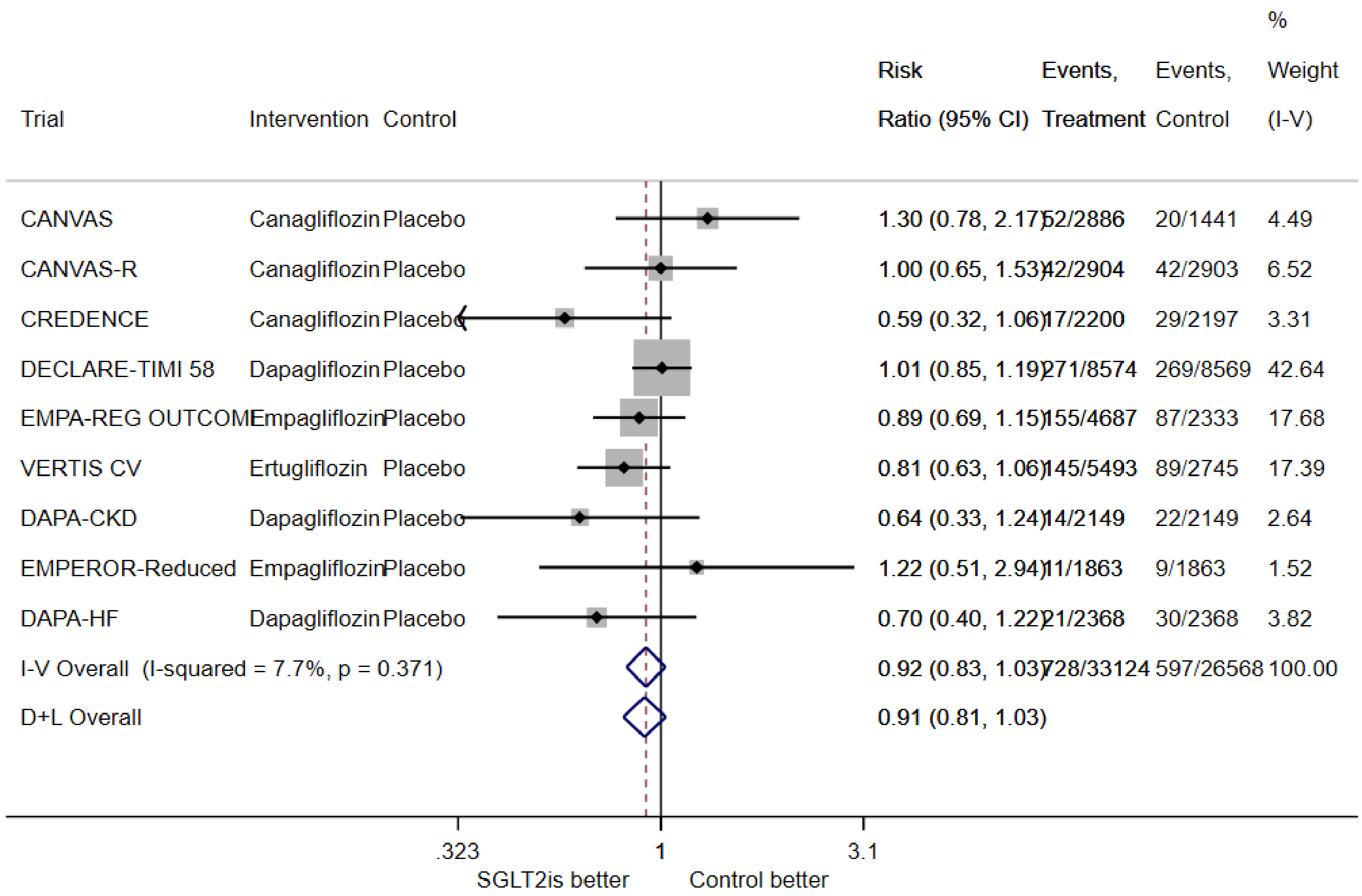

Figure S8 Meta-analysis of SGLT2is and Angina unstable

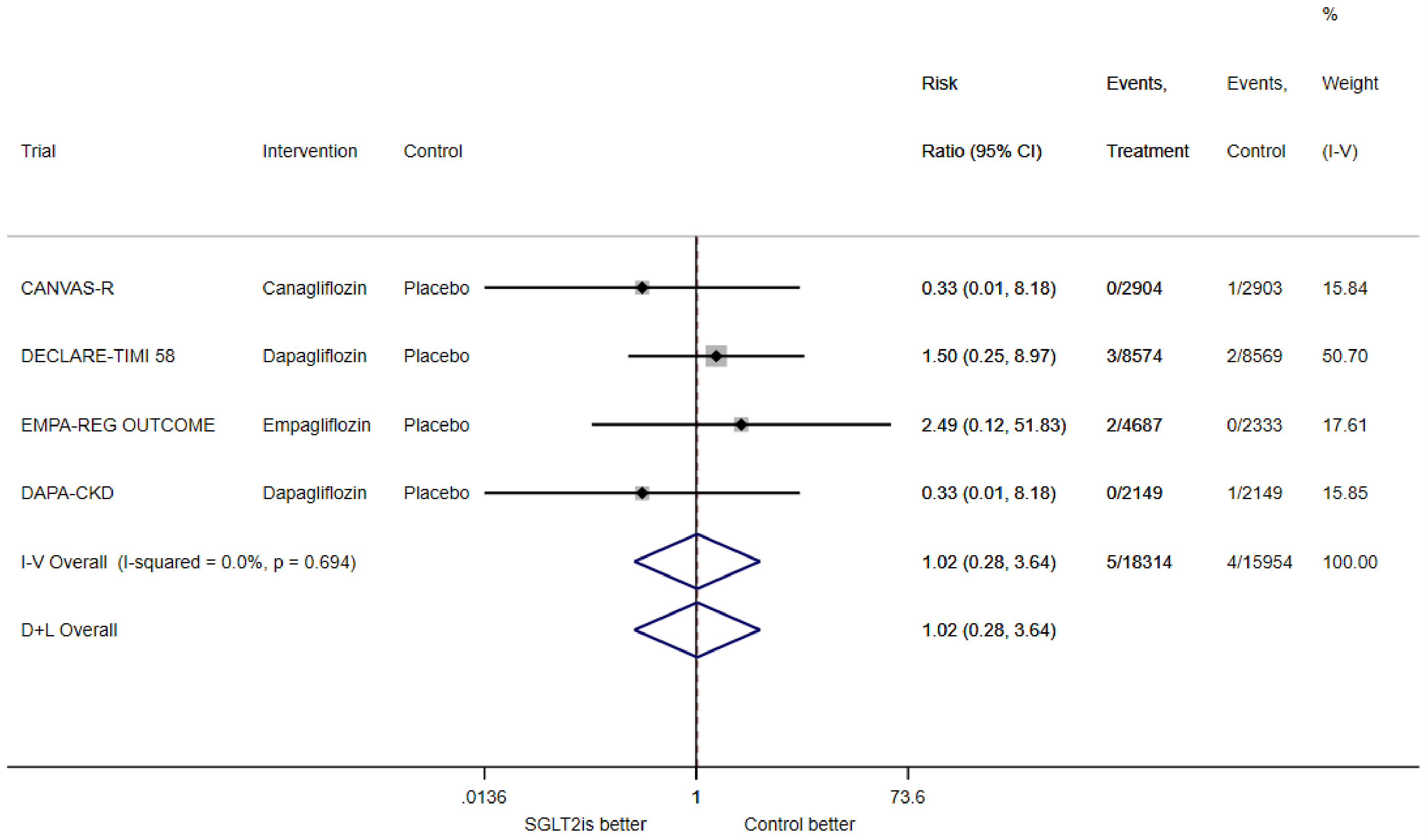

Figure S9 Meta-analysis of SGLT2is and Aortic valve disease

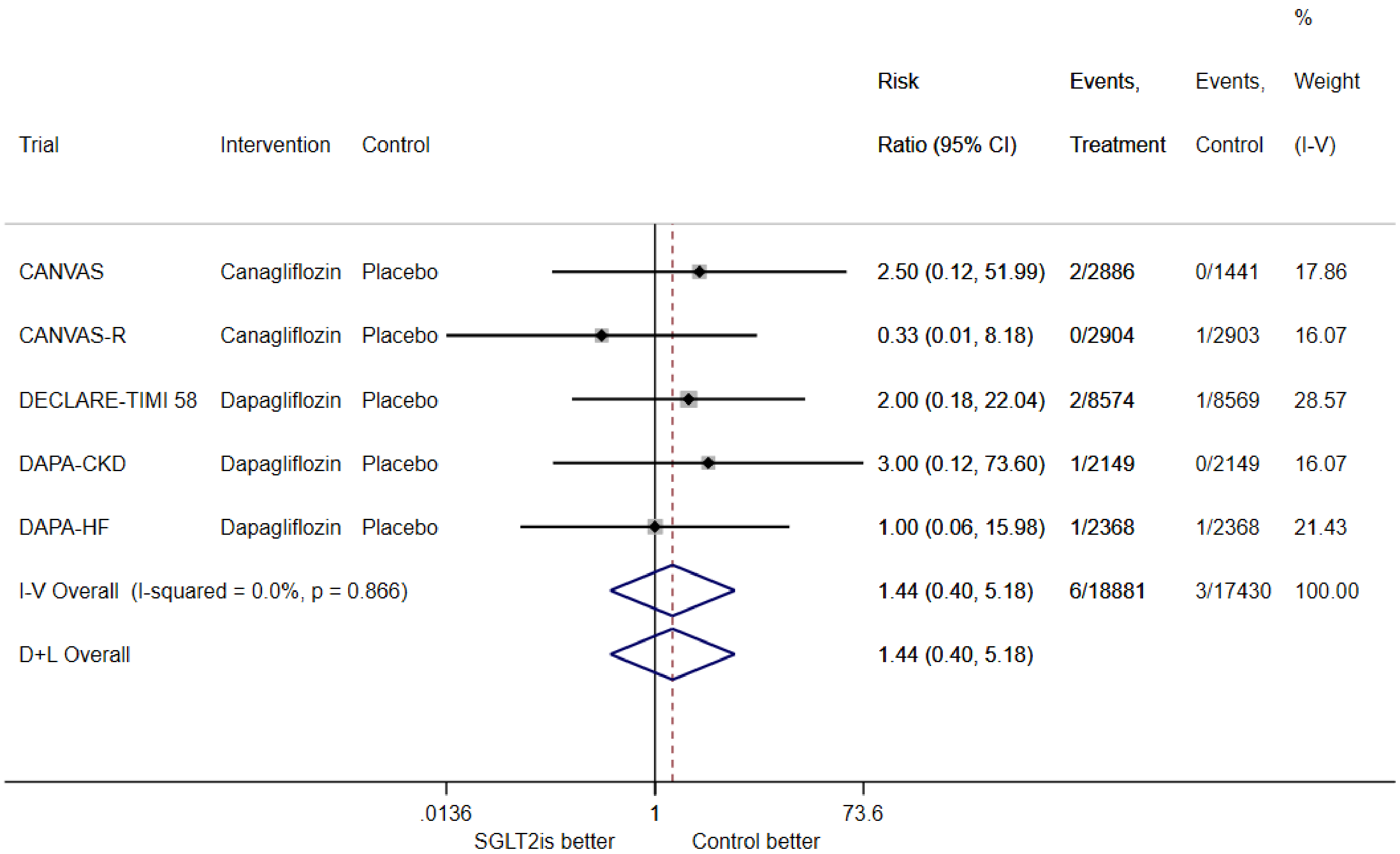

Figure S10 Meta-analysis of SGLT2is and Aortic valve incompetence

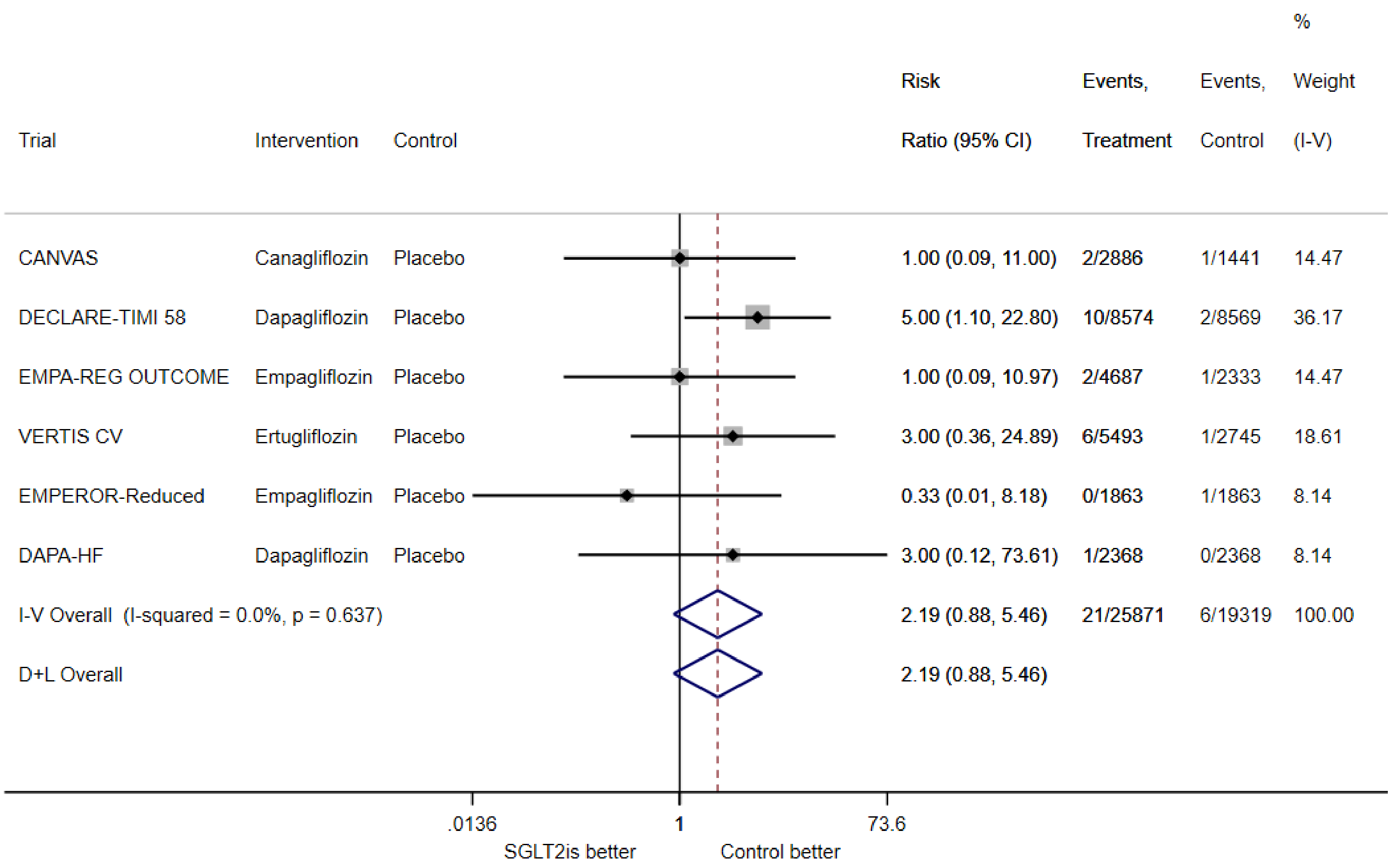

Figure S11 Meta-analysis of SGLT2is and Aortic valve stenosis

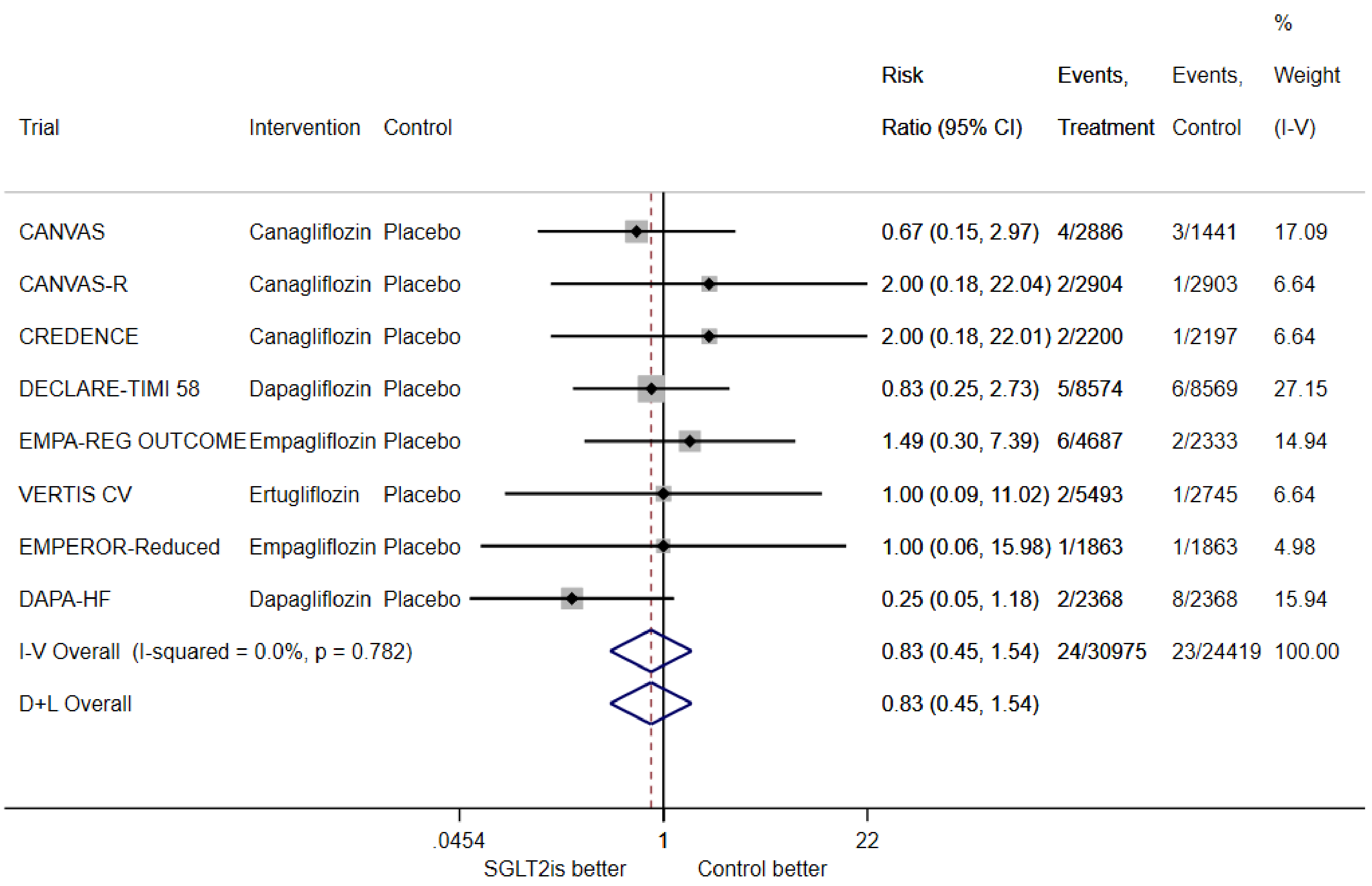

Figure S12 Meta-analysis of SGLT2is and Arrhythmia

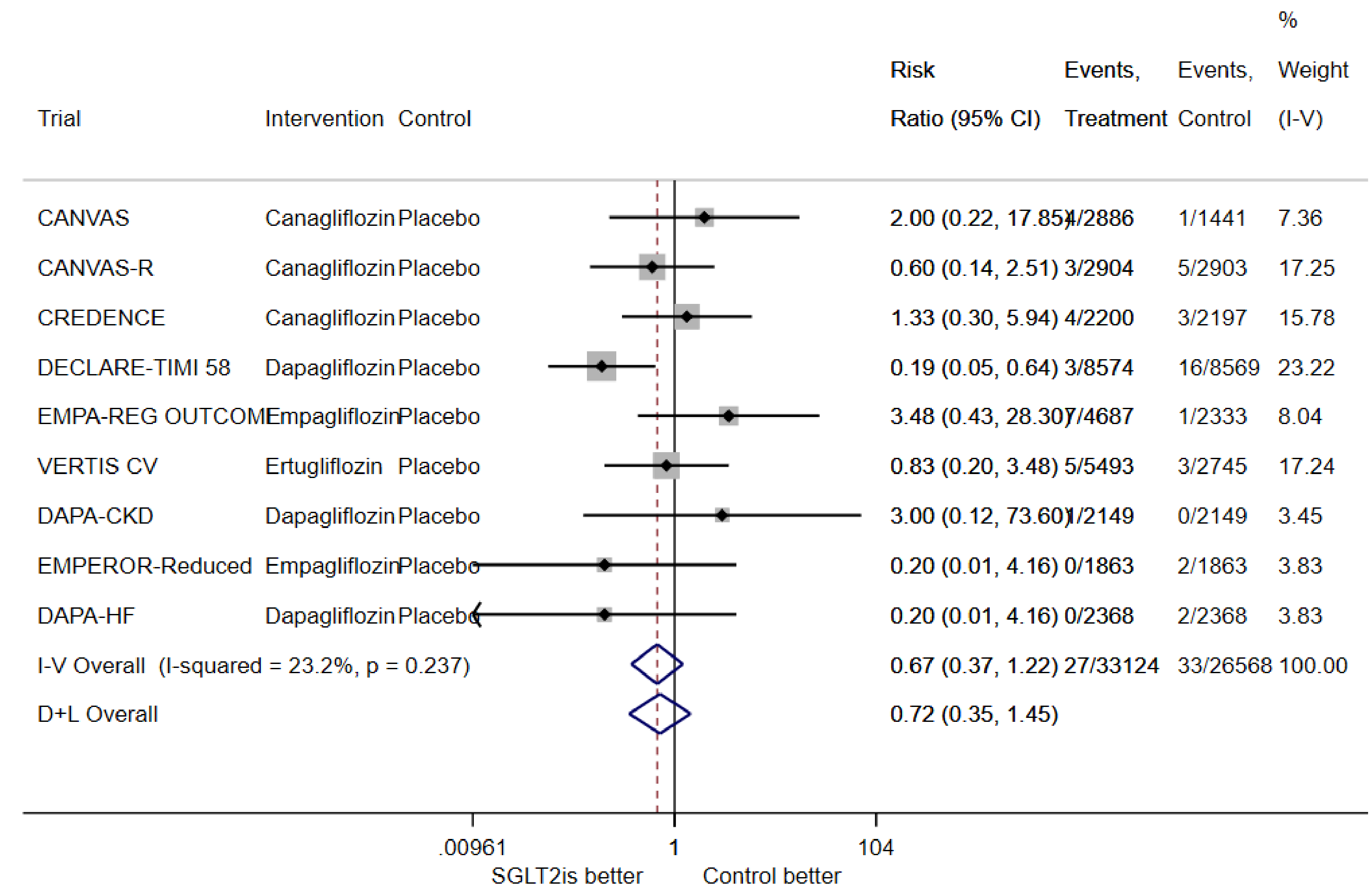

Figure S13 Meta-analysis of SGLT2is and Arteriosclerosis coronary artery

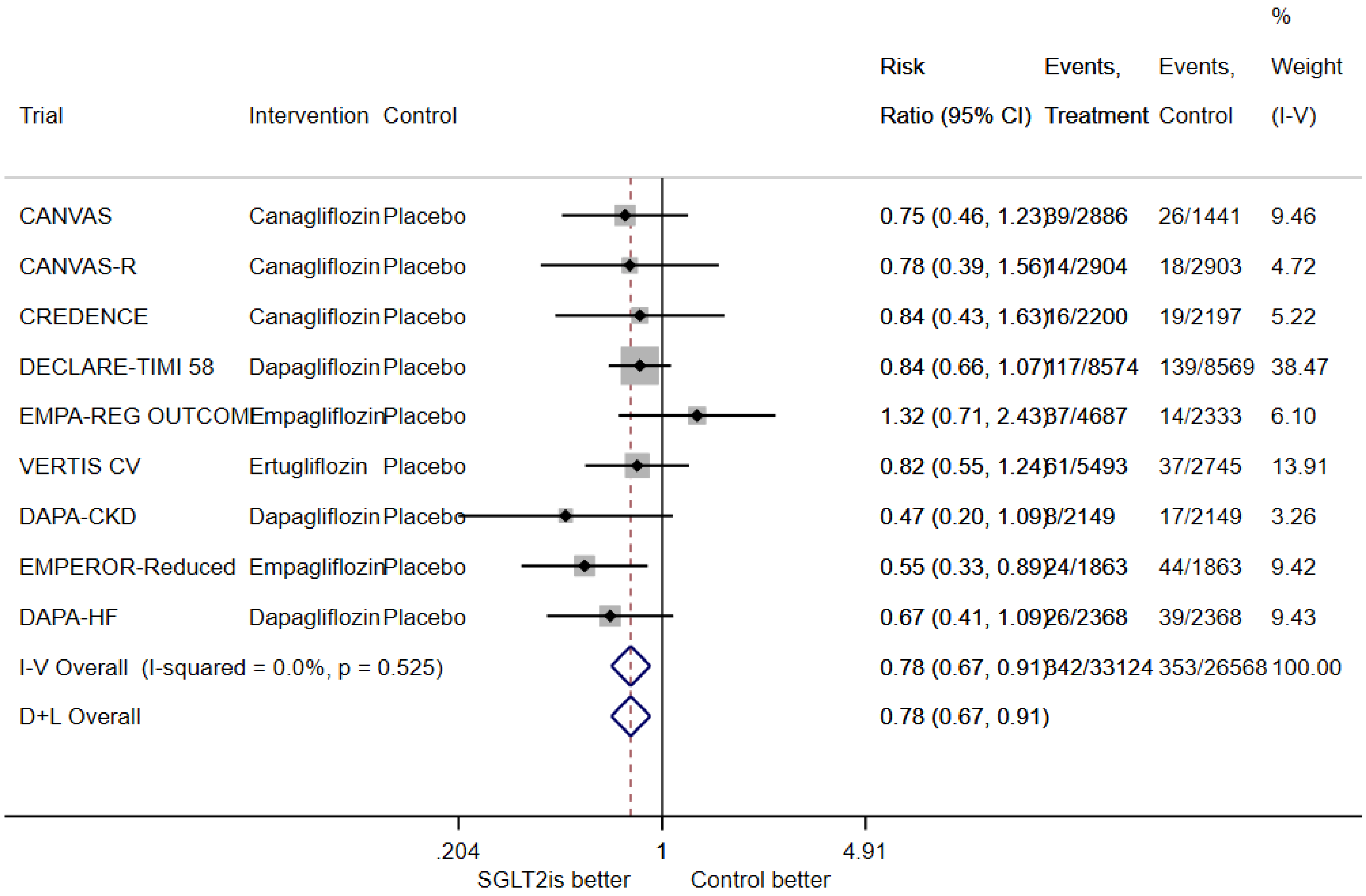

Figure S14 Meta-analysis of SGLT2is and Atrial fibrillation

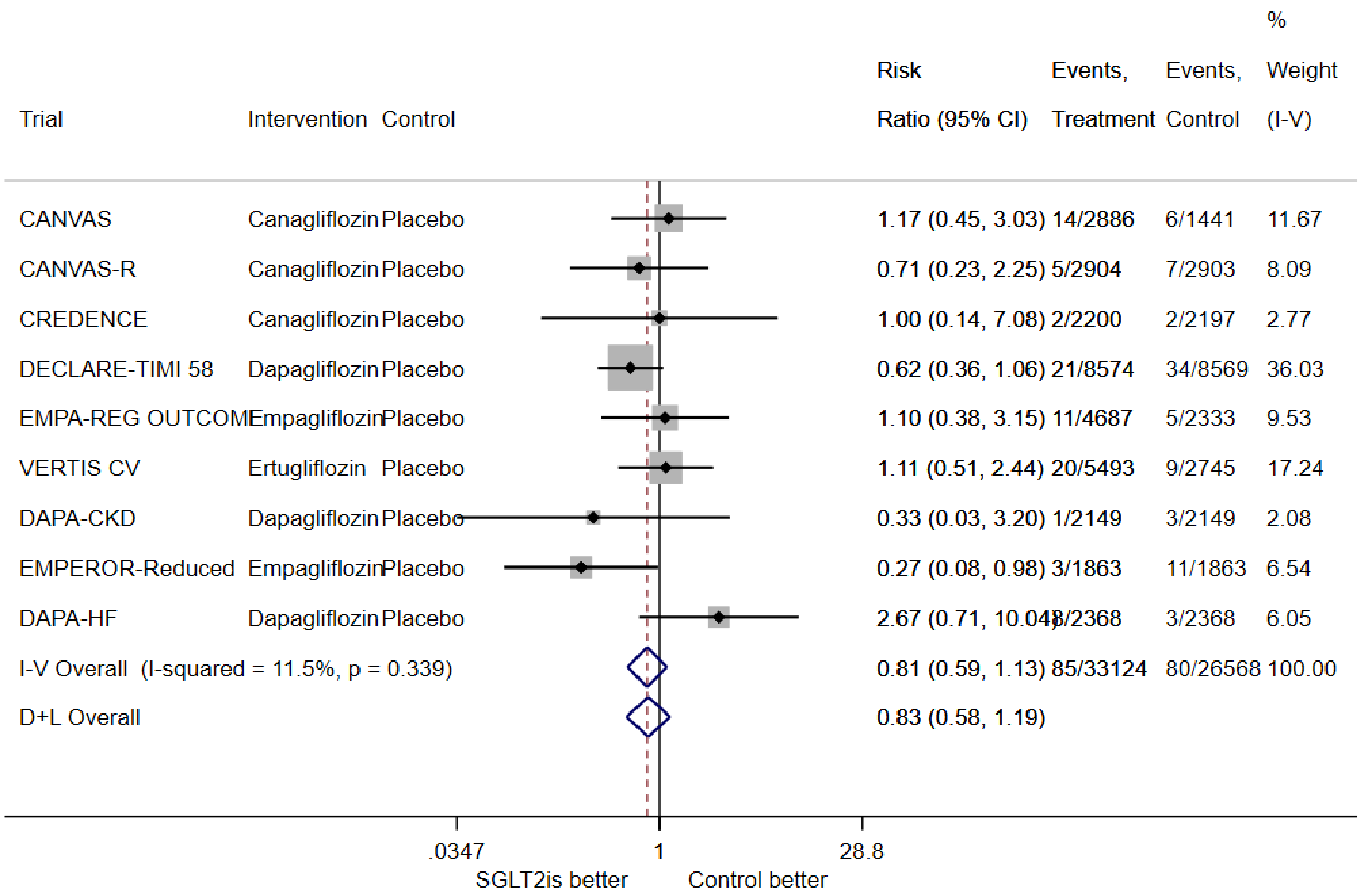

Figure S15 Meta-analysis of SGLT2is and Atrial flutter

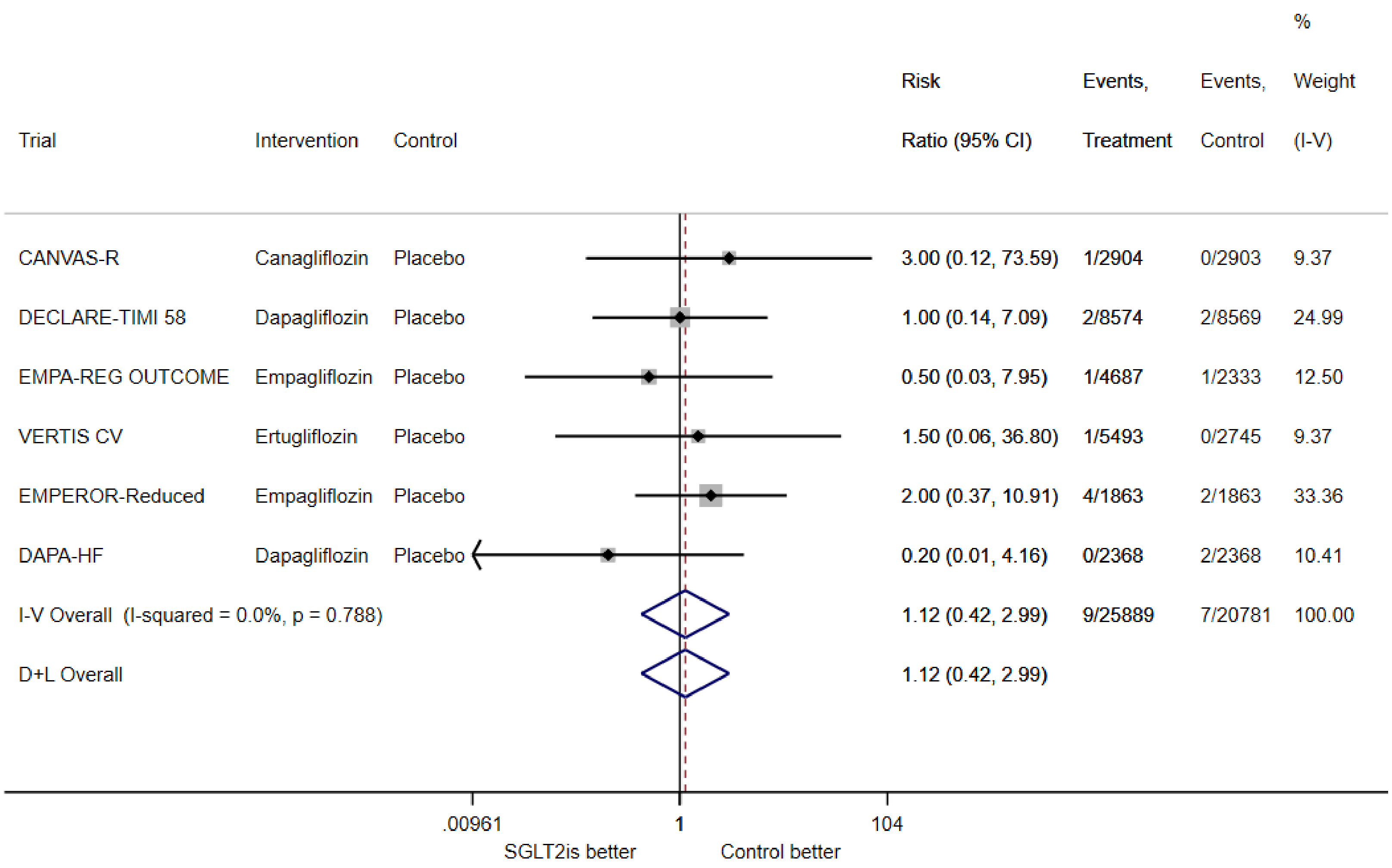

Figure S16 Meta-analysis of SGLT2is and Atrial tachycardia

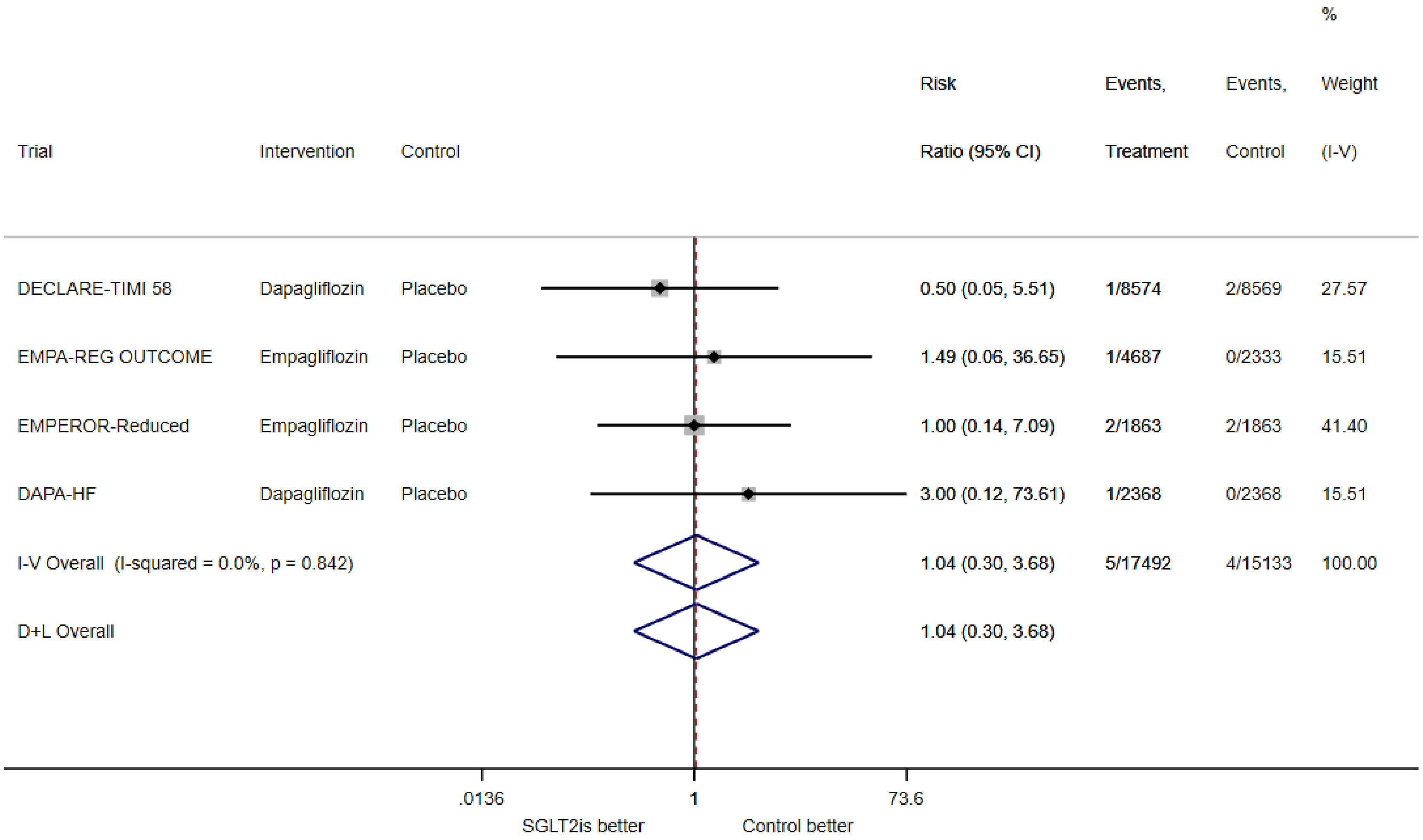

Figure S17 Meta-analysis of SGLT2is and Atrial thrombosis

Trial	Intervention	Control	Risk Ratio (95% CI)	Events, Treatment	Events, Control	% Weight (I-V)
CANVAS	Canagliflozin	Placebo	0.25 (0.02, 2.75)	1/2886	2/1441	9.45
CREDENCE	Canagliflozin	Placebo	1.00 (0.14, 7.08)	2/2200	2/2197	14.18
DECLARE-TIMI 58	Dapagliflozin	Placebo	0.29 (0.06, 1.37)	2/8574	7/8569	22.04
EMPA-REG OUTCOME	Empagliflozin	Placebo	0.50 (0.10, 2.46)	3/4687	3/2333	21.26
VERTIS CV	Ertugliflozin	Placebo	1.00 (0.18, 5.45)	4/5493	2/2745	18.90
DAPA-CKD	Dapagliflozin	Placebo	1.00 (0.14, 7.09)	2/2149	2/2149	14.18
I-V Overall (I-squared = 0.0%, p = 0.810)			0.57 (0.27, 1.20)	14/25989	18/19434	100.00
D+L Overall			0.57 (0.27, 1.20)			

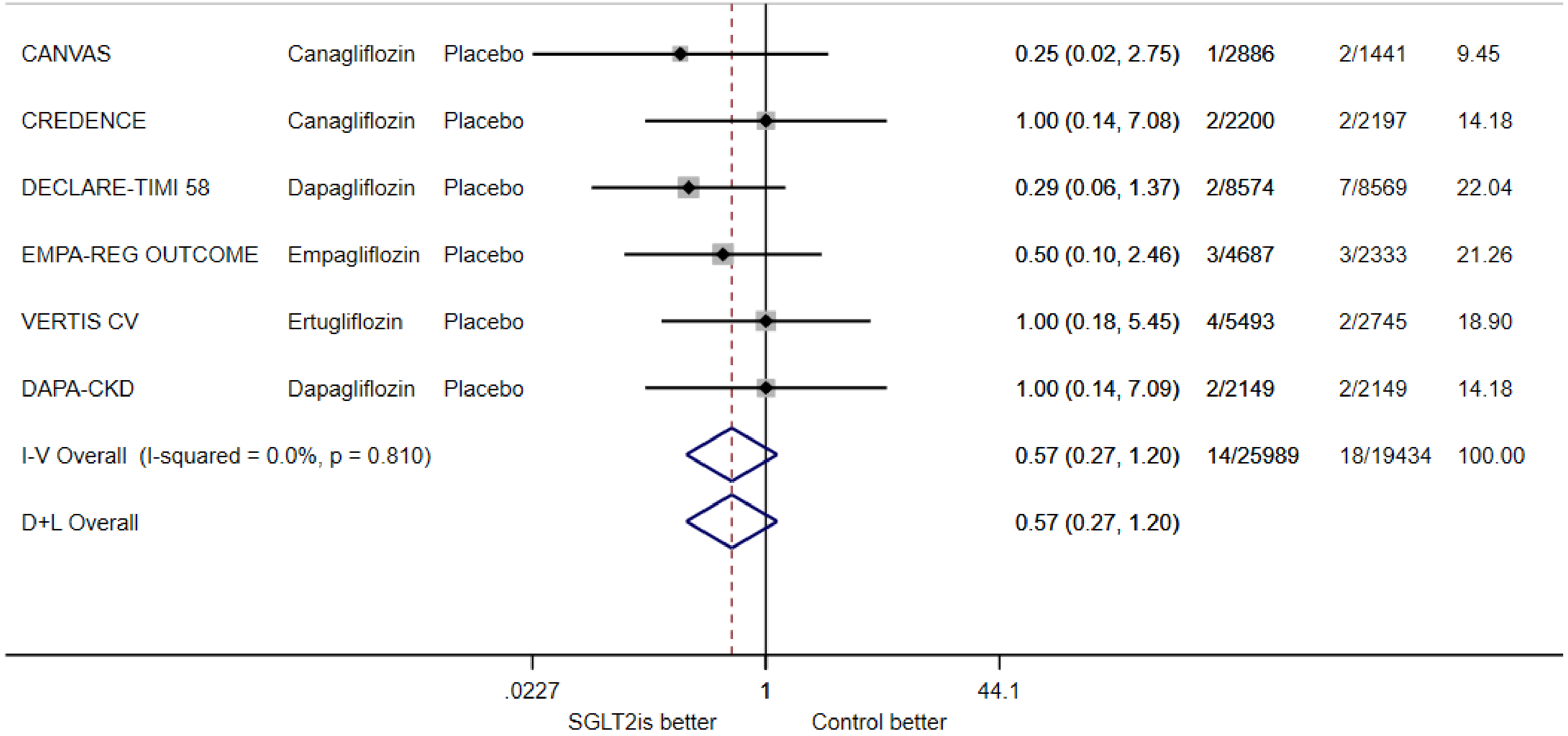

Figure S18 Meta-analysis of SGLT2is and Atrioventricular block

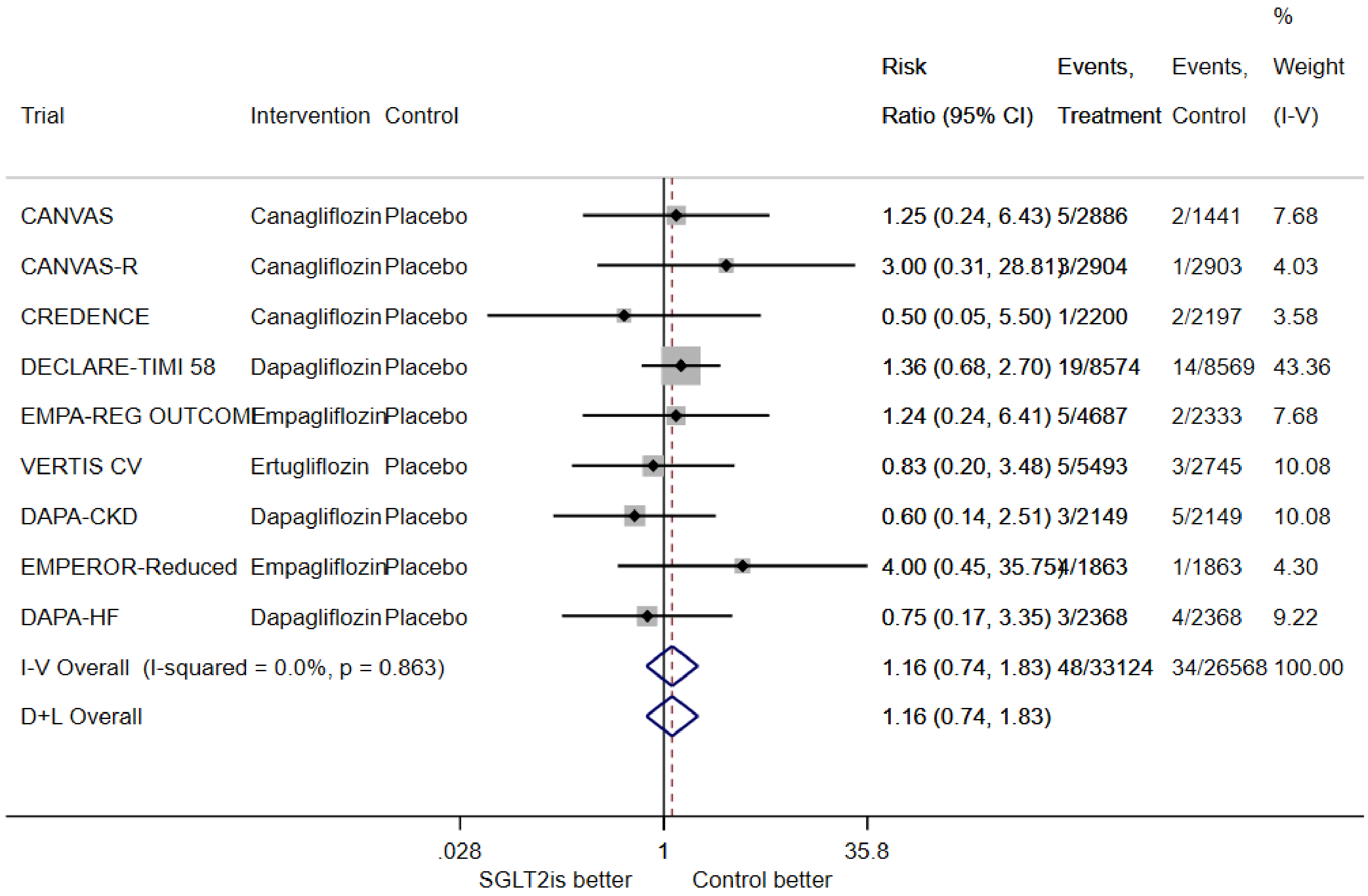

Figure S19 Meta-analysis of SGLT2is and Atrioventricular block complete

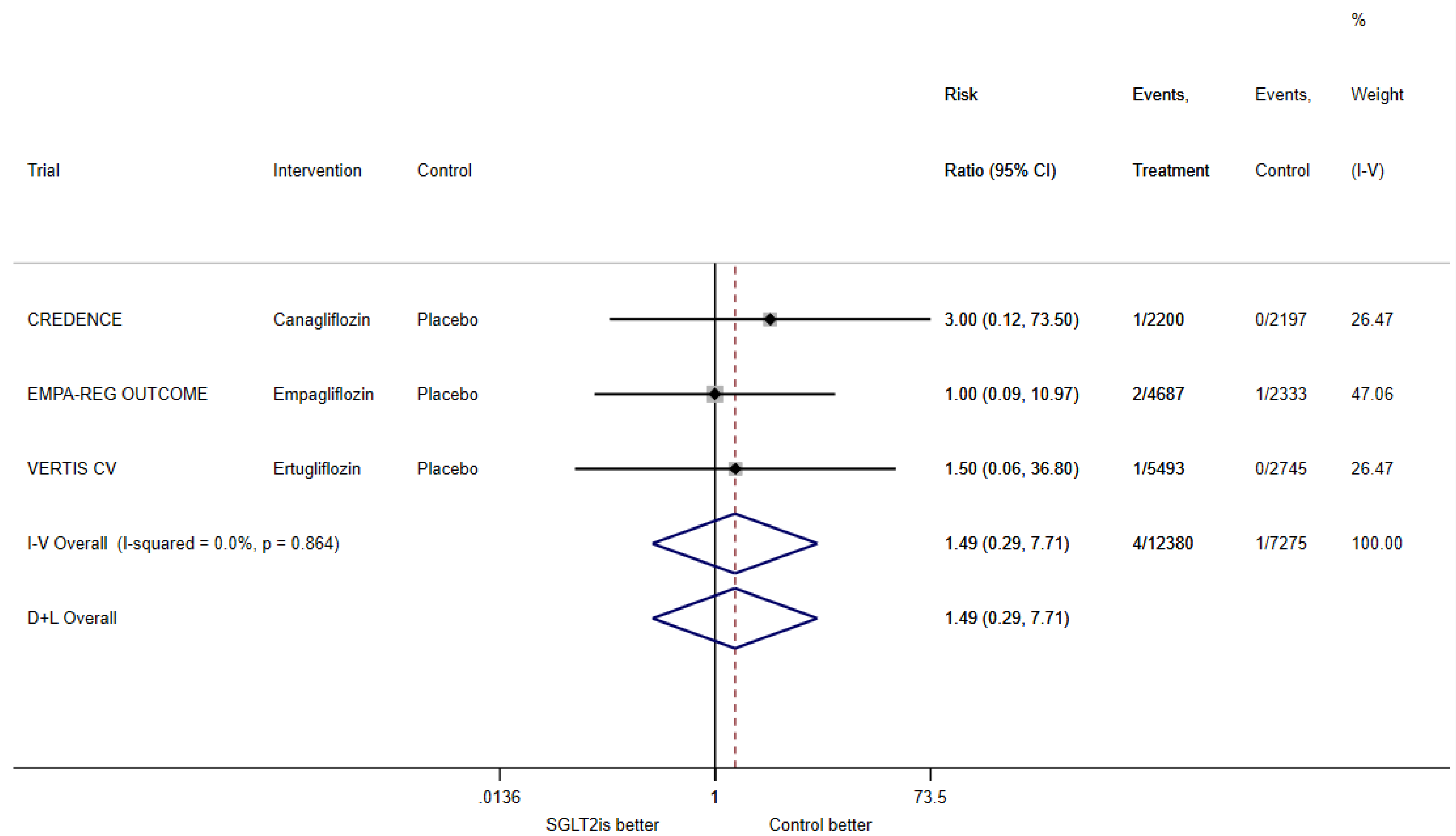

Figure S20 Meta-analysis of SGLT2is and Atrioventricular block first degree

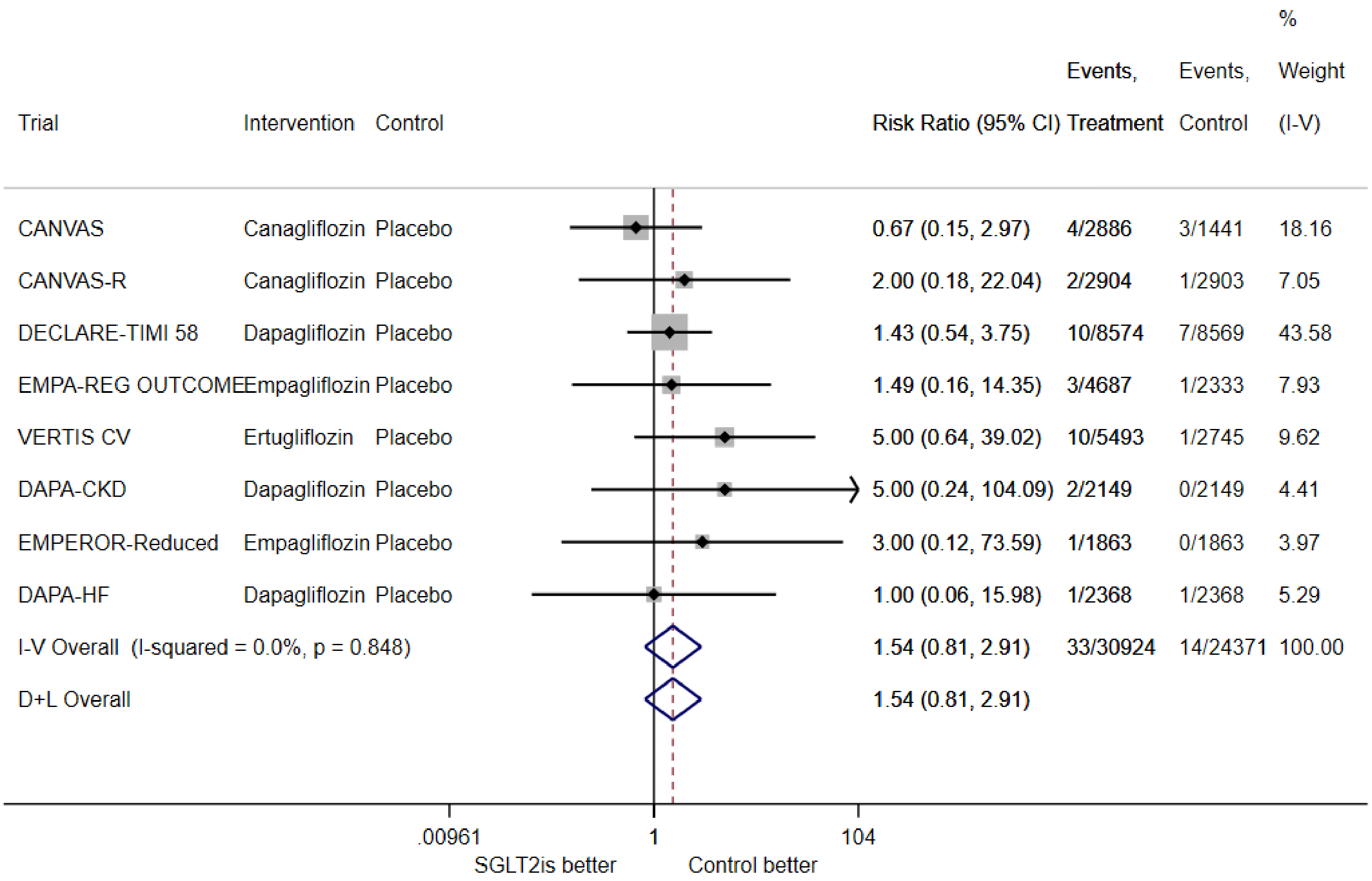

Figure S21 Meta-analysis of SGLT2is and Atrioventricular block second degree

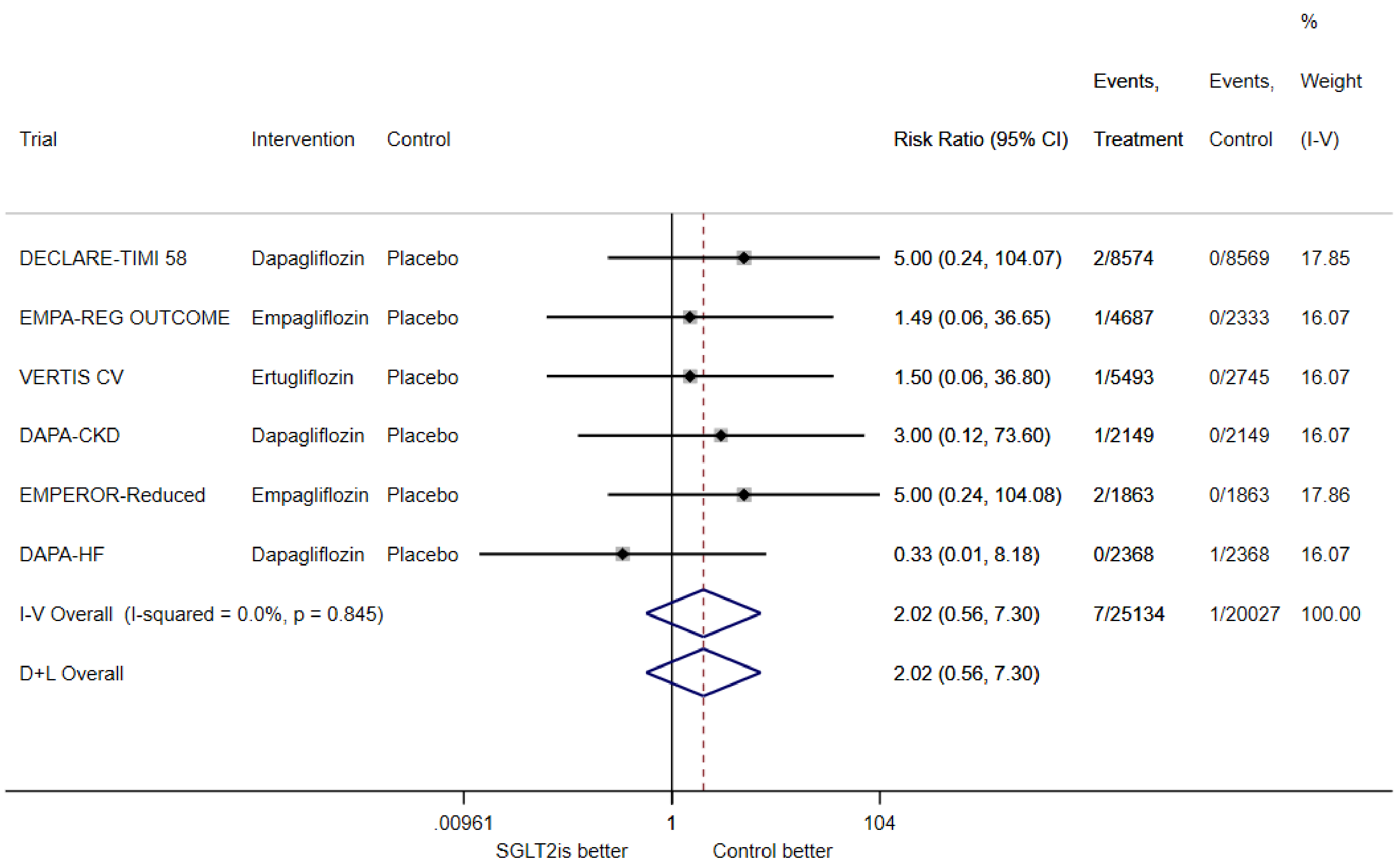

Figure S22 Meta-analysis of SGLT2is and Bradyarrhythmia

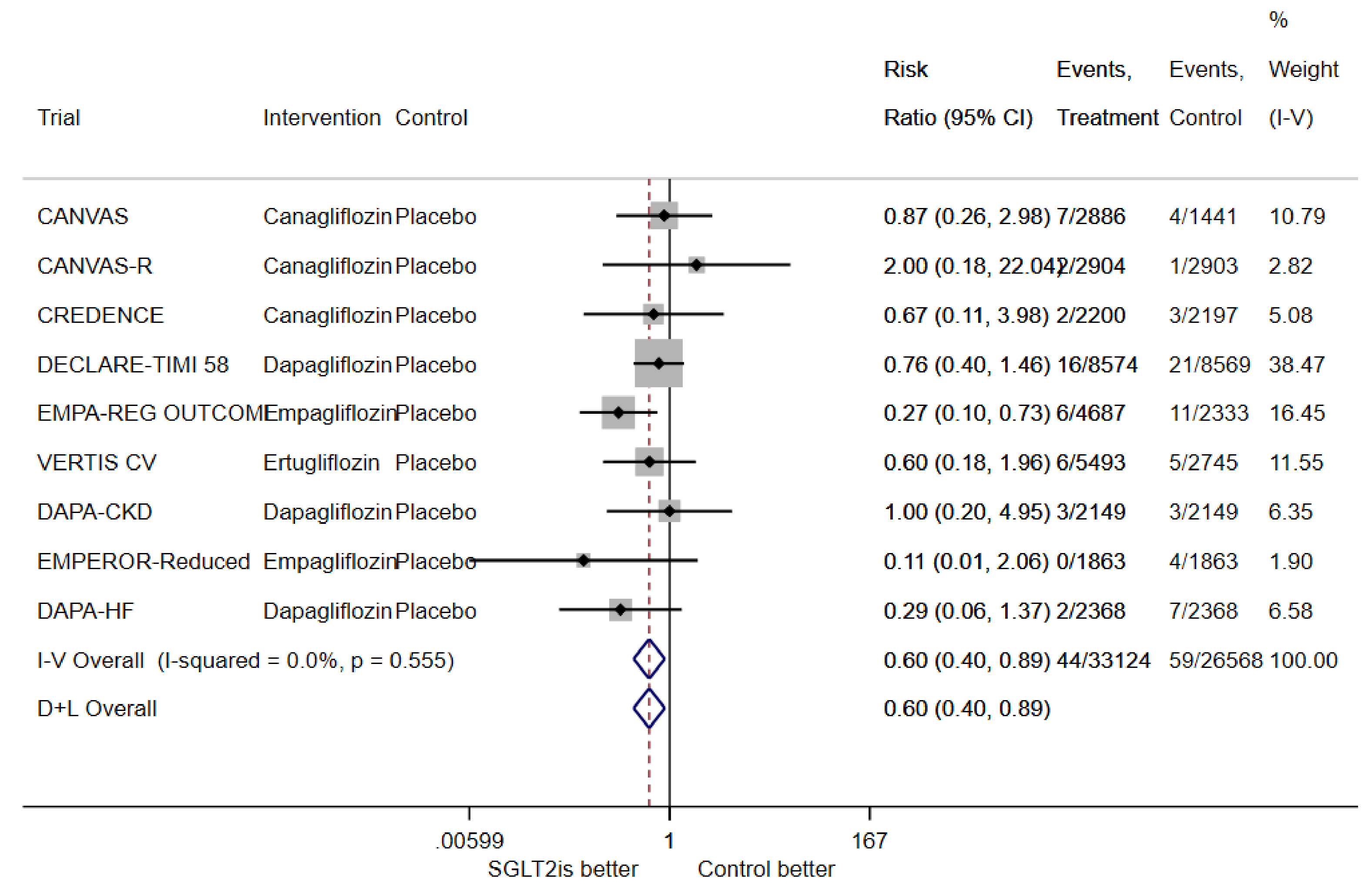

Figure S23 Meta-analysis of SGLT2is and Bradycardia

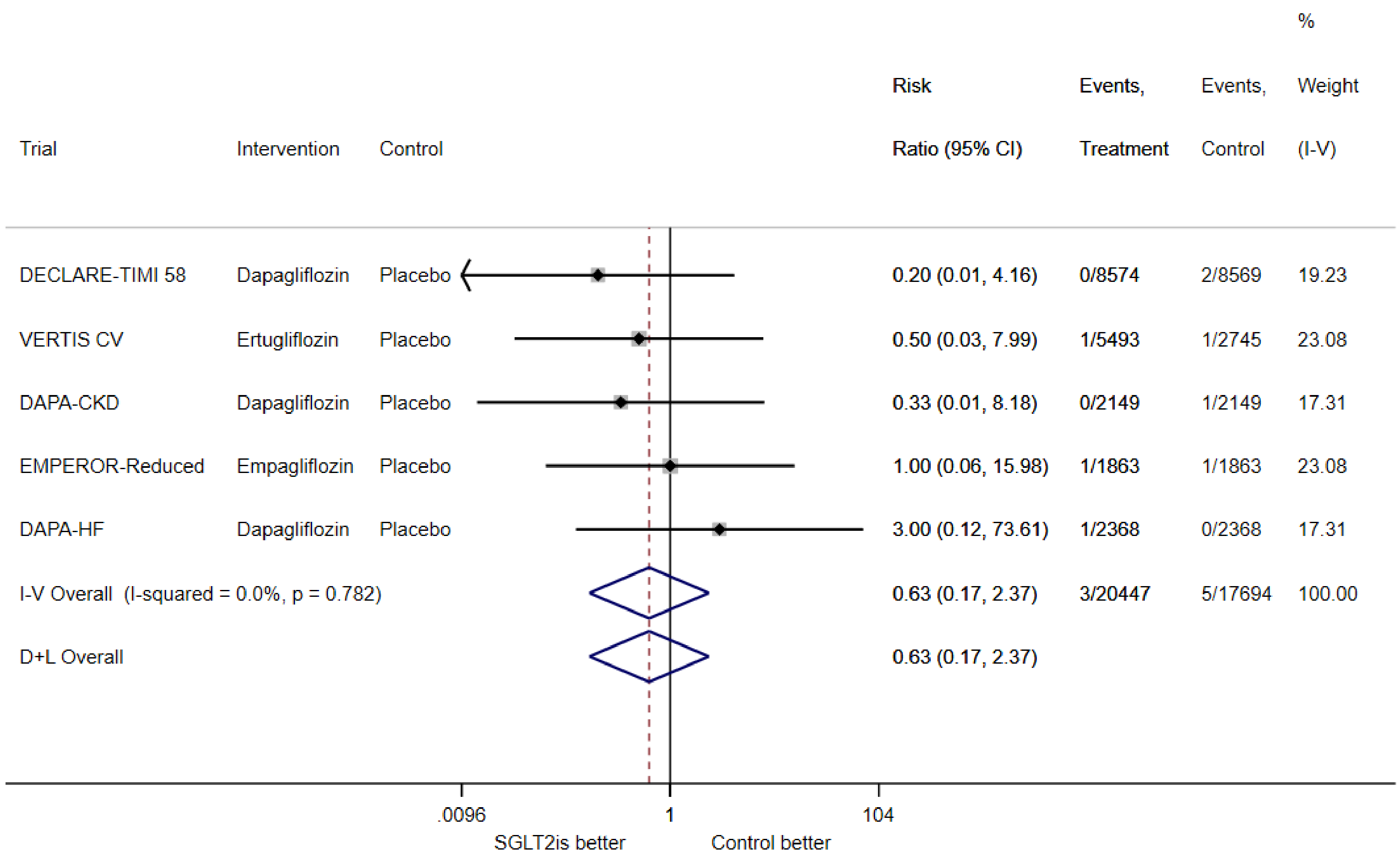

Figure S24 Meta-analysis of SGLT2is and Bundle branch block left

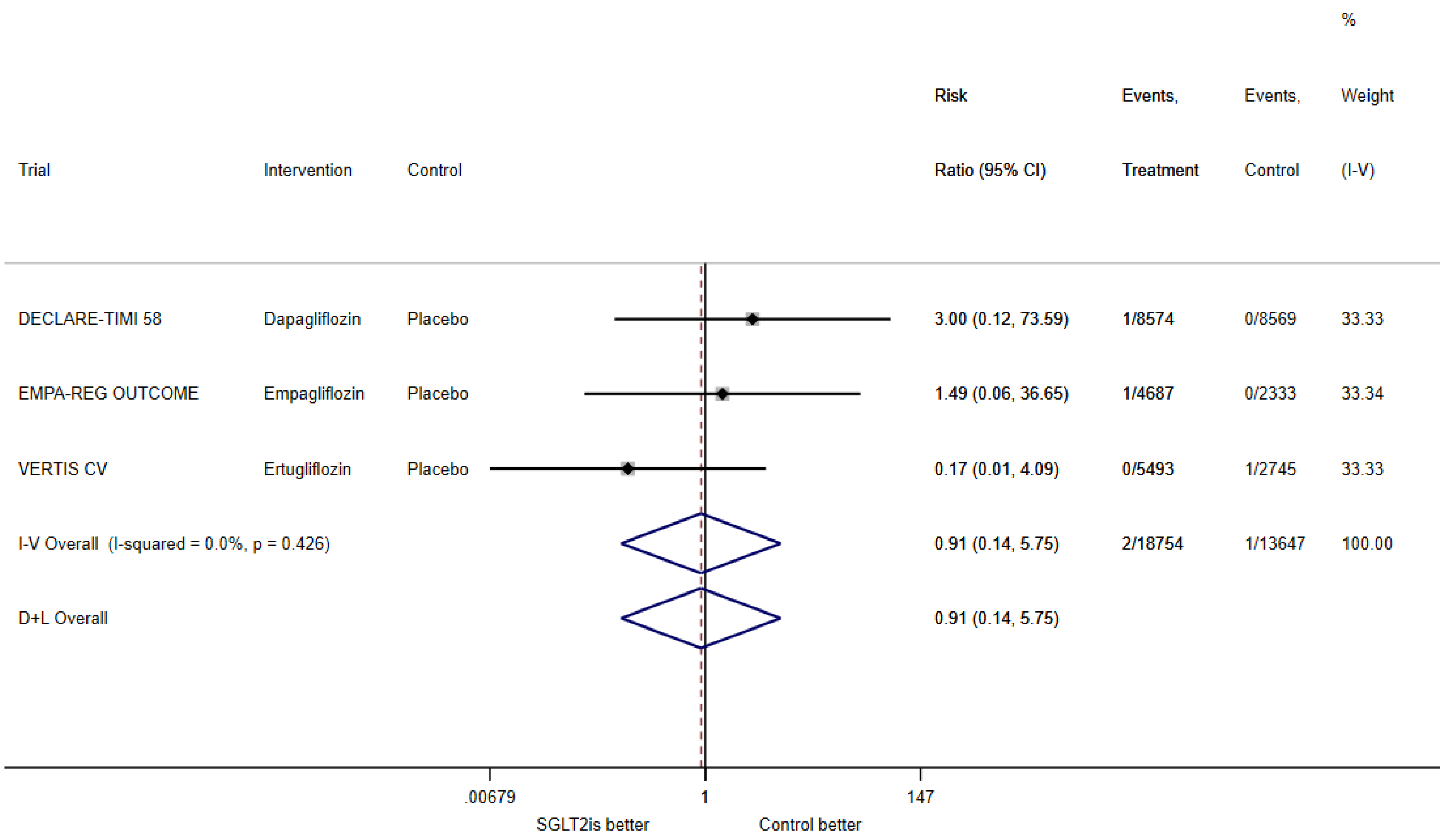

Figure S25 Meta-analysis of SGLT2is and Bundle branch block right

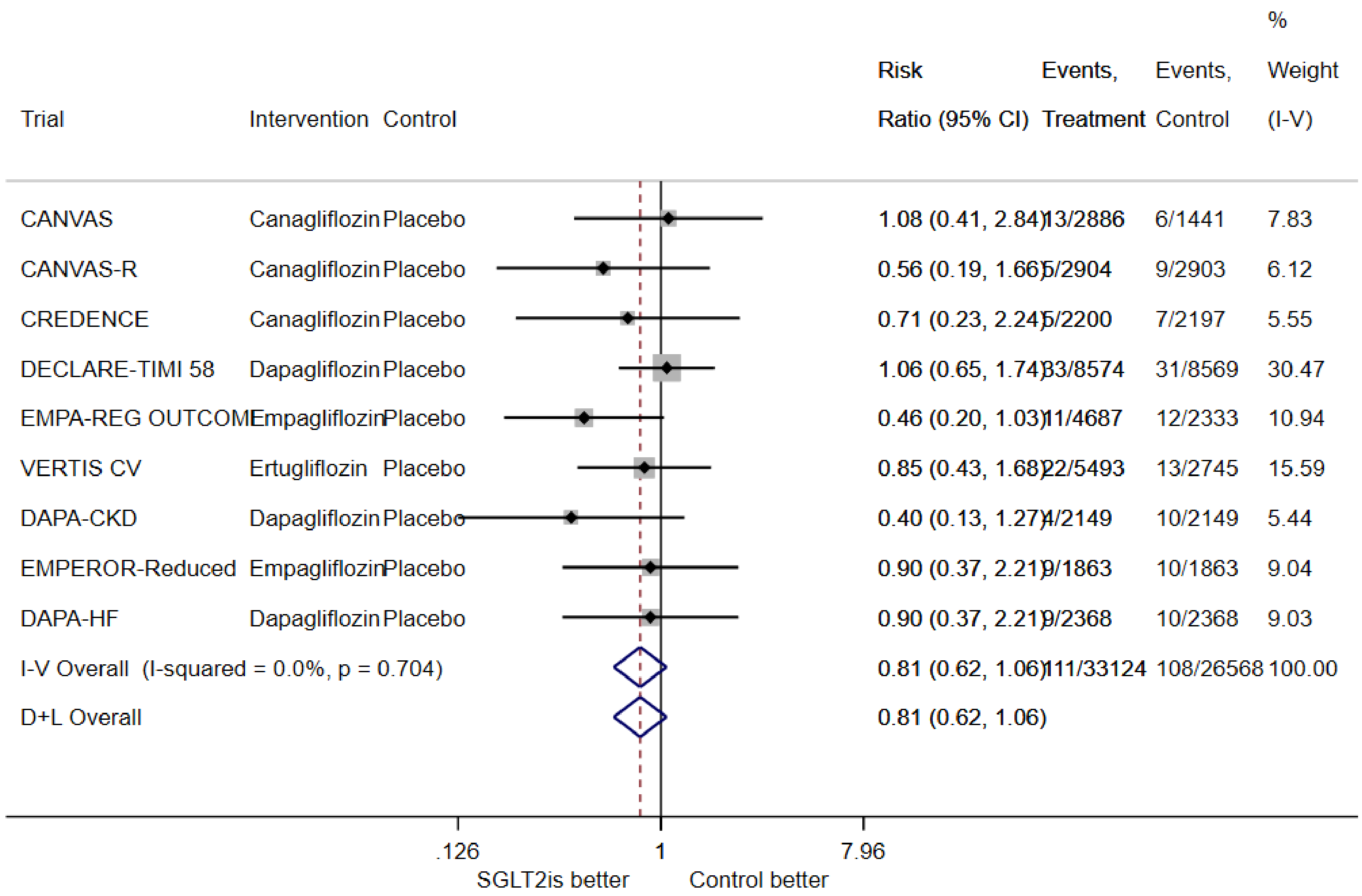

Figure S26 Meta-analysis of SGLT2is and Cardiac arrest

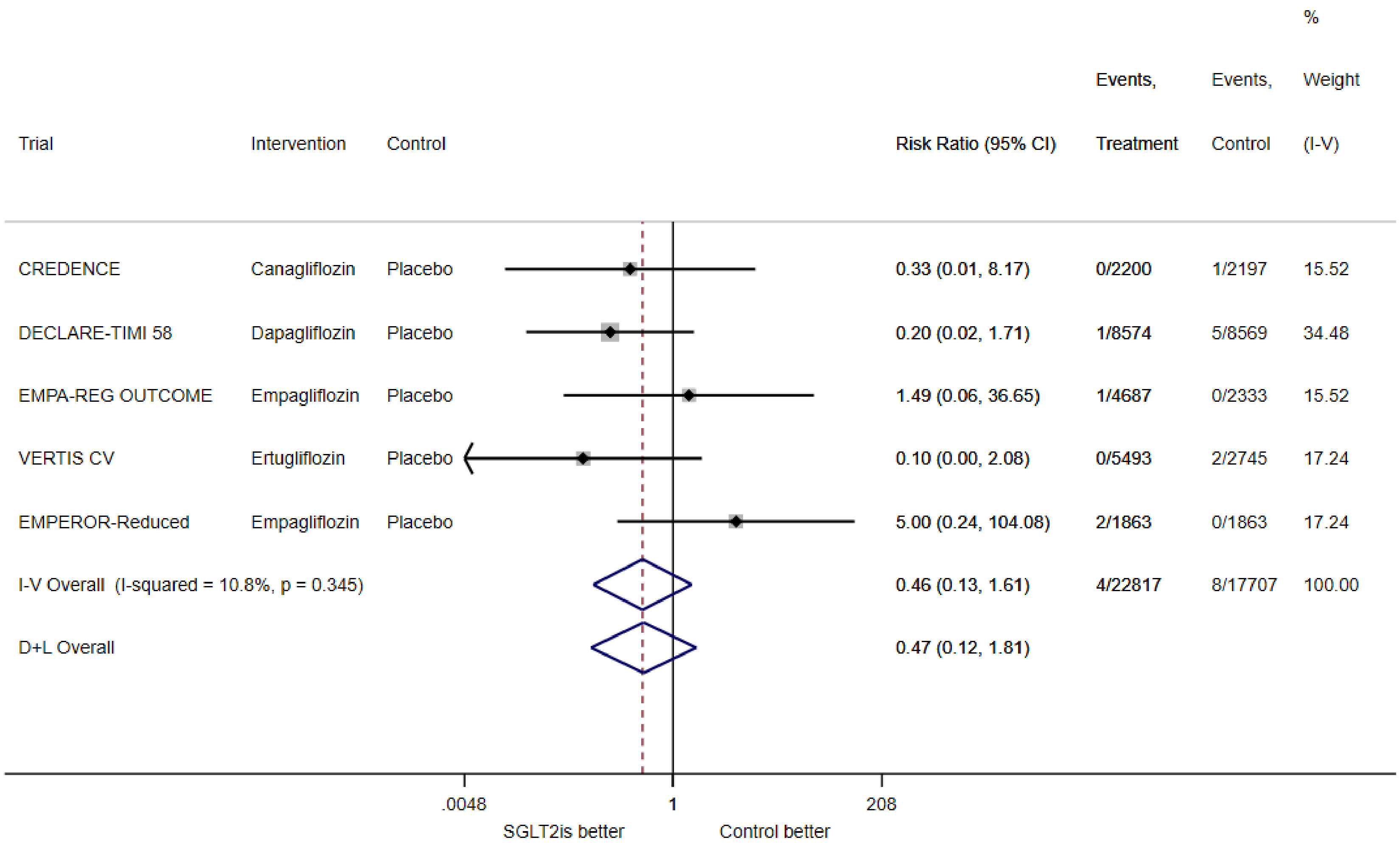

Figure S27 Meta-analysis of SGLT2is and Cardiac asthma

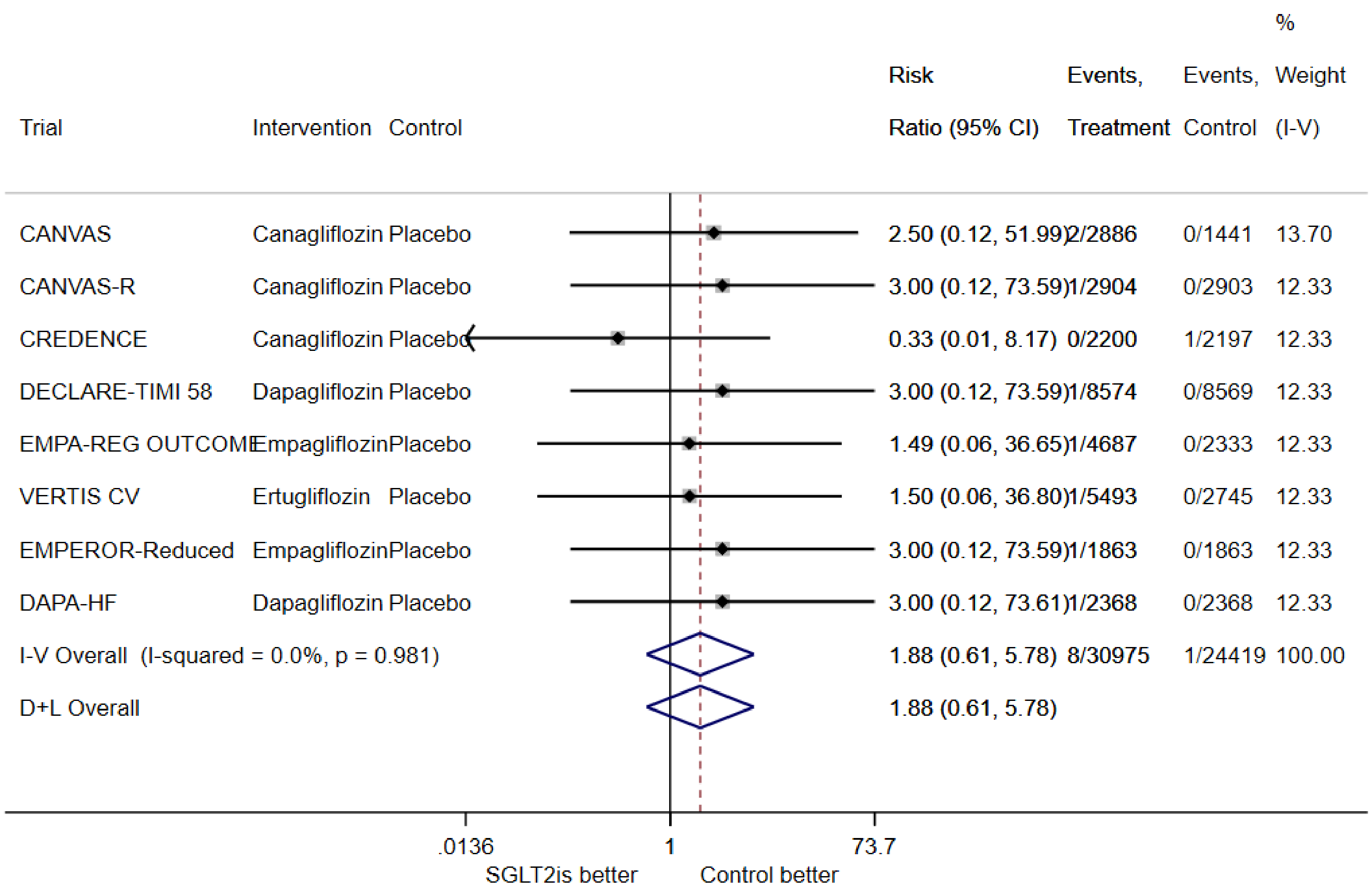

Figure S28 Meta-analysis of SGLT2is and Cardiac disorder

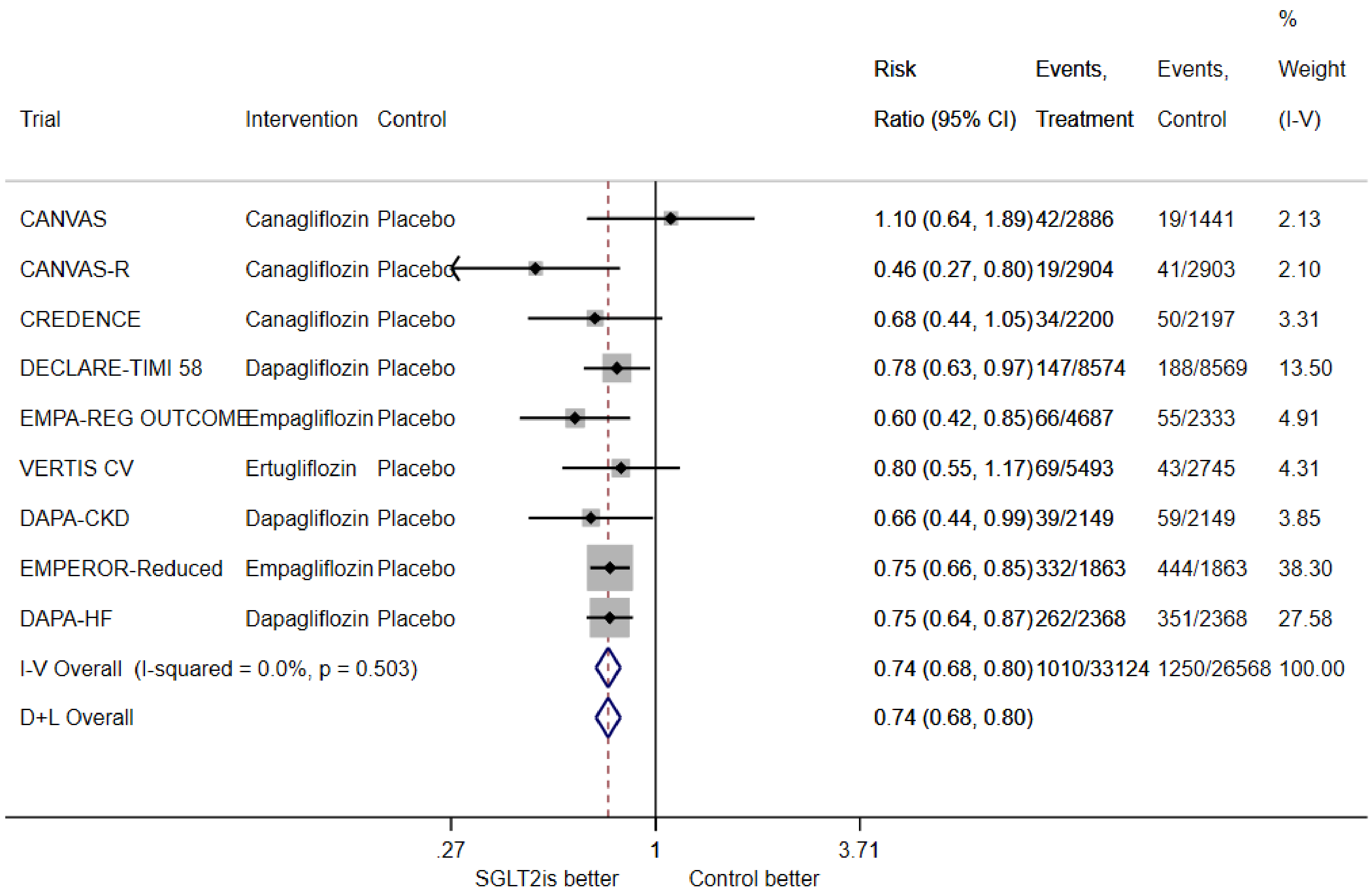

Figure S29 Meta-analysis of SGLT2is and Cardiac failure

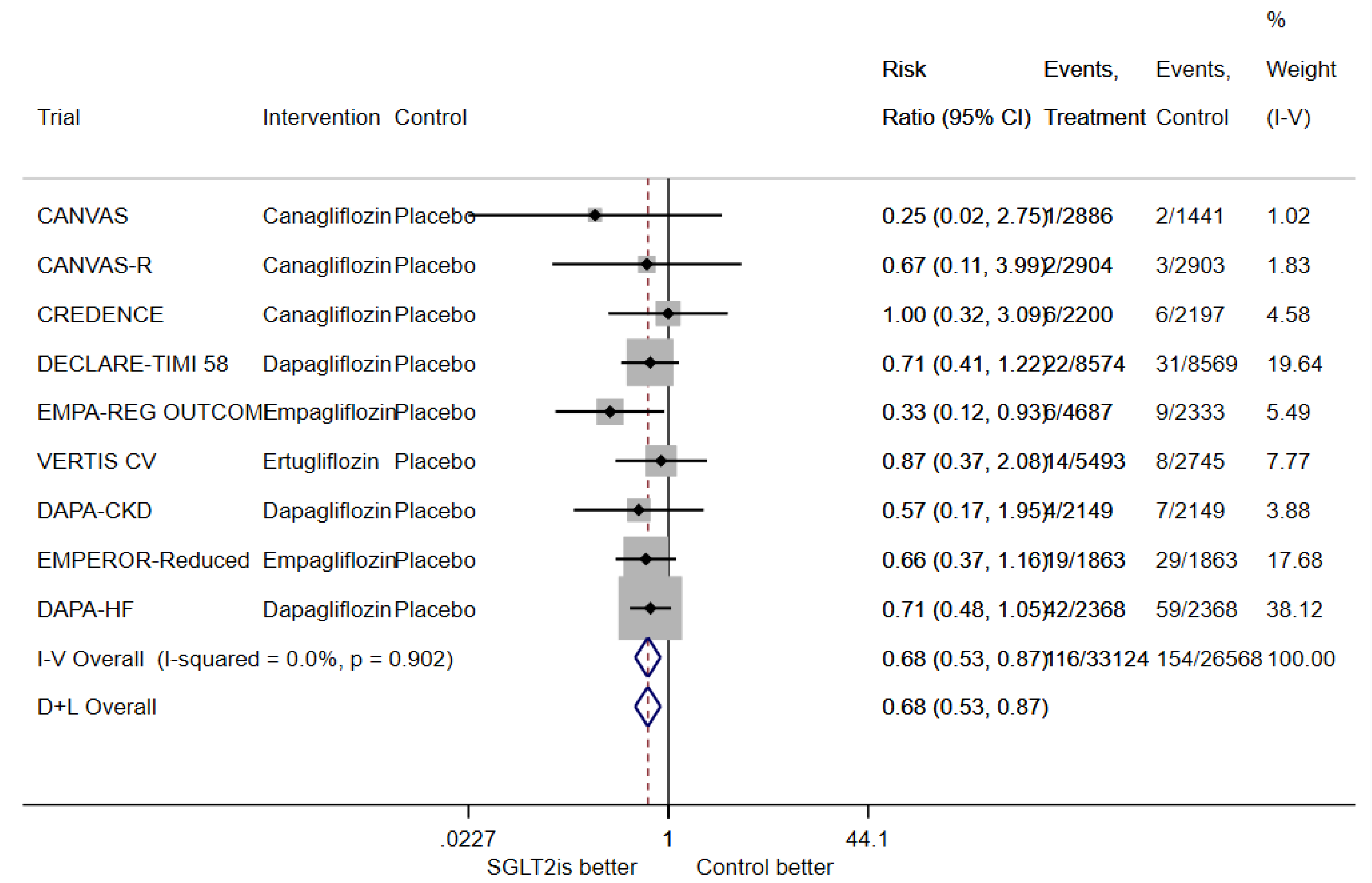

Figure S30 Meta-analysis of SGLT2is and Cardiac failure acute

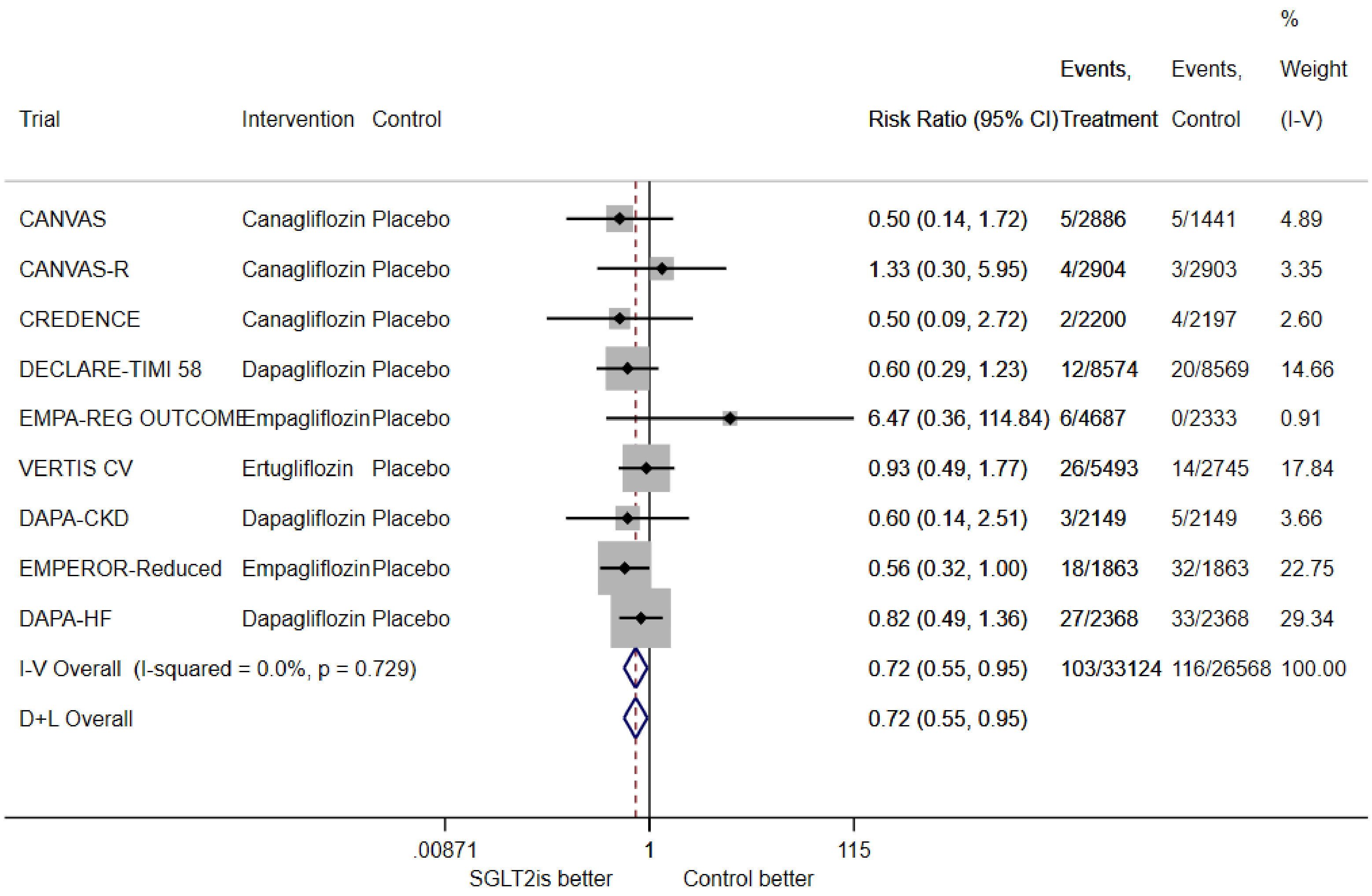

Figure S31 Meta-analysis of SGLT2is and Cardiac failure chronic

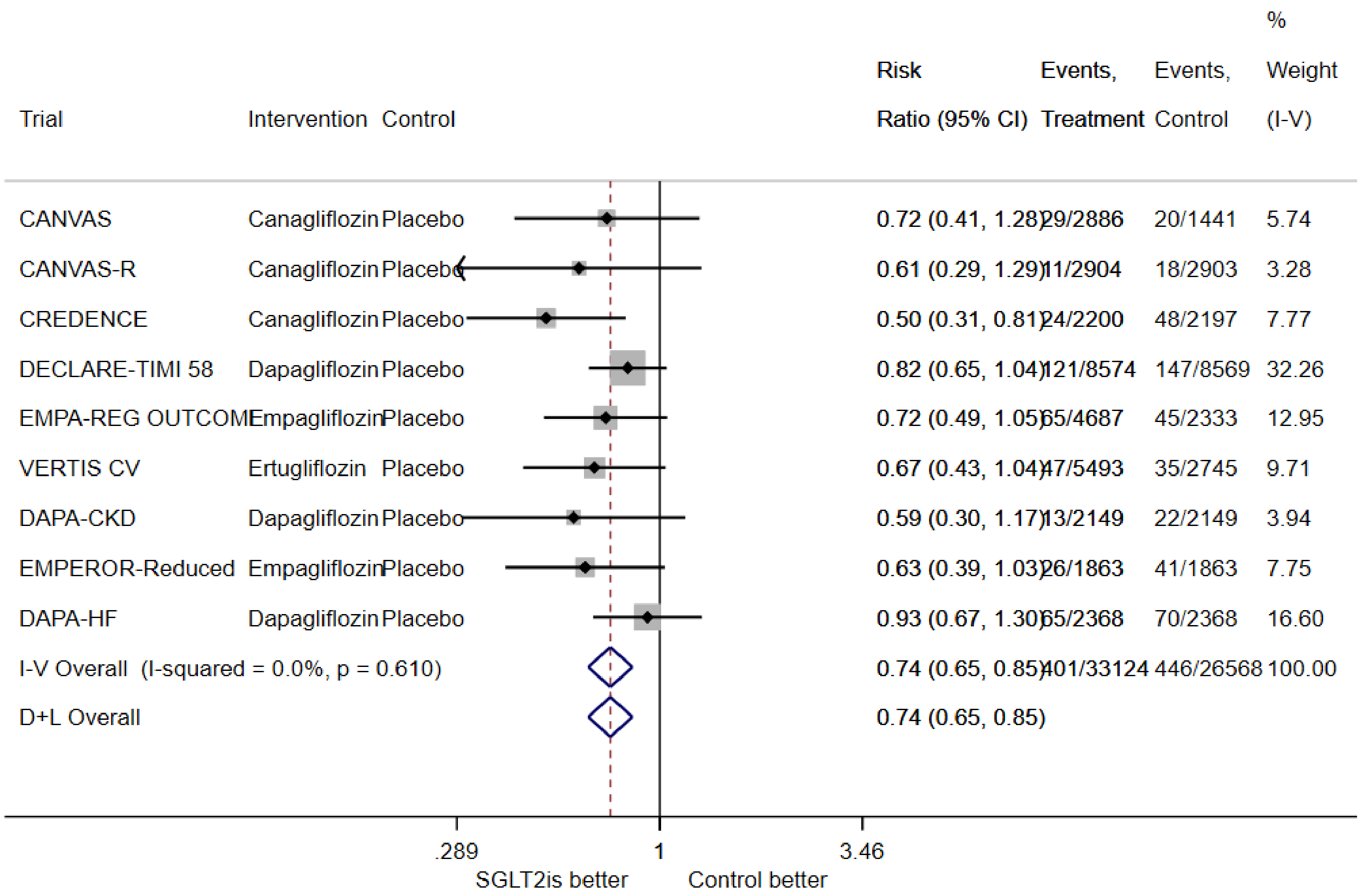

Figure S32 Meta-analysis of SGLT2is and Cardiac failure congestive

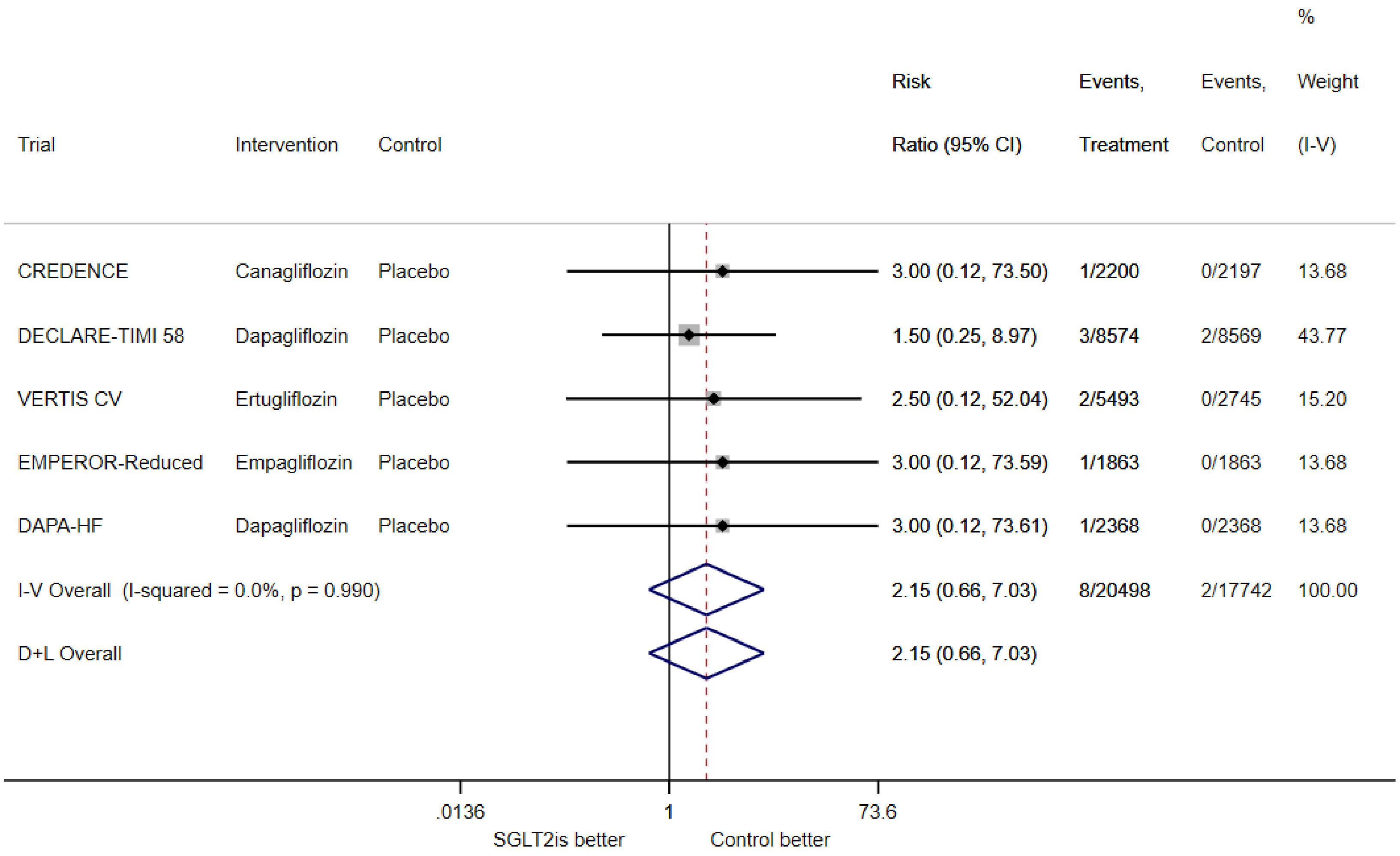

Figure S33 Meta-analysis of SGLT2is and Cardiac tamponade

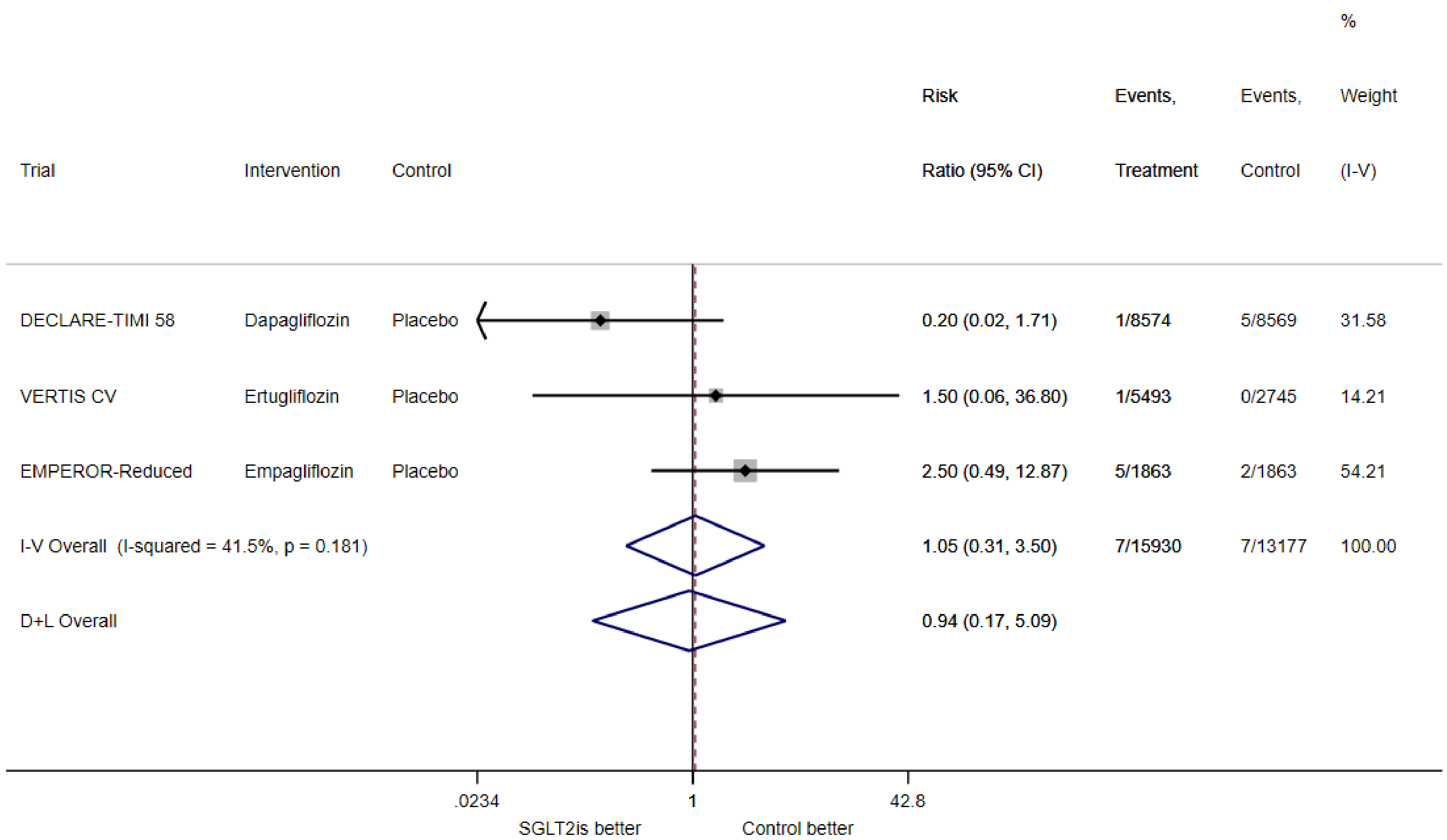

Figure S34 Meta-analysis of SGLT2is and Cardiac ventricular thrombosis

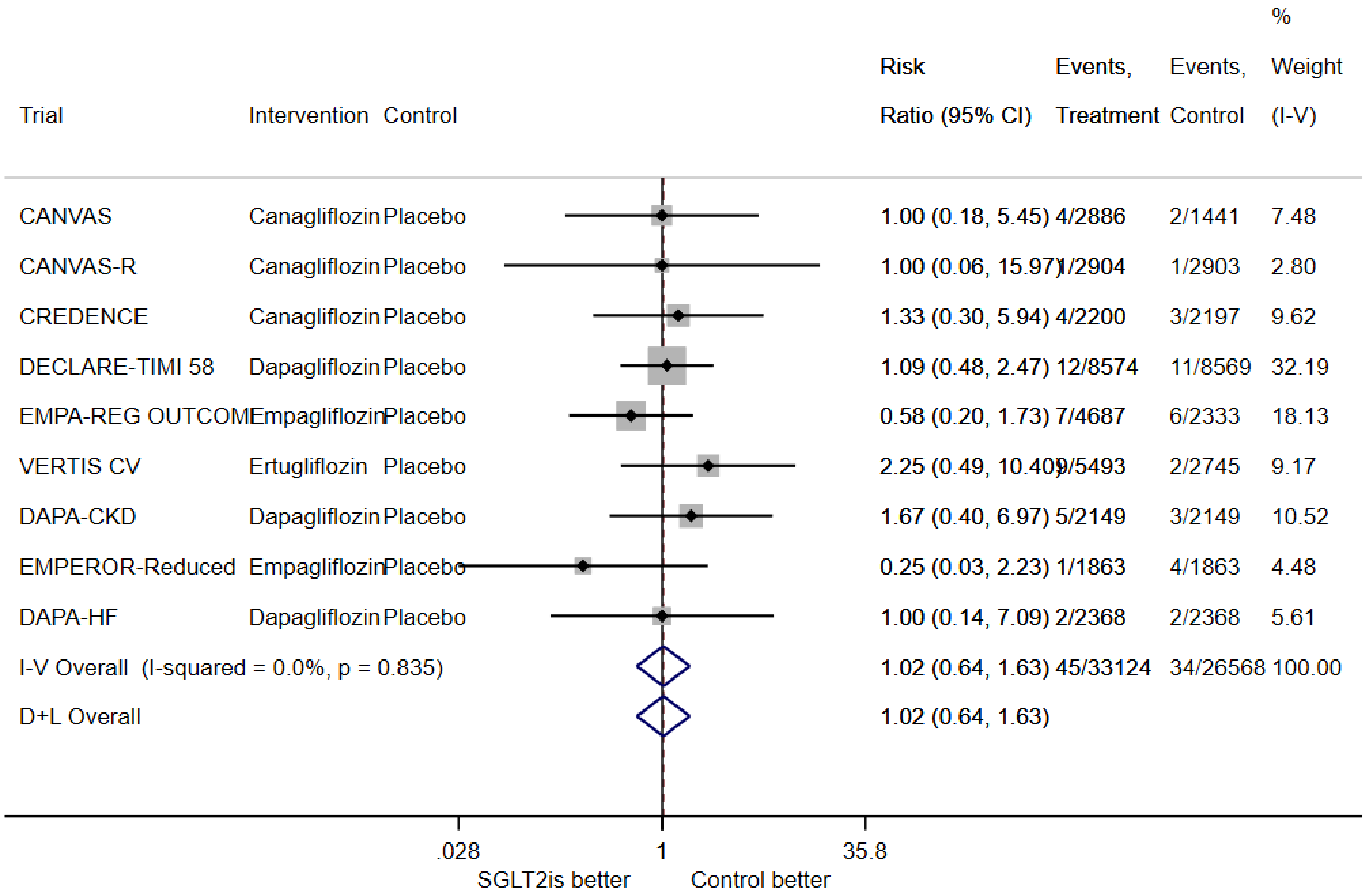

Figure S35 Meta-analysis of SGLT2is and Cardio-respiratory arrest

Trial	Intervention	Control	Risk Ratio (95% CI)	Events, Treatment	Events, Control	% Weight (I-V)
CANVAS	Canagliflozin	Placebo	6.49 (0.37, 115.18)	6/2886	0/1441	1.78
CANVAS-R	Canagliflozin	Placebo	0.20 (0.01, 4.16)	0/2904	2/2903	1.60
CREDENCE	Canagliflozin	Placebo	0.25 (0.05, 1.17)	2/2200	8/2197	6.14
DECLARE-TIMI 58	Dapagliflozin	Placebo	1.46 (0.72, 2.96)	19/8574	13/8569	29.63
EMPA-REG OUTCOME	Empagliflozin	Placebo	1.00 (0.18, 5.43)	4/4687	2/2333	5.11
VERTIS CV	Ertugliflozin	Placebo	0.44 (0.16, 1.20)	7/5493	8/2745	14.33
DAPA-CKD	Dapagliflozin	Placebo	1.00 (0.06, 15.98)	1/2149	1/2149	1.92
EMPEROR-Reduced	Empagliflozin	Placebo	0.73 (0.29, 1.80)	8/1863	11/1863	17.84
DAPA-HF	Dapagliflozin	Placebo	0.60 (0.26, 1.37)	9/2368	15/2368	21.66
I-V Overall (I-squared = 18.0%, p = 0.283)			0.78 (0.53, 1.14)	56/33124	60/26568	100.00
D+L Overall			0.75 (0.48, 1.18)			

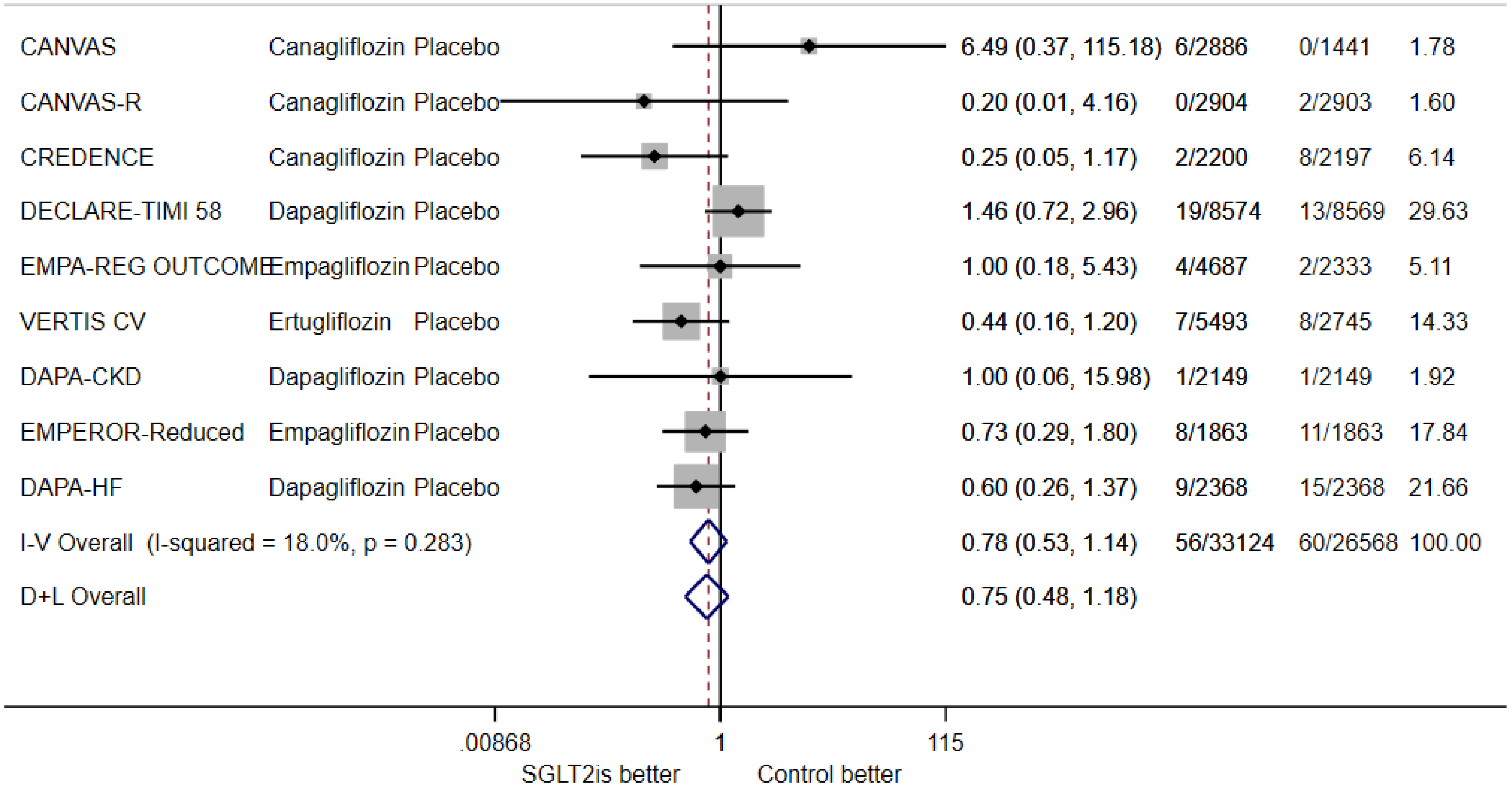

Figure S36 Meta-analysis of SGLT2is and Cardiogenic shock

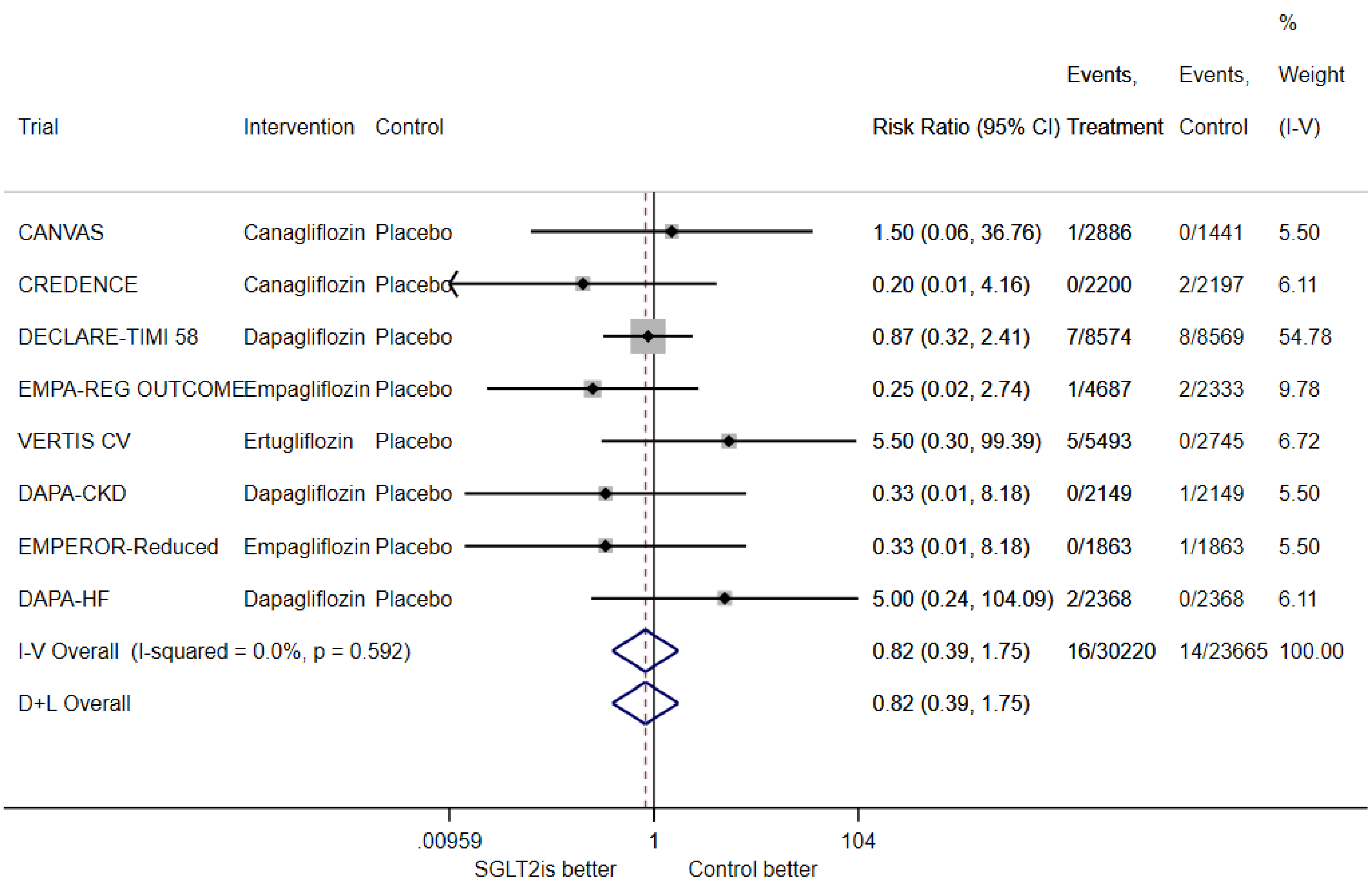

Figure S37 Meta-analysis of SGLT2is and Cardiomyopathy

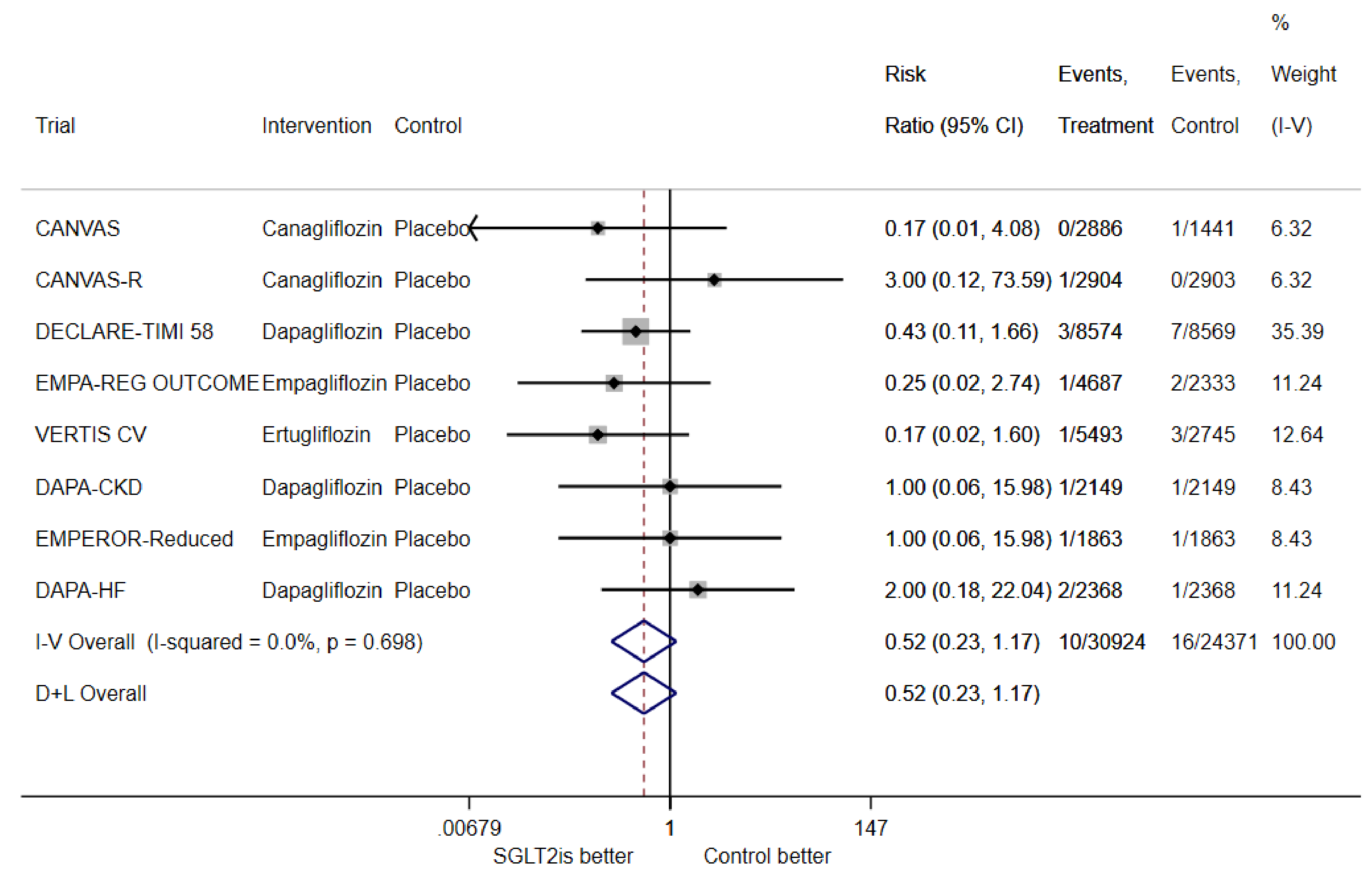

Figure S38 Meta-analysis of SGLT2is and Cardiopulmonary failure

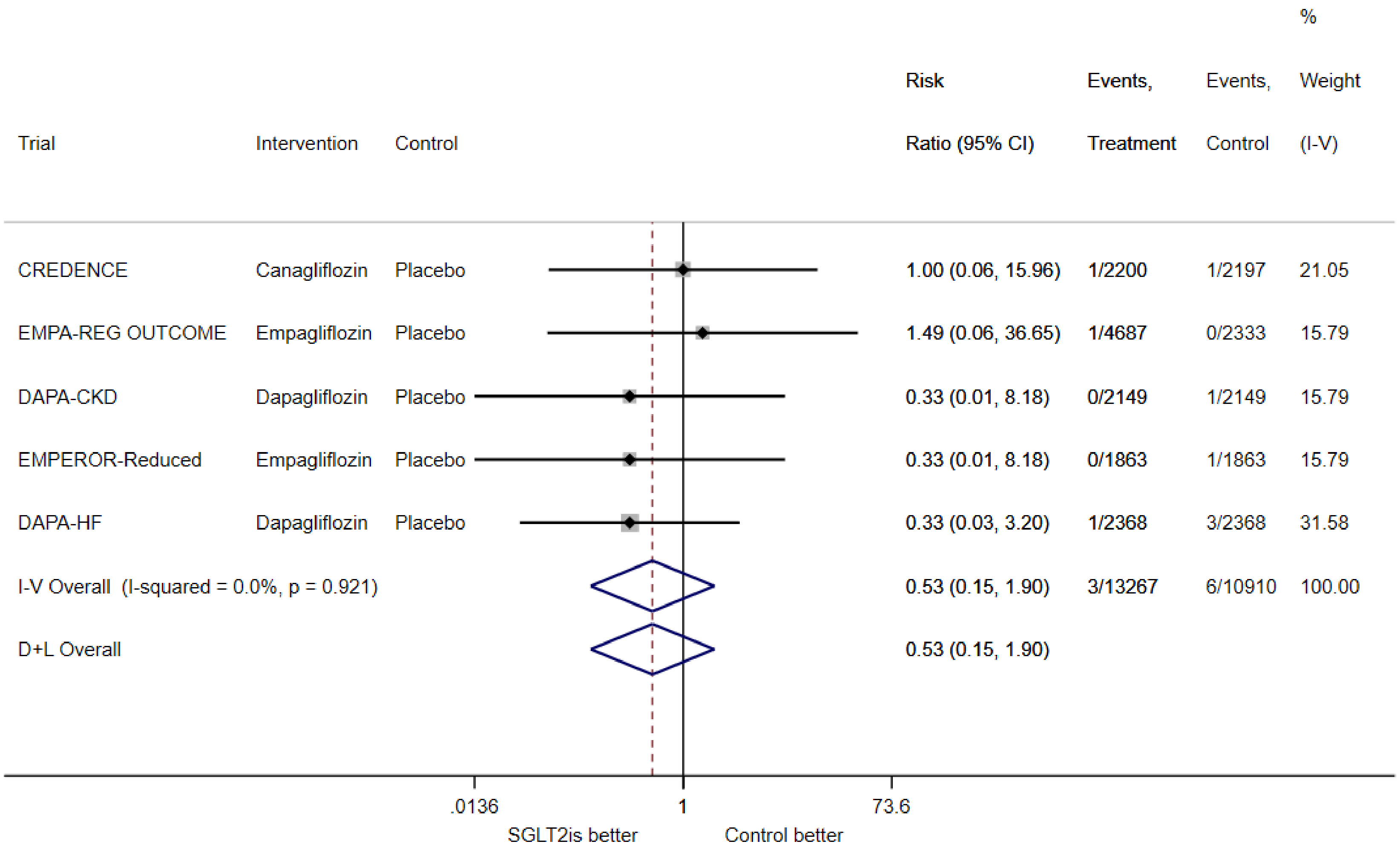

Figure S39 Meta-analysis of SGLT2is and Cardiorenal syndrome

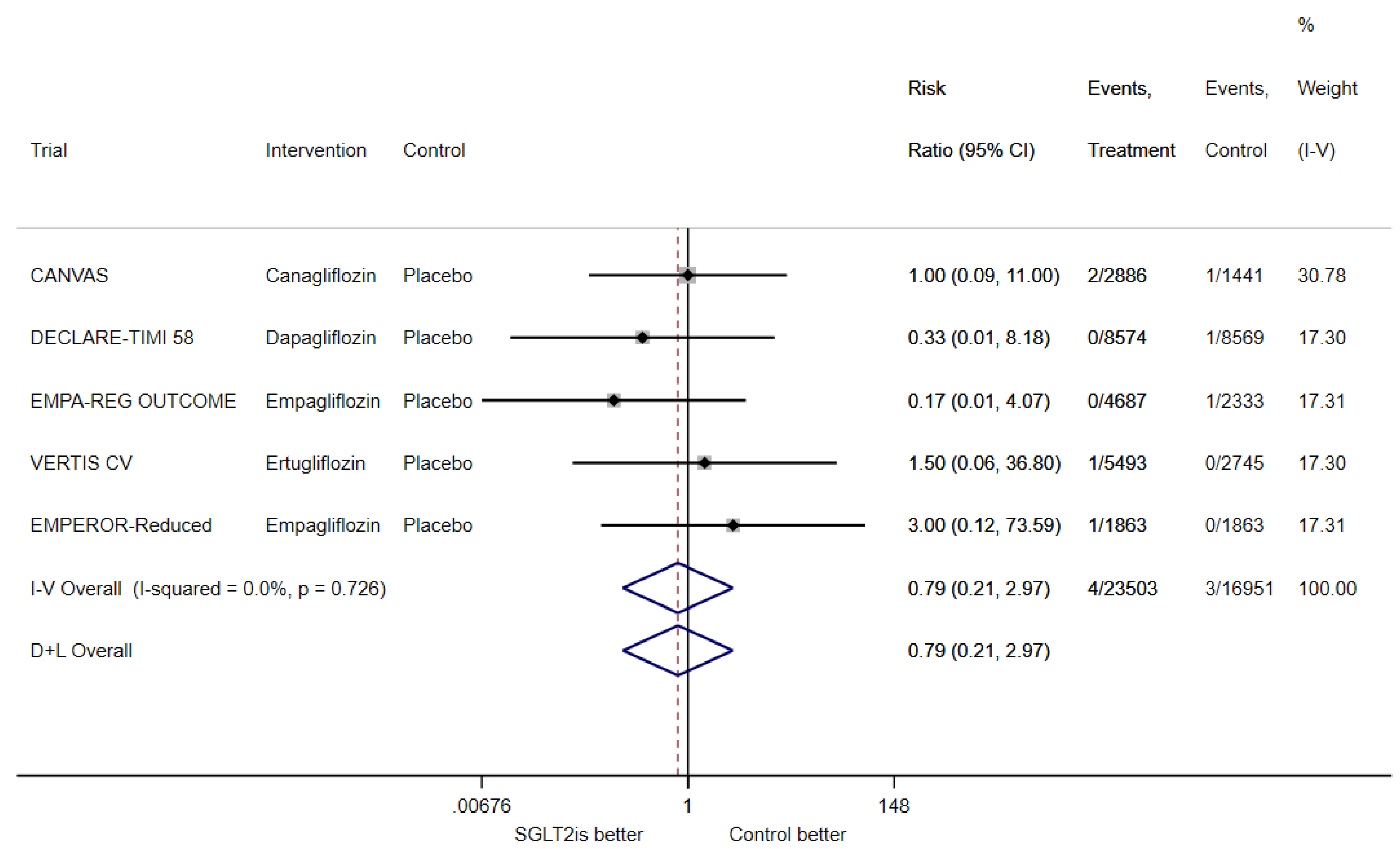

Figure S40 Meta-analysis of SGLT2is and Cardiovascular disorder

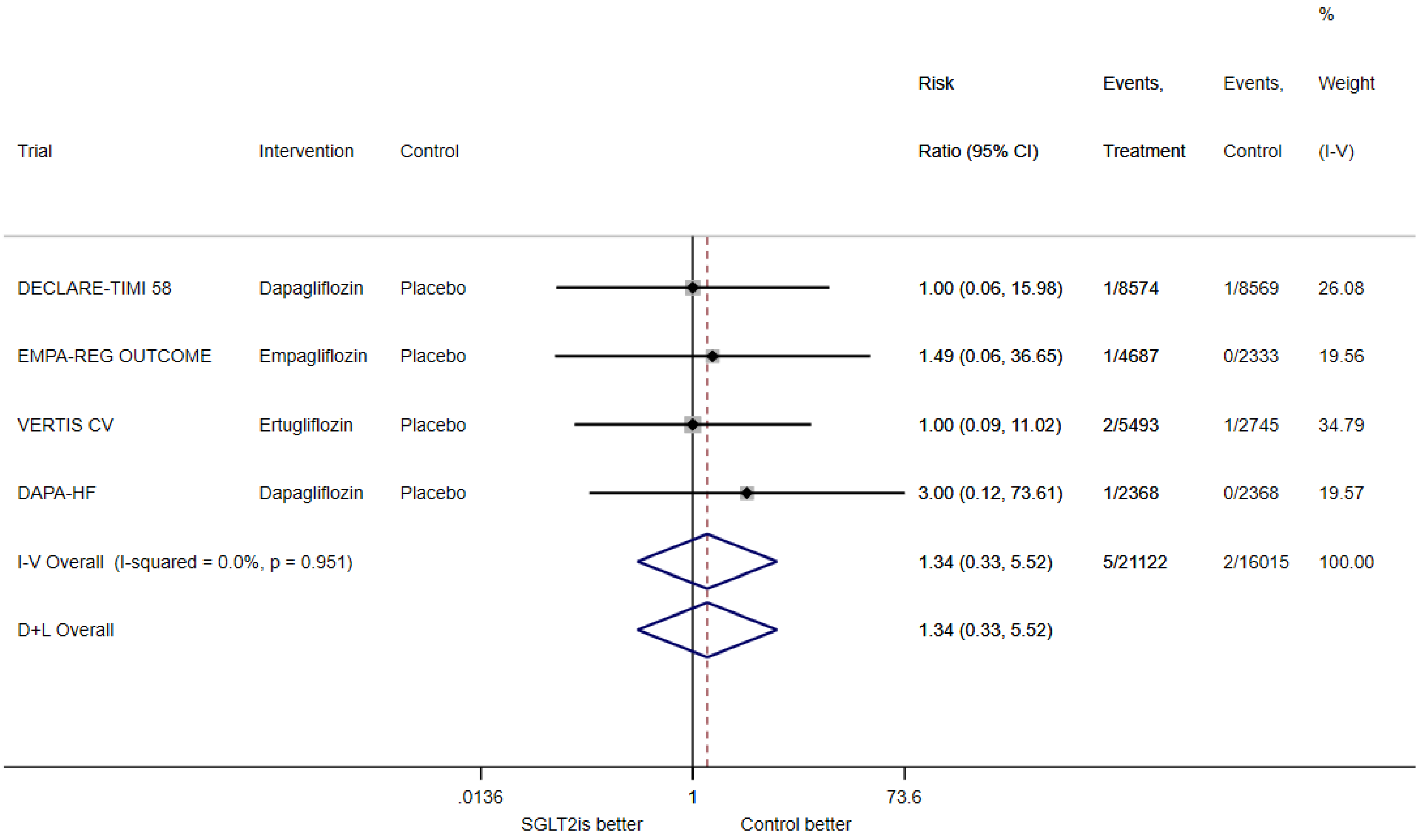

Figure S41 Meta-analysis of SGLT2is and Cardiovascular insufficiency

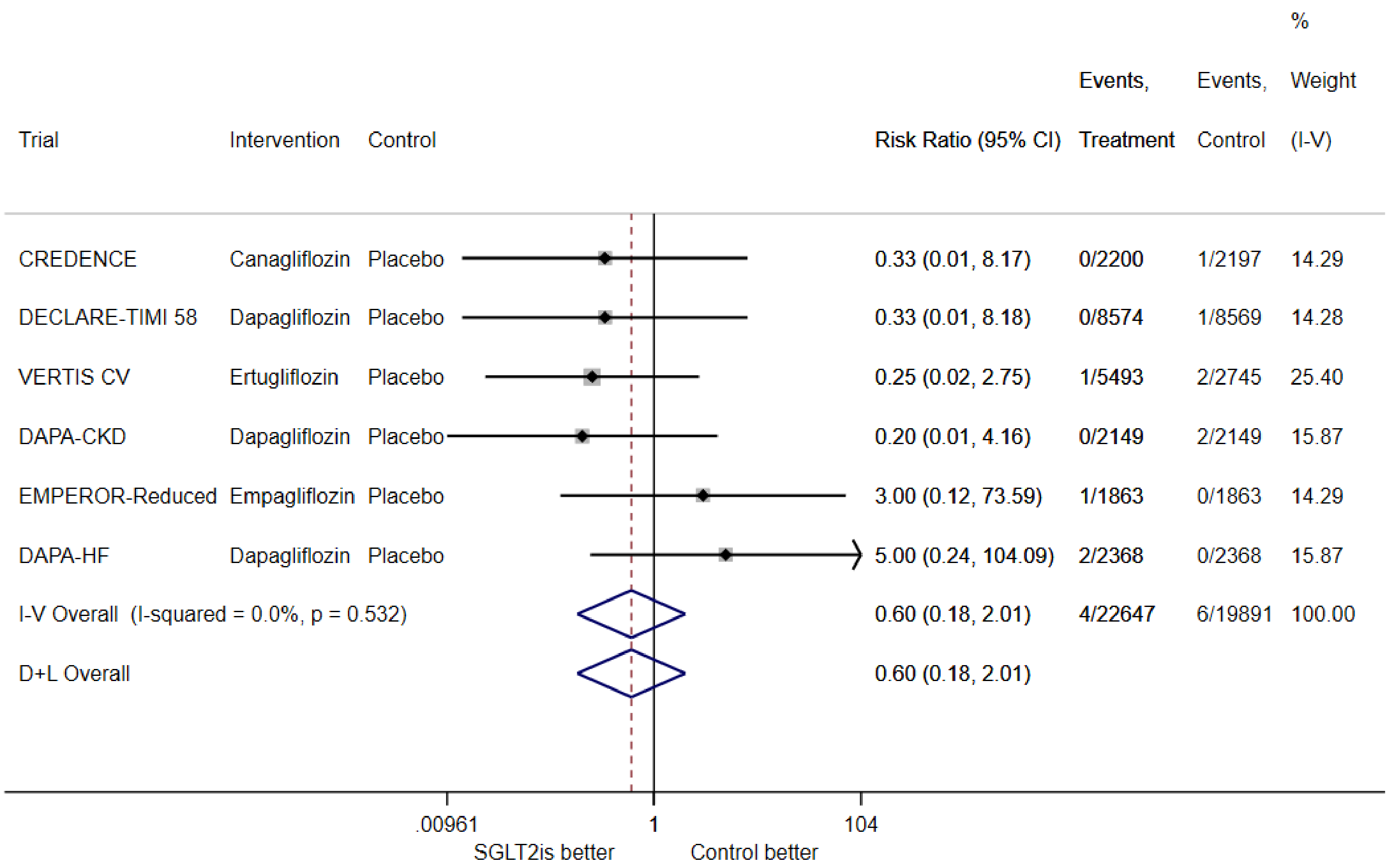

Figure S42 Meta-analysis of SGLT2is and Chronic left ventricular failure

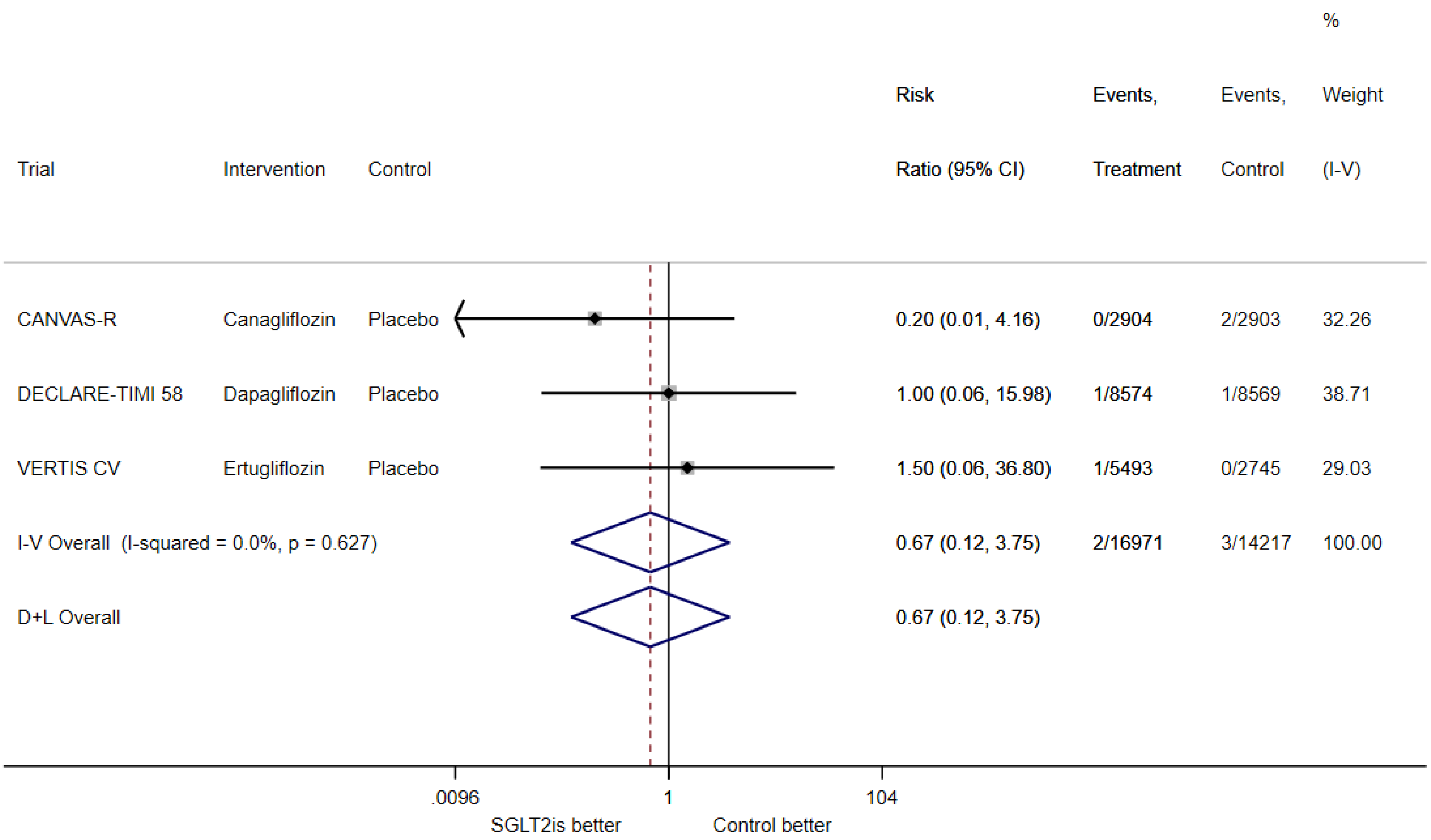

Figure S43 Meta-analysis of SGLT2is and Conduction disorder

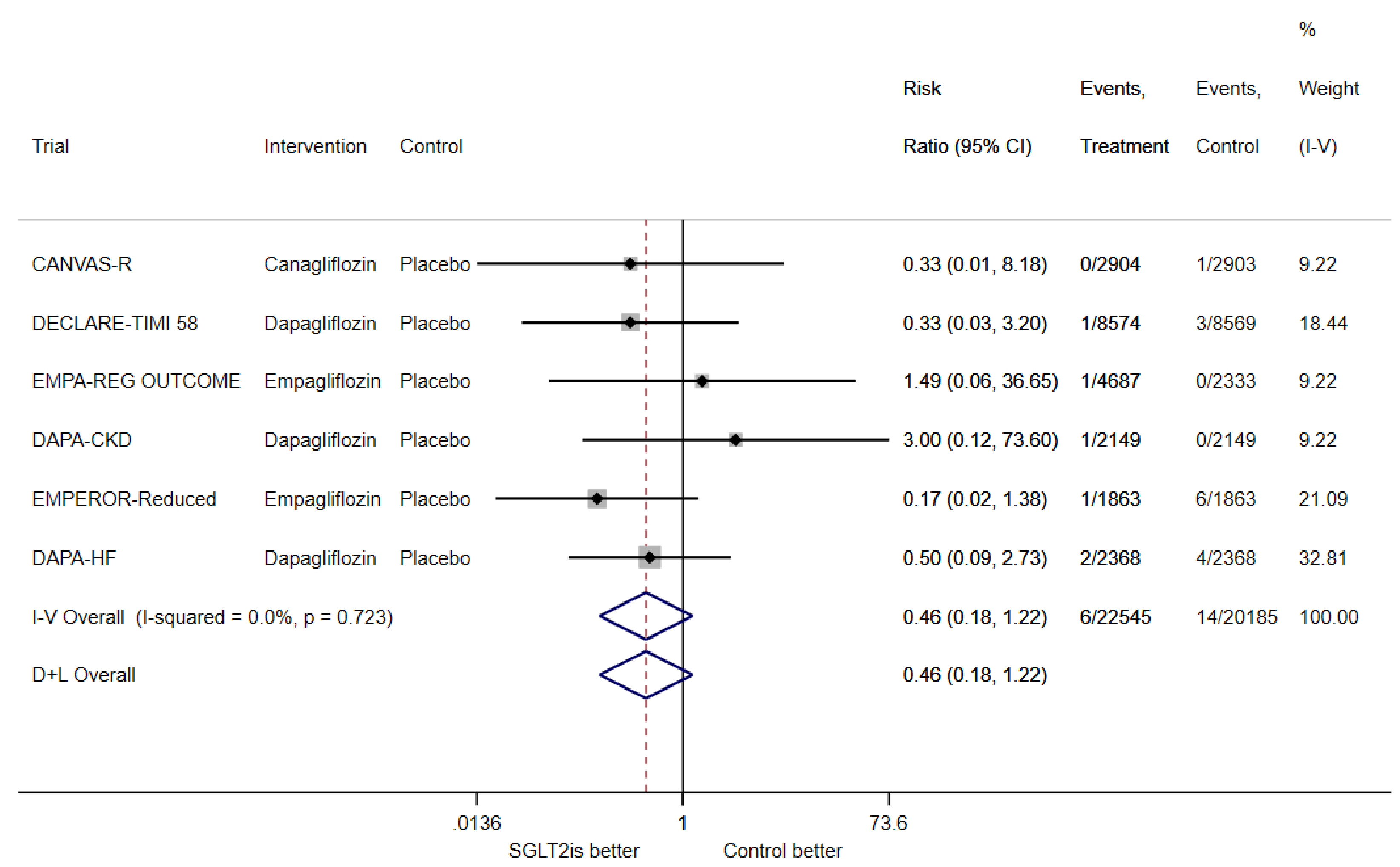

Figure S44 Meta-analysis of SGLT2is and Congestive cardiomyopathy

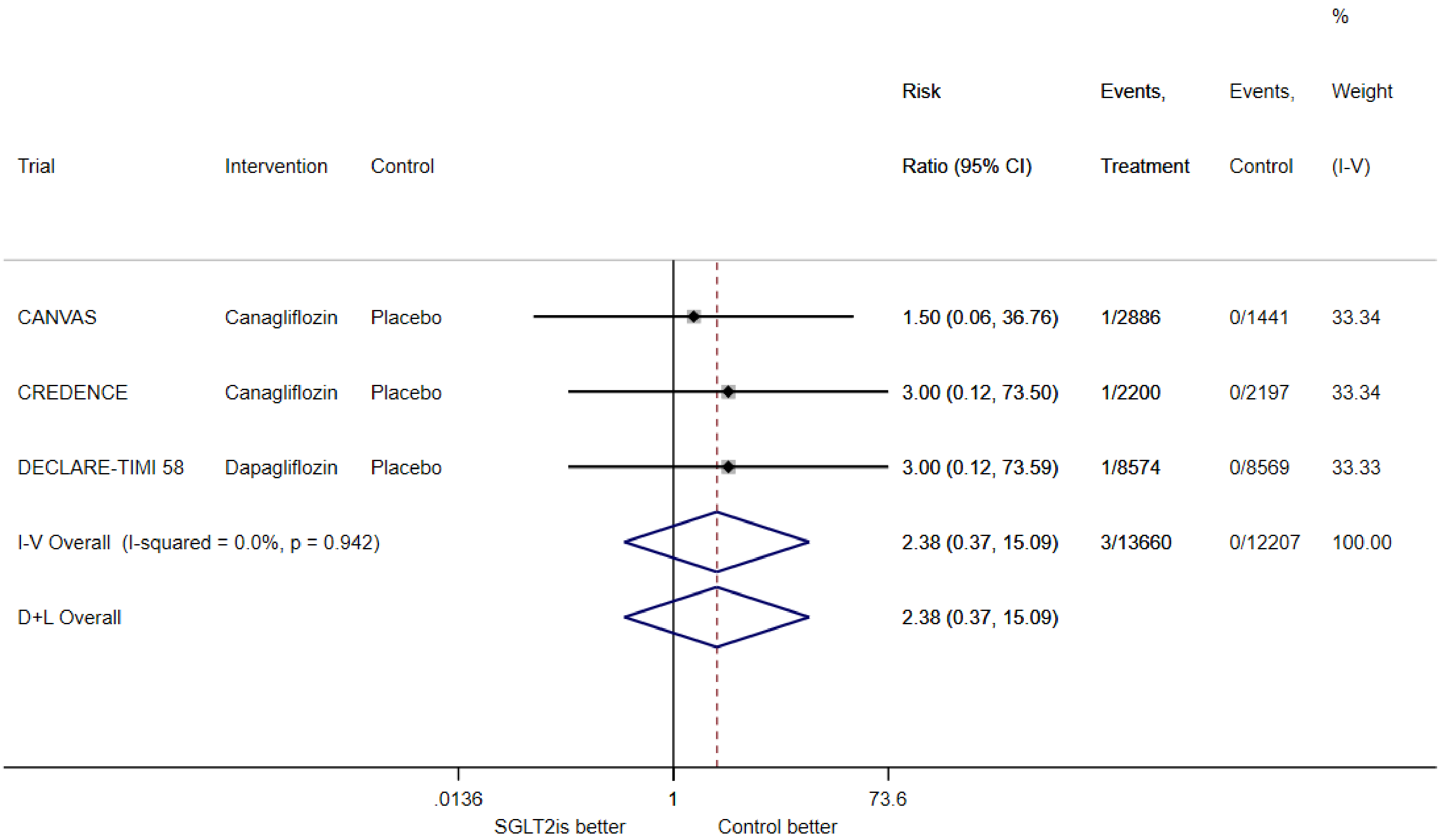

Figure S45 Meta-analysis of SGLT2is and Cor pulmonale

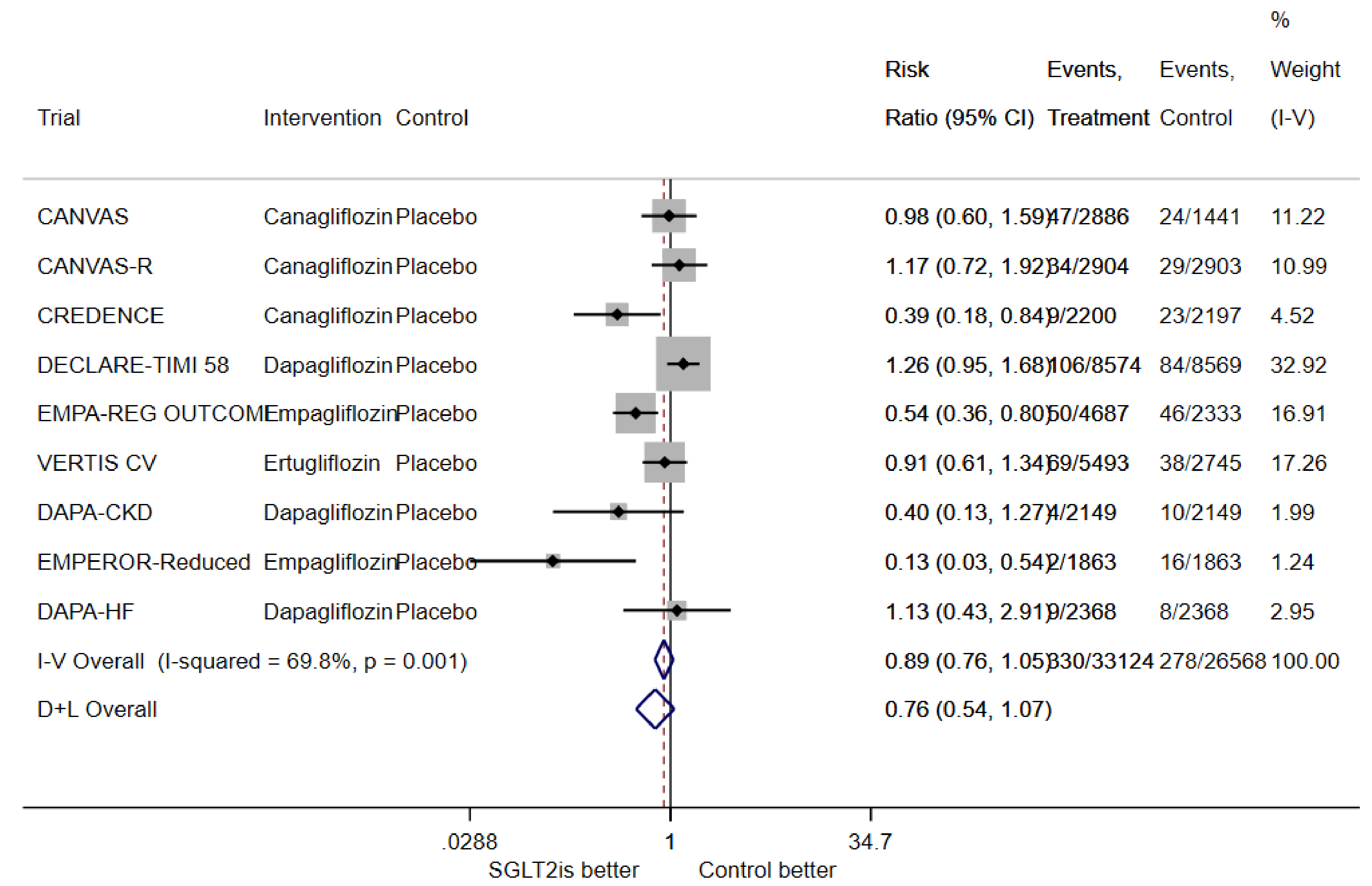

Figure S46 Meta-analysis of SGLT2is and Coronary artery disease

Trial	Intervention	Control	Risk Ratio (95% CI)	Events, Treatment	Events, Control	% Weight (I-V)
CANVAS	Canagliflozin	Placebo	0.17 (0.01, 4.08)	0/2886	1/1441	12.50
CANVAS-R	Canagliflozin	Placebo	3.00 (0.12, 73.59)	1/2904	0/2903	12.50
CREDENCE	Canagliflozin	Placebo	0.50 (0.05, 5.50)	1/2200	2/2197	22.23
EMPA-REG OUTCOME	Empagliflozin	Placebo	0.50 (0.03, 7.95)	1/4687	1/2333	16.67
VERTIS CV	Ertugliflozin	Placebo	1.00 (0.09, 11.02)	2/5493	1/2745	22.22
DAPA-HF	Dapagliflozin	Placebo	0.20 (0.01, 4.16)	0/2368	2/2368	13.89
I-V Overall (I-squared = 0.0%, p = 0.808)			0.56 (0.18, 1.73)	5/20538	7/13987	100.00
D+L Overall			0.56 (0.18, 1.73)			

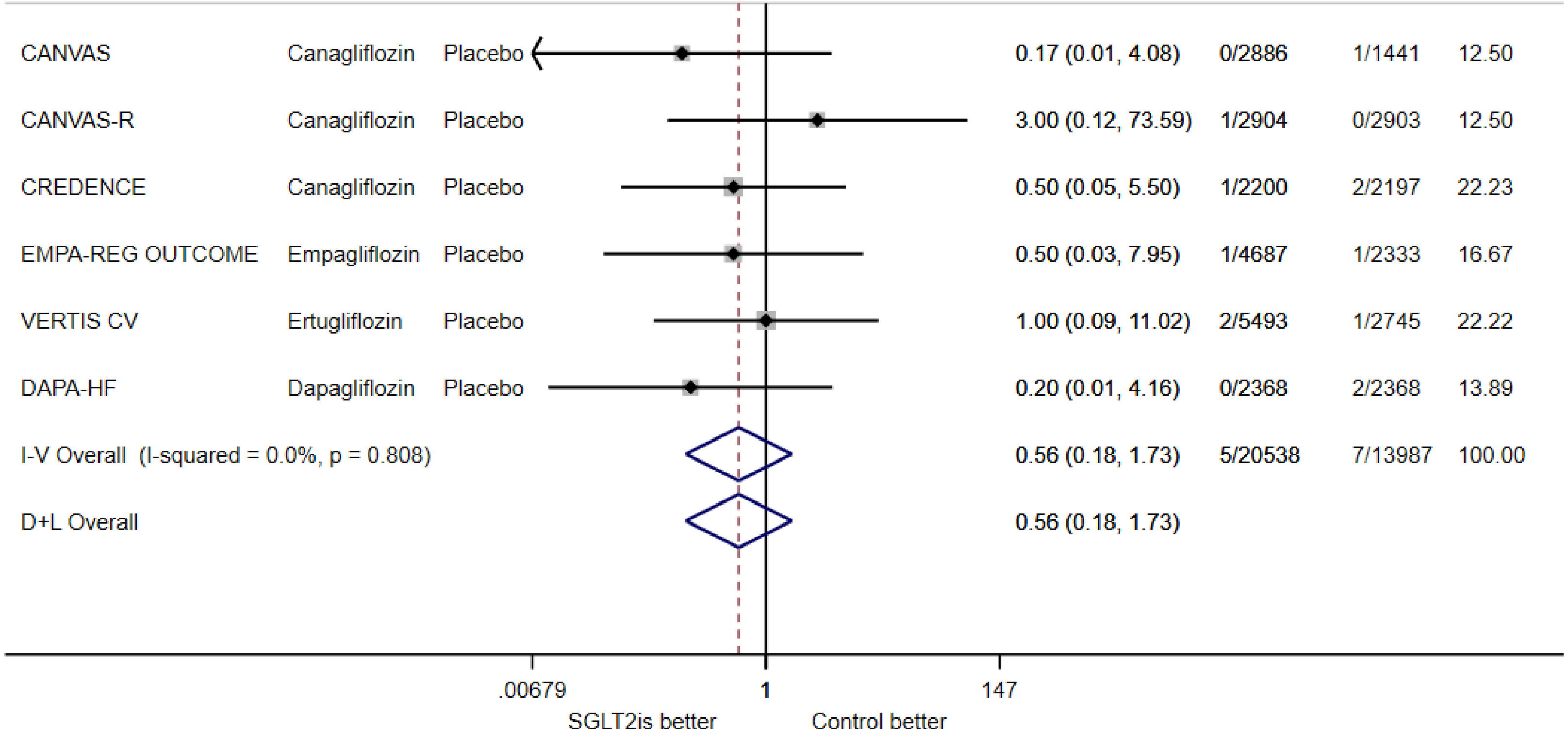

Figure S47 Meta-analysis of SGLT2is and Coronary artery insufficiency

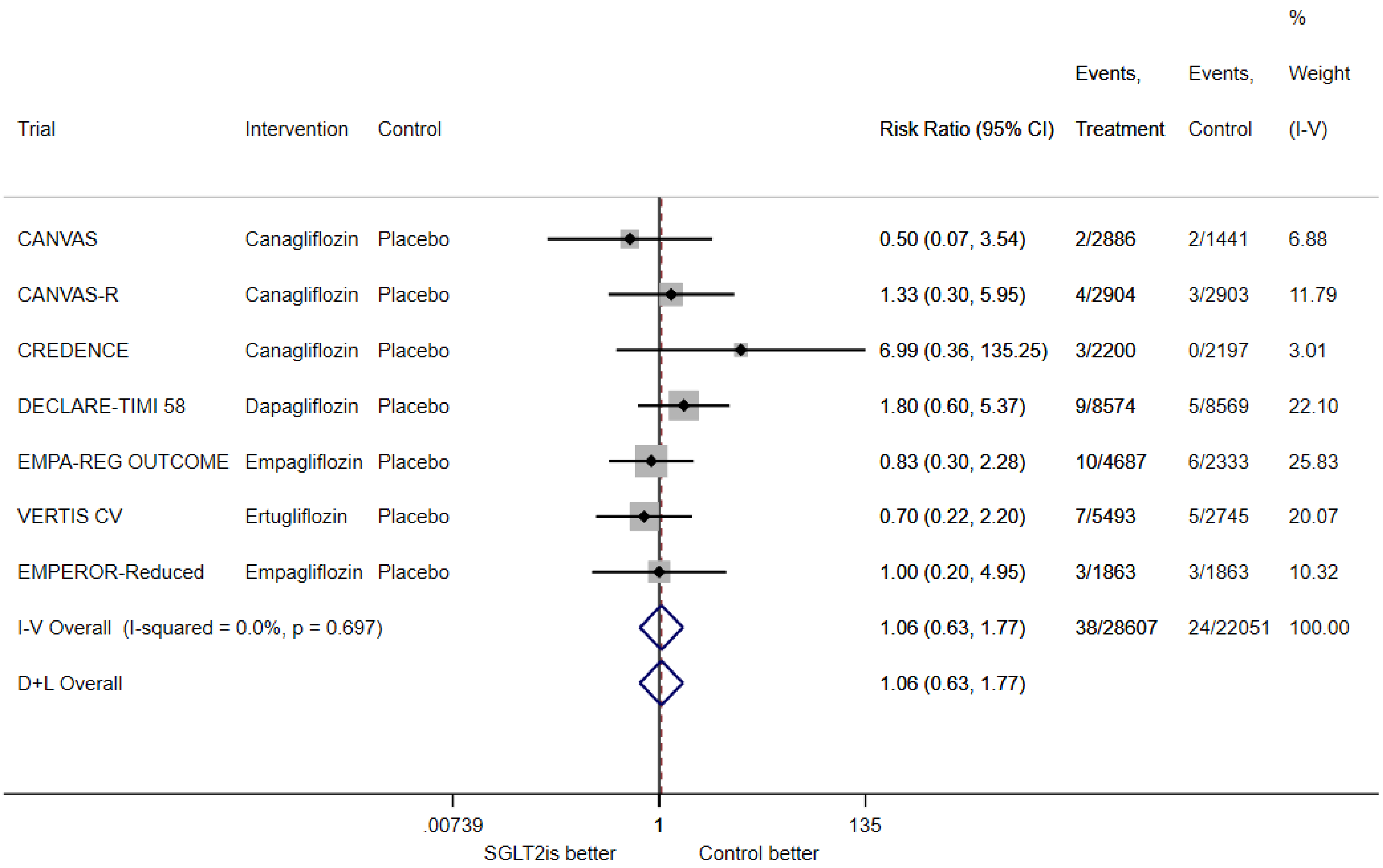

Figure S48 Meta-analysis of SGLT2is and Coronary artery occlusion

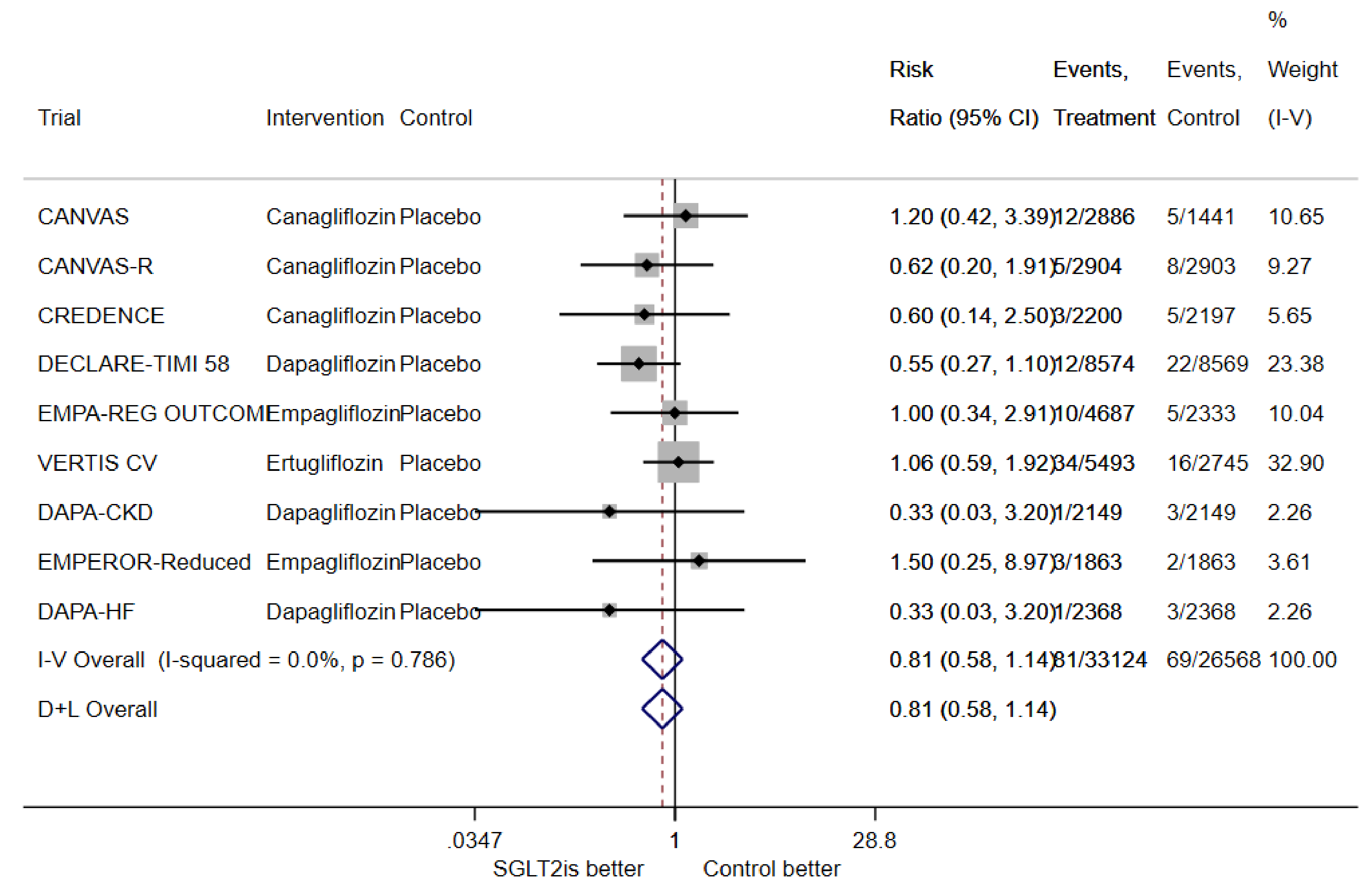

Figure S49 Meta-analysis of SGLT2is and Coronary artery stenosis

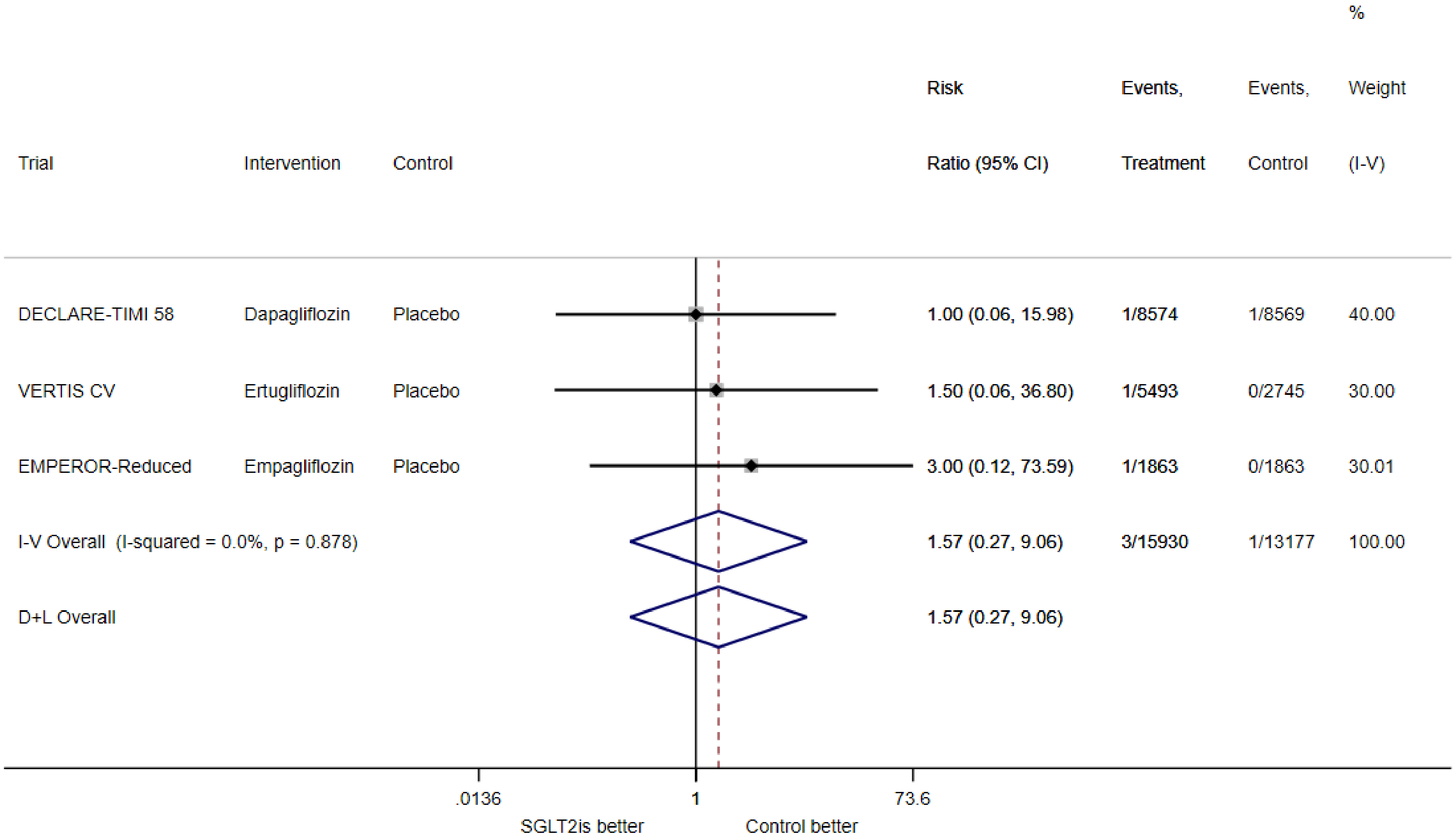

Figure S50 Meta-analysis of SGLT2is and Coronary ostial stenosis

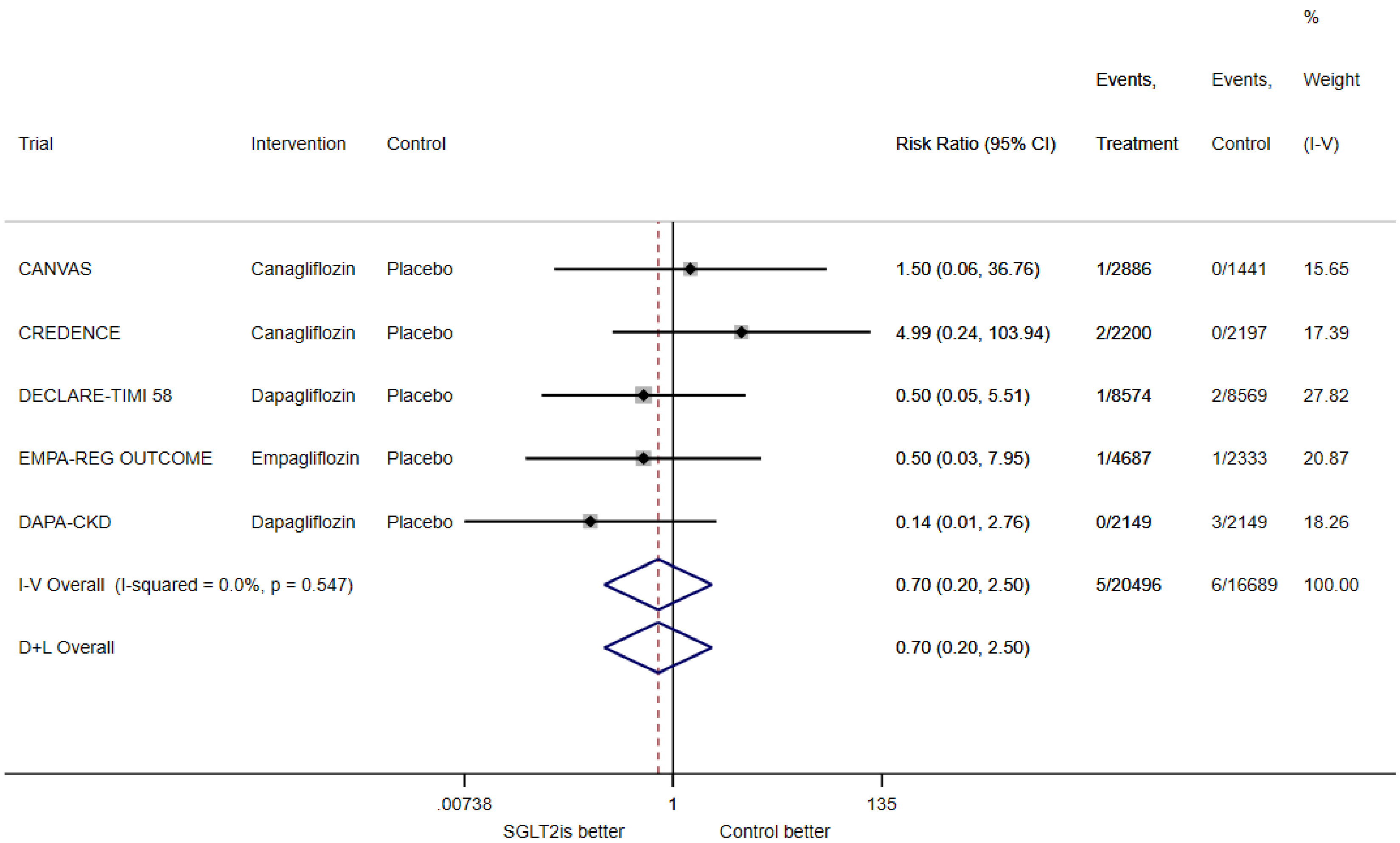

Figure S51 Meta-analysis of SGLT2is and Hypertensive heart disease

Trial	Intervention	Control	Risk Ratio (95% CI)	Events, Treatment	Events, Control	% Weight (I-V)
CANVAS	Canagliflozin	Placebo	0.50 (0.03, 7.98)	1/2886	1/1441	3.35
CANVAS-R	Canagliflozin	Placebo	0.25 (0.03, 2.23)	1/2904	4/2903	5.35
CREDENCE	Canagliflozin	Placebo	0.67 (0.19, 2.36)	4/2200	6/2197	16.09
DECLARE-TIMI 58	Dapagliflozin	Placebo	0.56 (0.26, 1.20)	10/8574	18/8569	43.07
EMPA-REG OUTCOME	Empagliflozin	Placebo	1.99 (0.22, 17.80)	4/4687	1/2333	5.35
VERTIS CV	Ertugliflozin	Placebo	2.50 (0.29, 21.38)	5/5493	1/2745	5.58
DAPA-CKD	Dapagliflozin	Placebo	1.00 (0.25, 3.99)	4/2149	4/2149	13.40
EMPEROR-Reduced	Empagliflozin	Placebo	1.00 (0.06, 15.98)	1/1863	1/1863	3.35
DAPA-HF	Dapagliflozin	Placebo	2.00 (0.18, 22.04)	2/2368	1/2368	4.46
I-V Overall (I-squared = 0.0%, p = 0.811)			0.74 (0.45, 1.23)	32/33124	37/26568	100.00
D+L Overall			0.74 (0.45, 1.23)			

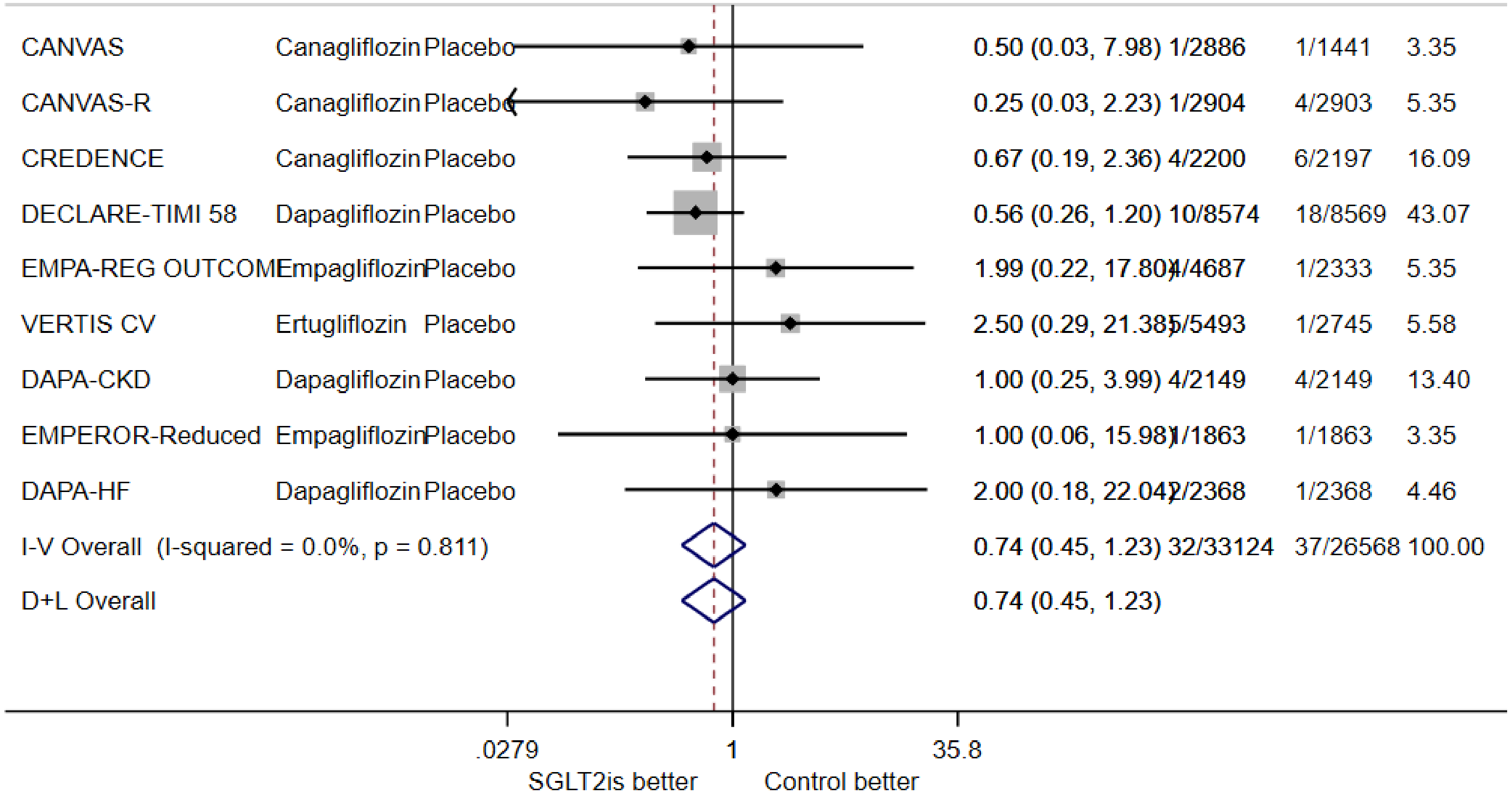

Figure S52 Meta-analysis of SGLT2is and Ischaemic cardiomyopathy

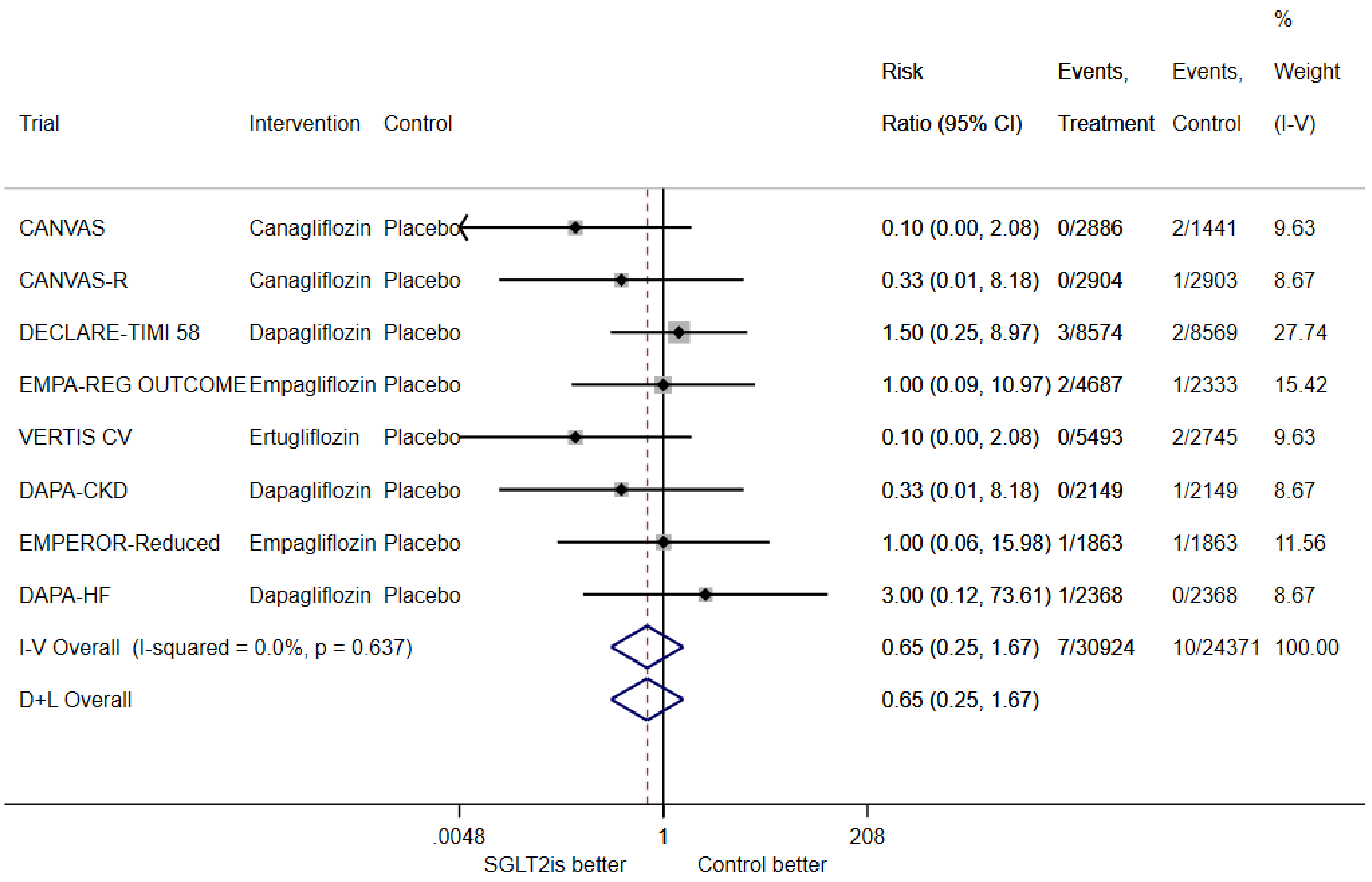

Figure S53 Meta-analysis of SGLT2is and Left ventricular dysfunction

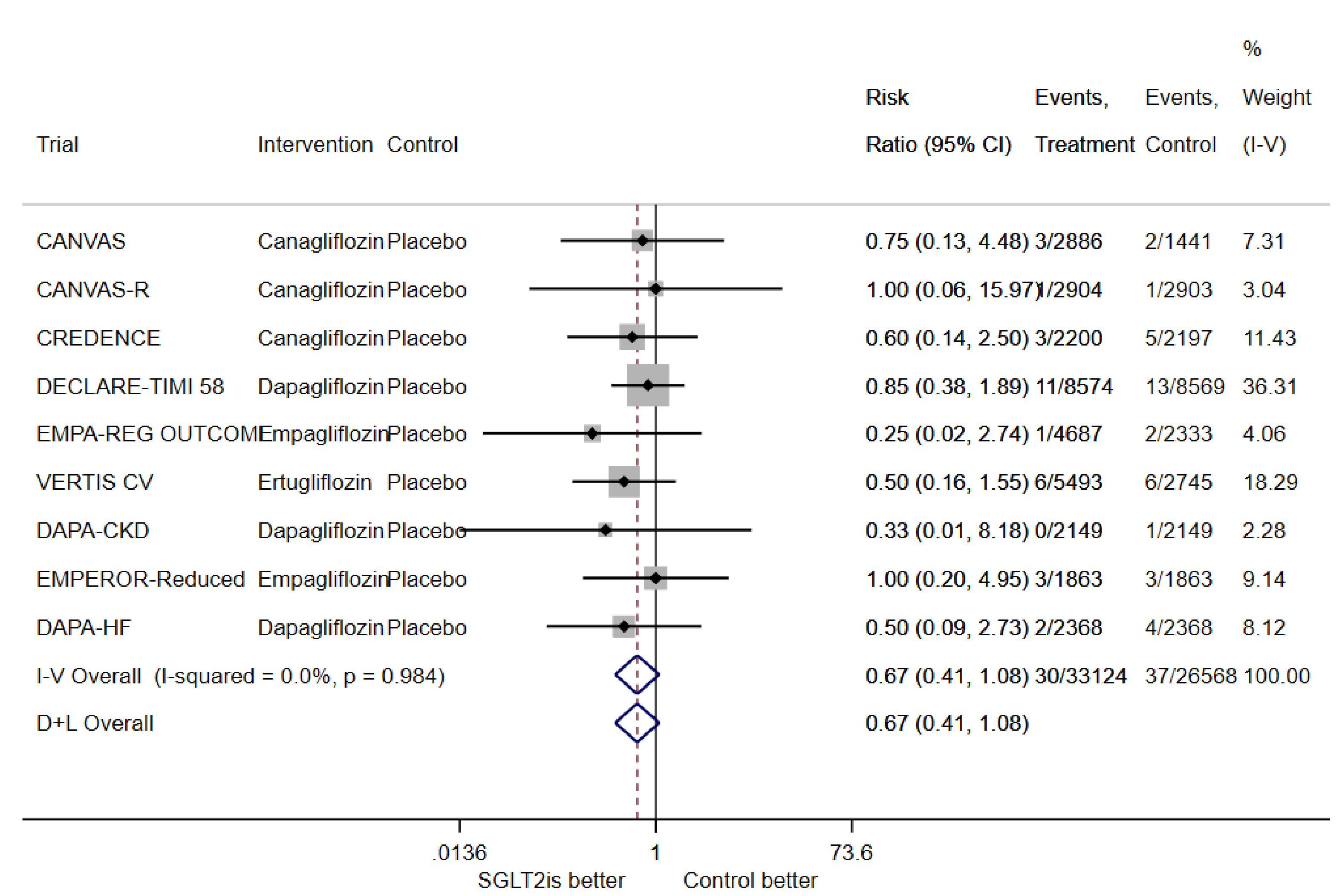

Figure S54 Meta-analysis of SGLT2is and Left ventricular failure

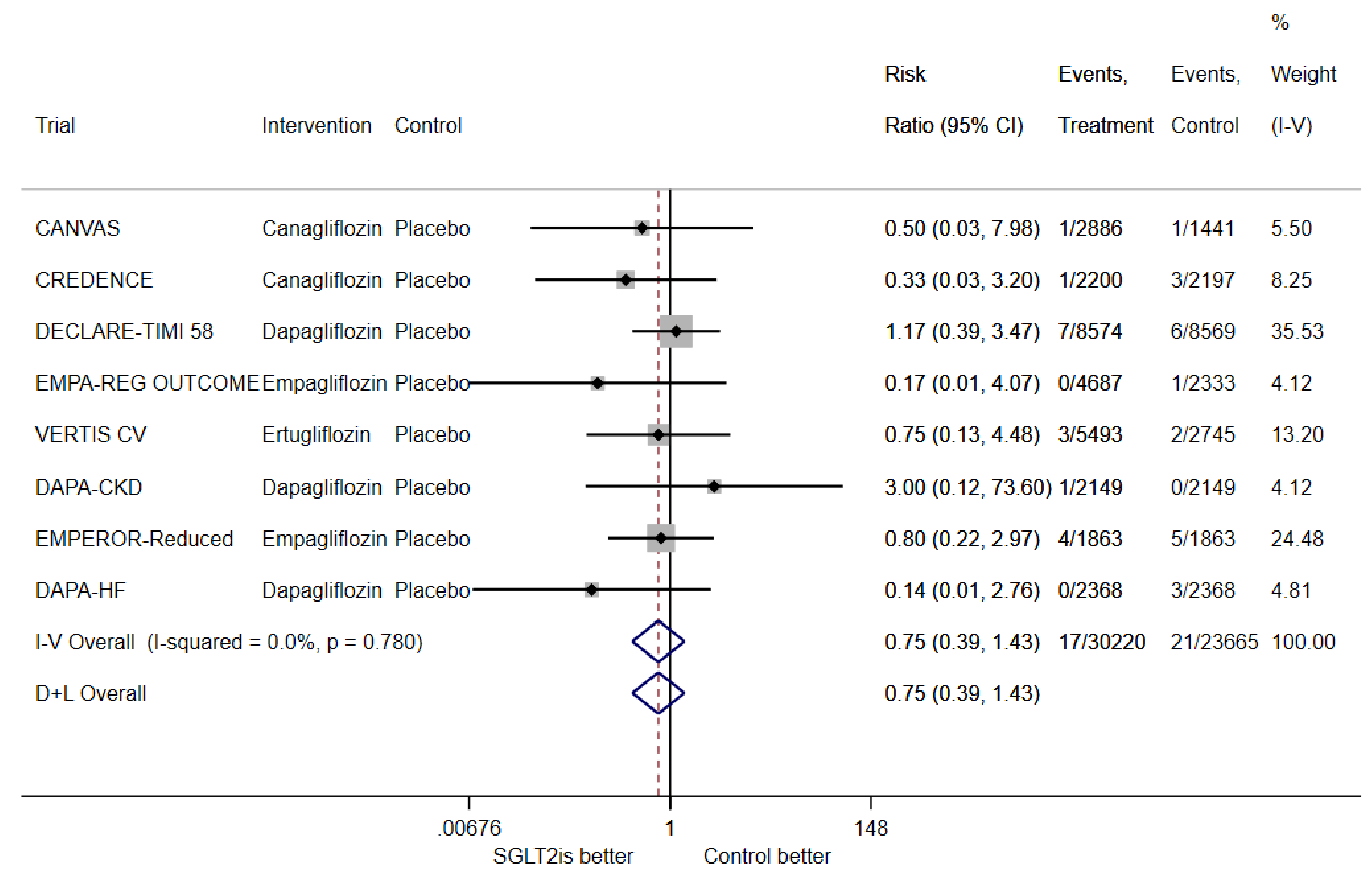

Figure S55 Meta-analysis of SGLT2is and Mitral valve incompetence

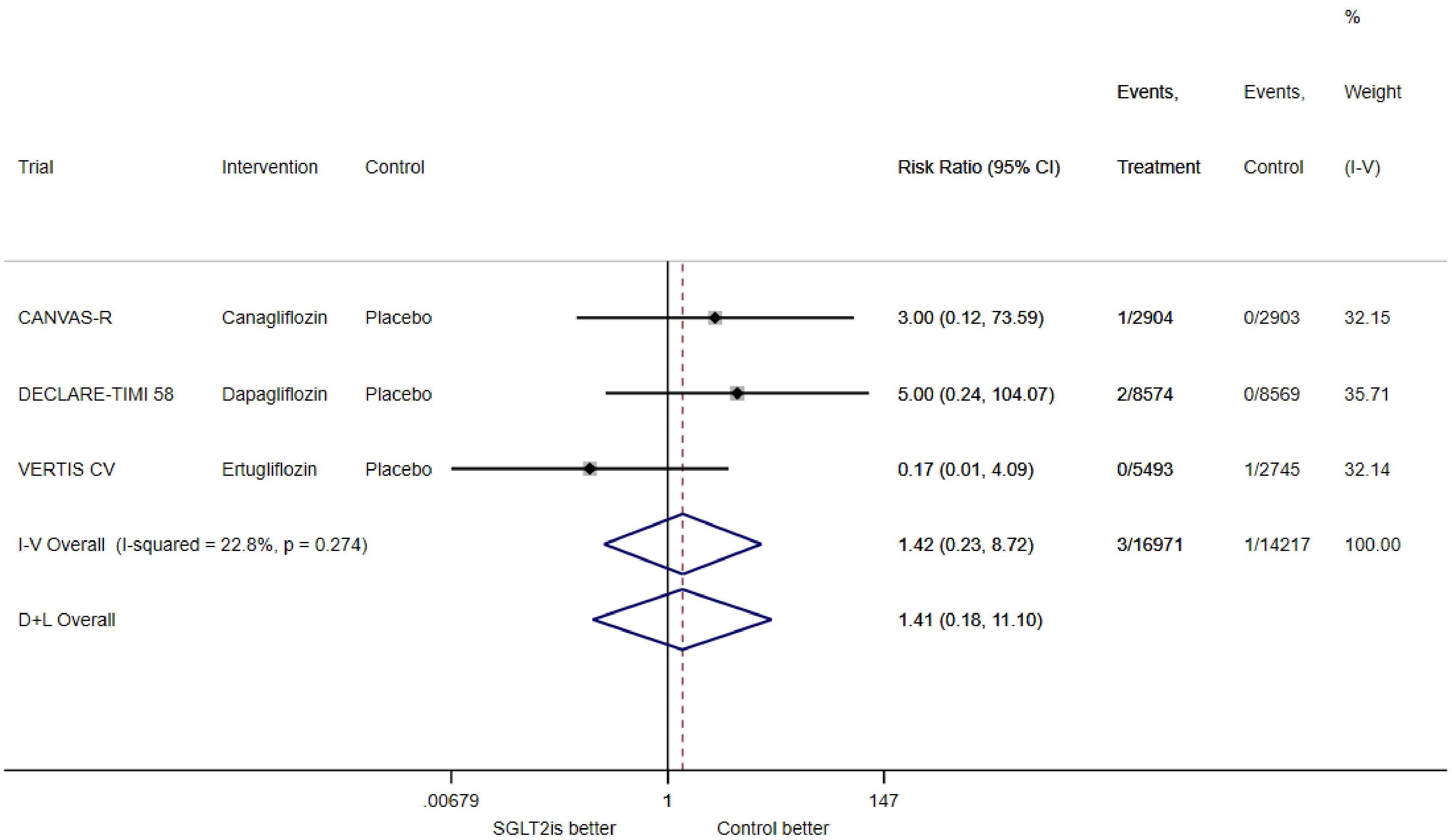

Figure S56 Meta-analysis of SGLT2is and Mitral valve stenosis

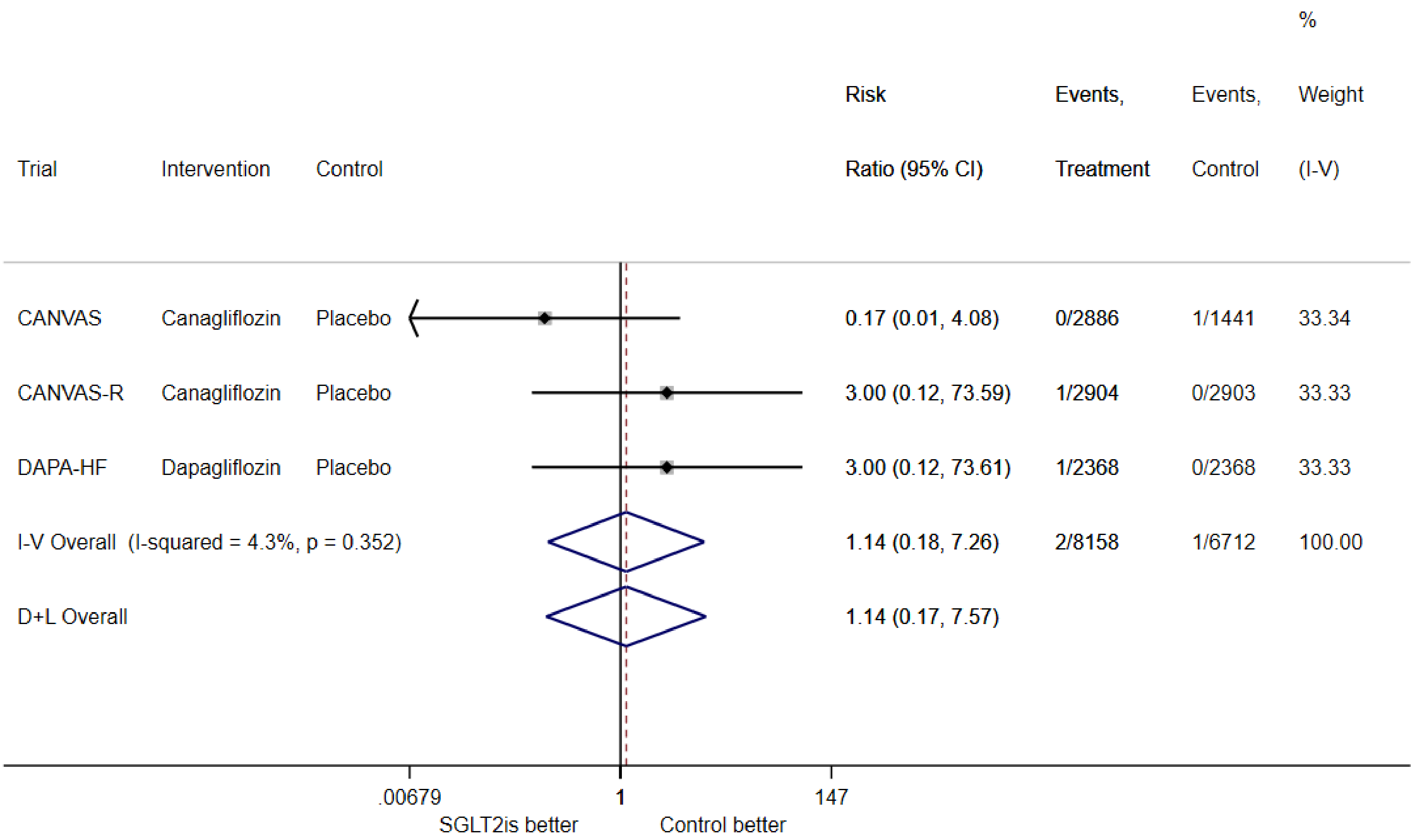

Figure S57 Meta-analysis of SGLT2is and Myocardial fibrosis

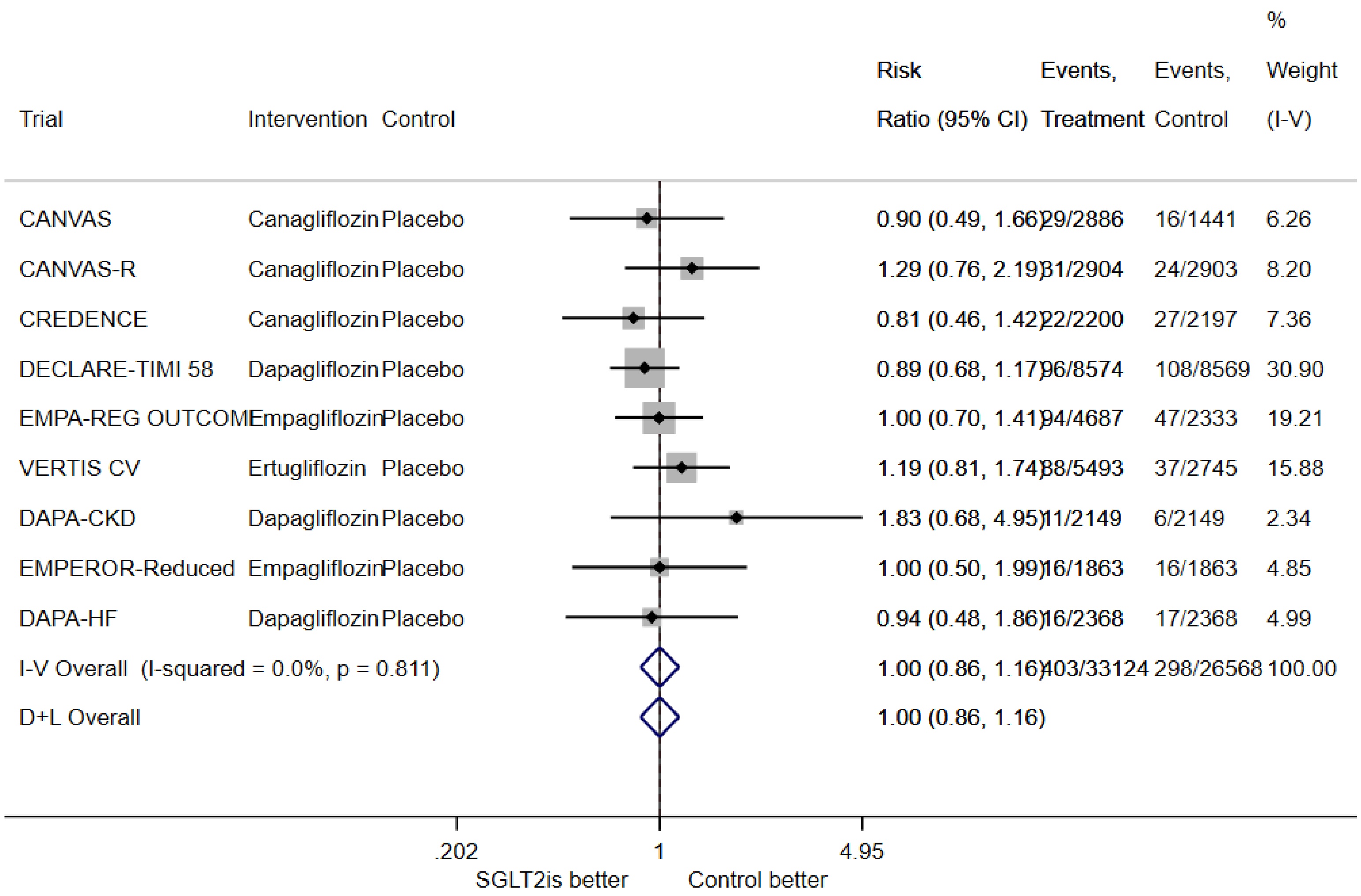

Figure S58 Meta-analysis of SGLT2is and Myocardial infarction

Trial	Intervention	Control	Risk Ratio (95% CI)	Events, Treatment	Events, Control	% Weight (I-V)
CANVAS	Canagliflozin	Placebo	0.94 (0.42, 2.11)	17/2886	9/1441	9.45
CANVAS-R	Canagliflozin	Placebo	0.78 (0.29, 2.08)	7/2904	9/2903	6.30
CREDENCE	Canagliflozin	Placebo	1.00 (0.38, 2.66)	8/2200	8/2197	6.41
DECLARE-TIMI 58	Dapagliflozin	Placebo	0.87 (0.59, 1.28)	47/8574	54/8569	40.35
EMPA-REG OUTCOME	Empagliflozin	Placebo	0.40 (0.19, 0.85)	12/4687	15/2333	10.69
VERTIS CV	Ertugliflozin	Placebo	0.83 (0.47, 1.49)	30/5493	18/2745	18.07
DAPA-CKD	Dapagliflozin	Placebo	1.00 (0.32, 3.10)	6/2149	6/2149	4.80
EMPEROR-Reduced	Empagliflozin	Placebo	3.00 (0.31, 28.81)	3/1863	1/1863	1.20
DAPA-HF	Dapagliflozin	Placebo	0.75 (0.17, 3.35)	3/2368	4/2368	2.74
I-V Overall (I-squared = 0.0%, p = 0.729)			0.82 (0.64, 1.04)	133/33124	124/26568	100.00
D+L Overall			0.82 (0.64, 1.04)			

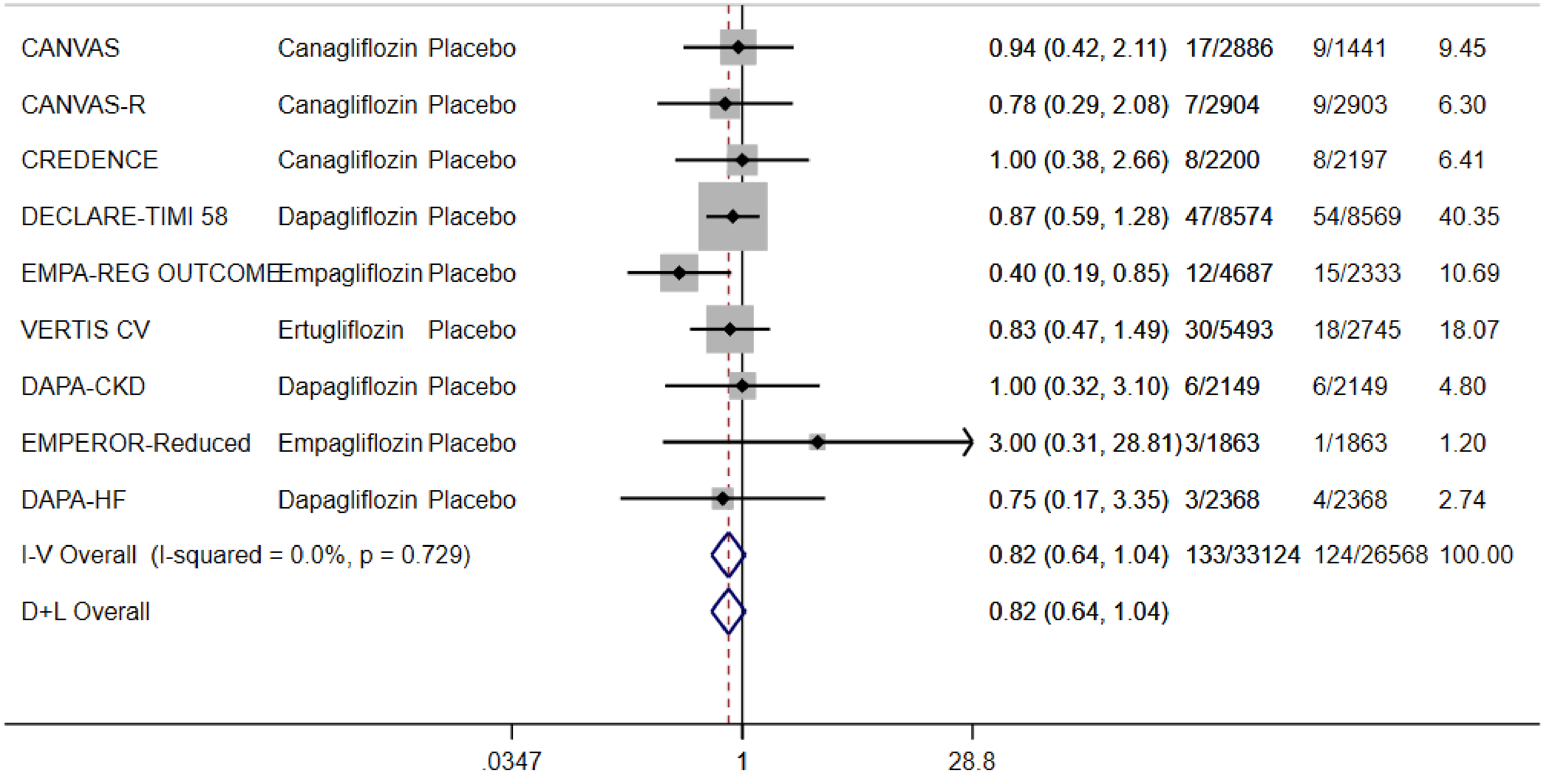

Figure S59 Meta-analysis of SGLT2is and Myocardial ischaemia

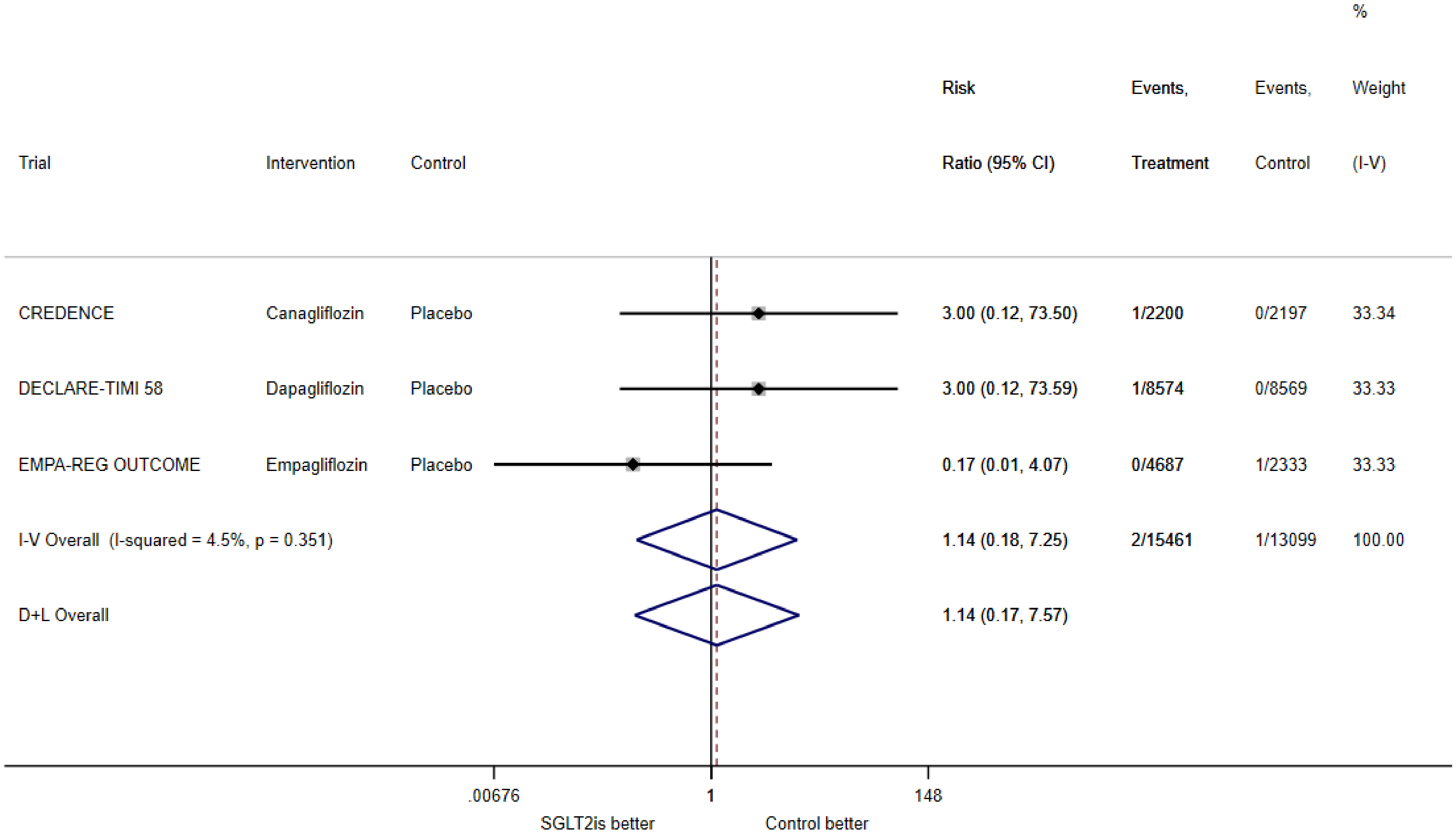

Figure S60 Meta-analysis of SGLT2is and Myocarditis

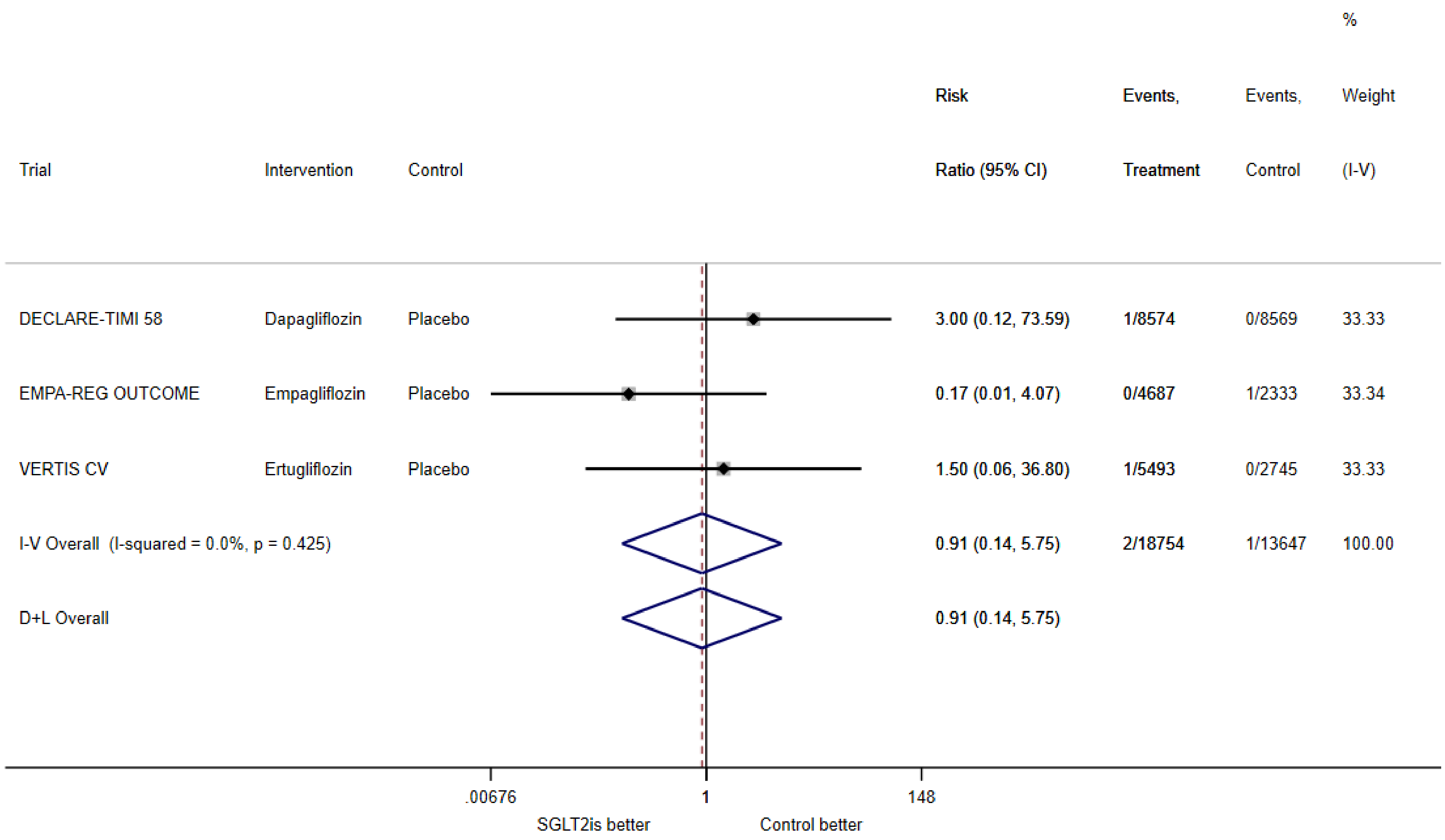

Figure S61 Meta-analysis of SGLT2is and Nodal arrhythmia

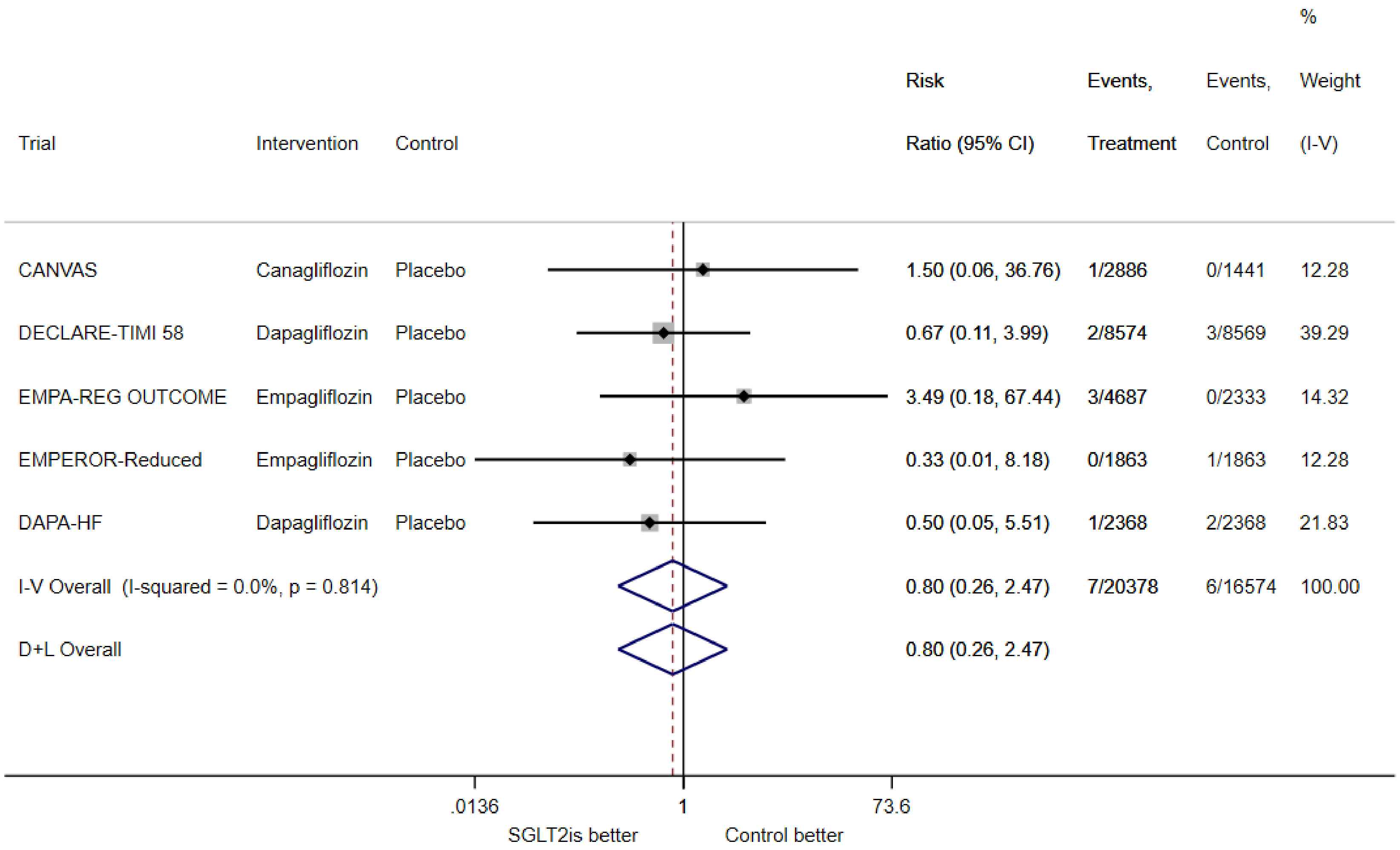

Figure S62 Meta-analysis of SGLT2is and Palpitations

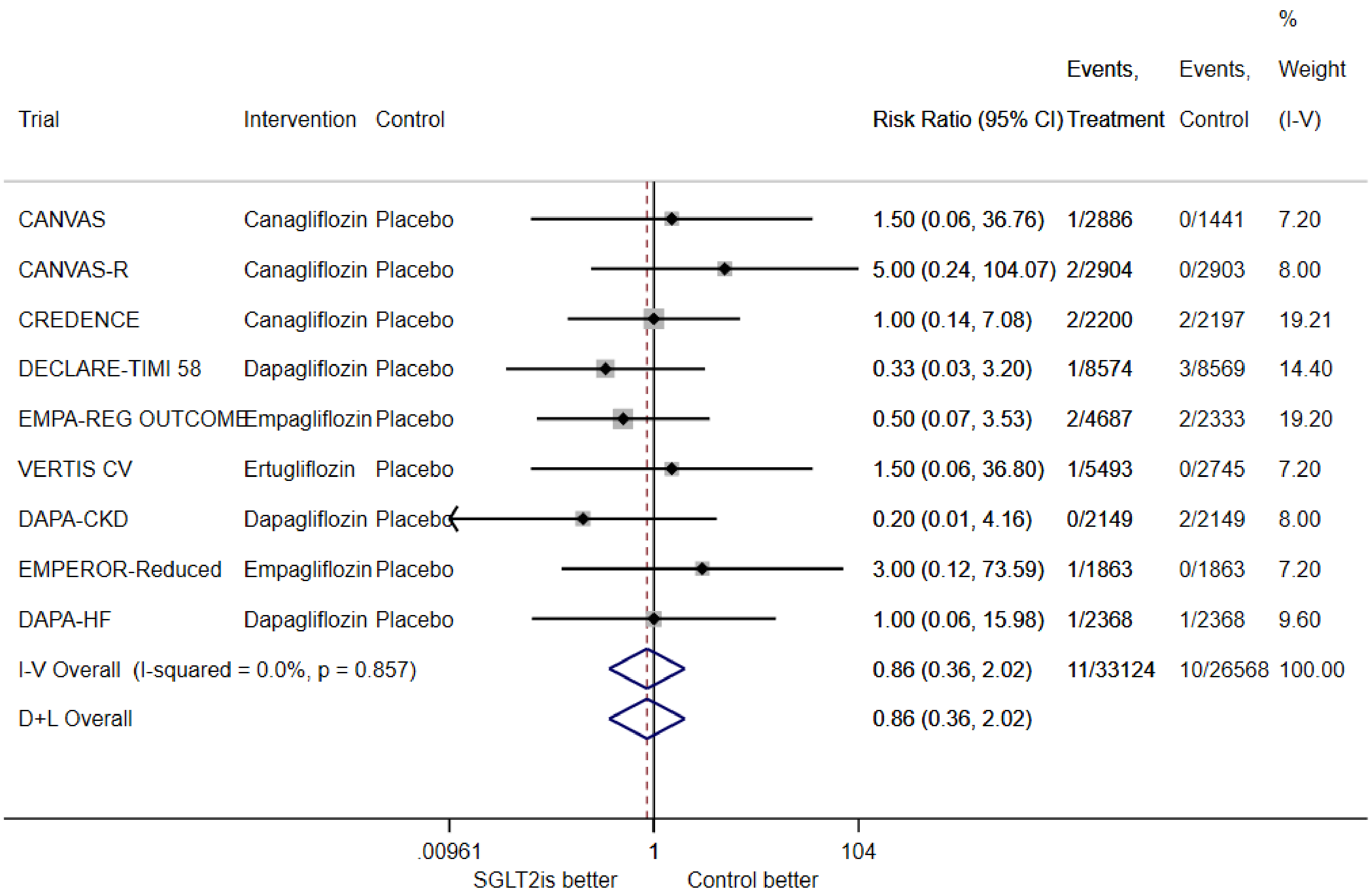

Figure S63 Meta-analysis of SGLT2is and Pericardial effusion

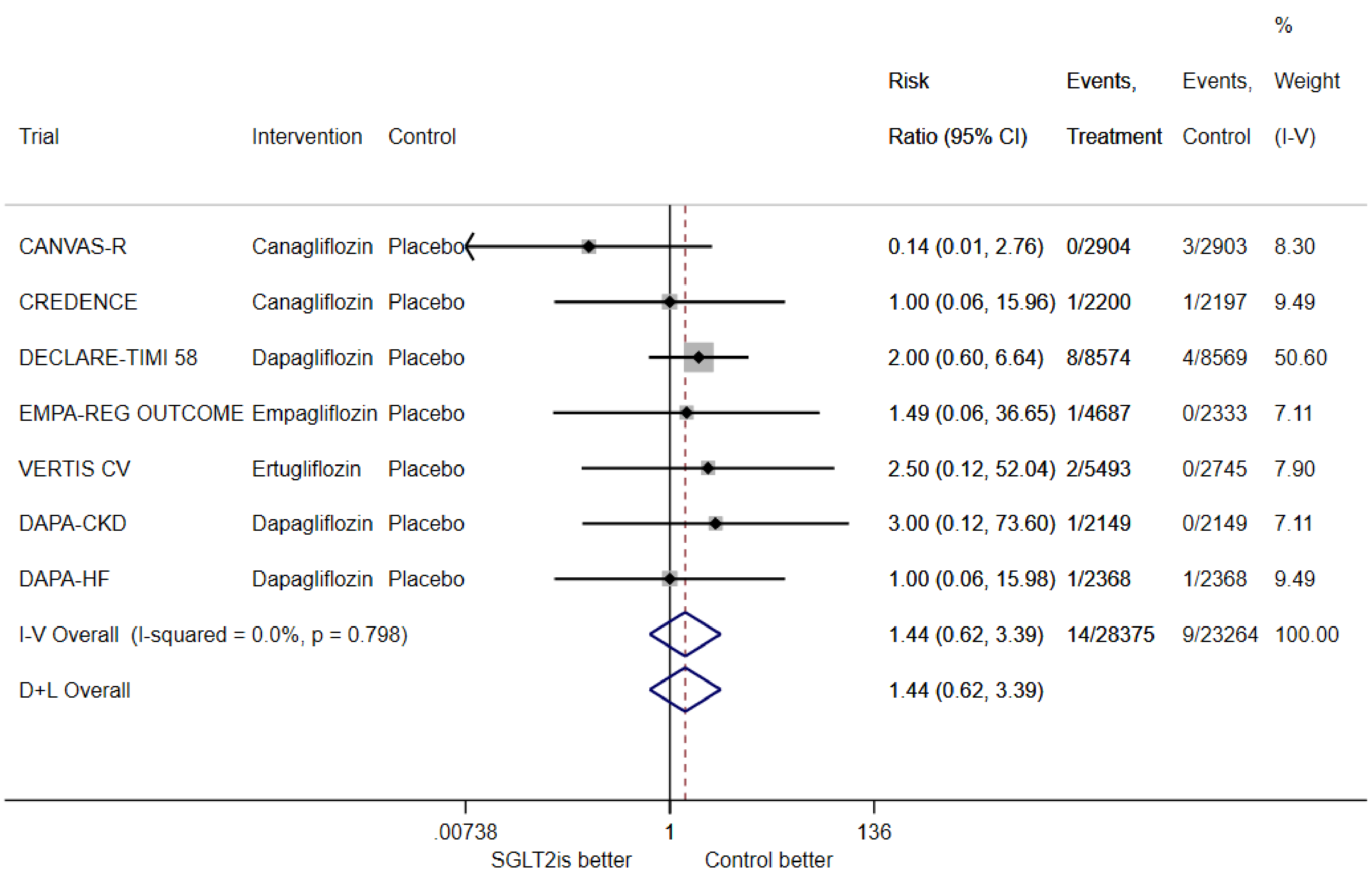

Figure S64 Meta-analysis of SGLT2is and Pericarditis

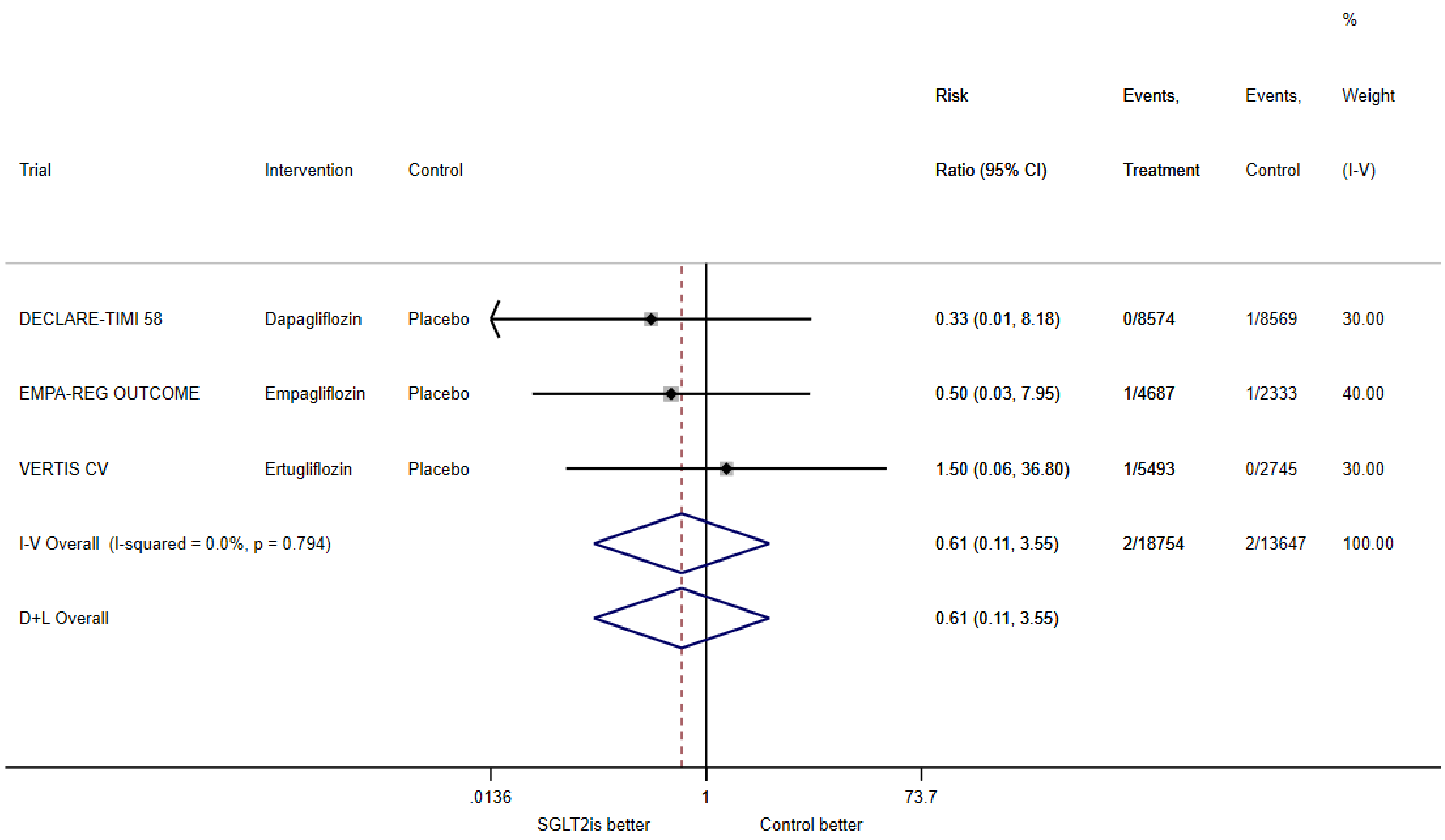

Figure S65 Meta-analysis of SGLT2is and Postinfarction angina

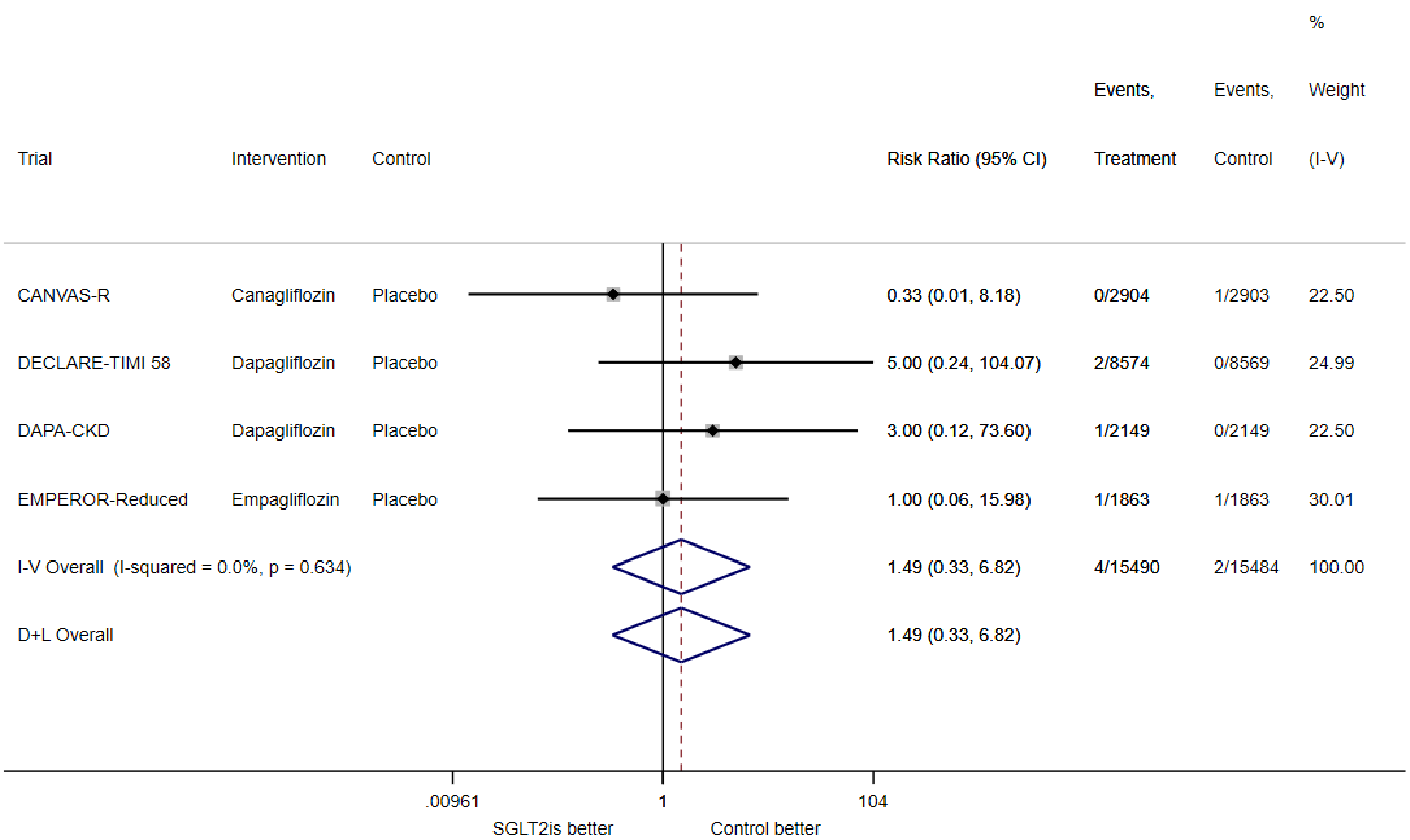

Figure S66 Meta-analysis of SGLT2is and Prinzmetal angina

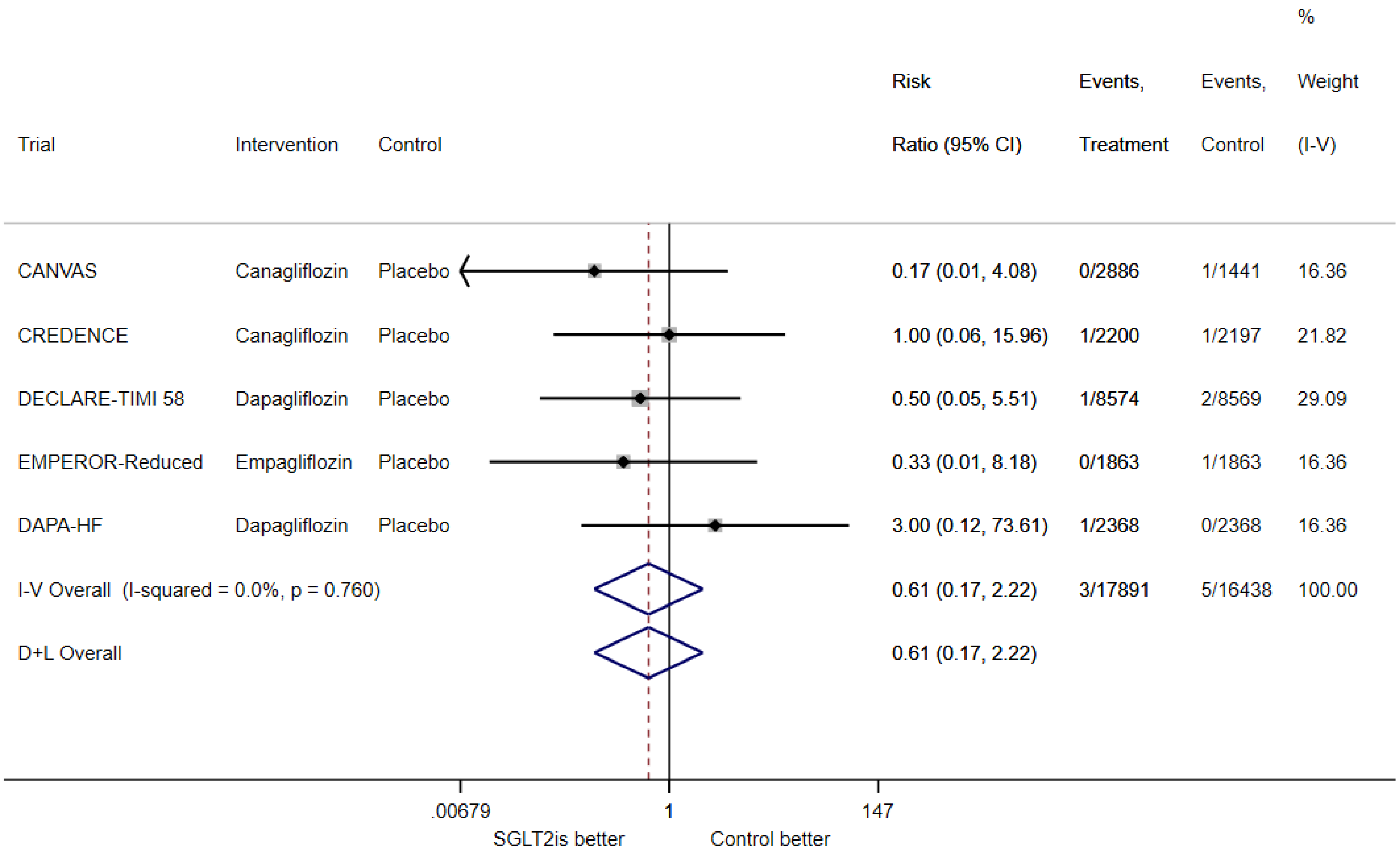

Figure S67 Meta-analysis of SGLT2is and Pulseless electrical activity

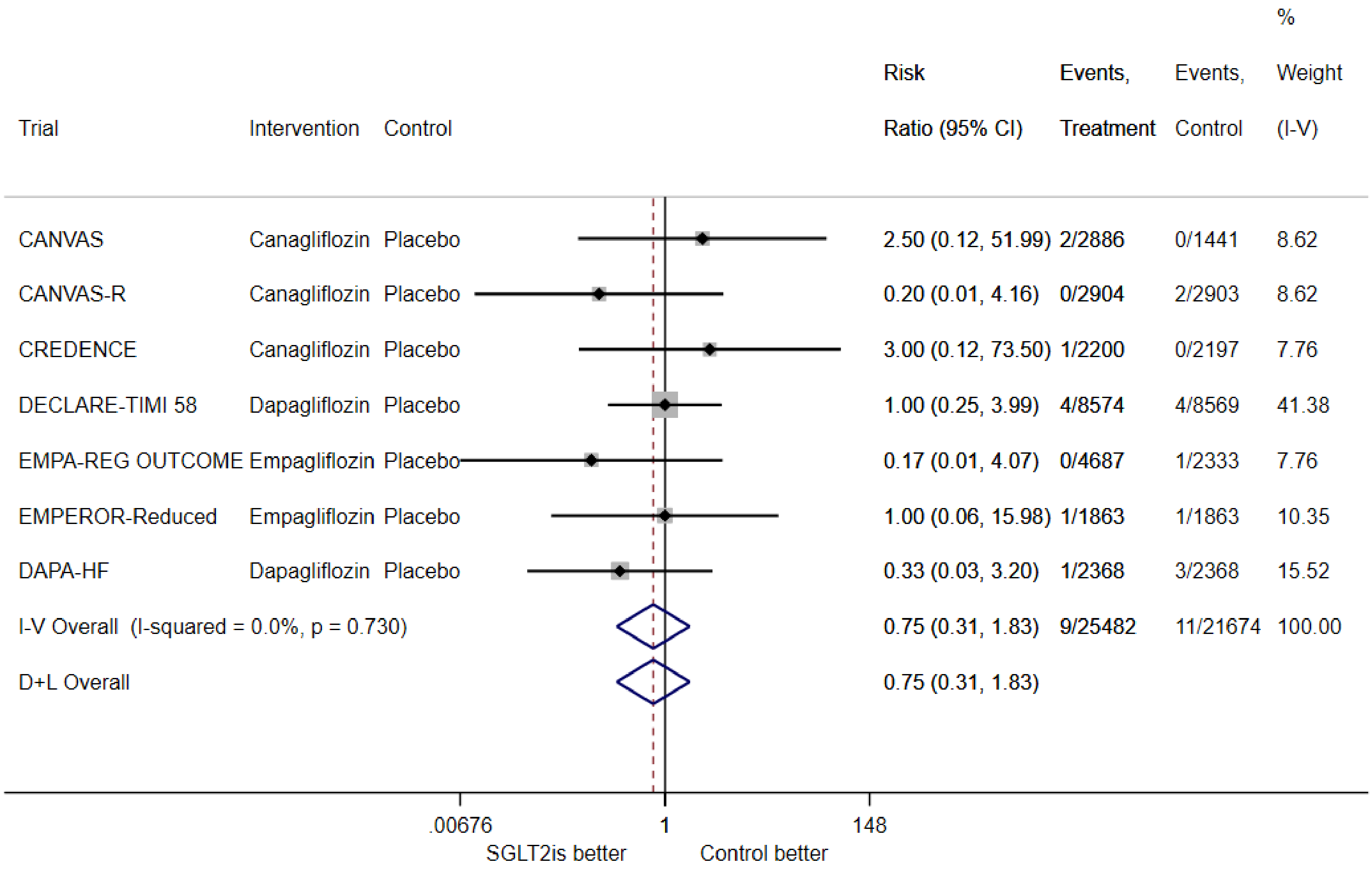

Figure S68 Meta-analysis of SGLT2is and Right ventricular failure

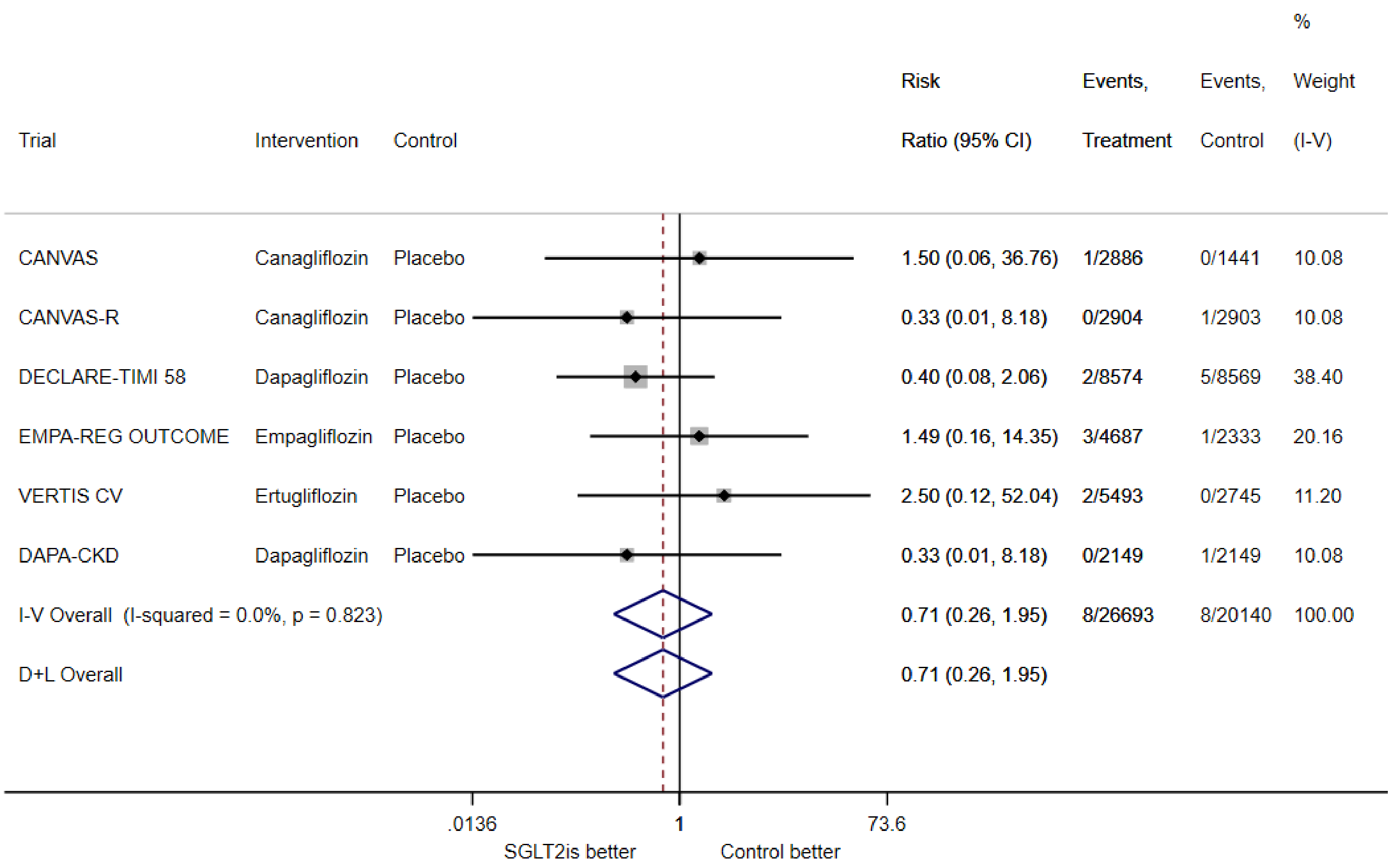

Figure S69 Meta-analysis of SGLT2is and Sinus bradycardia

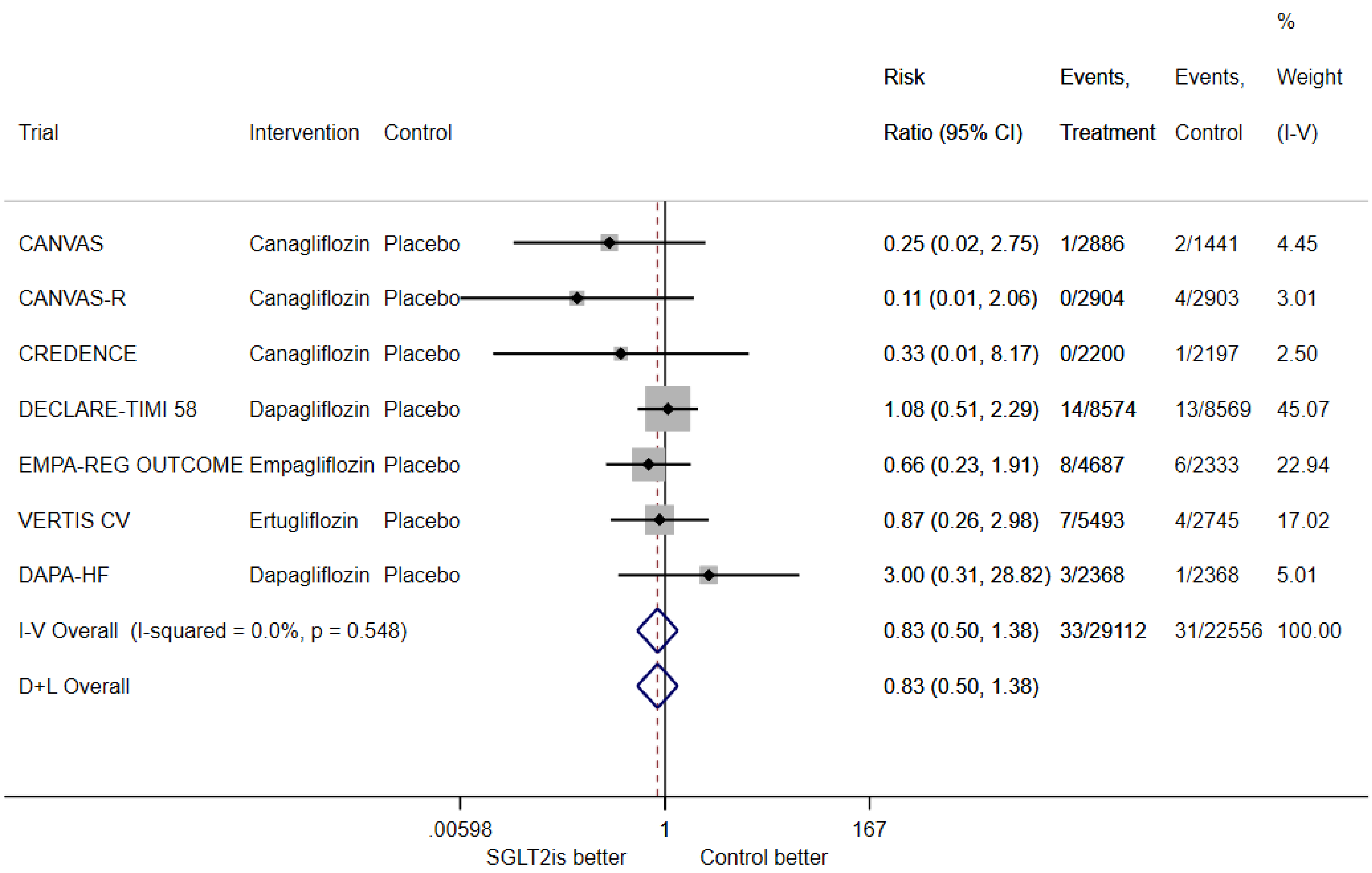

Figure S70 Meta-analysis of SGLT2is and Sinus node dysfunction

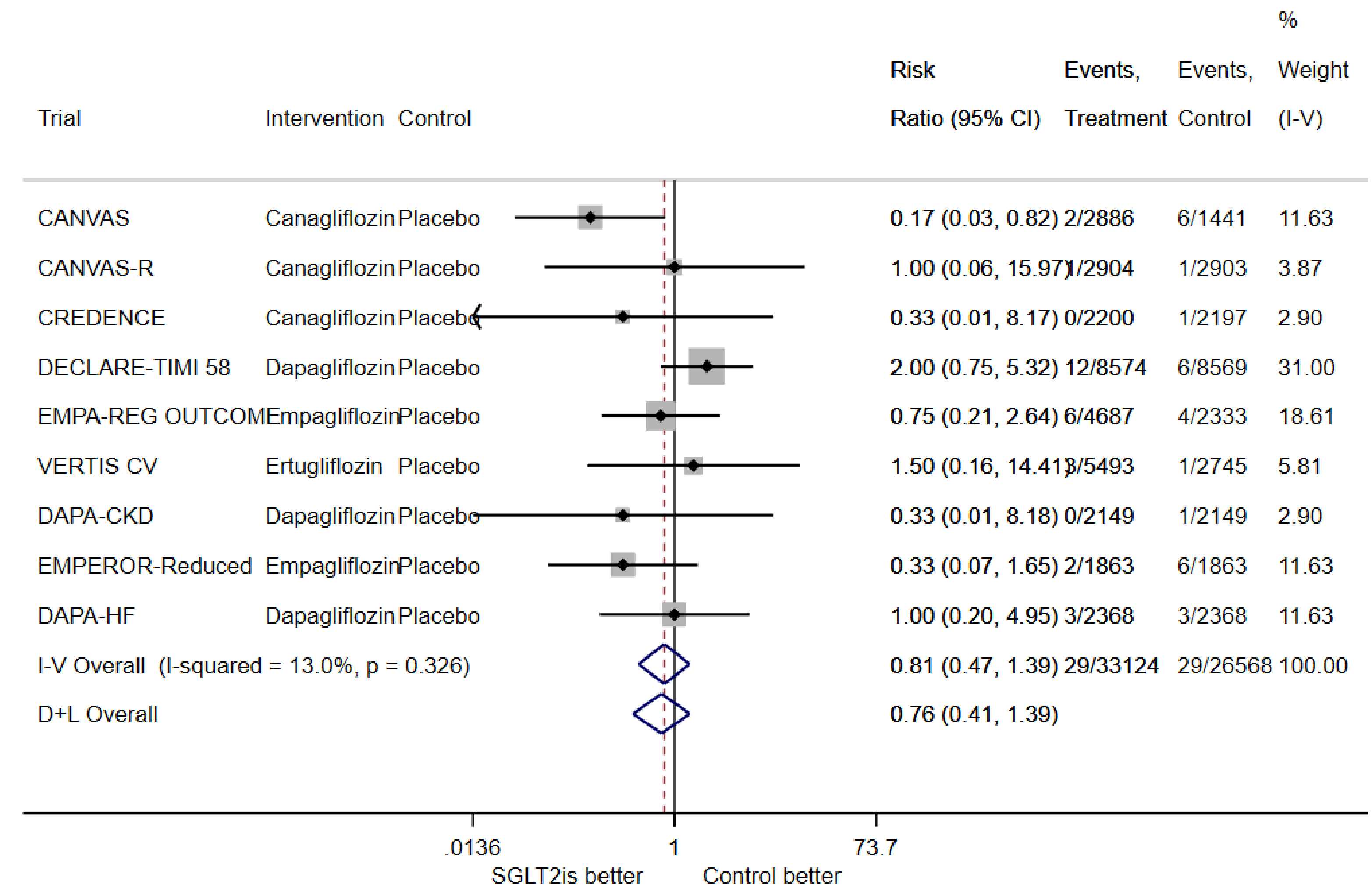

Figure S71 Meta-analysis of SGLT2is and Supraventricular tachycardia

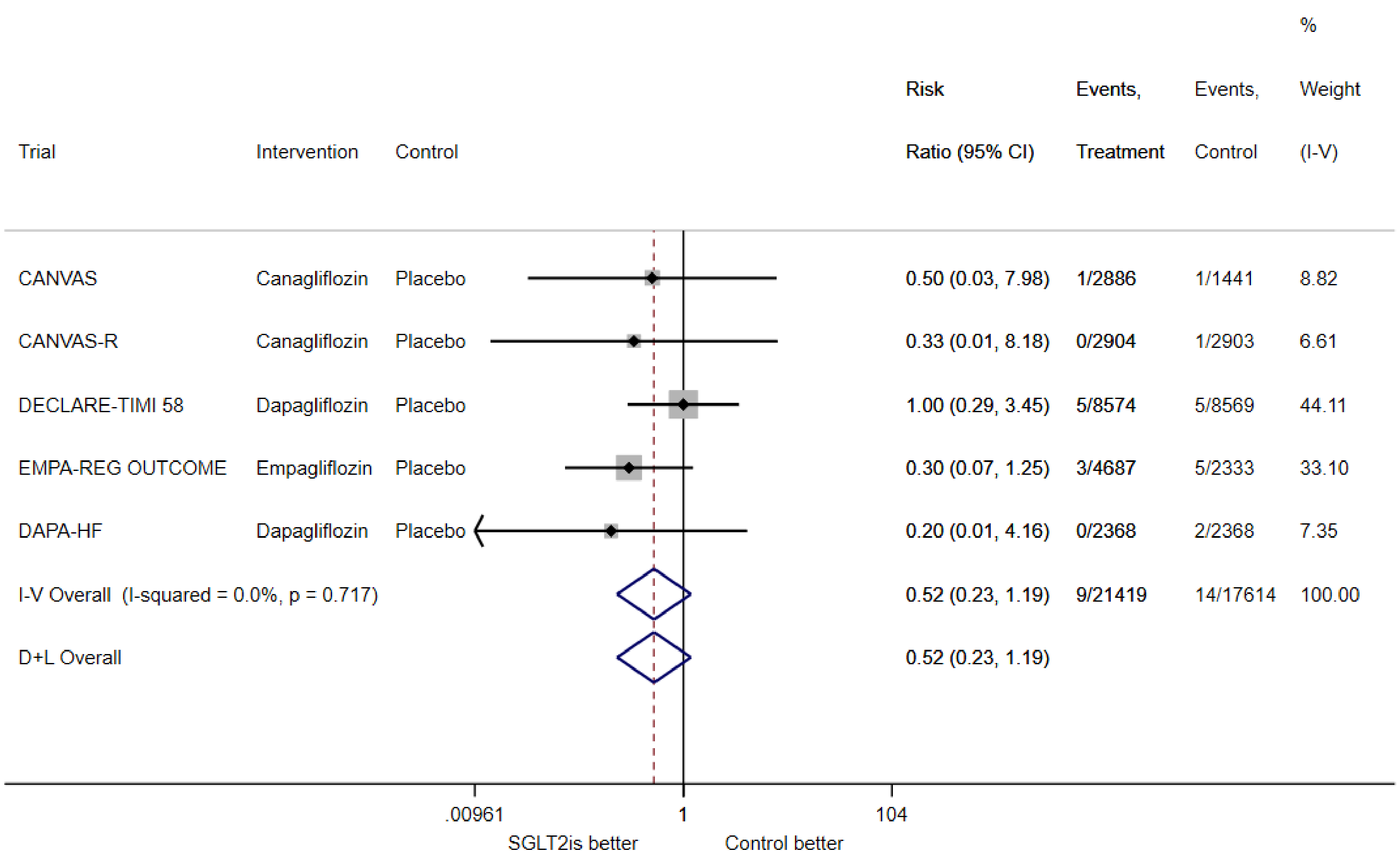

Figure S72 Meta-analysis of SGLT2is and Tachycardia

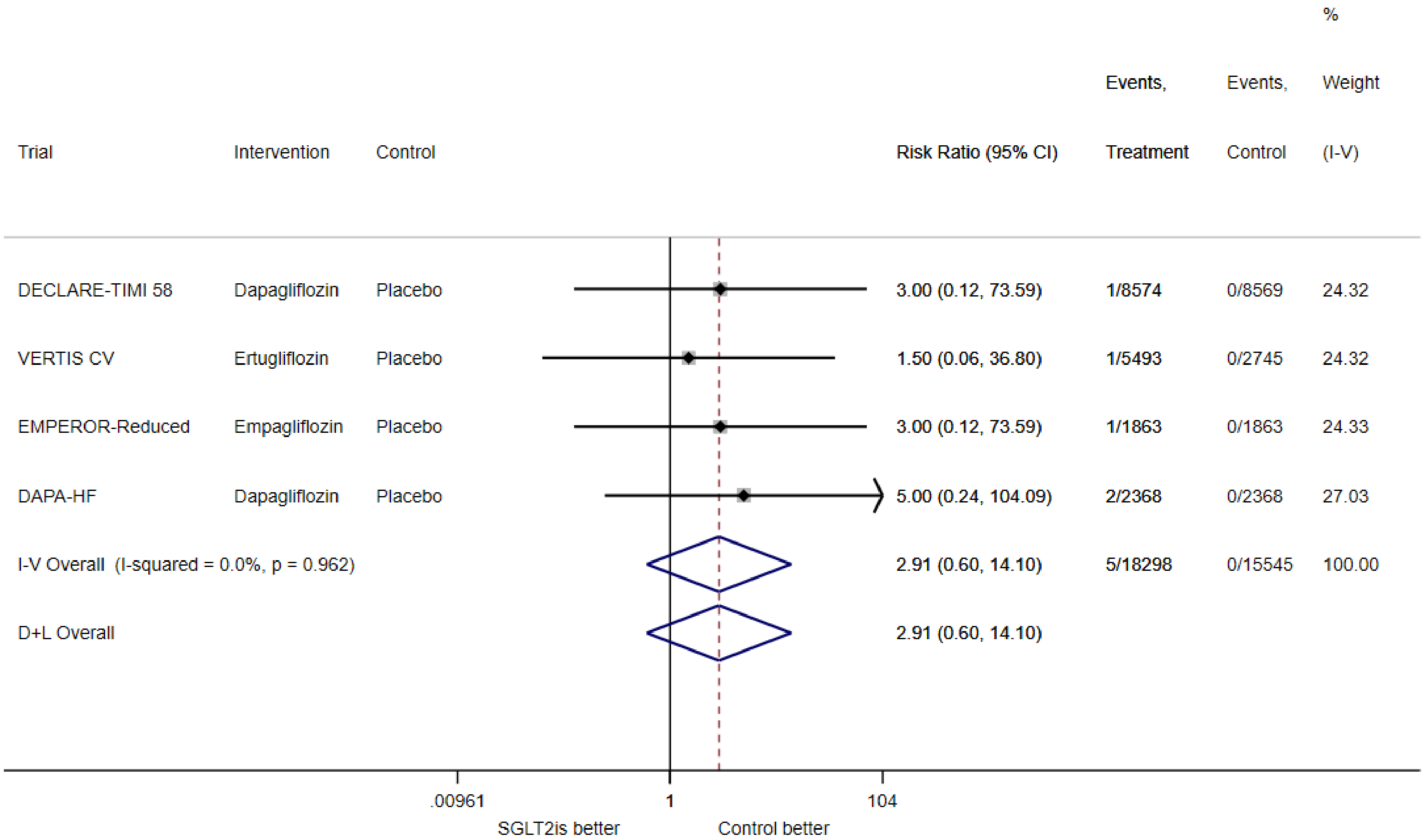

Figure S73 Meta-analysis of SGLT2is and Torsade de pointes

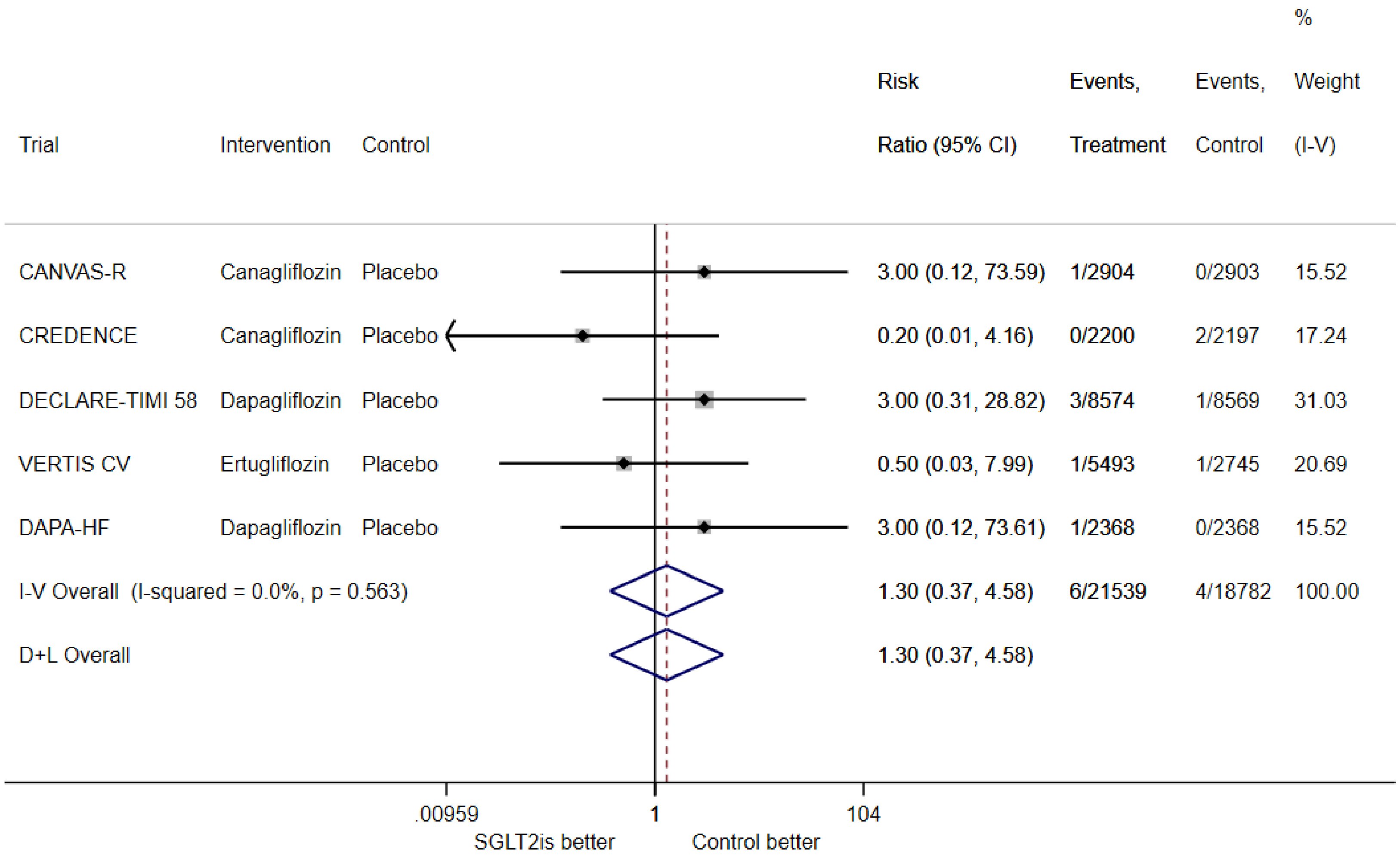

Figure S74 Meta-analysis of SGLT2is and Tricuspid valve incompetence

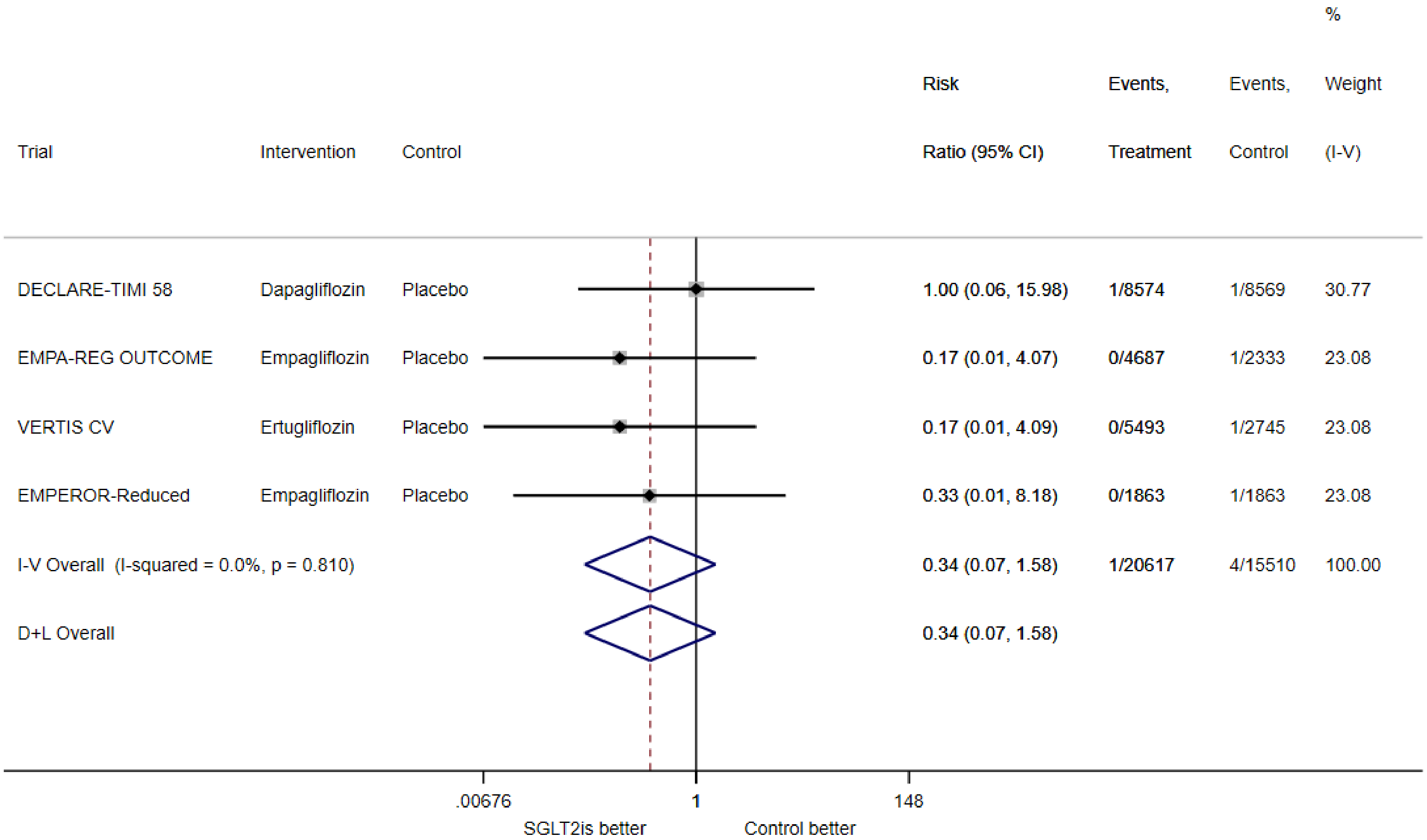

Figure S75 Meta-analysis of SGLT2is and Trifascicular block

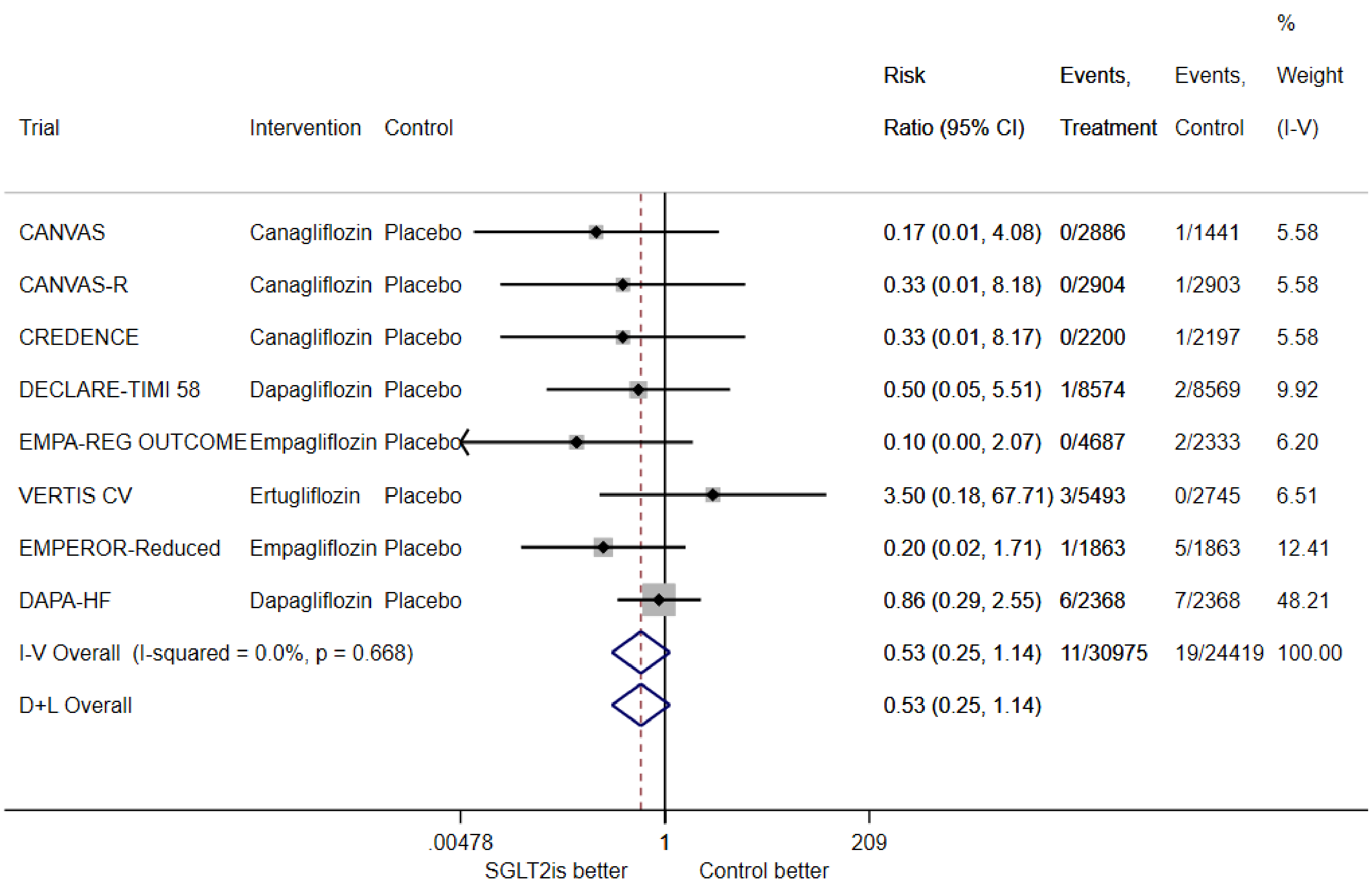

Figure S76 Meta-analysis of SGLT2is and Ventricular arrhythmia

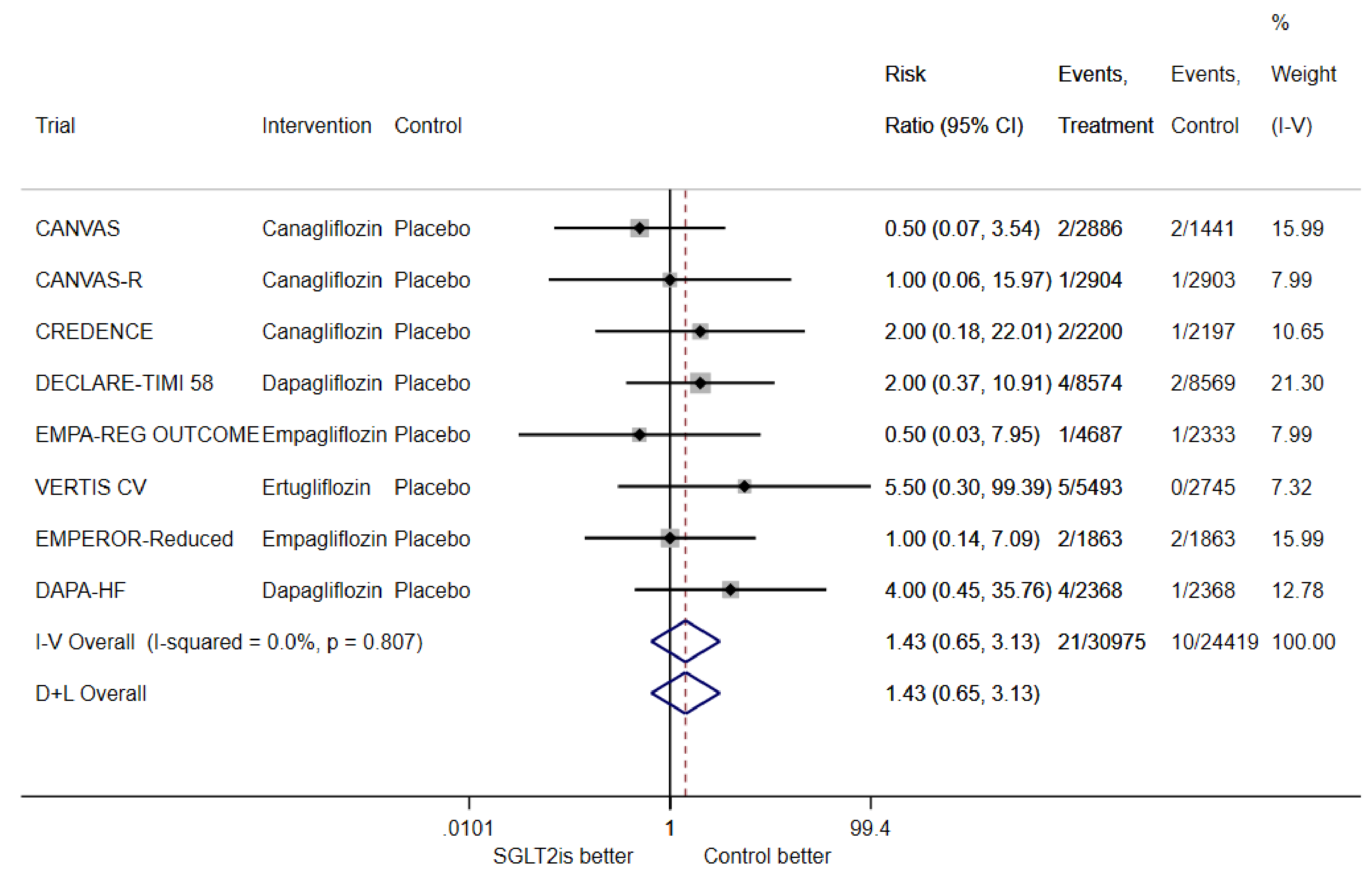

Figure S77 Meta-analysis of SGLT2is and Ventricular extrasystoles

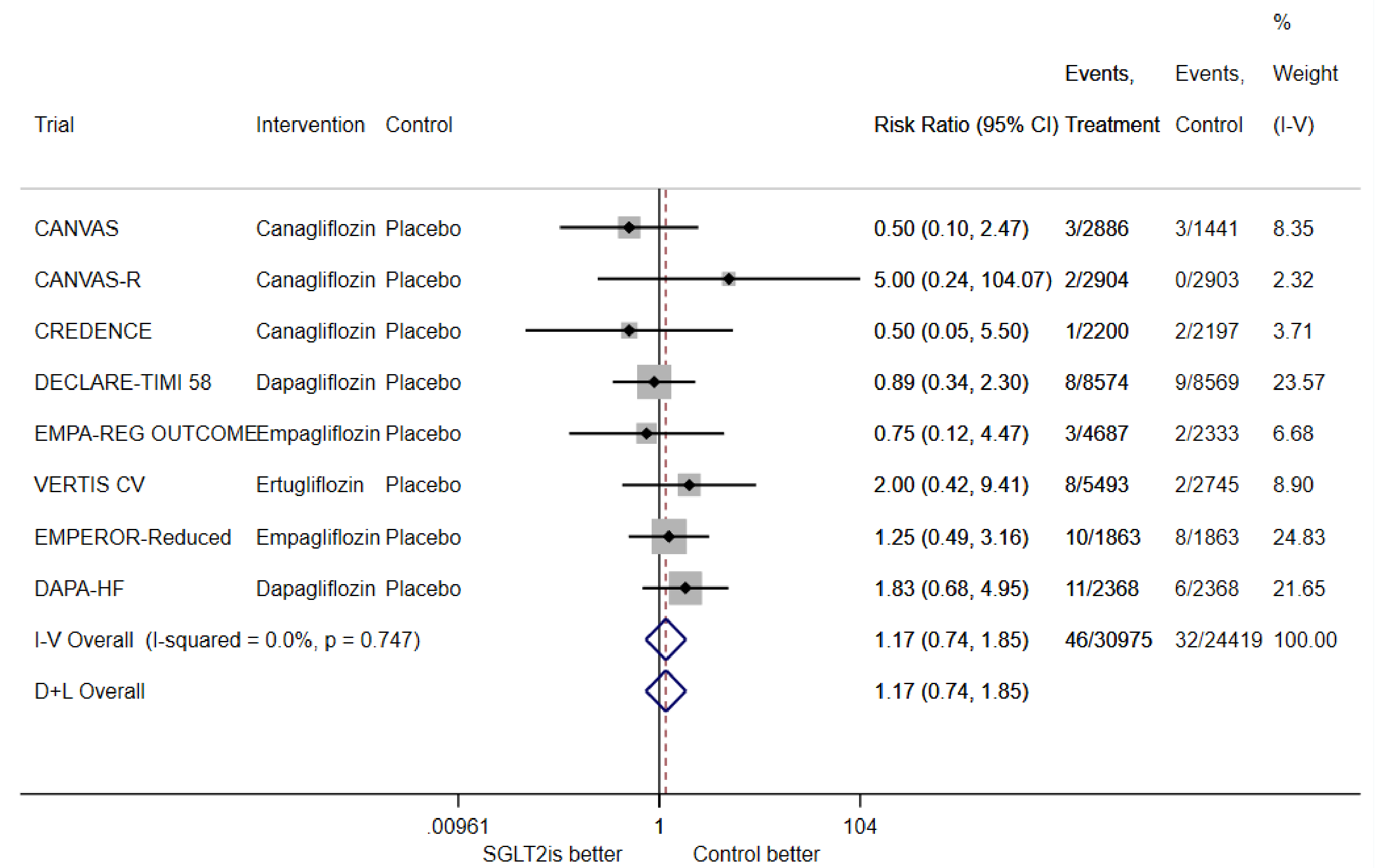

Figure S78 Meta-analysis of SGLT2is and Ventricular fibrillation

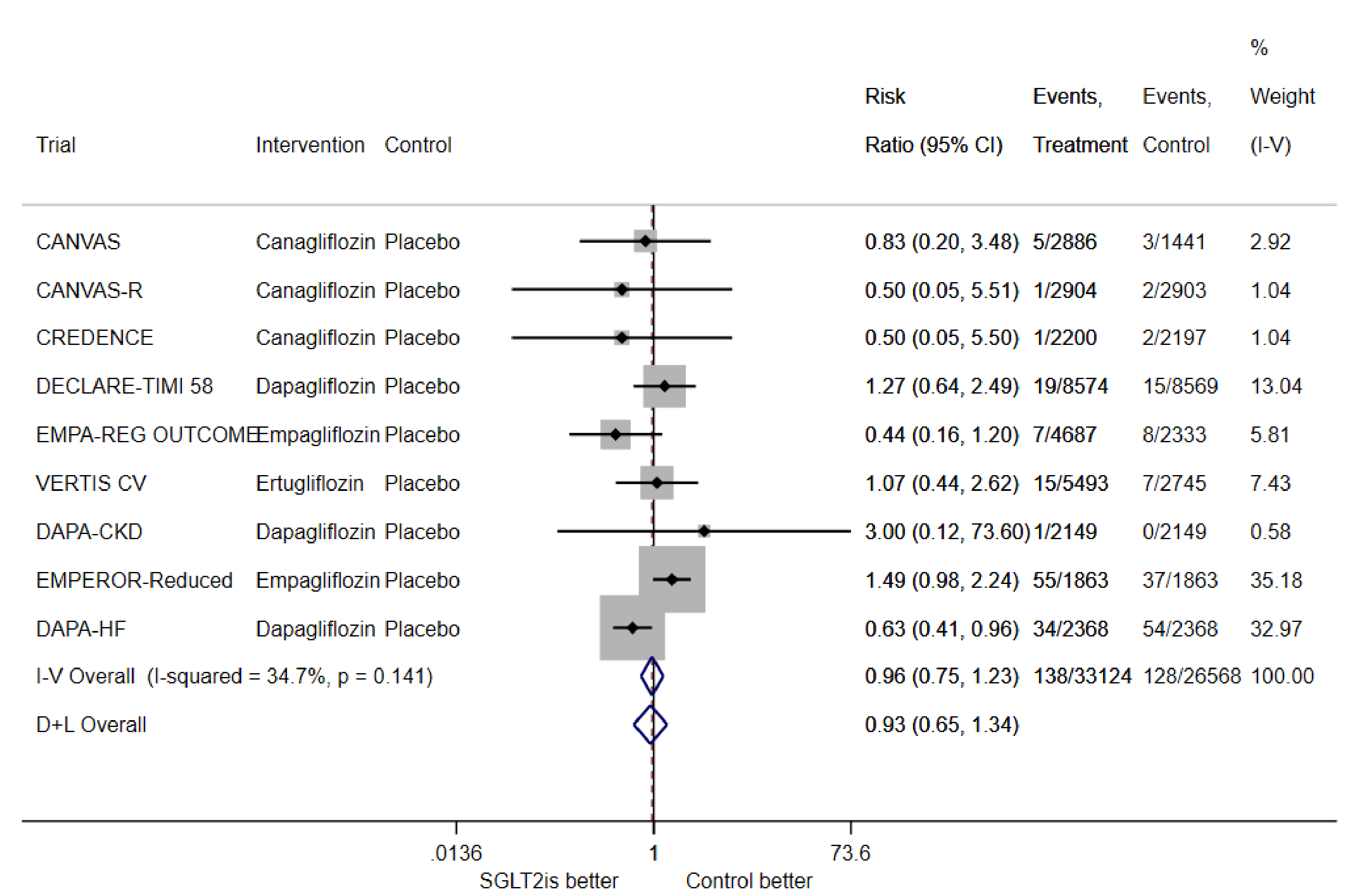

Figure S79 Meta-analysis of SGLT2is and Ventricular tachycardia

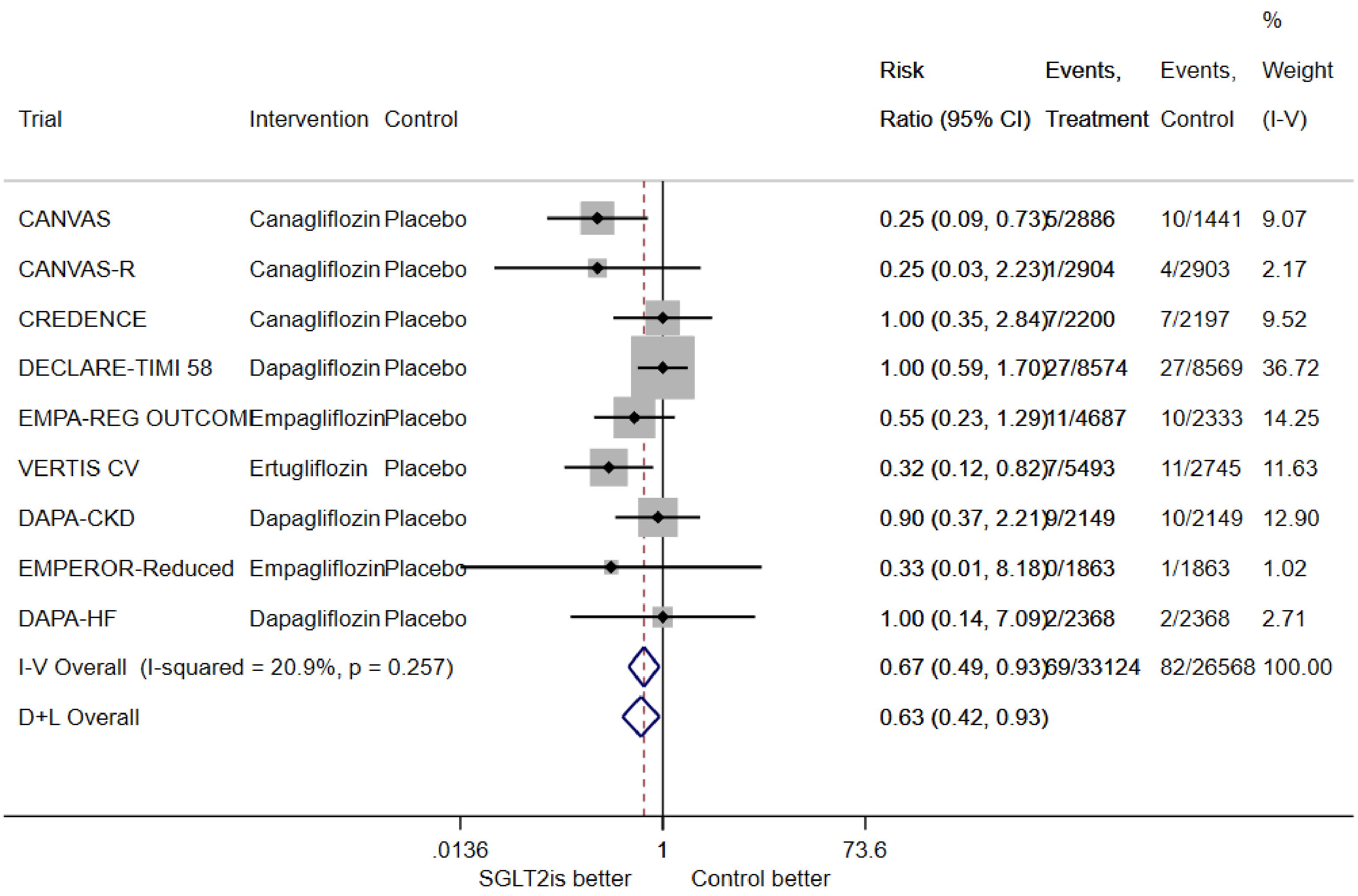

Figure S80 Meta-analysis of SGLT2is and Hypertension

Trial	Intervention	Control	Risk Ratio (95% CI)	Events, Treatment	Events, Control	% Weight (I-V)
CANVAS	Canagliflozin	Placebo	0.50 (0.03, 7.98)	1/2886	1/1441	10.48
CREDENCE	Canagliflozin	Placebo	0.50 (0.05, 5.50)	1/2200	2/2197	13.98
DECLARE-TIMI 58	Dapagliflozin	Placebo	0.29 (0.06, 1.37)	2/8574	7/8569	32.61
EMPA-REG OUTCOME	Empagliflozin	Placebo	0.10 (0.00, 2.07)	0/4687	2/2333	8.73
VERTIS CV	Ertugliflozin	Placebo	1.50 (0.06, 36.80)	1/5493	0/2745	7.86
DAPA-CKD	Dapagliflozin	Placebo	0.08 (0.00, 1.36)	0/2149	6/2149	9.73
EMPEROR-Reduced	Empagliflozin	Placebo	0.33 (0.01, 8.18)	0/1863	1/1863	7.86
DAPA-HF	Dapagliflozin	Placebo	0.20 (0.01, 4.16)	0/2368	2/2368	8.74
I-V Overall (I-squared = 0.0%, p = 0.910)			0.29 (0.12, 0.72)	5/30220	21/23665	100.00
D+L Overall			0.29 (0.12, 0.72)			

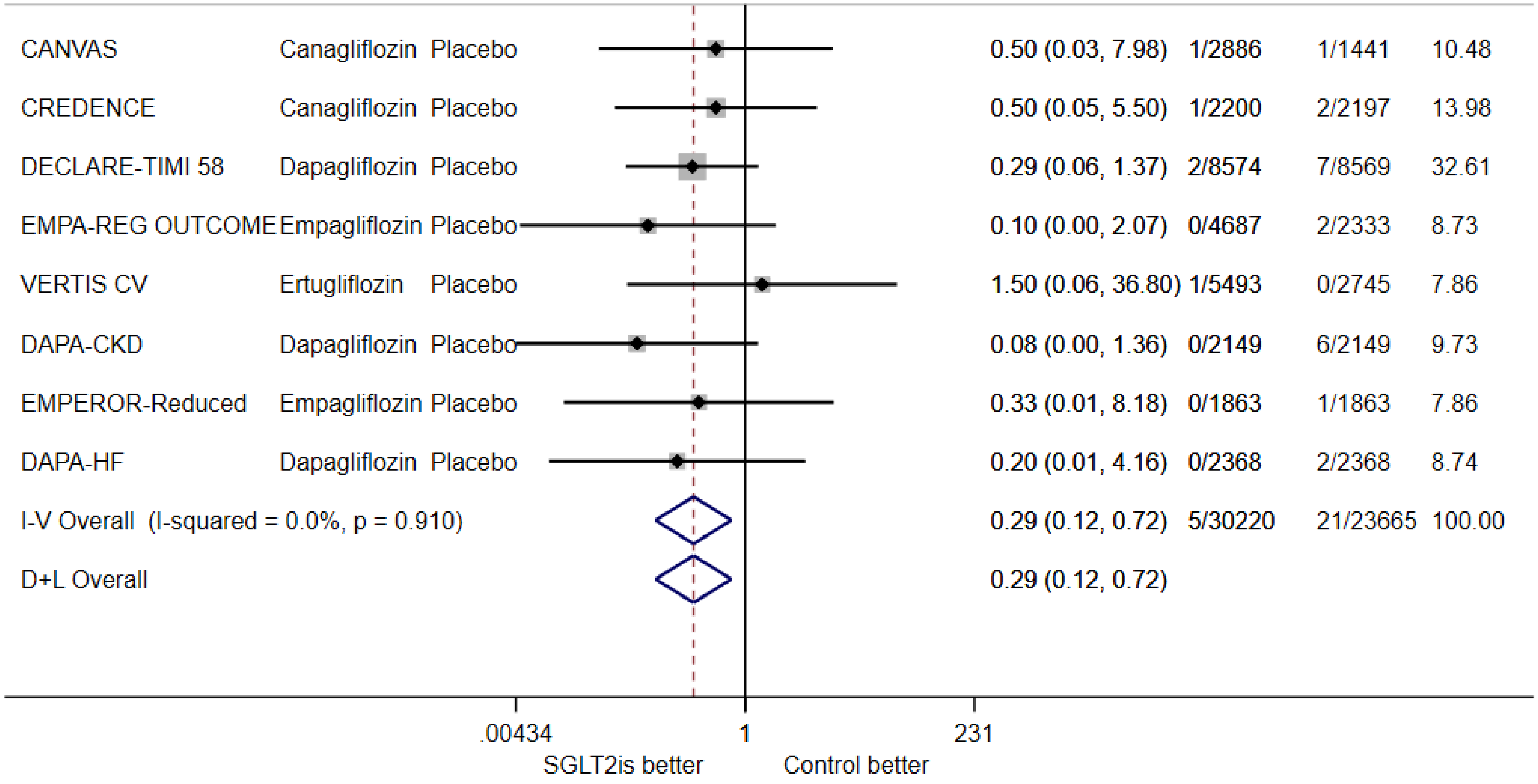

Figure S81 Meta-analysis of SGLT2is and Hypertensive emergency

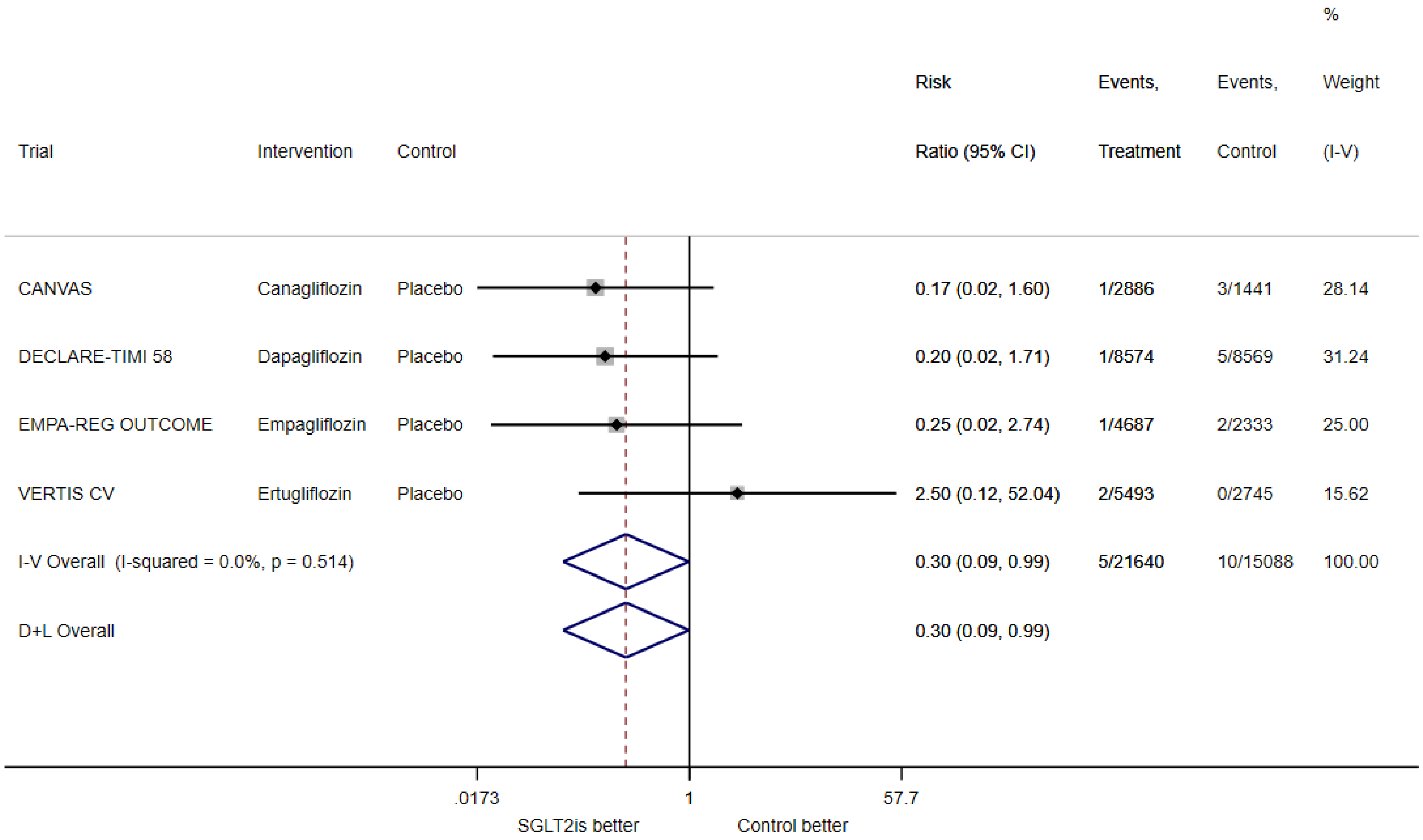

Figure S82 Meta-analysis of SGLT2is and Varicose vein

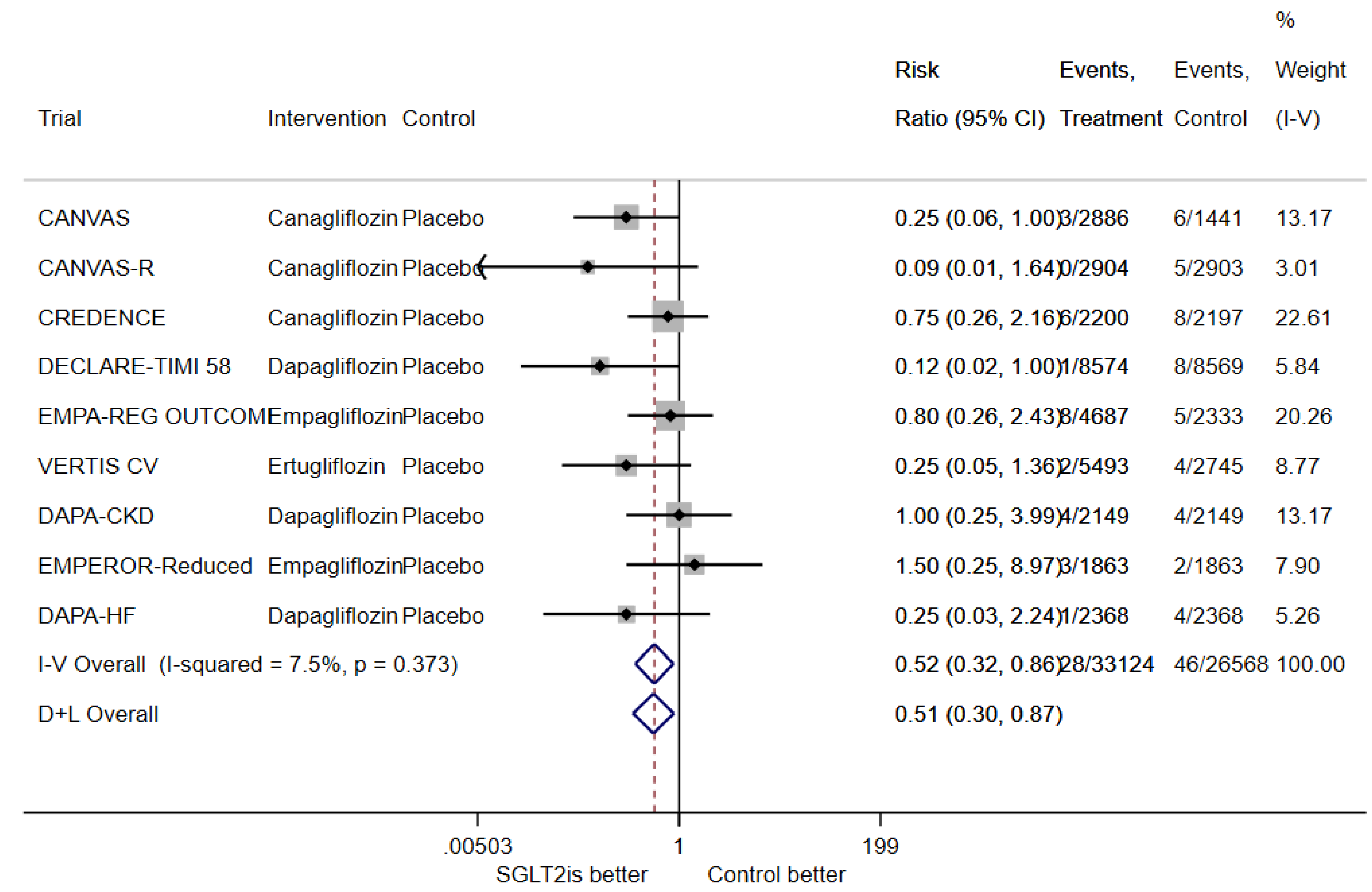

Figure S83 Meta-analysis of SGLT2is and Acute pulmonary oedema

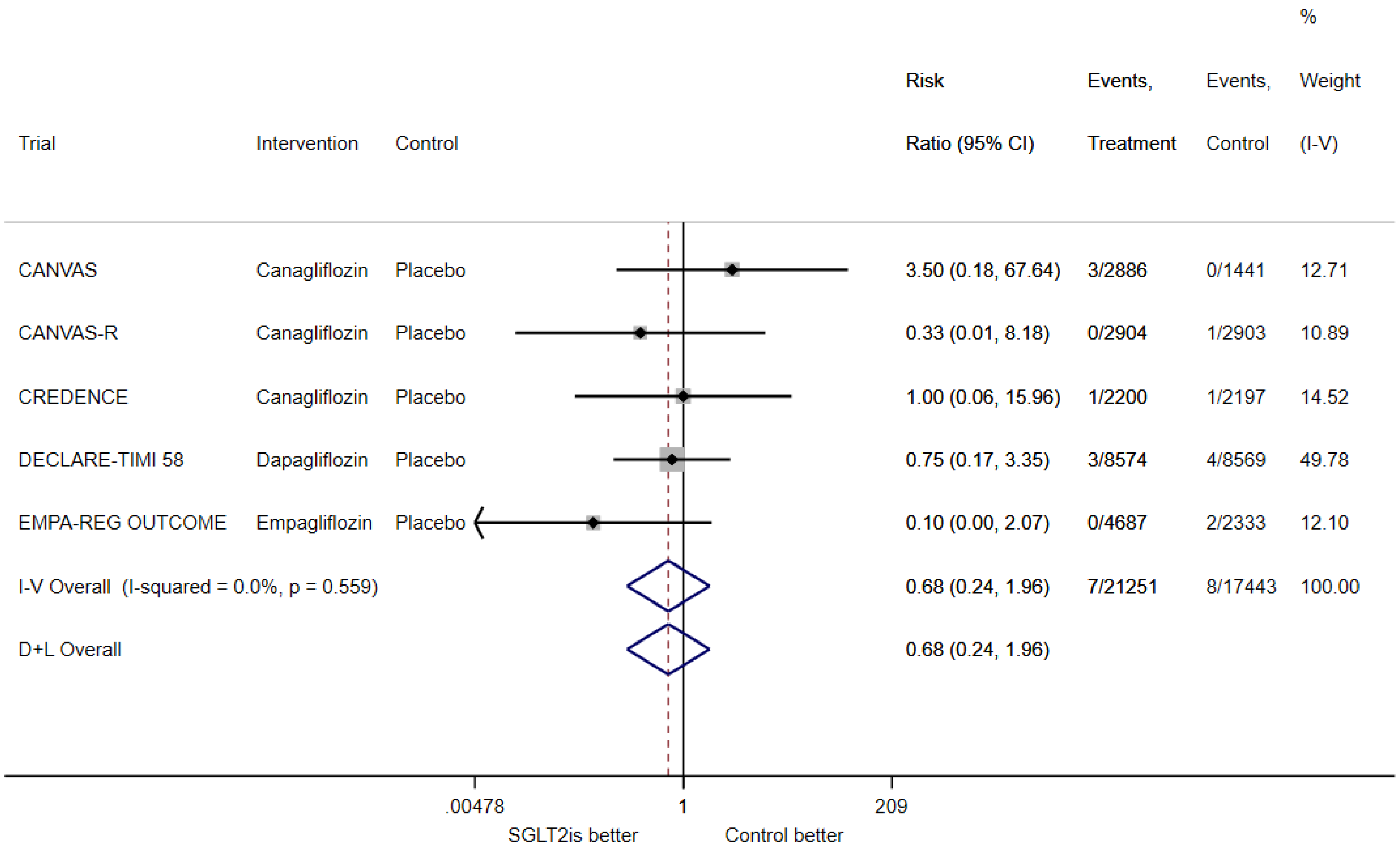

Figure S84 Meta-analysis of SGLT2is and Acute respiratory distress syndrome

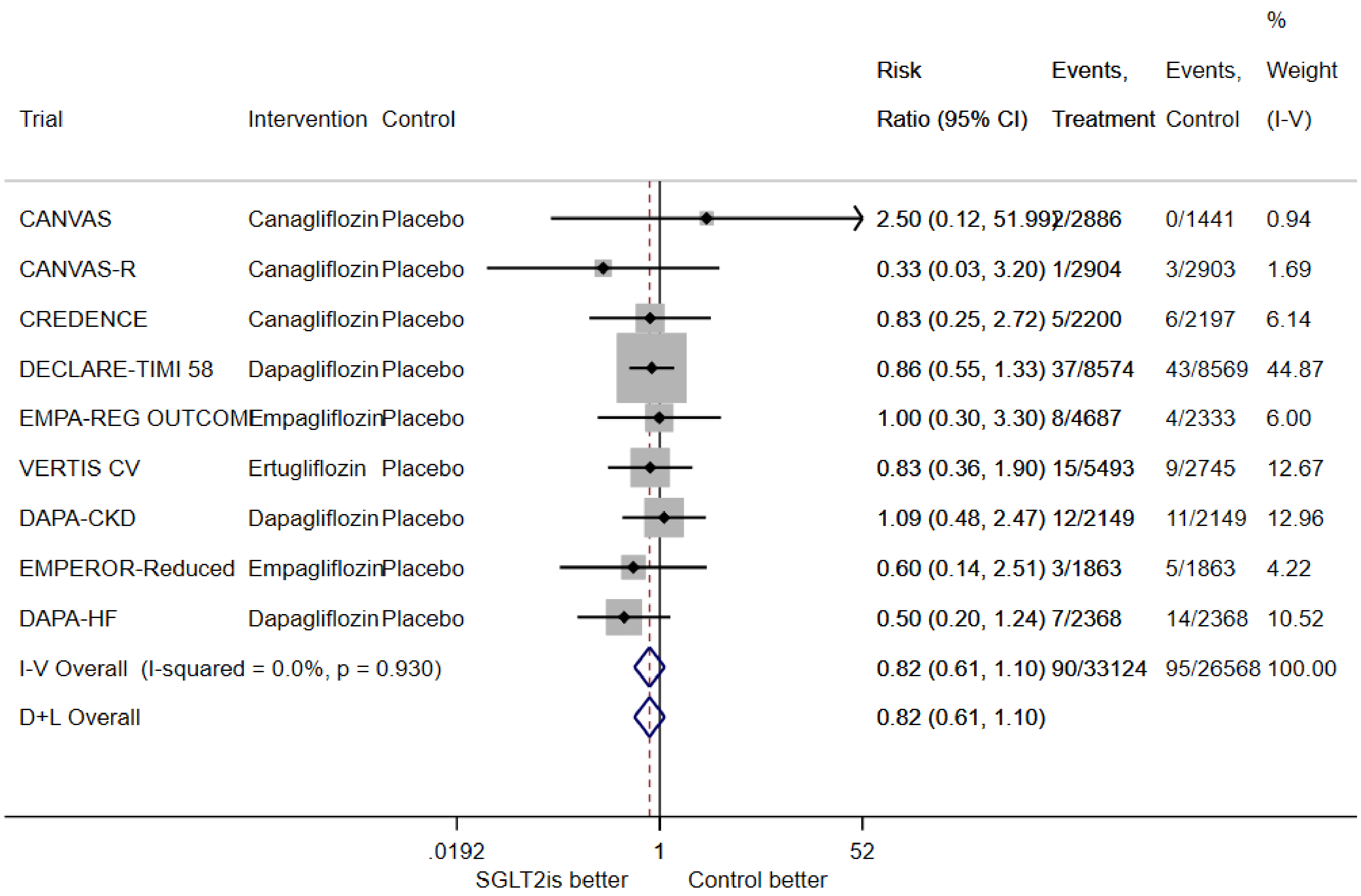

Figure S85 Meta-analysis of SGLT2is and Acute respiratory failure

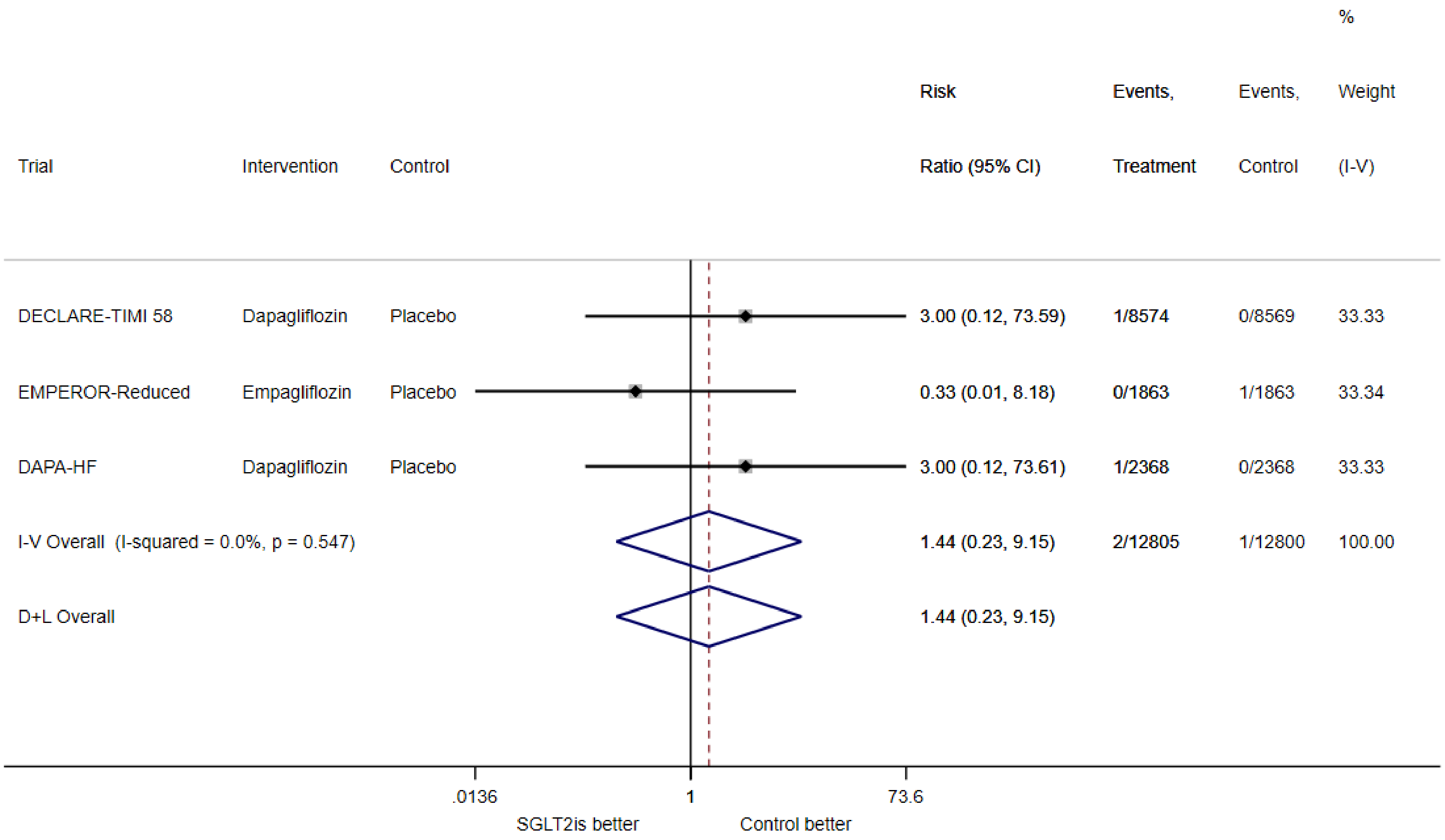

Figure S86 Meta-analysis of SGLT2is and Aspiration

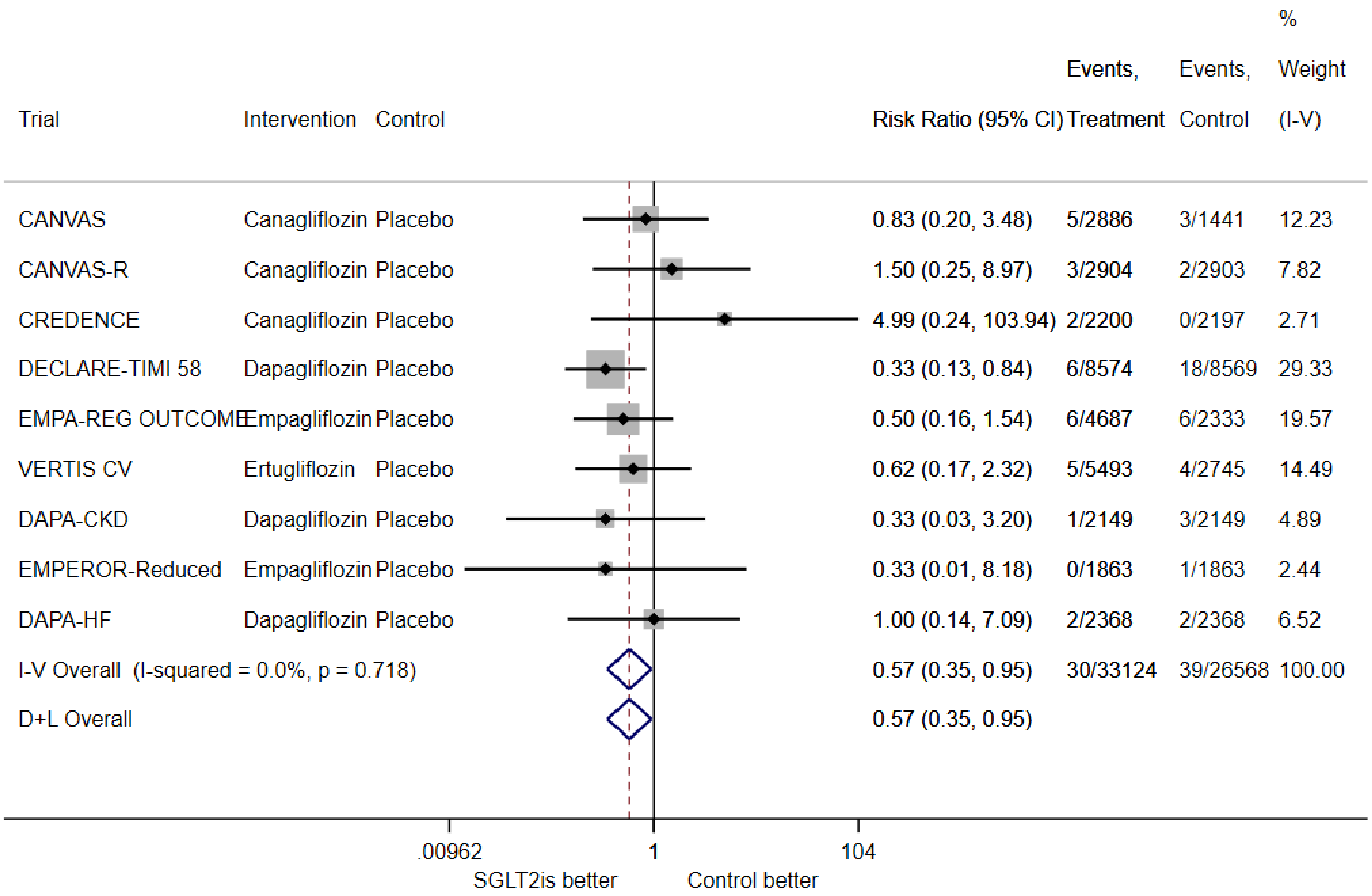

Figure S87 Meta-analysis of SGLT2is and Asthma

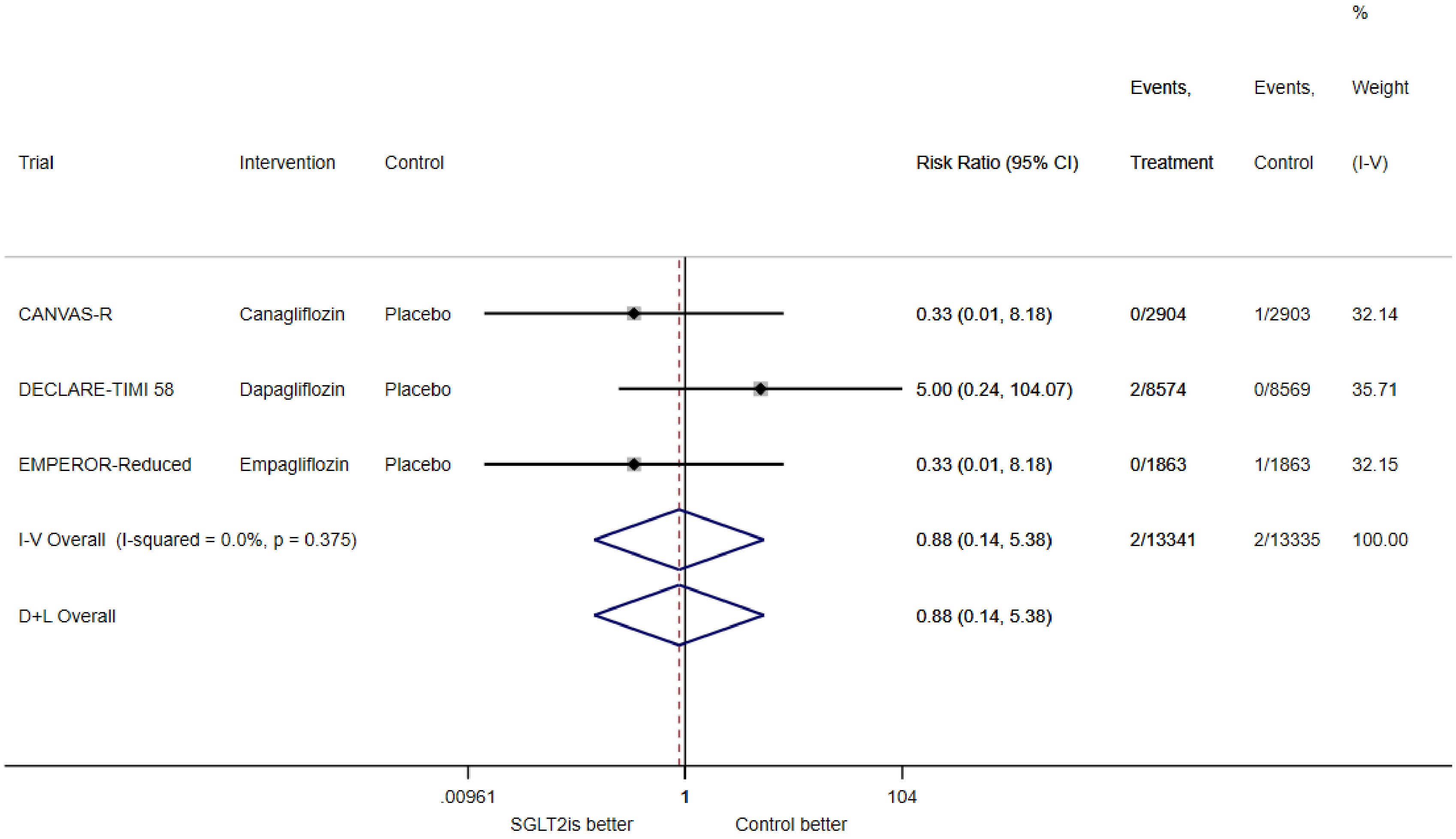

Figure S88 Meta-analysis of SGLT2is and Asthmatic crisis

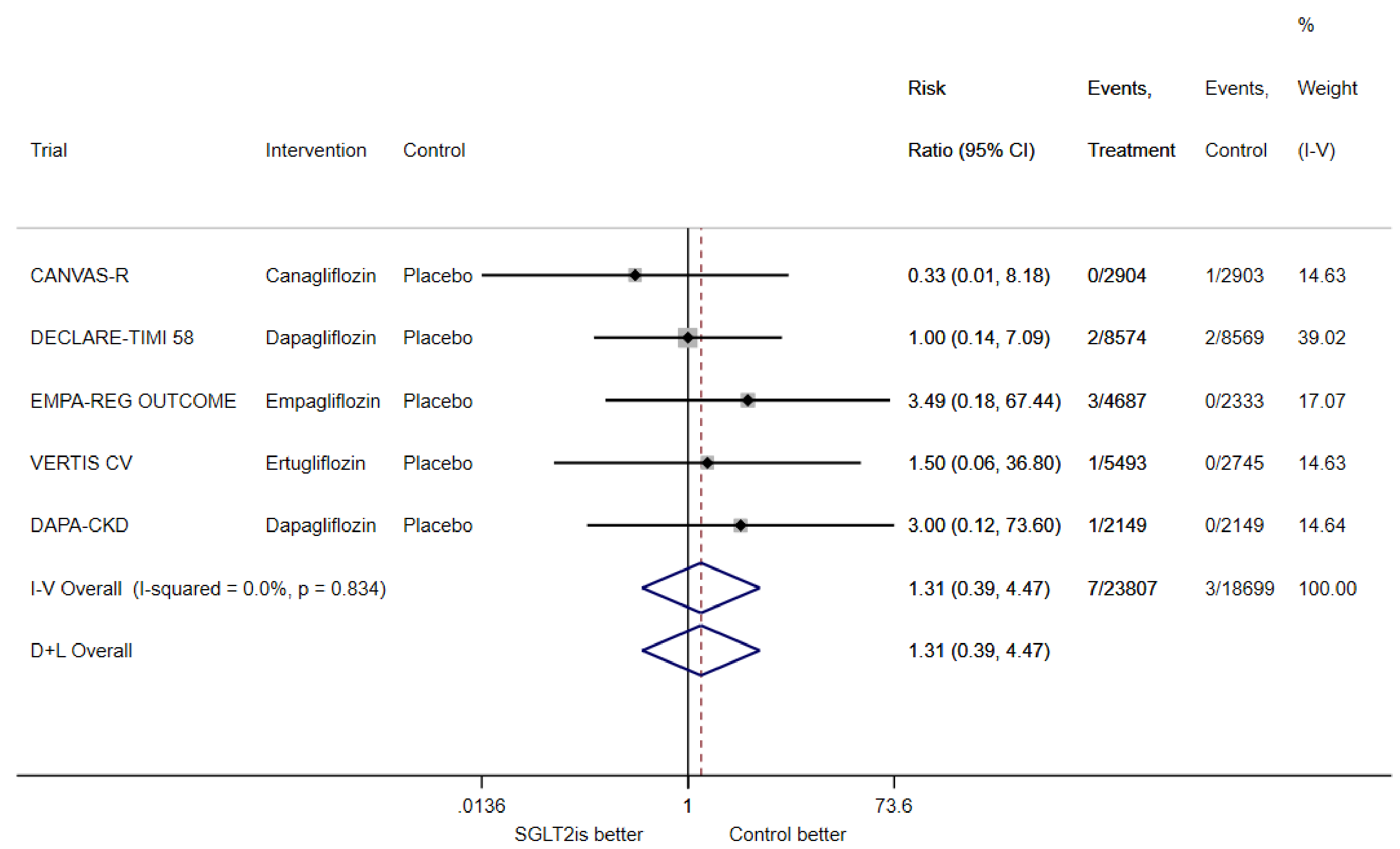

Figure S89 Meta-analysis of SGLT2is and Atelectasis

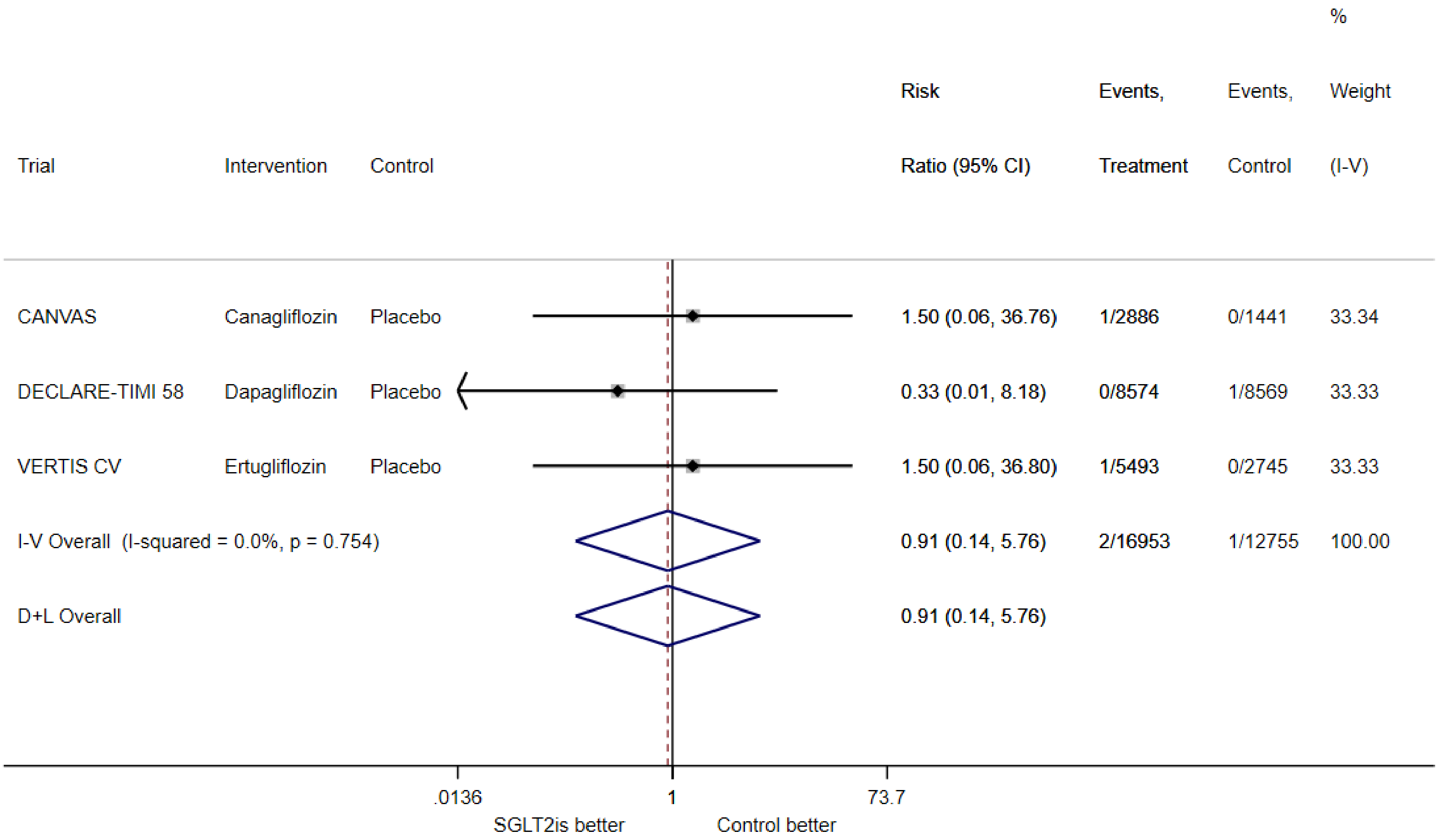

Figure S90 Meta-analysis of SGLT2is and Bronchial hyperreactivity

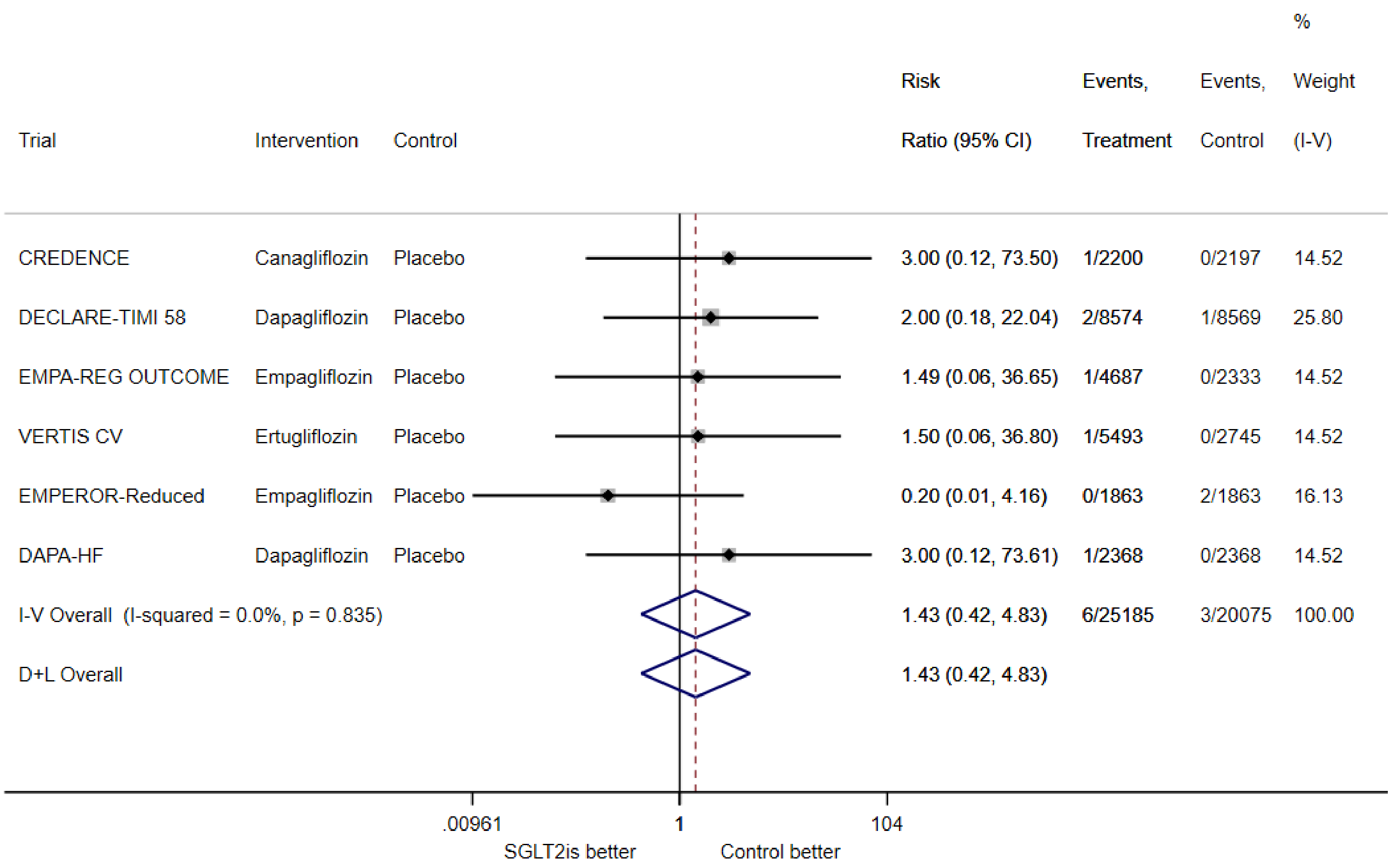

Figure S91 Meta-analysis of SGLT2is and Bronchiectasis

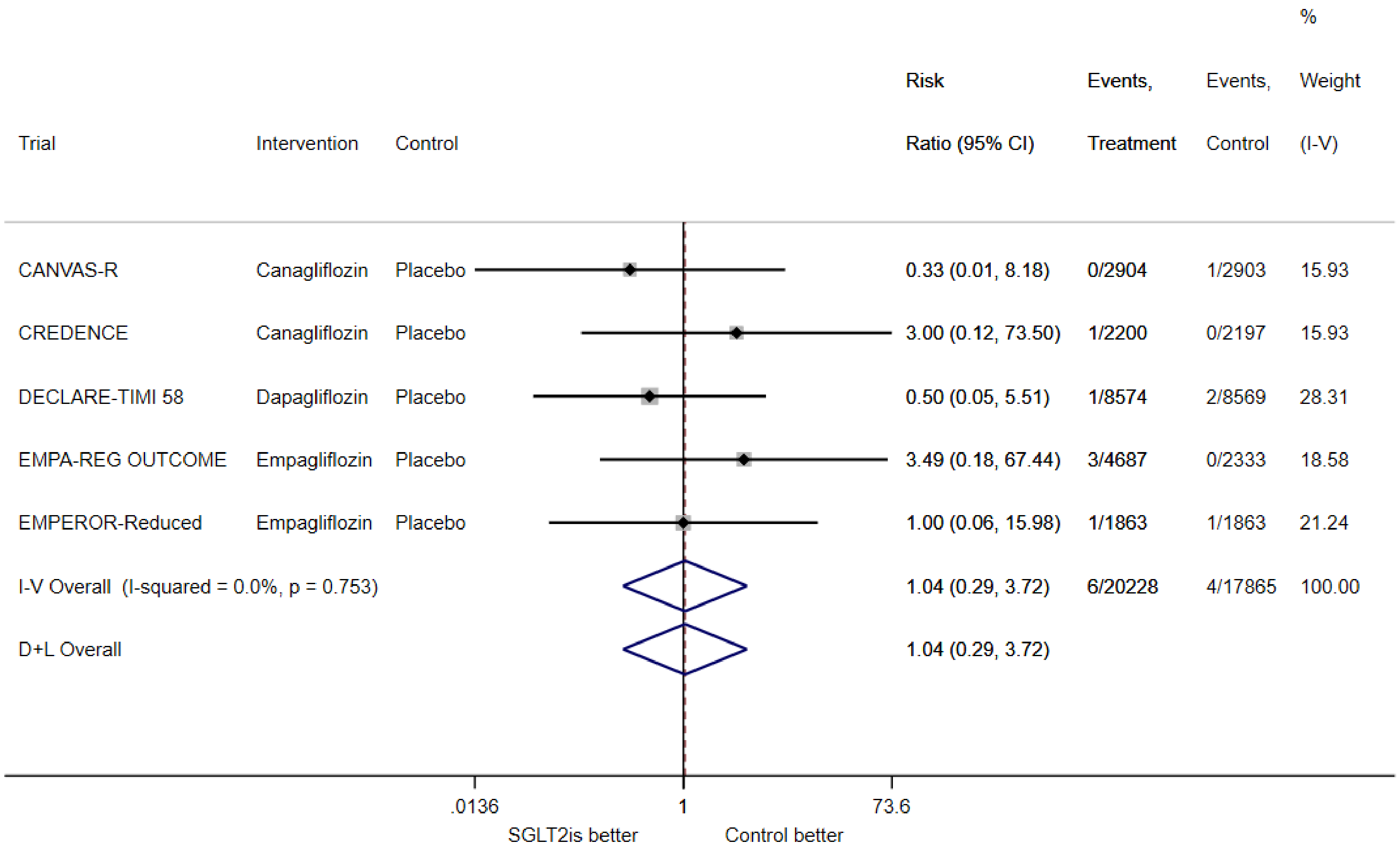

Figure S92 Meta-analysis of SGLT2is and Bronchitis chronic

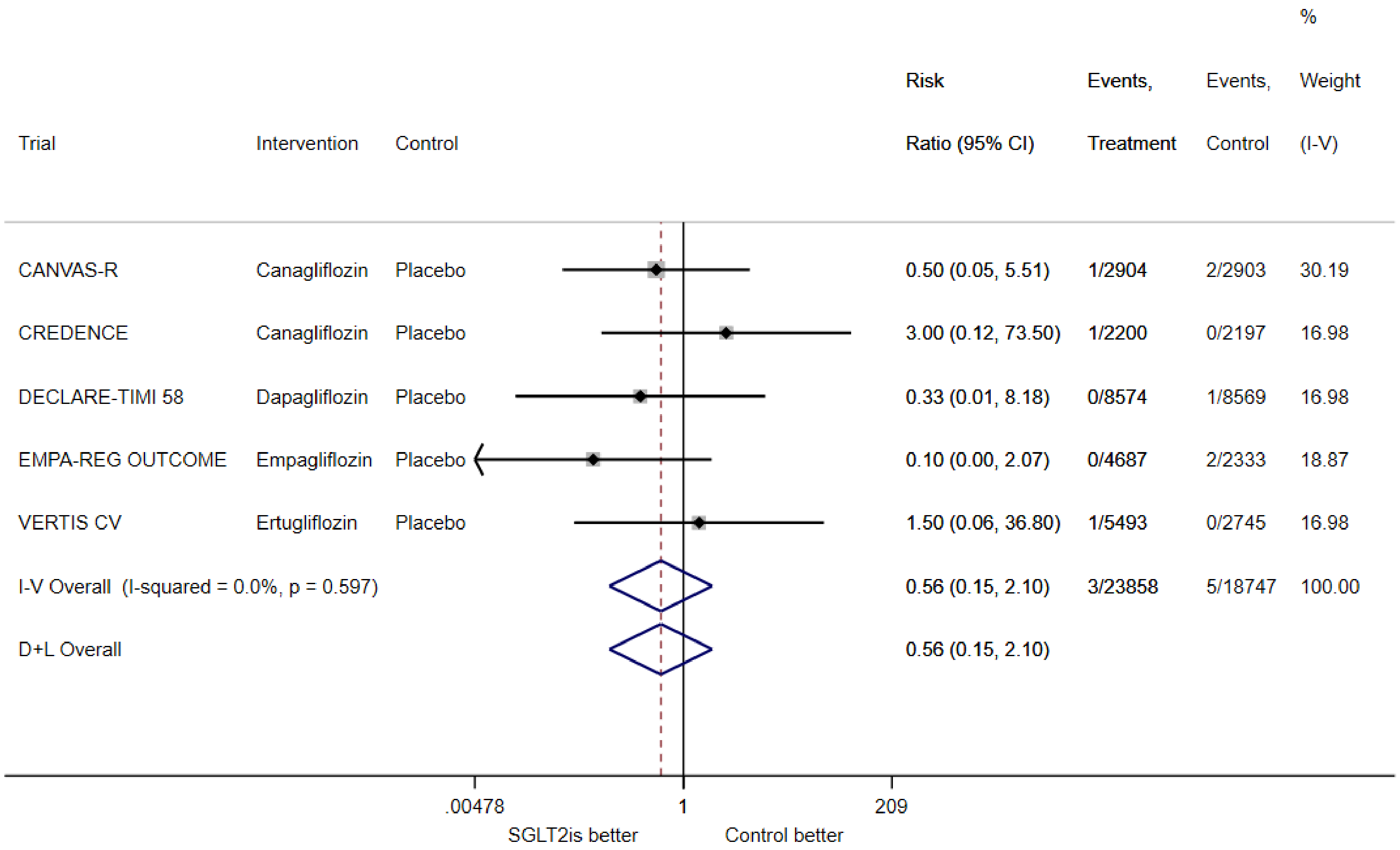

Figure S93 Meta-analysis of SGLT2is and Bronchospasm

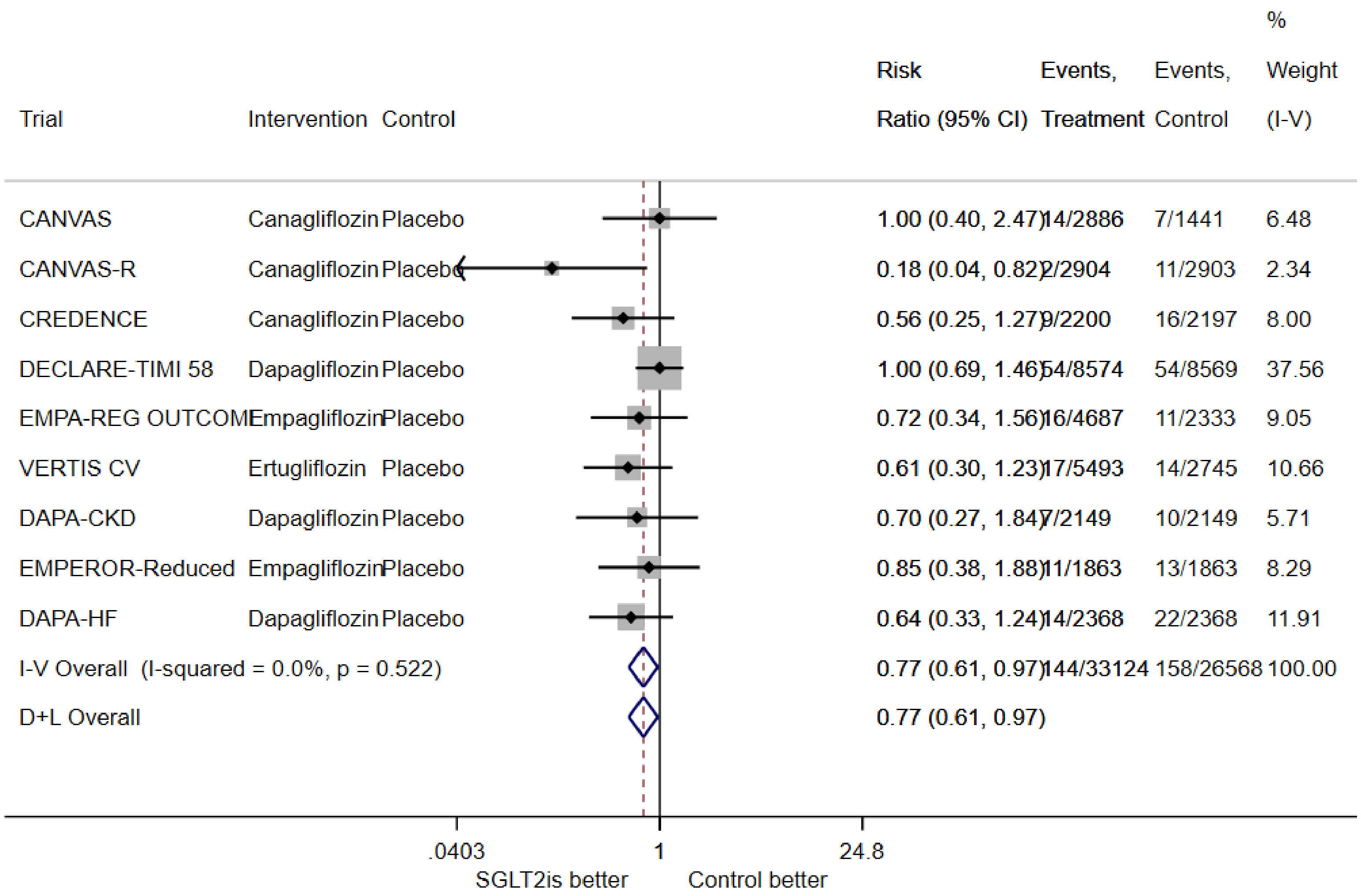

Figure S94 Meta-analysis of SGLT2is and Chronic obstructive pulmonary disease

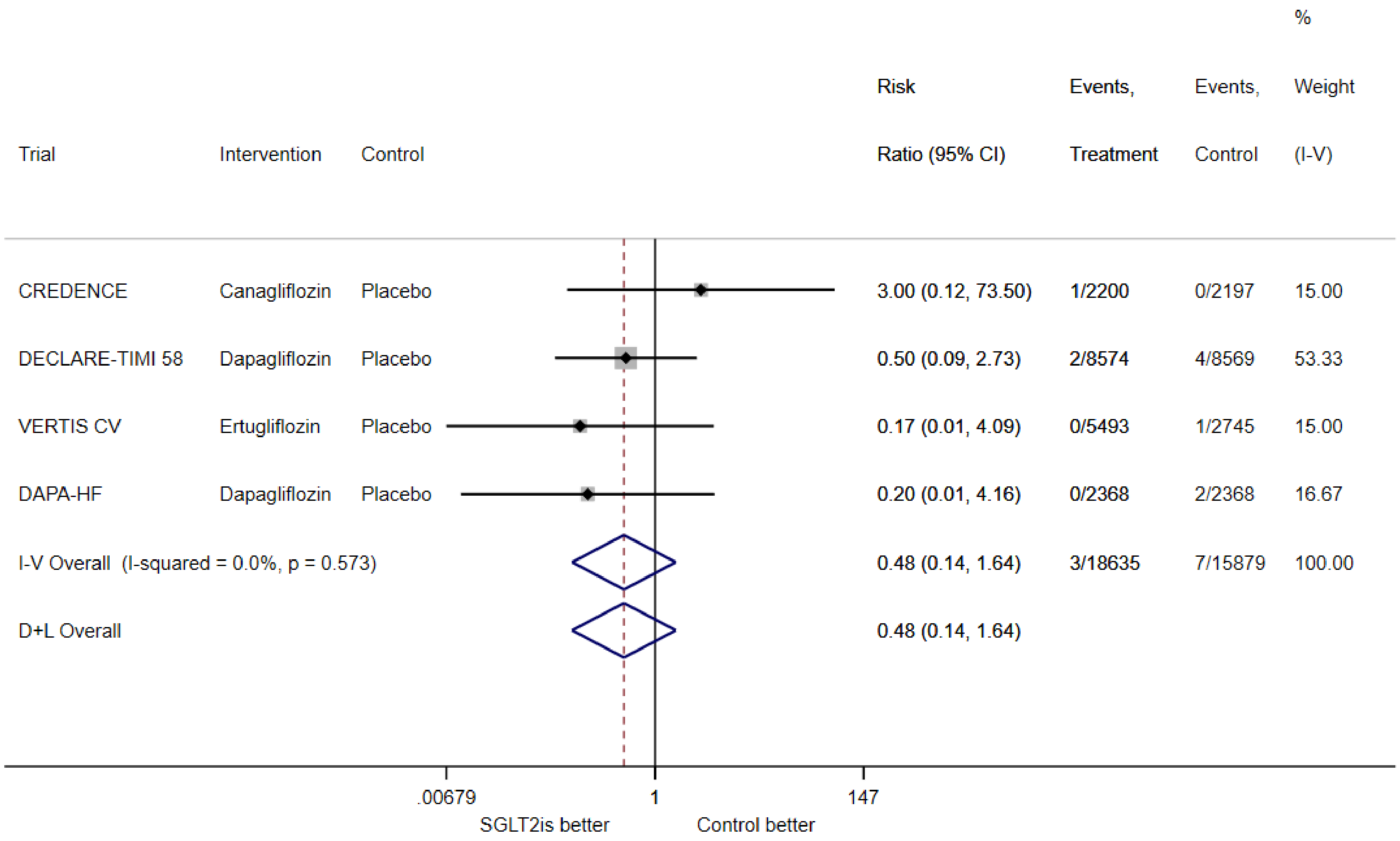

Figure S95 Meta-analysis of SGLT2is and Chronic respiratory failure

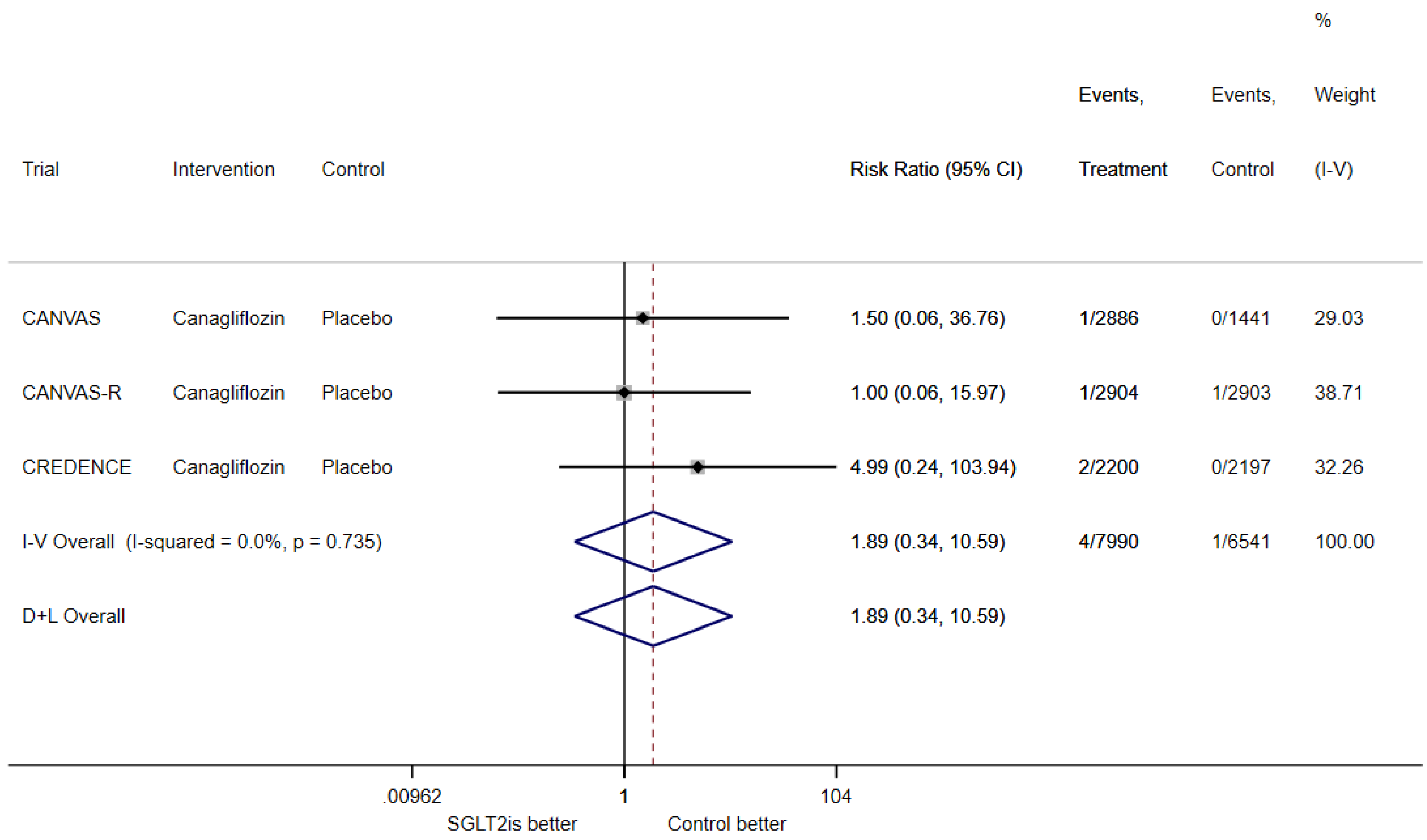

Figure S96 Meta-analysis of SGLT2is and Cough

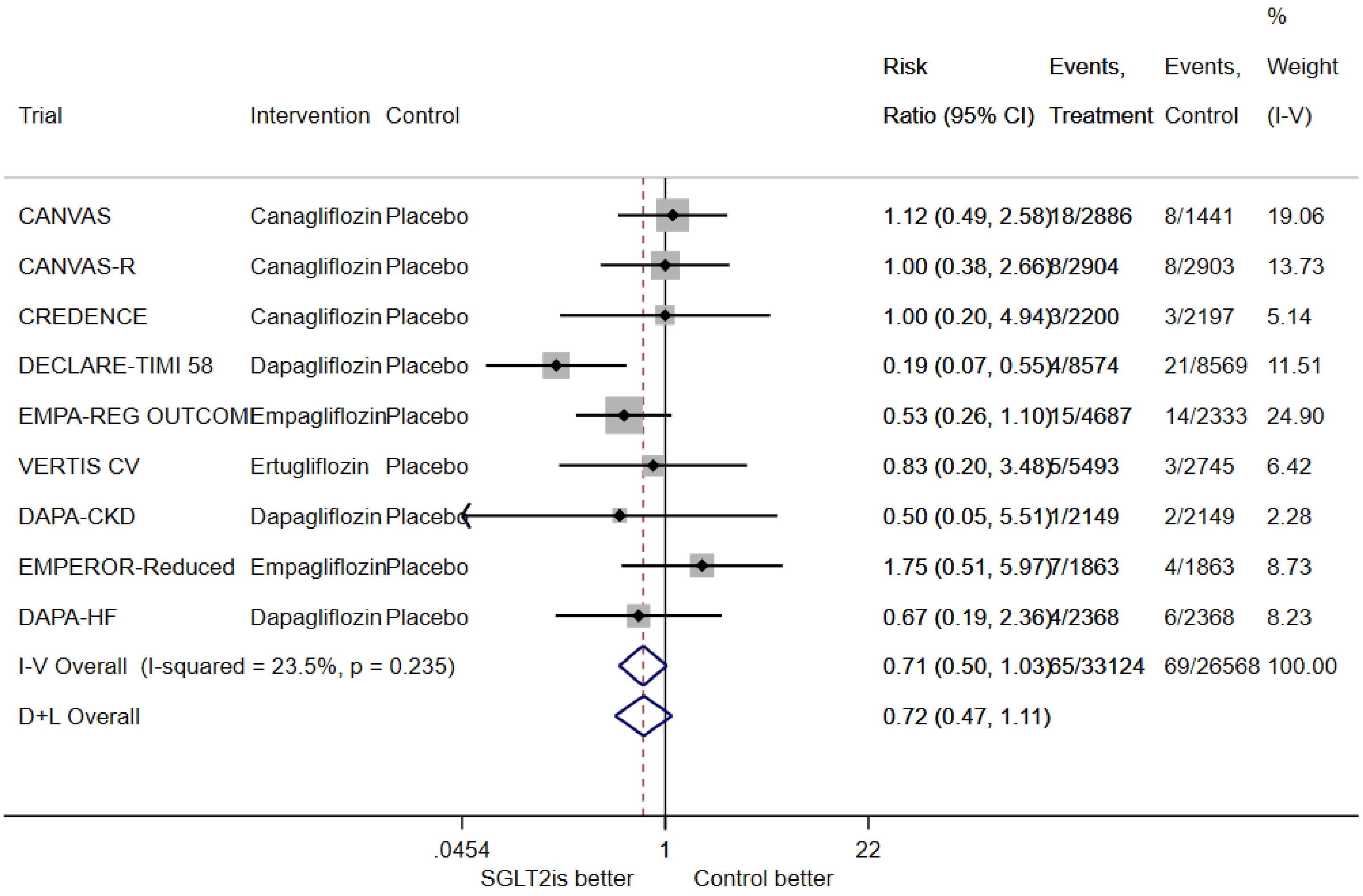

Figure S97 Meta-analysis of SGLT2is and Dyspnoea

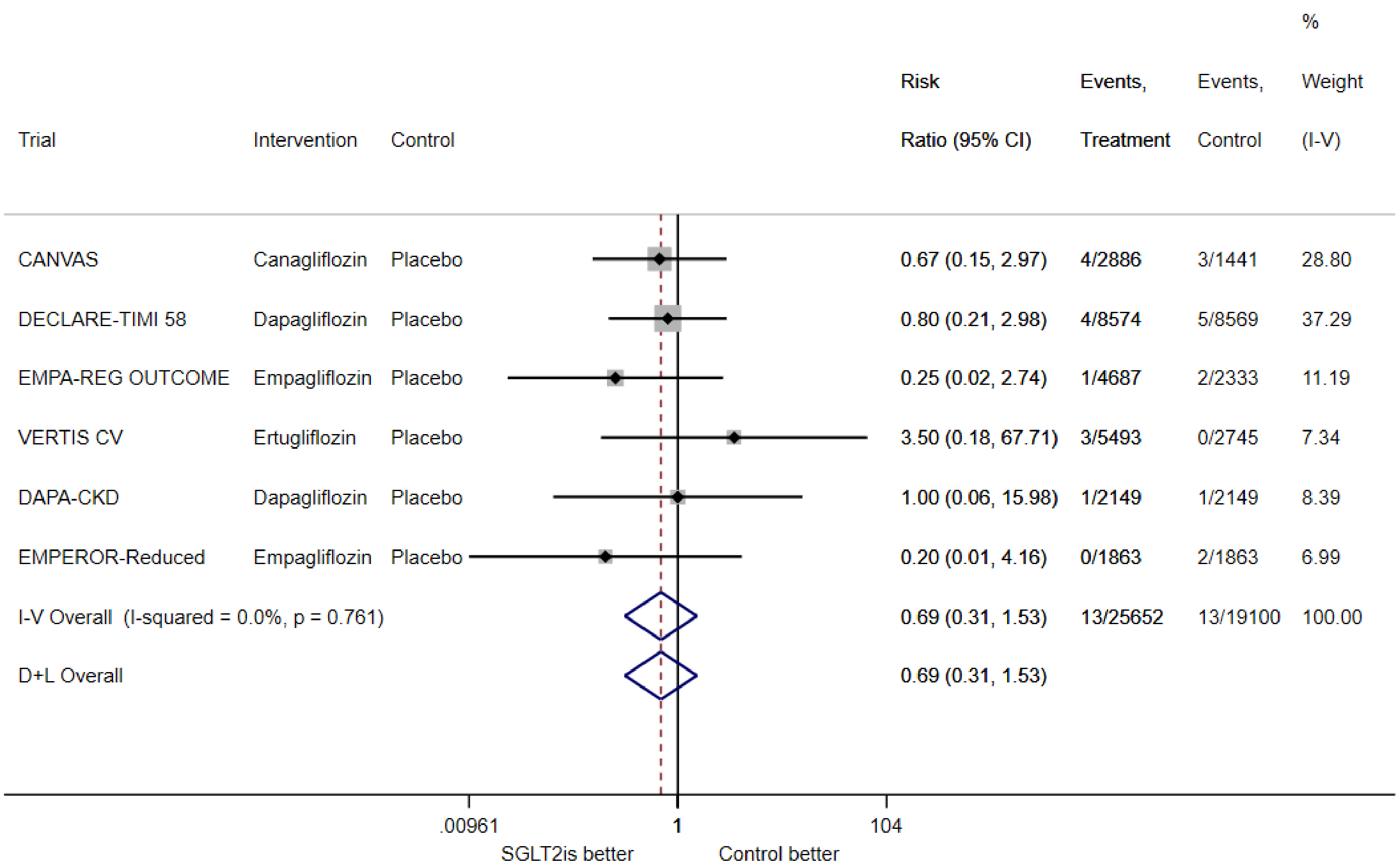

Figure S98 Meta-analysis of SGLT2is and Dyspnoea exertional

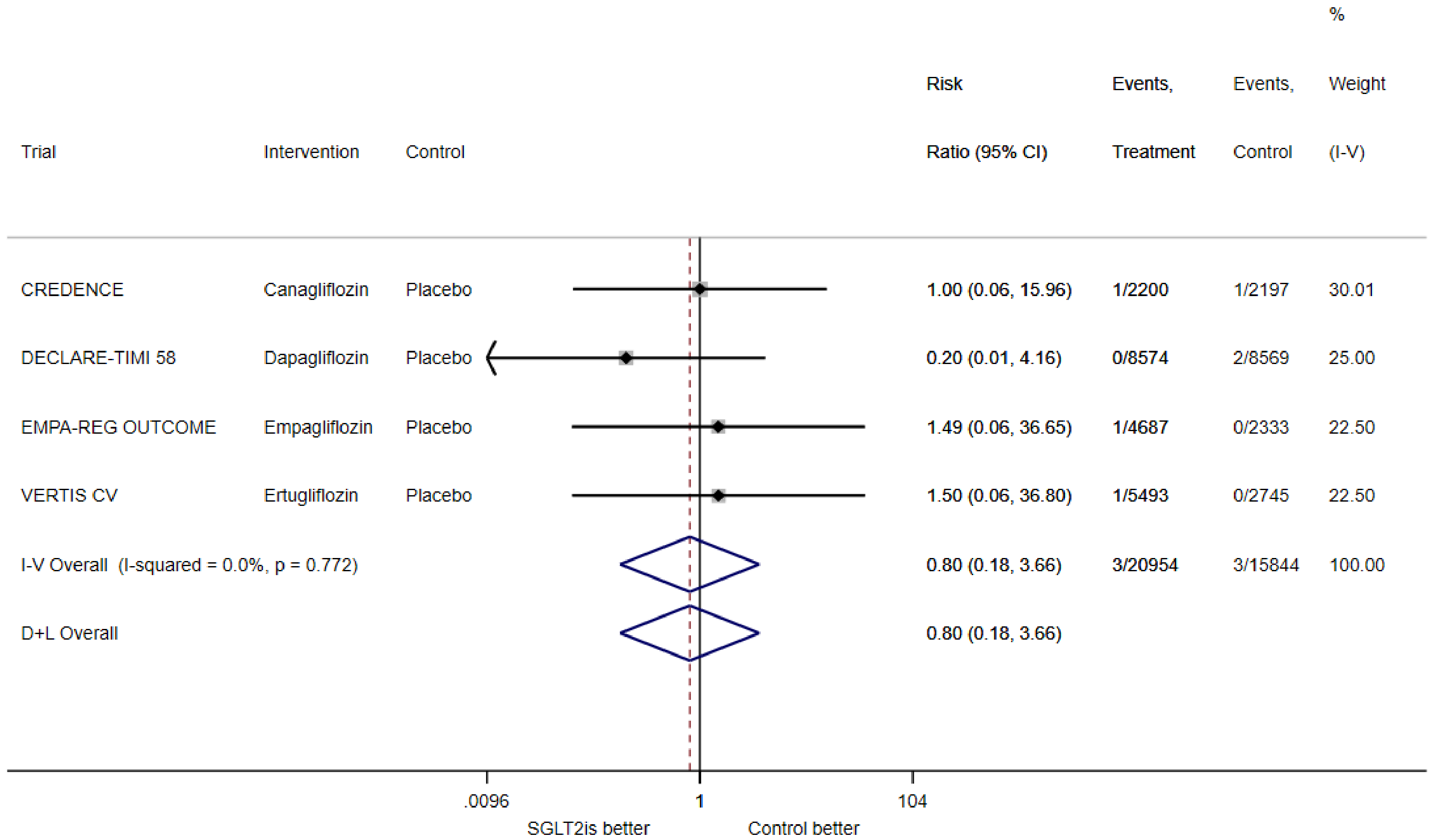

Figure S99 Meta-analysis of SGLT2is and Emphysema

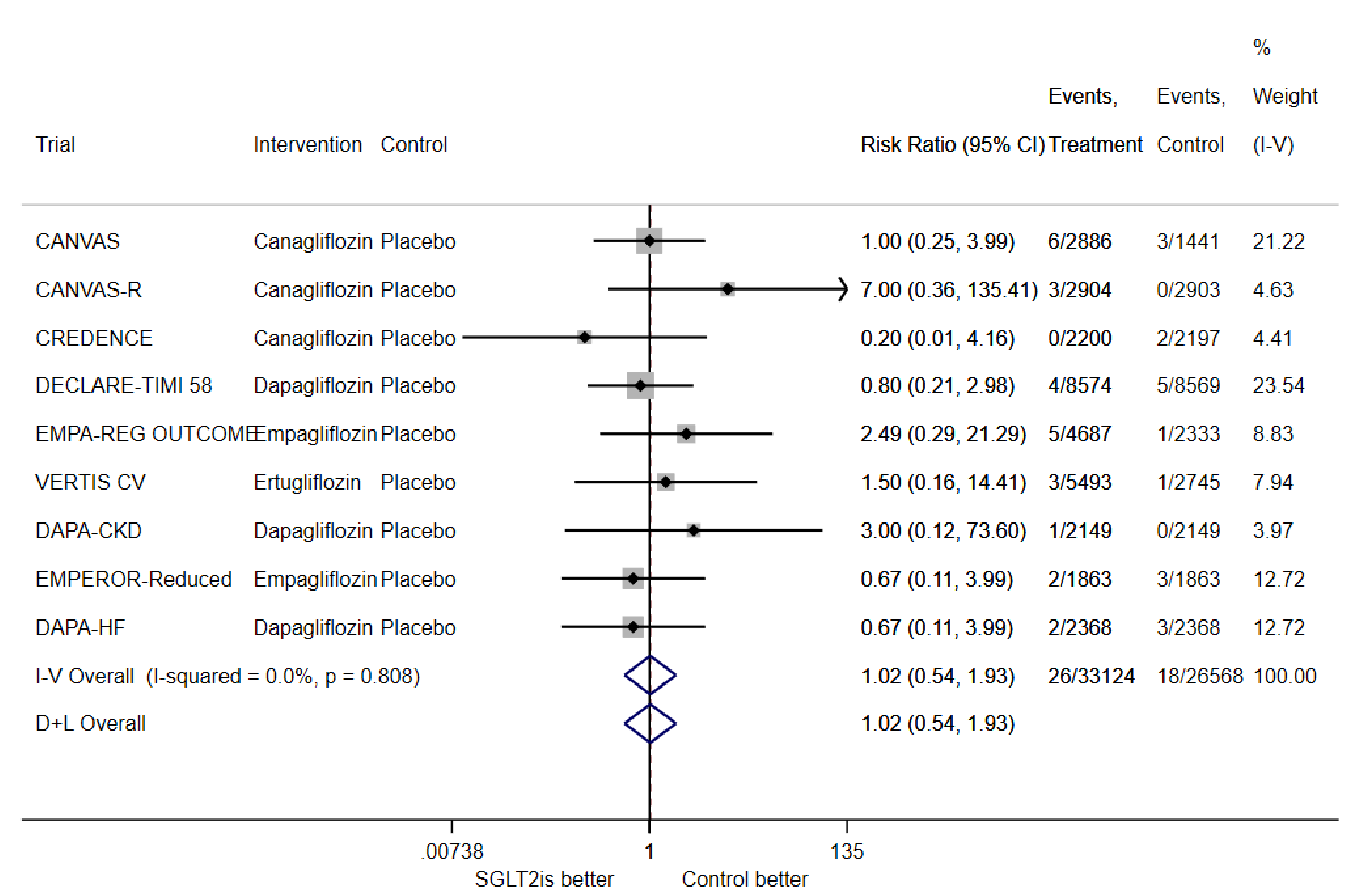

Figure S100 Meta-analysis of SGLT2is and Epistaxis

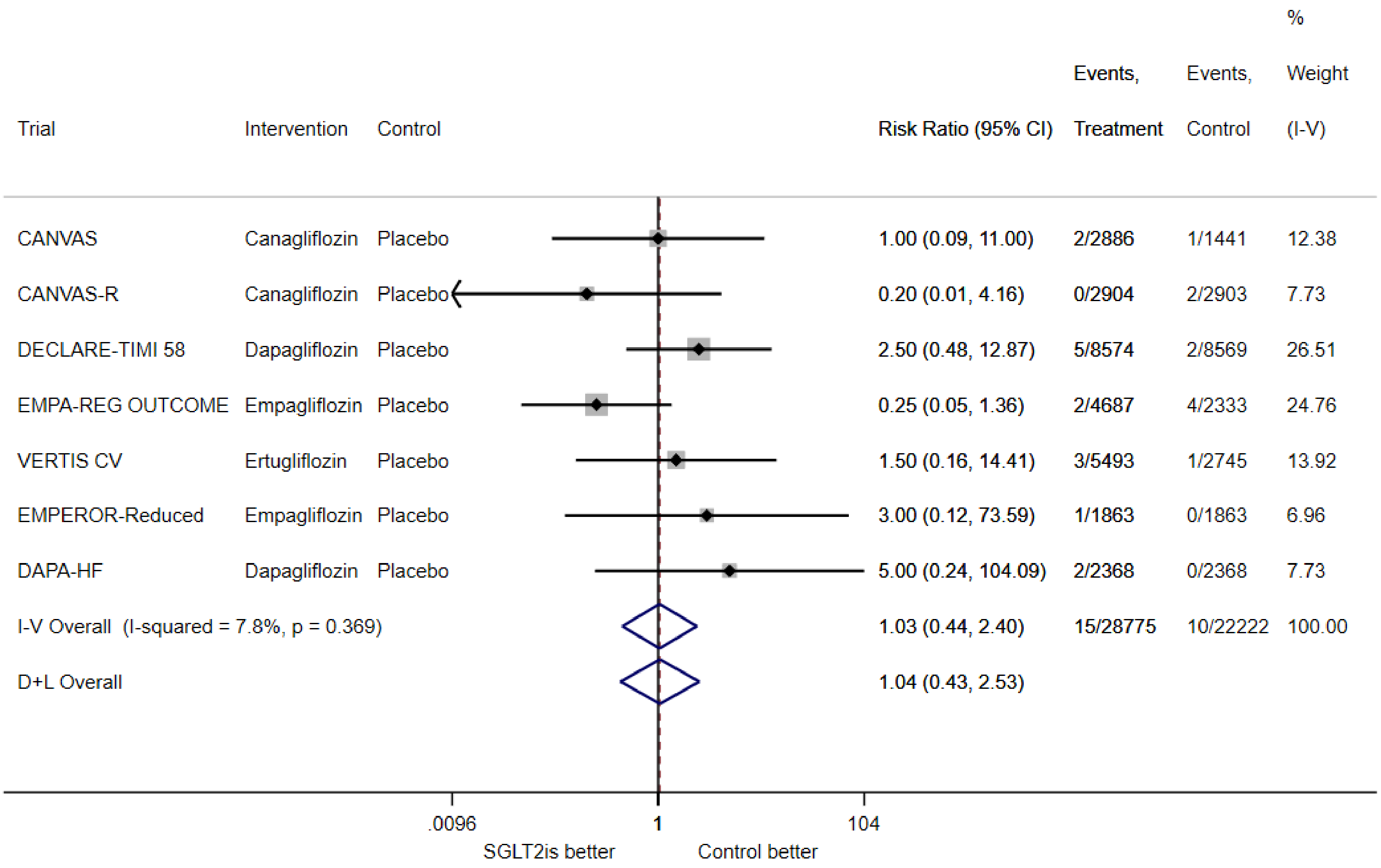

Figure S101 Meta-analysis of SGLT2is and Haemoptysis

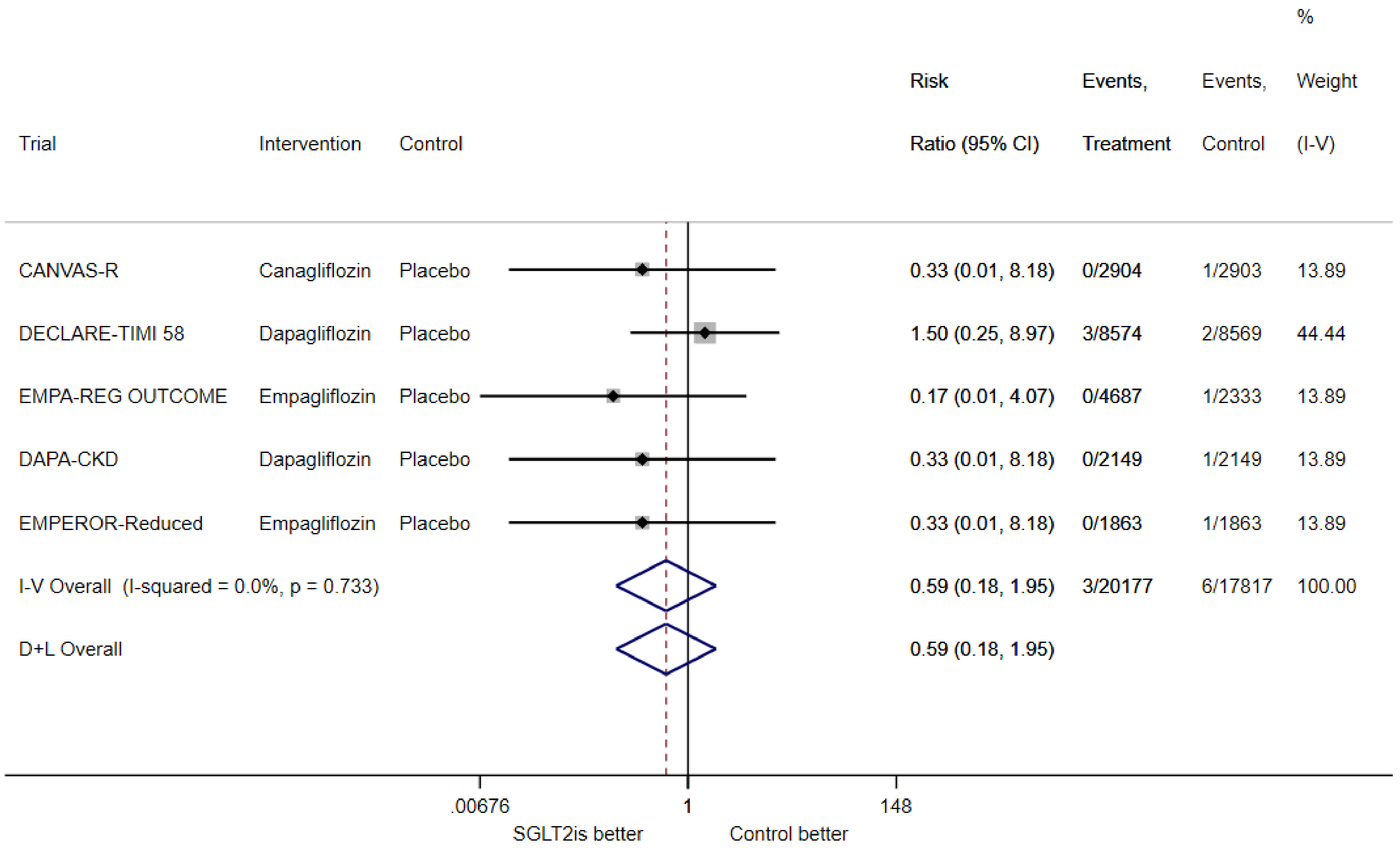

Figure S102 Meta-analysis of SGLT2is and Haemothorax

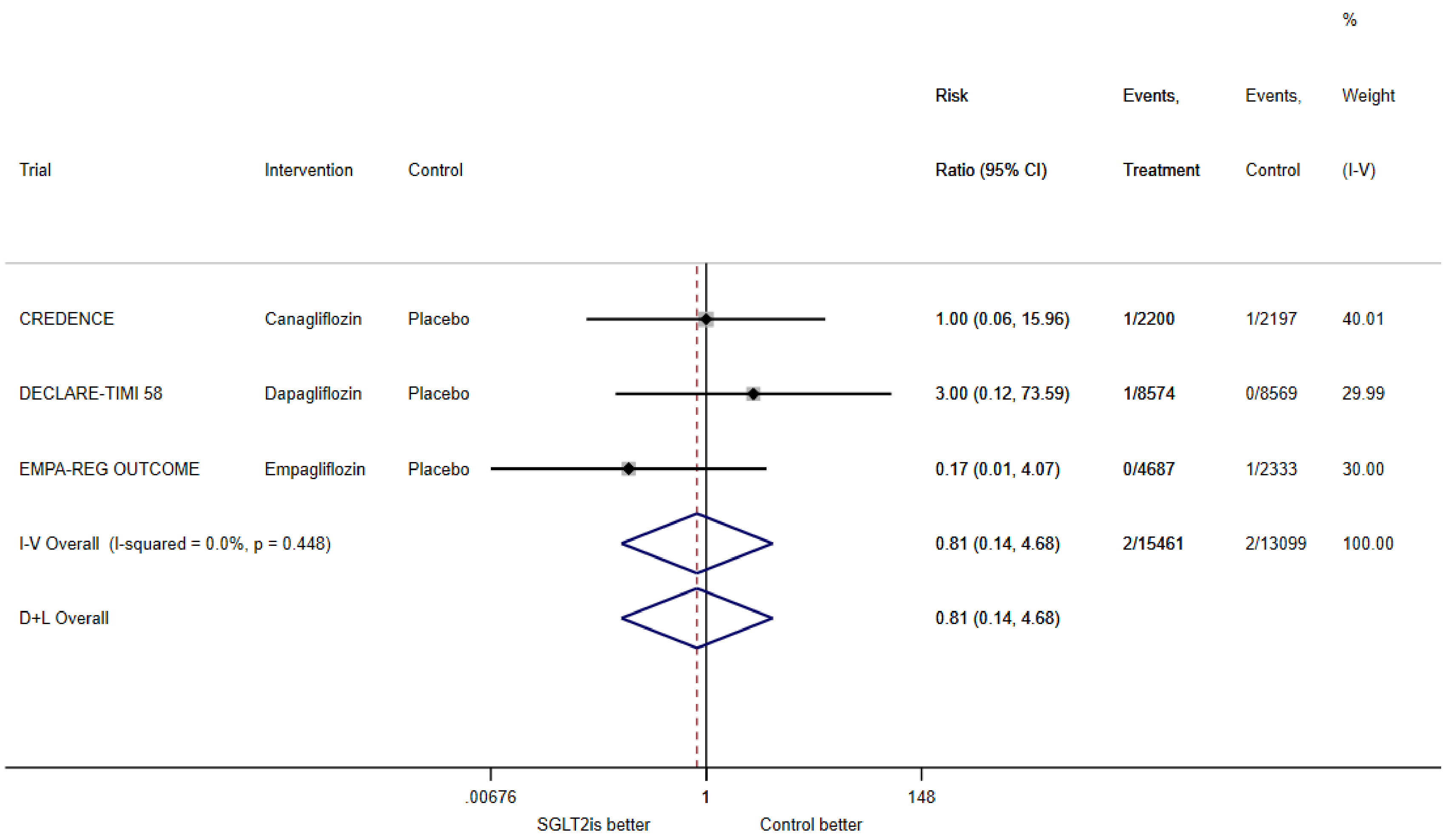

Figure S103 Meta-analysis of SGLT2is and Hydrothorax

Trial	Intervention	Control	Risk Ratio (95% CI)	Events, Treatment	Events, Control	% Weight (I-V)
CANVAS	Canagliflozin	Placebo	1.00 (0.09, 11.00)	2/2886	1/1441	13.80
CREDENCE	Canagliflozin	Placebo	3.00 (0.12, 73.50)	1/2200	0/2197	7.76
DECLARE-TIMI 58	Dapagliflozin	Placebo	1.00 (0.20, 4.95)	3/8574	3/8569	31.03
EMPA-REG OUTCOME	Empagliflozin	Placebo	1.49 (0.16, 14.35)	3/4687	1/2333	15.52
VERTIS CV	Ertugliflozin	Placebo	0.25 (0.02, 2.75)	1/5493	2/2745	13.79
DAPA-CKD	Dapagliflozin	Placebo	0.33 (0.01, 8.18)	0/2149	1/2149	7.76
DAPA-HF	Dapagliflozin	Placebo	1.00 (0.06, 15.98)	1/2368	1/2368	10.34
I-V Overall (I-squared = 0.0%, p = 0.898)			0.88 (0.36, 2.14)	11/28357	9/21802	100.00
D+L Overall			0.88 (0.36, 2.14)			

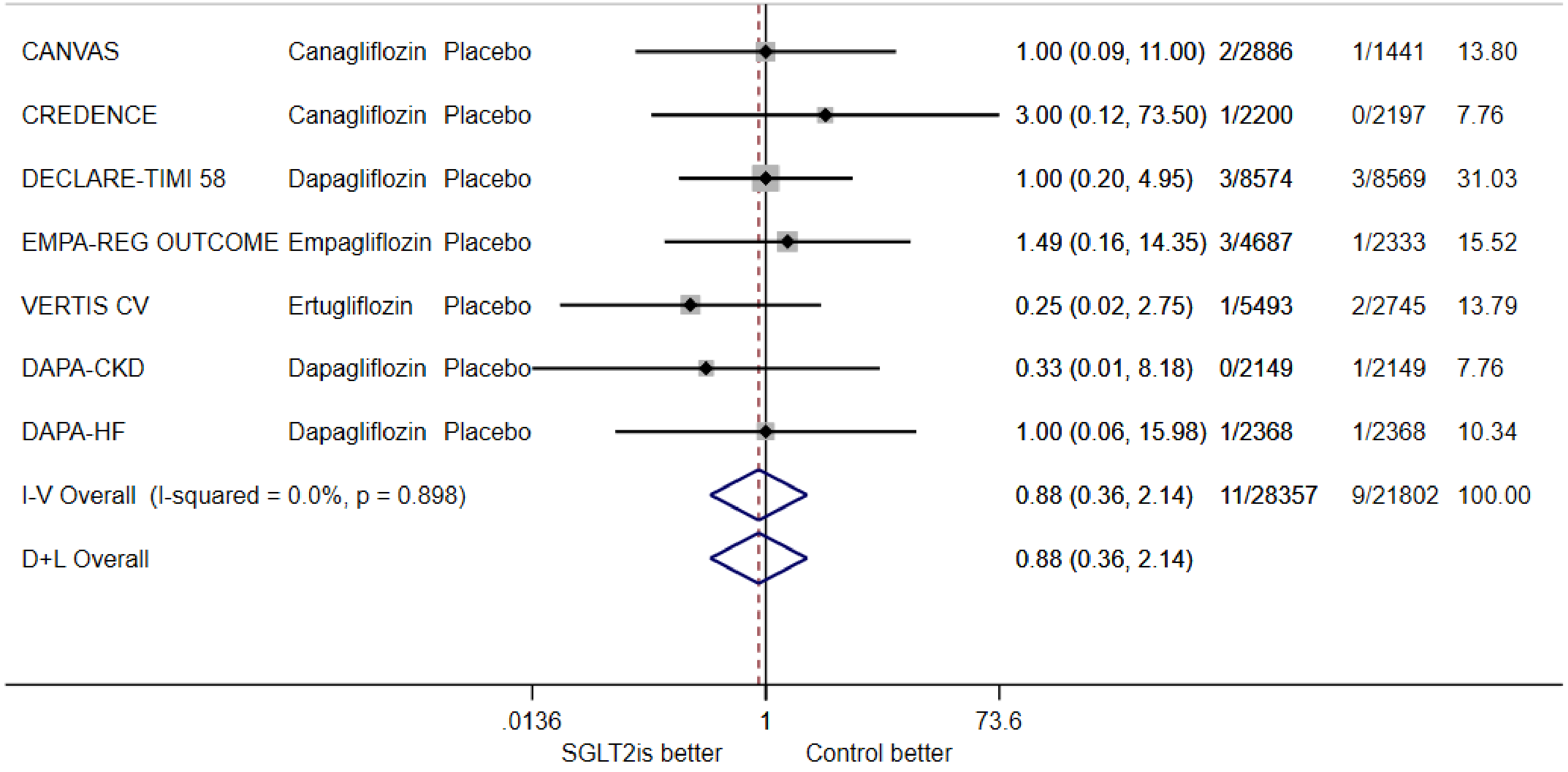

Figure S104 Meta-analysis of SGLT2is and Hypoxia

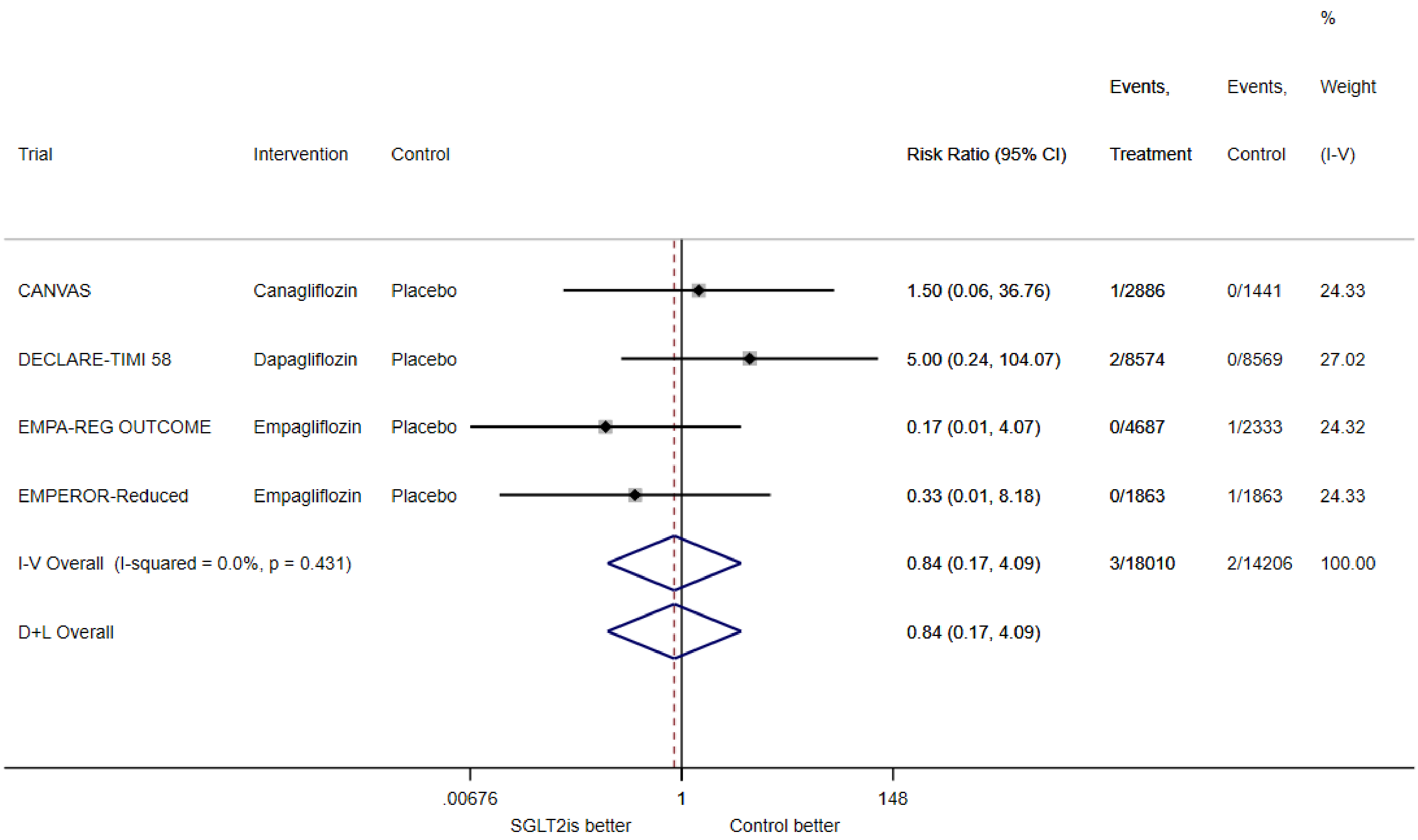

Figure S105 Meta-analysis of SGLT2is and Idiopathic pulmonary fibrosis

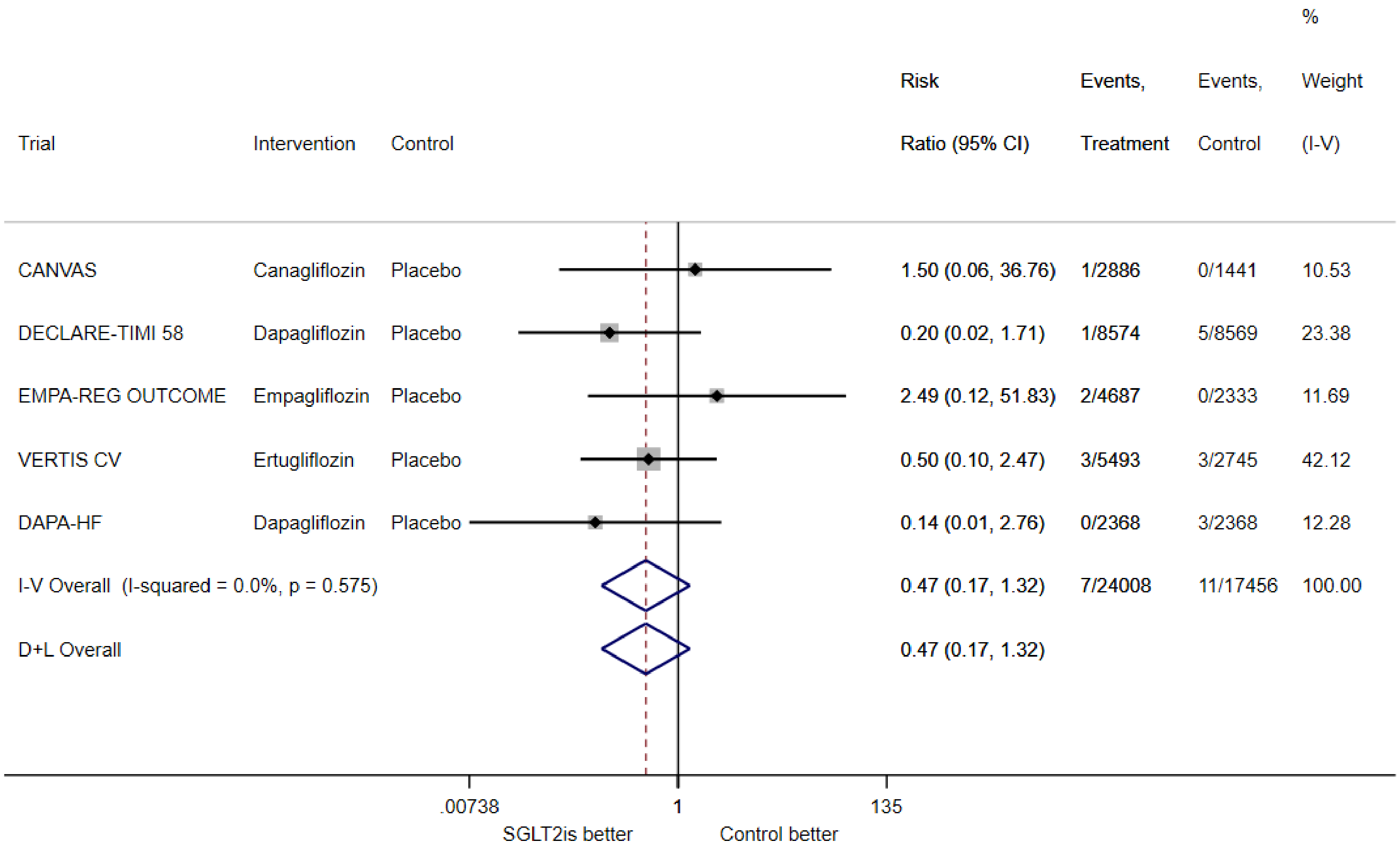

Figure S106 Meta-analysis of SGLT2is and Interstitial lung disease

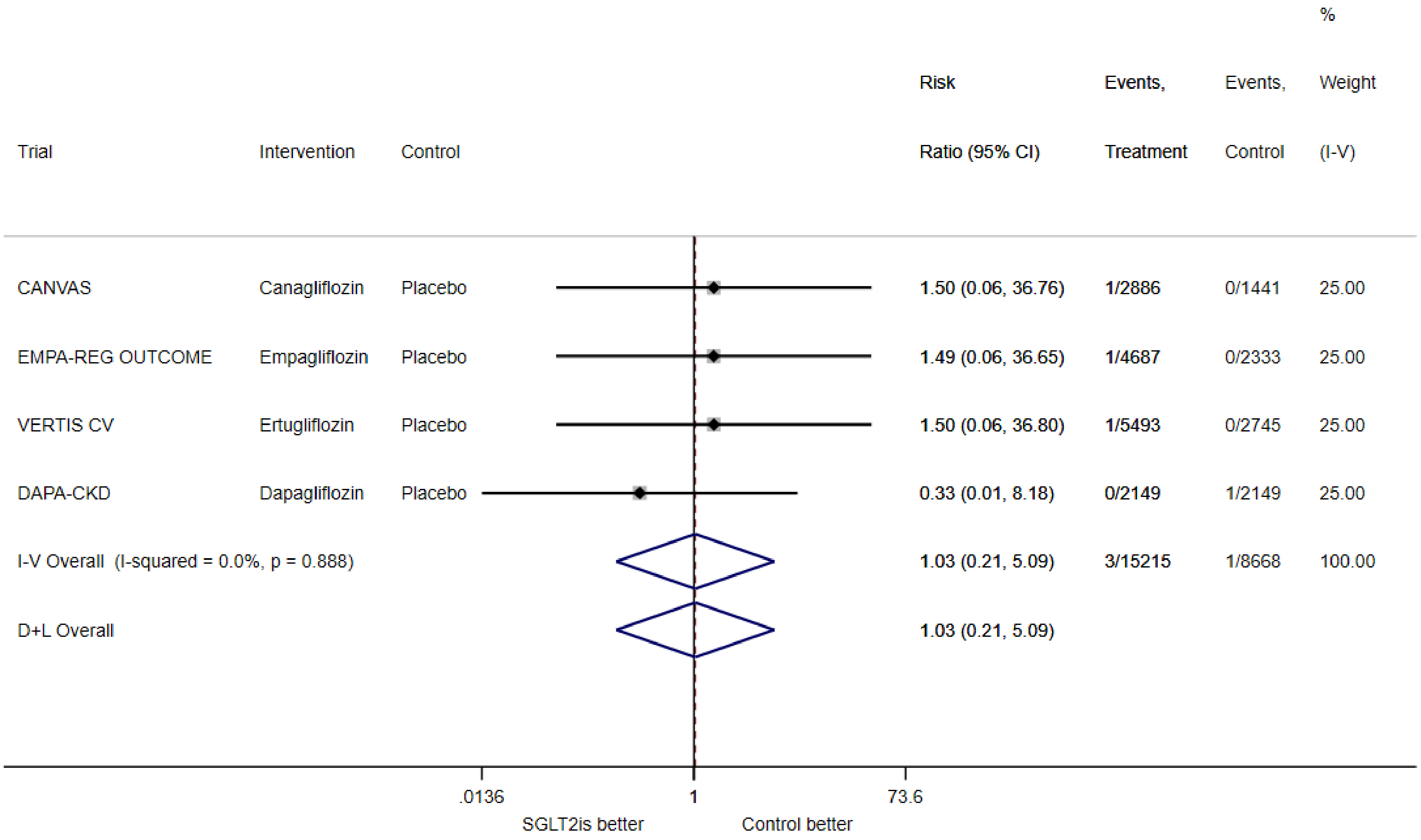

Figure S107 Meta-analysis of SGLT2is and Laryngeal oedema

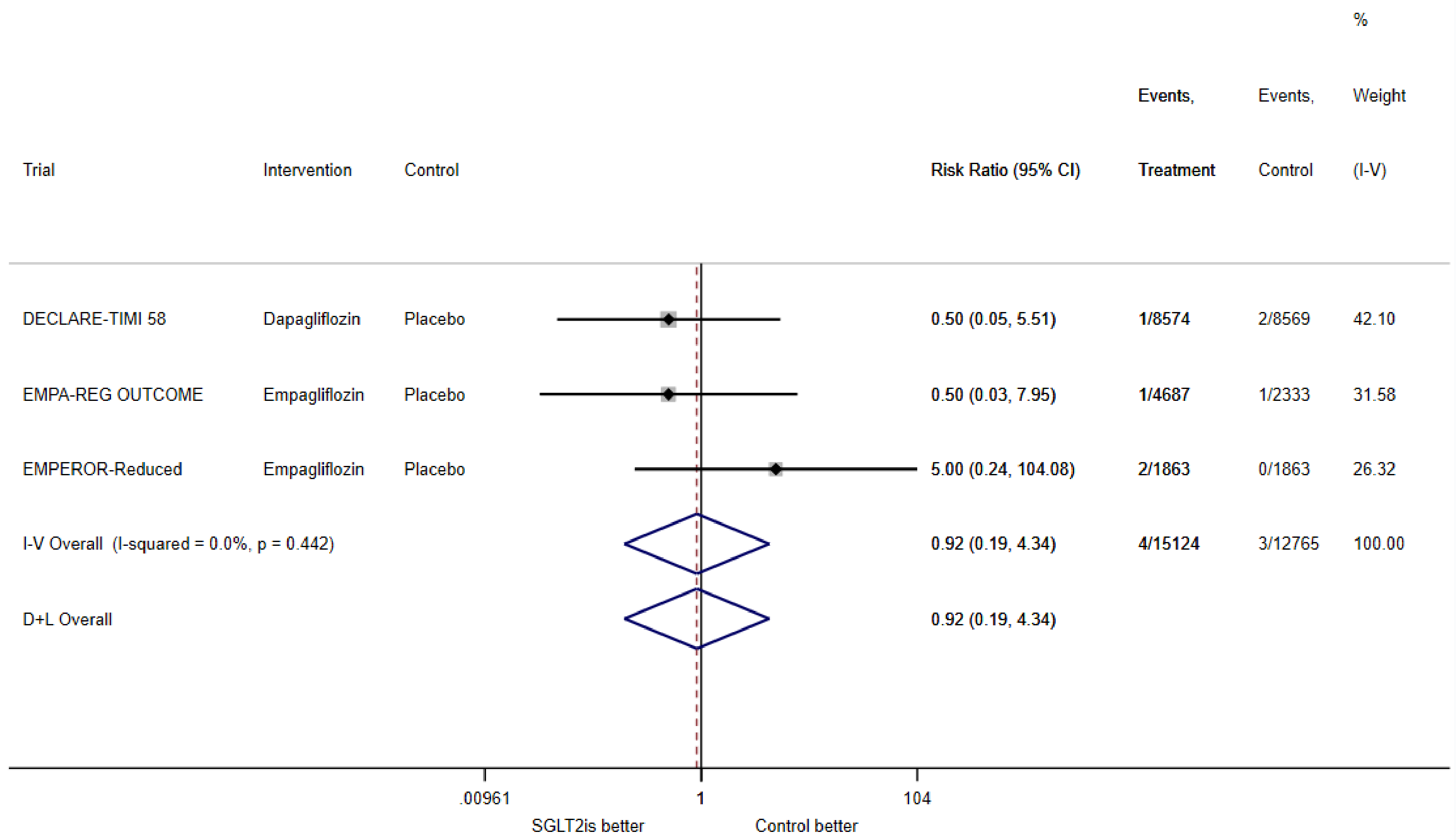

Figure S108 Meta-analysis of SGLT2is and Lung disorder

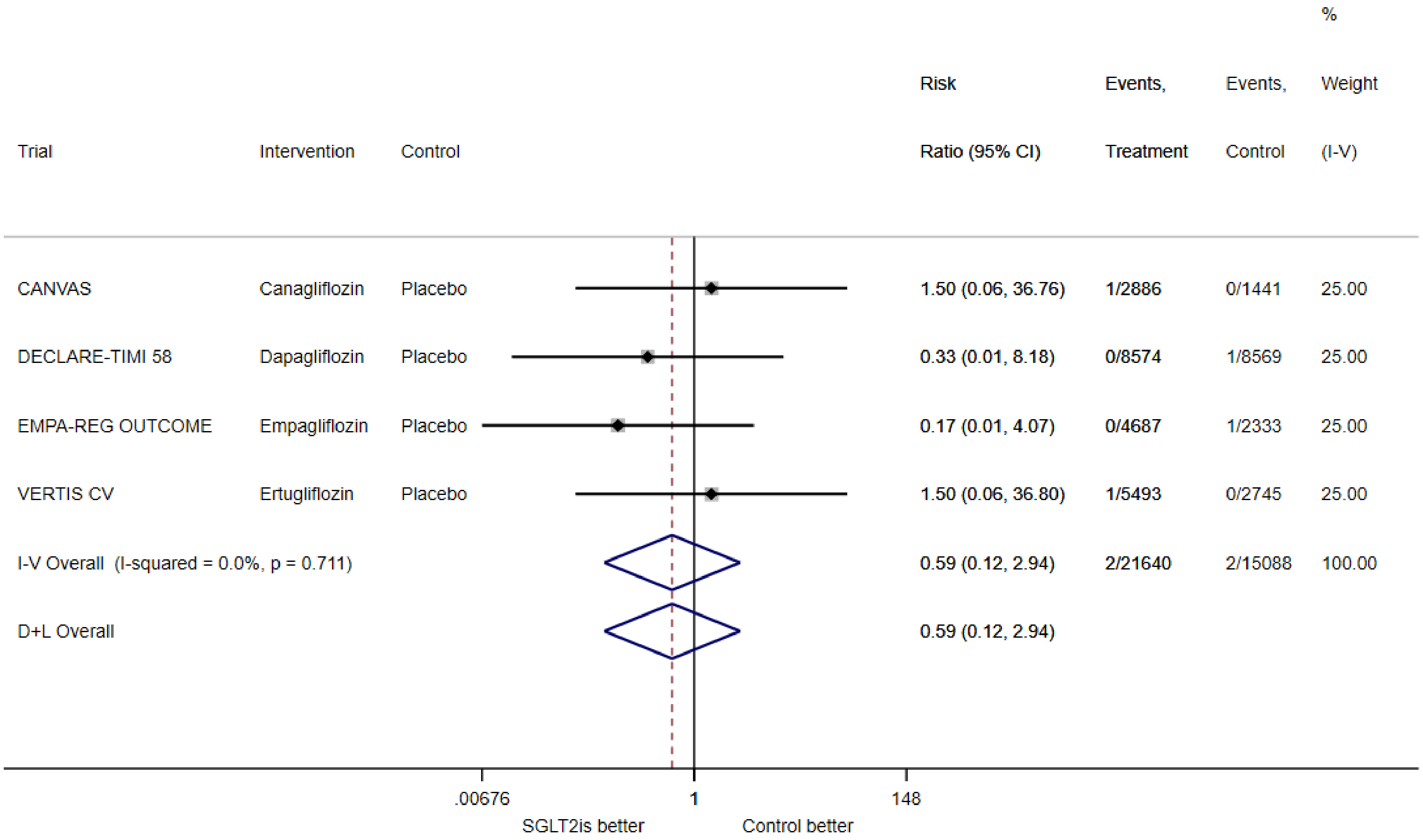

Figure S109 Meta-analysis of SGLT2is and Lung infiltration

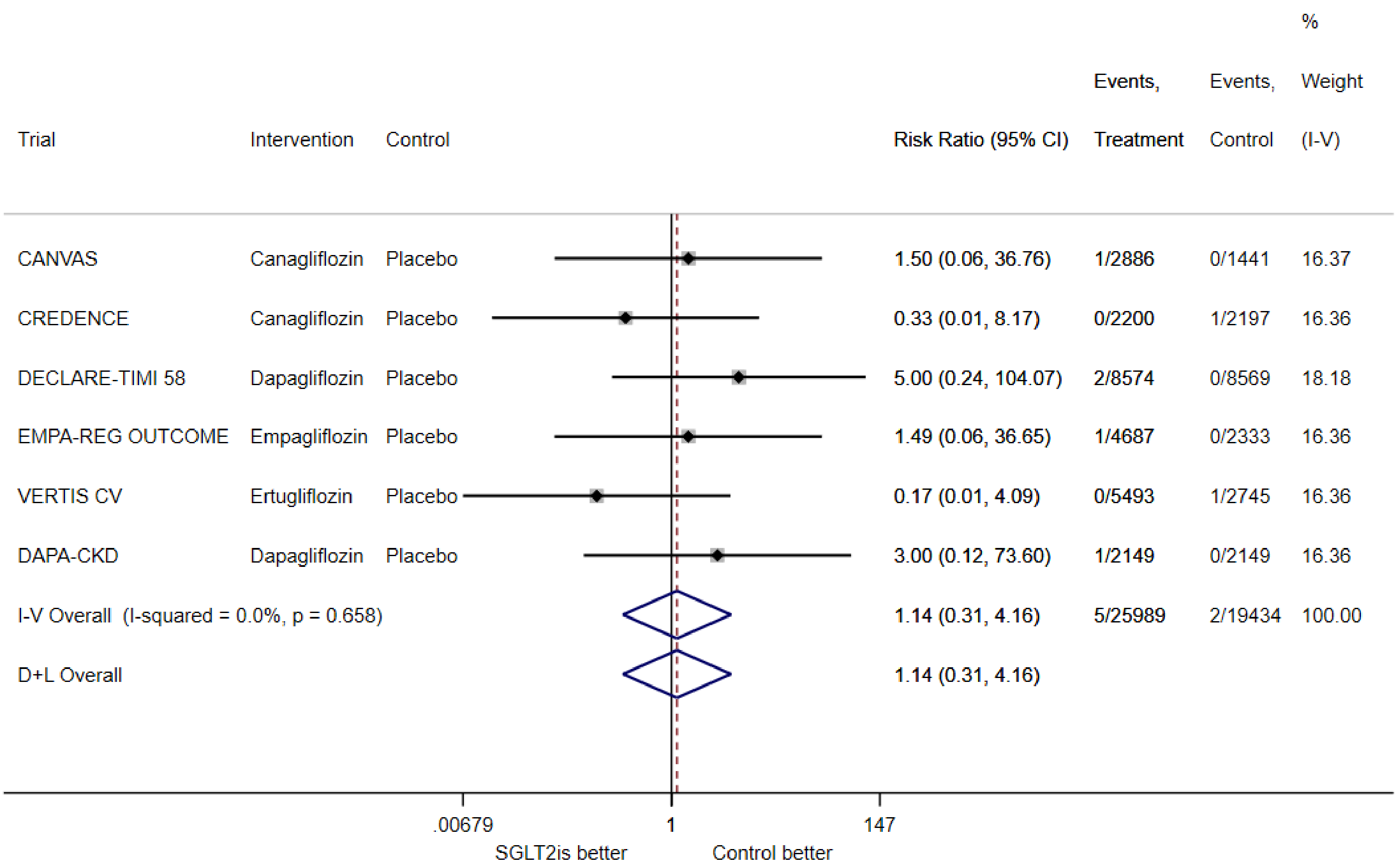

Figure S110 Meta-analysis of SGLT2is and Nasal polyps

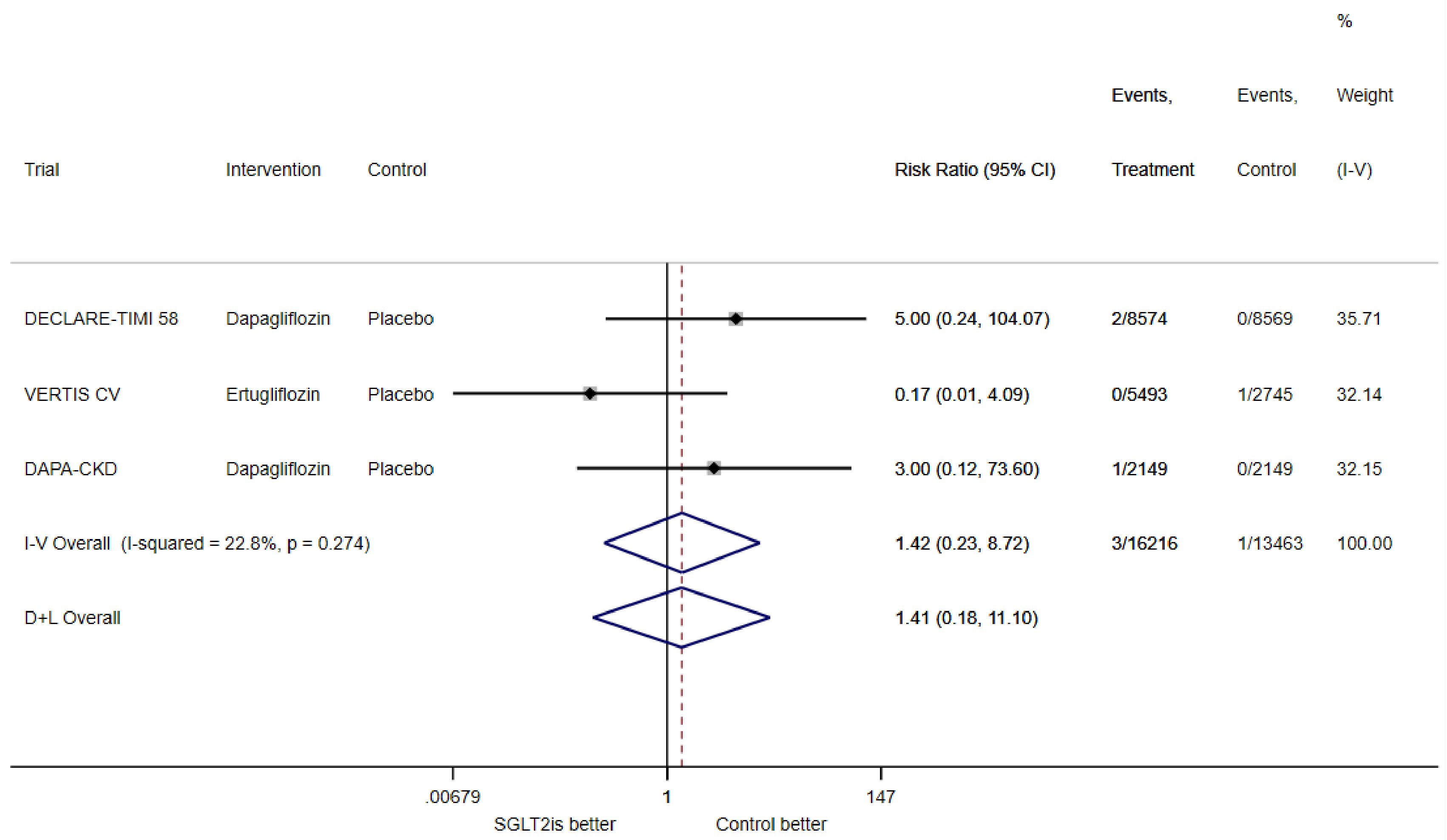

Figure S111 Meta-analysis of SGLT2is and Nasal septum deviation

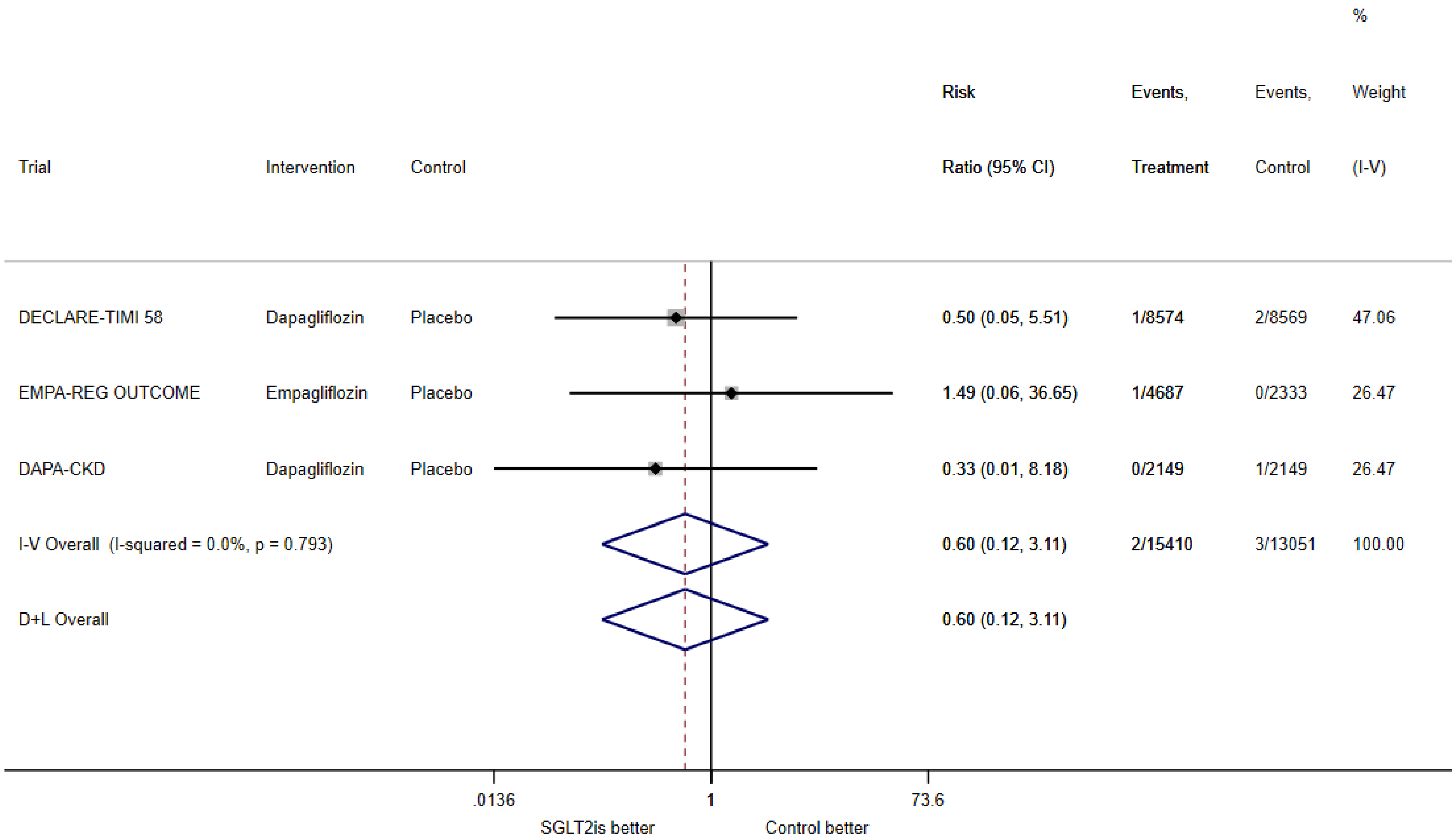

Figure S112 Meta-analysis of SGLT2is and Obstructive airways disorder

Trial	Intervention	Control	Risk Ratio (95% CI)	Events, Treatment	Events, Control	% Weight (I-V)
EMPA-REG OUTCOME	Empagliflozin	Placebo	1.49 (0.06, 36.65)	1/4687	0/2333	33.33
VERTIS CV	Ertugliflozin	Placebo	1.50 (0.06, 36.80)	1/5493	0/2745	33.33
EMPEROR-Reduced	Empagliflozin	Placebo	0.33 (0.01, 8.18)	0/1863	1/1863	33.34
I-V Overall (I-squared = 0.0%, p = 0.754)			0.91 (0.14, 5.76)	2/12043	1/6941	100.00
D+L Overall			0.91 (0.14, 5.76)			

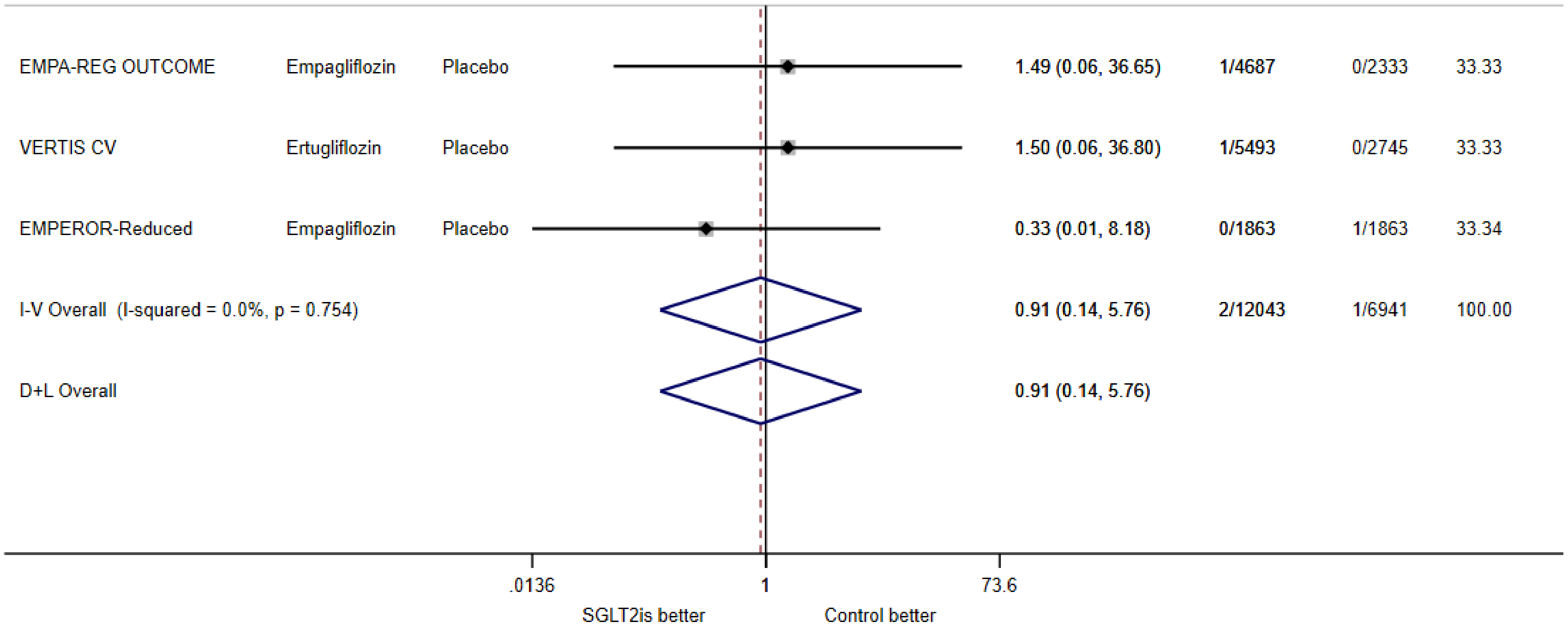

Figure S113 Meta-analysis of SGLT2is and Paranasal cyst

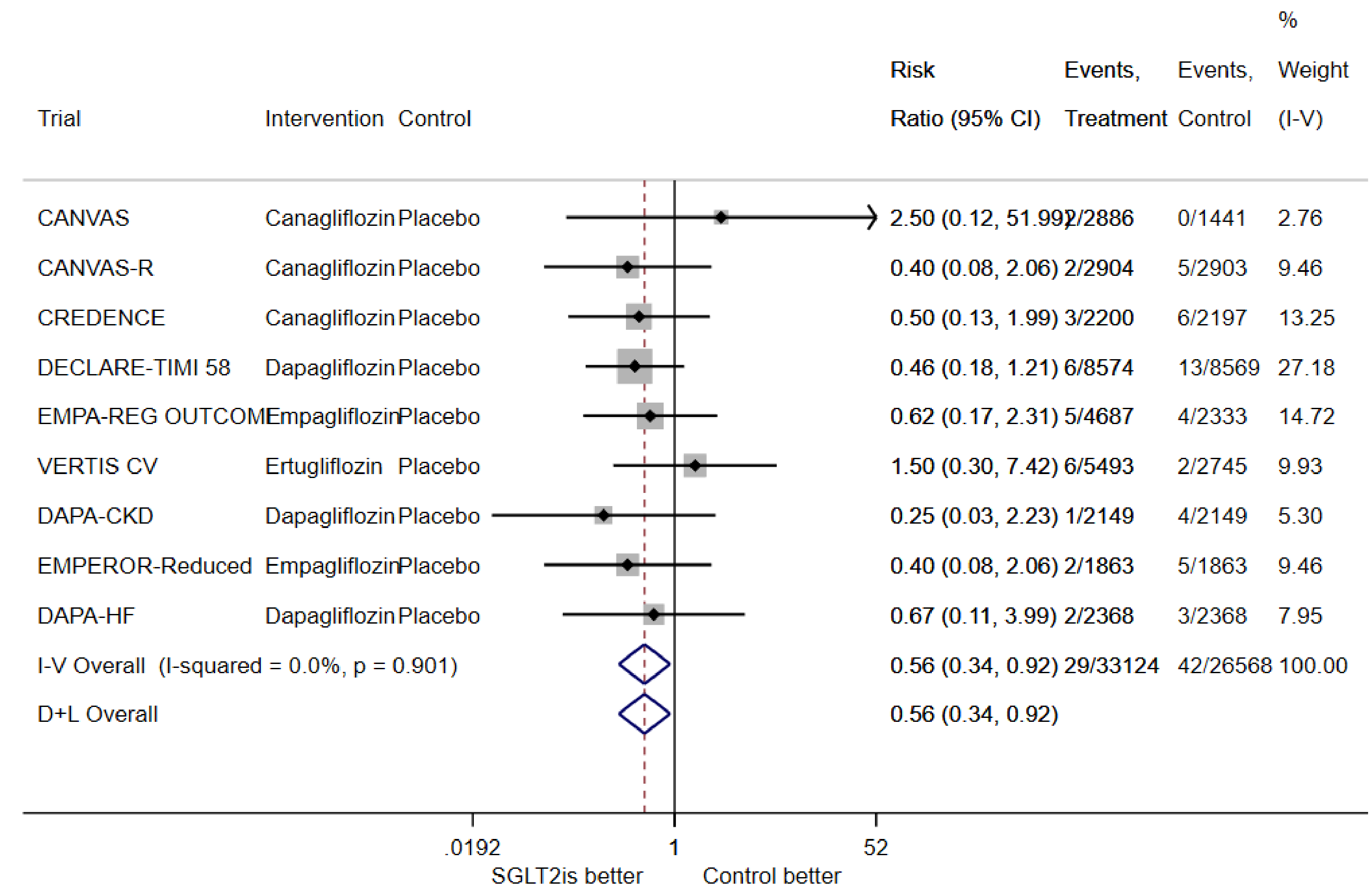

Figure S114 Meta-analysis of SGLT2is and Pleural effusion

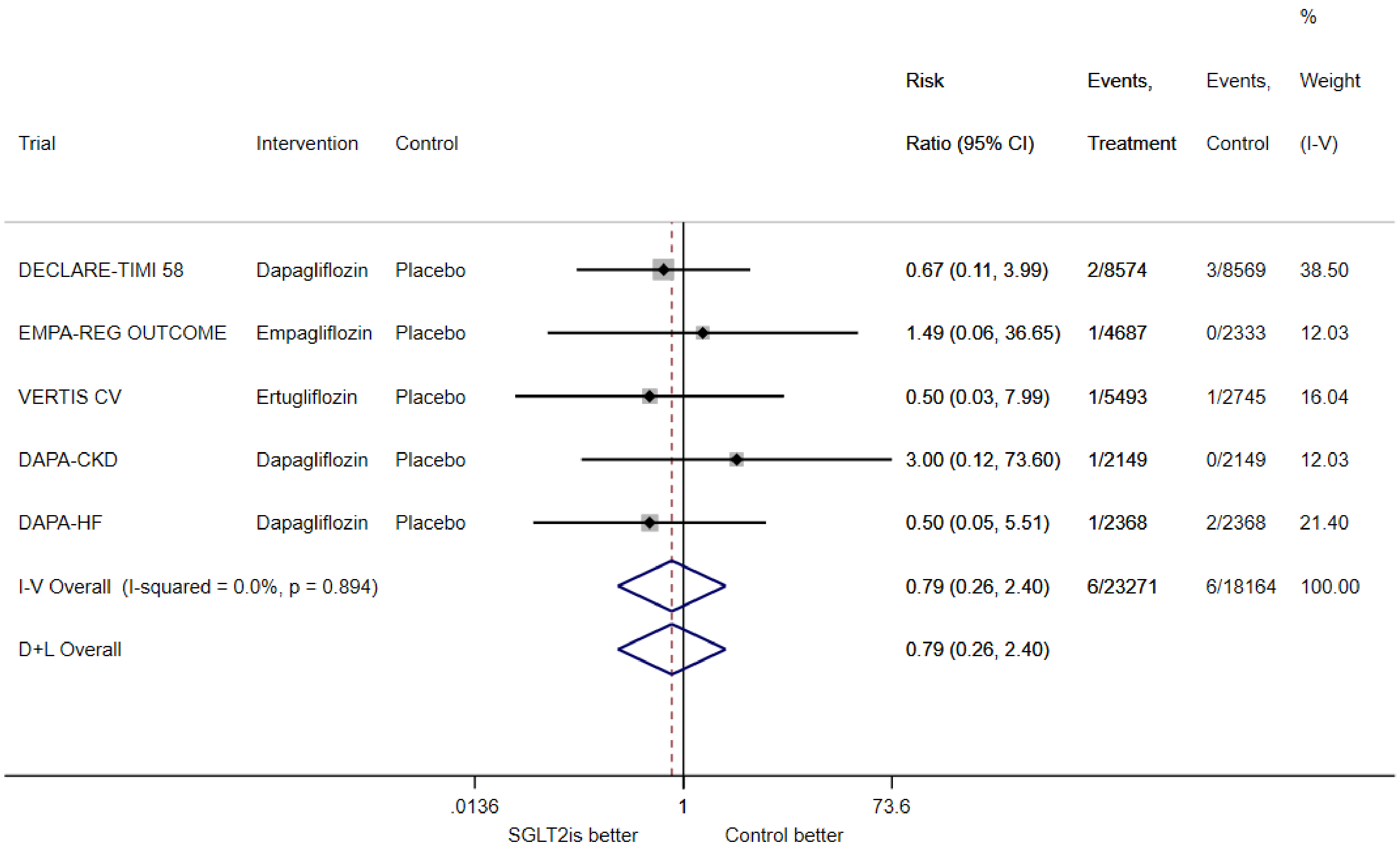

Figure S115 Meta-analysis of SGLT2is and Pleurisy

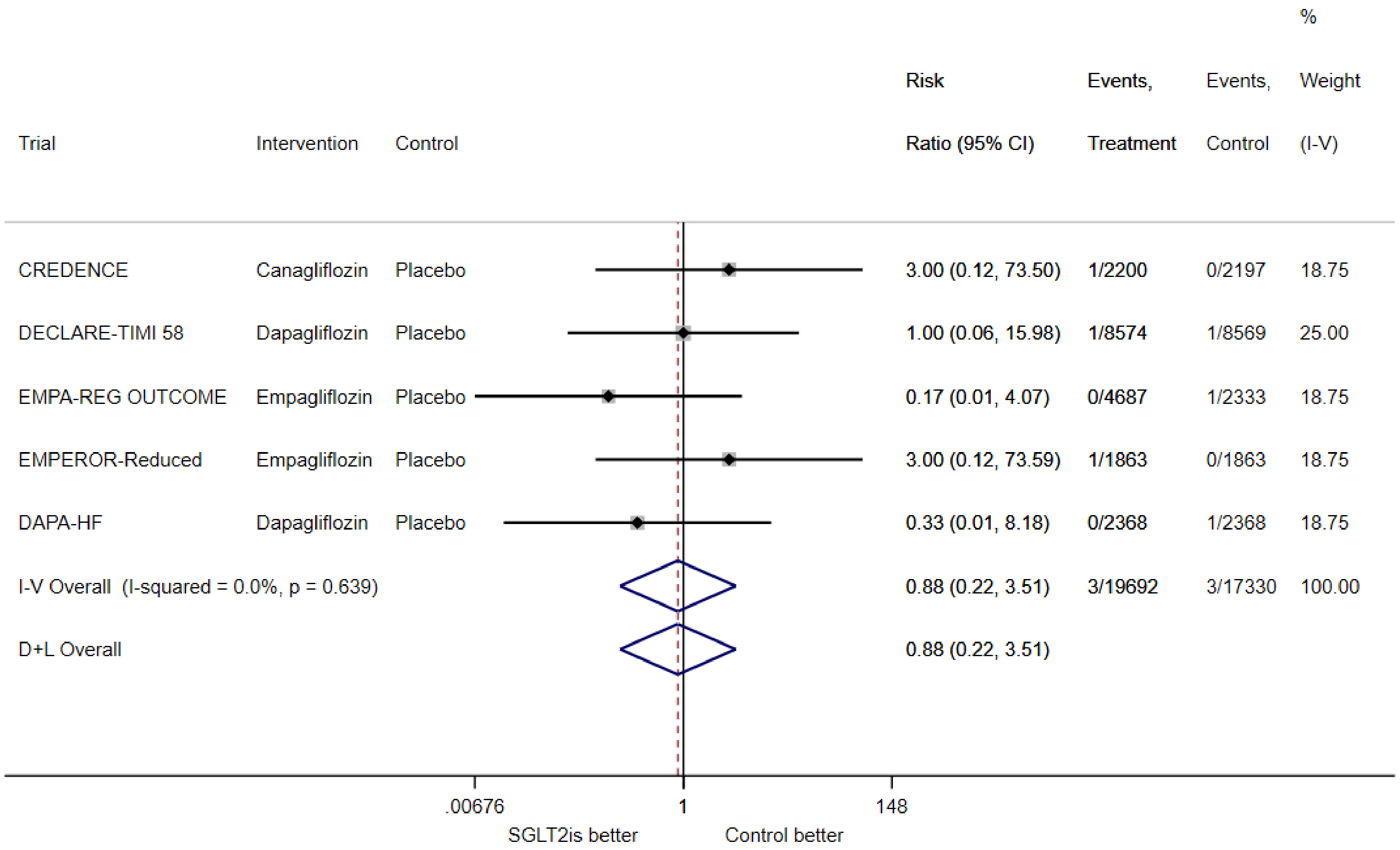

Figure S116 Meta-analysis of SGLT2is and Pleuritic pain

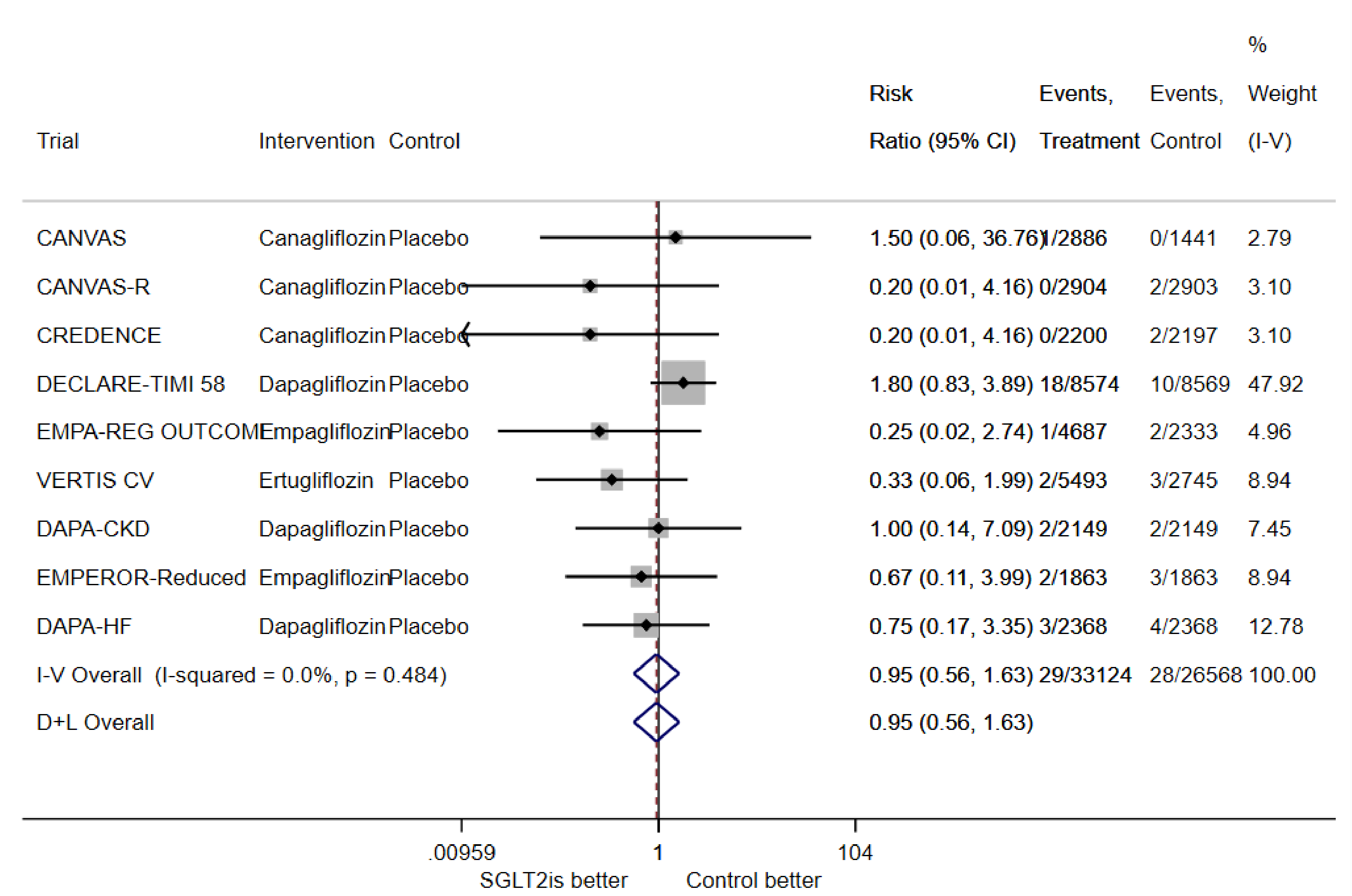

Figure S117 Meta-analysis of SGLT2is and Pneumonia aspiration

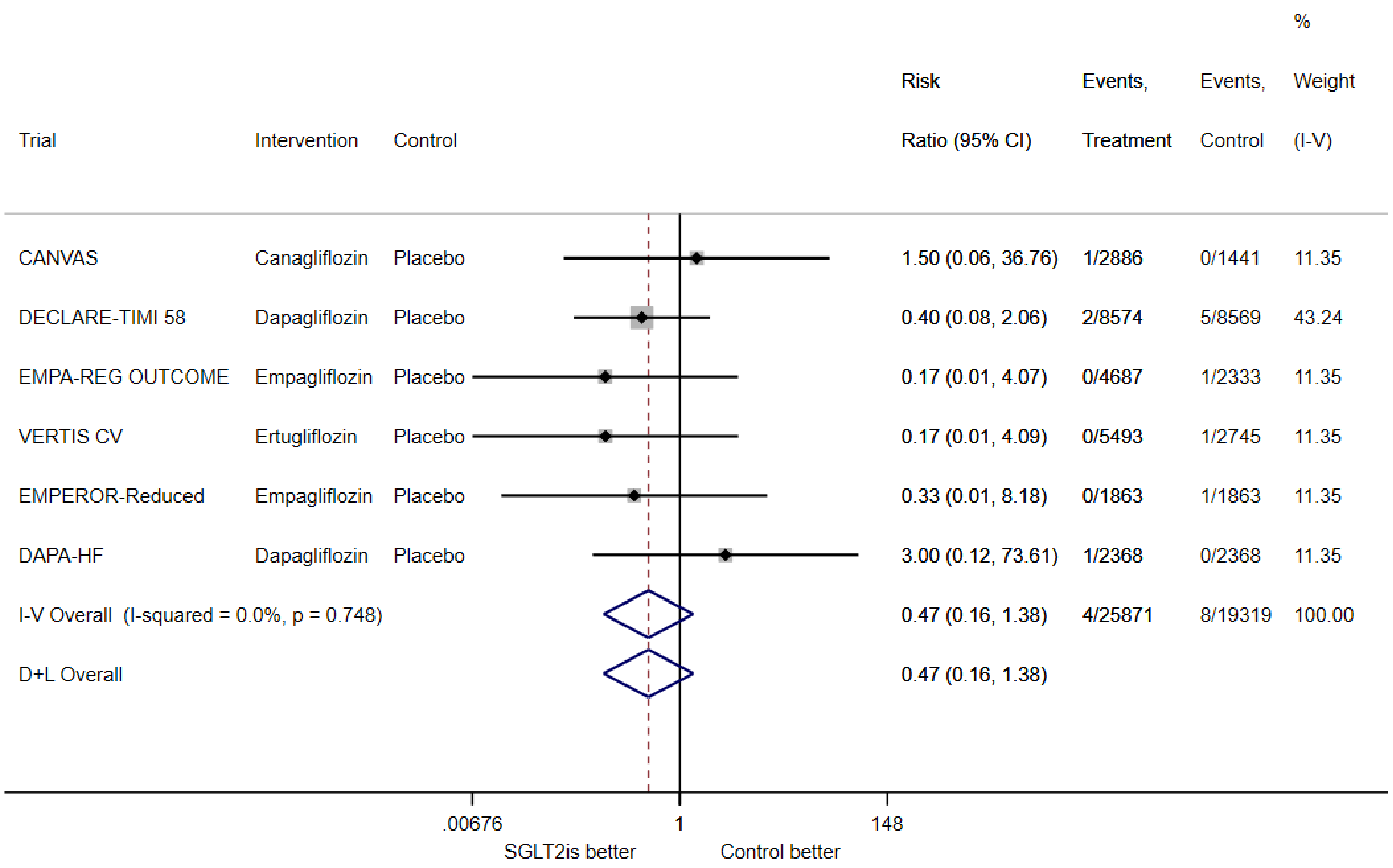

Figure S118 Meta-analysis of SGLT2is and Pneumonitis

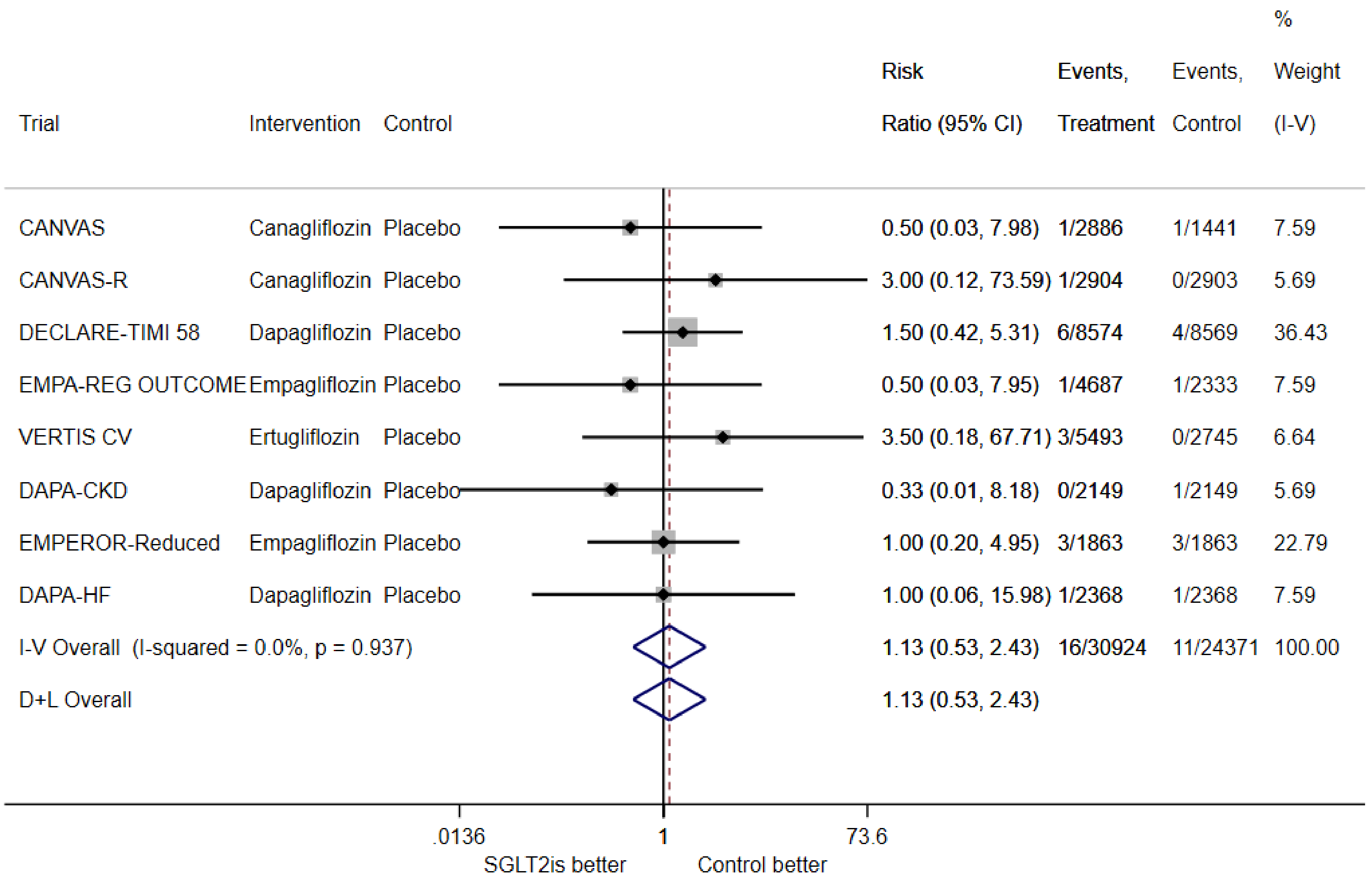

Figure S119 Meta-analysis of SGLT2is and Pneumothorax

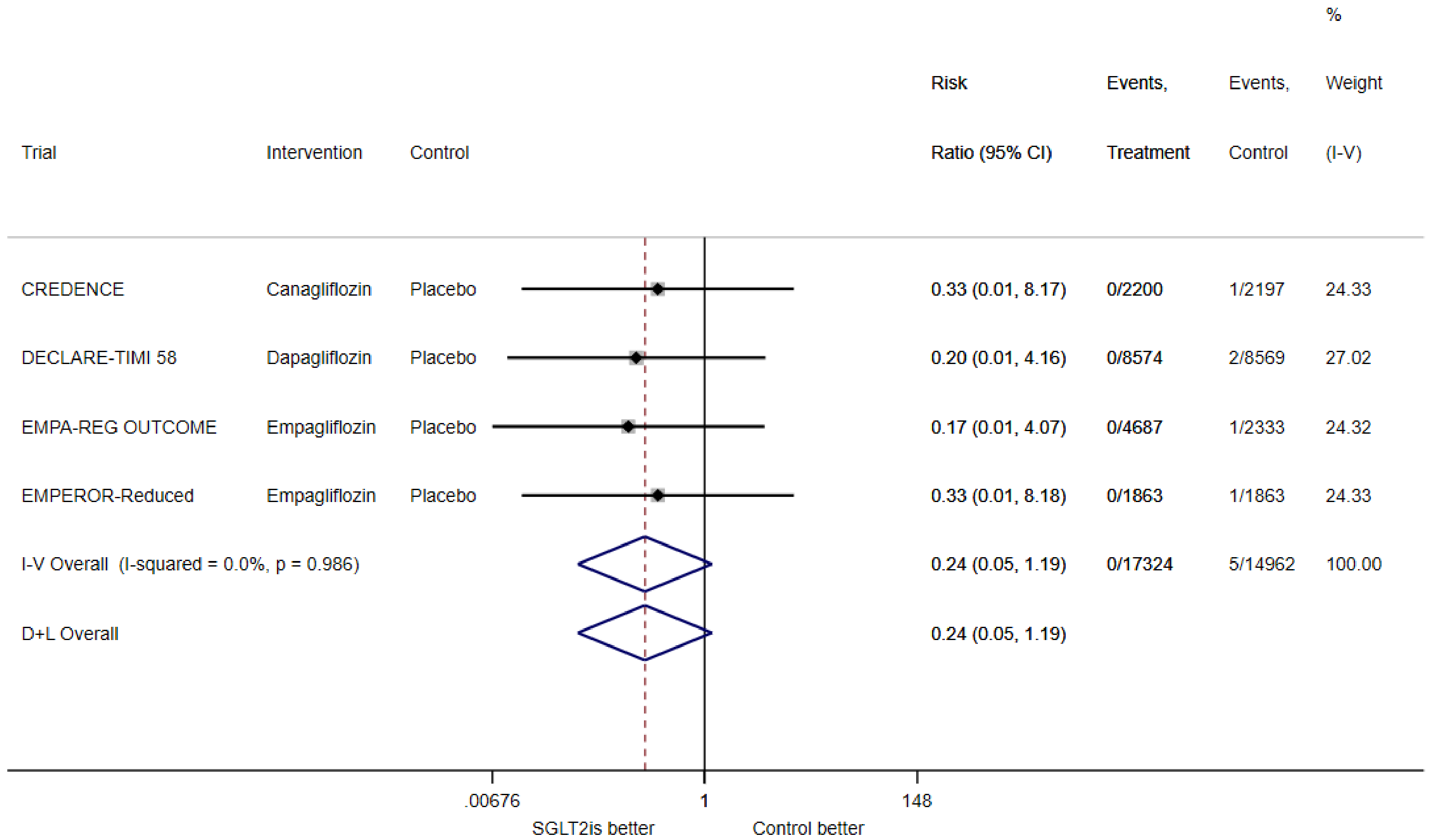

Figure S120 Meta-analysis of SGLT2is and Pulmonary arterial hypertension

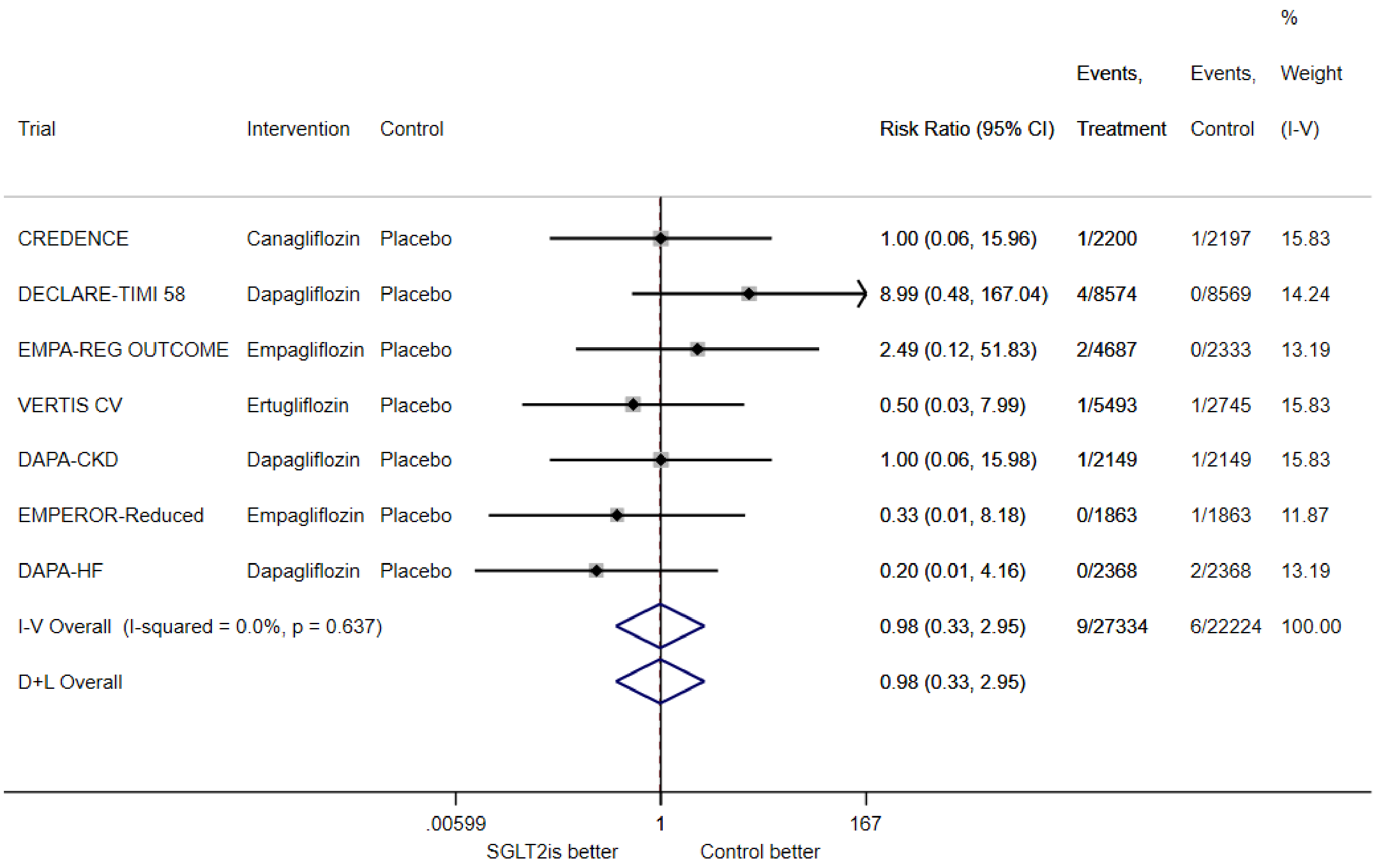

Figure S121 Meta-analysis of SGLT2is and Pulmonary congestion

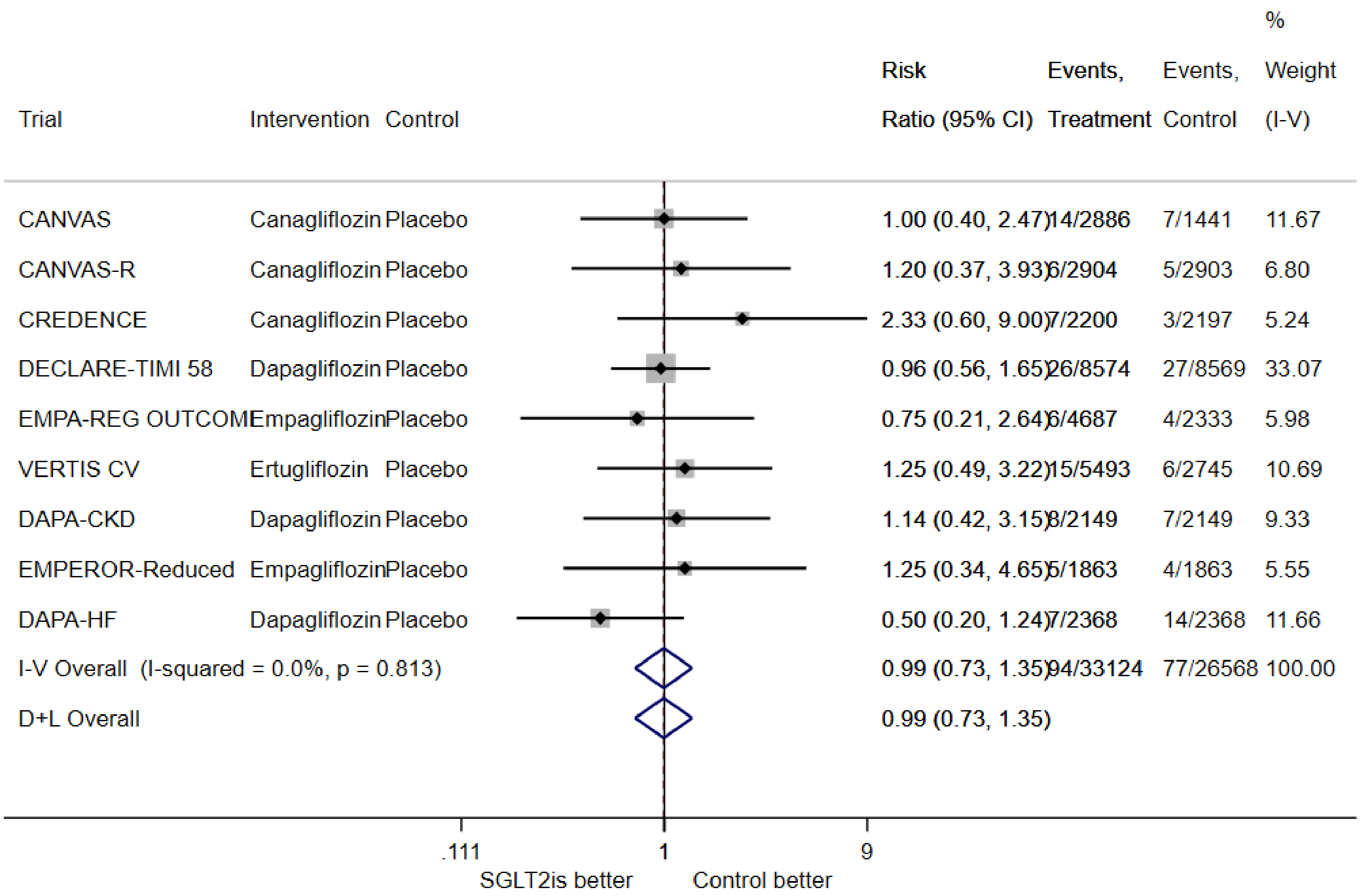

Figure S122 Meta-analysis of SGLT2is and Pulmonary embolism

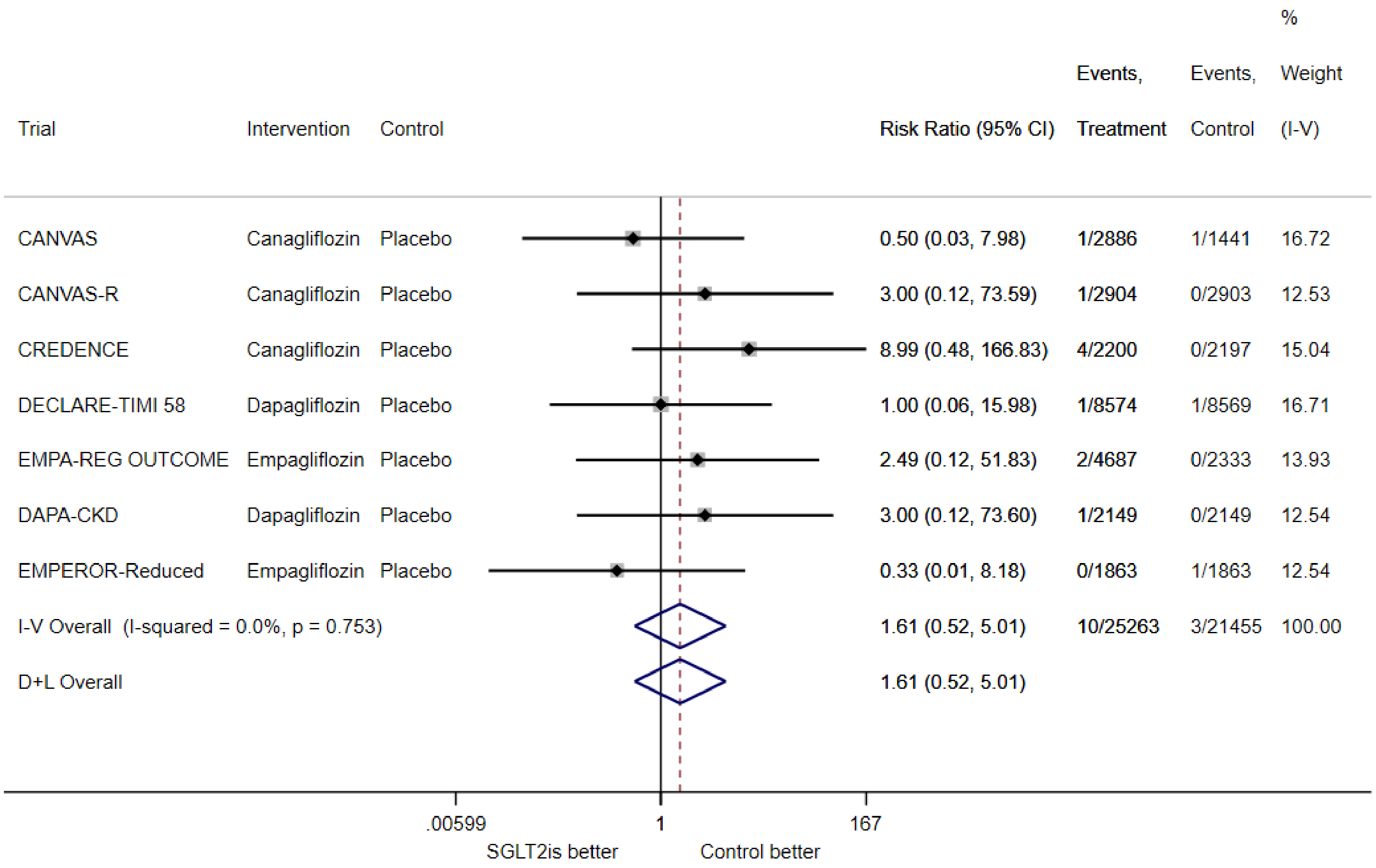

Figure S123 Meta-analysis of SGLT2is and Pulmonary fibrosis

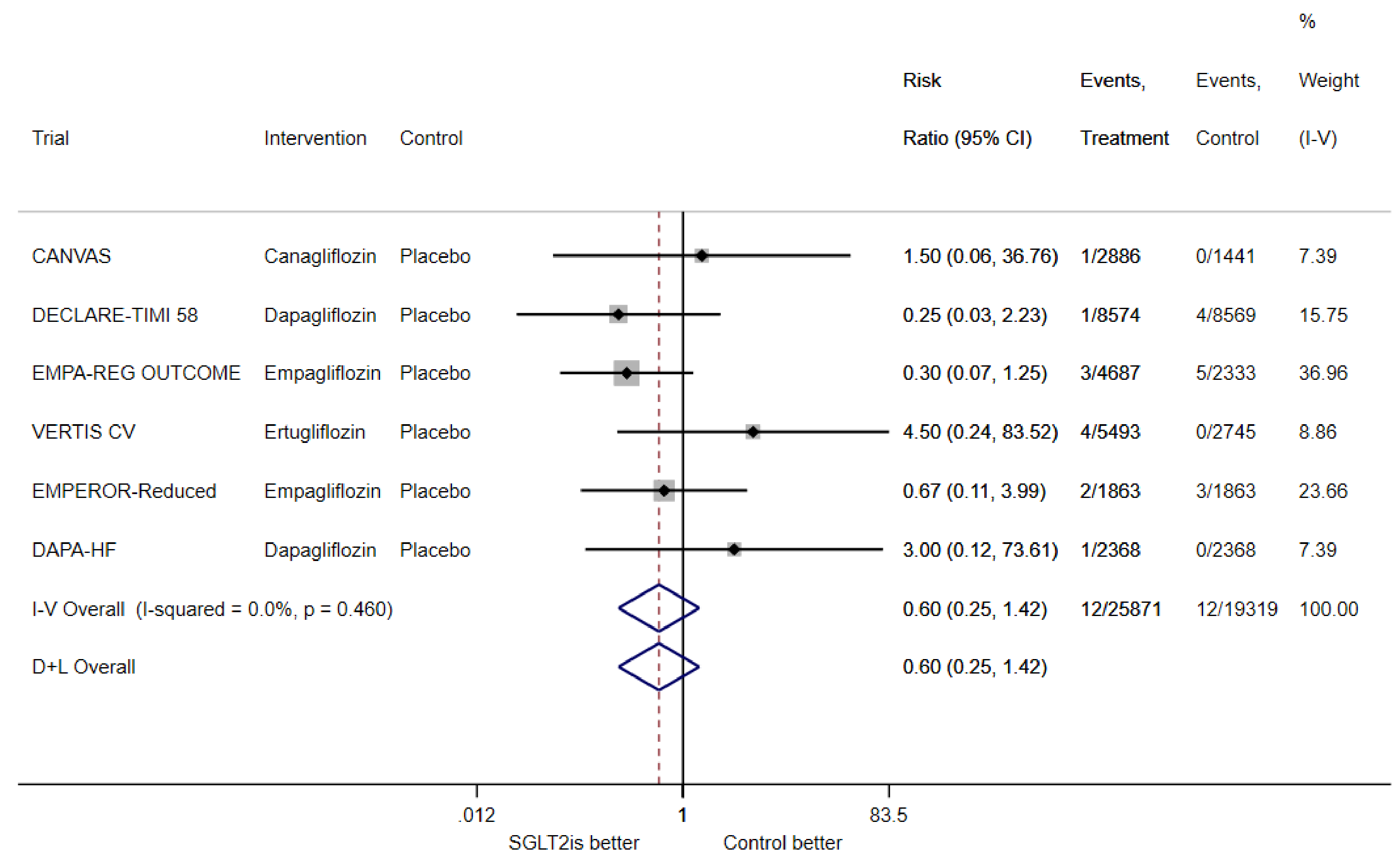

Figure S124 Meta-analysis of SGLT2is and Pulmonary hypertension

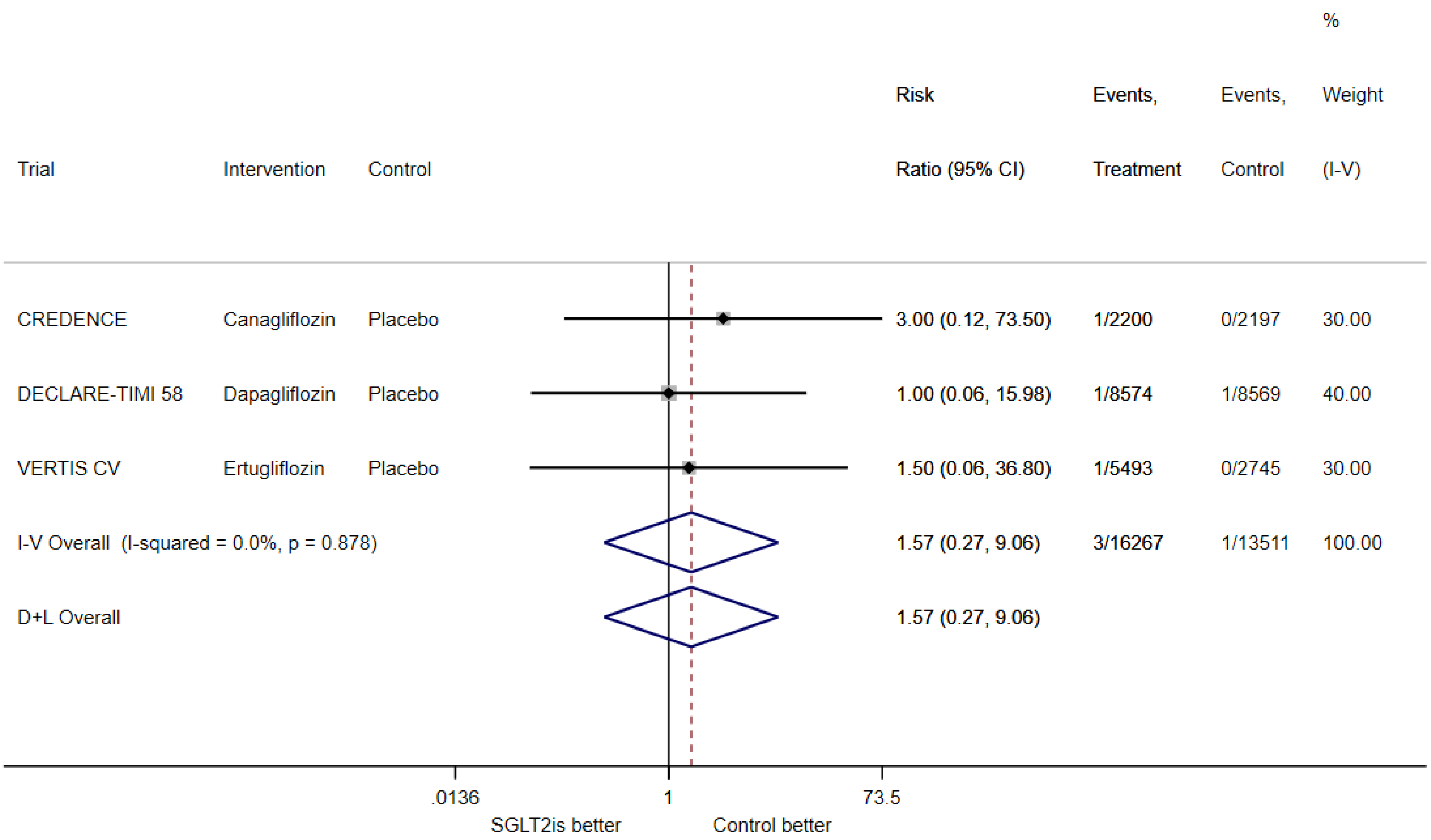

Figure S125 Meta-analysis of SGLT2is and Pulmonary infarction

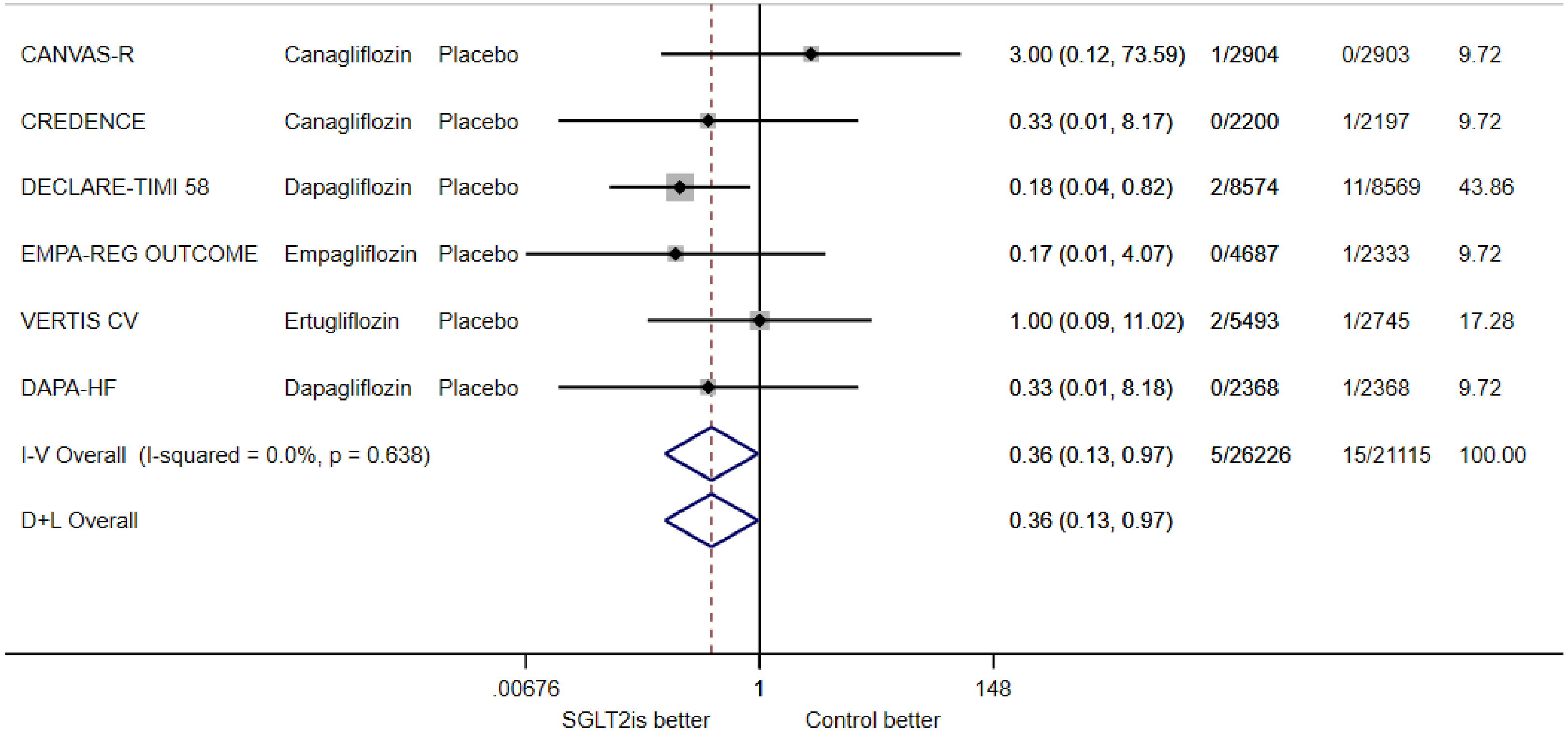

Trial	Intervention	Control	Risk Ratio (95% CI)	Events, Treatment	Events, Control	% Weight (I-V)
CANVAS-R	Canagliflozin	Placebo	3.00 (0.12, 73.59)	1/2904	0/2903	9.72
CREDENCE	Canagliflozin	Placebo	0.33 (0.01, 8.17)	0/2200	1/2197	9.72
DECLARE-TIMI 58	Dapagliflozin	Placebo	0.18 (0.04, 0.82)	2/8574	11/8569	43.86
EMPA-REG OUTCOME	Empagliflozin	Placebo	0.17 (0.01, 4.07)	0/4687	1/2333	9.72
VERTIS CV	Ertugliflozin	Placebo	1.00 (0.09, 11.02)	2/5493	1/2745	17.28
DAPA-HF	Dapagliflozin	Placebo	0.33 (0.01, 8.18)	0/2368	1/2368	9.72
I-V Overall (I-squared = 0.0%, p = 0.638)			0.36 (0.13, 0.97)	5/26226	15/21115	100.00
D+L Overall			0.36 (0.13, 0.97)			

Figure S126 Meta-analysis of SGLT2is and Pulmonary mass

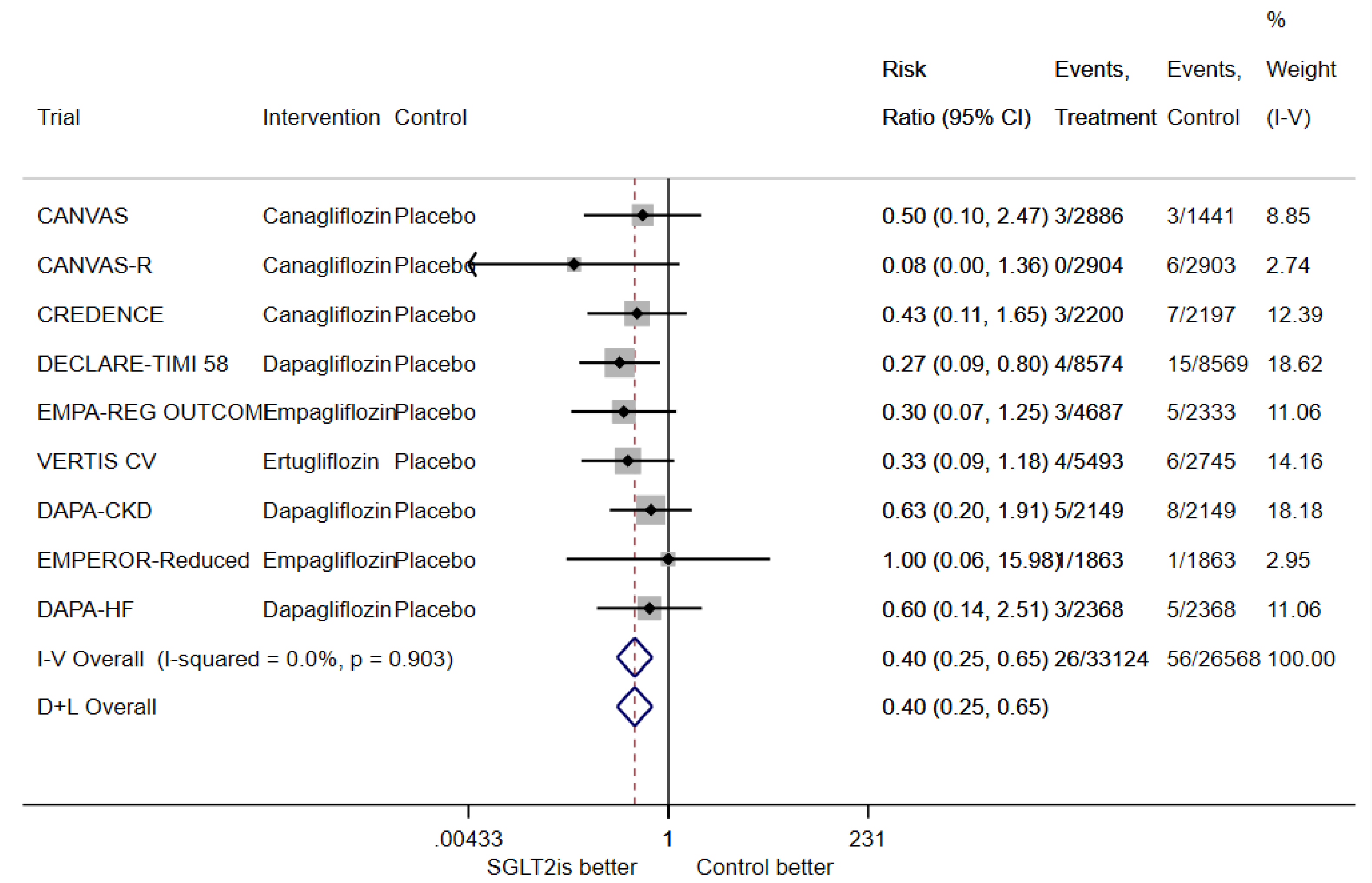

Figure S127 Meta-analysis of SGLT2is and Pulmonary oedema

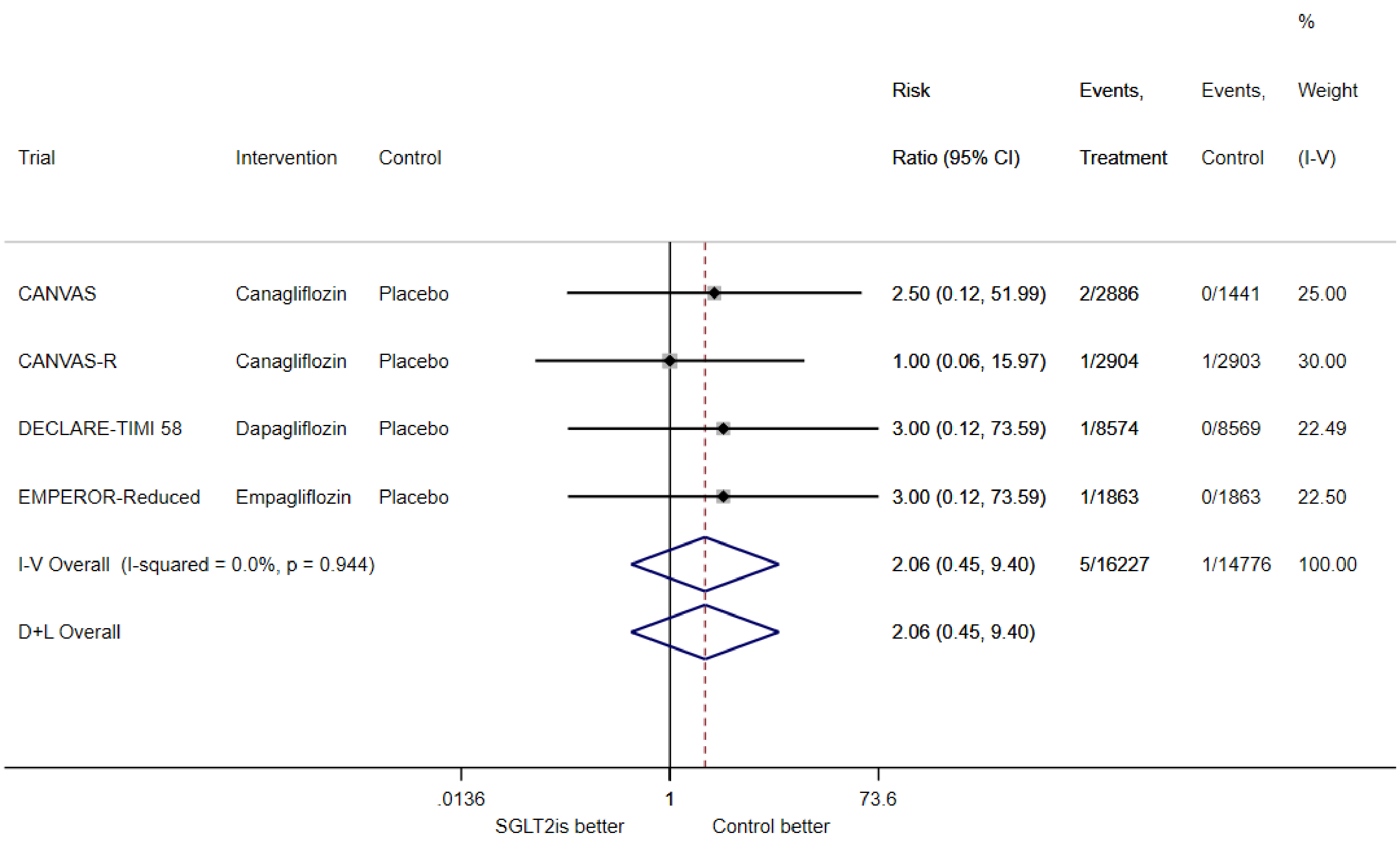

Figure S128 Meta-analysis of SGLT2is and Respiratory acidosis

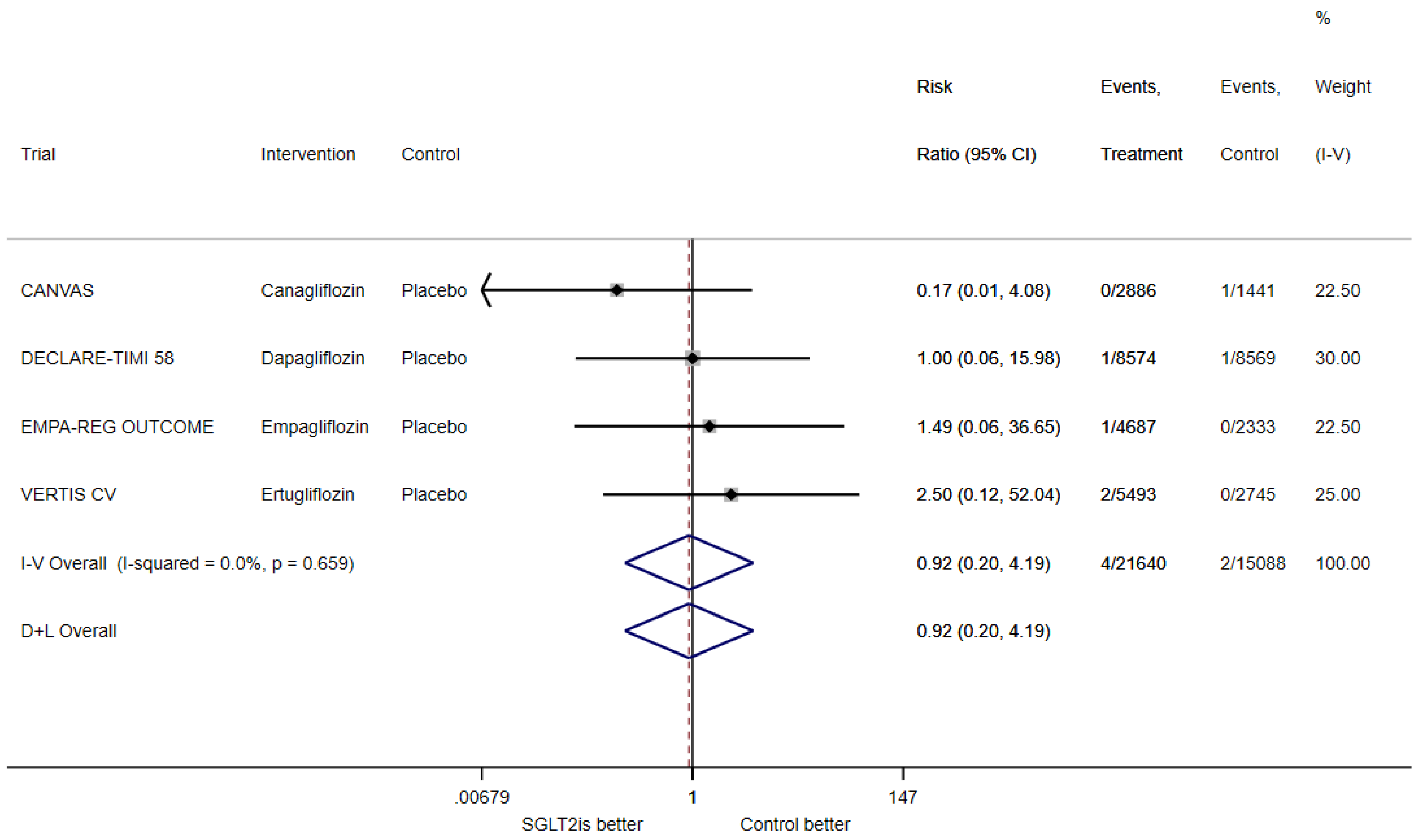

Figure S129 Meta-analysis of SGLT2is and Respiratory arrest

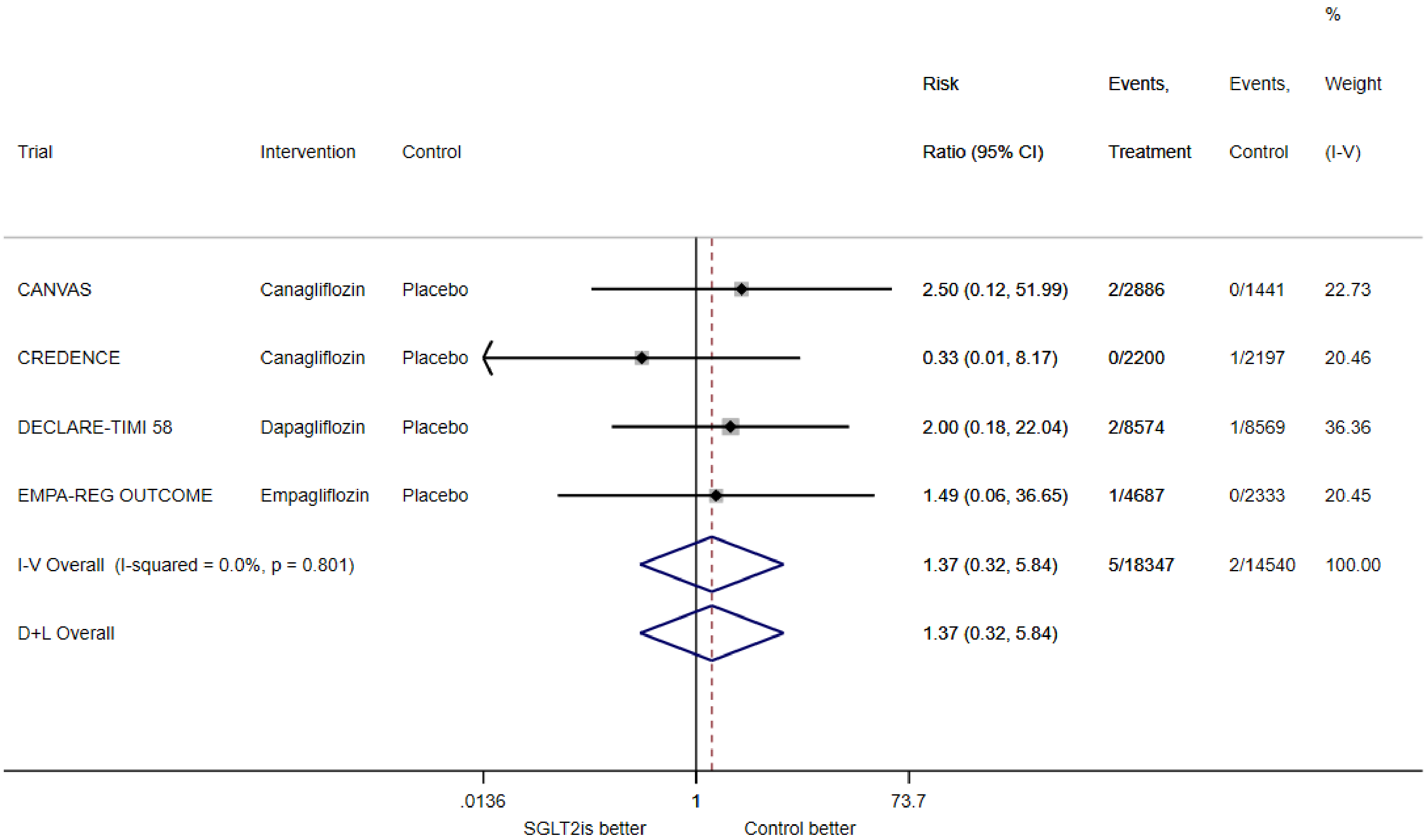

Figure S130 Meta-analysis of SGLT2is and Respiratory distress

Trial	Intervention	Control	Risk Ratio (95% CI)	Events, Treatment	Events, Control	% Weight (I-V)
CANVAS	Canagliflozin	Placebo	0.50 (0.16, 1.55)	6/2886	6/1441	8.14
CANVAS-R	Canagliflozin	Placebo	0.33 (0.03, 3.20)	1/2904	3/2903	2.03
CREDENCE	Canagliflozin	Placebo	1.00 (0.42, 2.39)	10/2200	10/2197	13.59
DECLARE-TIMI 58	Dapagliflozin	Placebo	1.21 (0.70, 2.07)	29/8574	24/8569	35.63
EMPA-REG OUTCOME	Empagliflozin	Placebo	0.33 (0.12, 0.93)	6/4687	9/2333	9.76
VERTIS CV	Ertugliflozin	Placebo	0.68 (0.31, 1.48)	15/5493	11/2745	17.23
DAPA-CKD	Dapagliflozin	Placebo	0.33 (0.07, 1.65)	2/2149	6/2149	4.06
EMPEROR-Reduced	Empagliflozin	Placebo	0.40 (0.08, 2.06)	2/1863	5/1863	3.87
DAPA-HF	Dapagliflozin	Placebo	0.43 (0.11, 1.66)	3/2368	7/2368	5.69
I-V Overall (I-squared = 11.5%, p = 0.339)			0.73 (0.53, 1.01)	74/33124	81/26568	100.00
D+L Overall			0.69 (0.48, 0.99)			

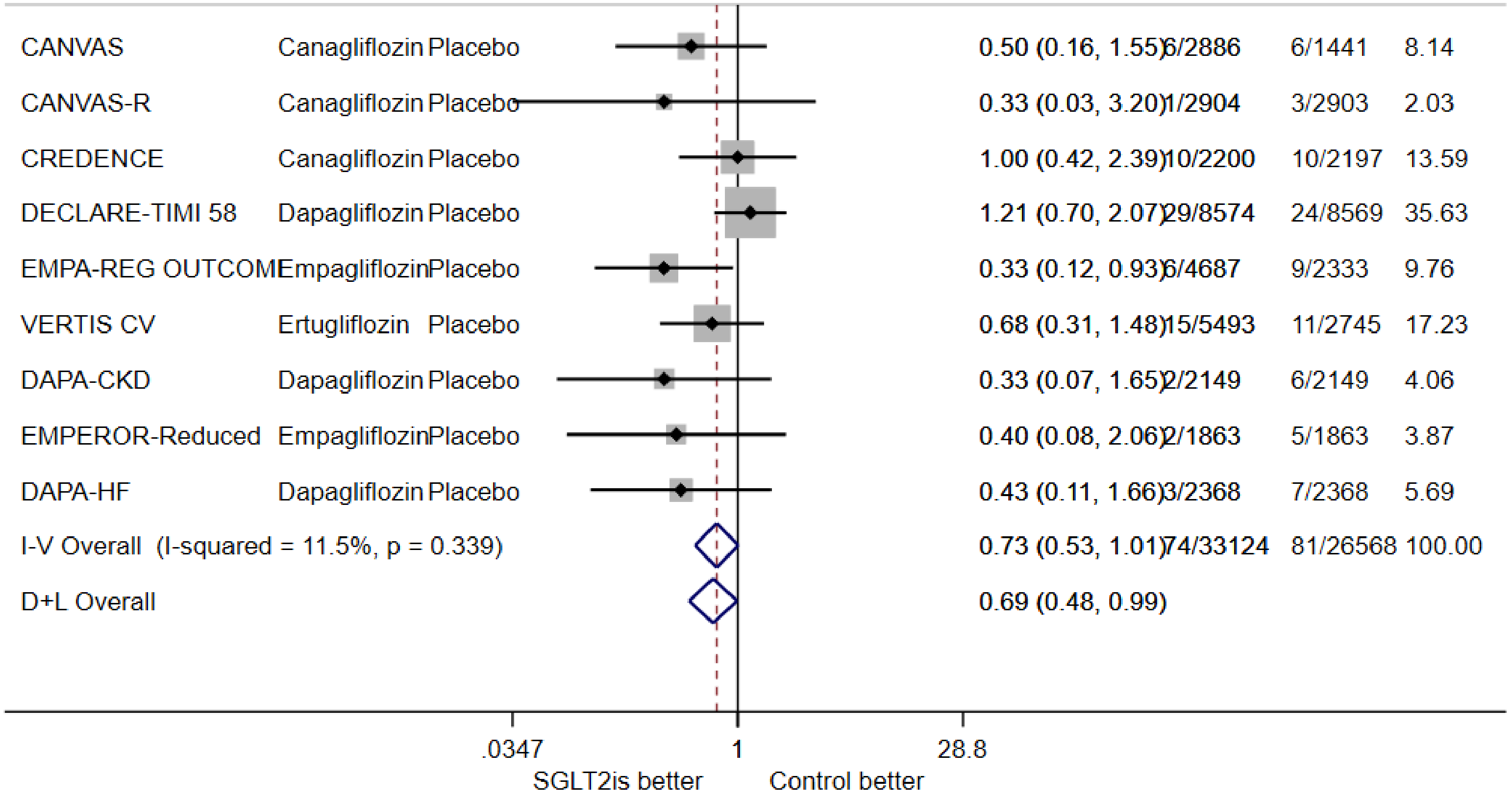

Figure S131 Meta-analysis of SGLT2is and Respiratory failure

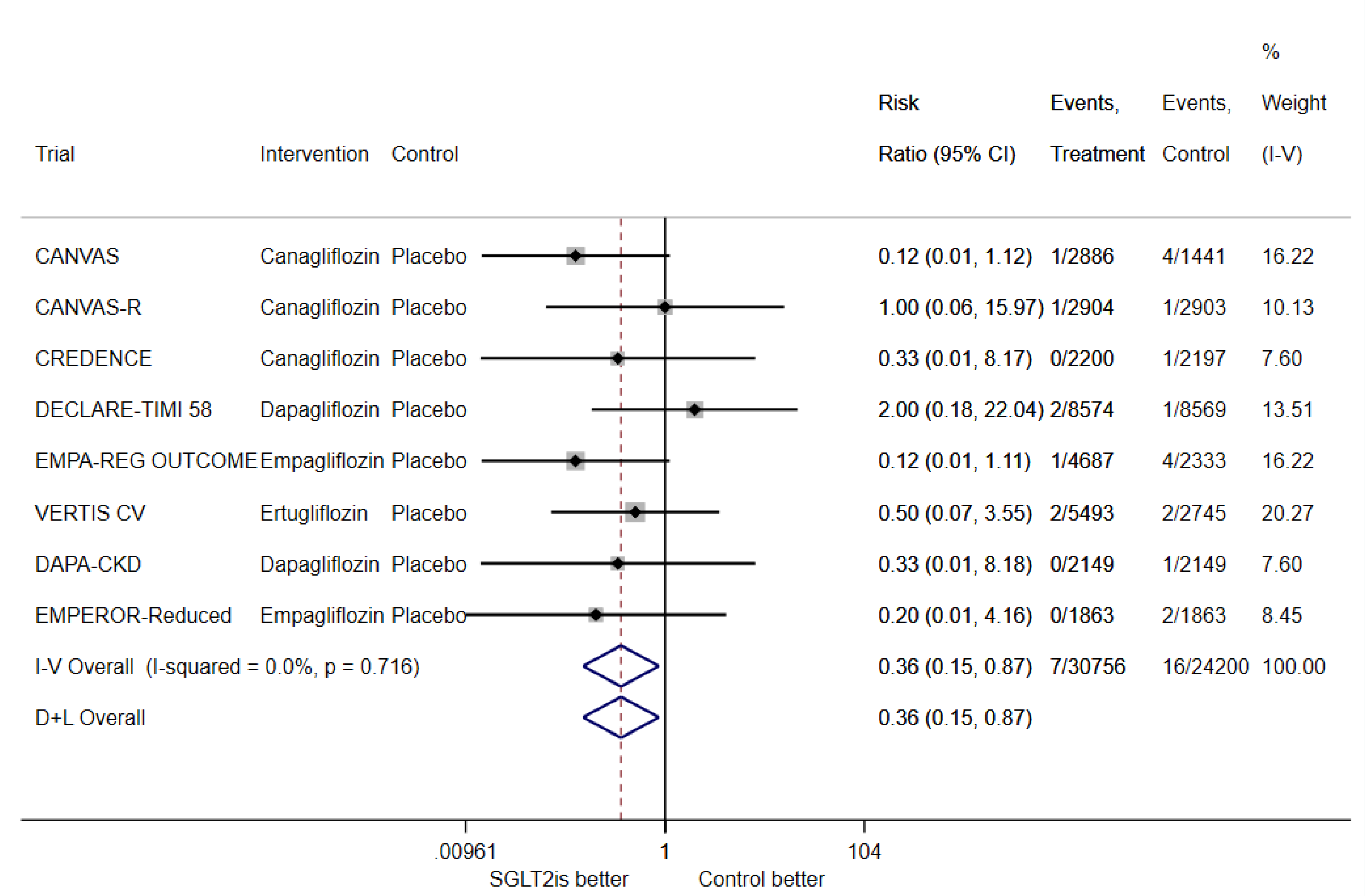

Figure S132 Meta-analysis of SGLT2is and Sleep apnoea syndrome

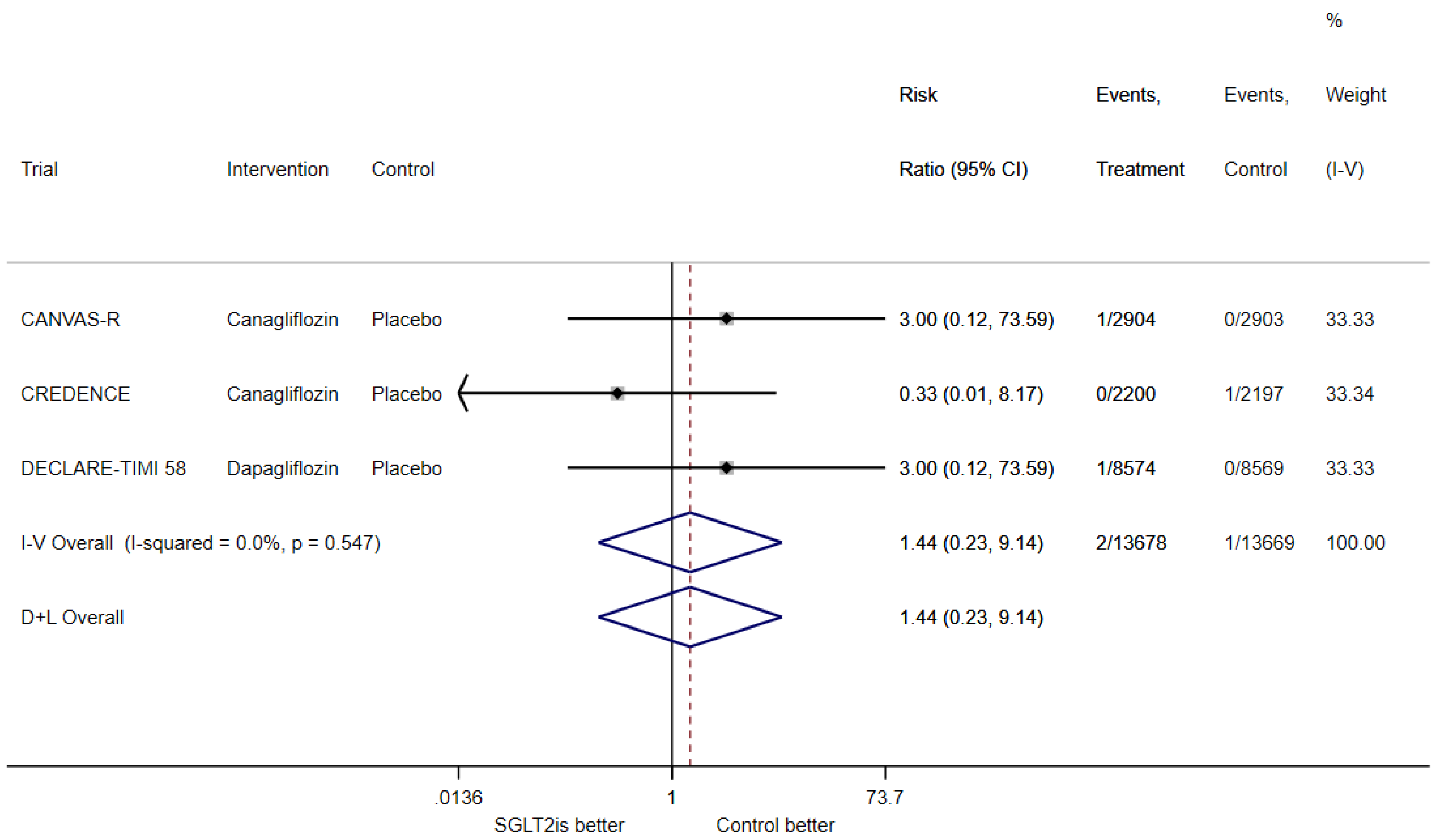

Figure S133 Meta-analysis of SGLT2is and Vocal cord leukoplakia

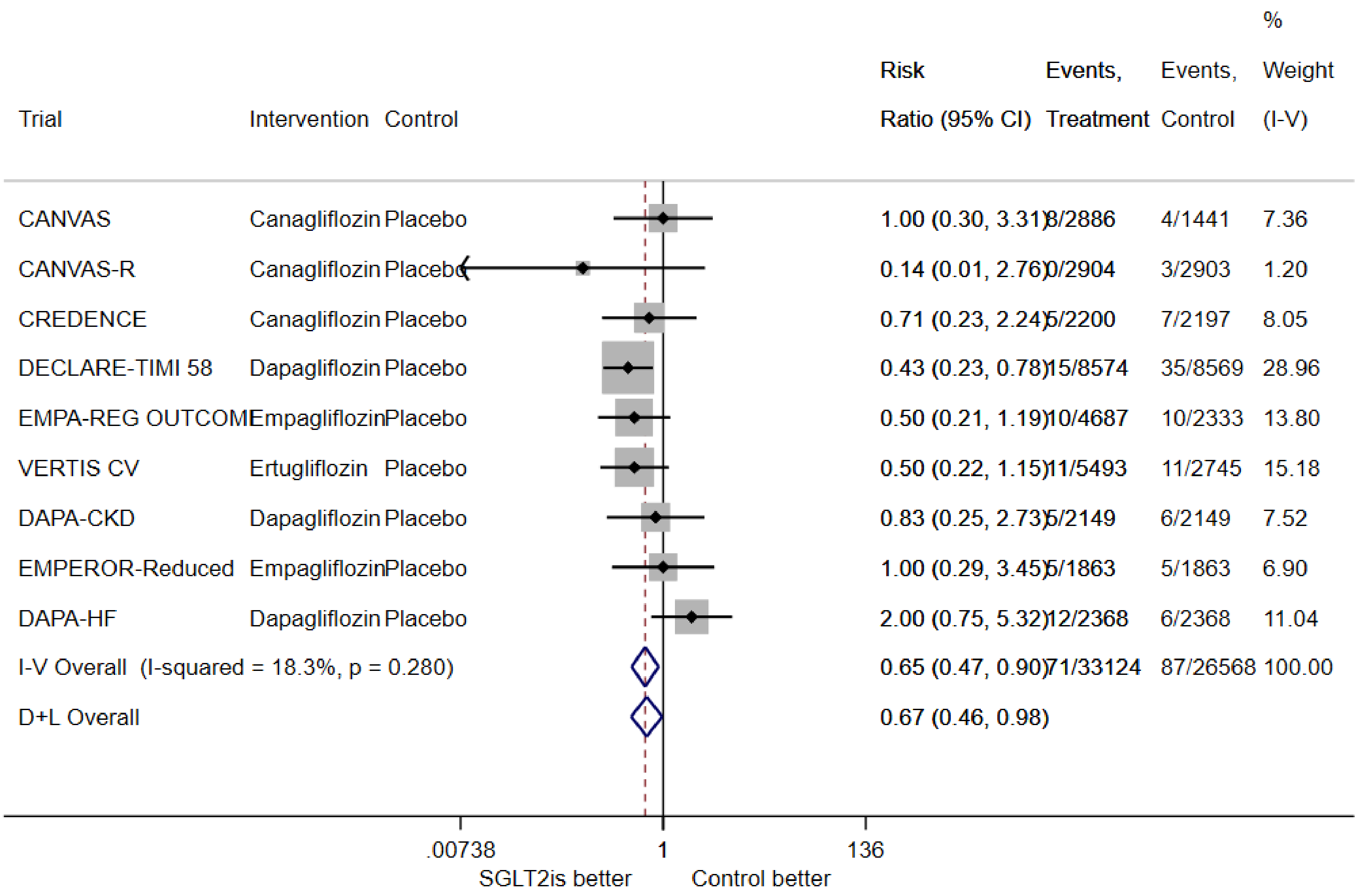

Figure S134 Meta-analysis of SGLT2is and Bronchitis

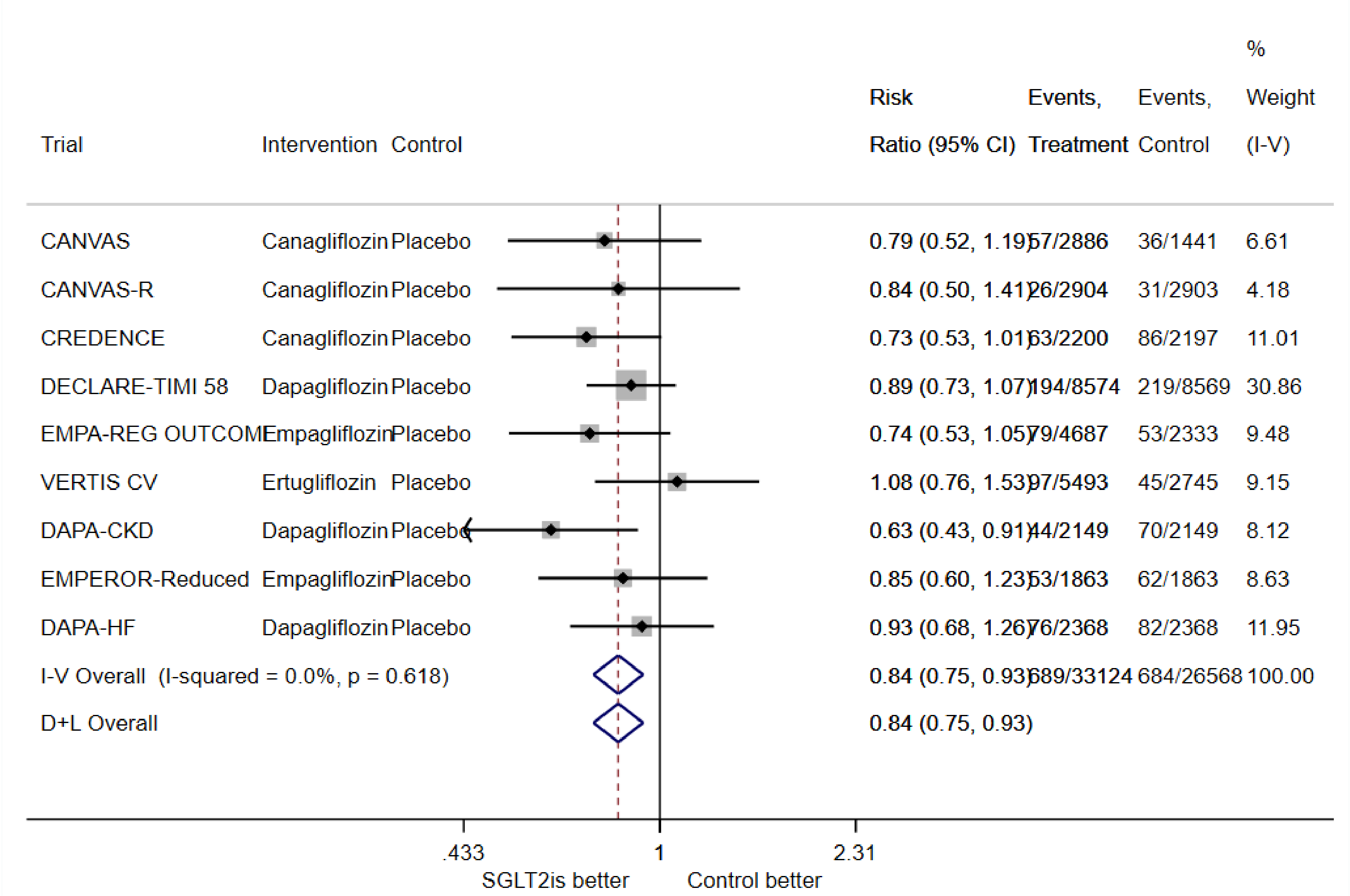

Figure S135 Meta-analysis of SGLT2is and Pneumonia

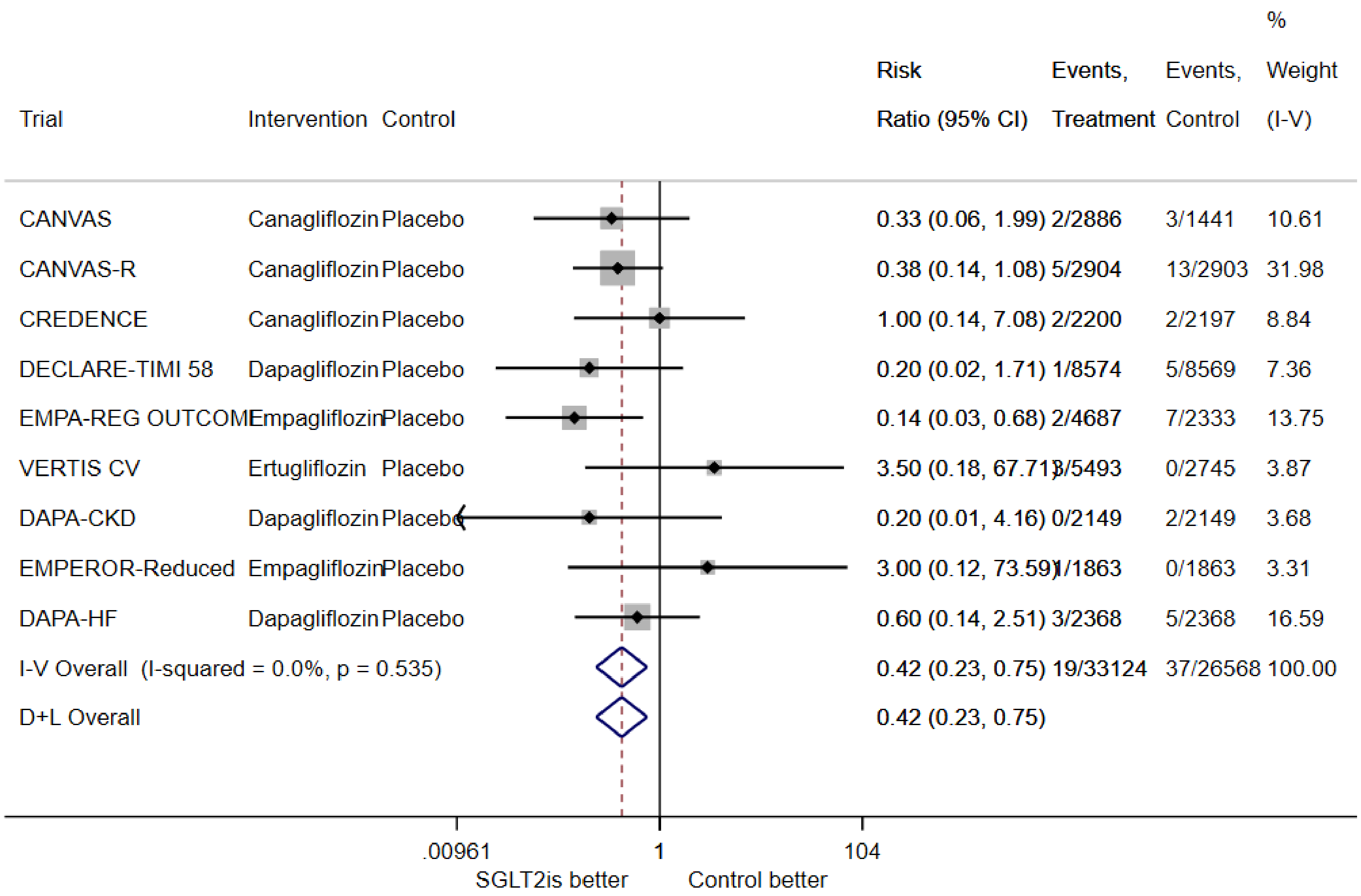

Figure S136 Meta-analysis of SGLT2is and Respiratory tract infection

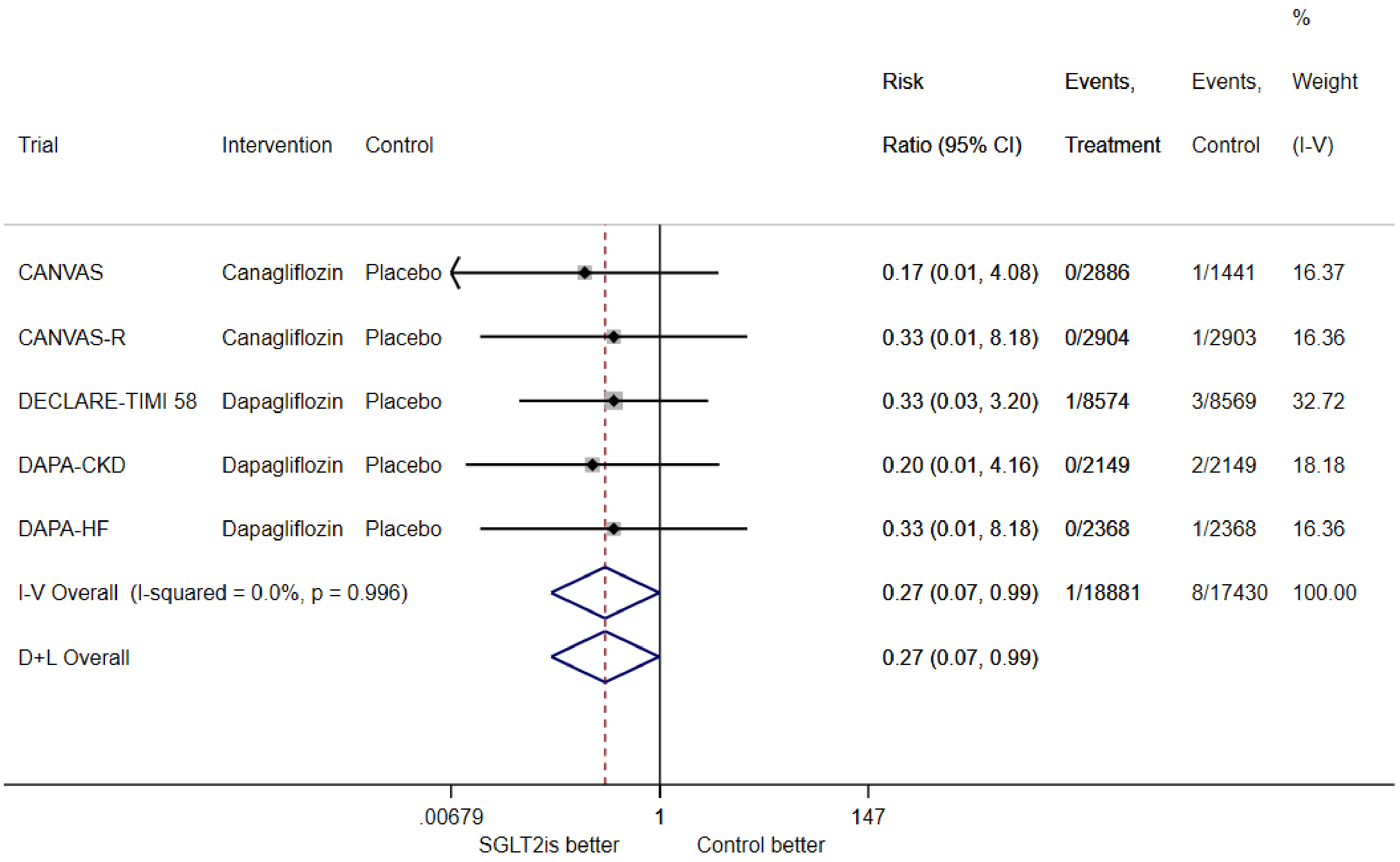

Figure S137 Meta-analysis of SGLT2is and Non-small cell lung cancer

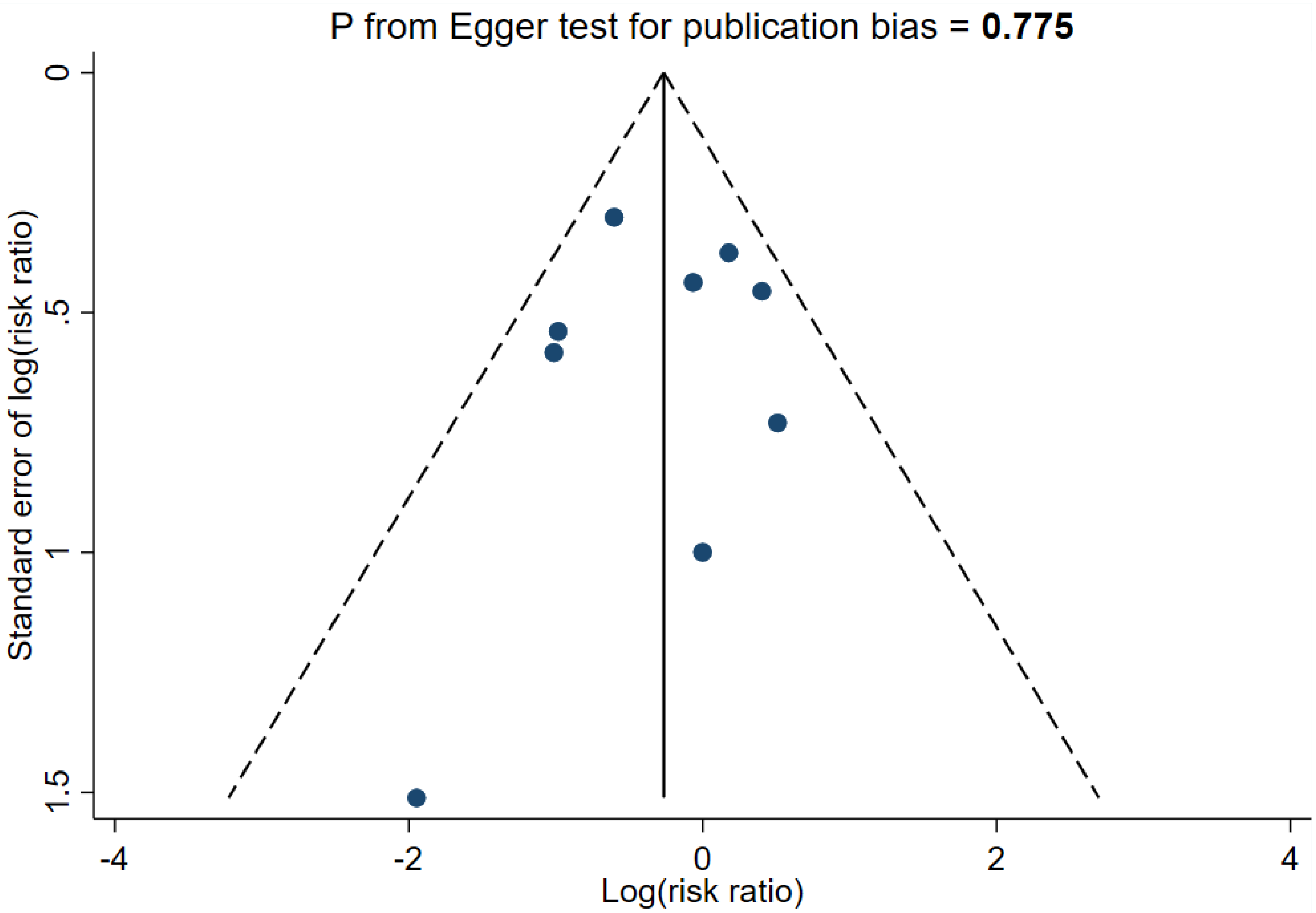

Figure S138 Funnel plot of Acute coronary syndrome

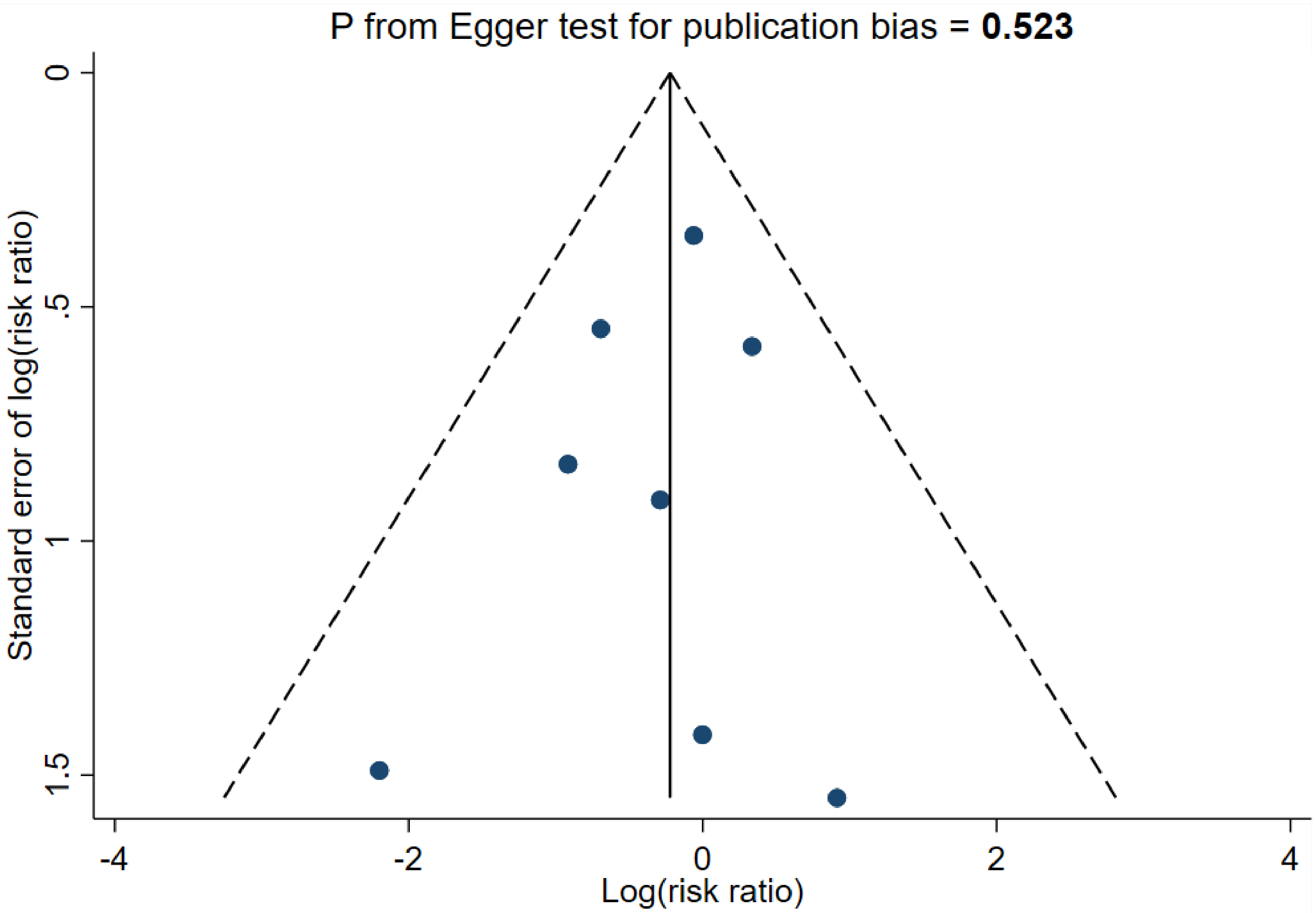

Figure S139 Funnel plot of Acute left ventricular failure

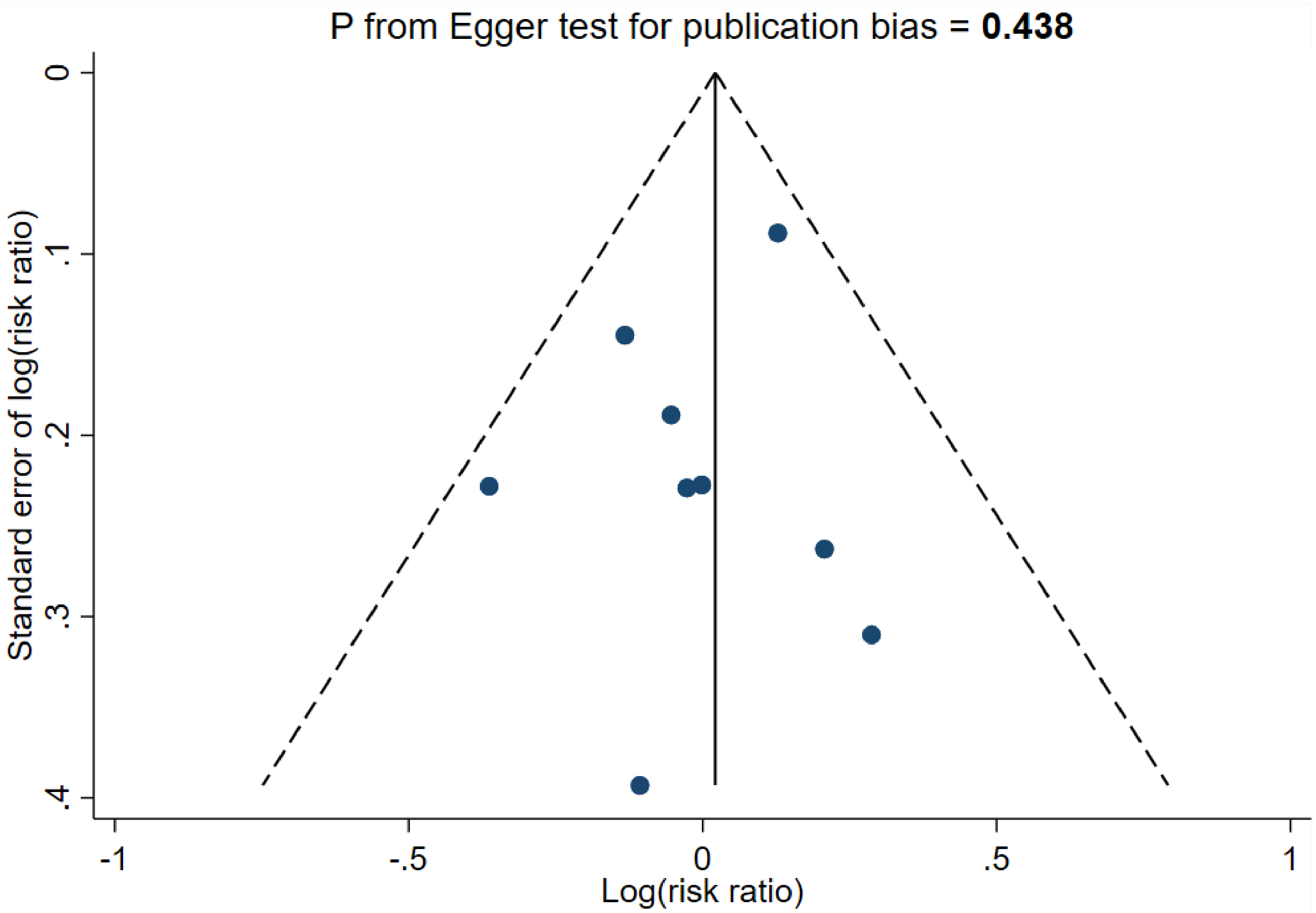

Figure S140 Funnel plot of Acute myocardial infarction

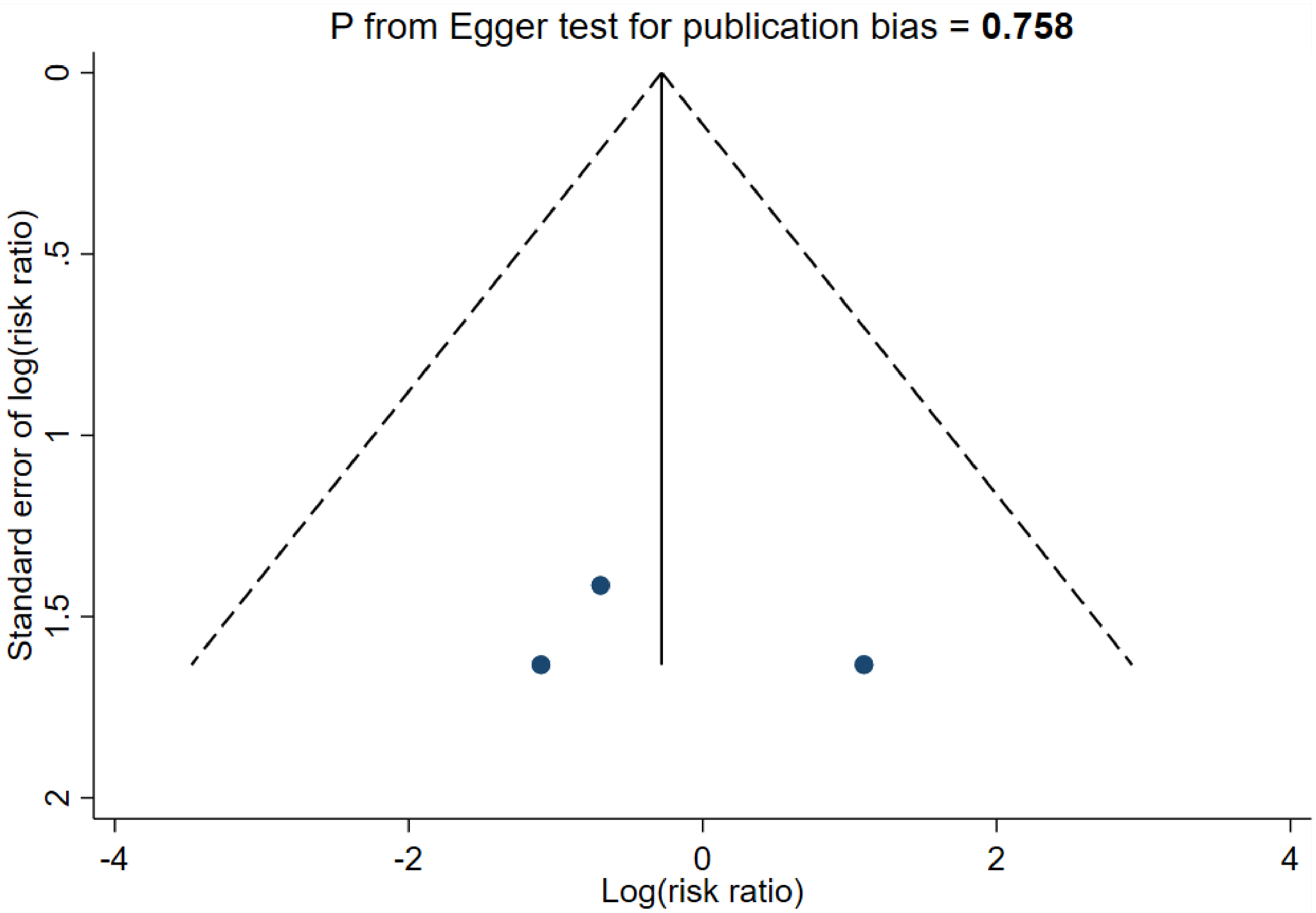

Figure S141 Funnel plot of Adams-stokes syndrome

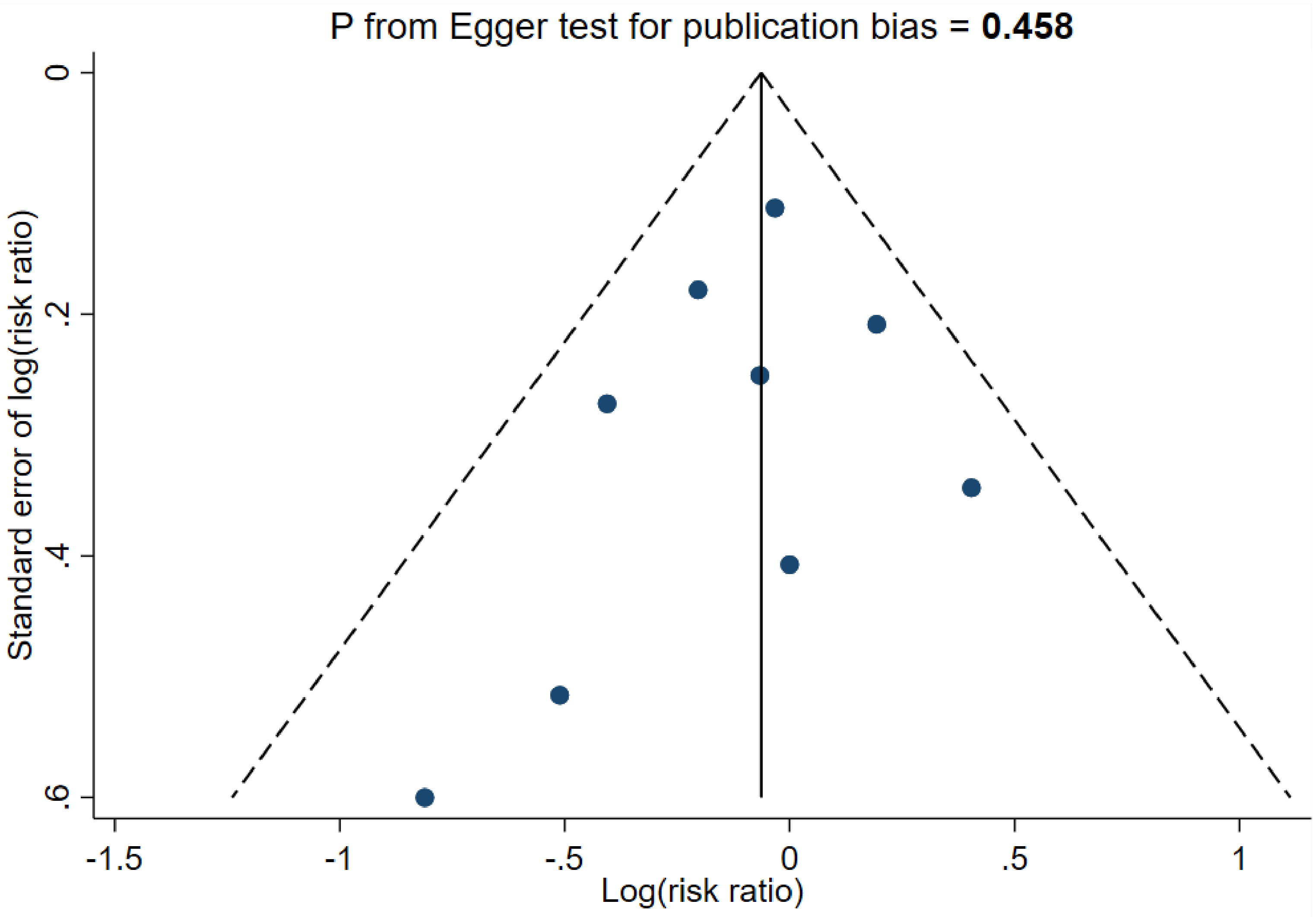

Figure S142 Funnel plot of Angina pectoris

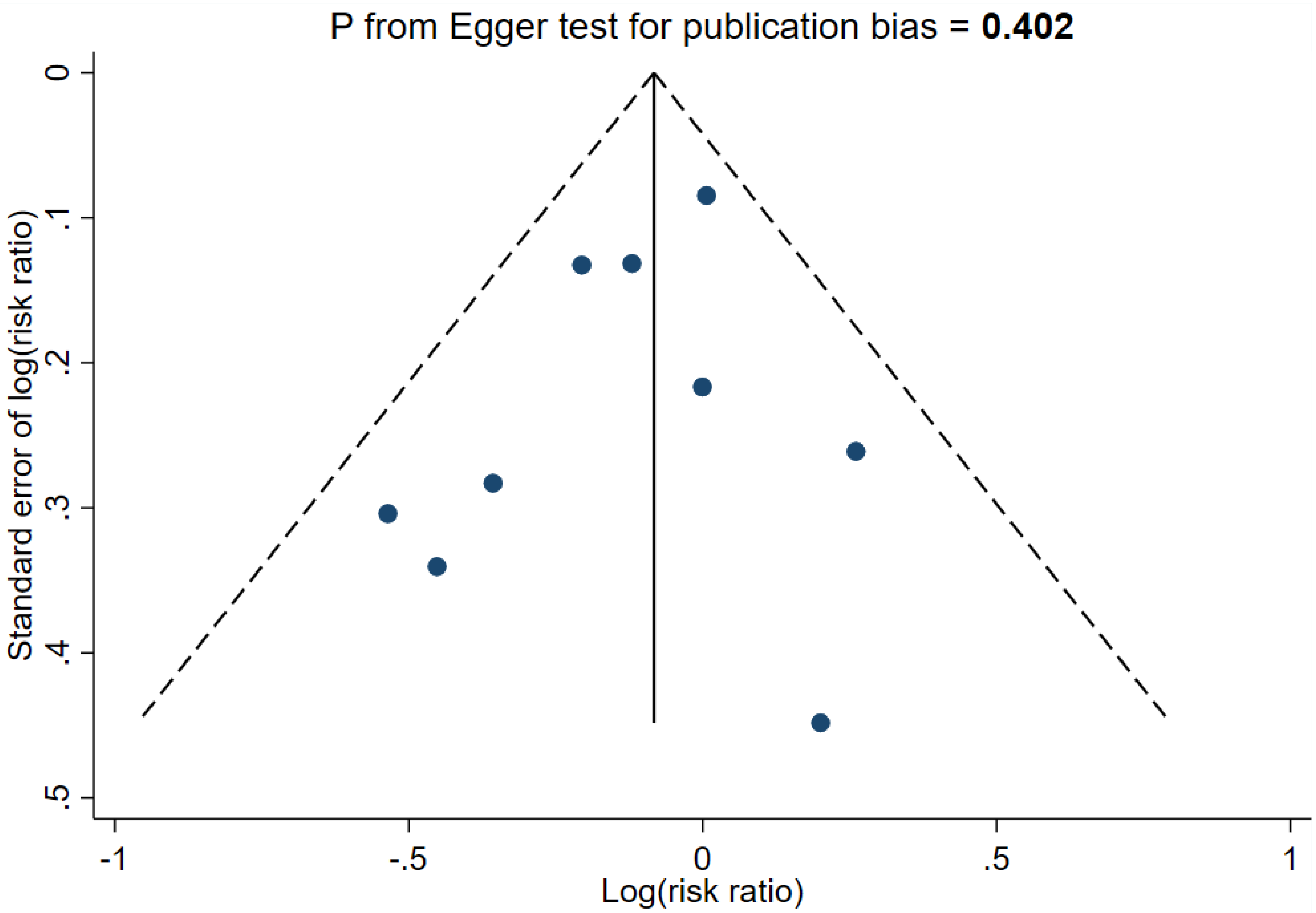

Figure S143 Funnel plot of Angina unstable

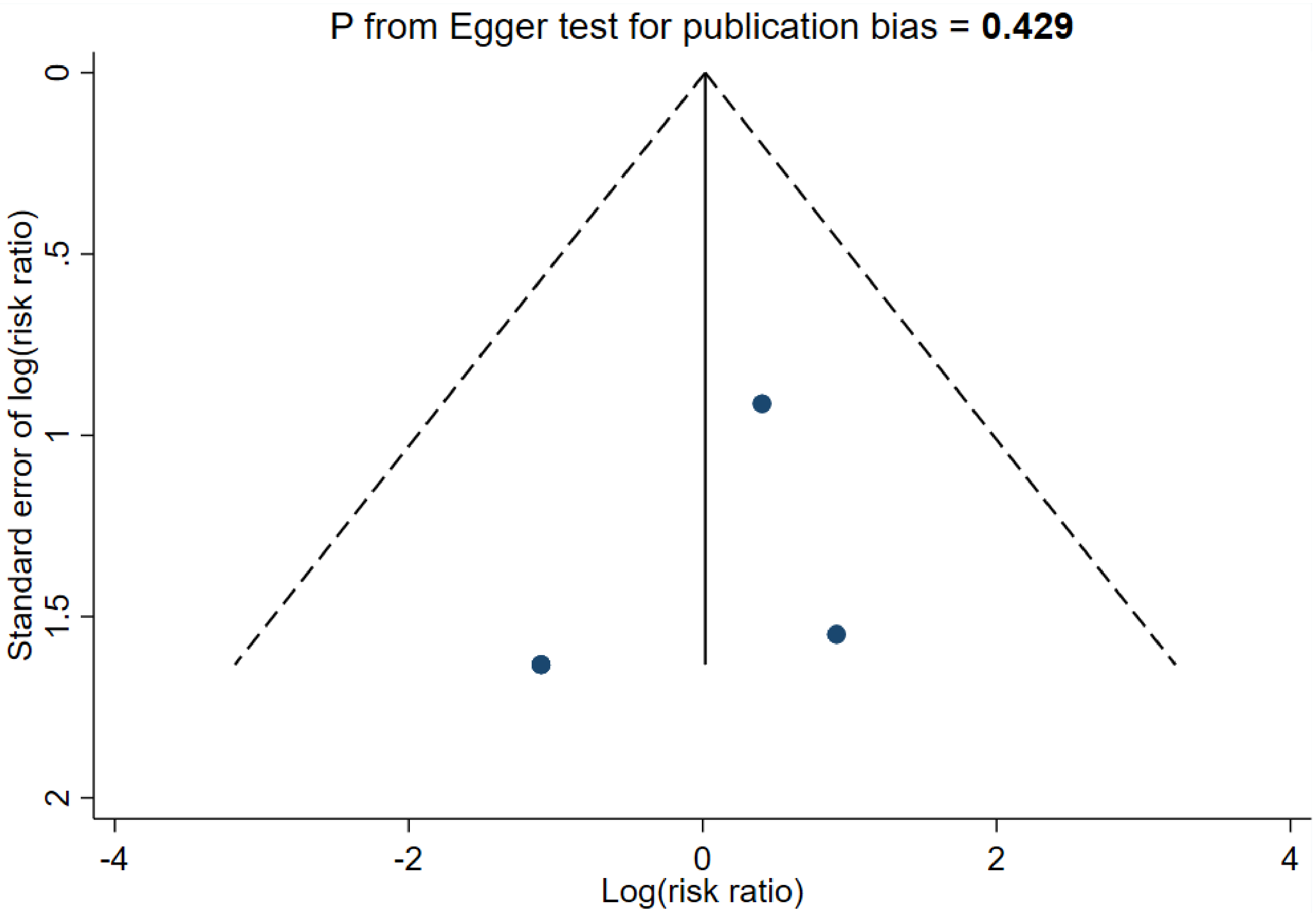

Figure S144 Funnel plot of Aortic valve disease

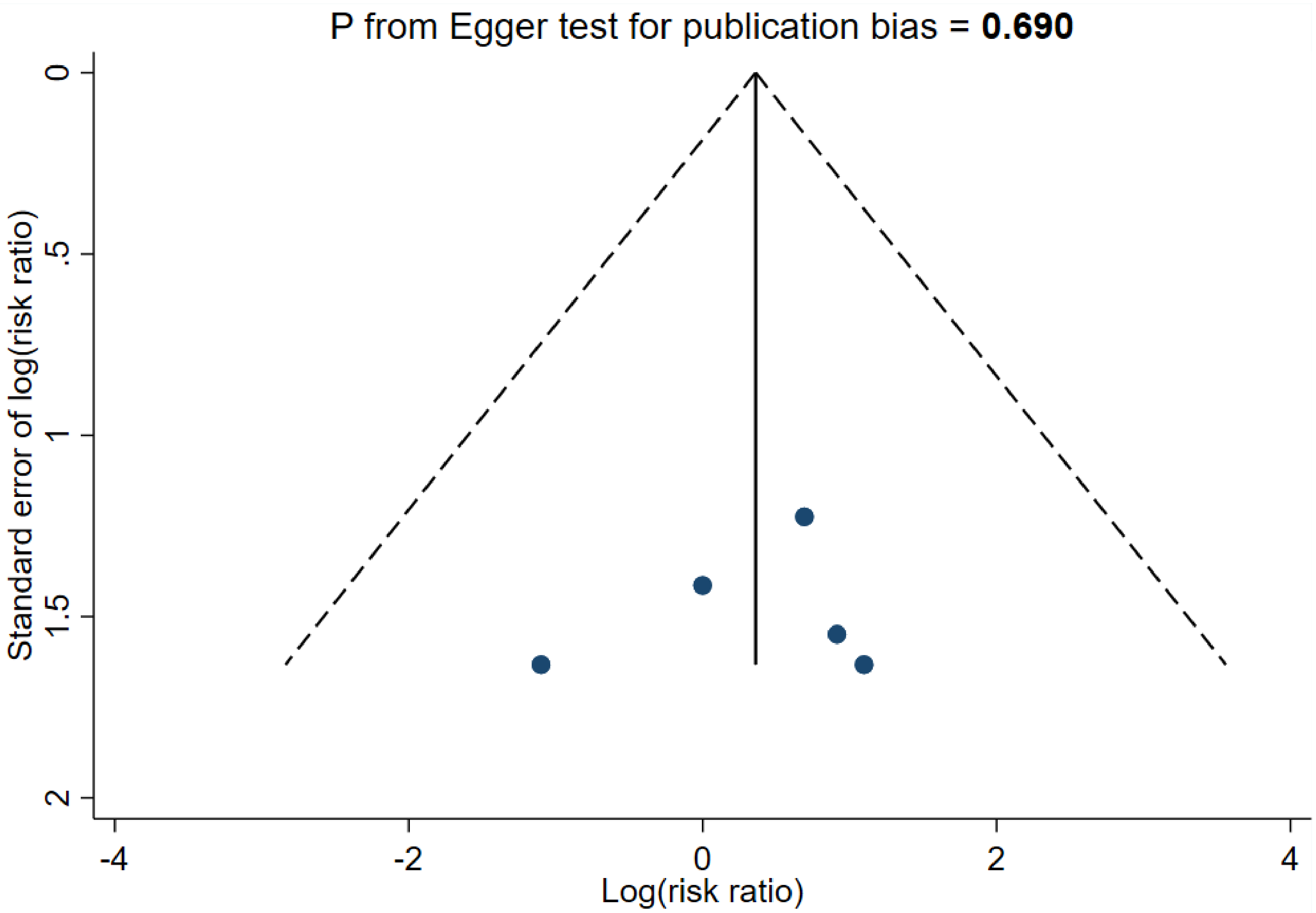

Figure S145 Funnel plot of Aortic valve incompetence

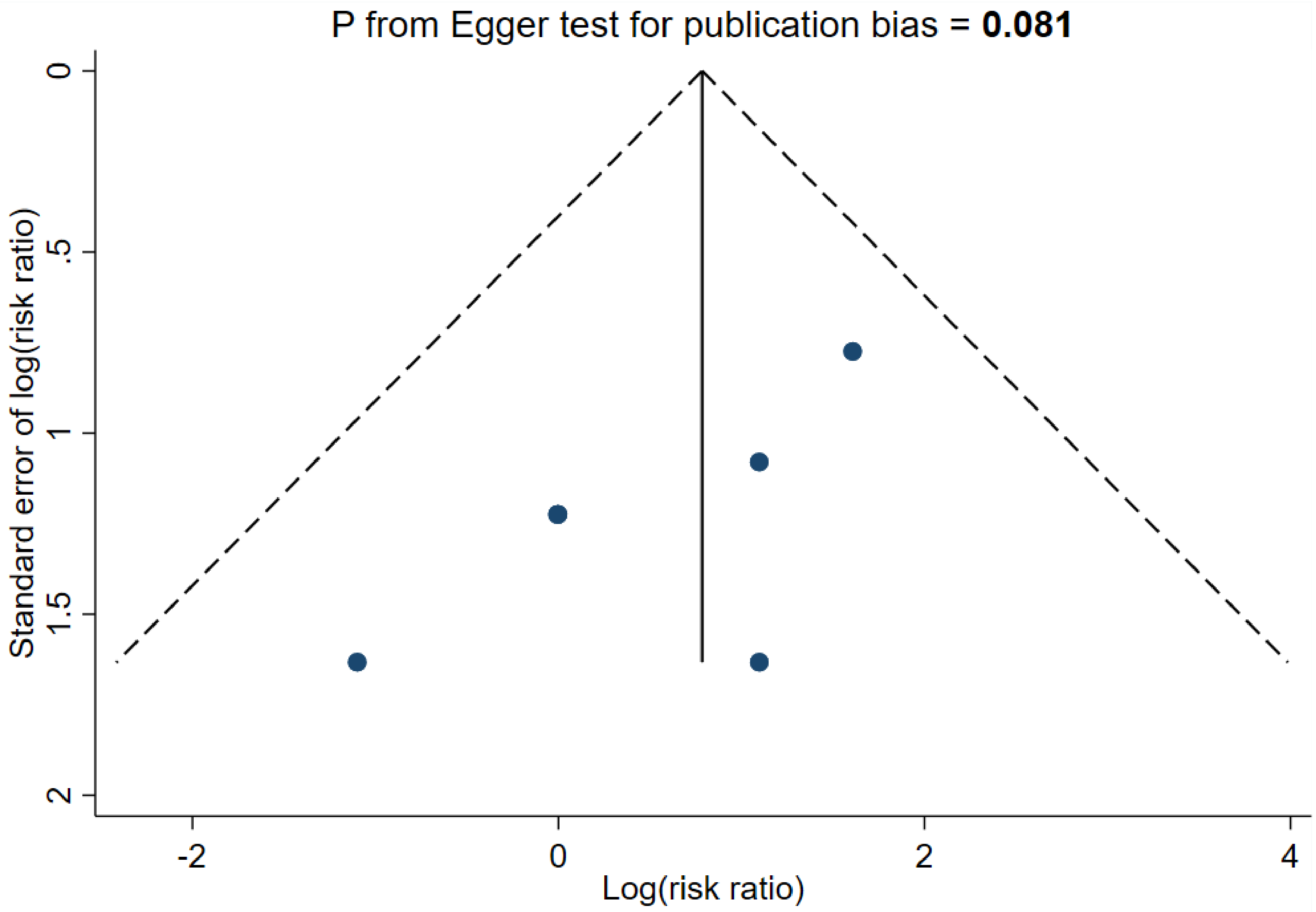

Figure S146 Funnel plot of Aortic valve stenosis

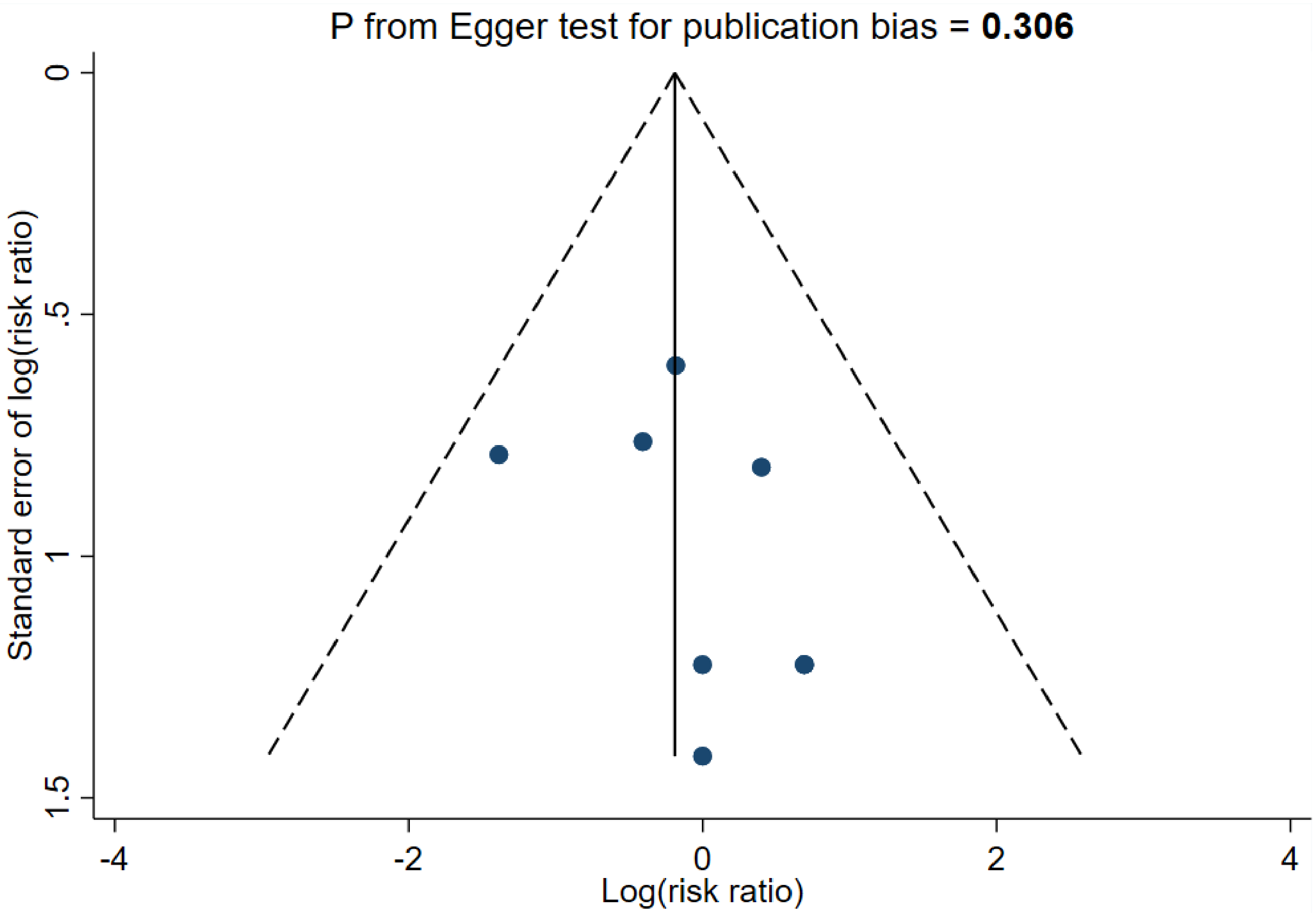

Figure S147 Funnel plot of Arrhythmia

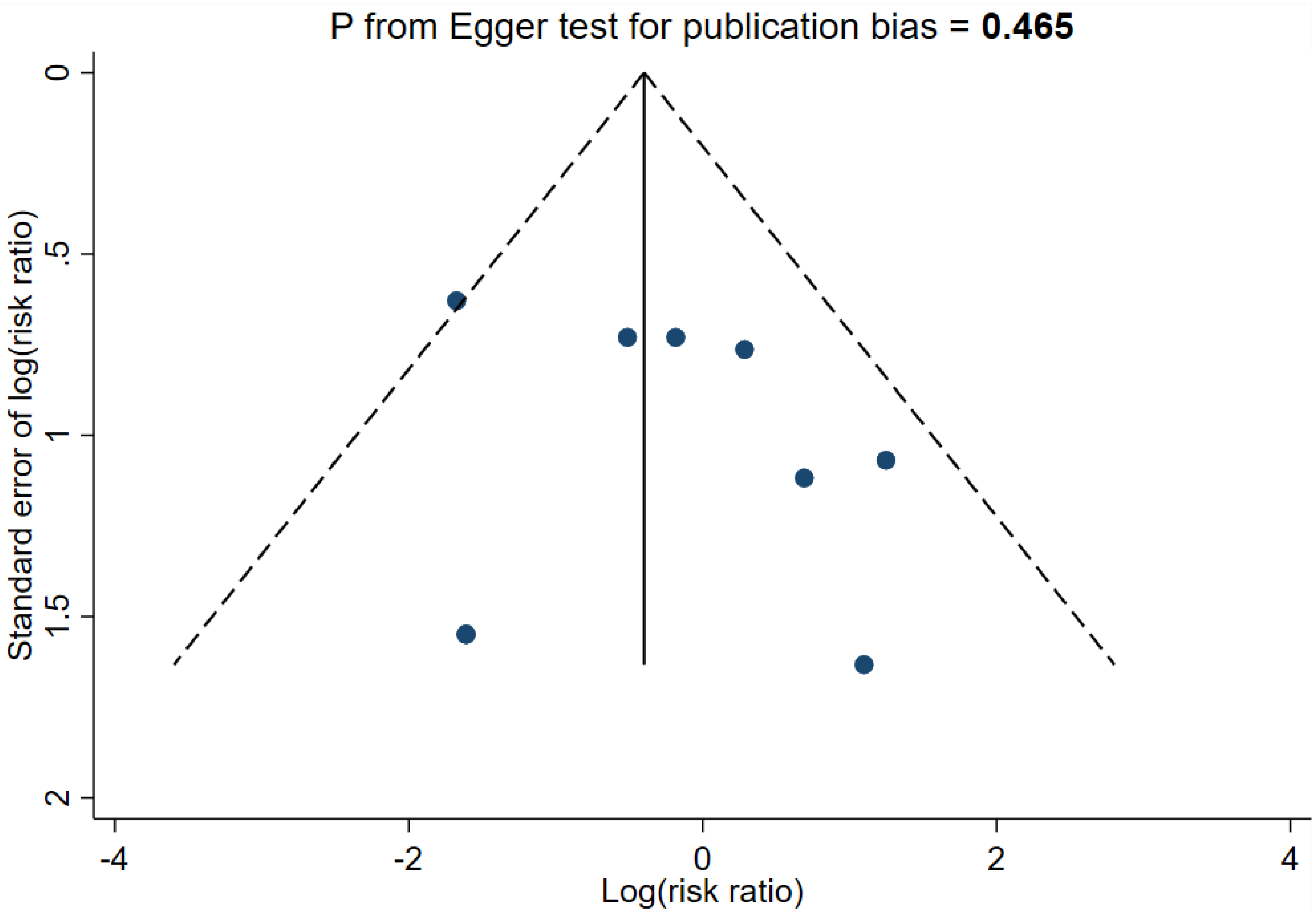

Figure S148 Funnel plot of Arteriosclerosis coronary artery

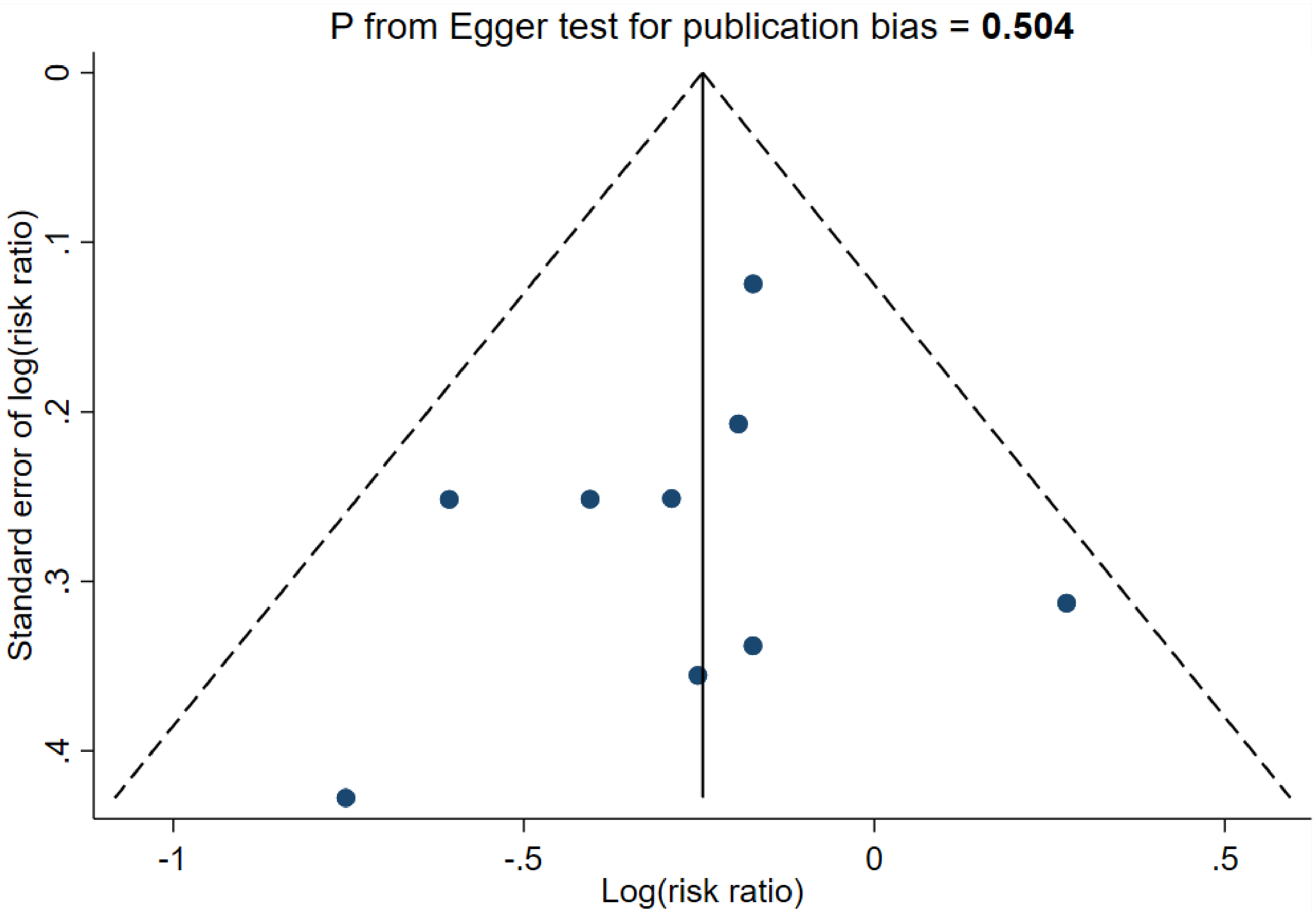

Figure S149 Funnel plot of Atrial fibrillation

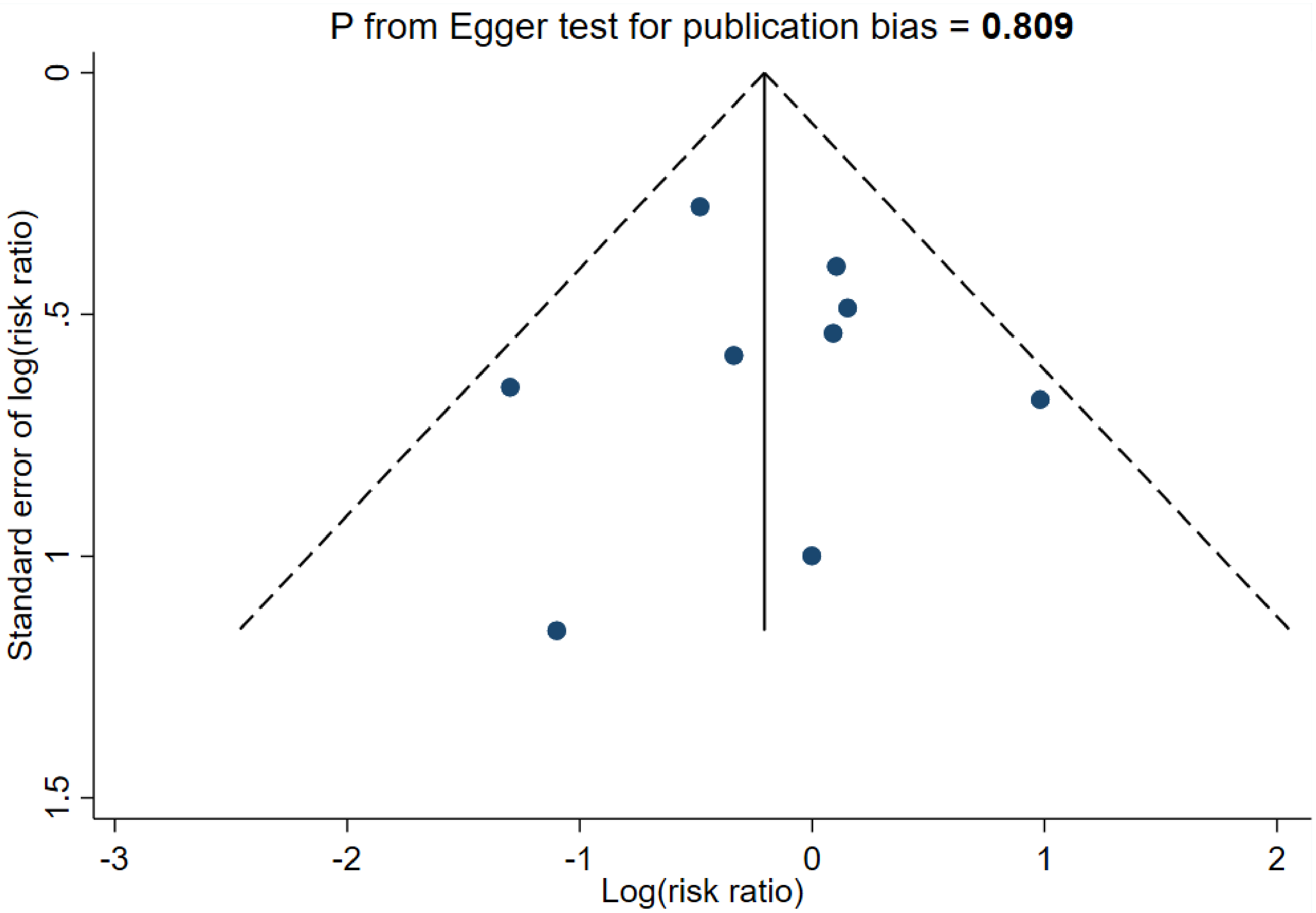

Figure S150 Funnel plot of Atrial flutter

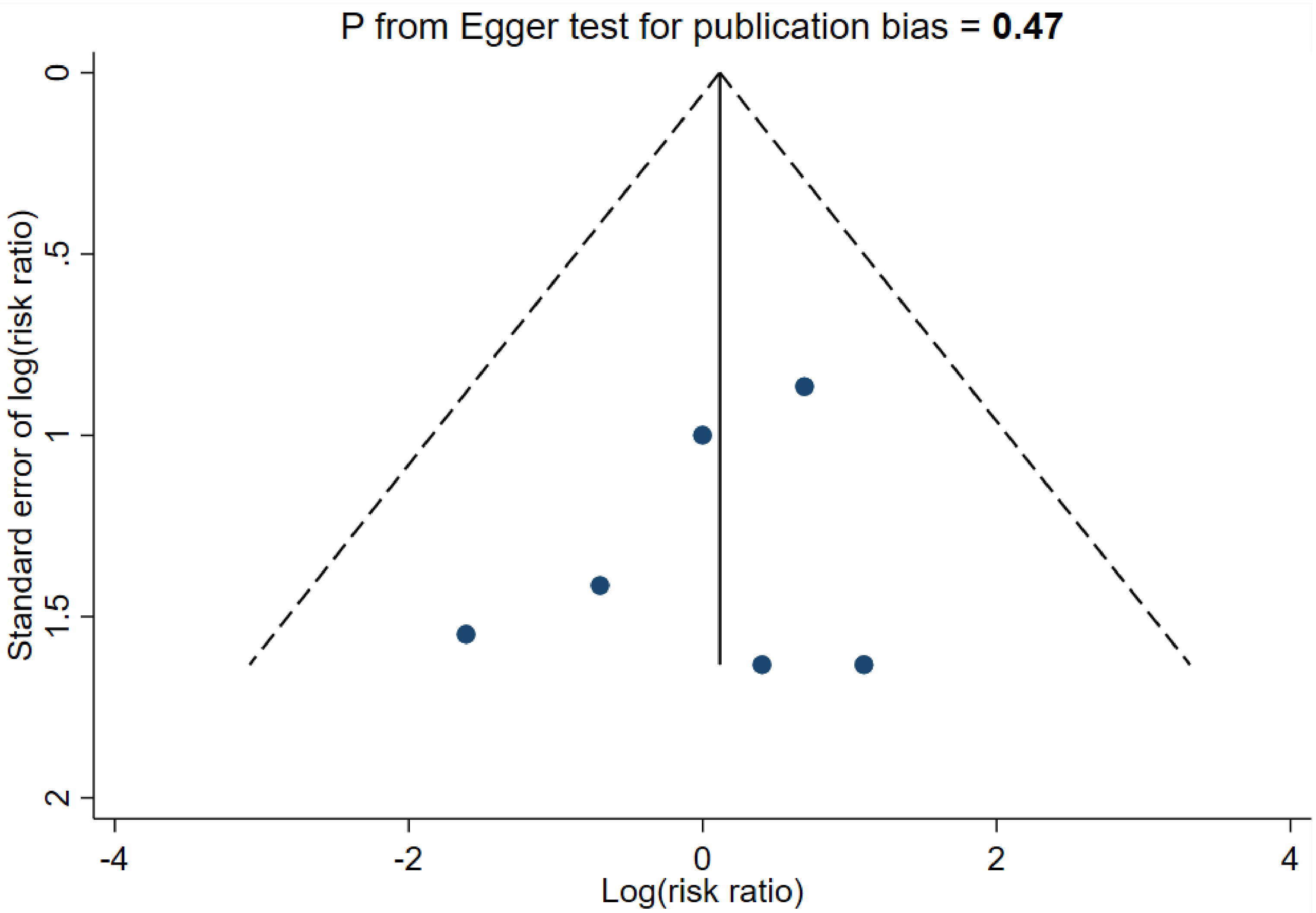

Figure S151 Funnel plot of Atrial tachycardia

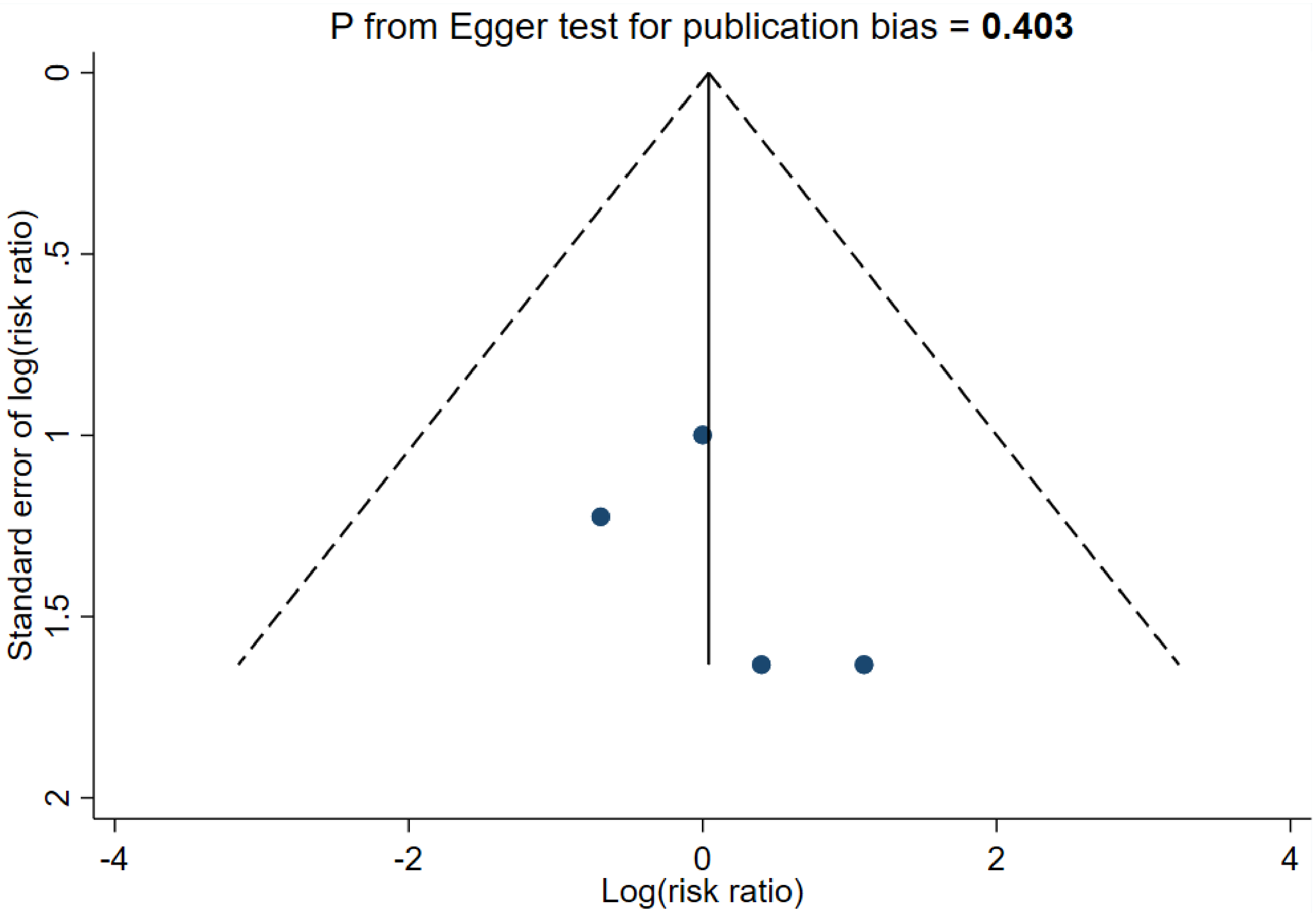

Figure S152 Funnel plot of Atrial thrombosis

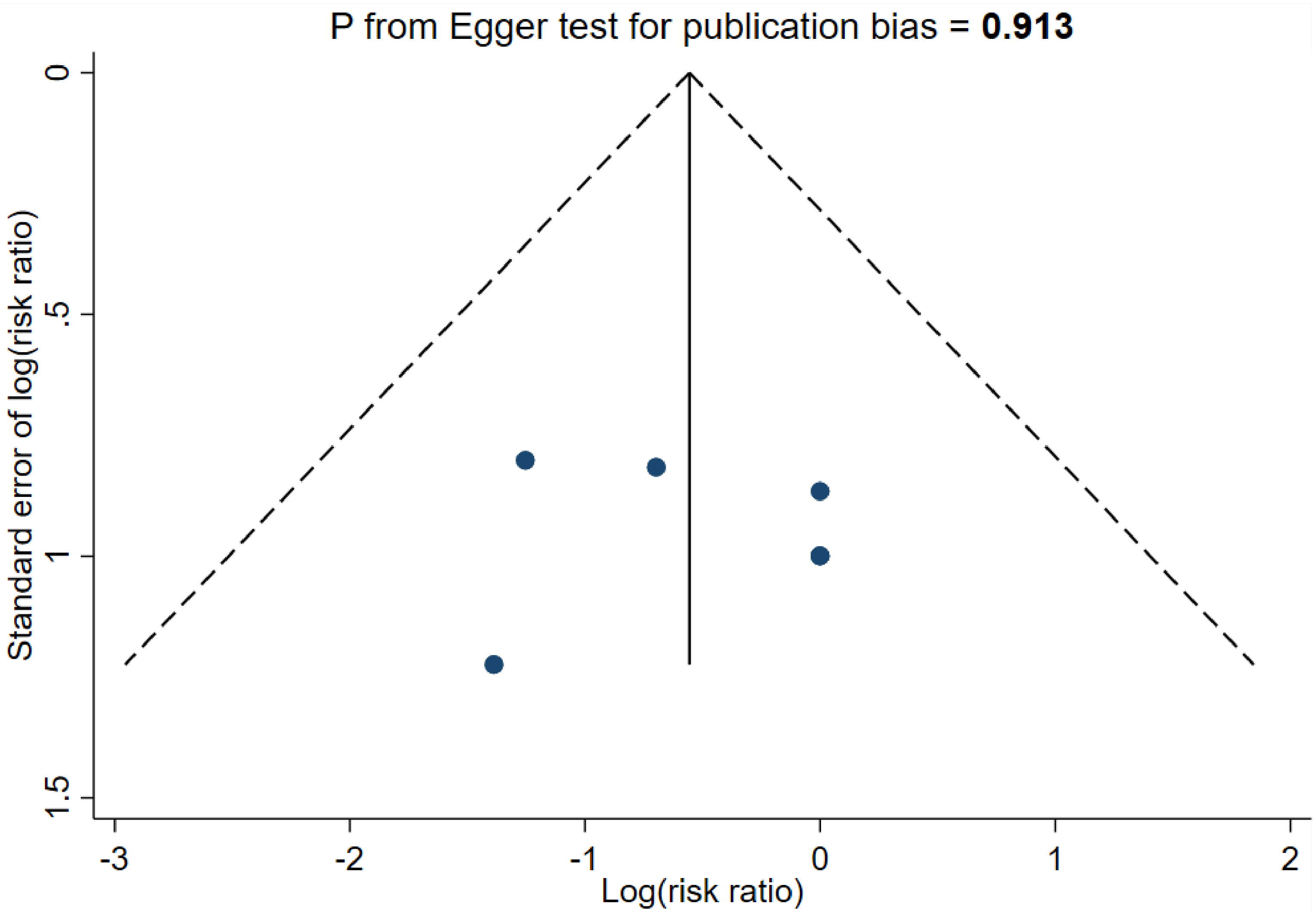

Figure S153 Funnel plot of Atrioventricular block

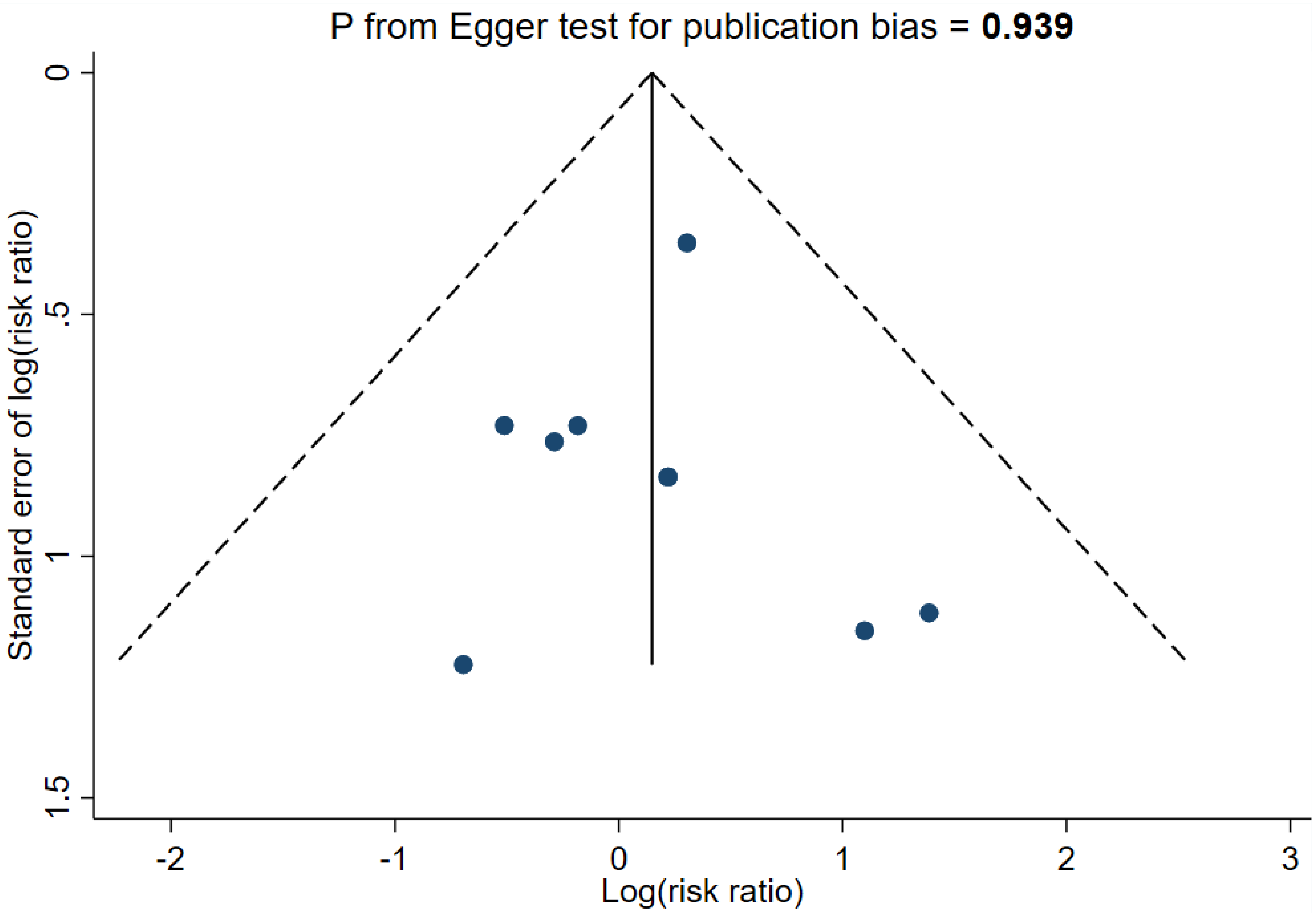

Figure S154 Funnel plot of Atrioventricular block complete

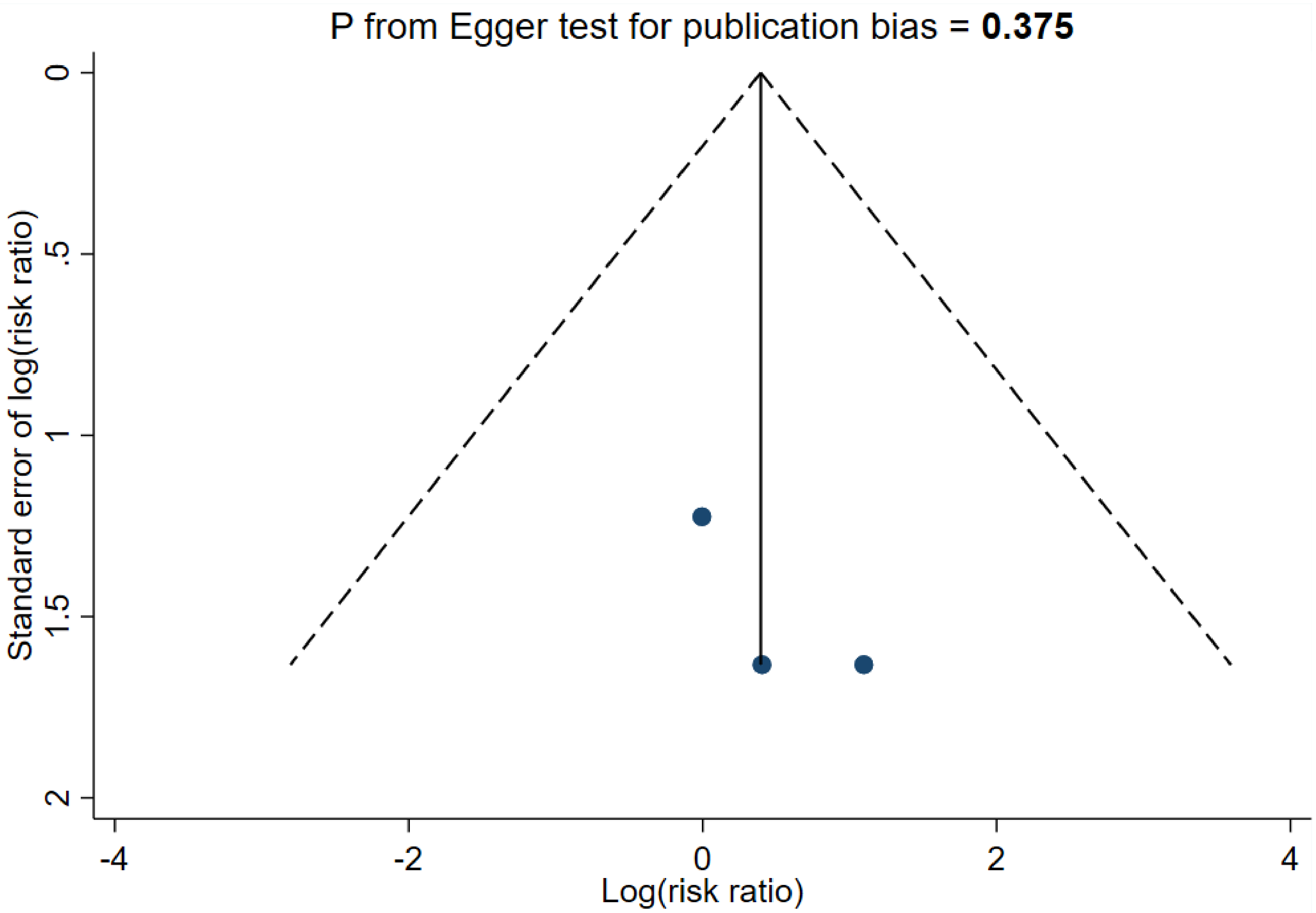

Figure S155 Funnel plot of Atrioventricular block first degree

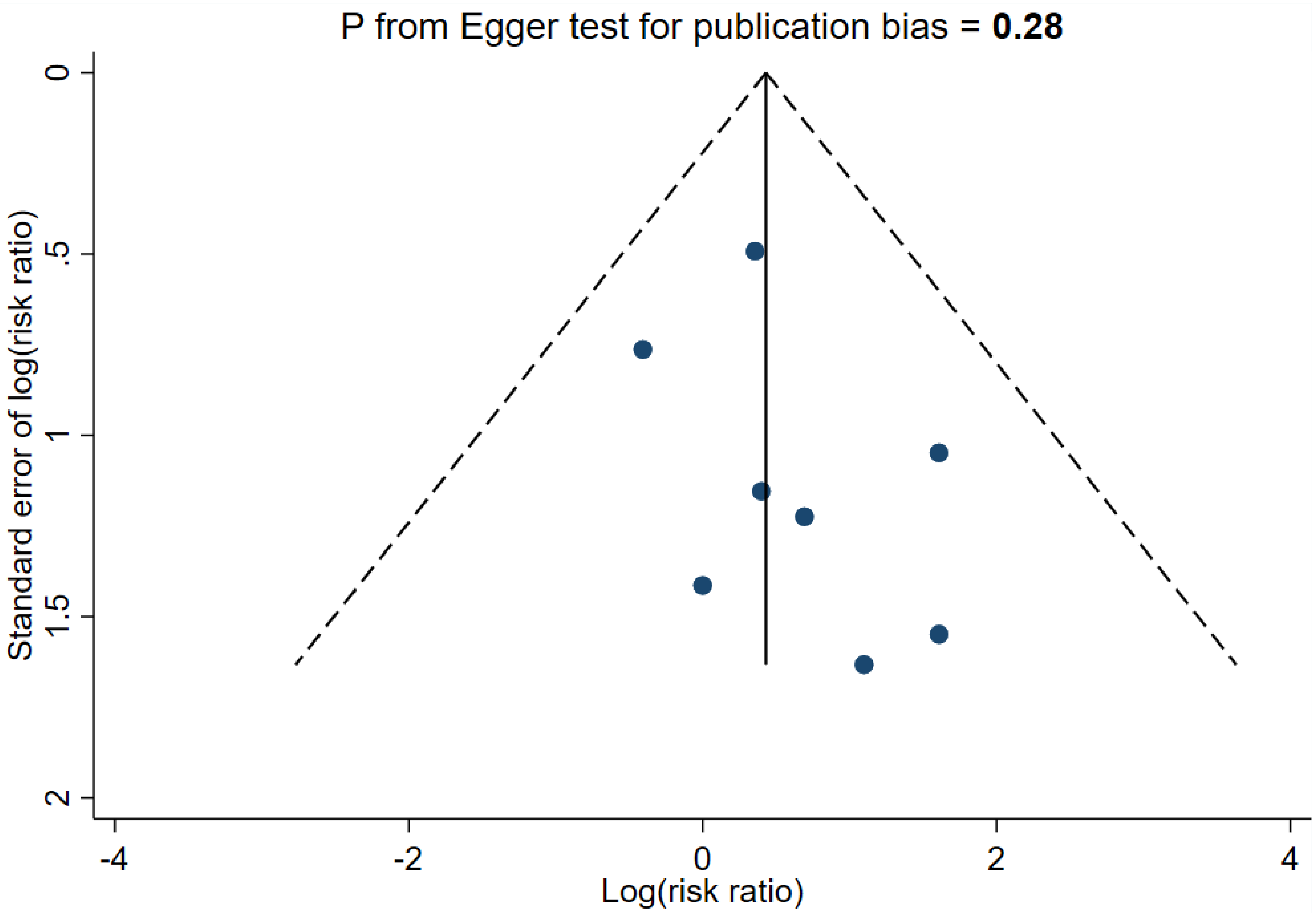

Figure S156 Funnel plot of Atrioventricular block second degree

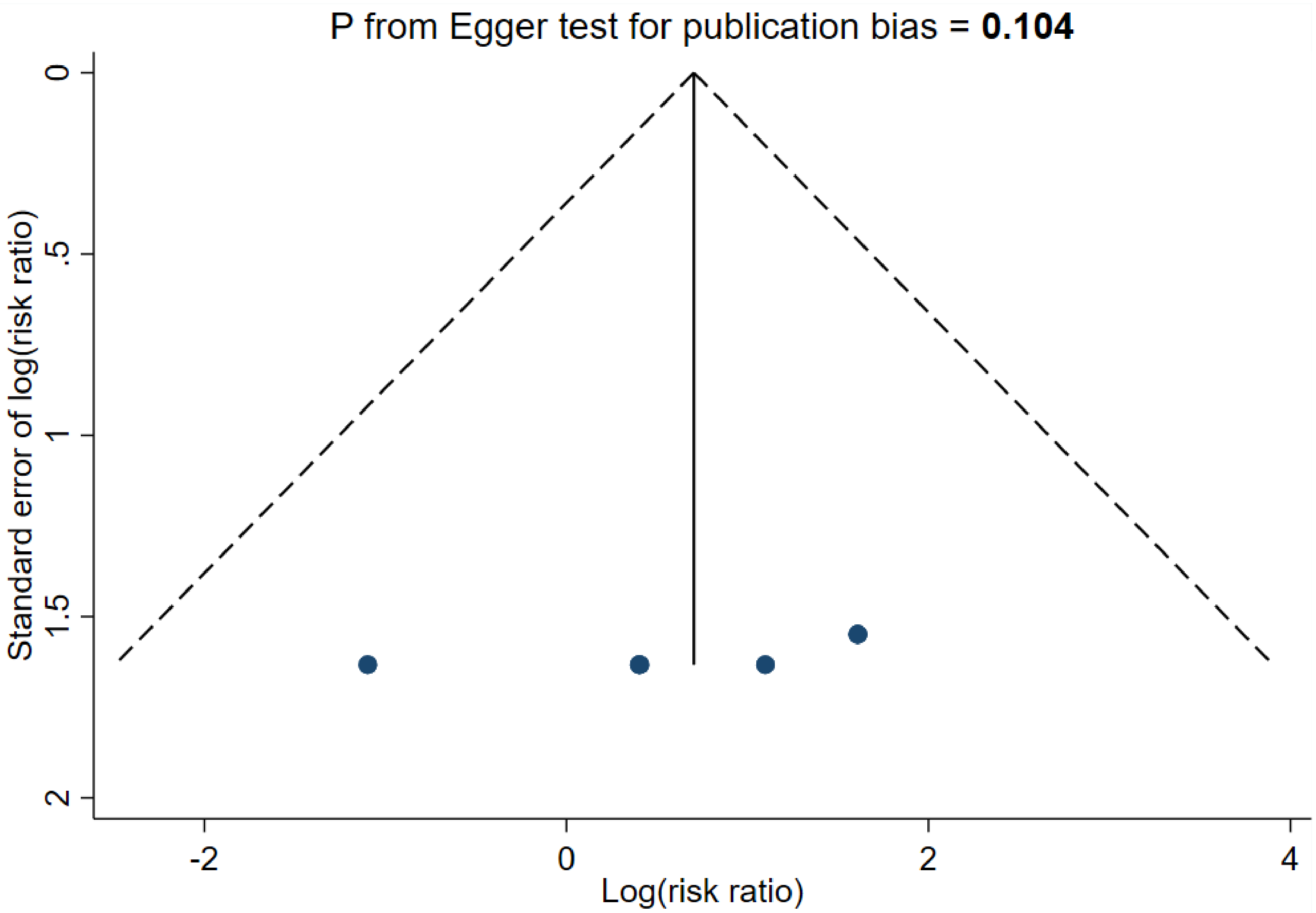

Figure S157 Funnel plot of Bradyarrhythmia

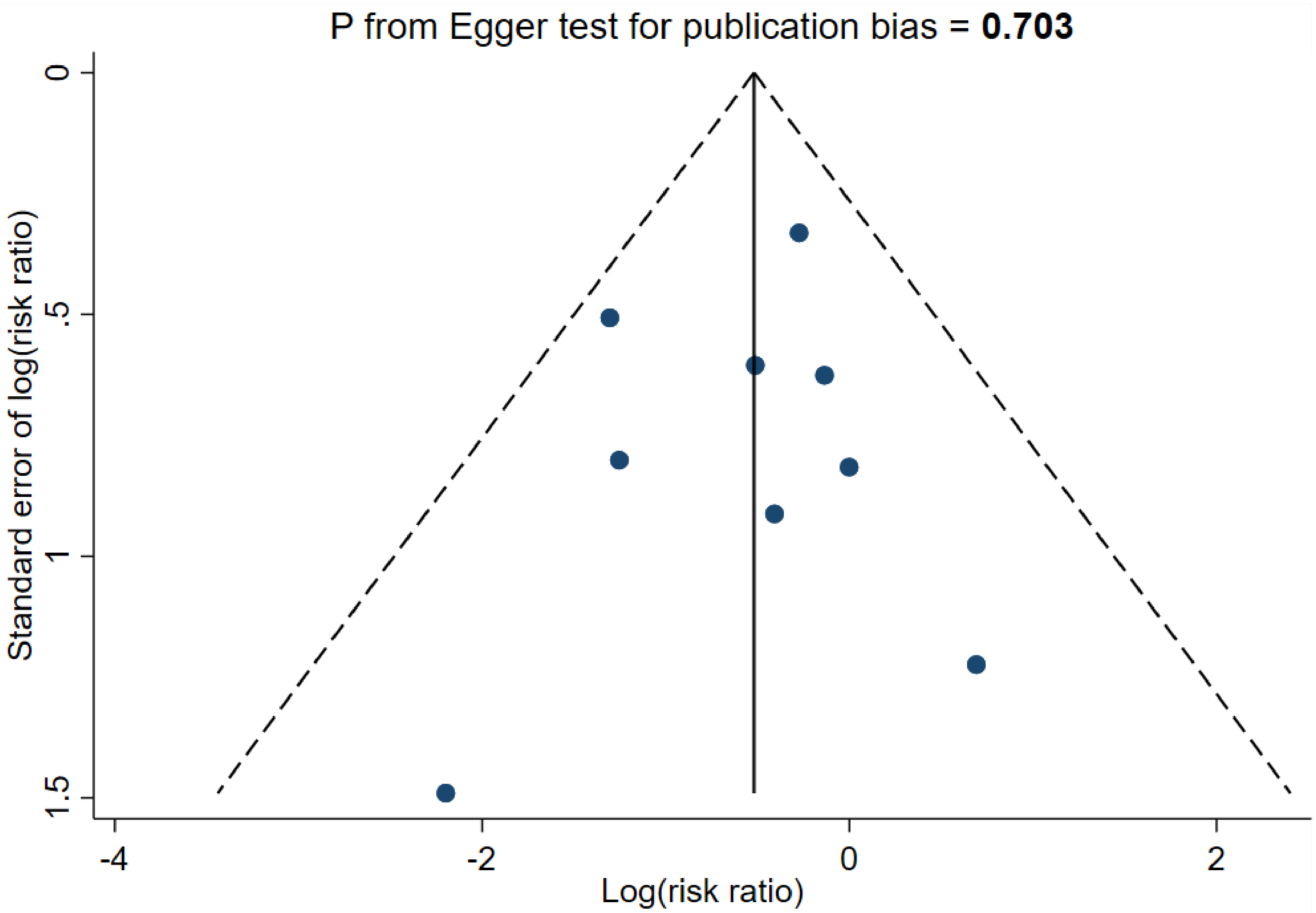

Figure S158 Funnel plot of Bradycardia

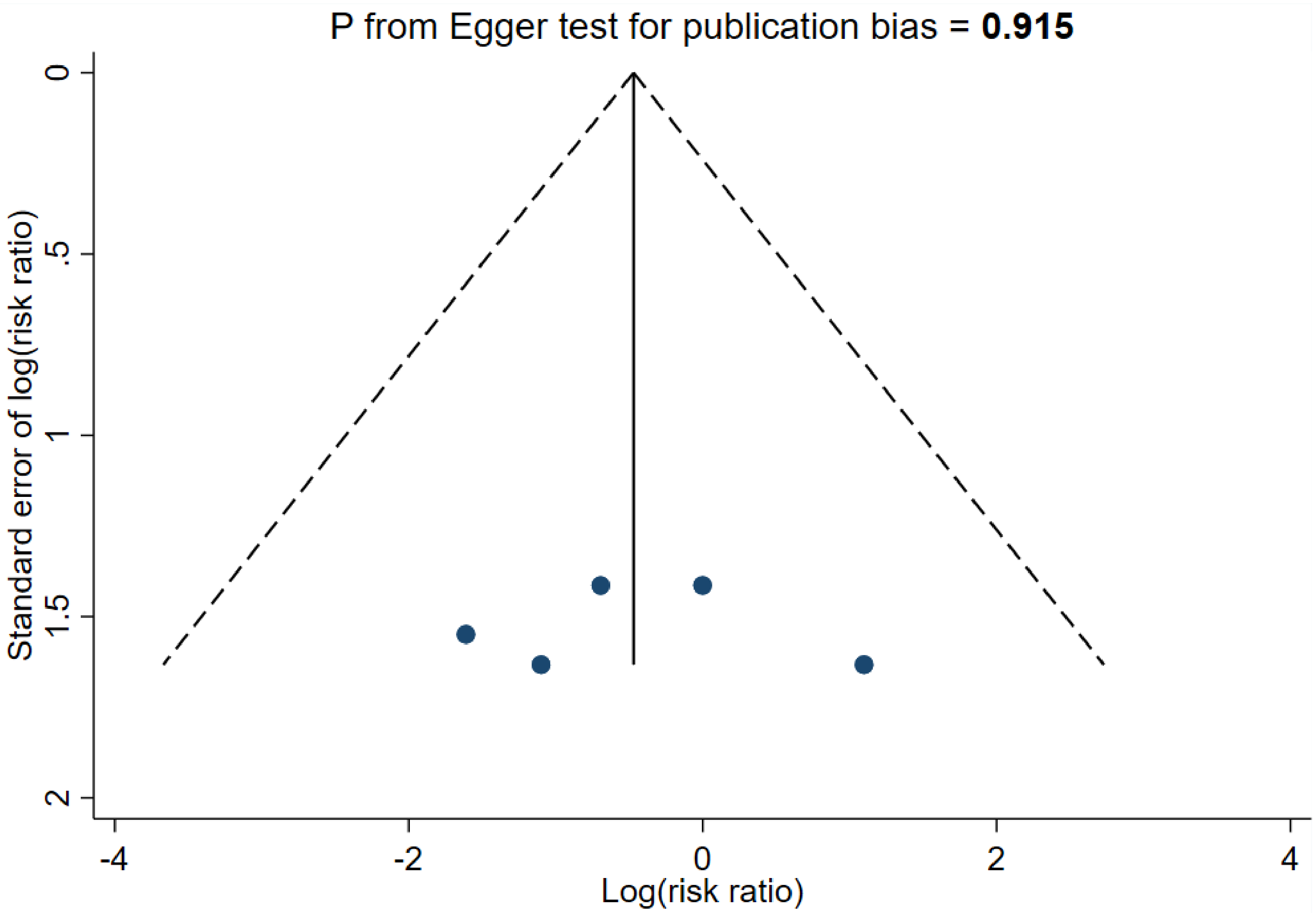

Figure S159 Funnel plot of Bundle branch block left

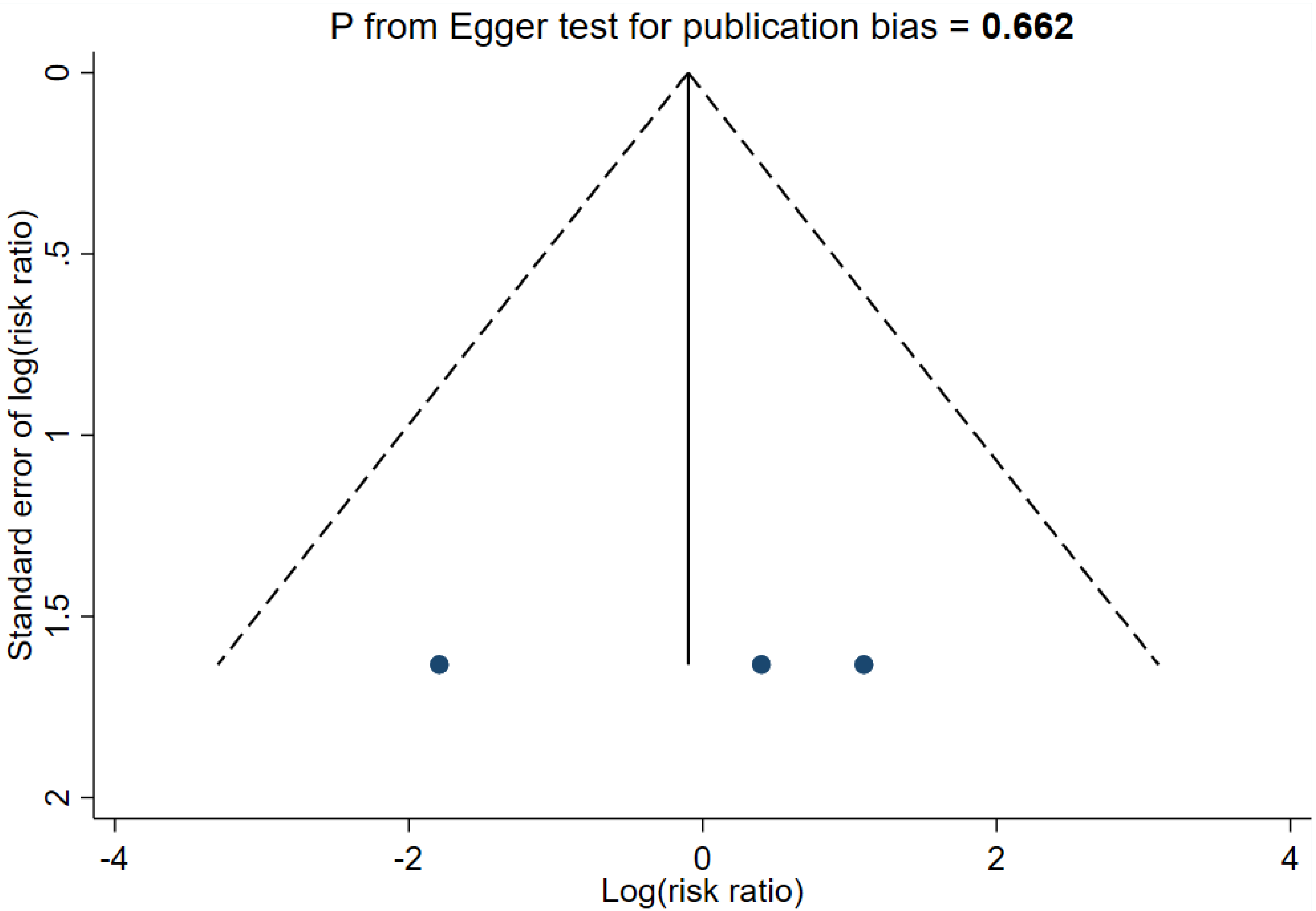

Figure S160 Funnel plot of Bundle branch block right

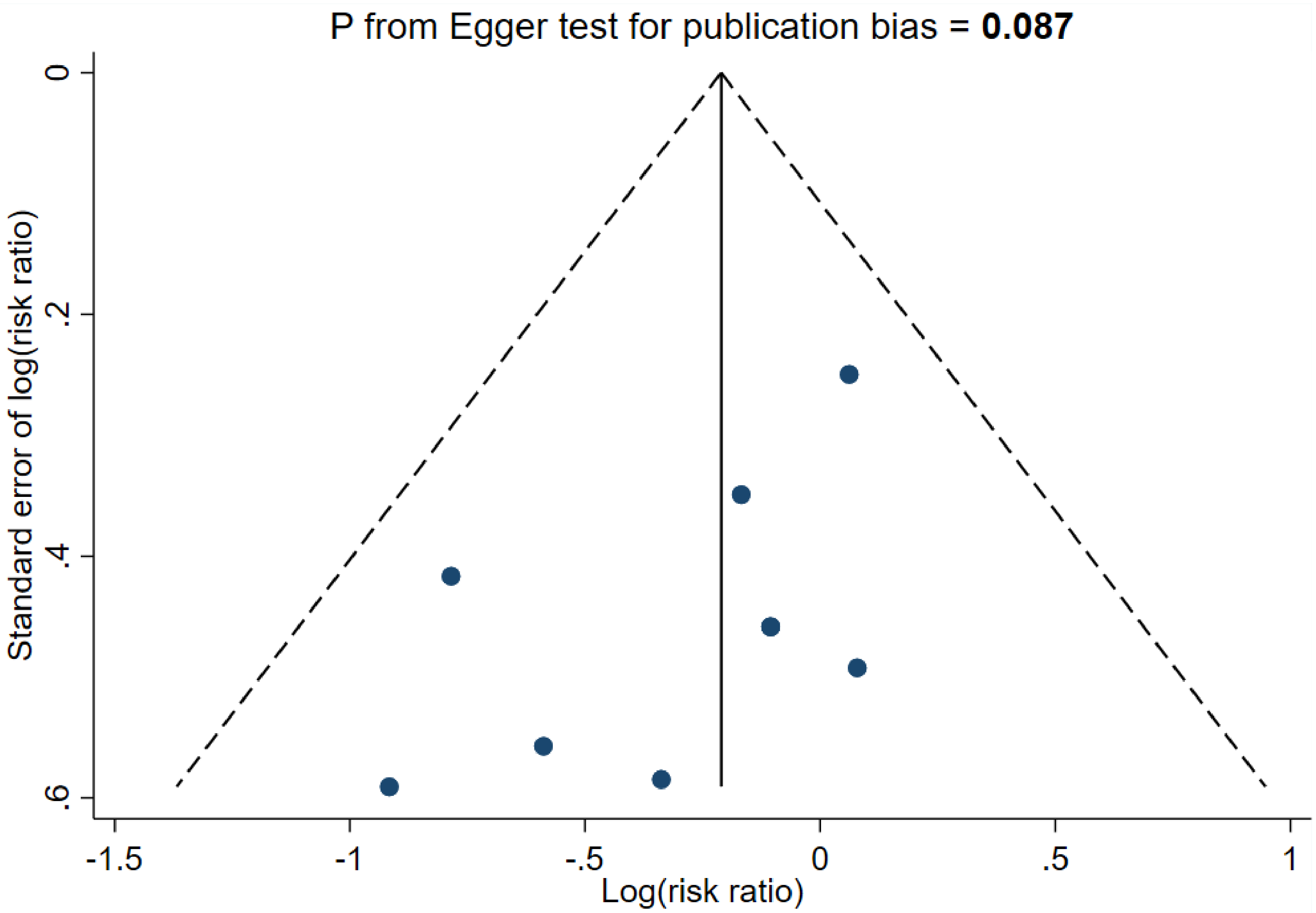

Figure S161 Funnel plot of Cardiac arrest

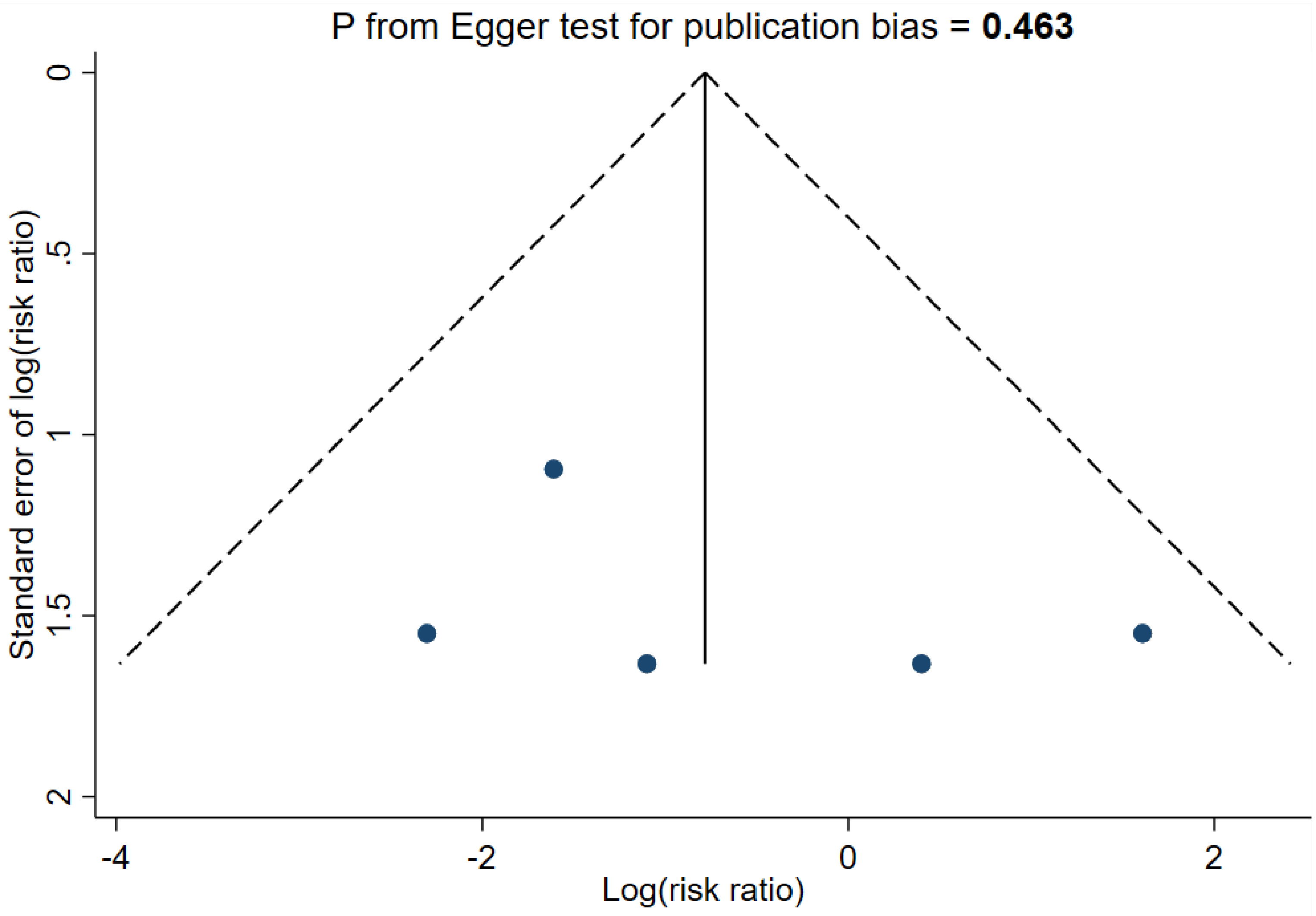

Figure S162 Funnel plot of Cardiac asthma

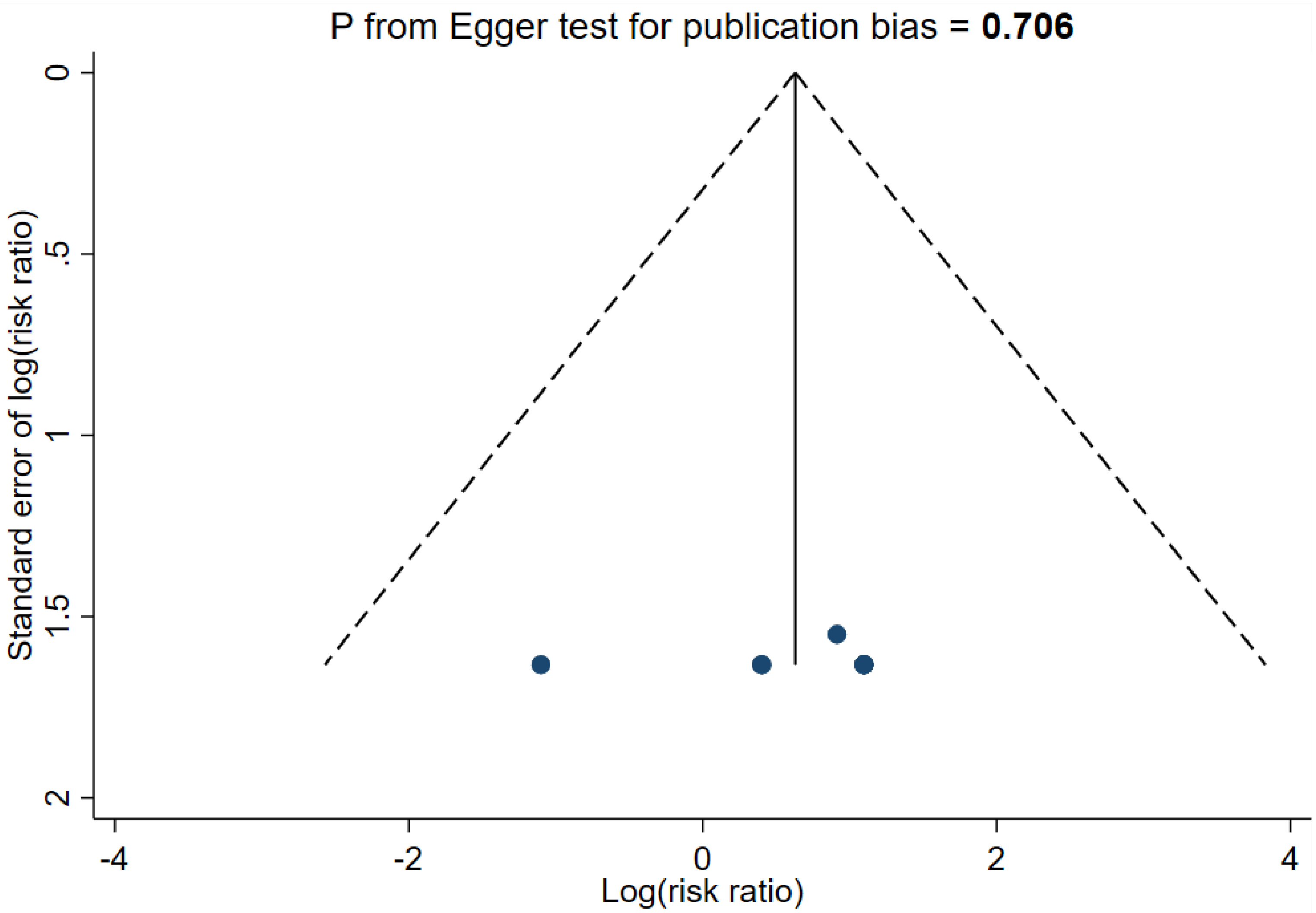

Figure S163 Funnel plot of Cardiac disorder

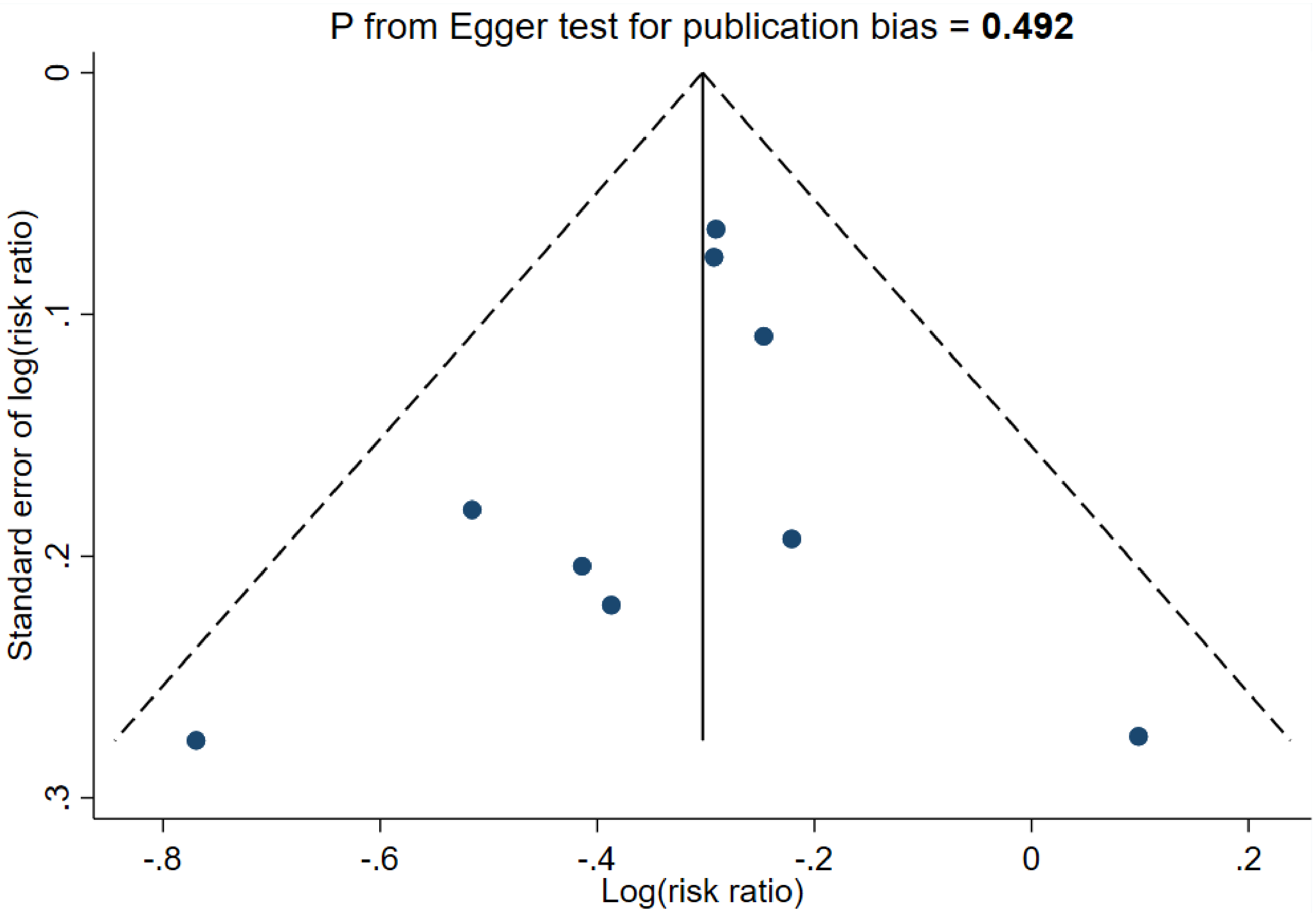

Figure S164 Funnel plot of Cardiac failure

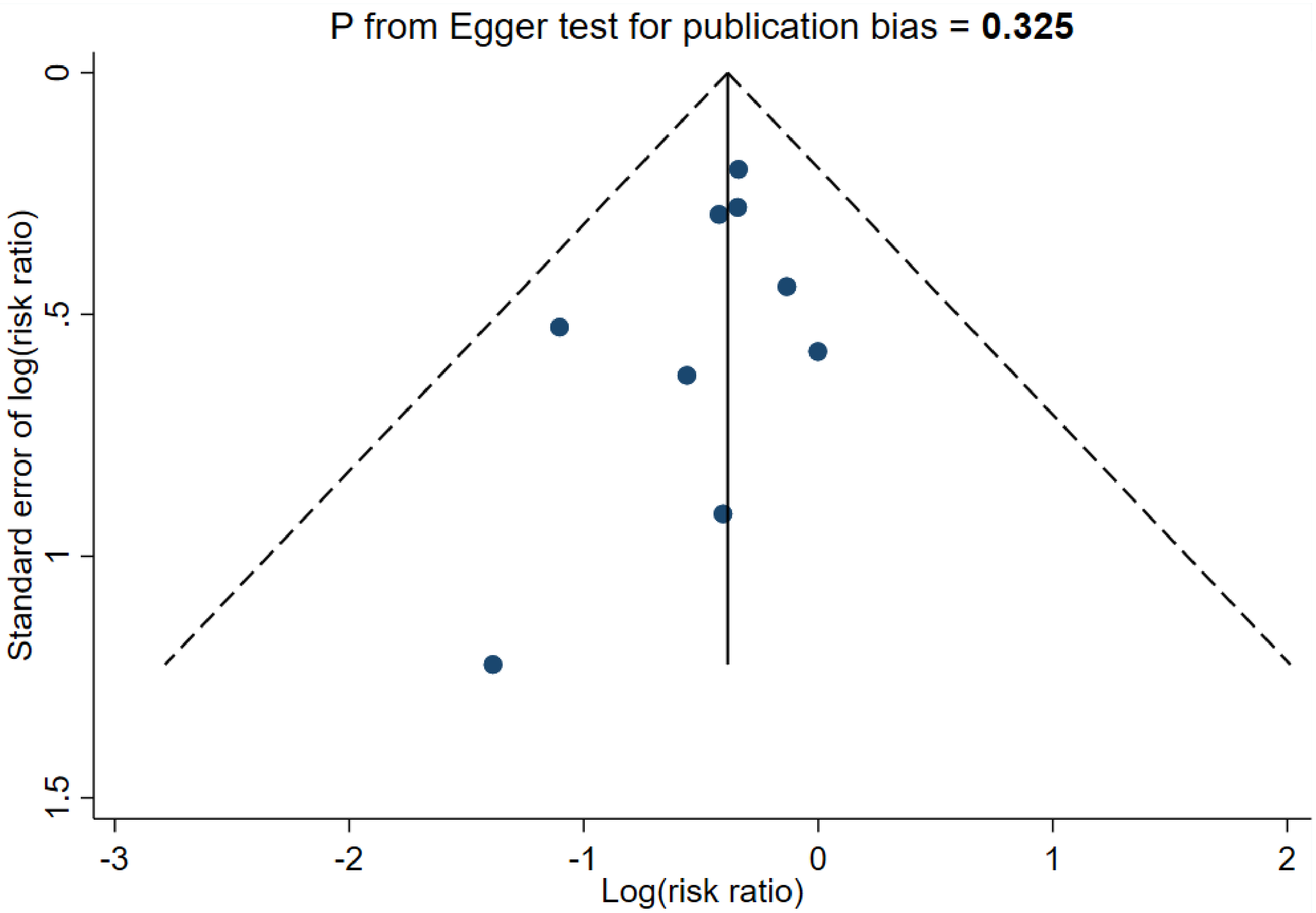

Figure S165 Funnel plot of Cardiac failure acute

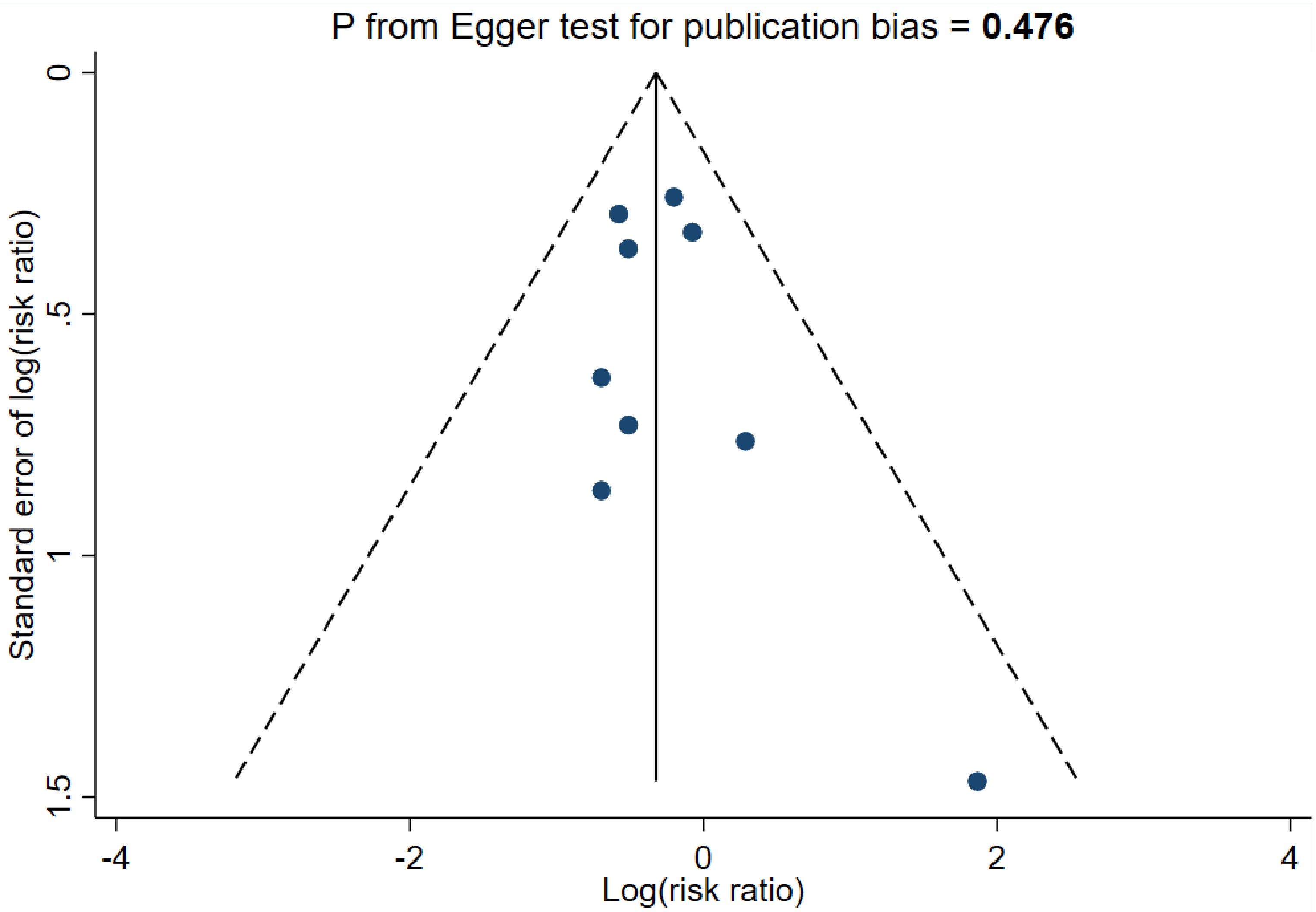

Figure S166 Funnel plot of Cardiac failure chronic

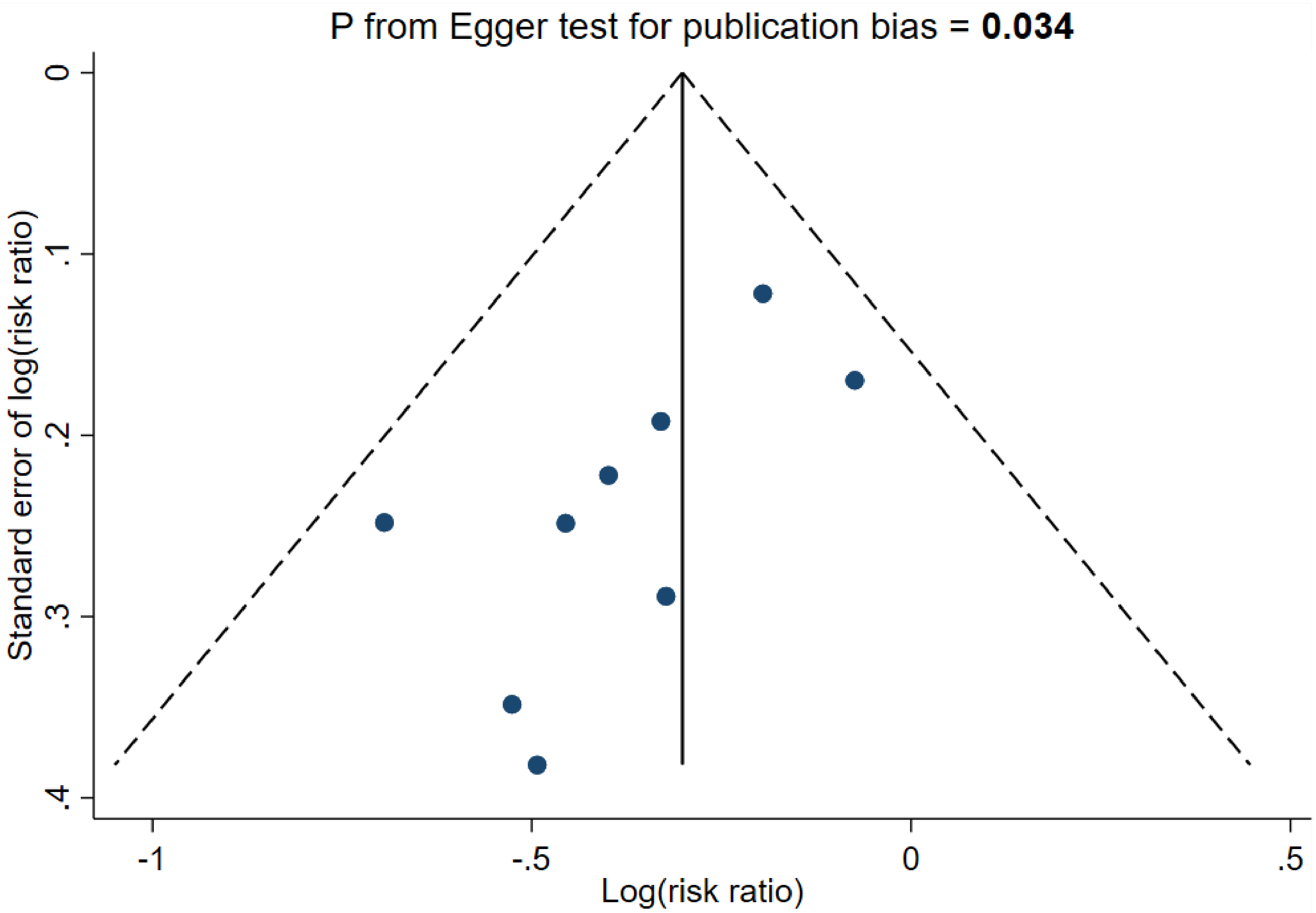

Figure S167 Funnel plot of Cardiac failure congestive

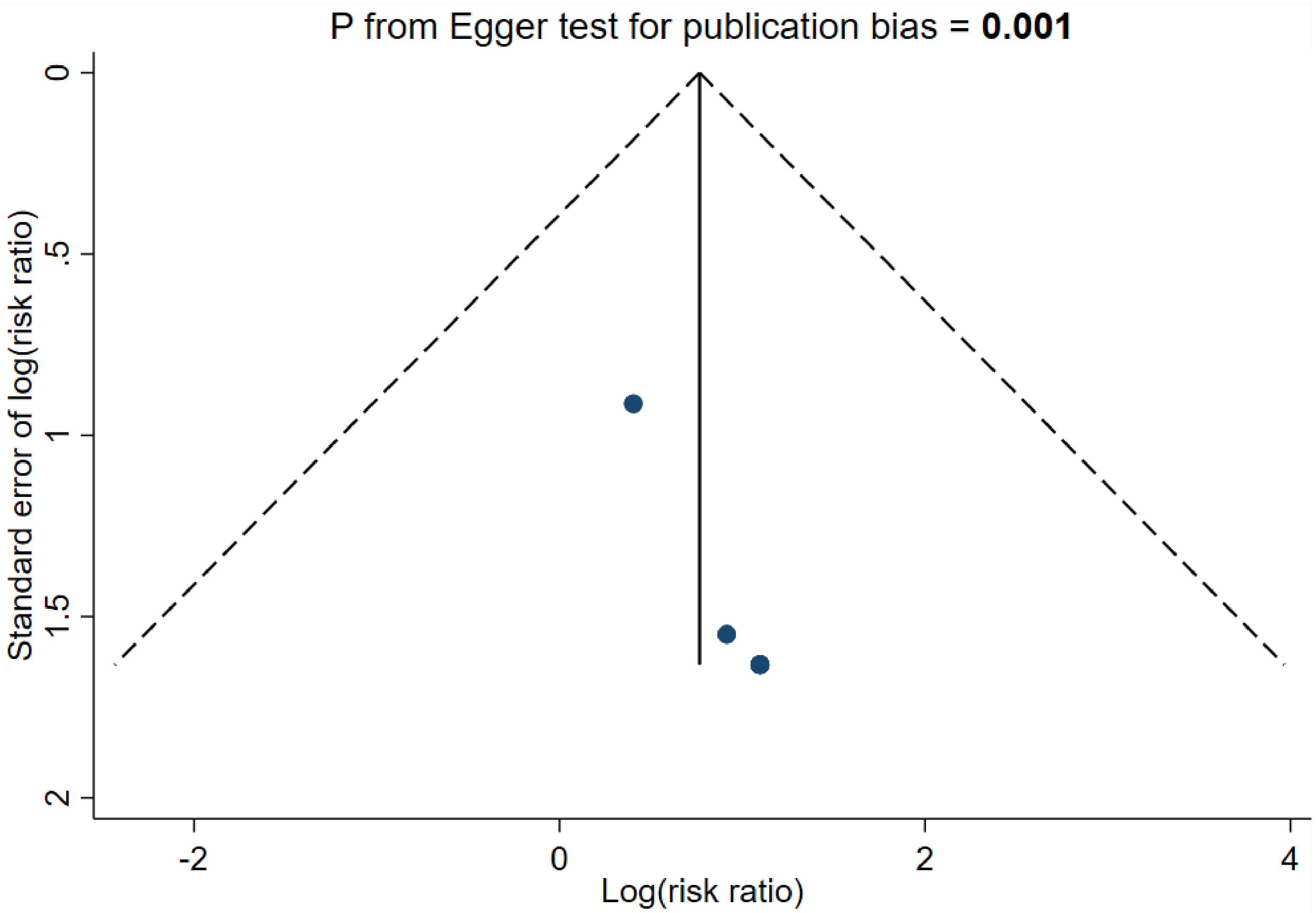

Figure S168 Funnel plot of Cardiac tamponade

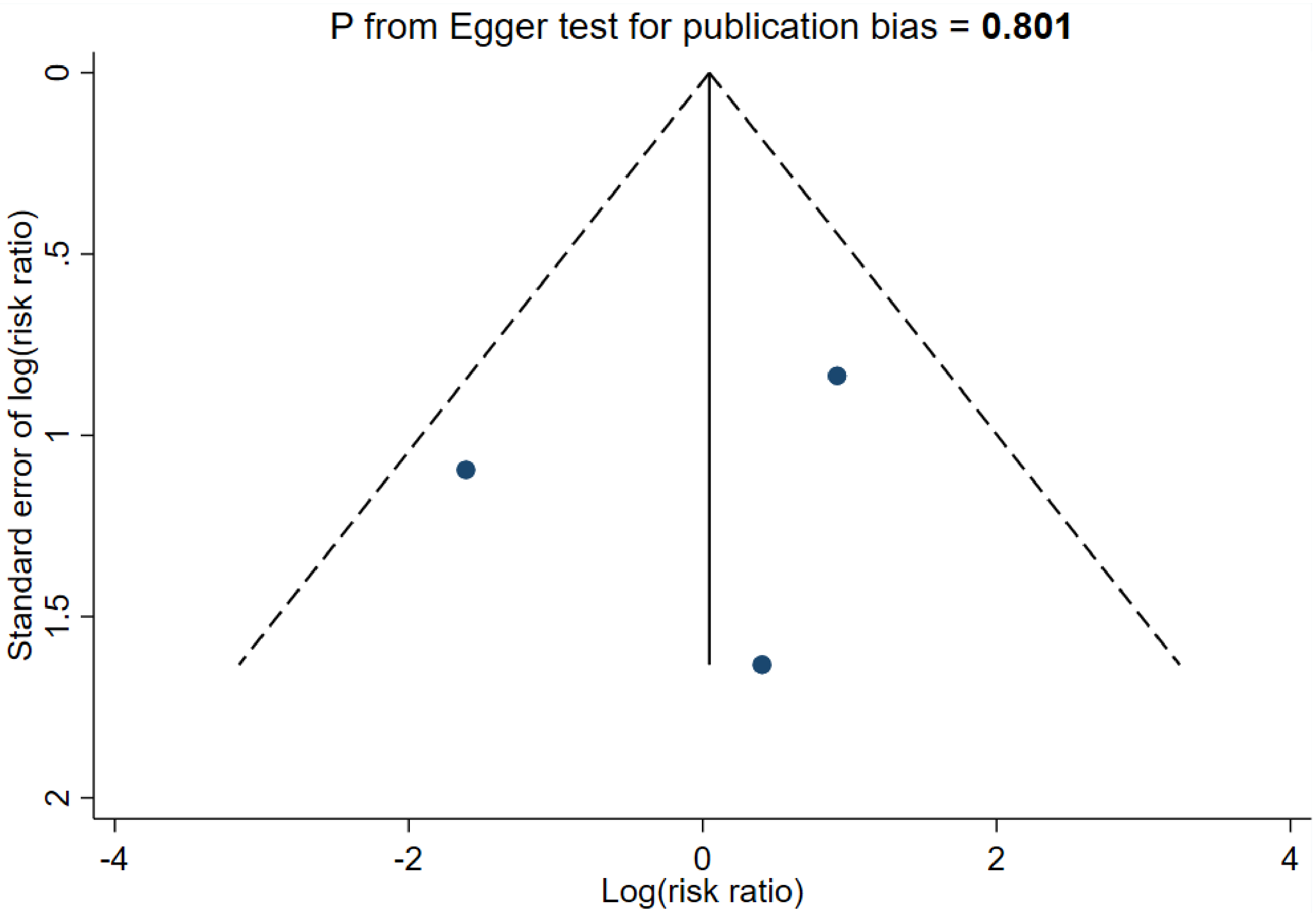

Figure S169 Funnel plot of Cardiac ventricular thrombosis

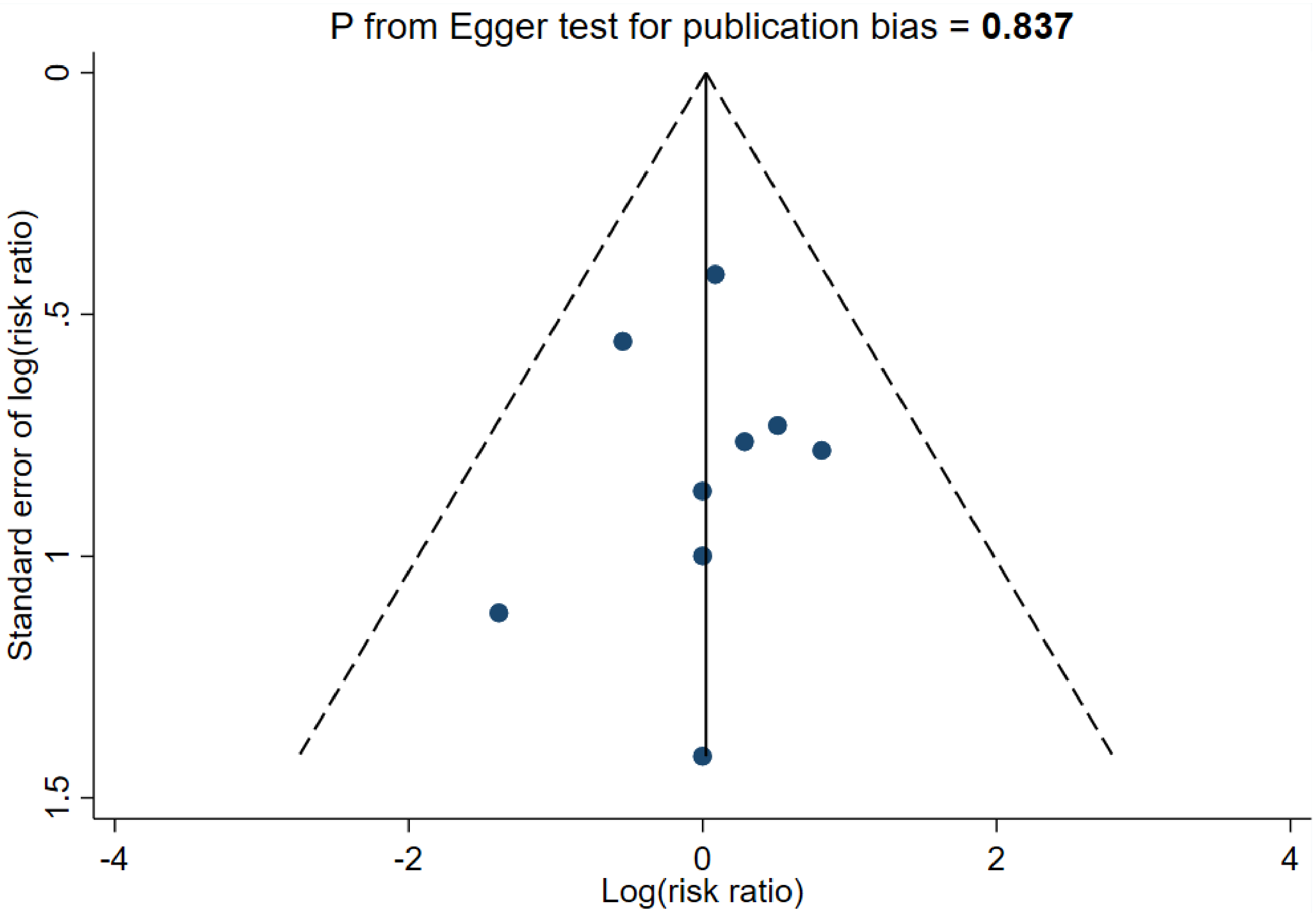

Figure S170 Funnel plot of Cardio-respiratory arrest

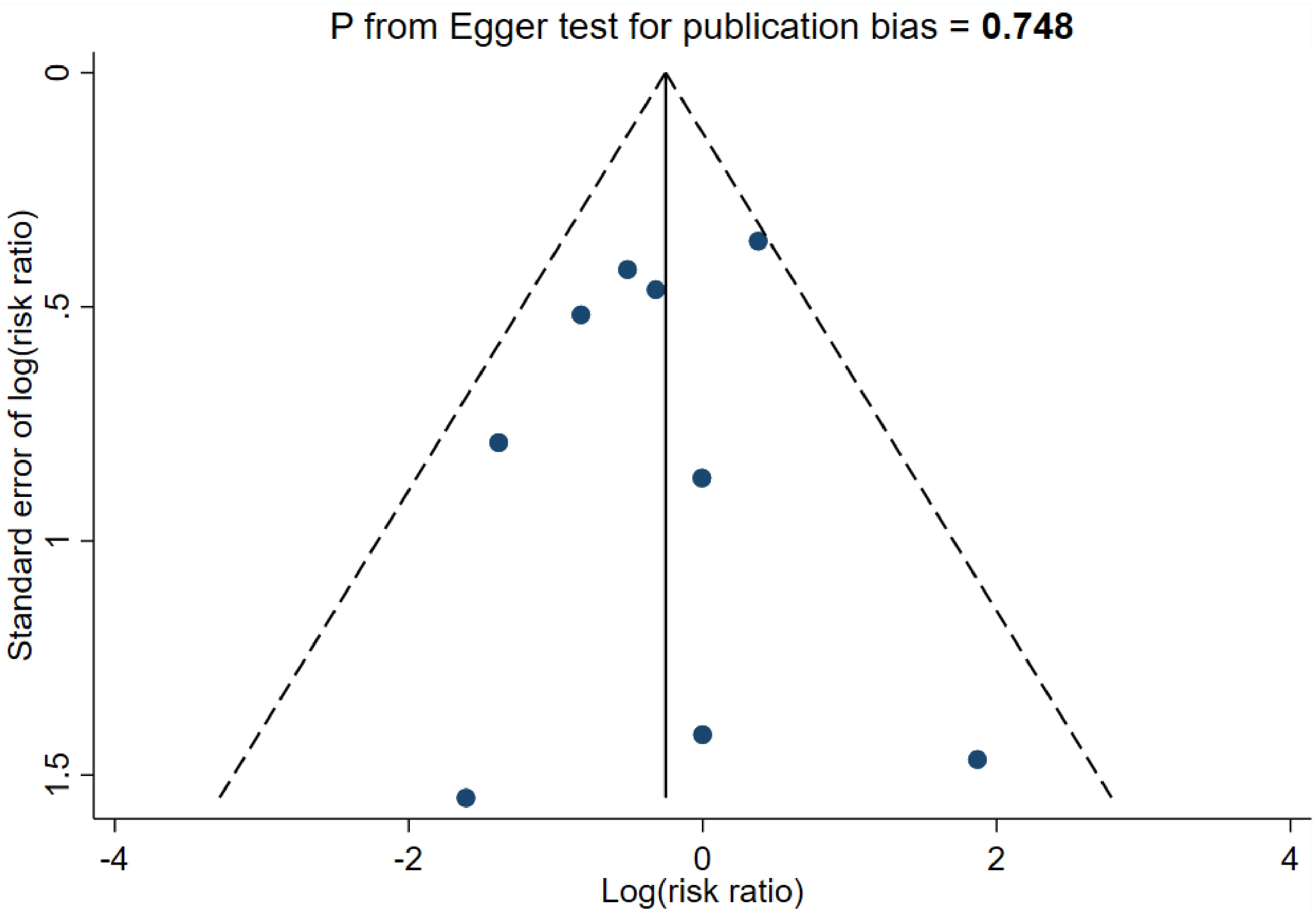

Figure S171 Funnel plot of Cardiogenic shock

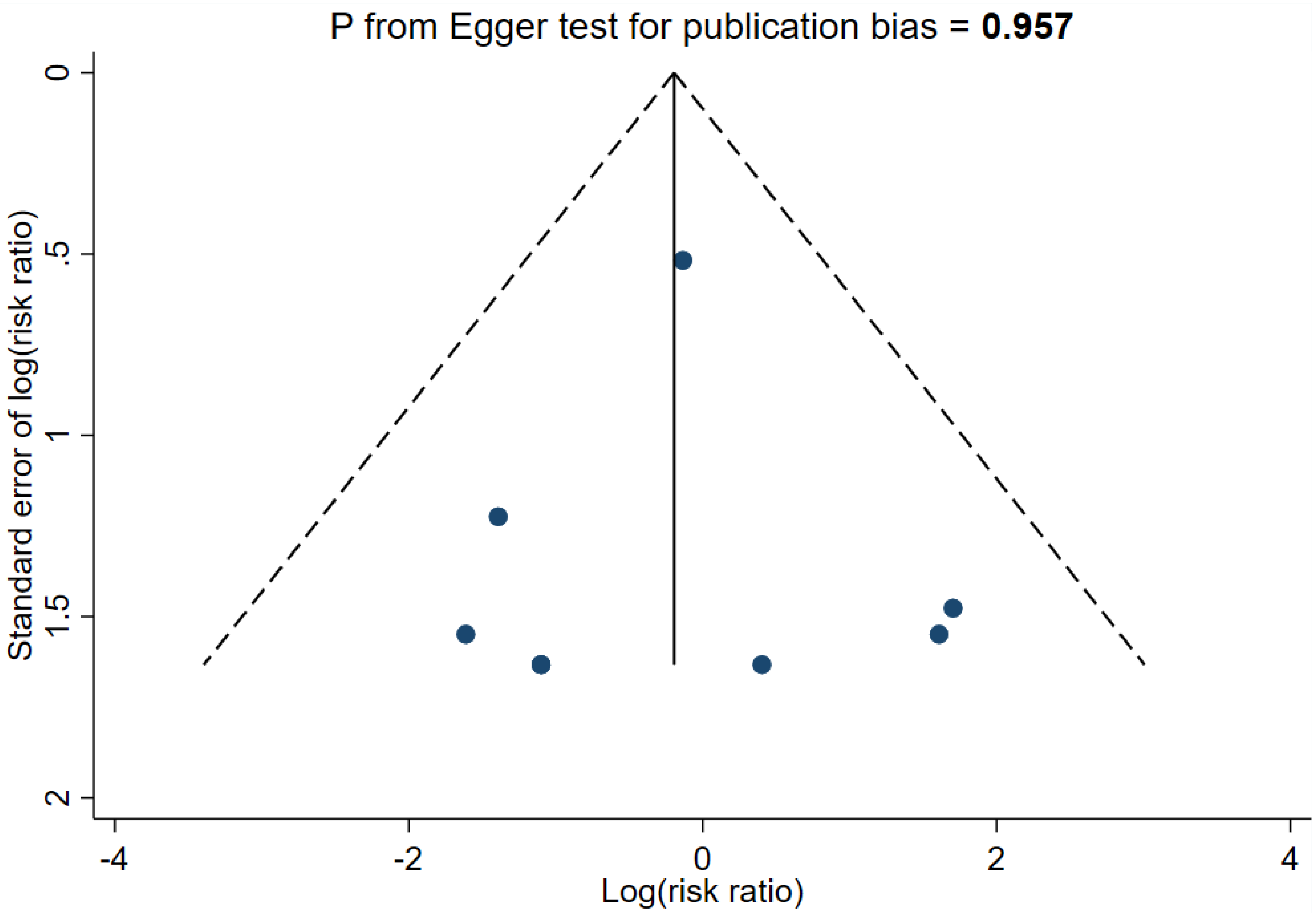

Figure S172 Funnel plot of Cardiomyopathy

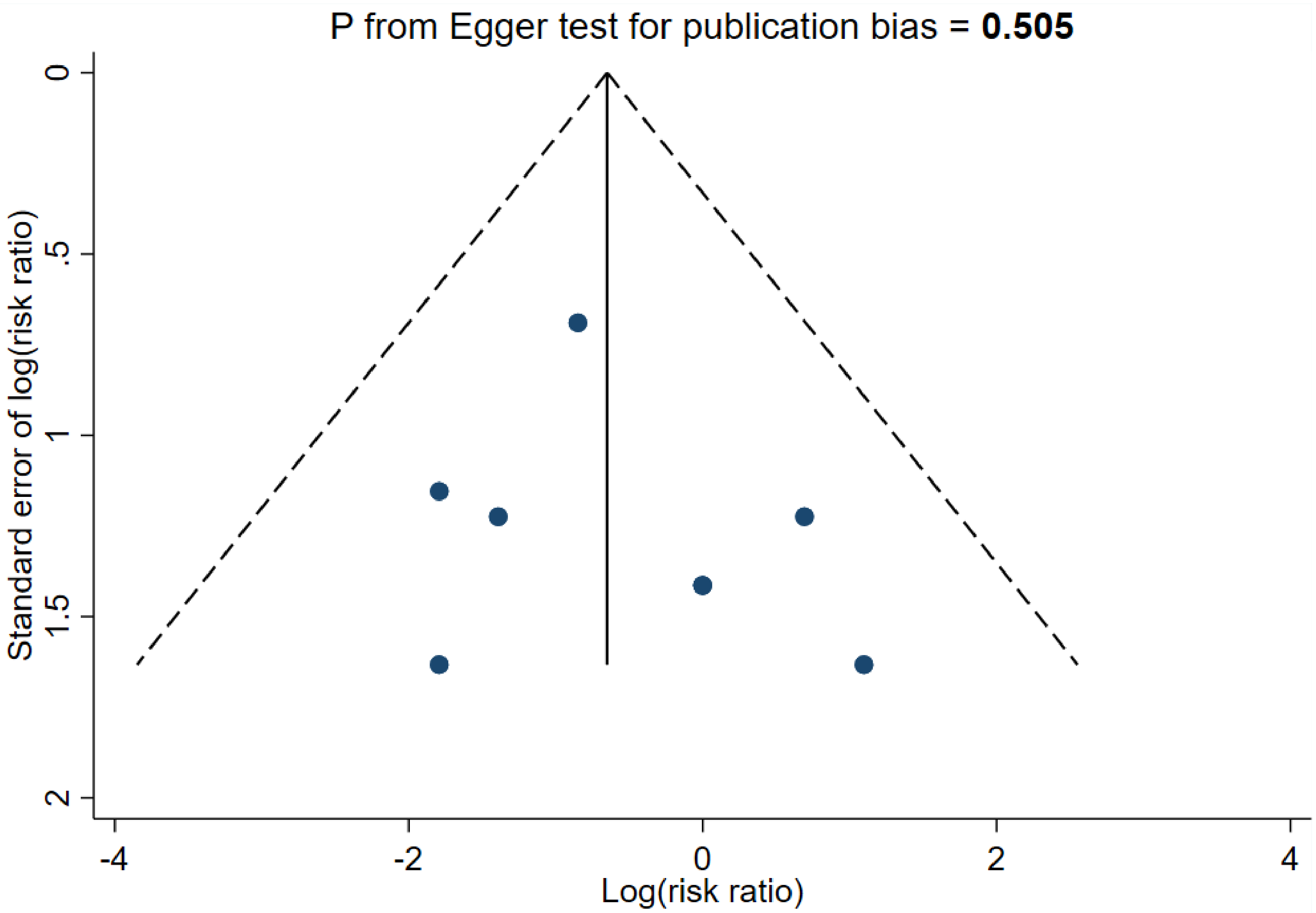

Figure S173 Funnel plot of Cardiopulmonary failure

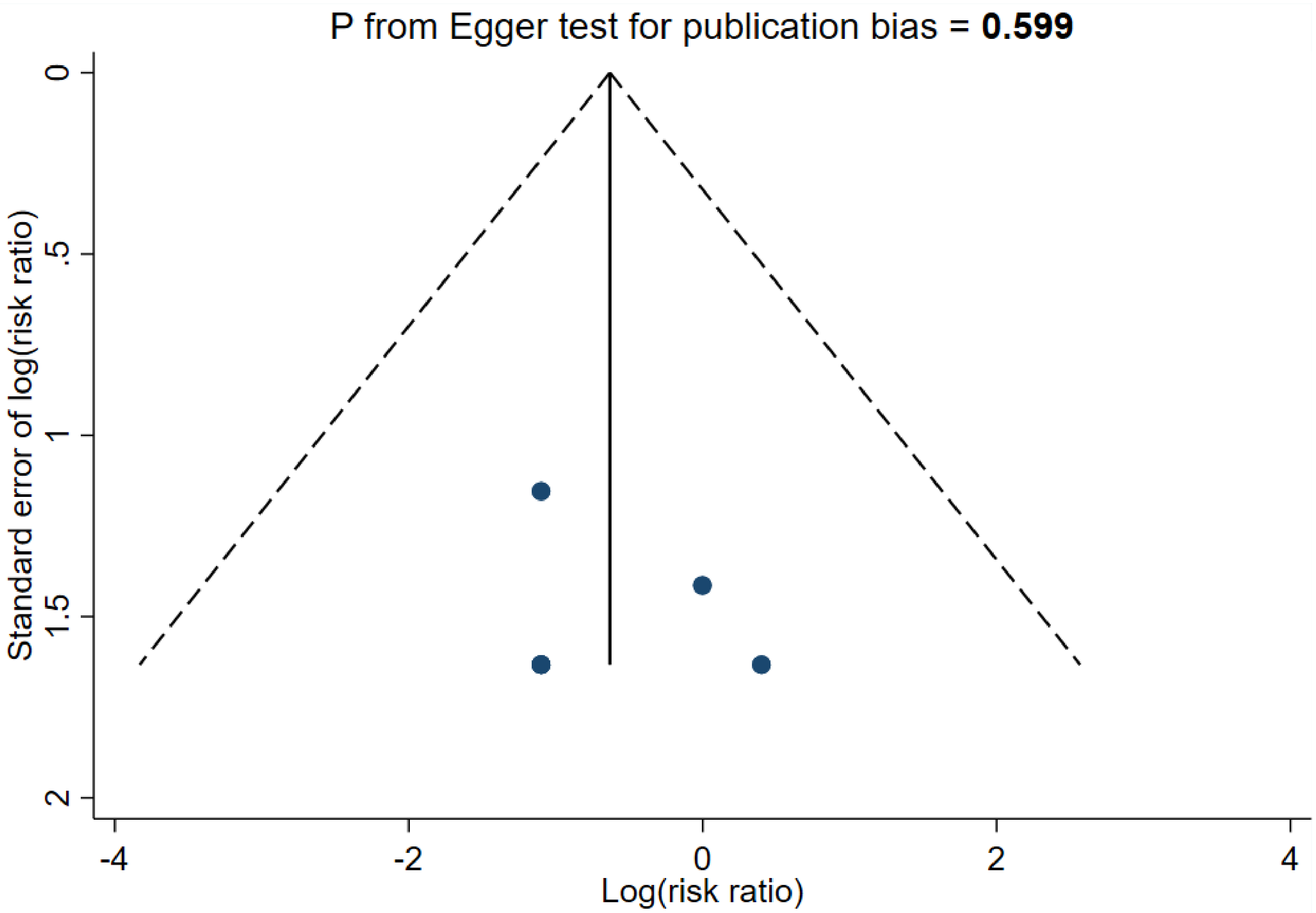

Figure S174 Funnel plot of Cardiorenal syndrome

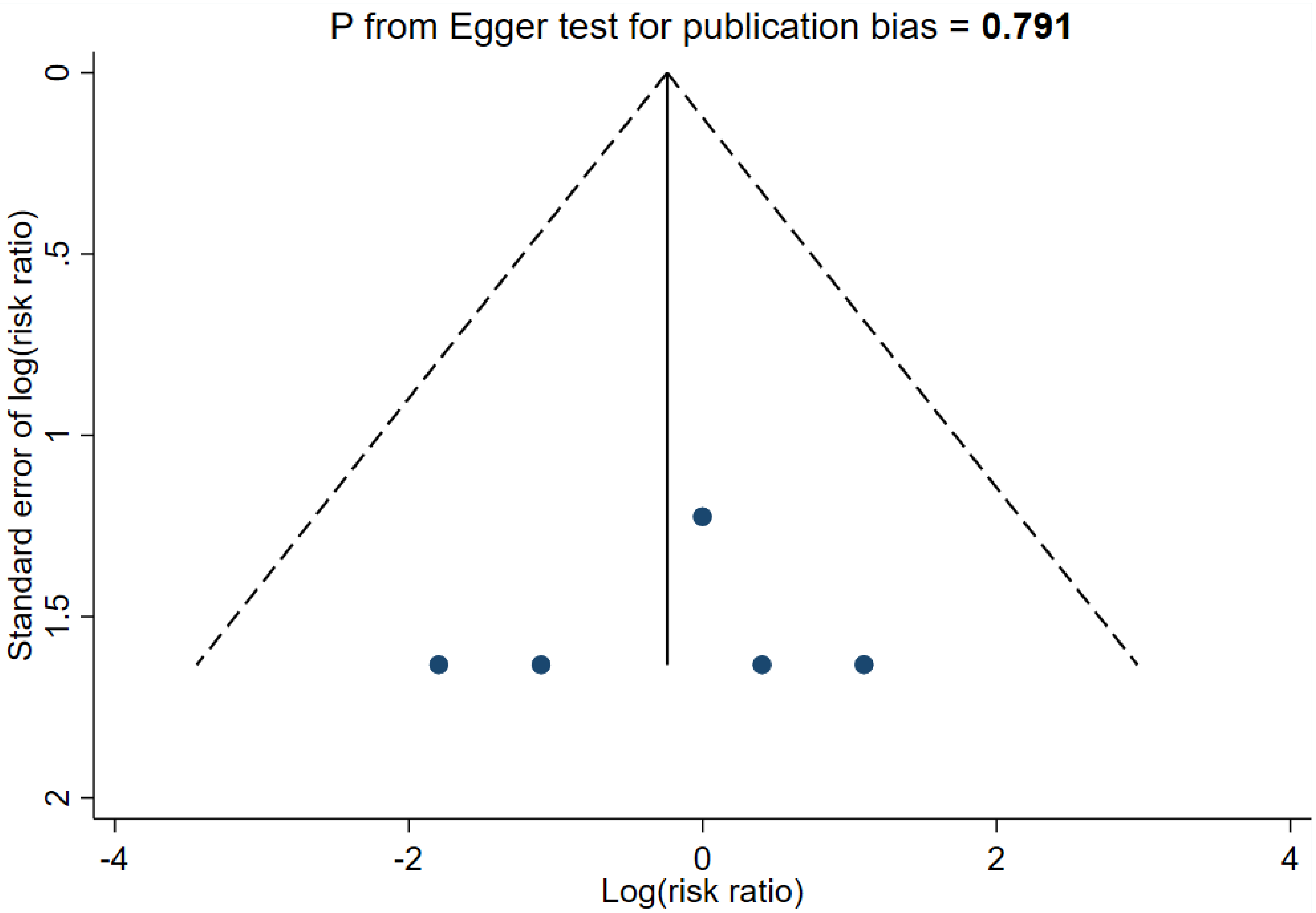

Figure S175 Funnel plot of Cardiovascular disorder

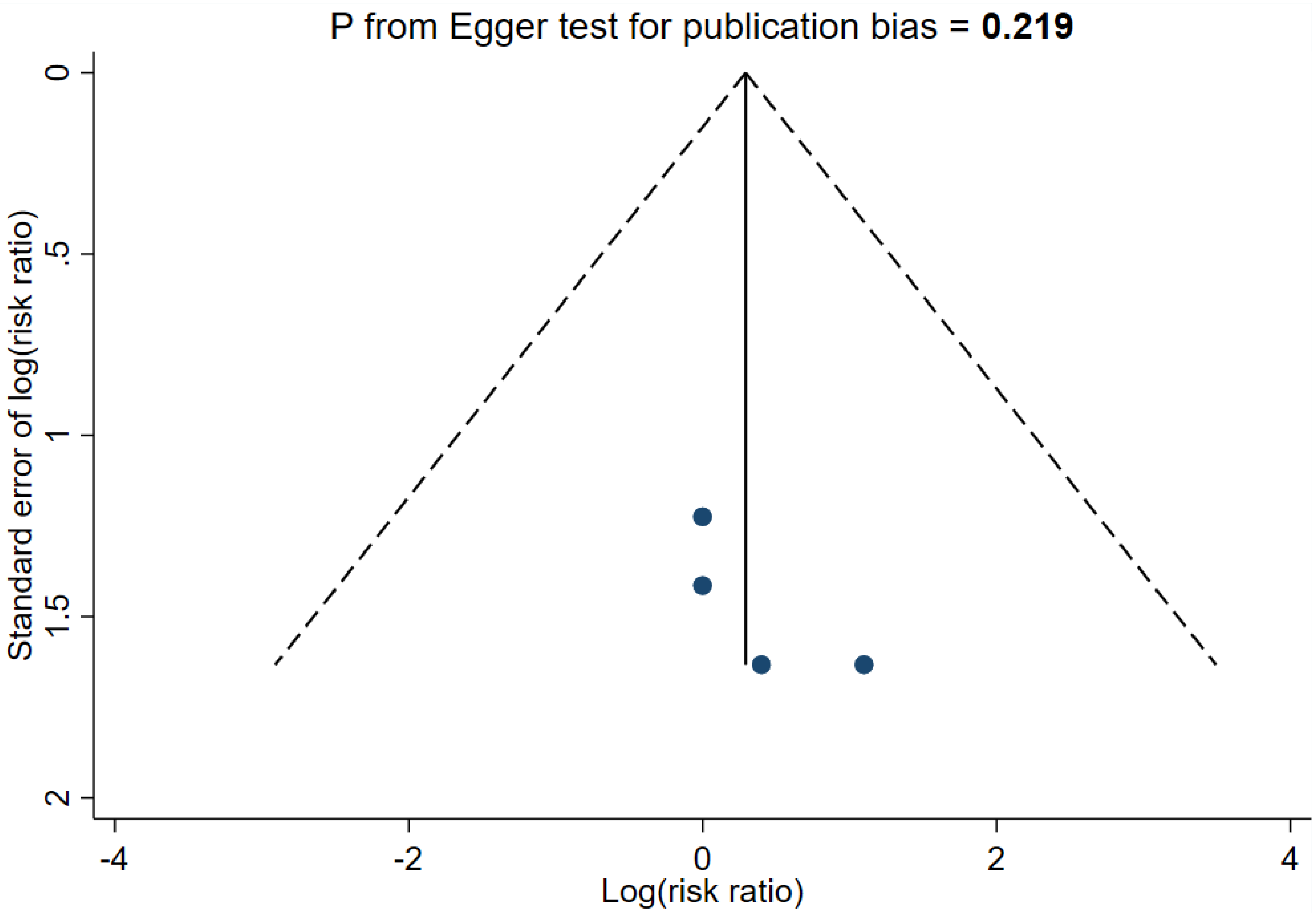

Figure S176 Funnel plot of Cardiovascular insufficiency

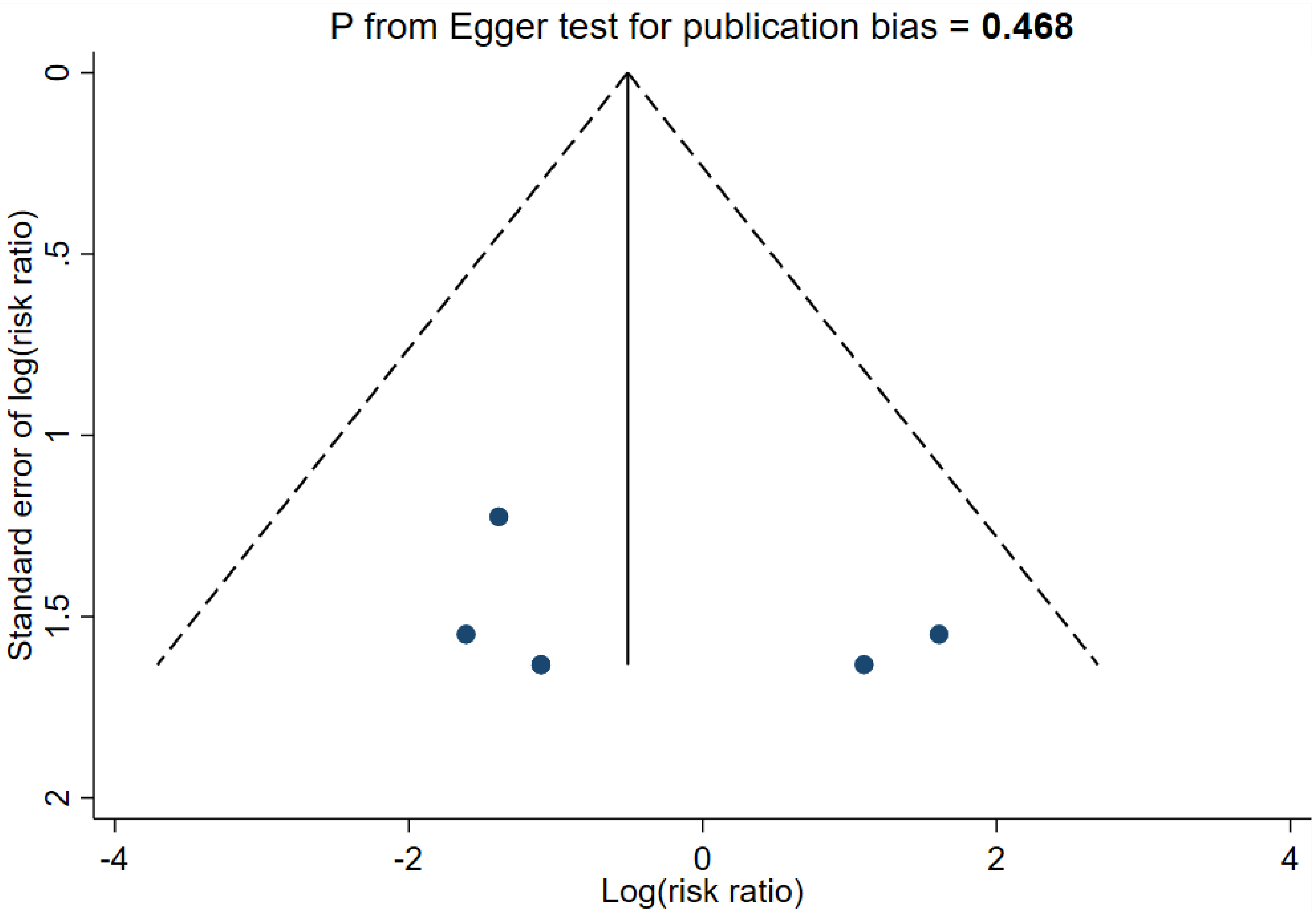

Figure S177 Funnel plot of Chronic left ventricular failure

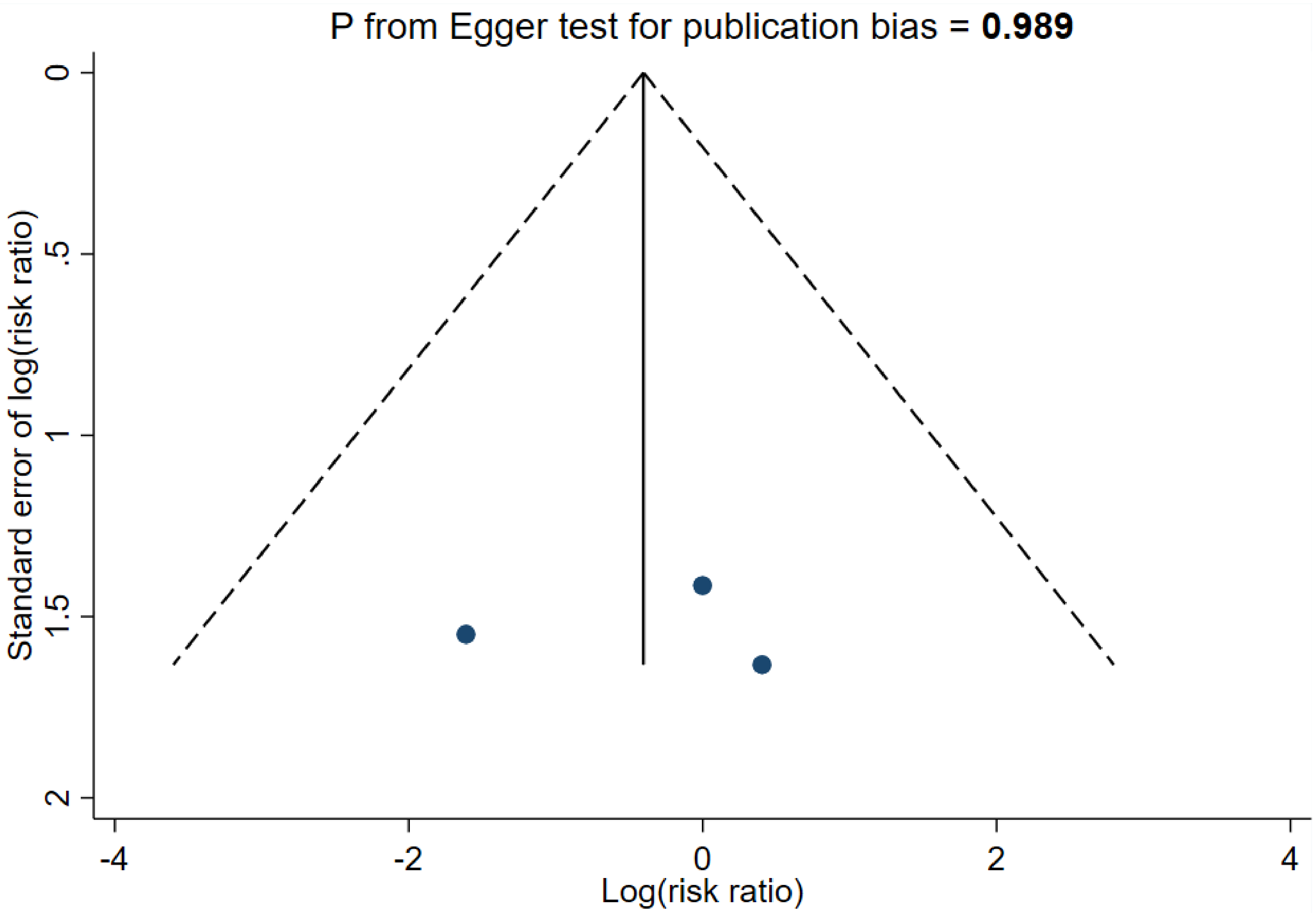

Figure S178 Funnel plot of Conduction disorder

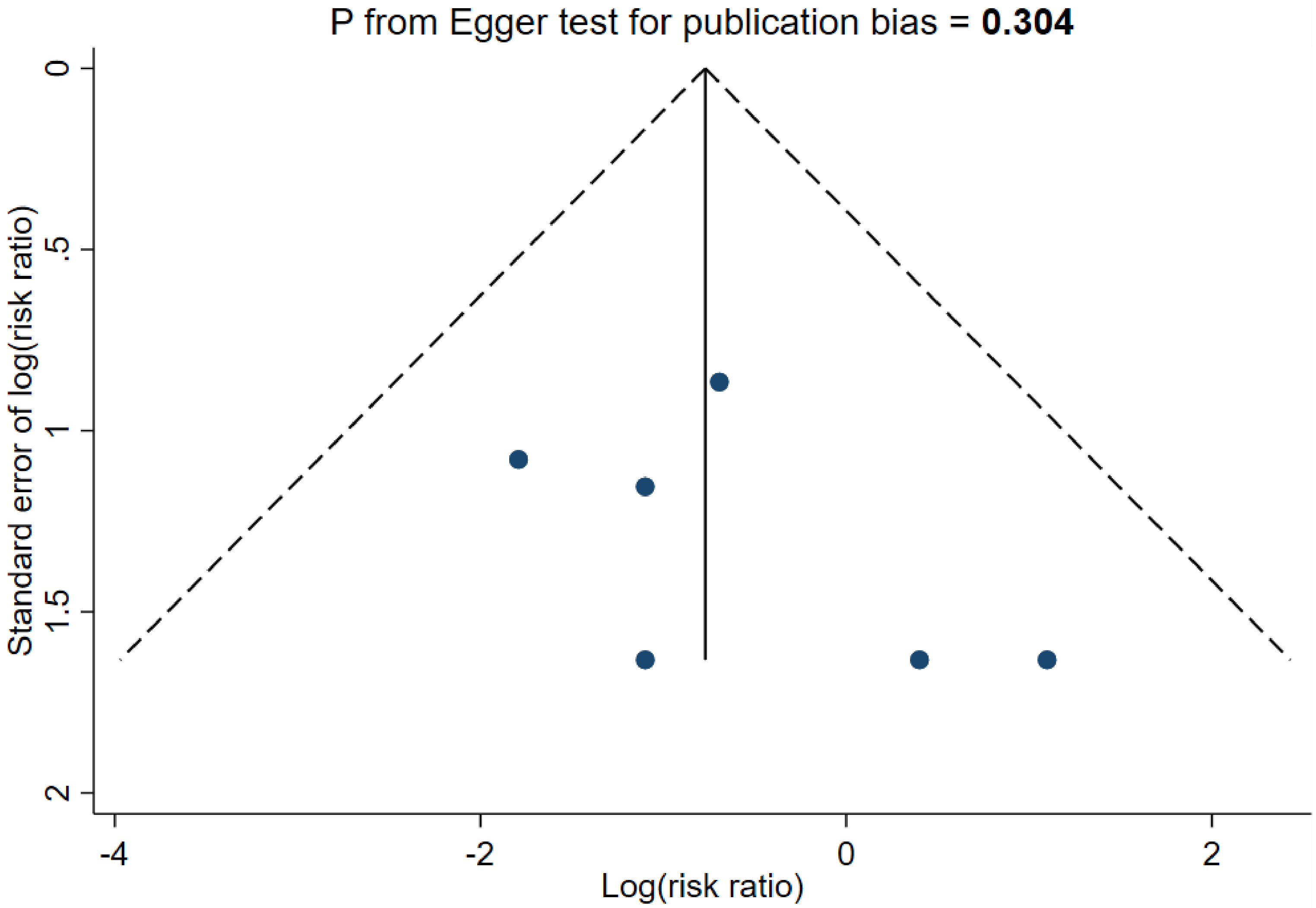

Figure S179 Funnel plot of Congestive cardiomyopathy

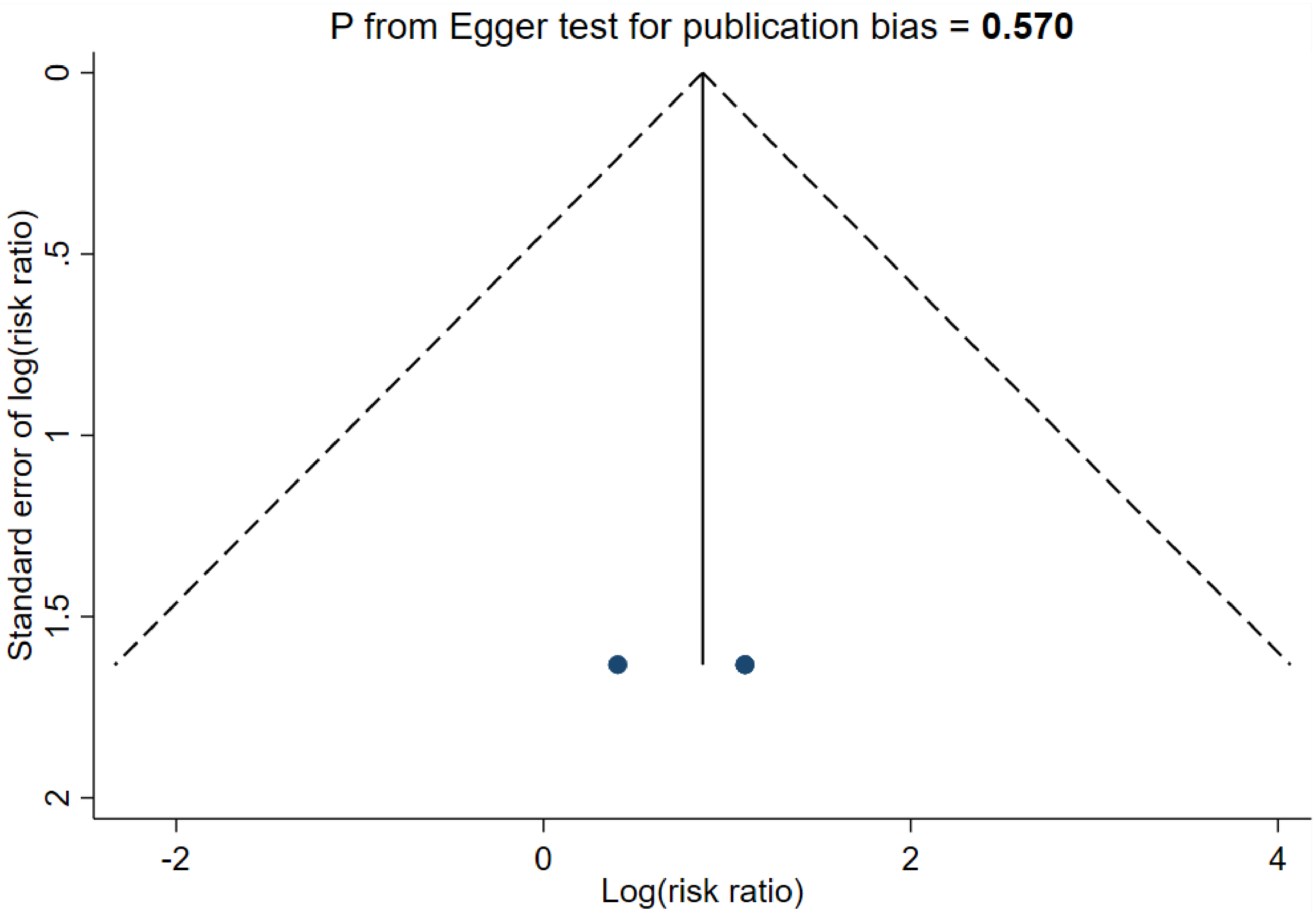

Figure S180 Funnel plot of Cor pulmonale

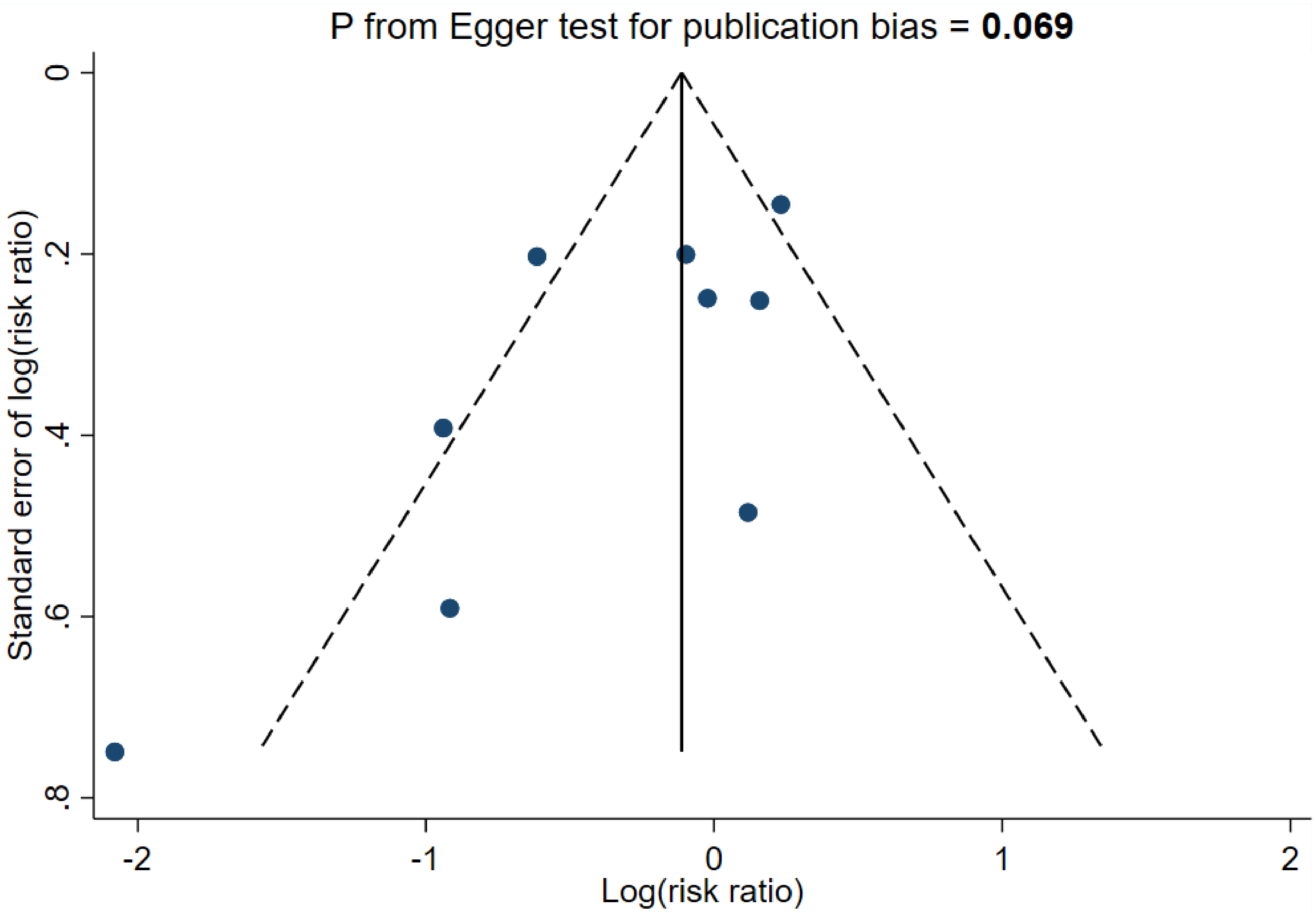

Figure S181 Funnel plot of Coronary artery disease

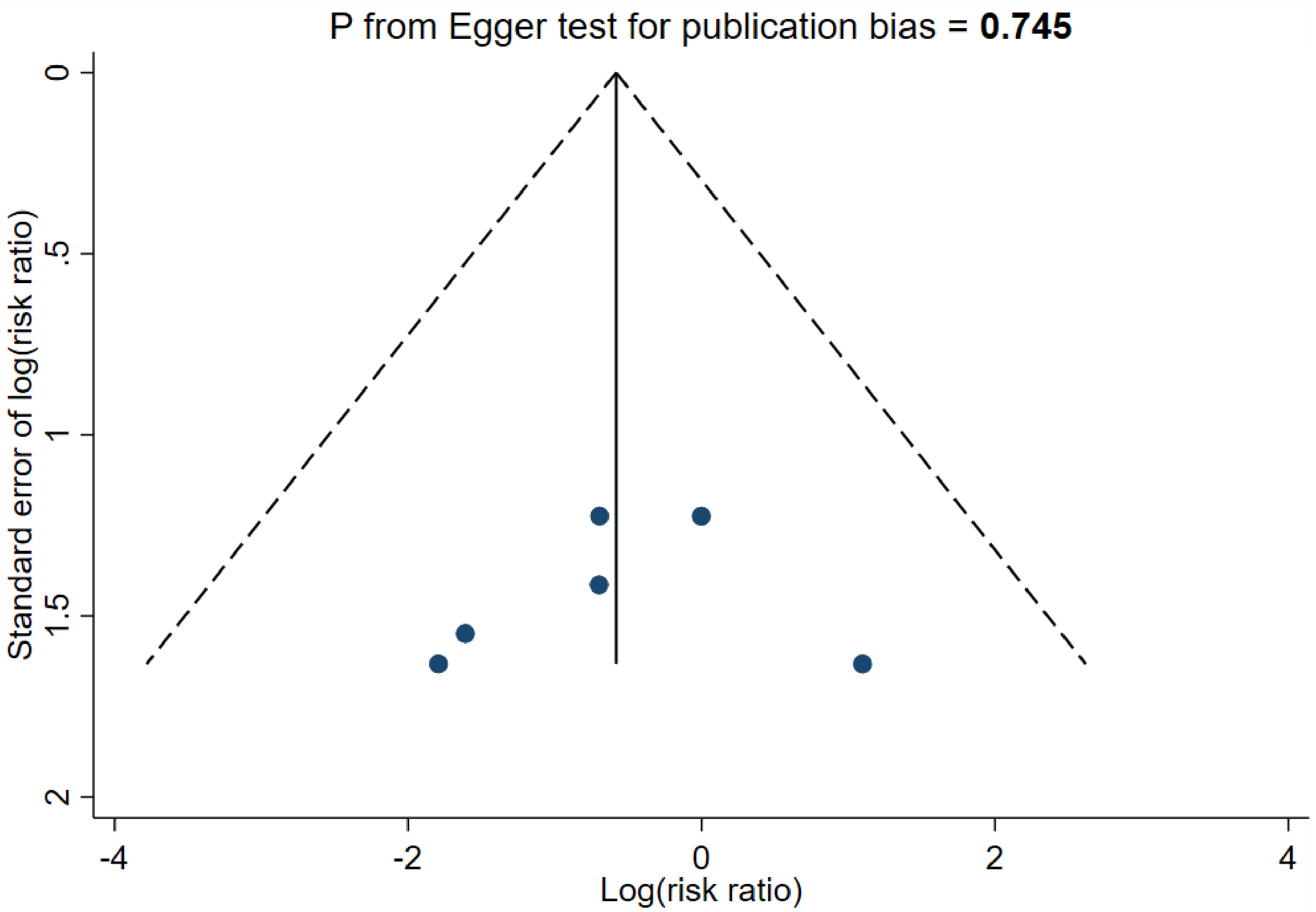

Figure S182 Funnel plot of Coronary artery insufficiency

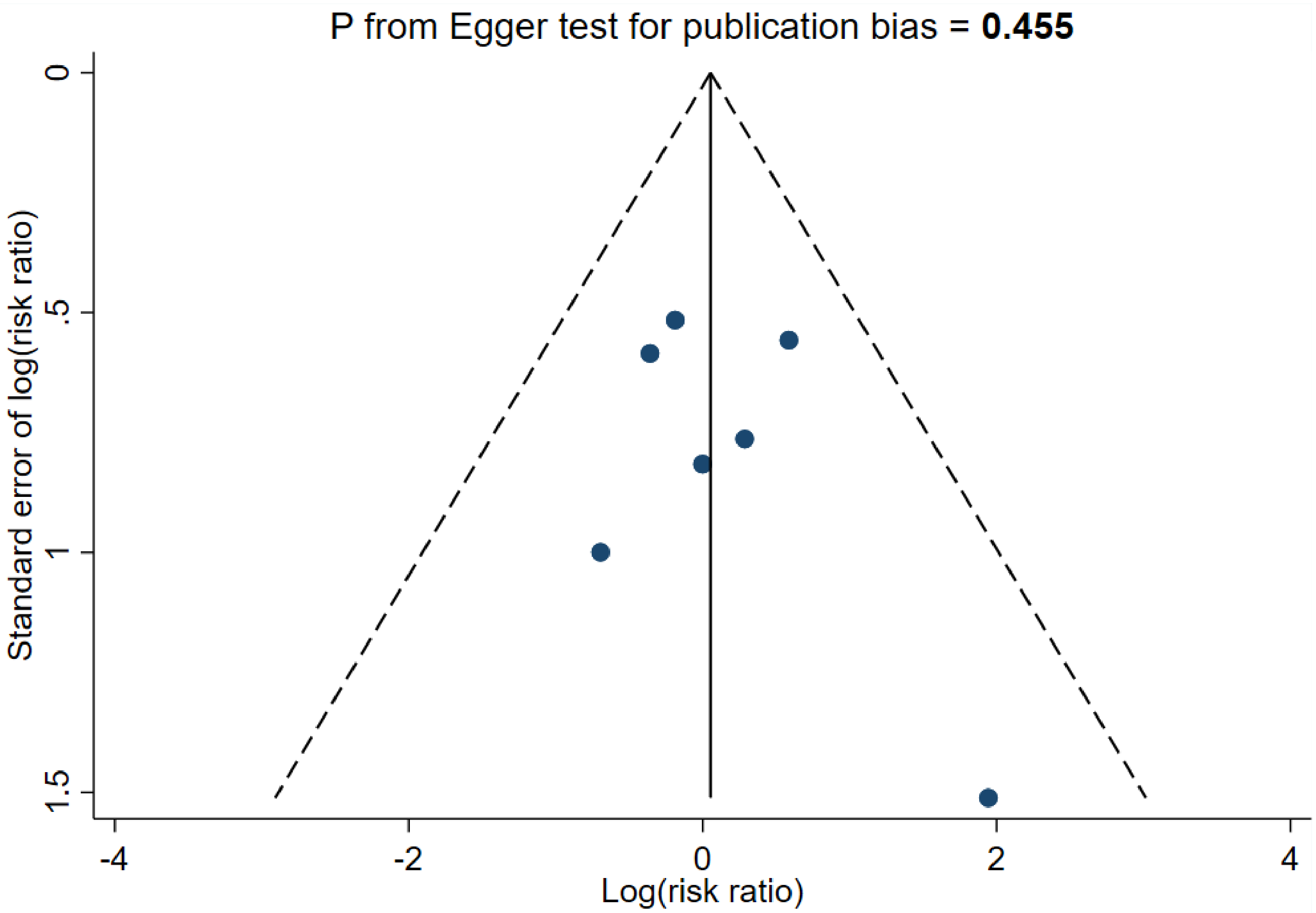

Figure S183 Funnel plot of Coronary artery occlusion

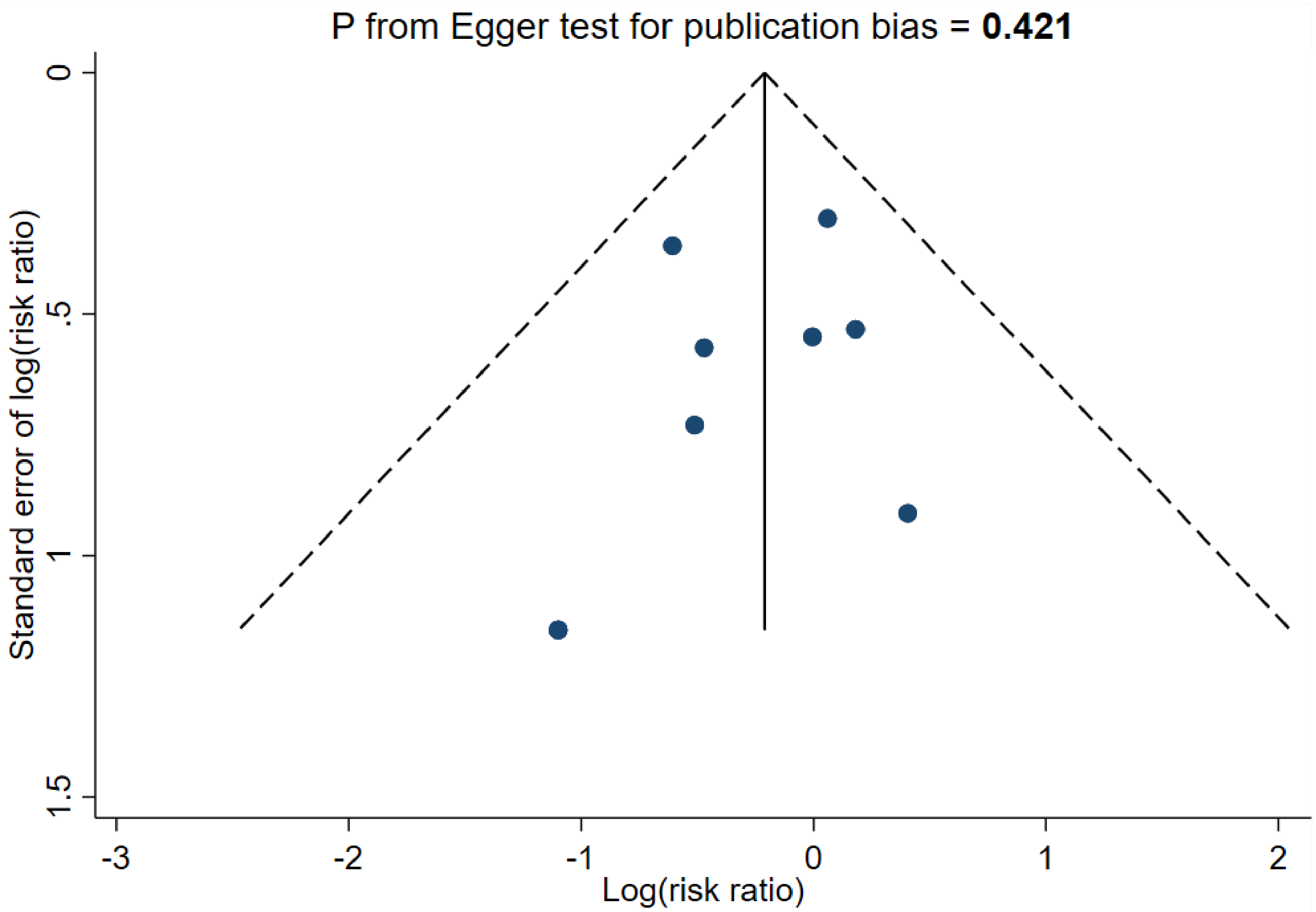

Figure S184 Funnel plot of Coronary artery stenosis

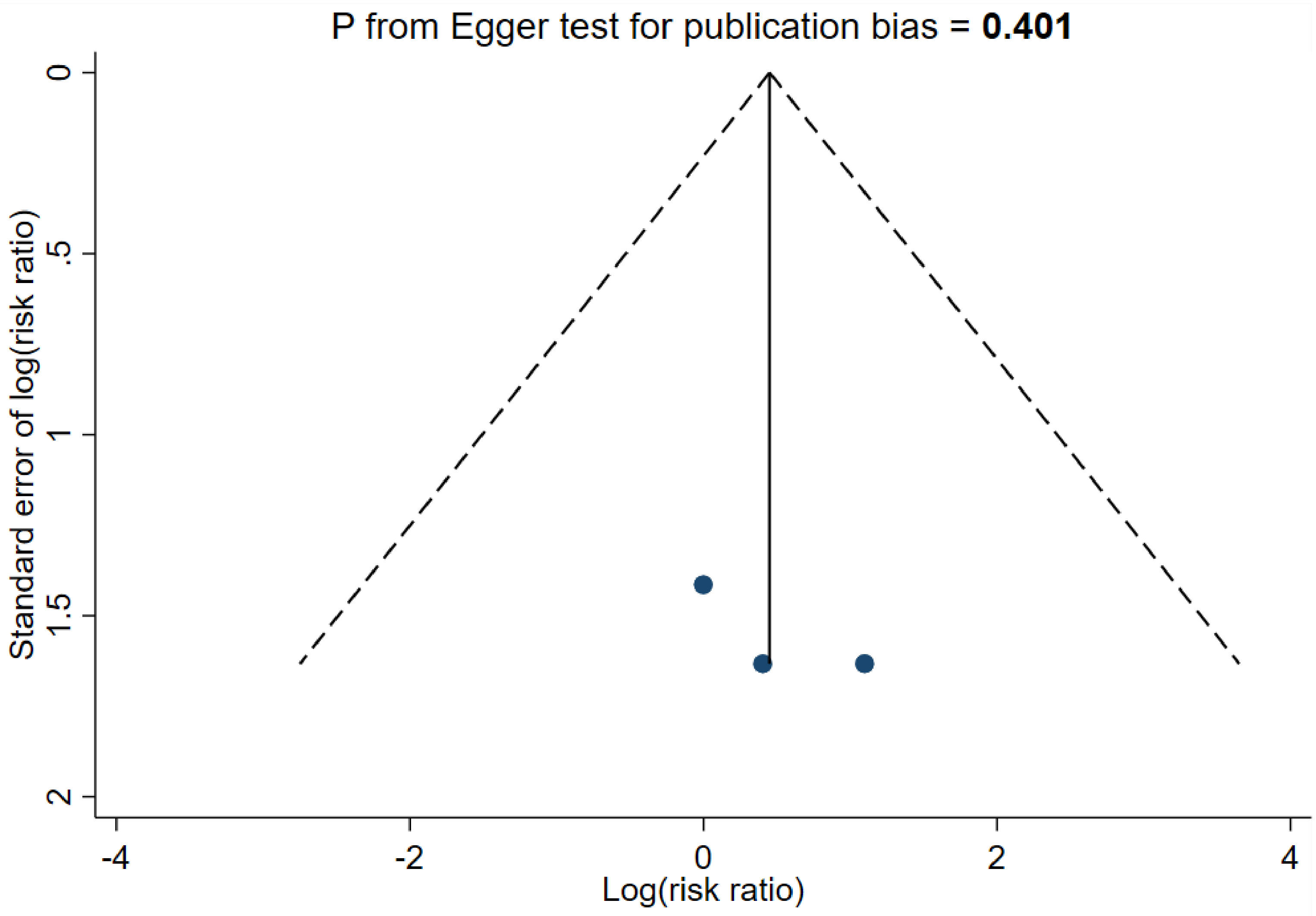

Figure S185 Funnel plot of Coronary ostial stenosis

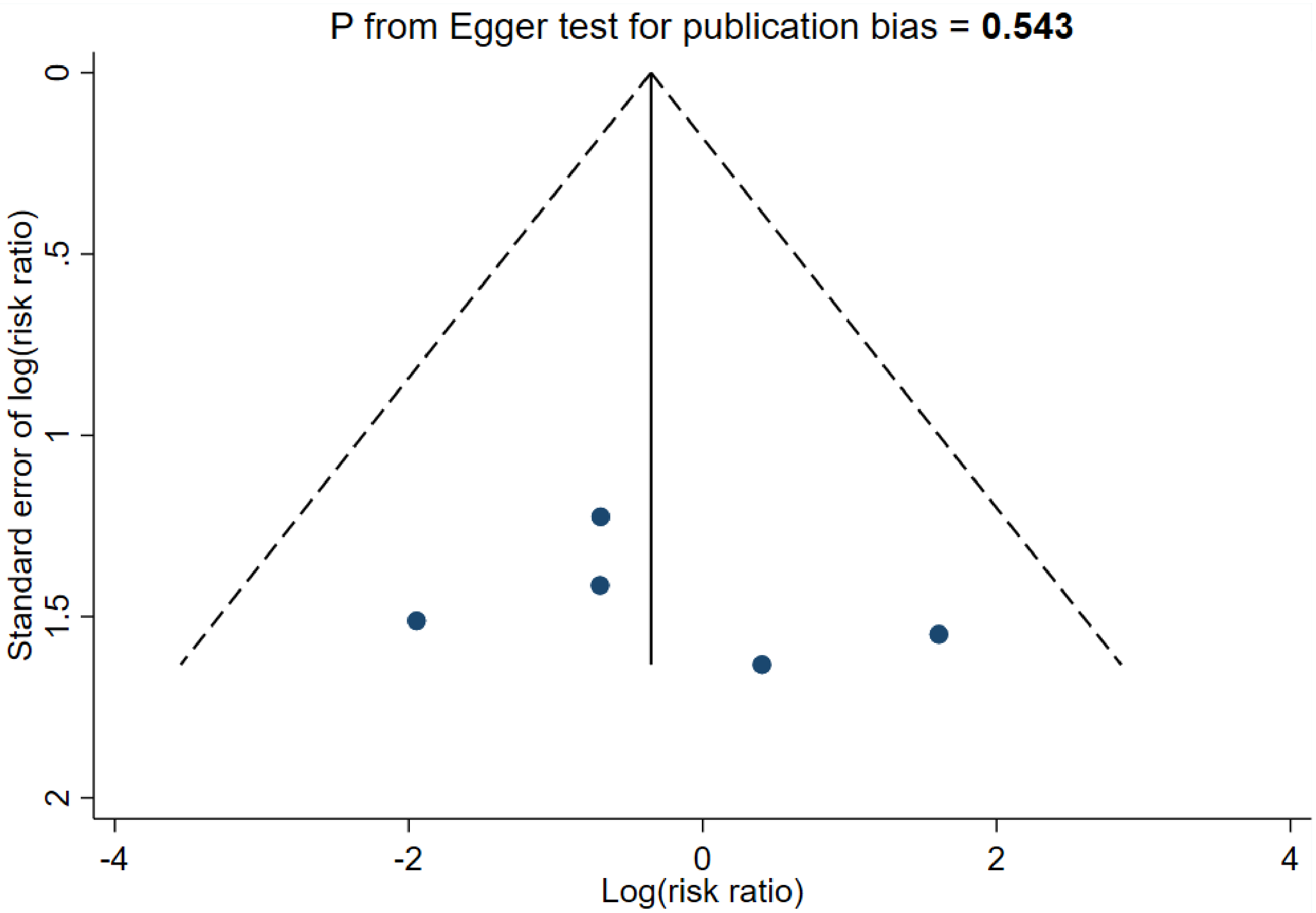

Figure S186 Funnel plot of Hypertensive heart disease

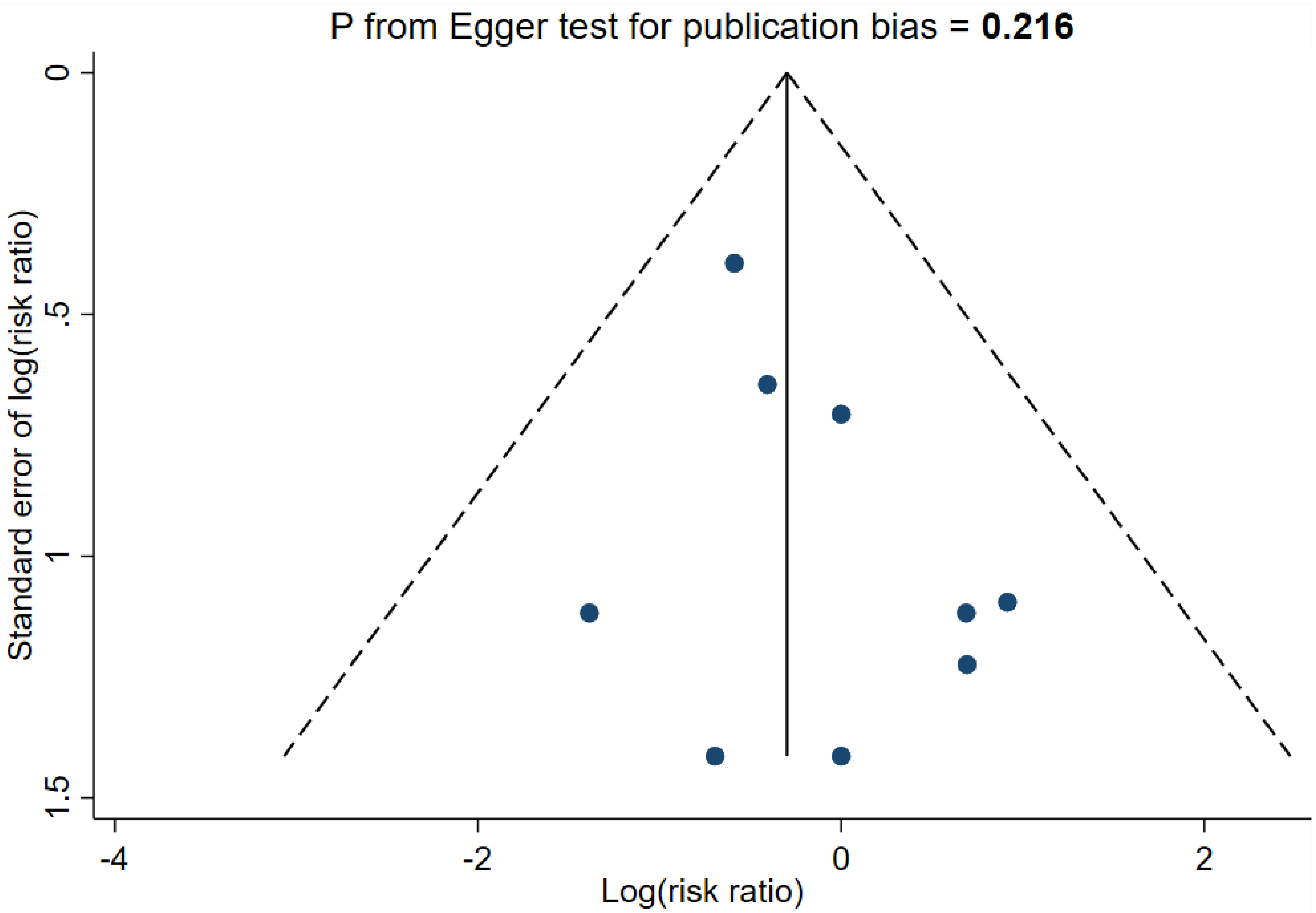

Figure S187 Funnel plot of Ischaemic cardiomyopathy

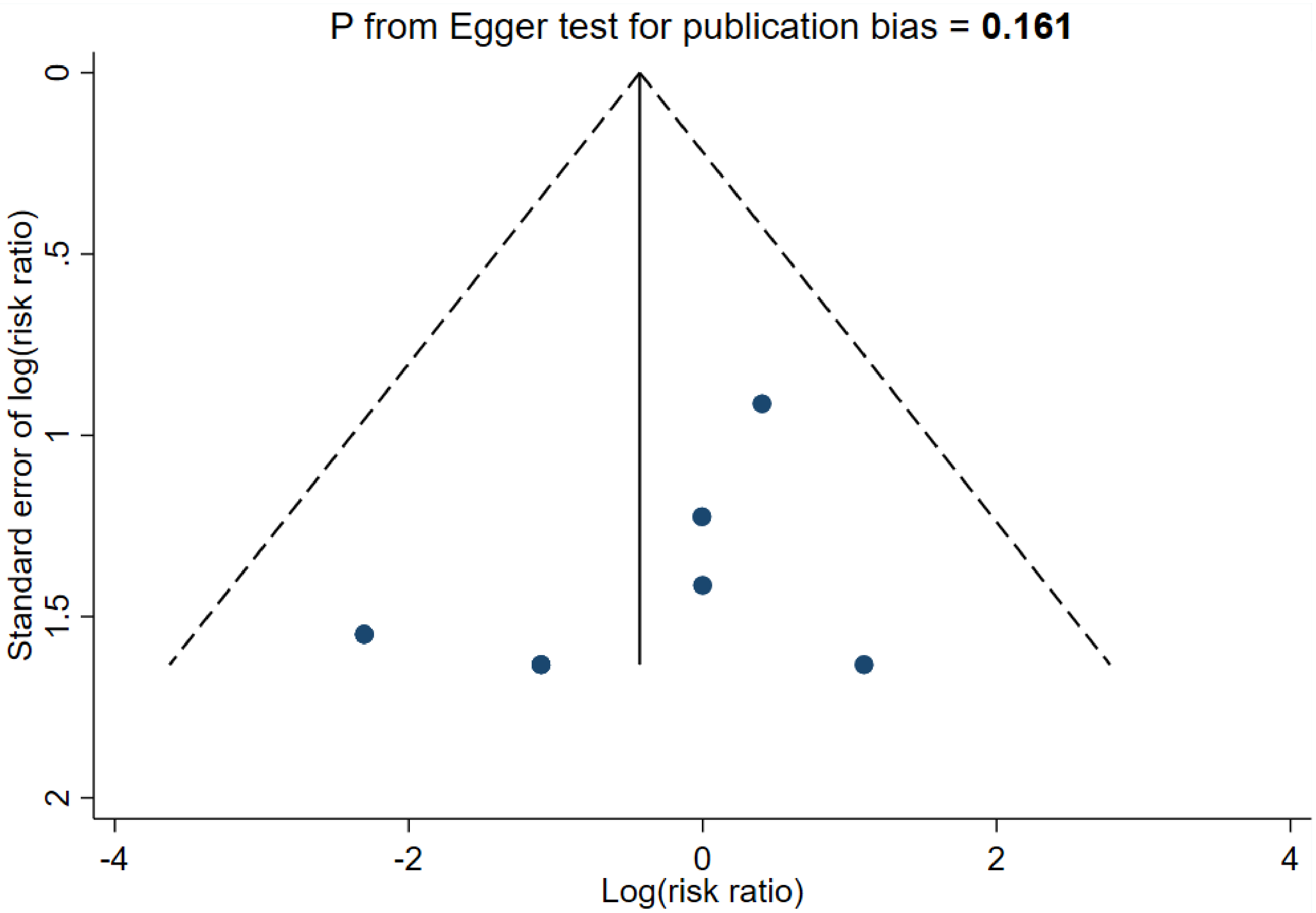

Figure S188 Funnel plot of Left ventricular dysfunction

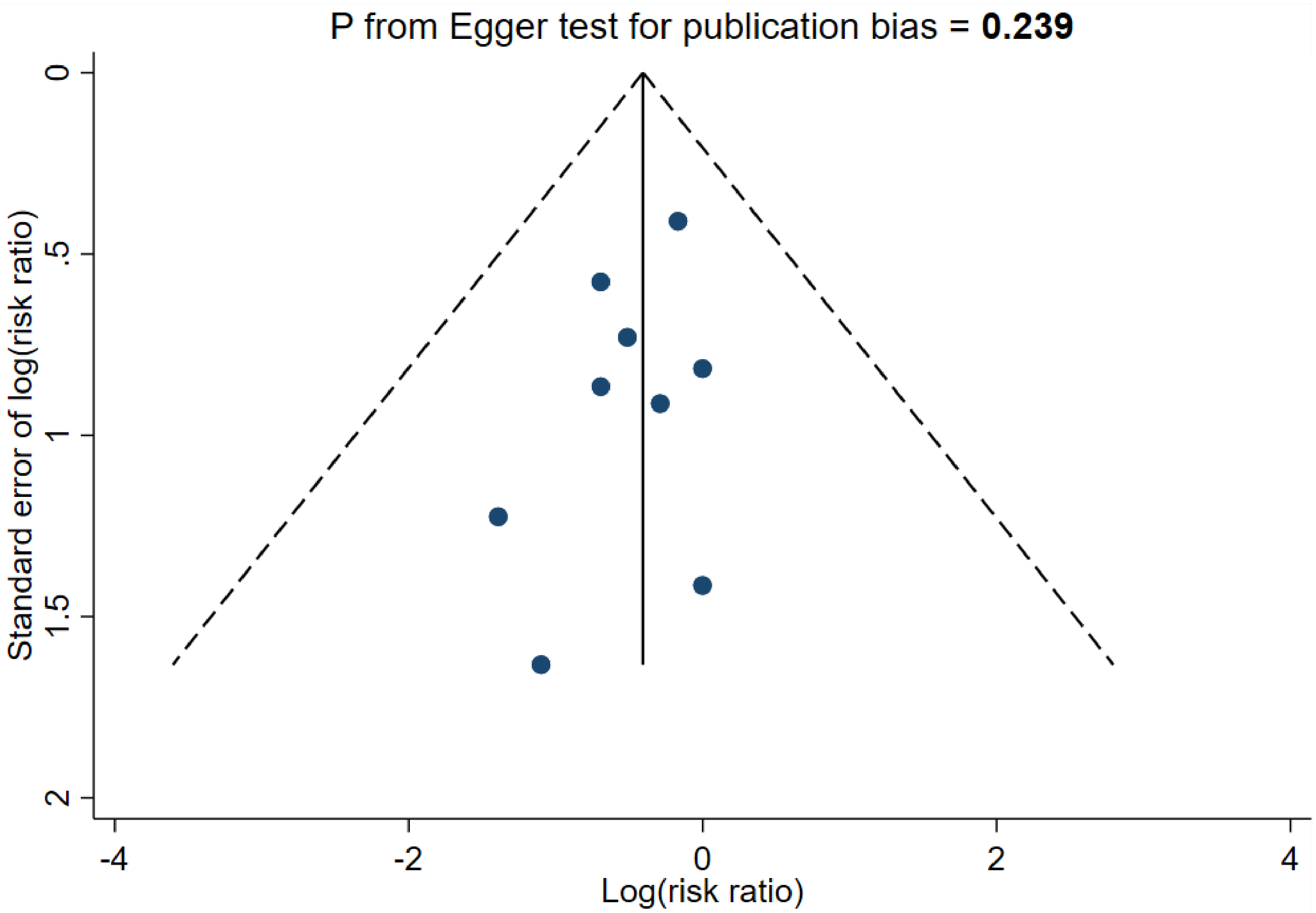

Figure S189 Funnel plot of Left ventricular failure

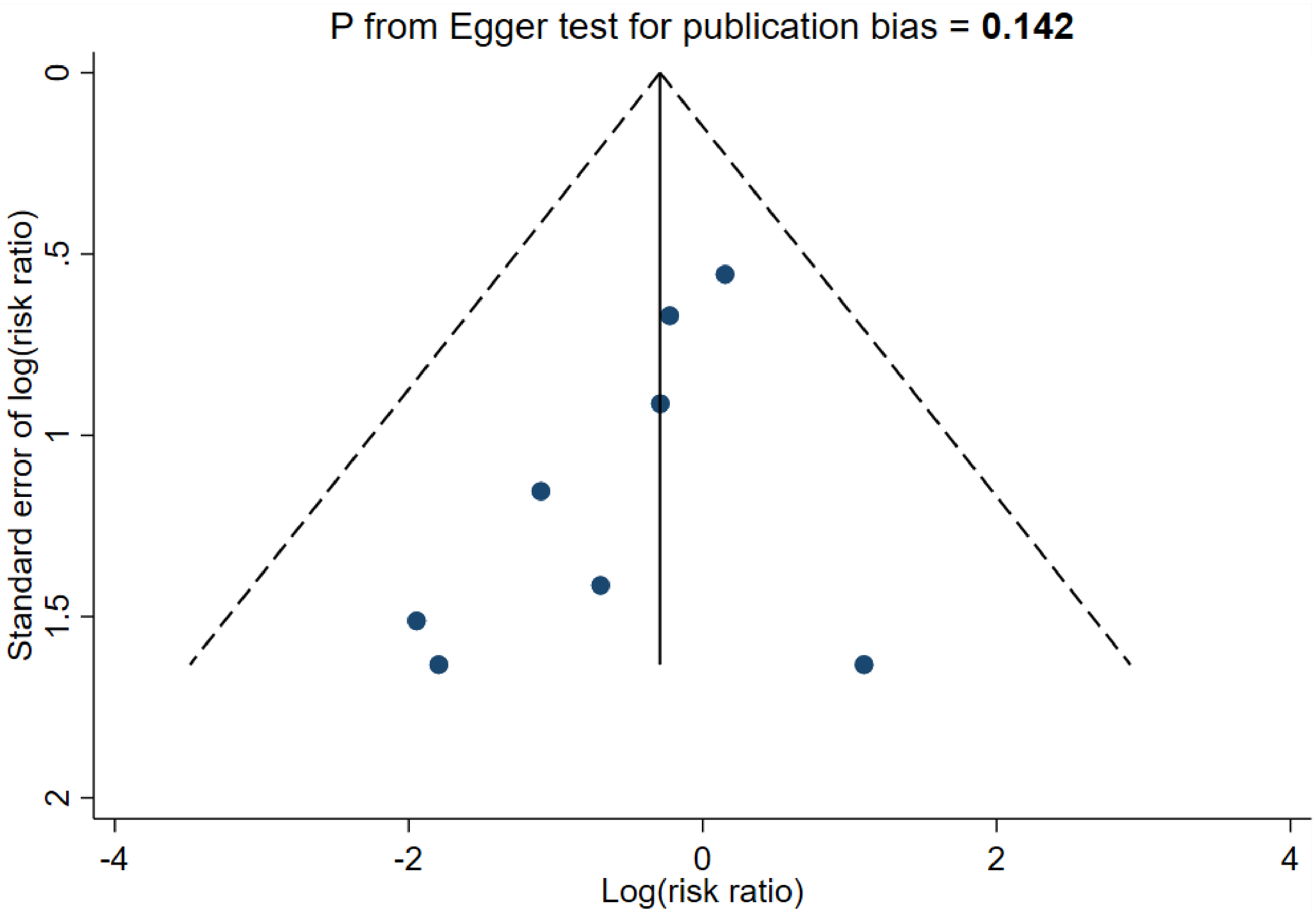

Figure S190 Funnel plot of Mitral valve incompetence

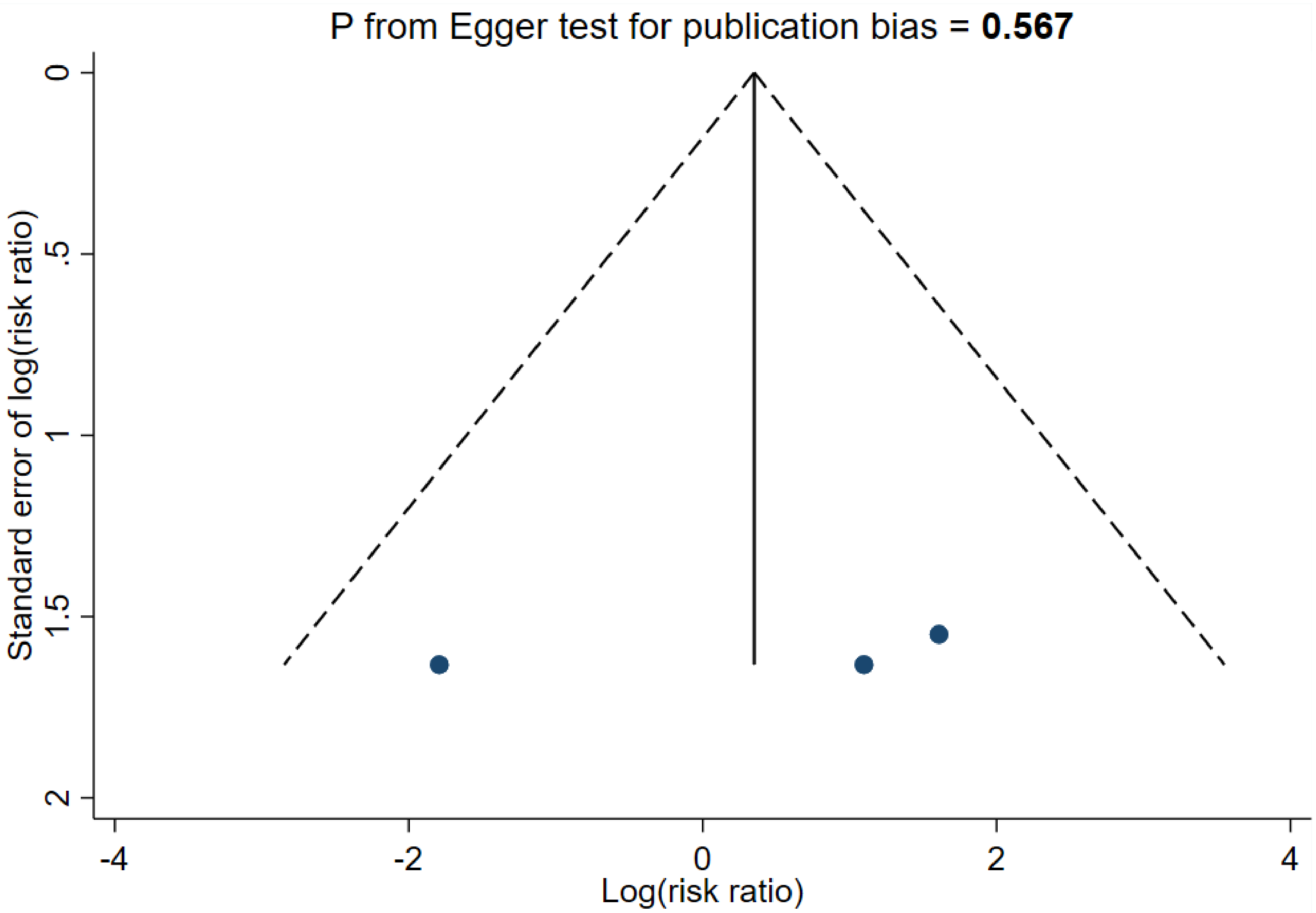

Figure S191 Funnel plot of Mitral valve stenosis

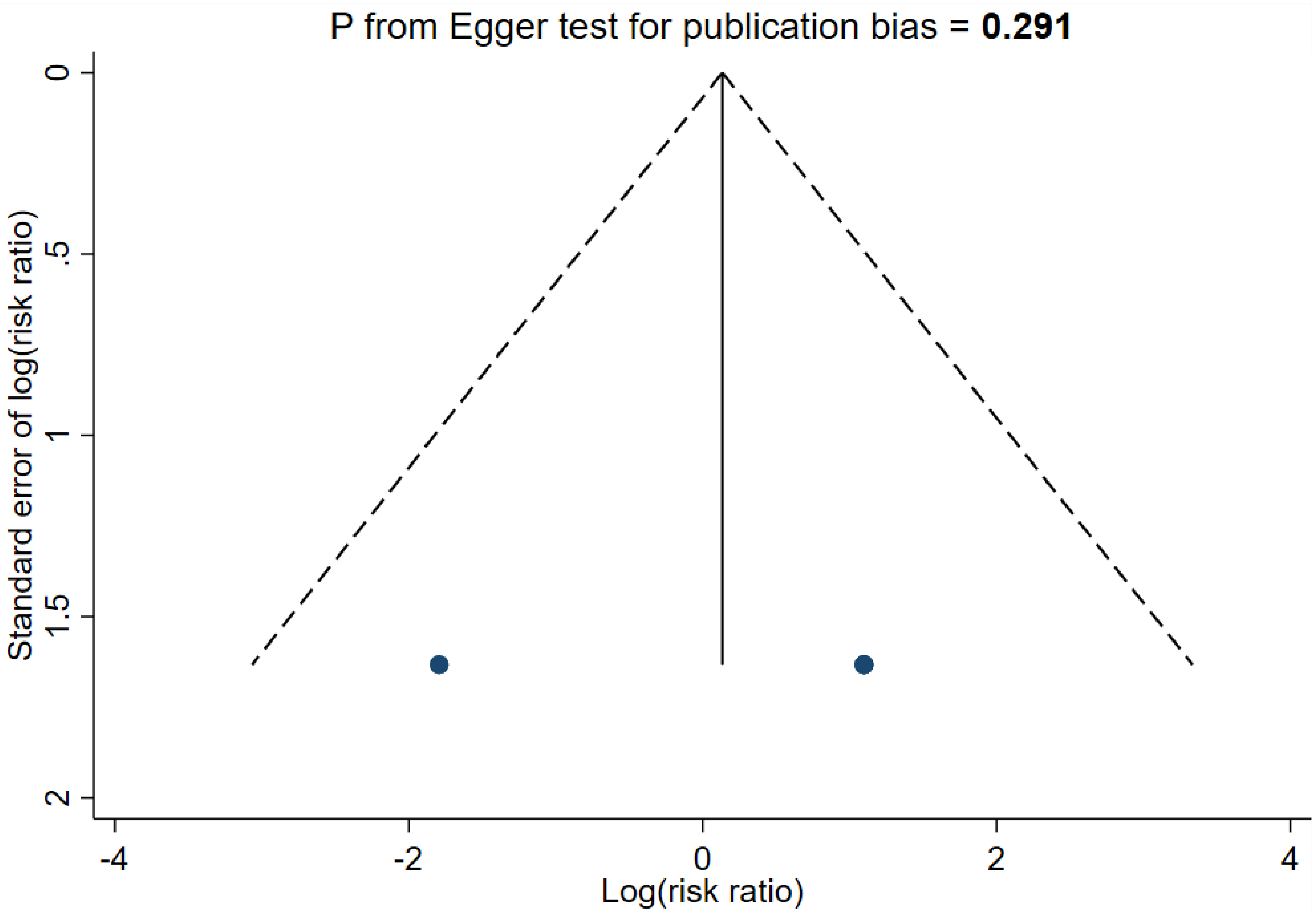

Figure S192 Funnel plot of Myocardial fibrosis

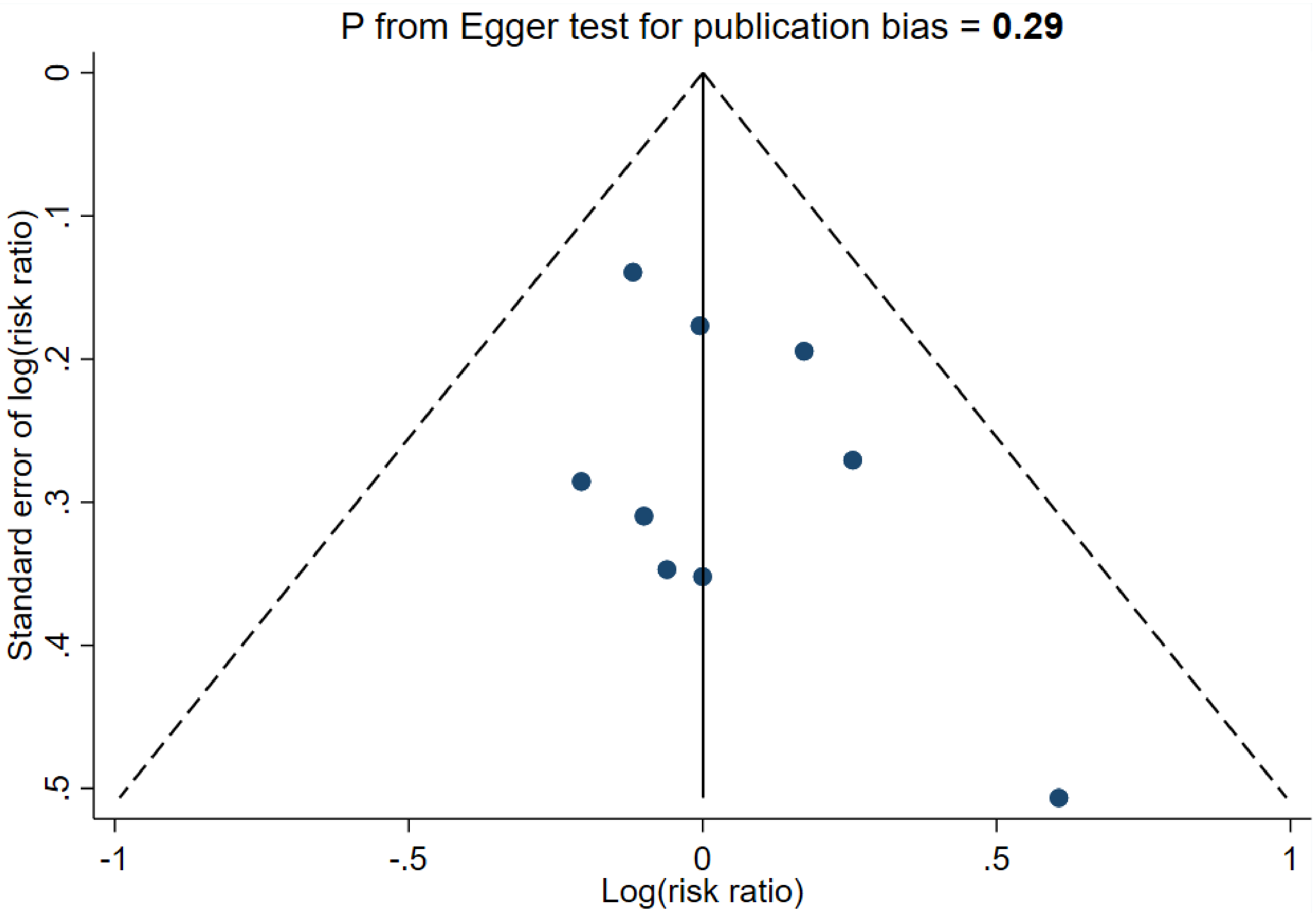

Figure S193 Funnel plot of Myocardial infarction

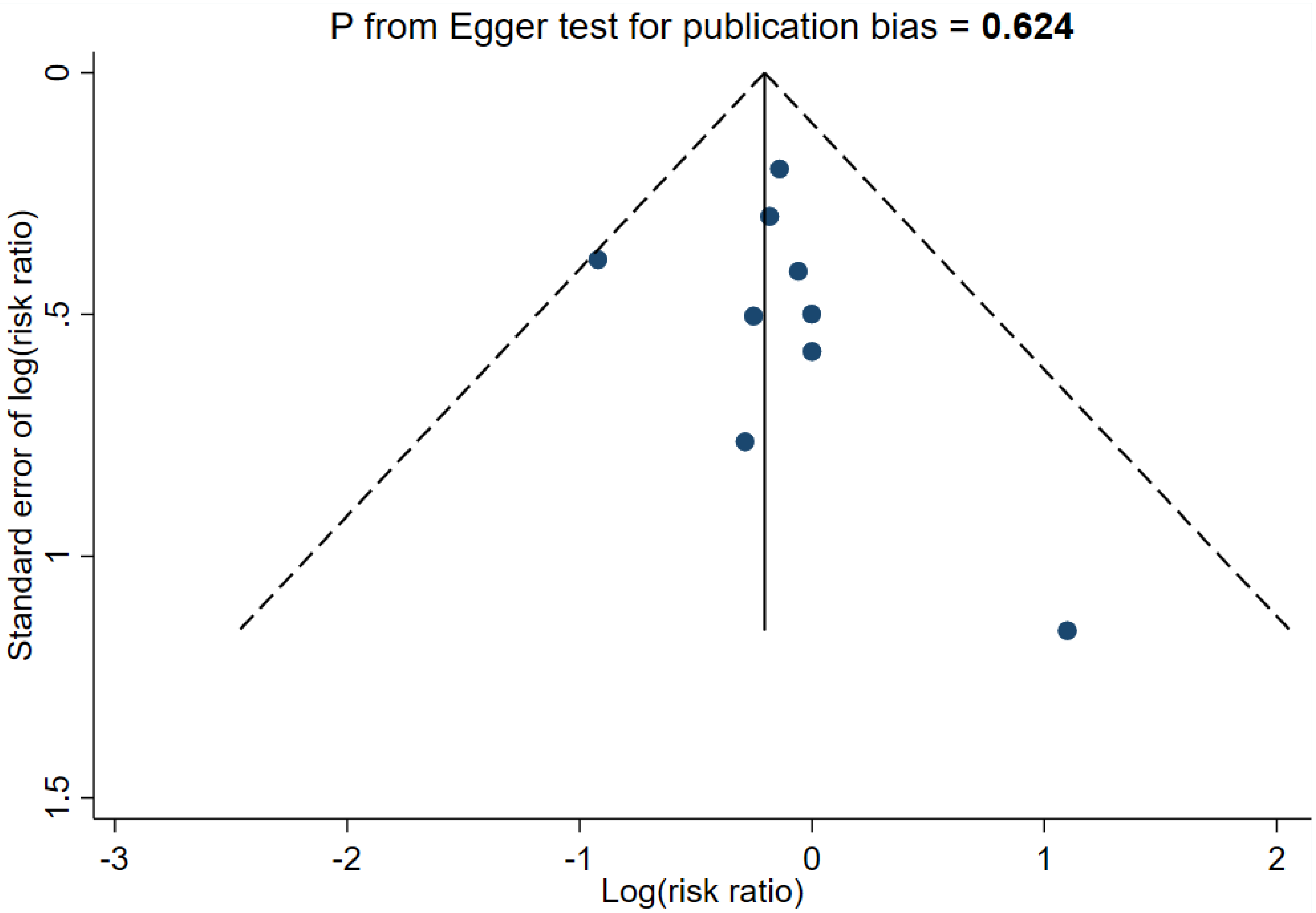

Figure S194 Funnel plot of Myocardial ischaemia

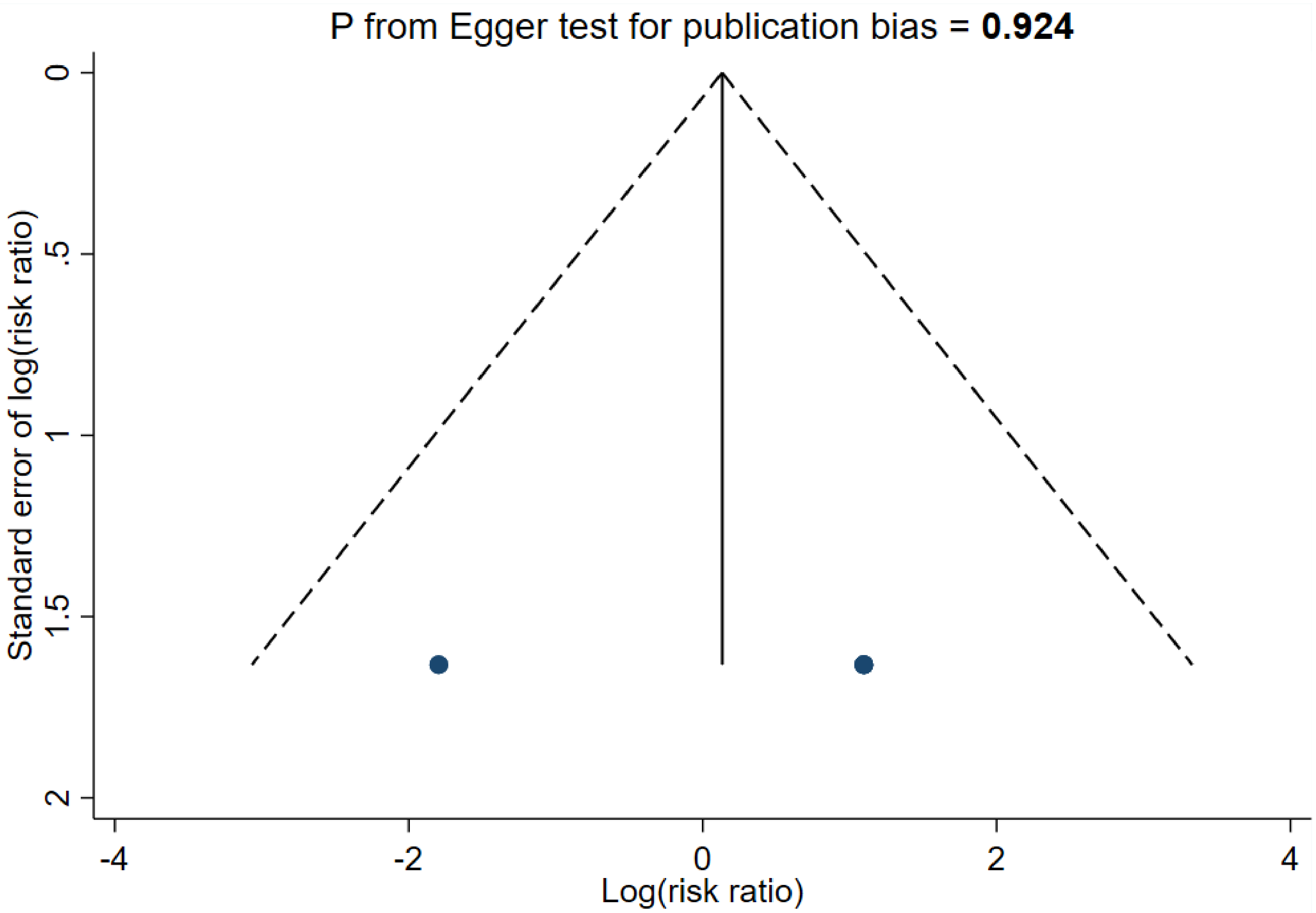

Figure S195 Funnel plot of Myocarditis

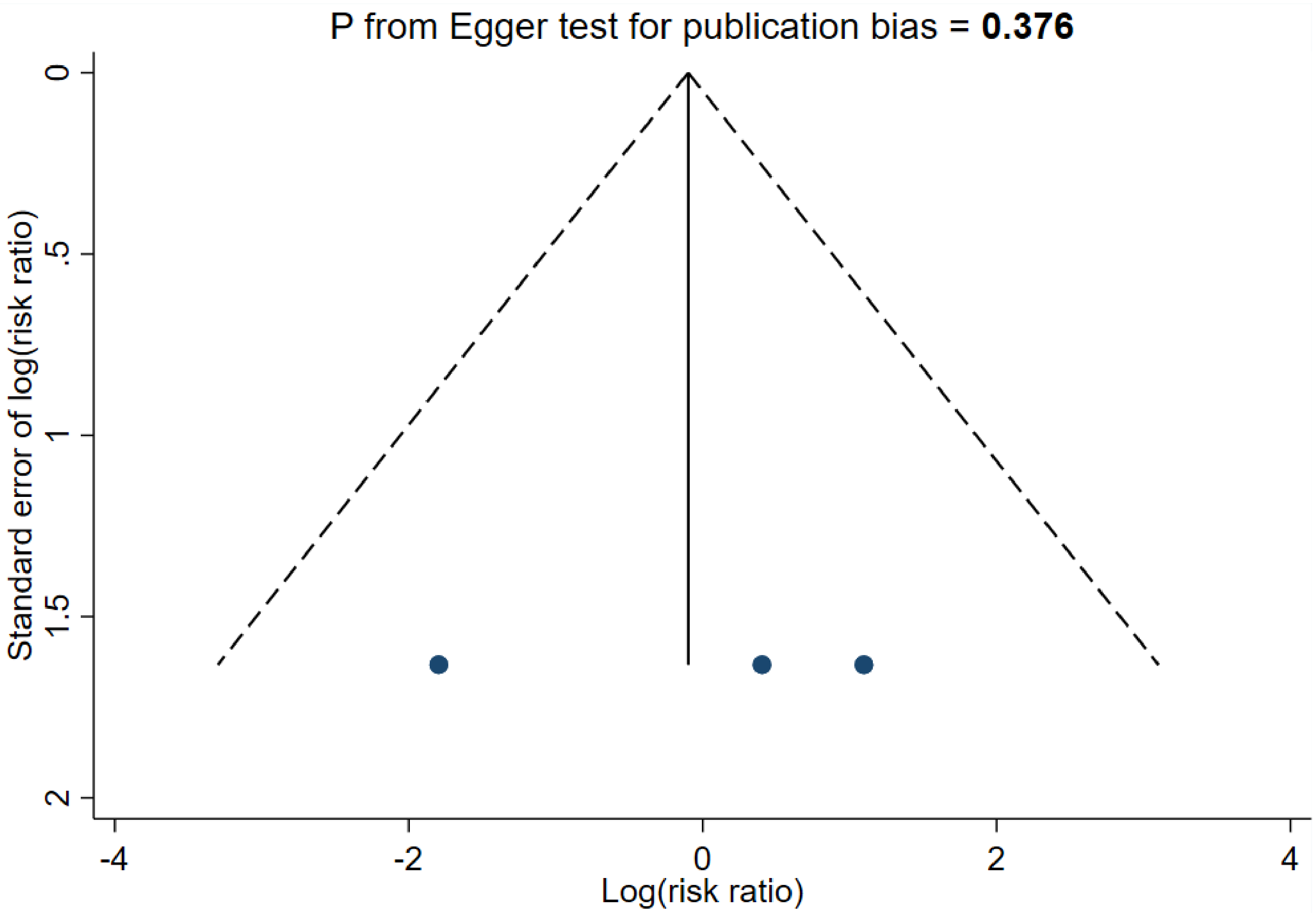

Figure S196 Funnel plot of Nodal arrhythmia

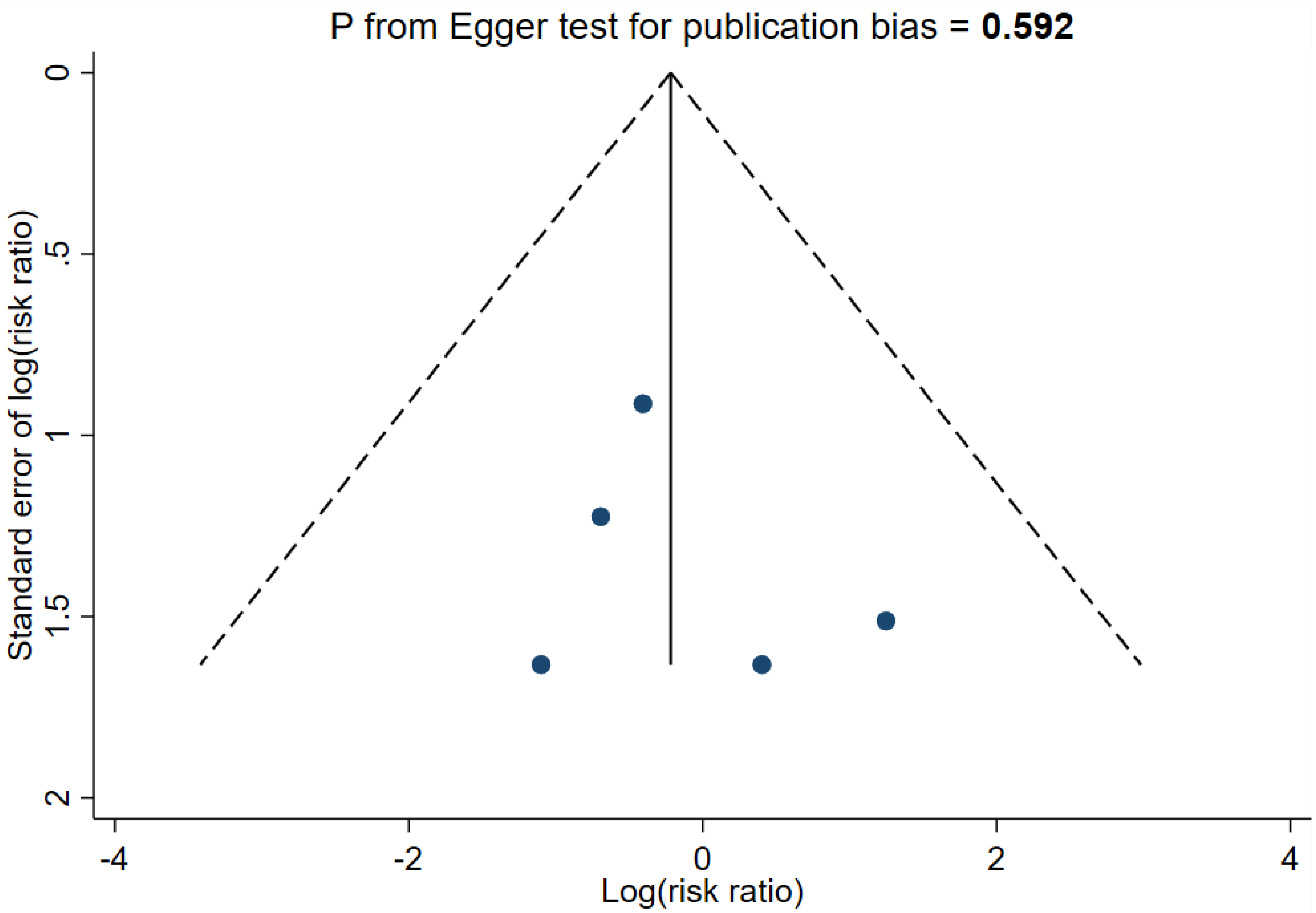

Figure S197 Funnel plot of Palpitations

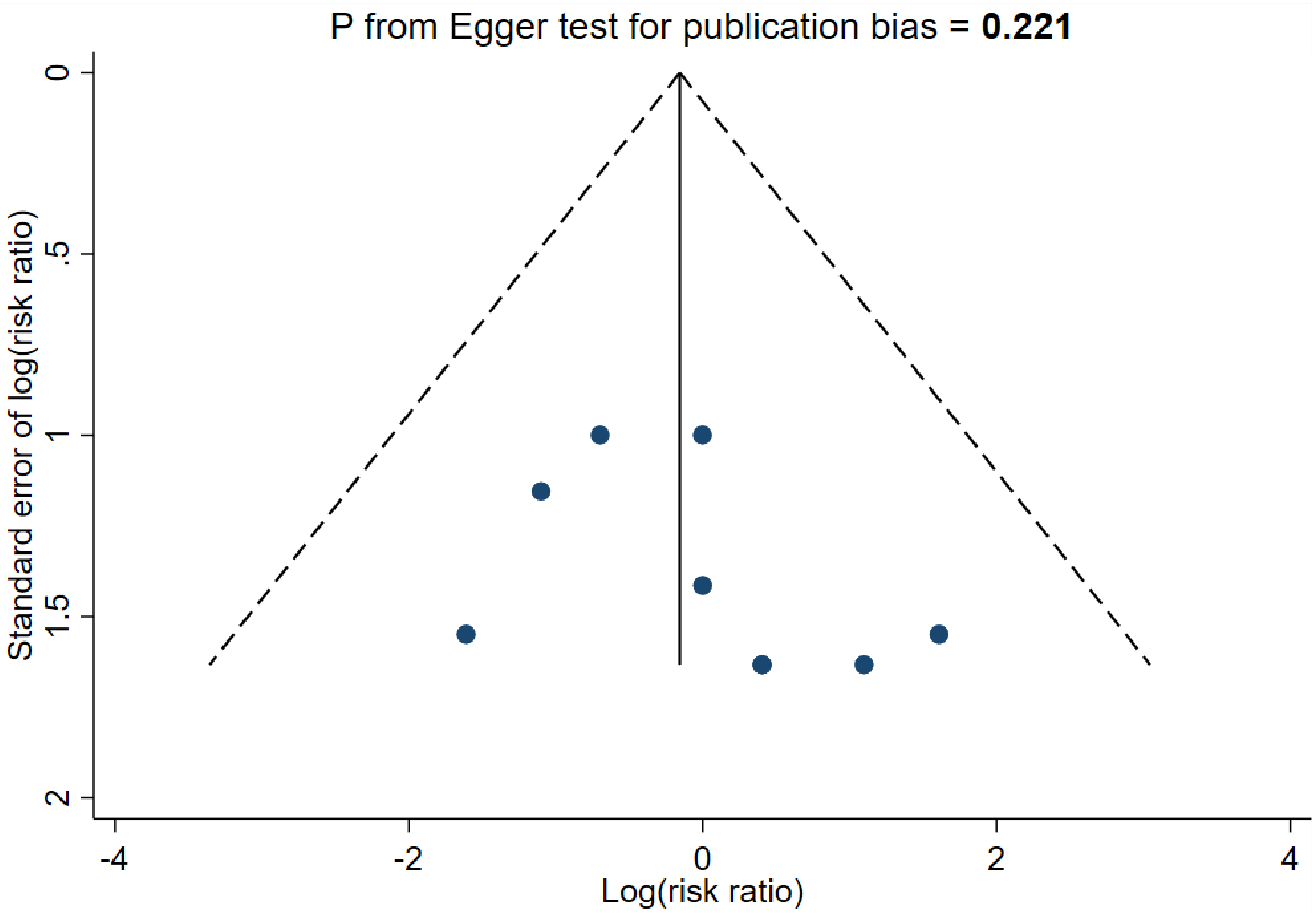

Figure S198 Funnel plot of Pericardial effusion

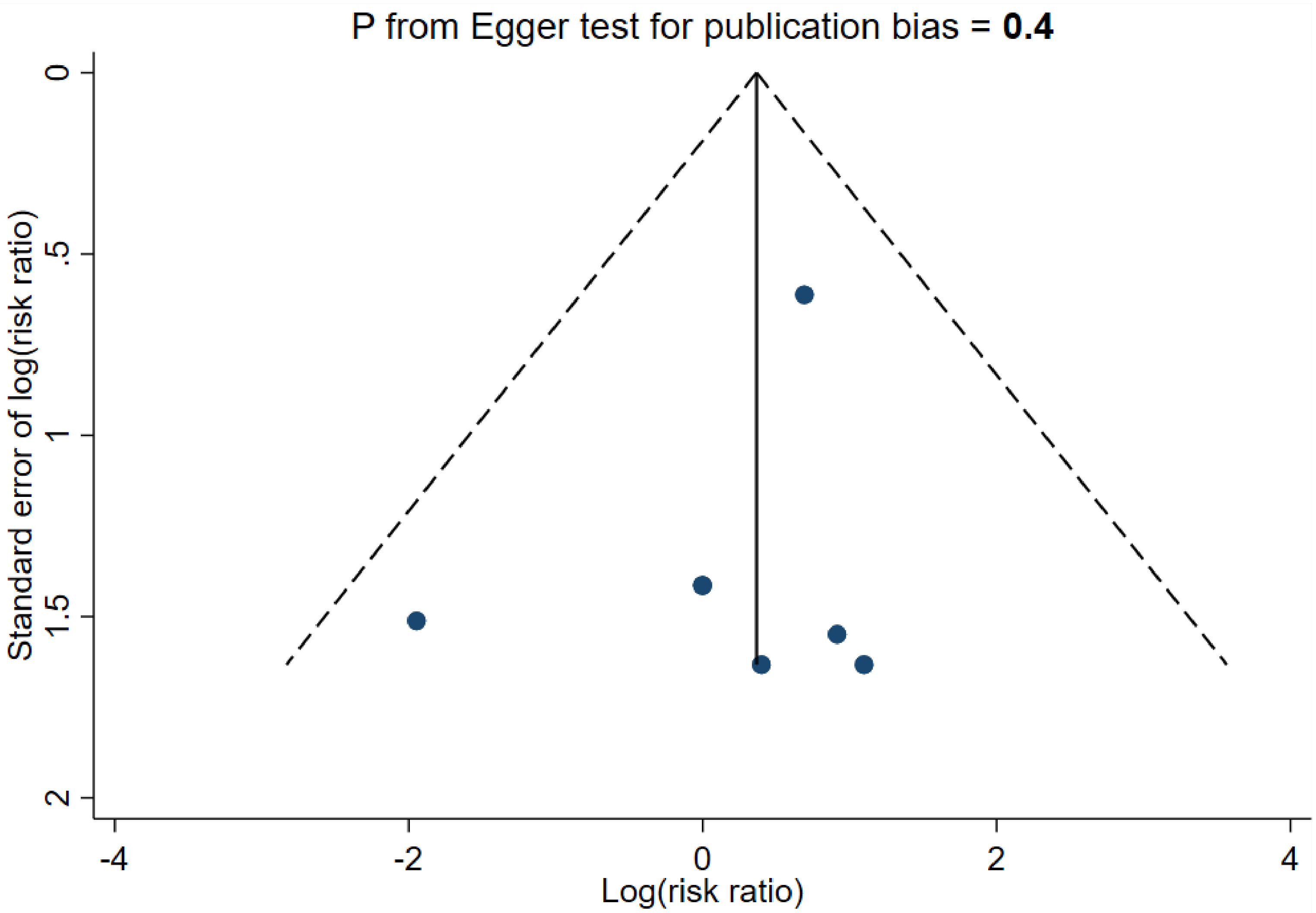

Figure S199 Funnel plot of Pericarditis

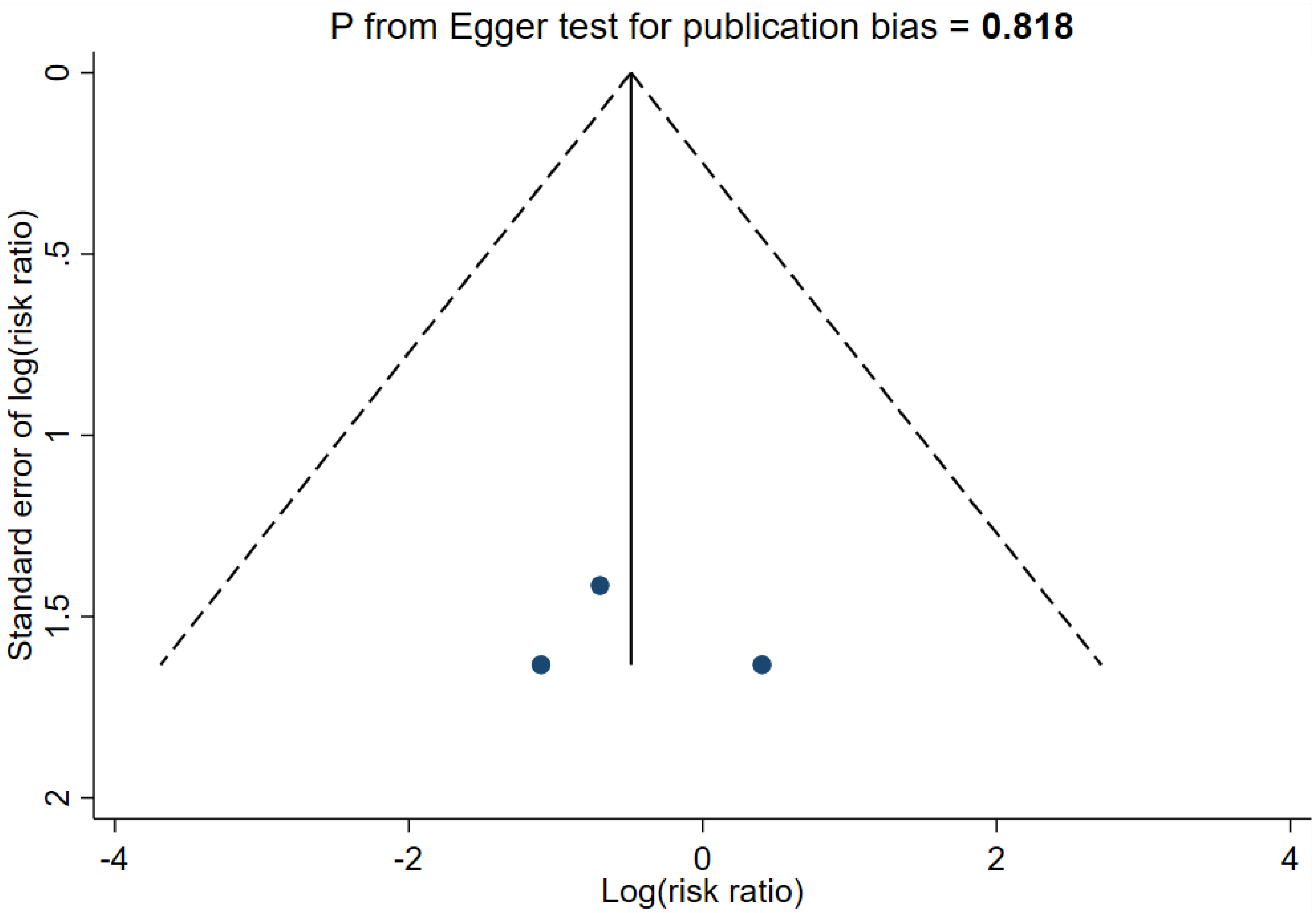

Figure S200 Funnel plot of Postinfarction angina

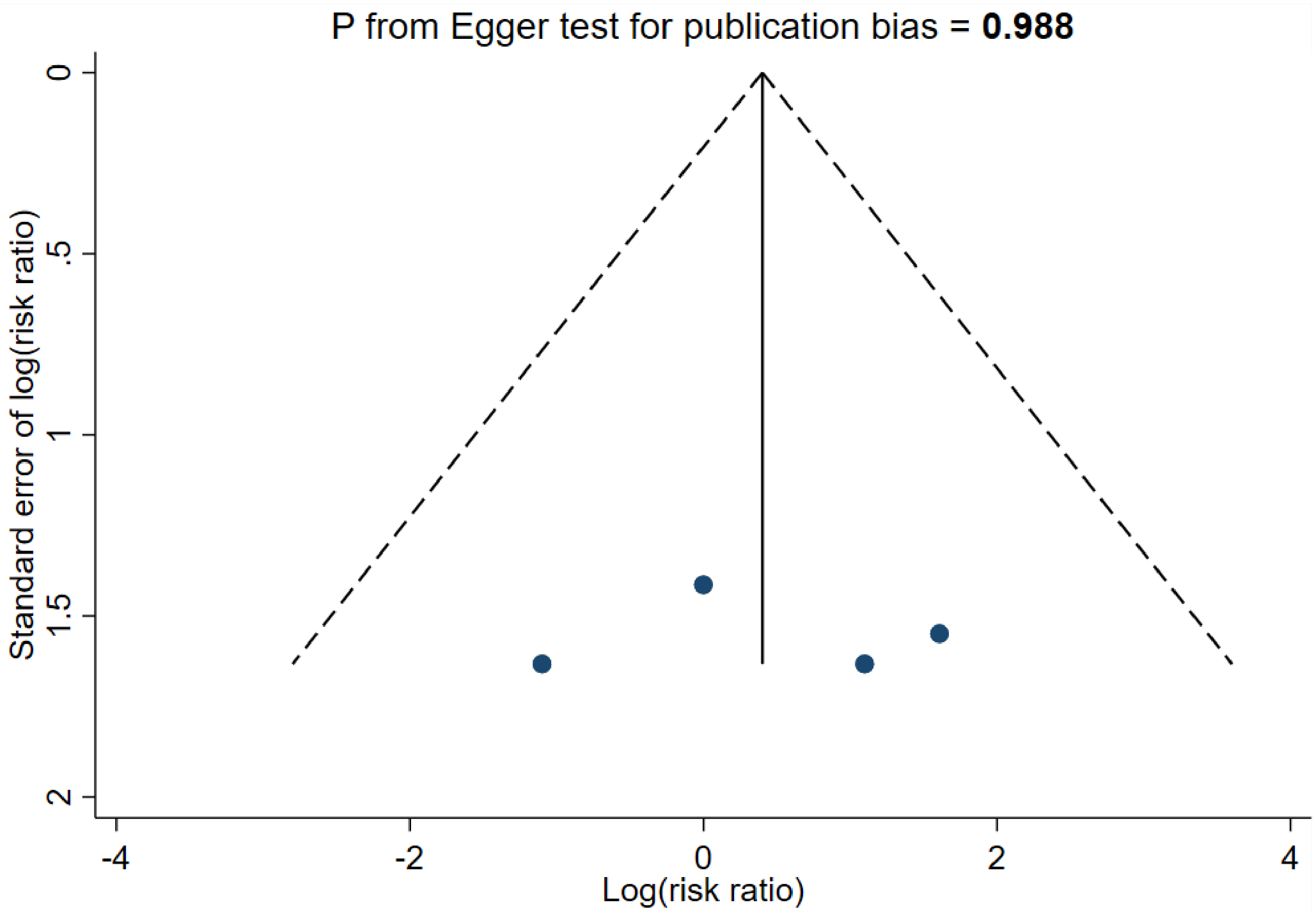

Figure S201 Funnel plot of Prinzmetal angina

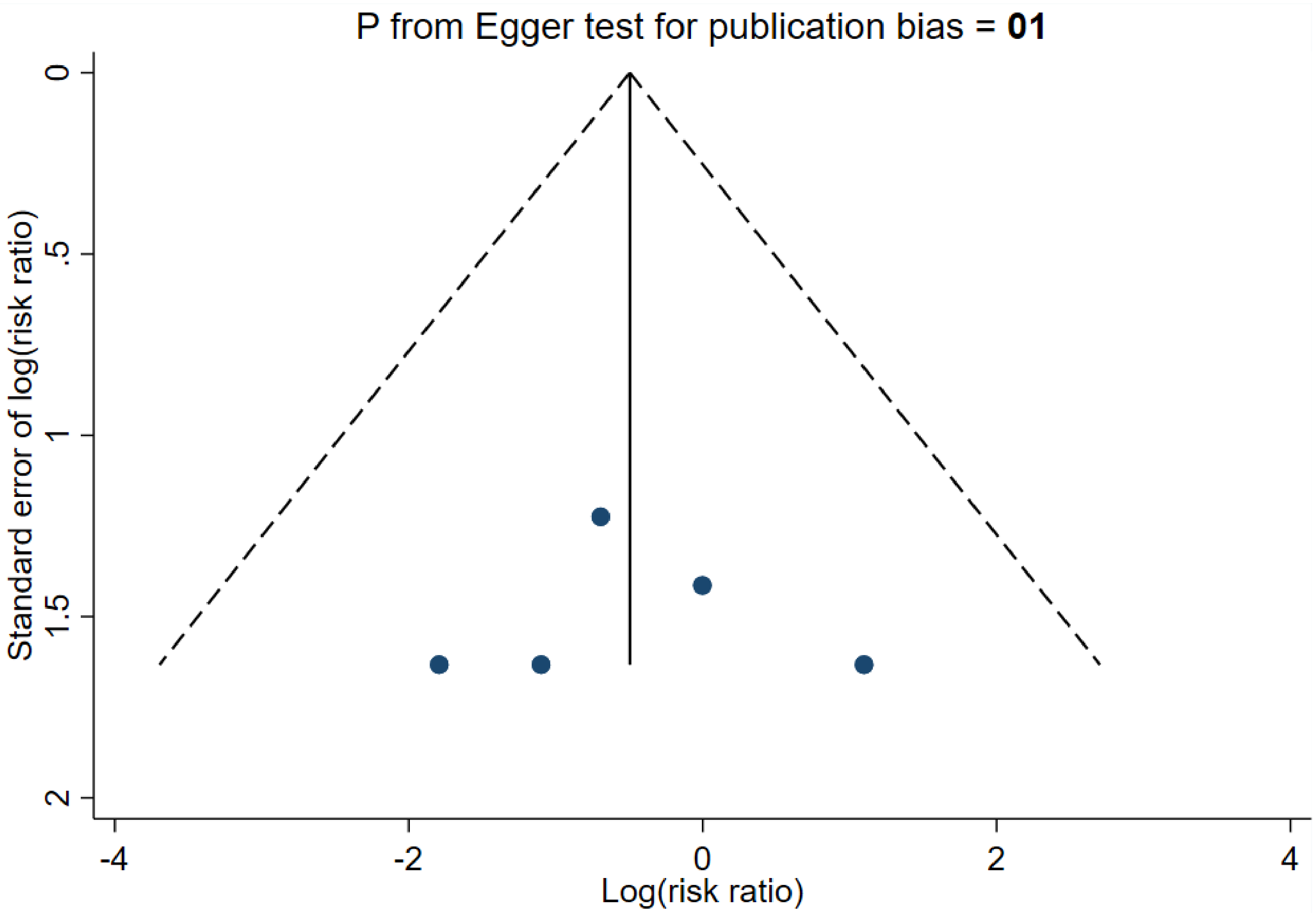

Figure S202 Funnel plot of Pulseless electrical activity

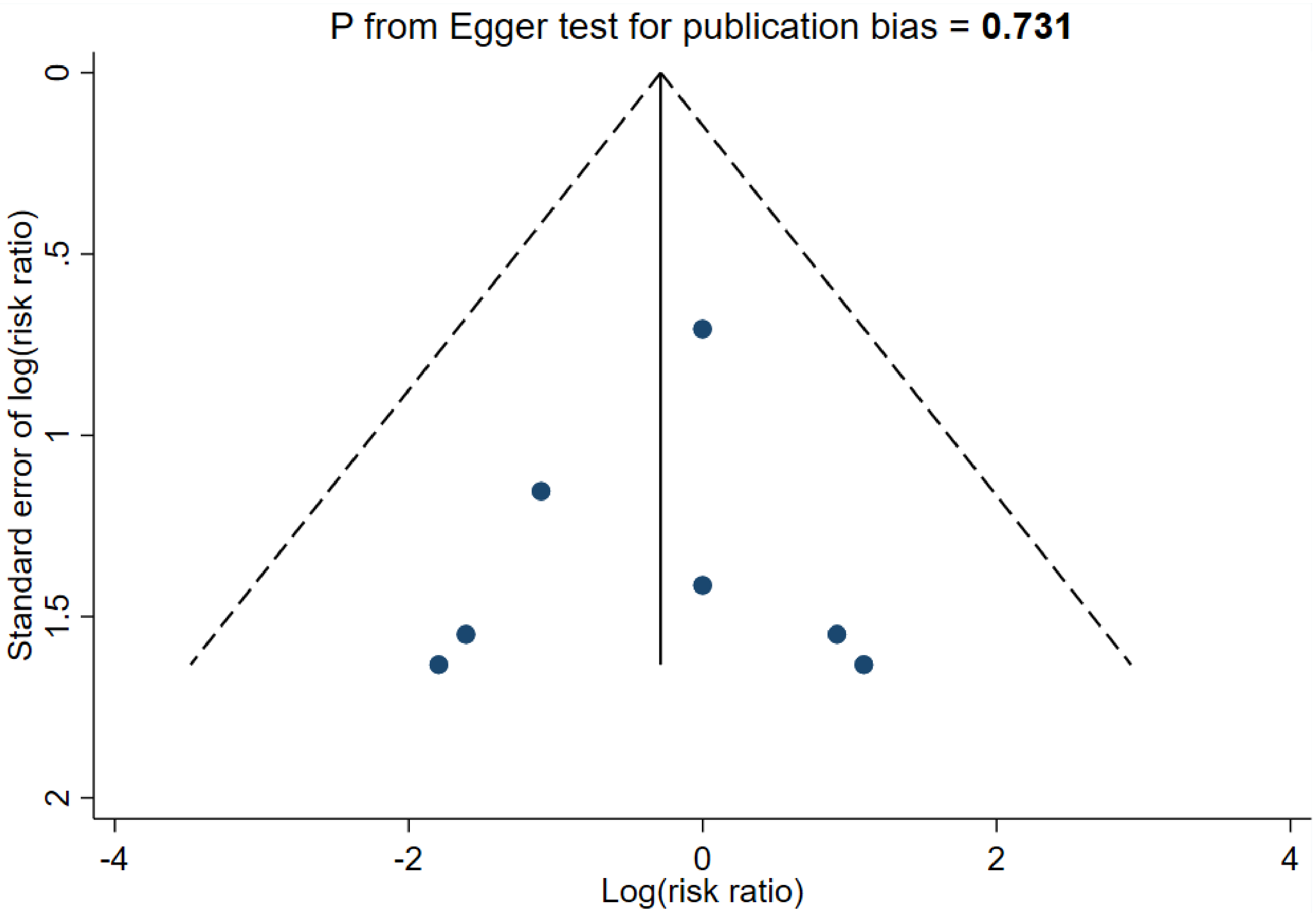

Figure S203 Funnel plot of Right ventricular failure

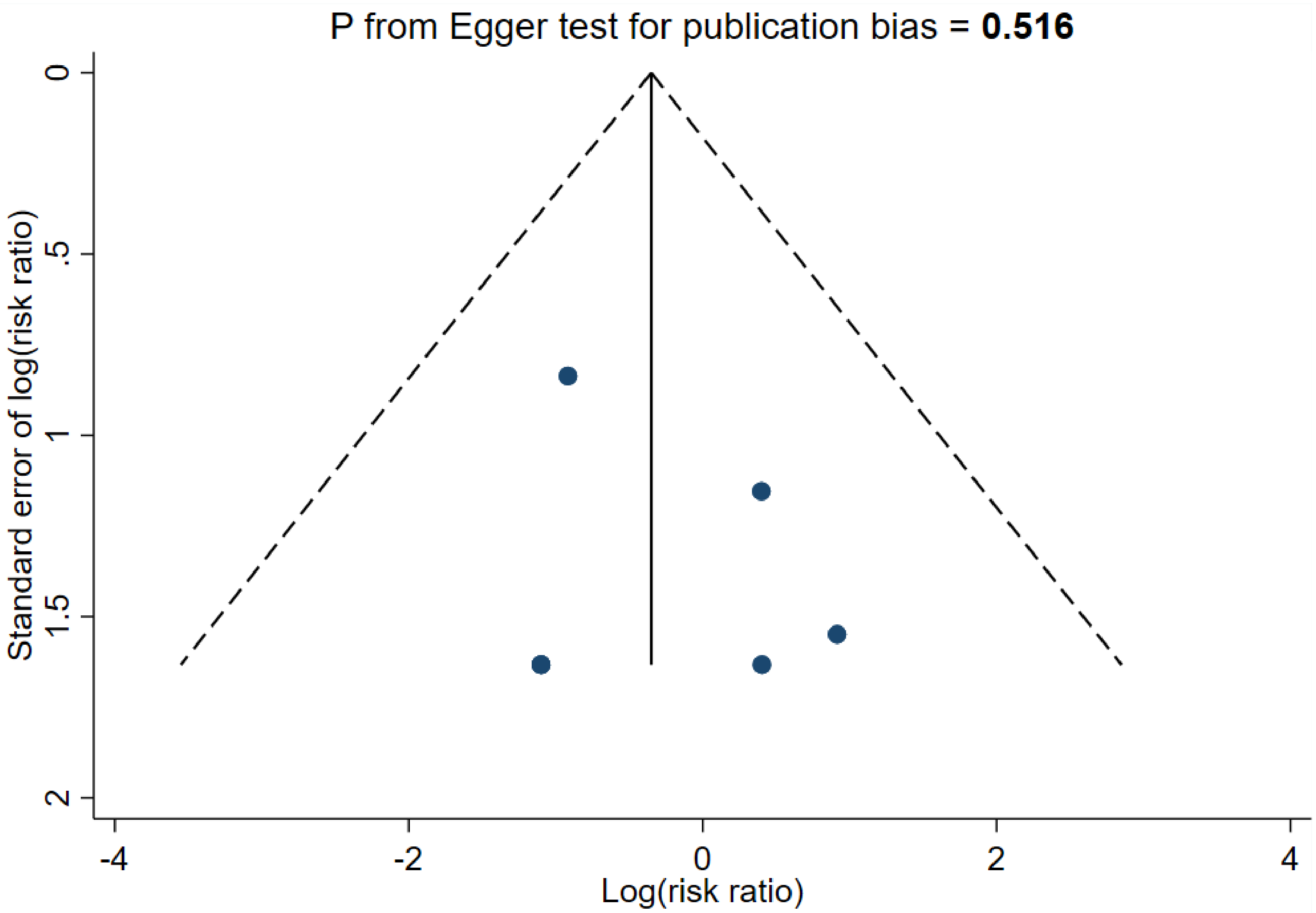

Figure S204 Funnel plot of Sinus bradycardia

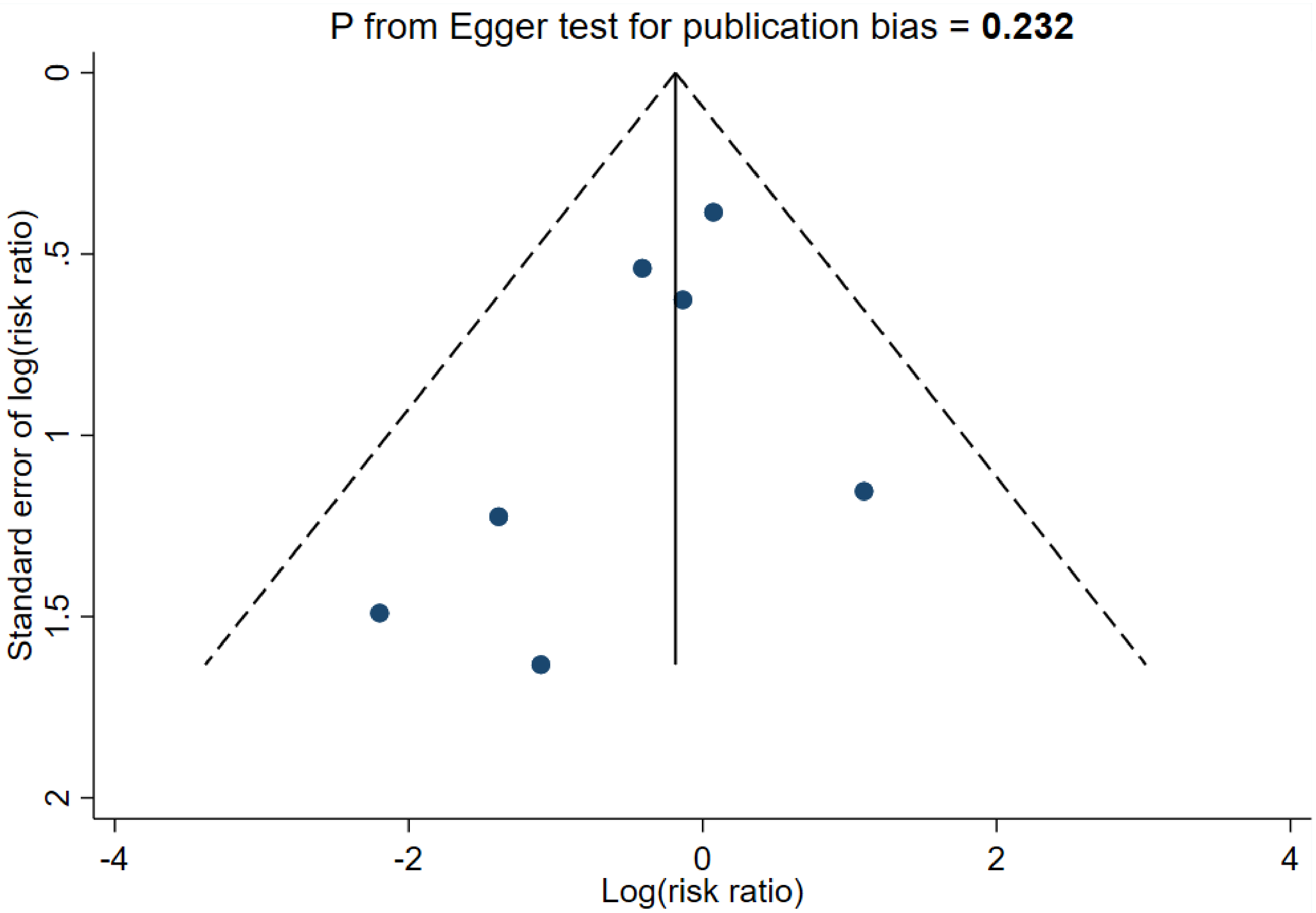

Figure S205 Funnel plot of Sinus node dysfunction

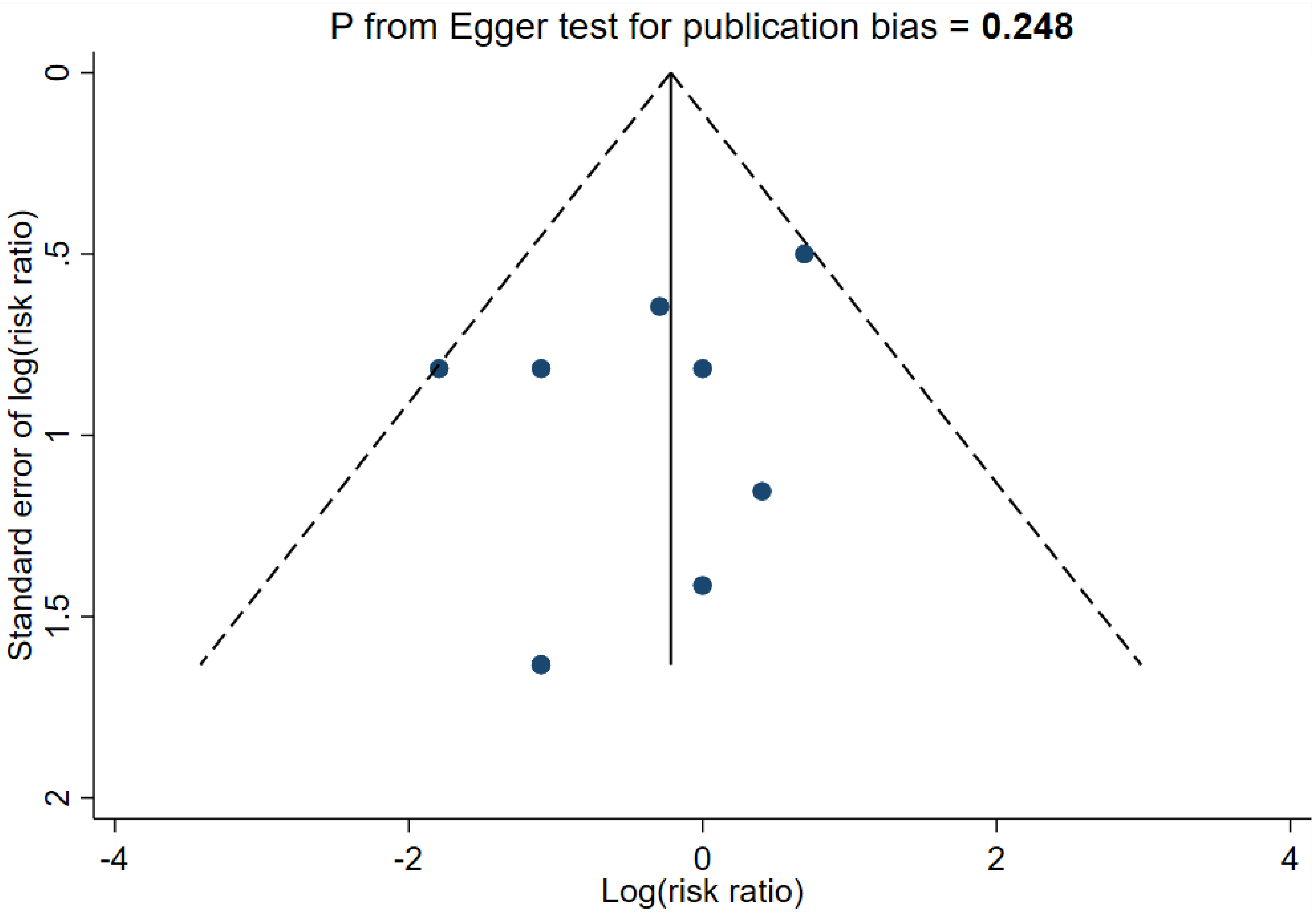

Figure S206 Funnel plot of Supraventricular tachycardia

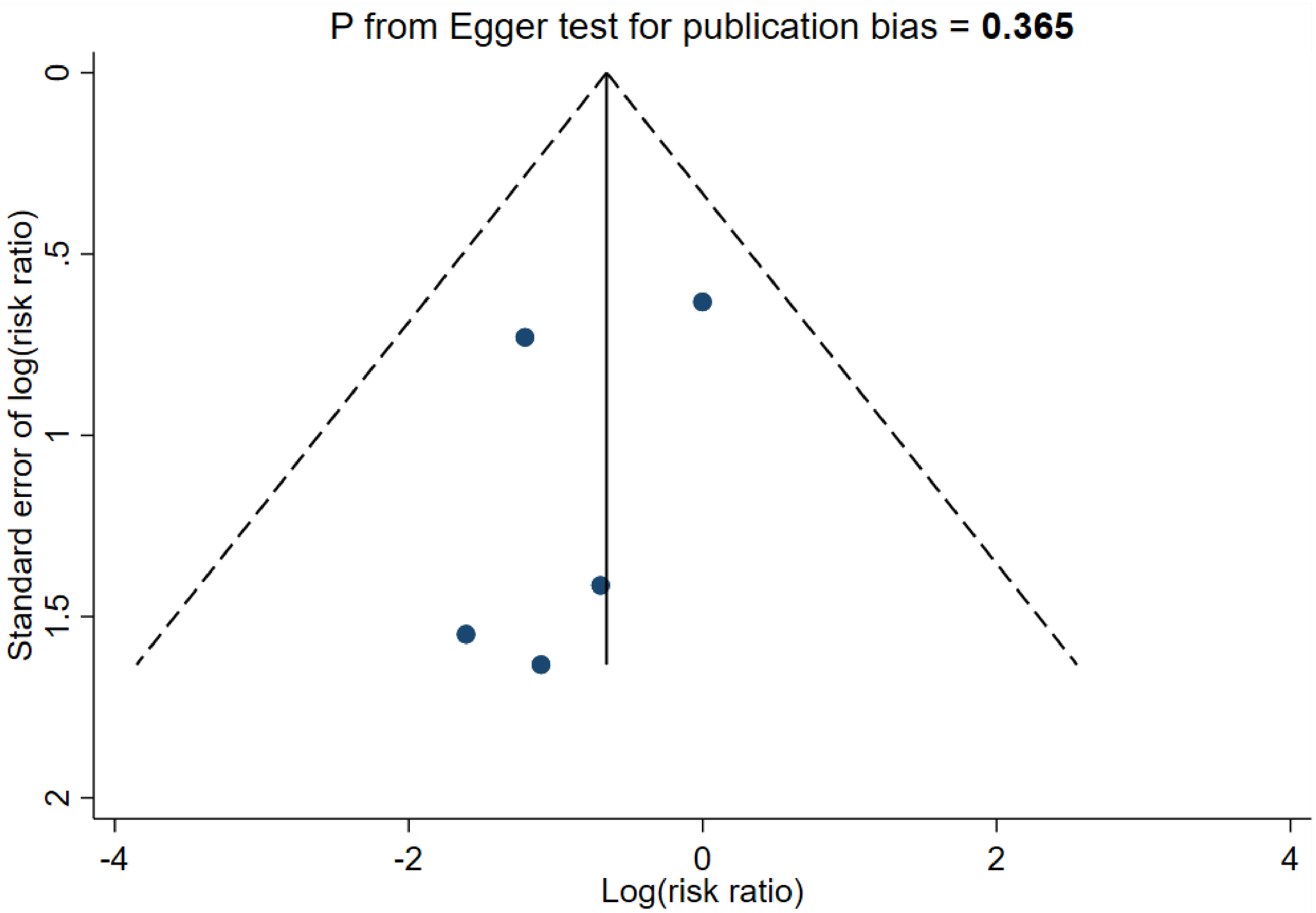

Figure S207 Funnel plot of Tachycardia

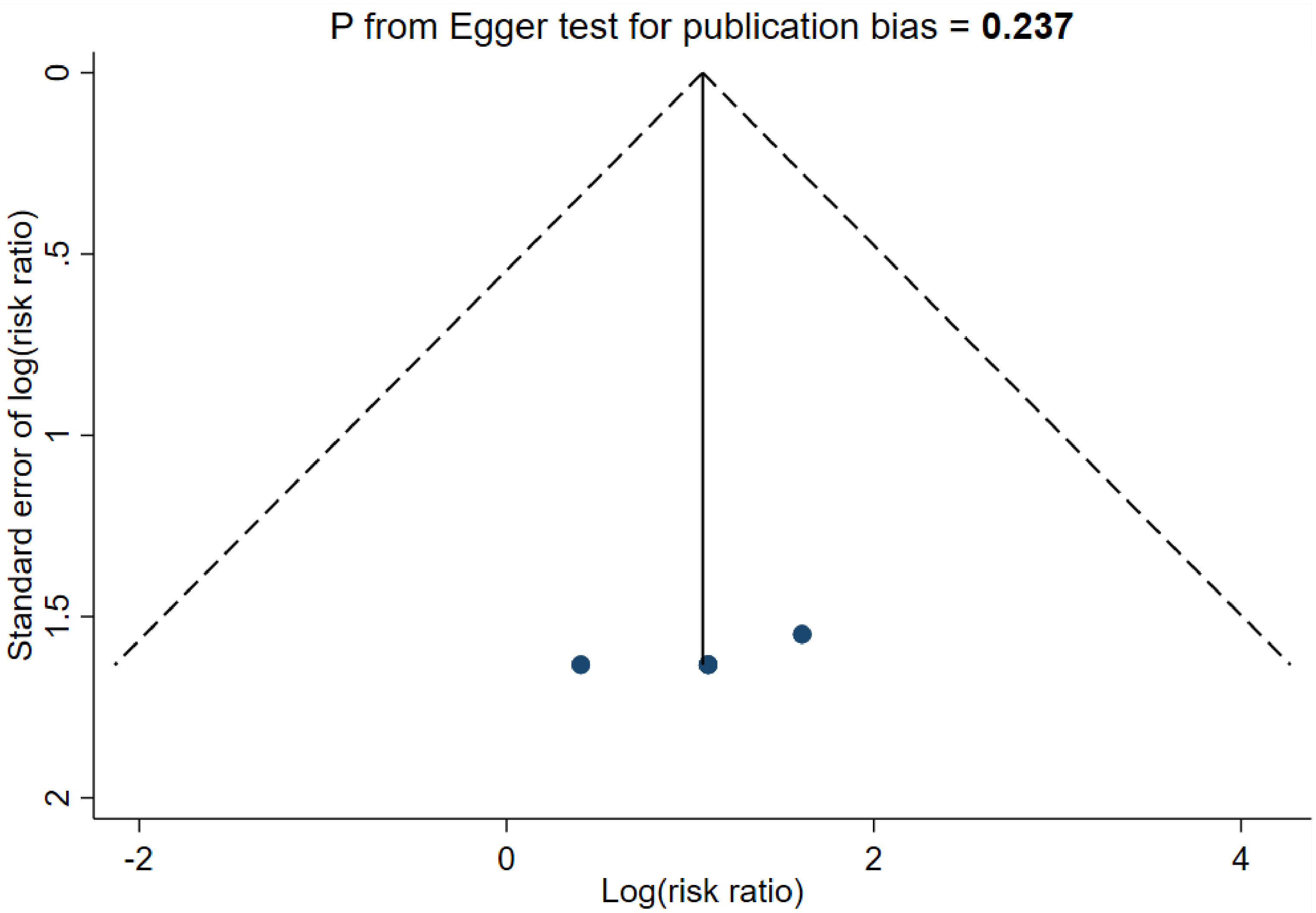

Figure S208 Funnel plot of Torsade de pointes

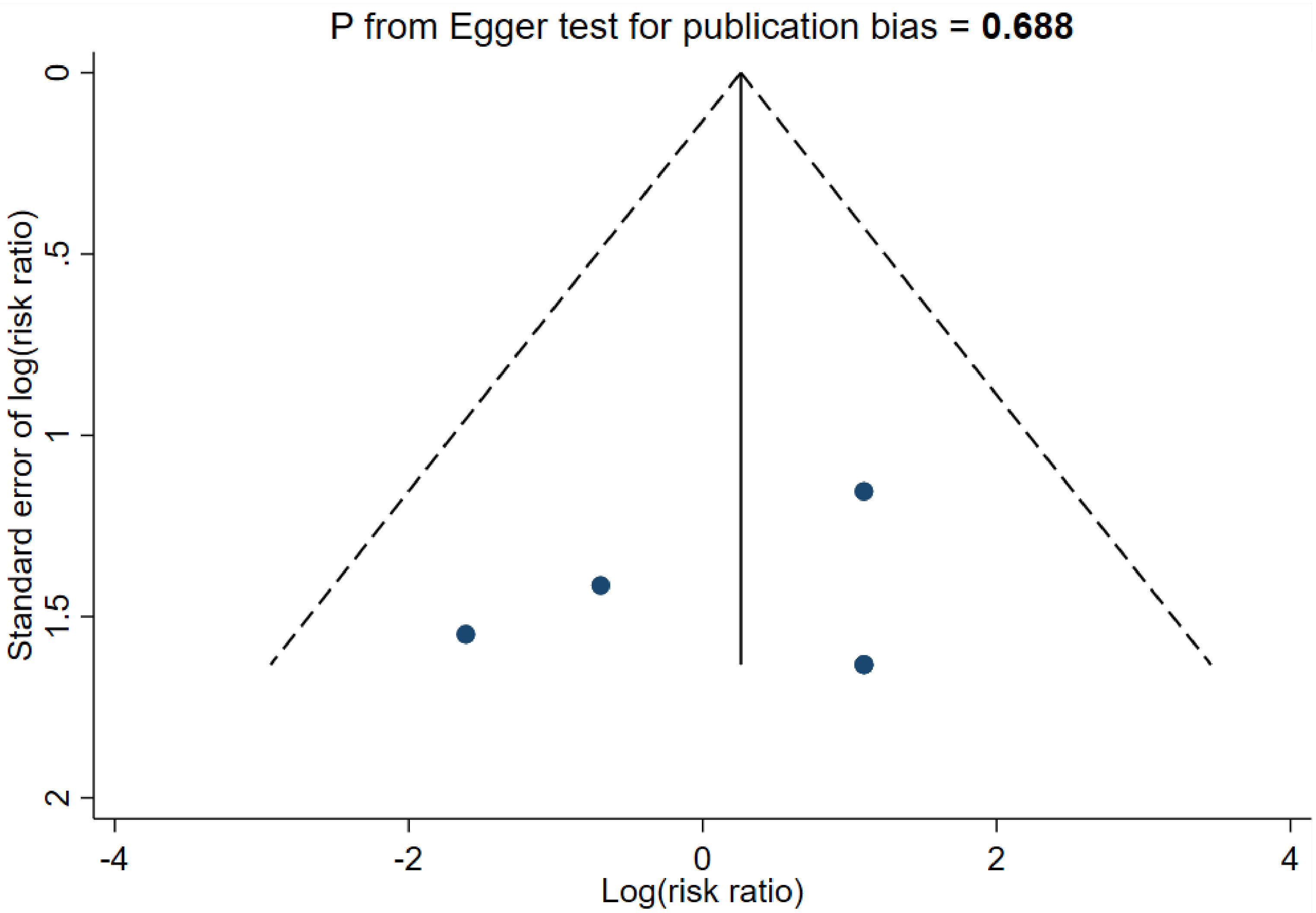

Figure S209 Funnel plot of Tricuspid valve incompetence

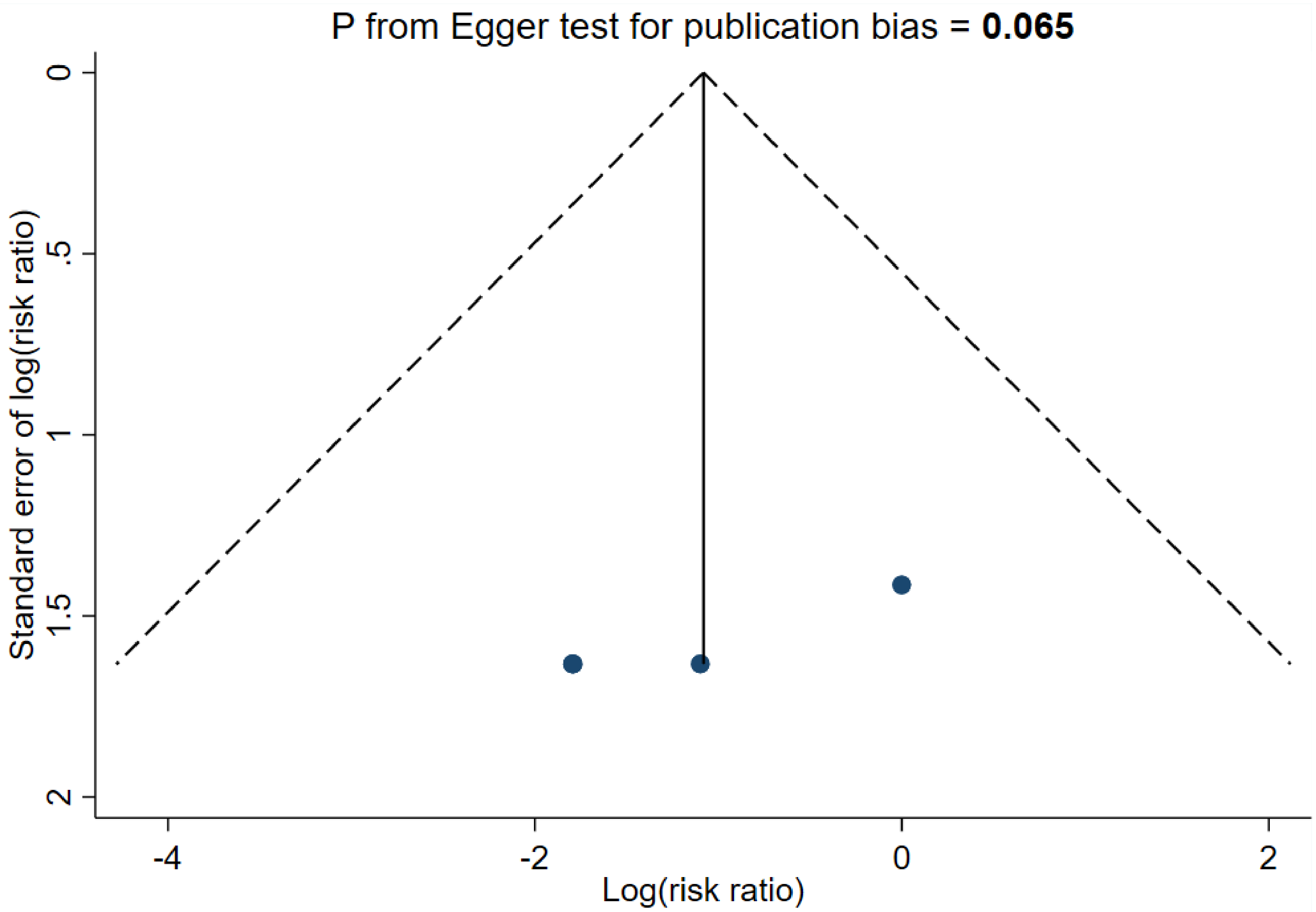

Figure S210 Funnel plot of Trifascicular block

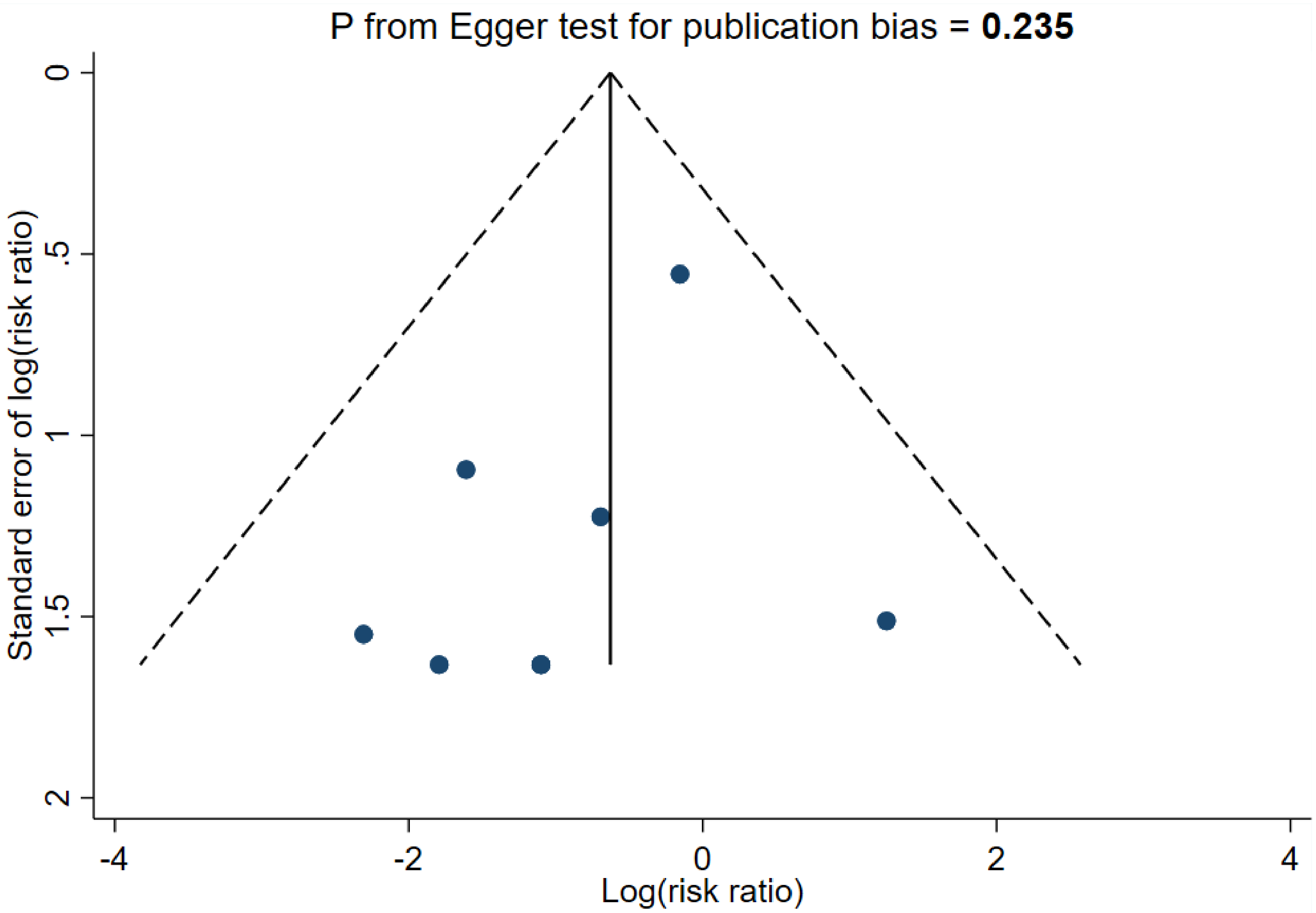

Figure S211 Funnel plot of Ventricular arrhythmia

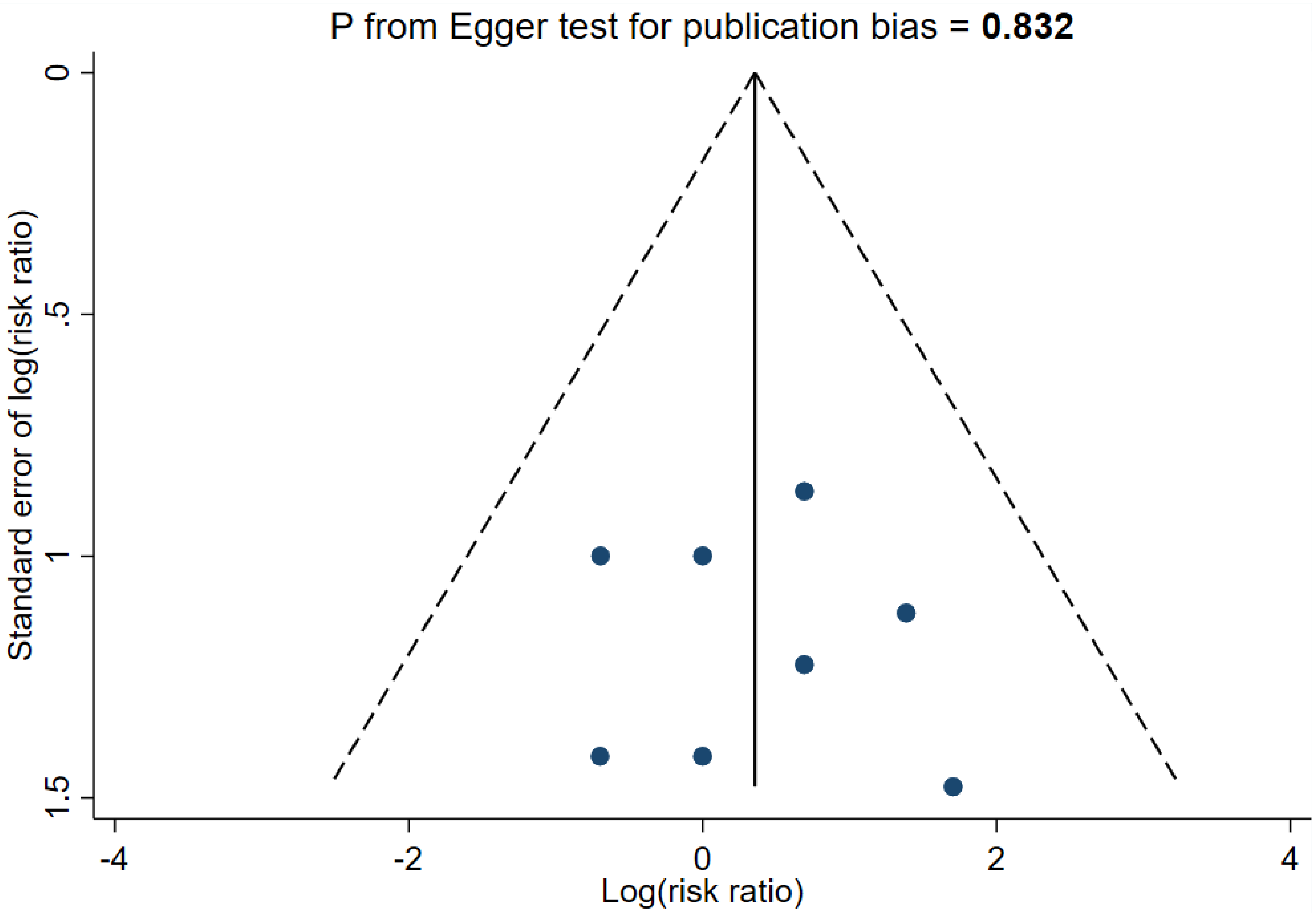

Figure S212 Funnel plot of Ventricular extrasystoles

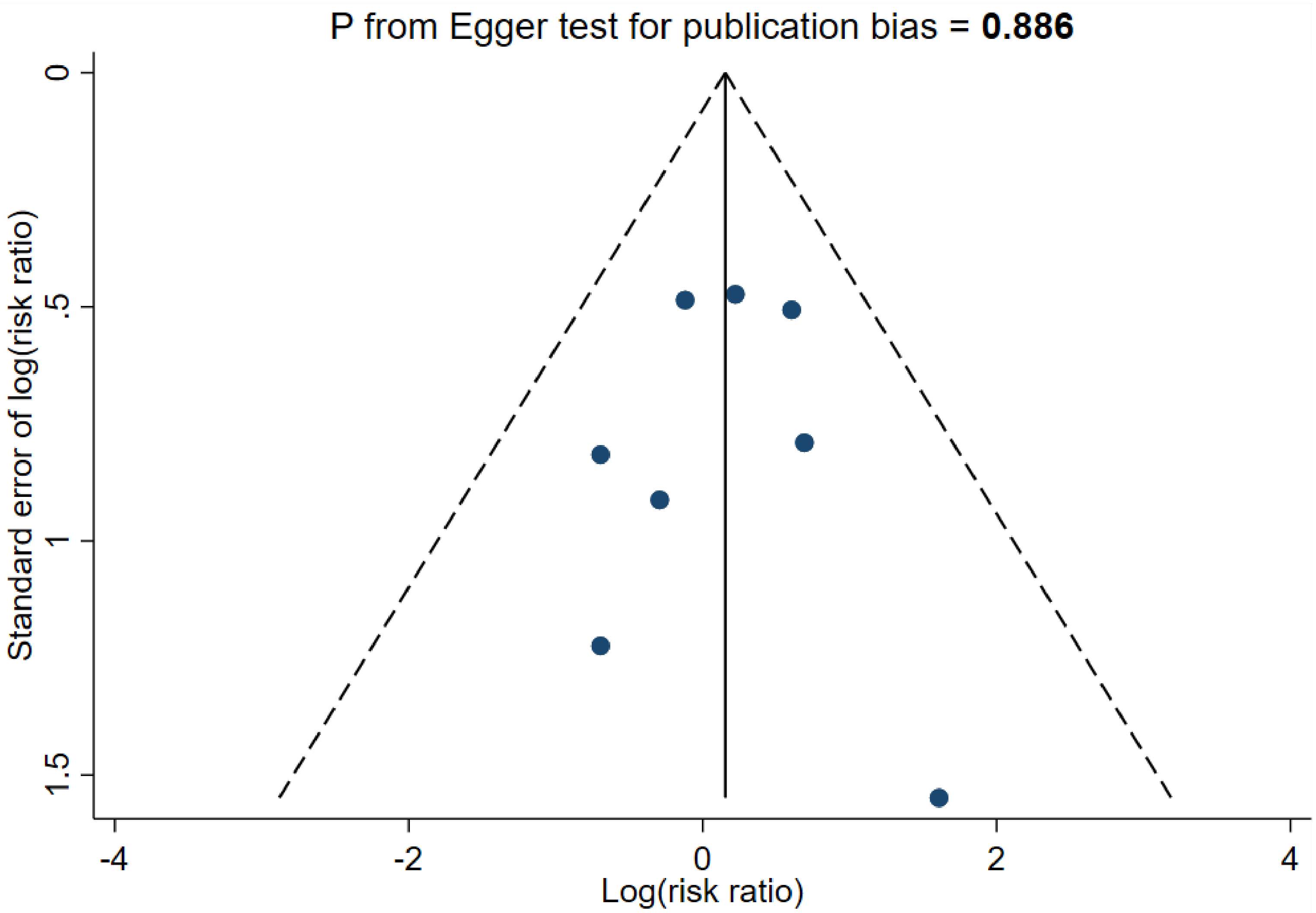

Figure S213 Funnel plot of Ventricular fibrillation

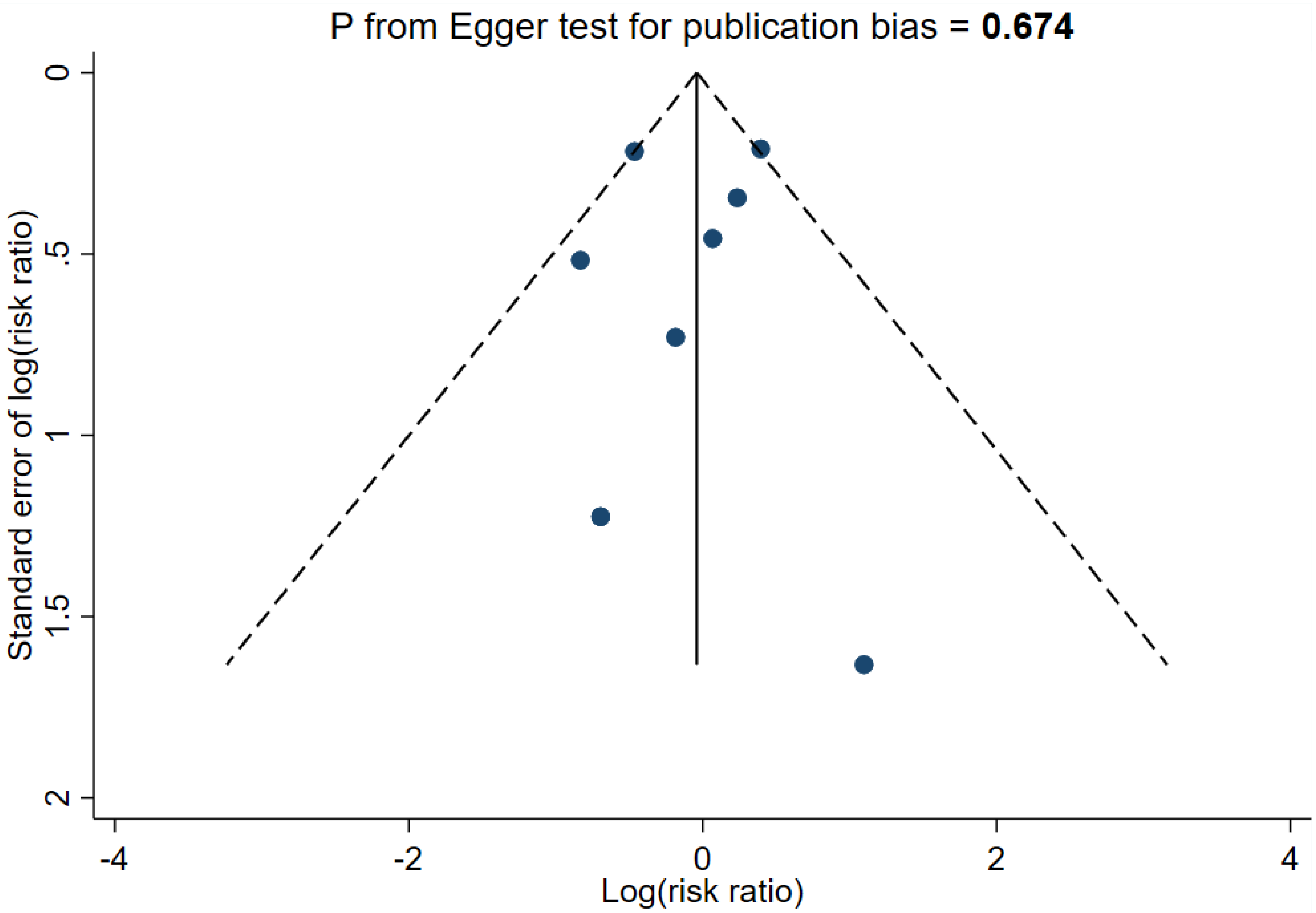

Figure S214 Funnel plot of Ventricular tachycardia

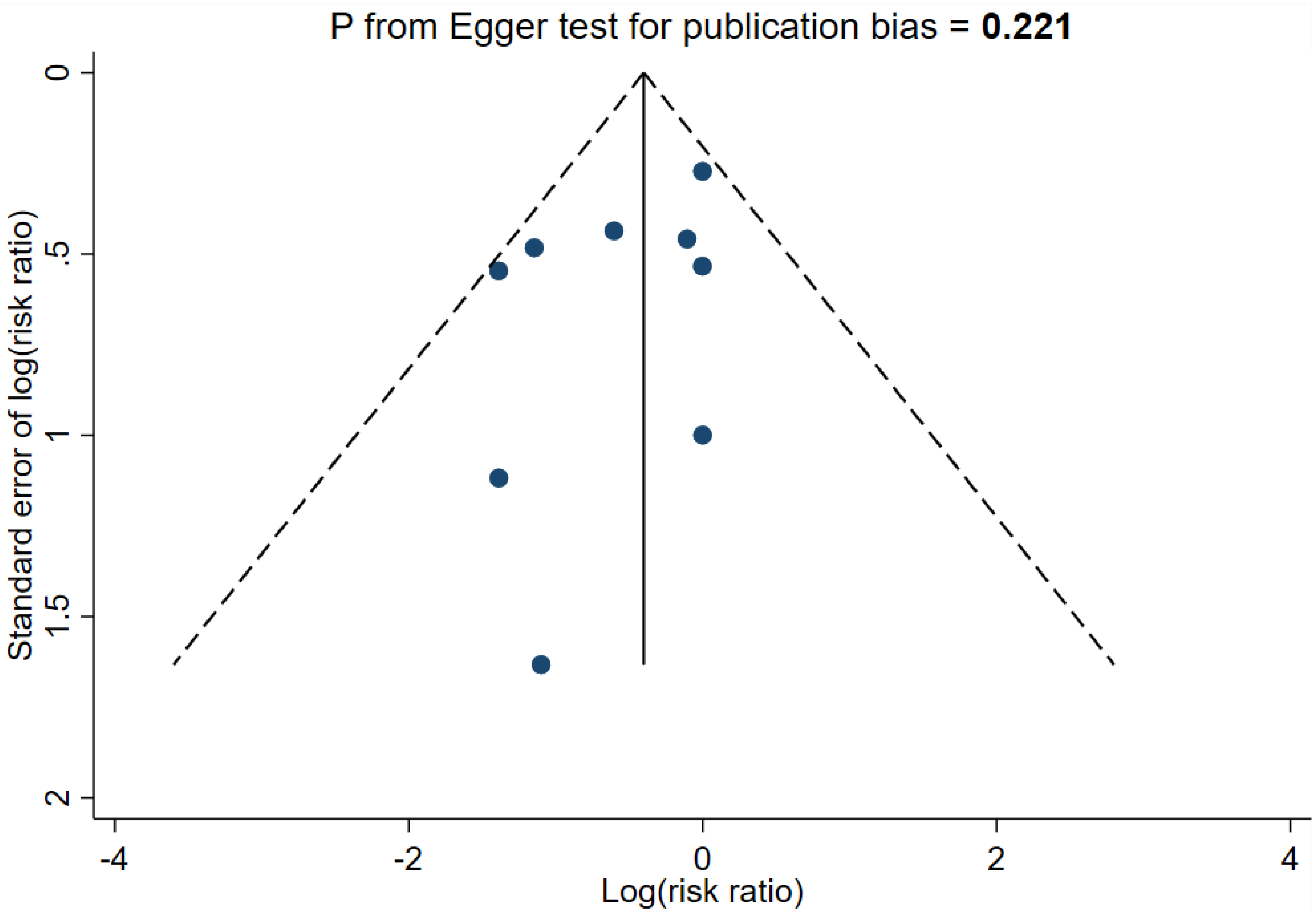

Figure S215 Funnel plot of Hypertension

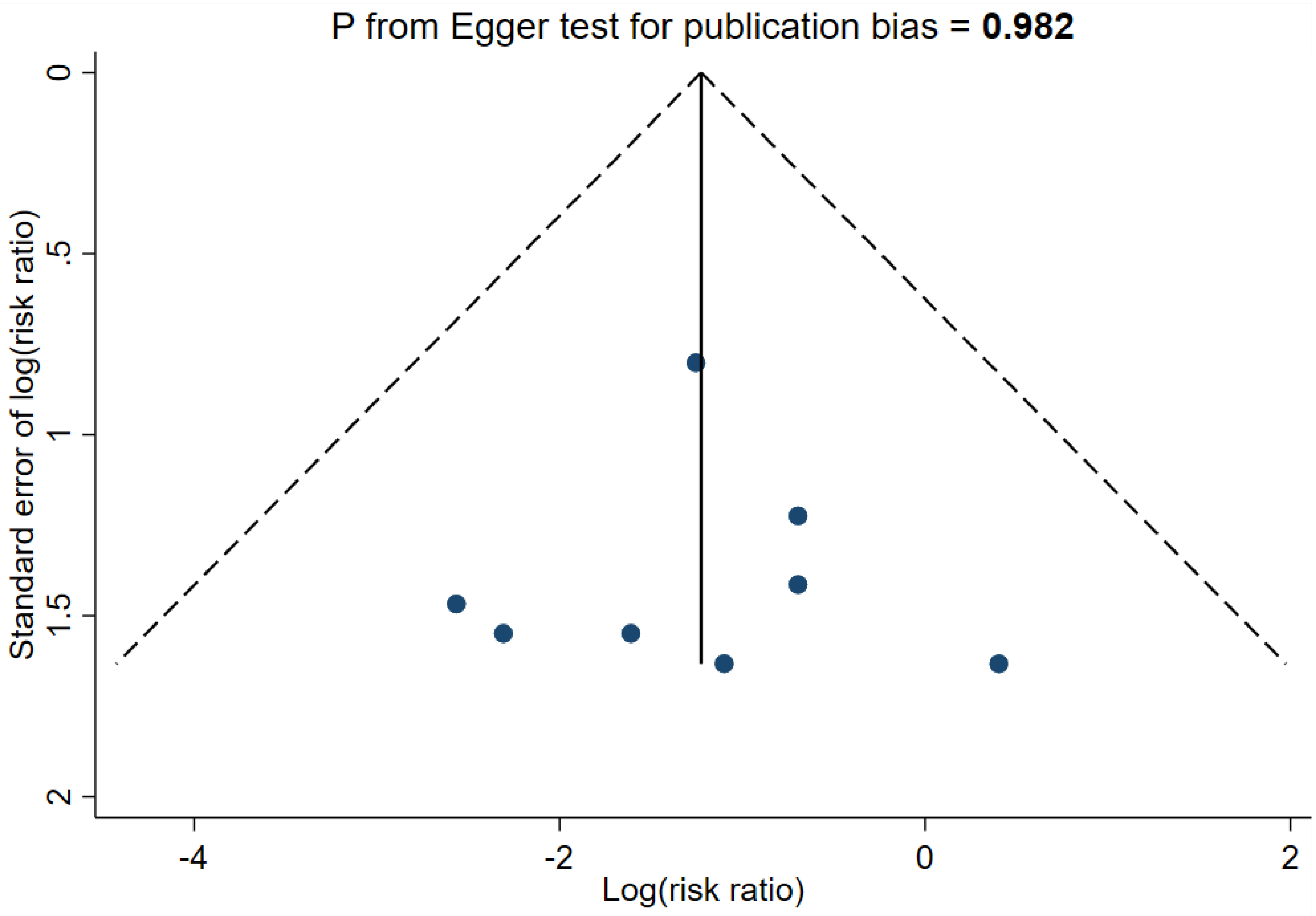

Figure S216 Funnel plot of Hypertensive emergency

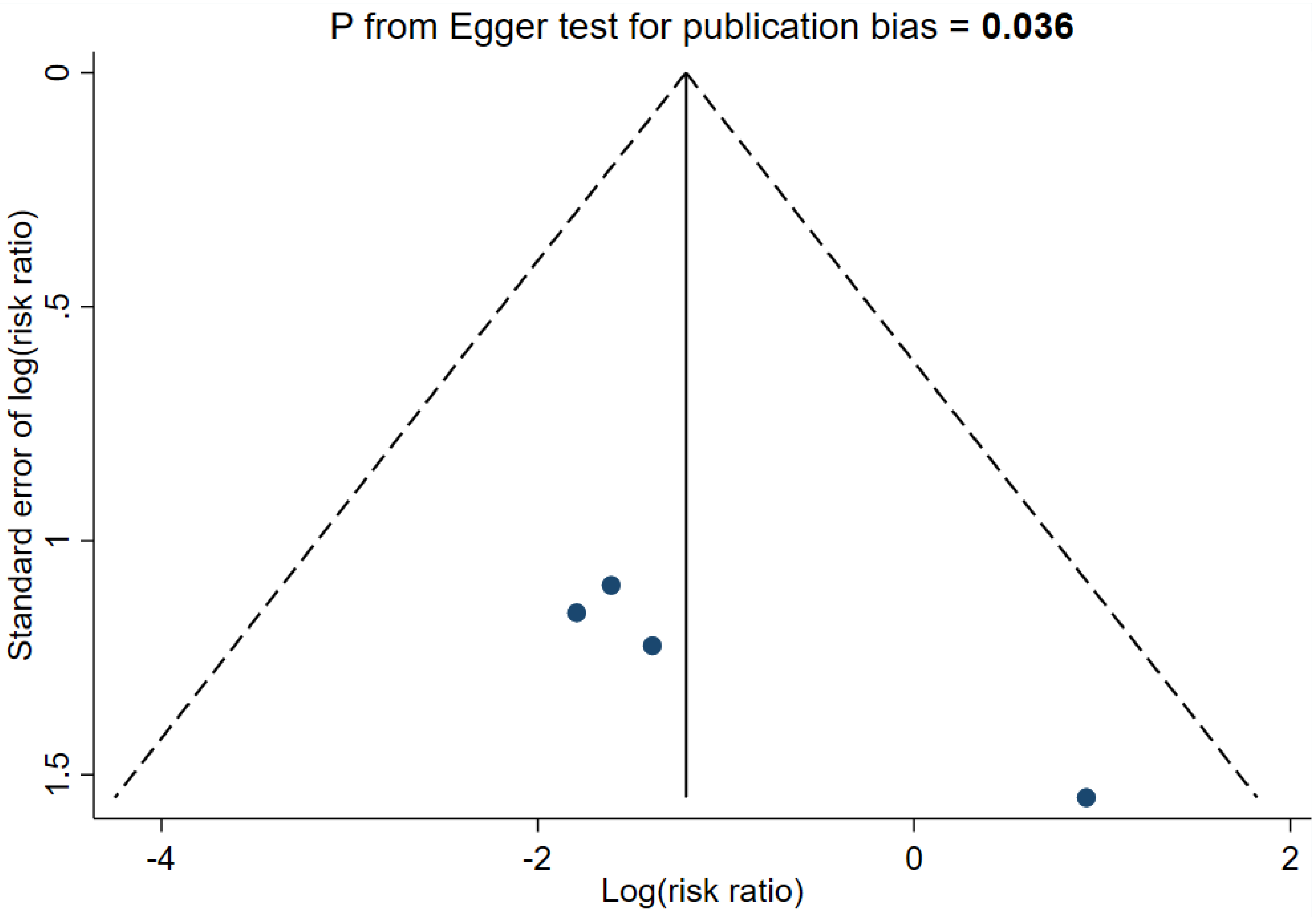

Figure S217 Funnel plot of Varicose vein

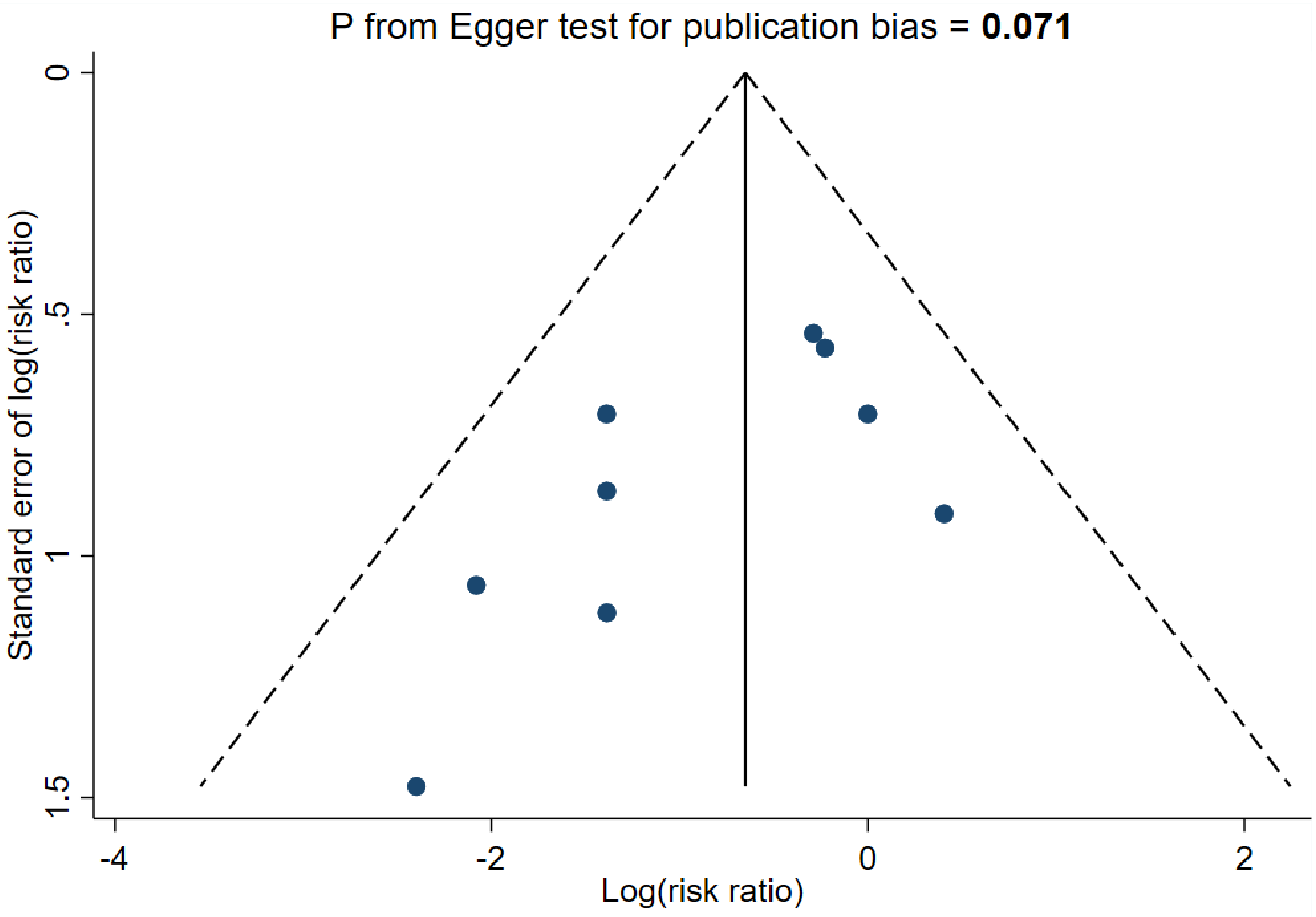

Figure S218 Funnel plot of Acute pulmonary oedema

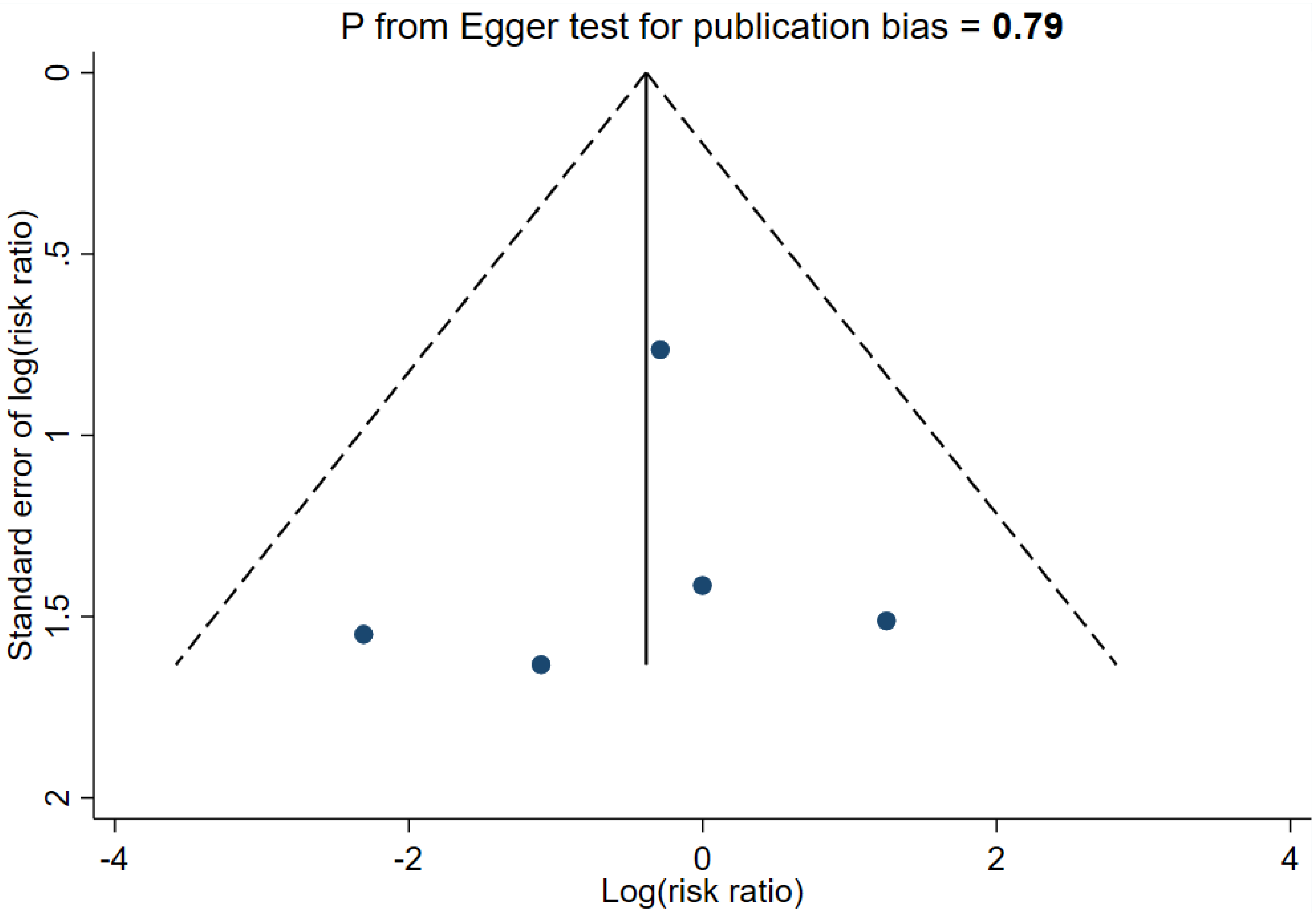

Figure S219 Funnel plot of Acute respiratory distress syndrome

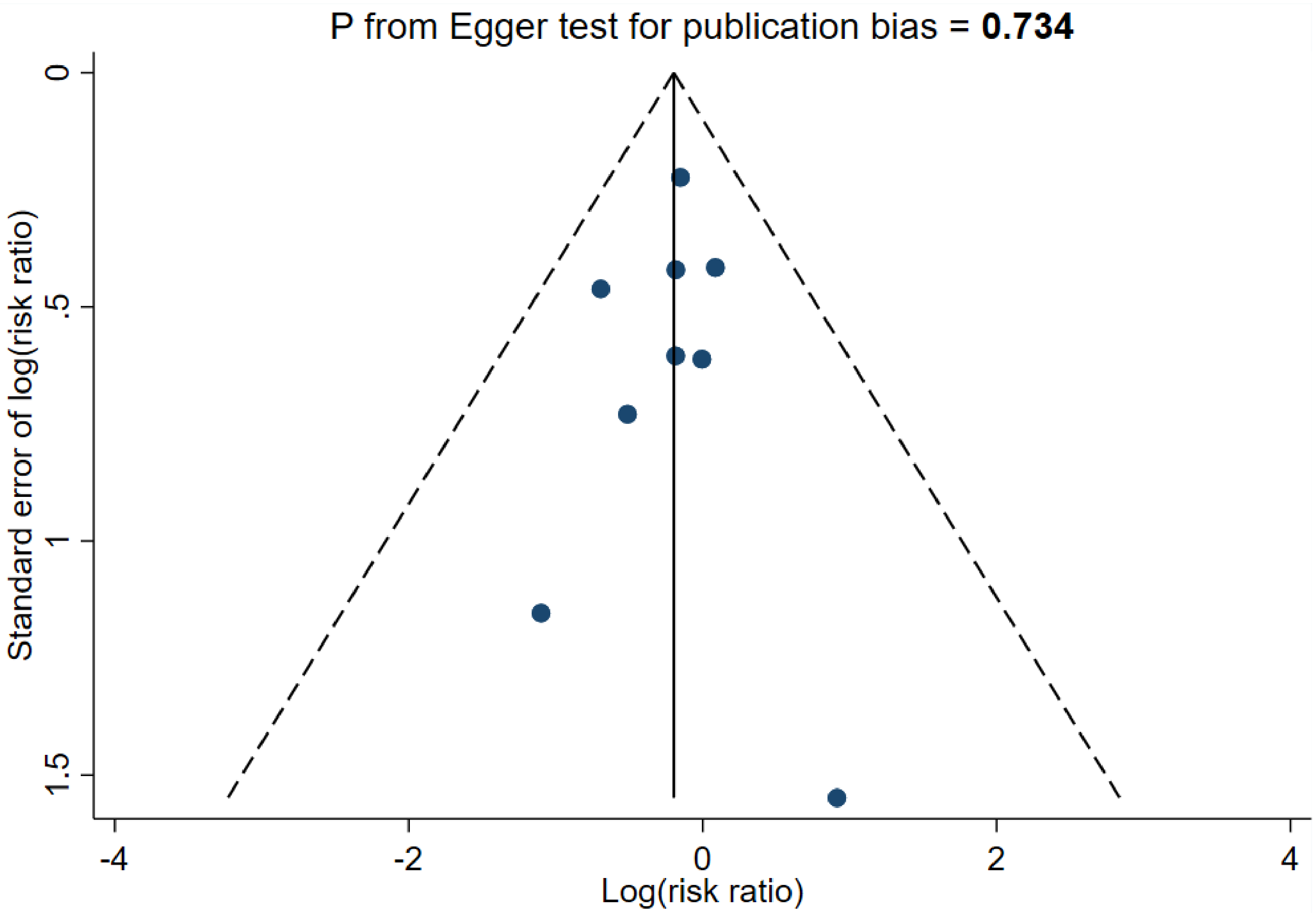

Figure S220 Funnel plot of Acute respiratory failure

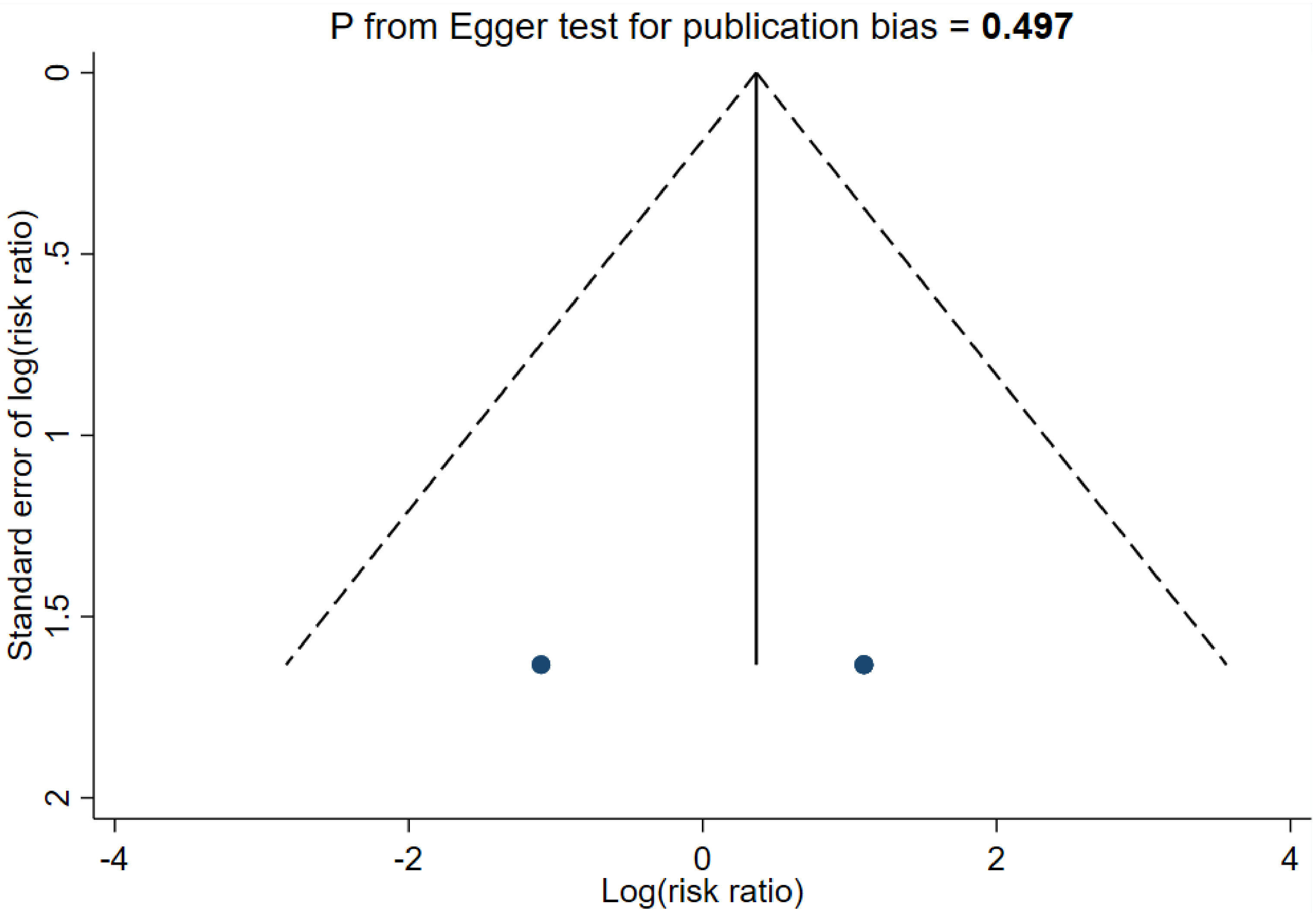

Figure S221 Funnel plot of Aspiration

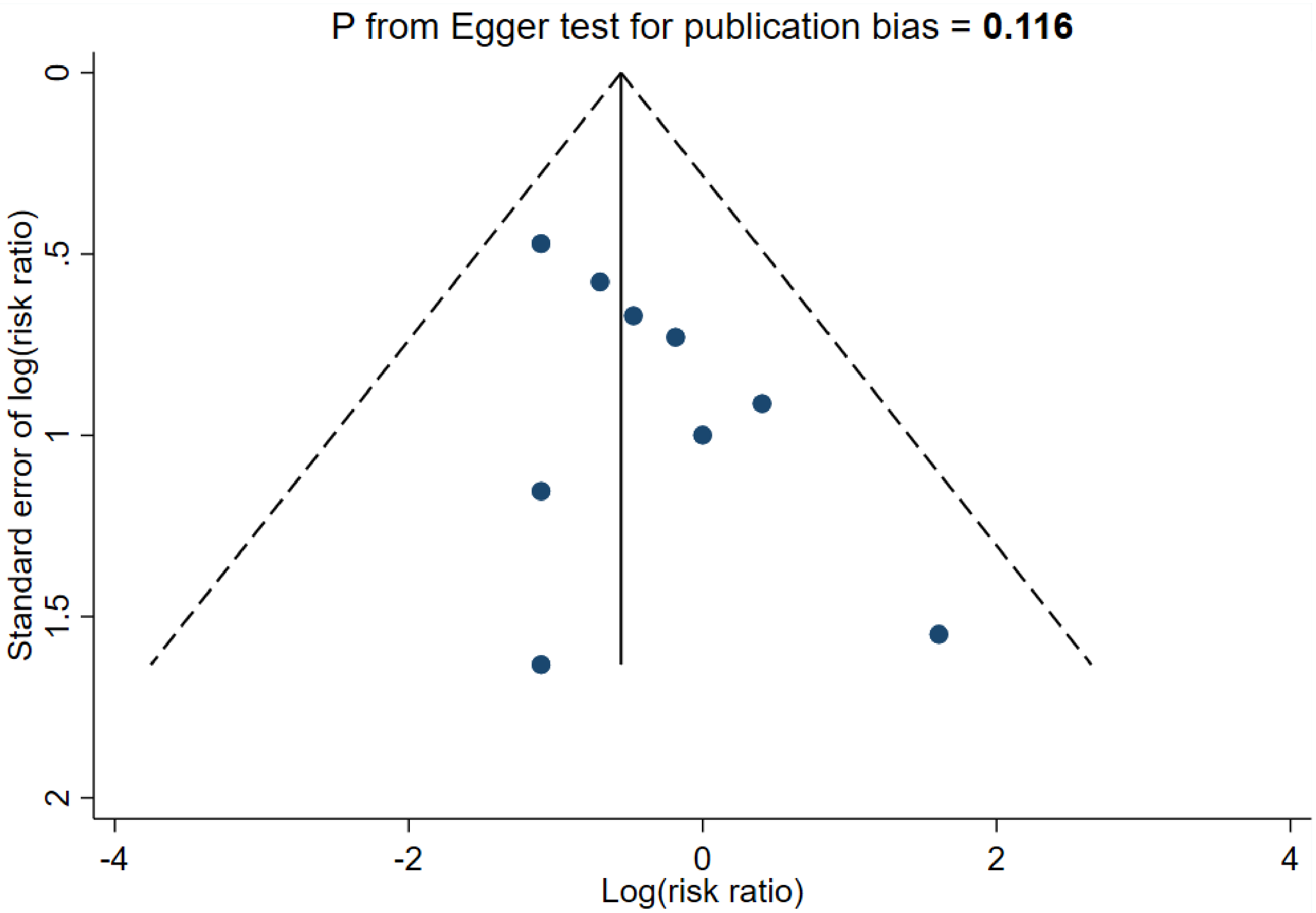

Figure S222 Funnel plot of Asthma

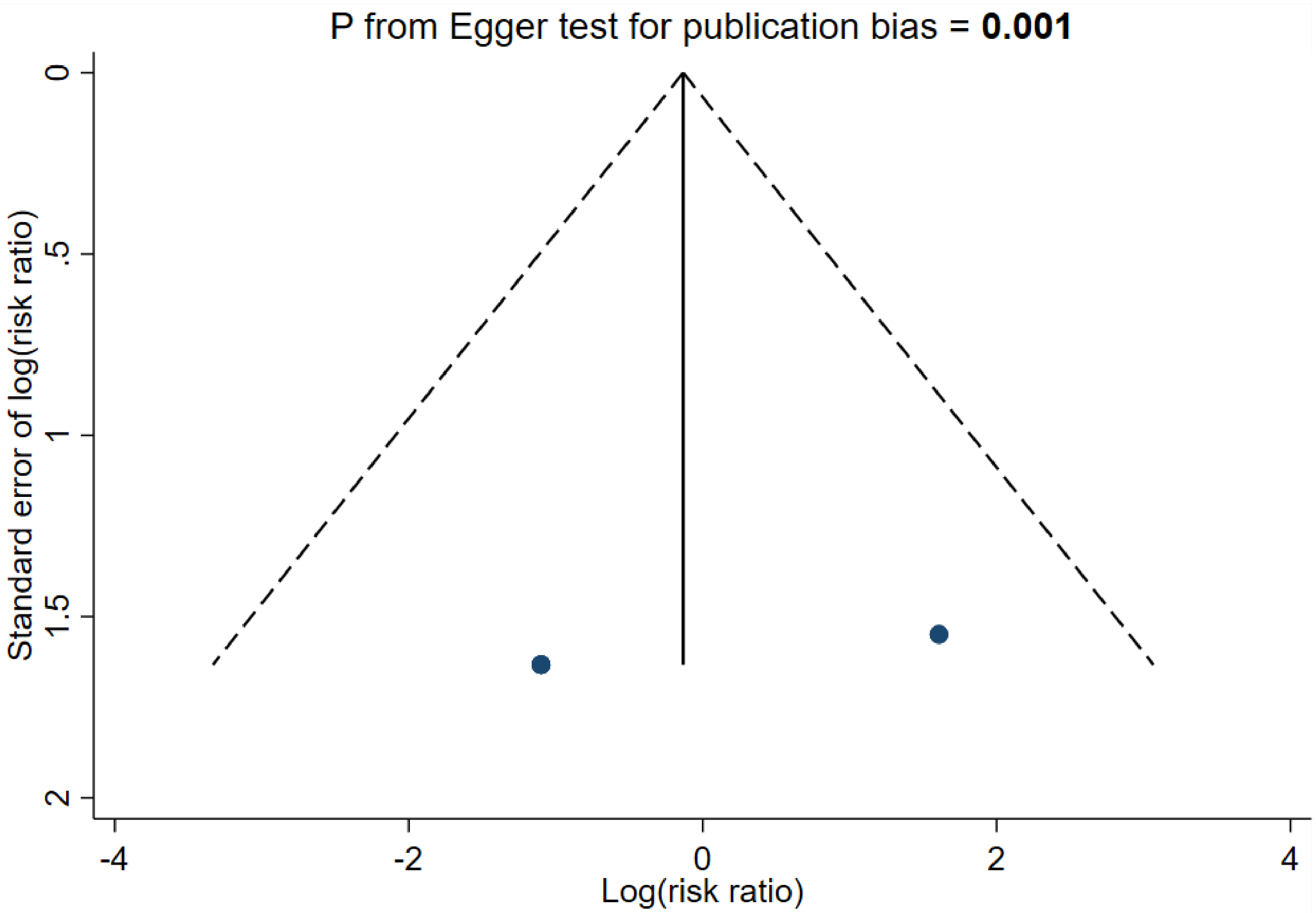

Figure S223 Funnel plot of Asthmatic crisis

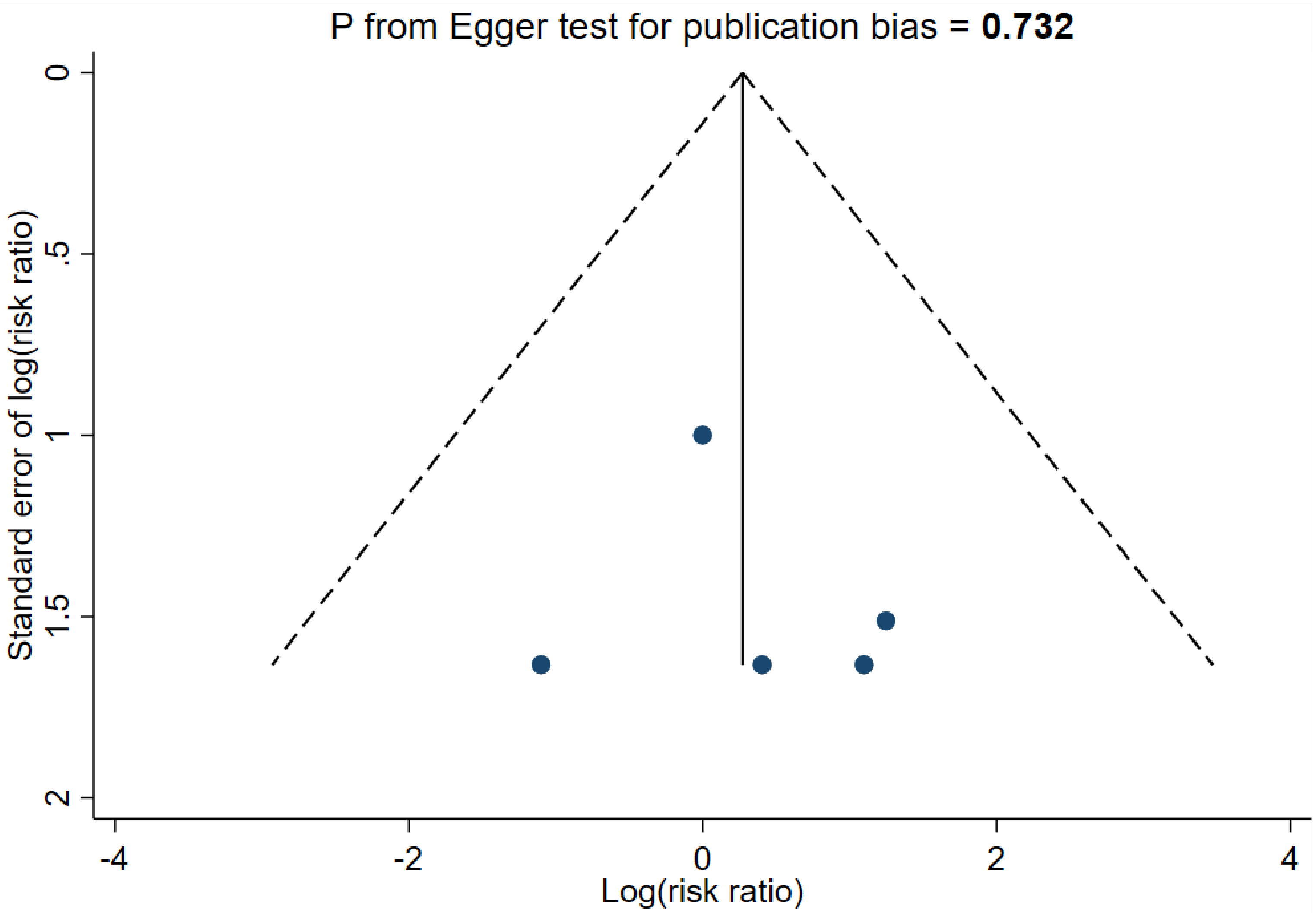

Figure S224 Funnel plot of Atelectasis

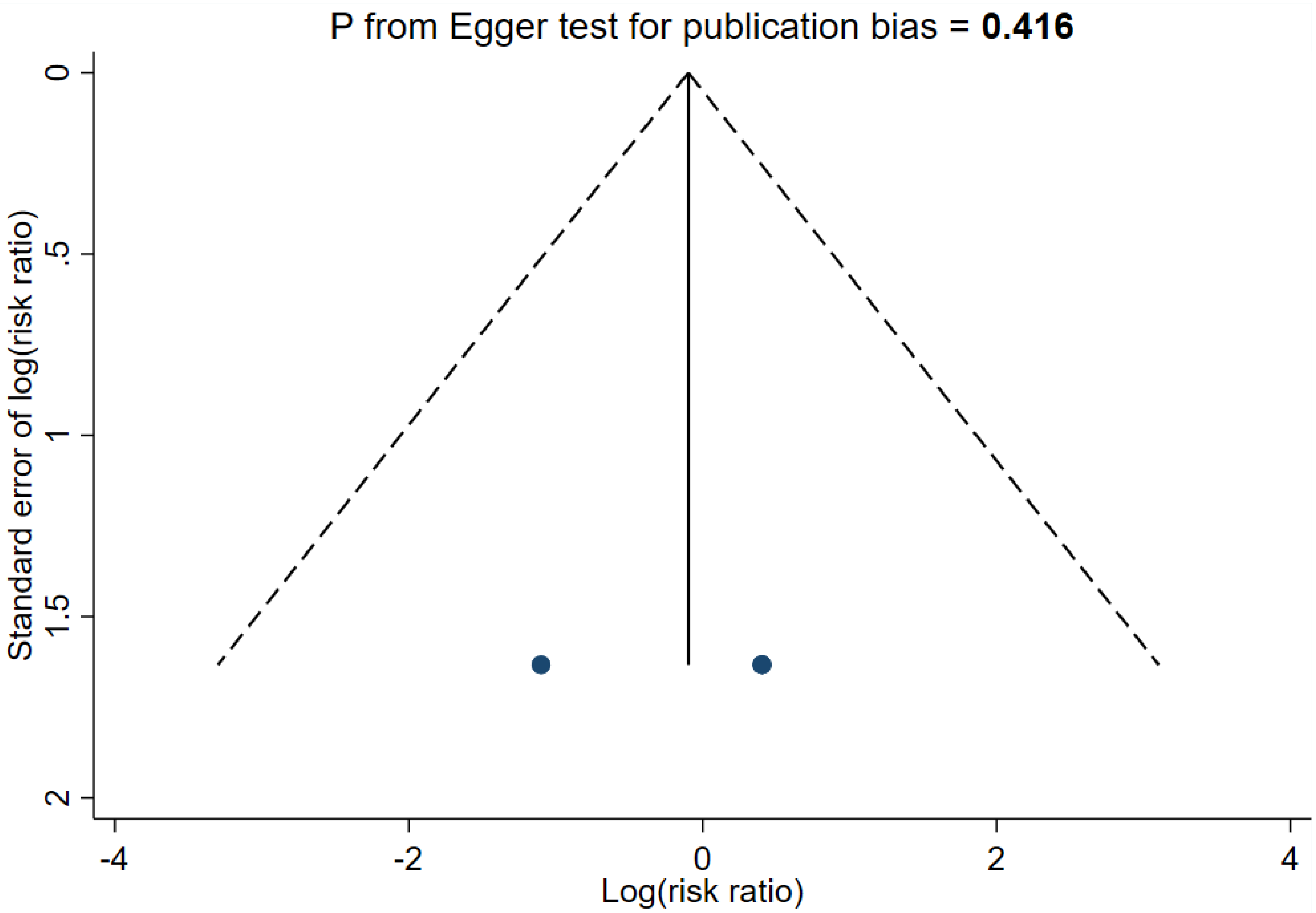

Figure S225 Funnel plot of Bronchial hyperreactivity

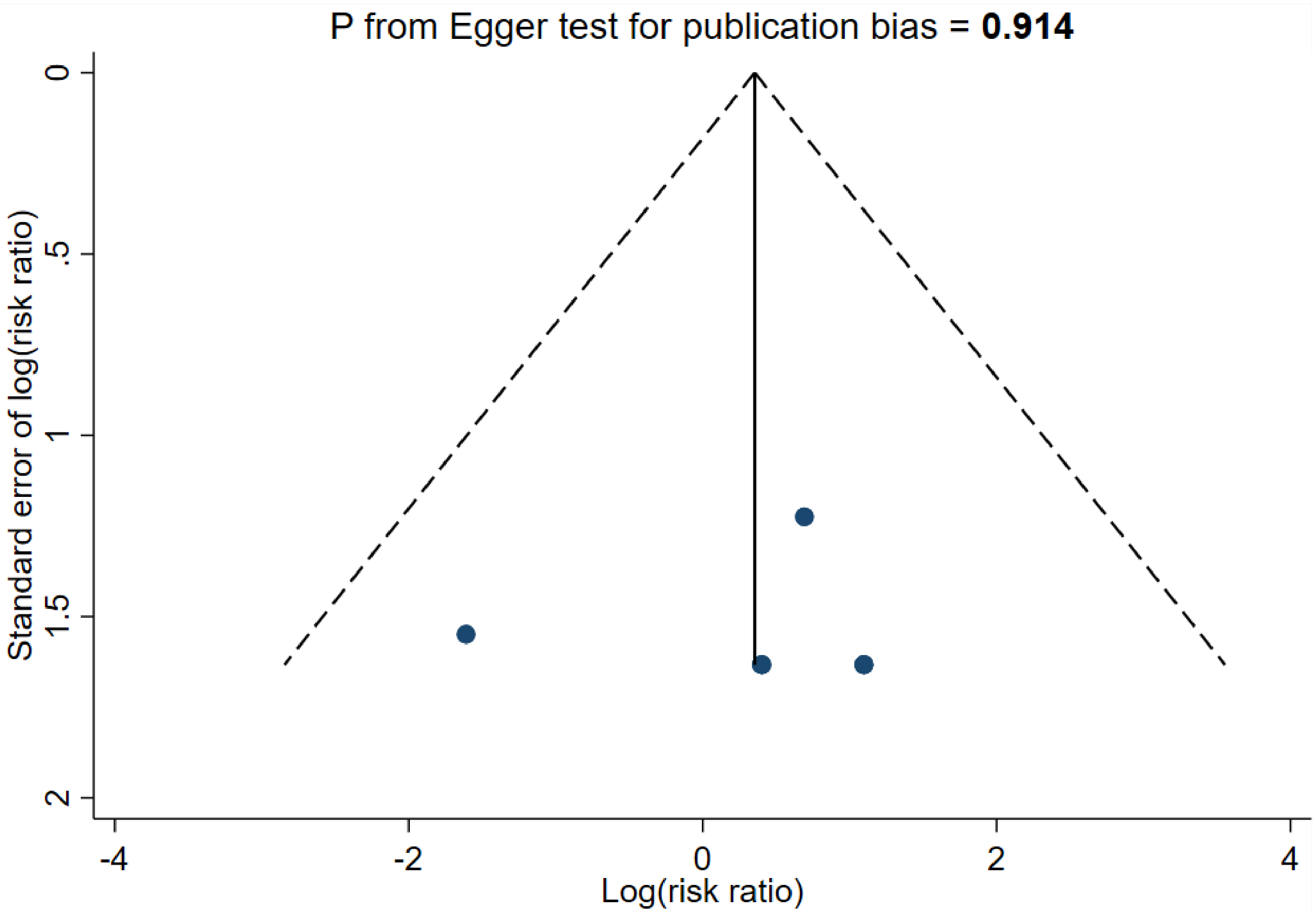

Figure S226 Funnel plot of Bronchiectasis

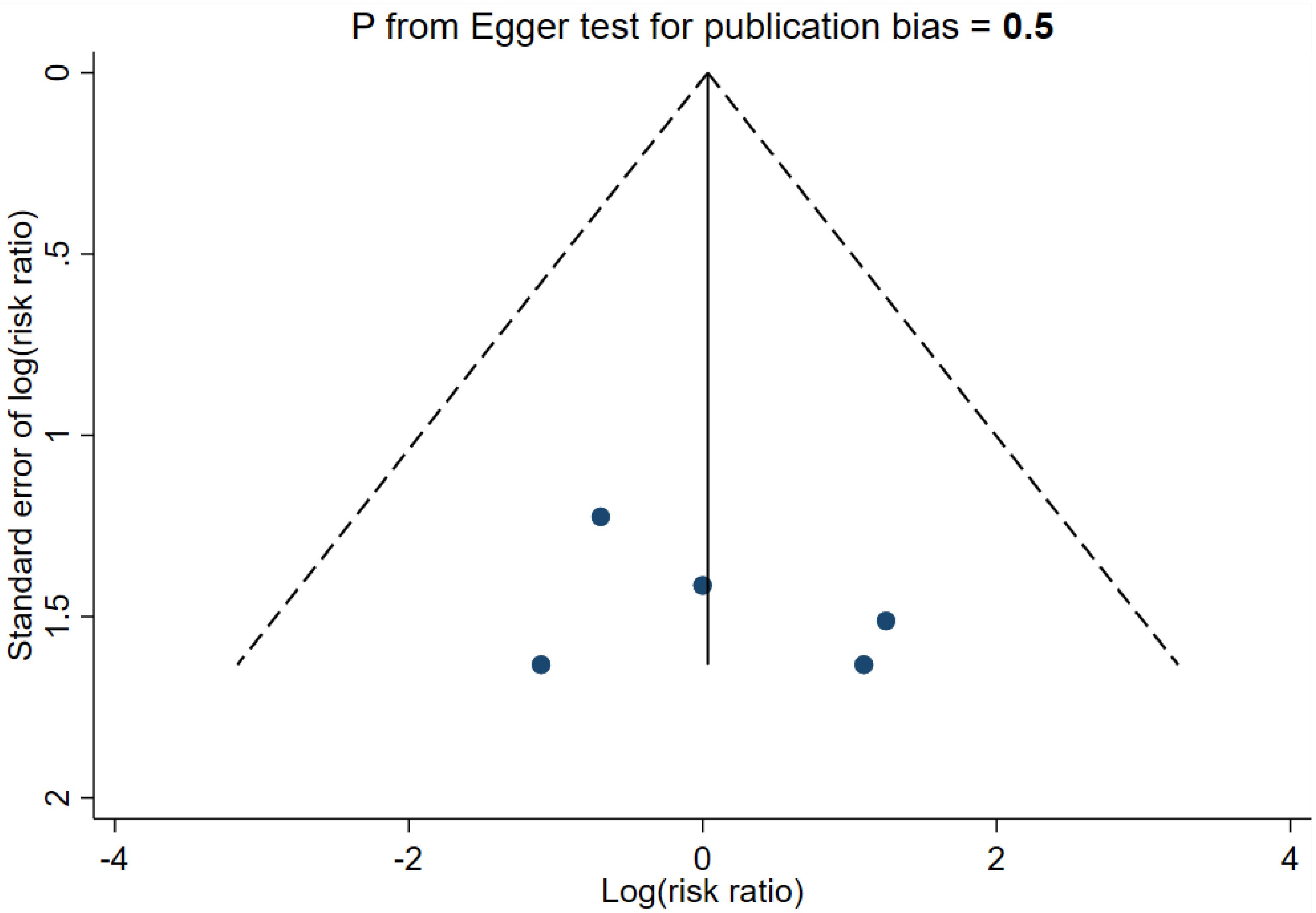

Figure S227 Funnel plot of Bronchitis chronic

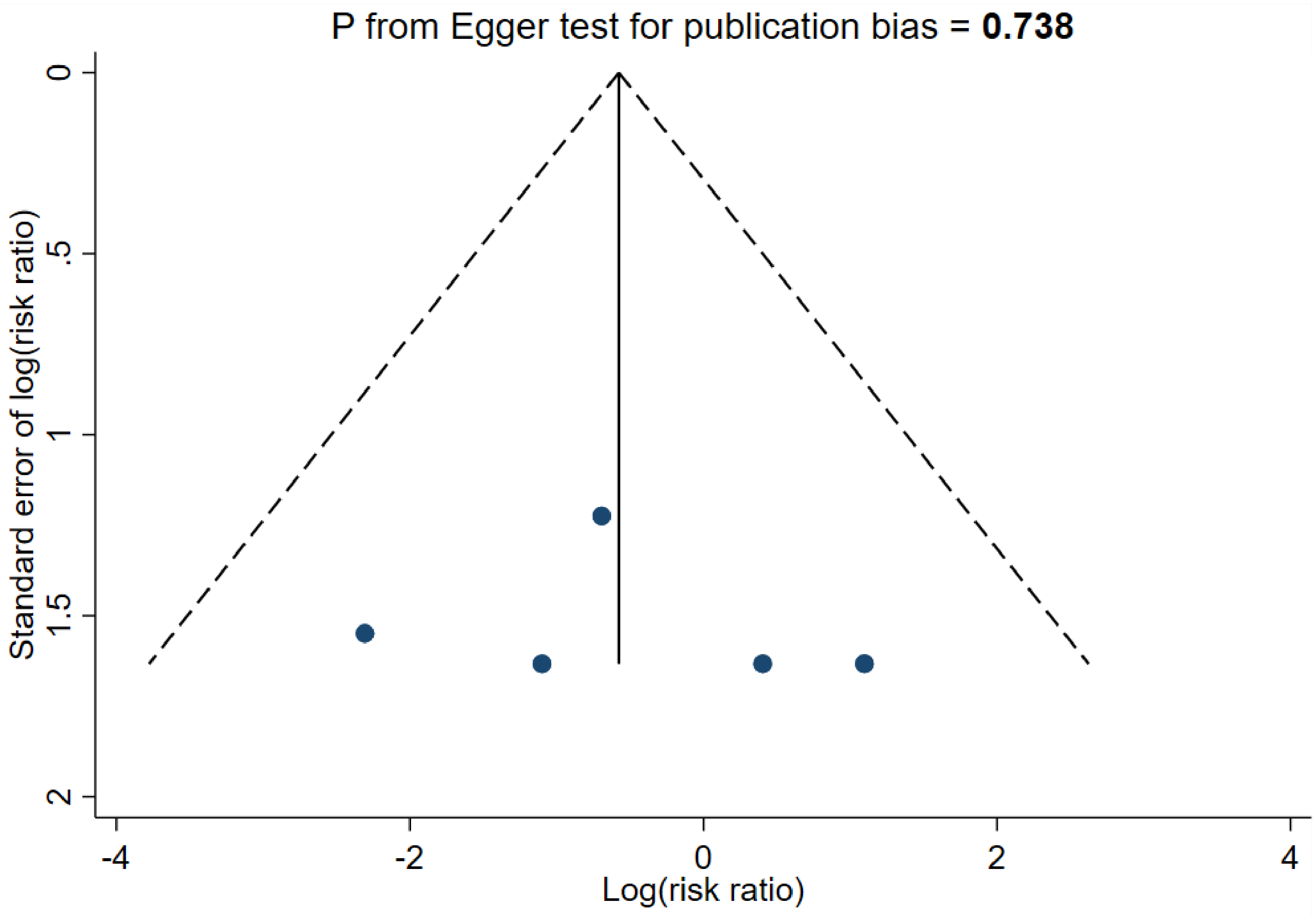

Figure S228 Funnel plot of Bronchospasm

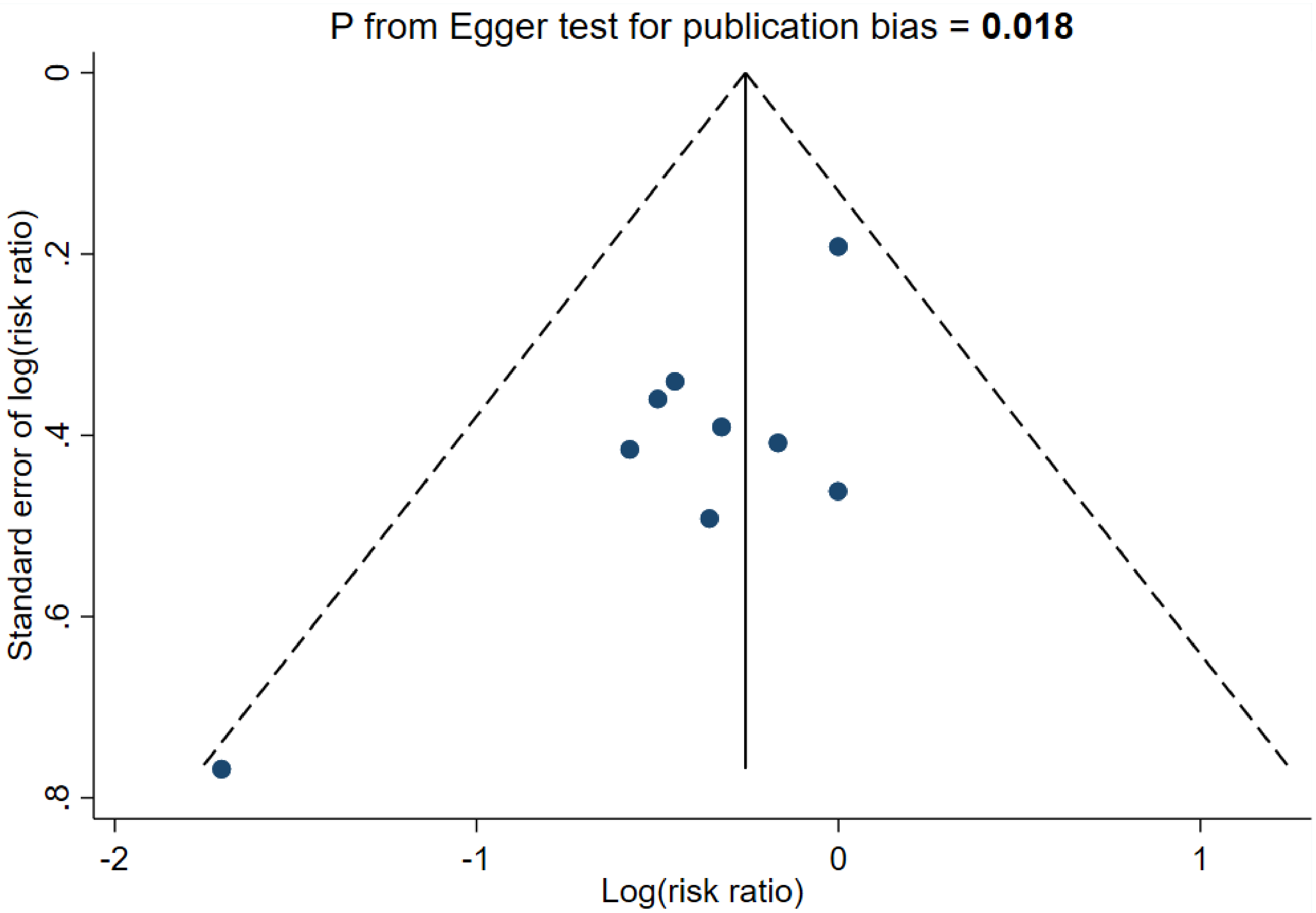

Figure S229 Funnel plot of Chronic obstructive pulmonary disease

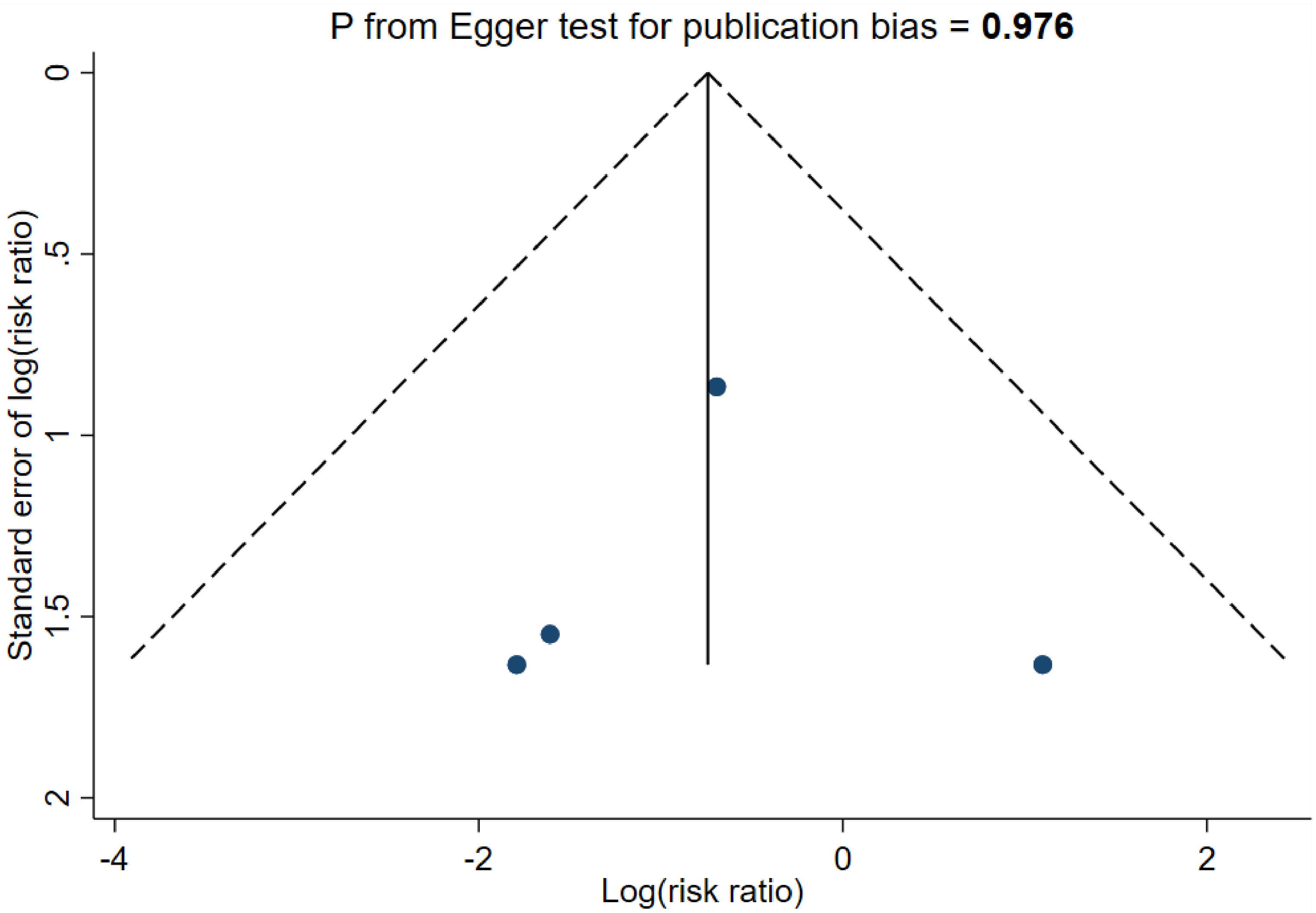

Figure S230 Funnel plot of Chronic respiratory failure

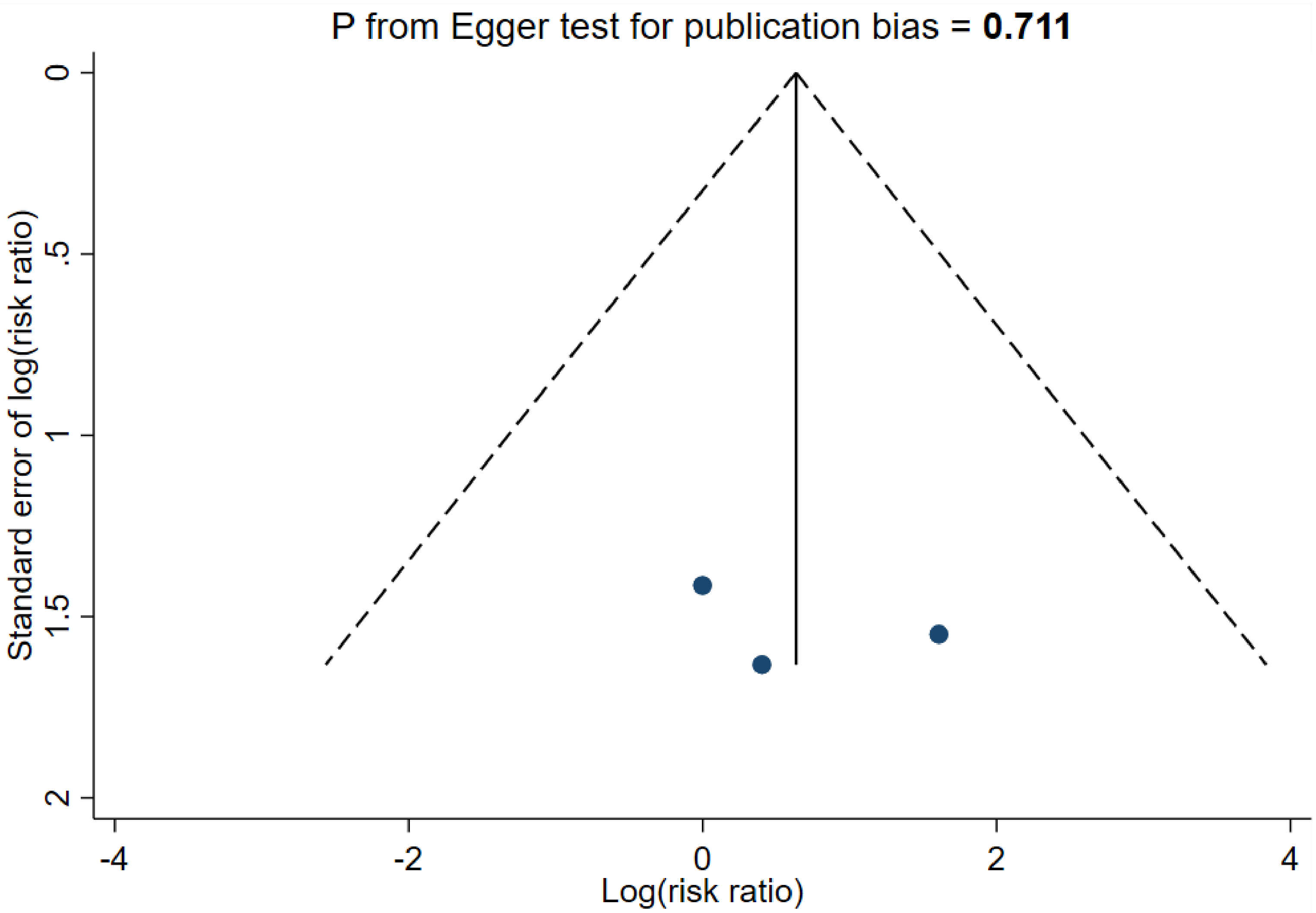

Figure S231 Funnel plot of Cough

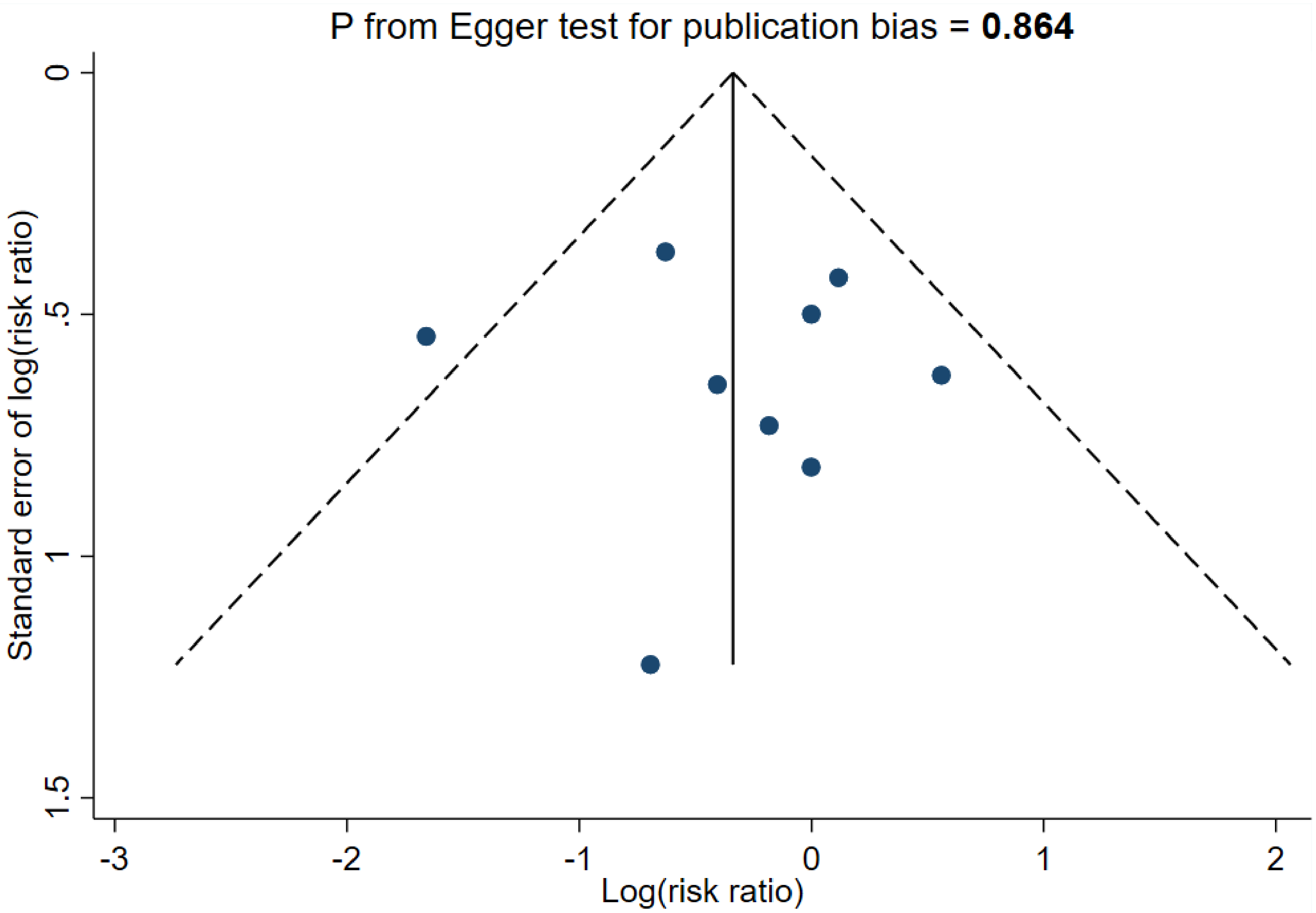

Figure S232 Funnel plot of Dyspnoea

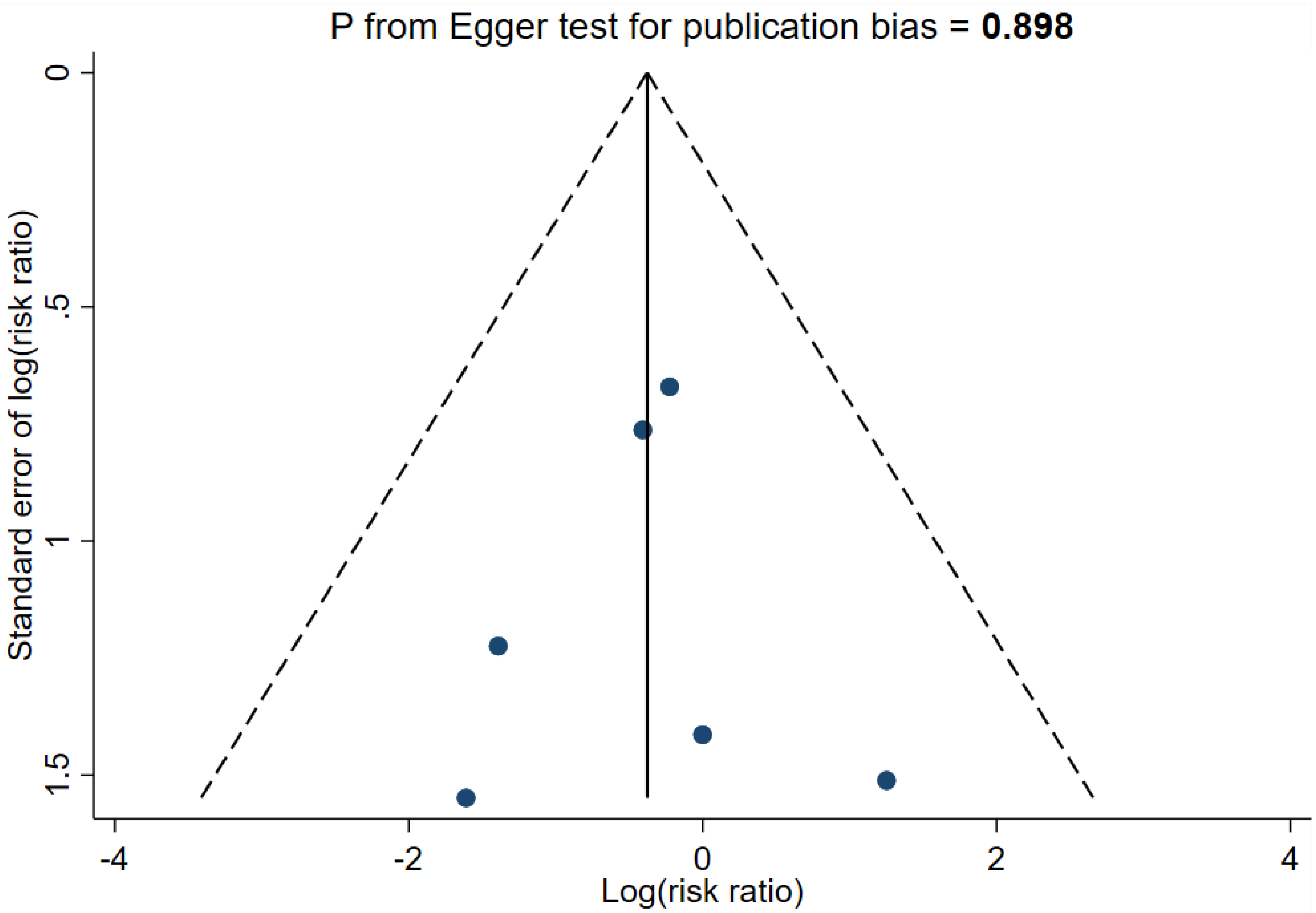

Figure S233 Funnel plot of Dyspnoea exertional

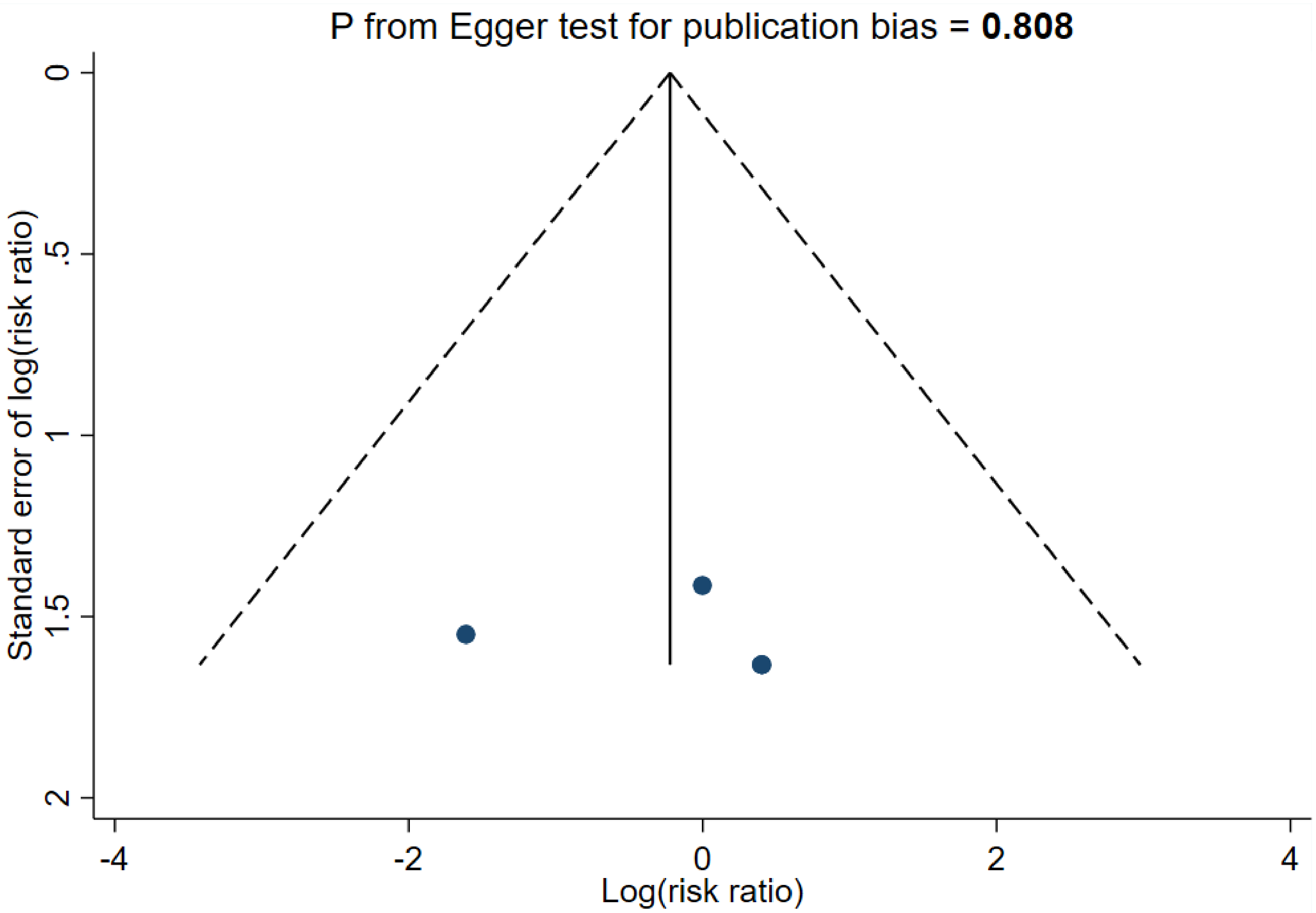

Figure S234 Funnel plot of Emphysema

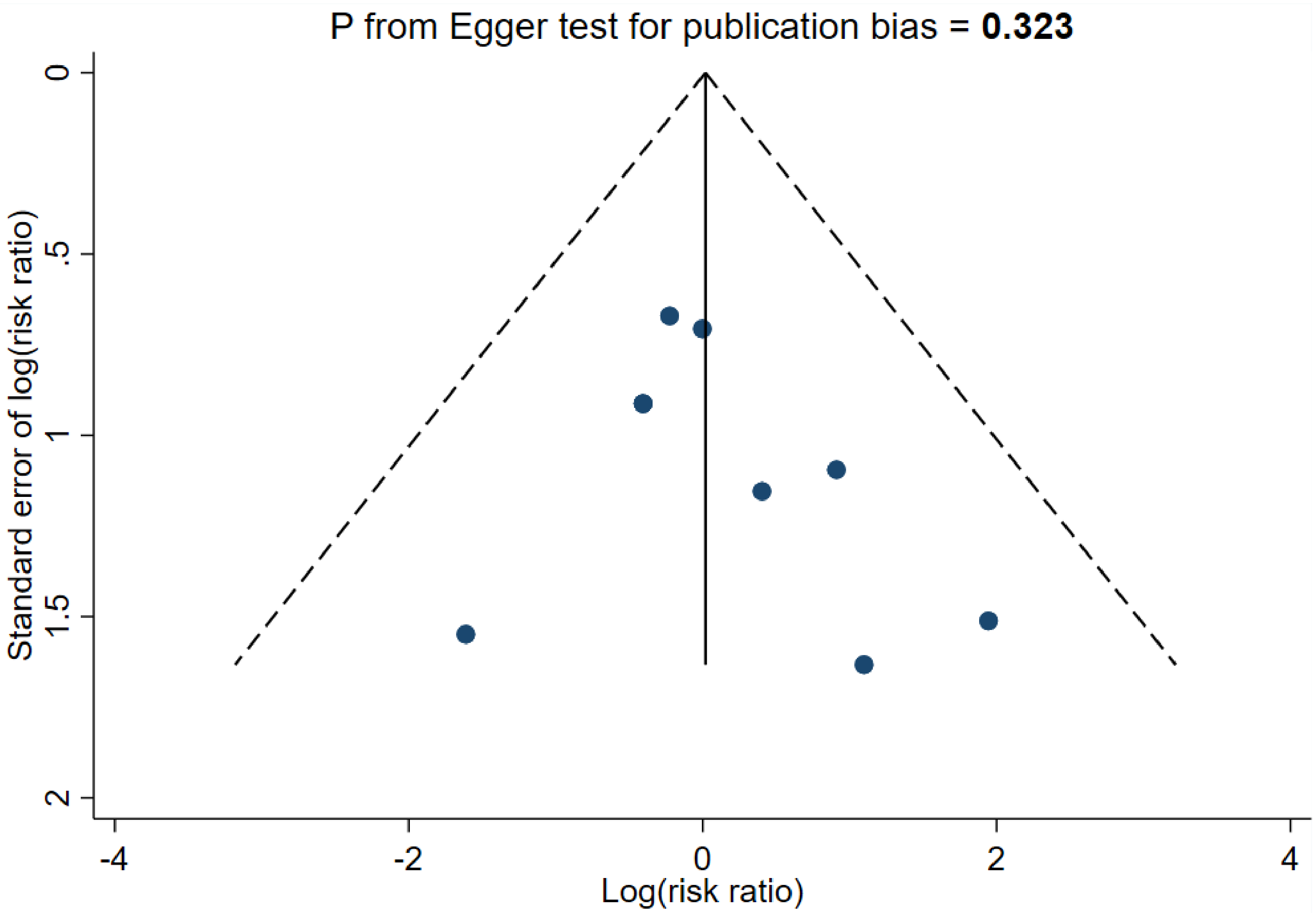

Figure S235 Funnel plot of Epistaxis

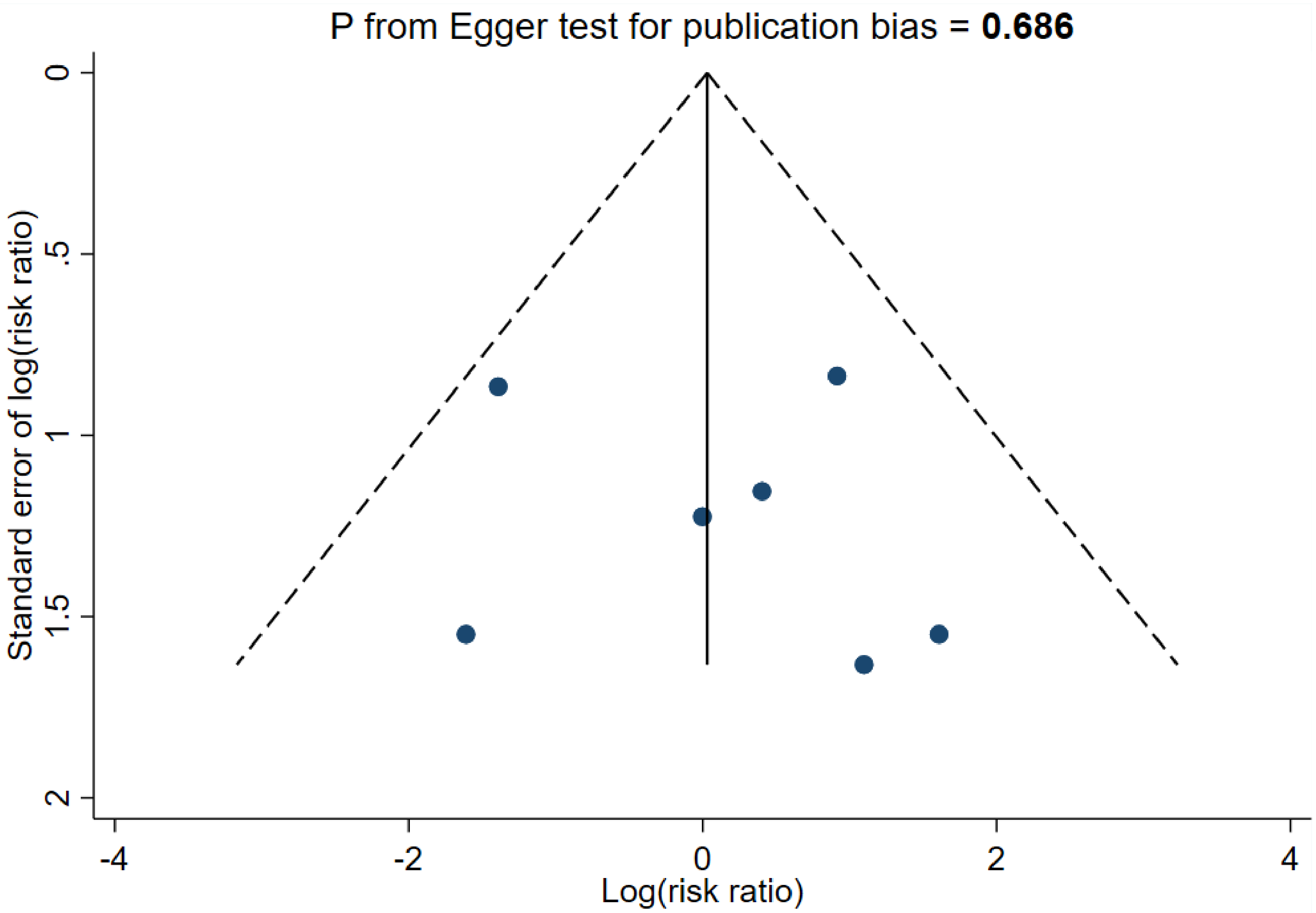

Figure S236 Funnel plot of Haemoptysis

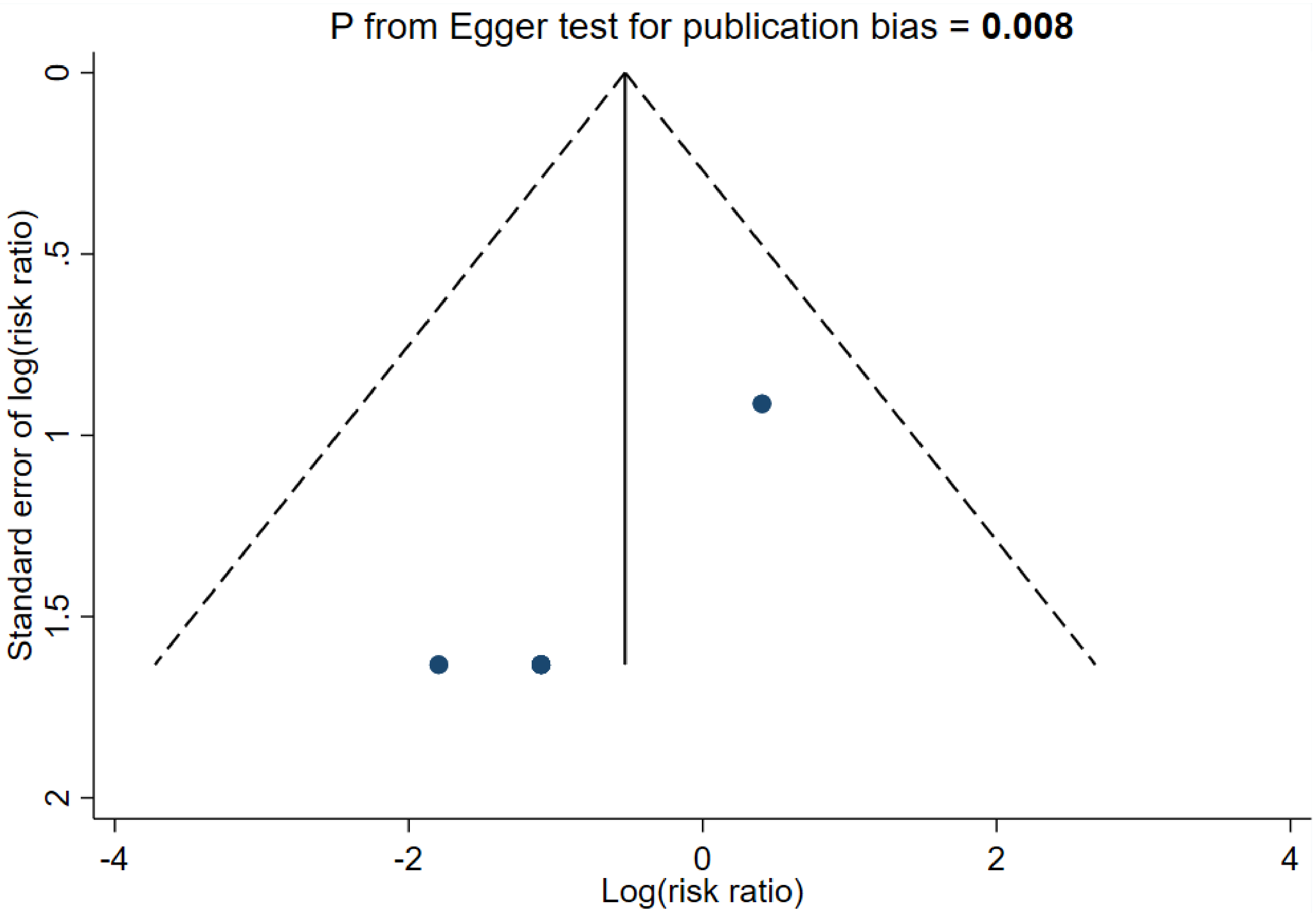

Figure S237 Funnel plot of Haemothorax

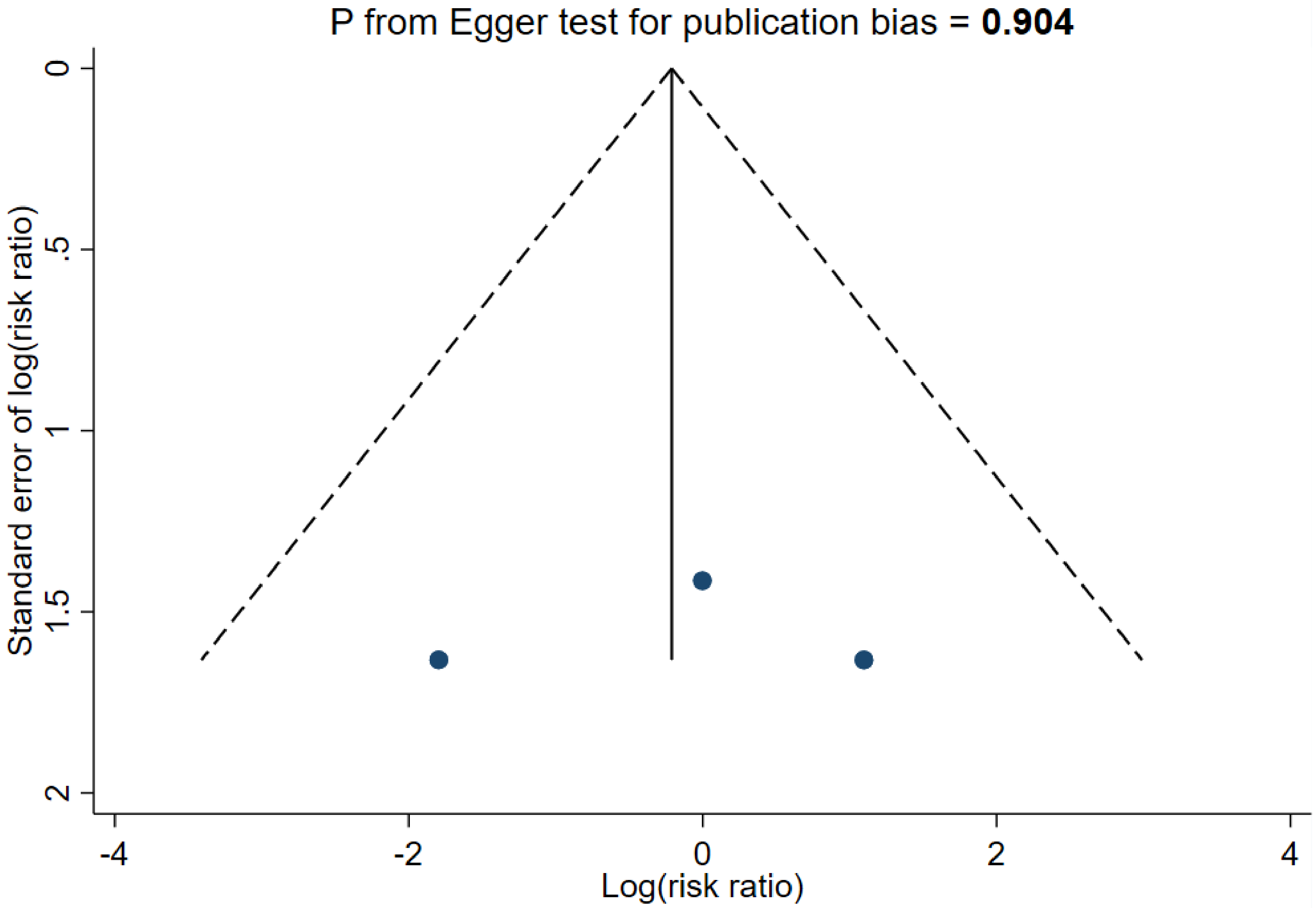

Figure S238 Funnel plot of Hydrothorax

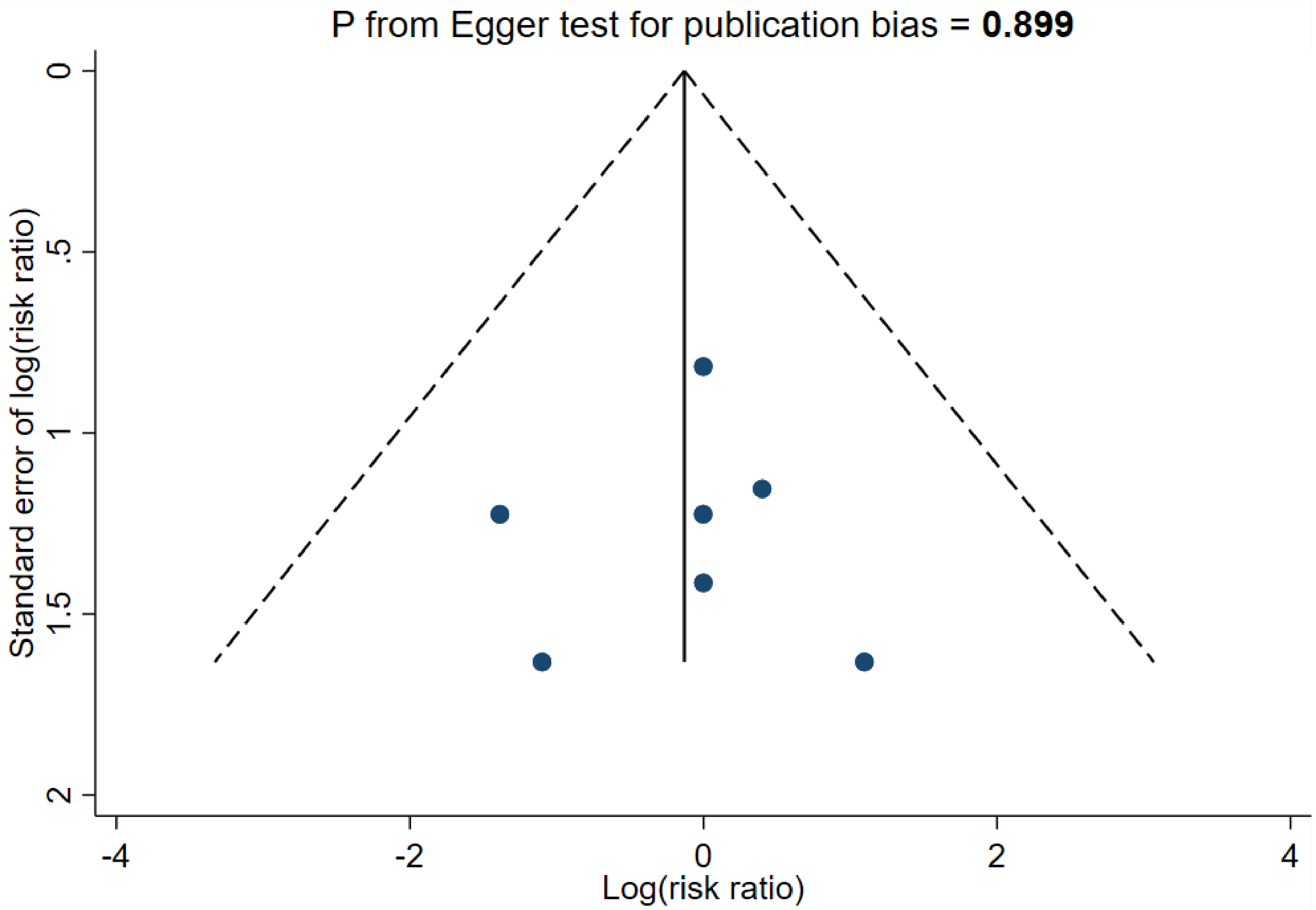

Figure S239 Funnel plot of Hypoxia

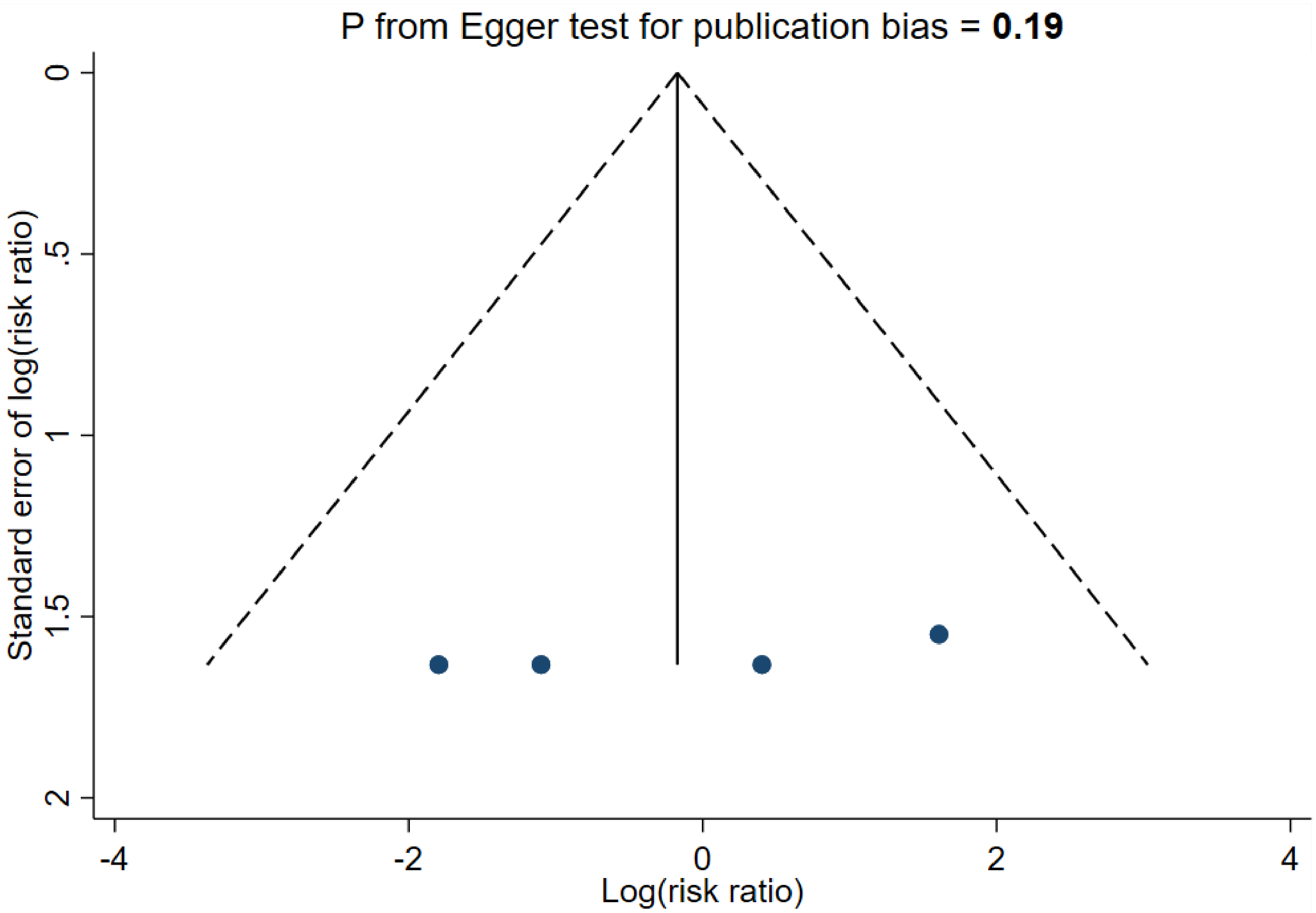

Figure S240 Funnel plot of Idiopathic pulmonary fibrosis

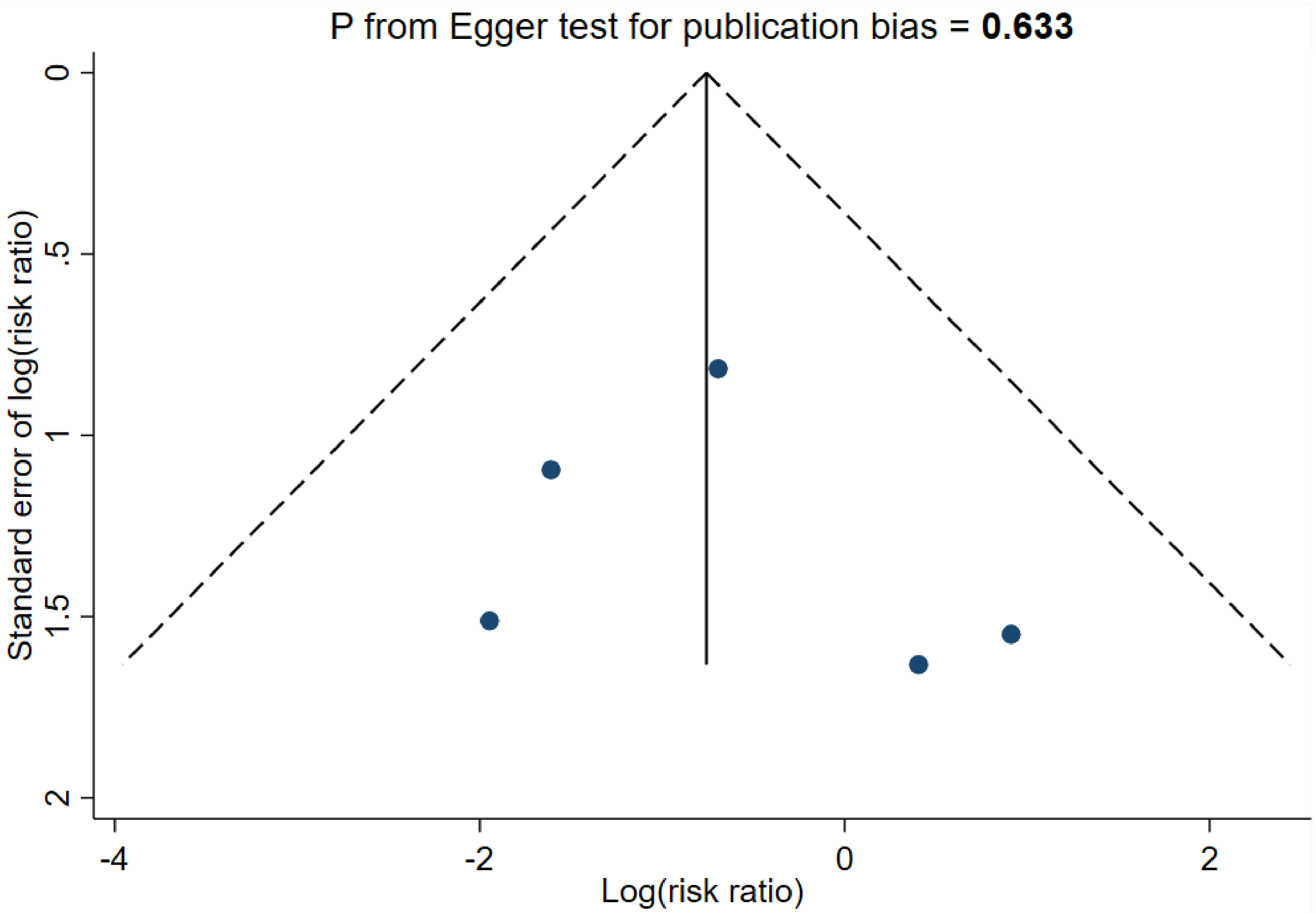

Figure S241 Funnel plot of Interstitial lung disease

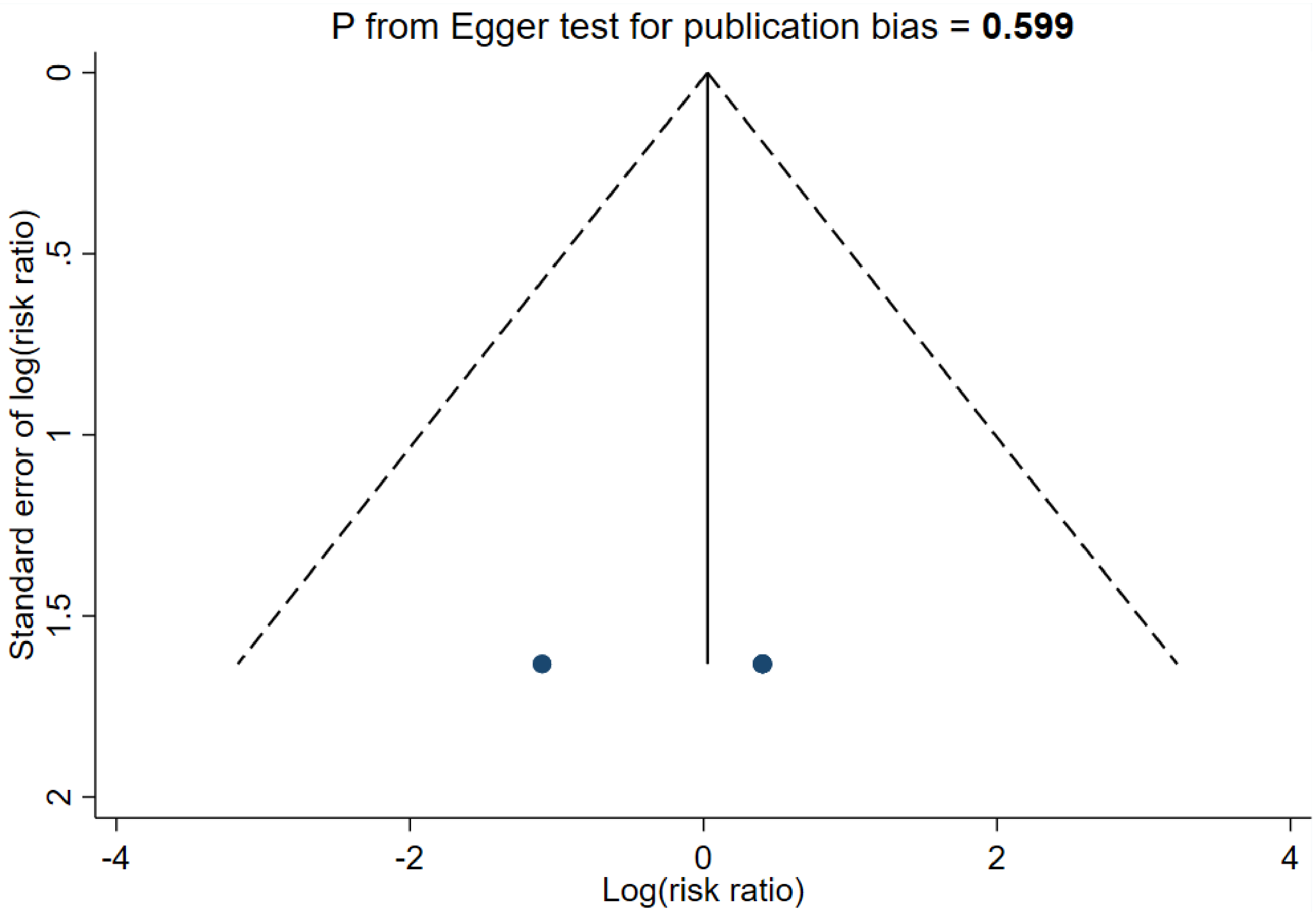

Figure S242 Funnel plot of Laryngeal oedema

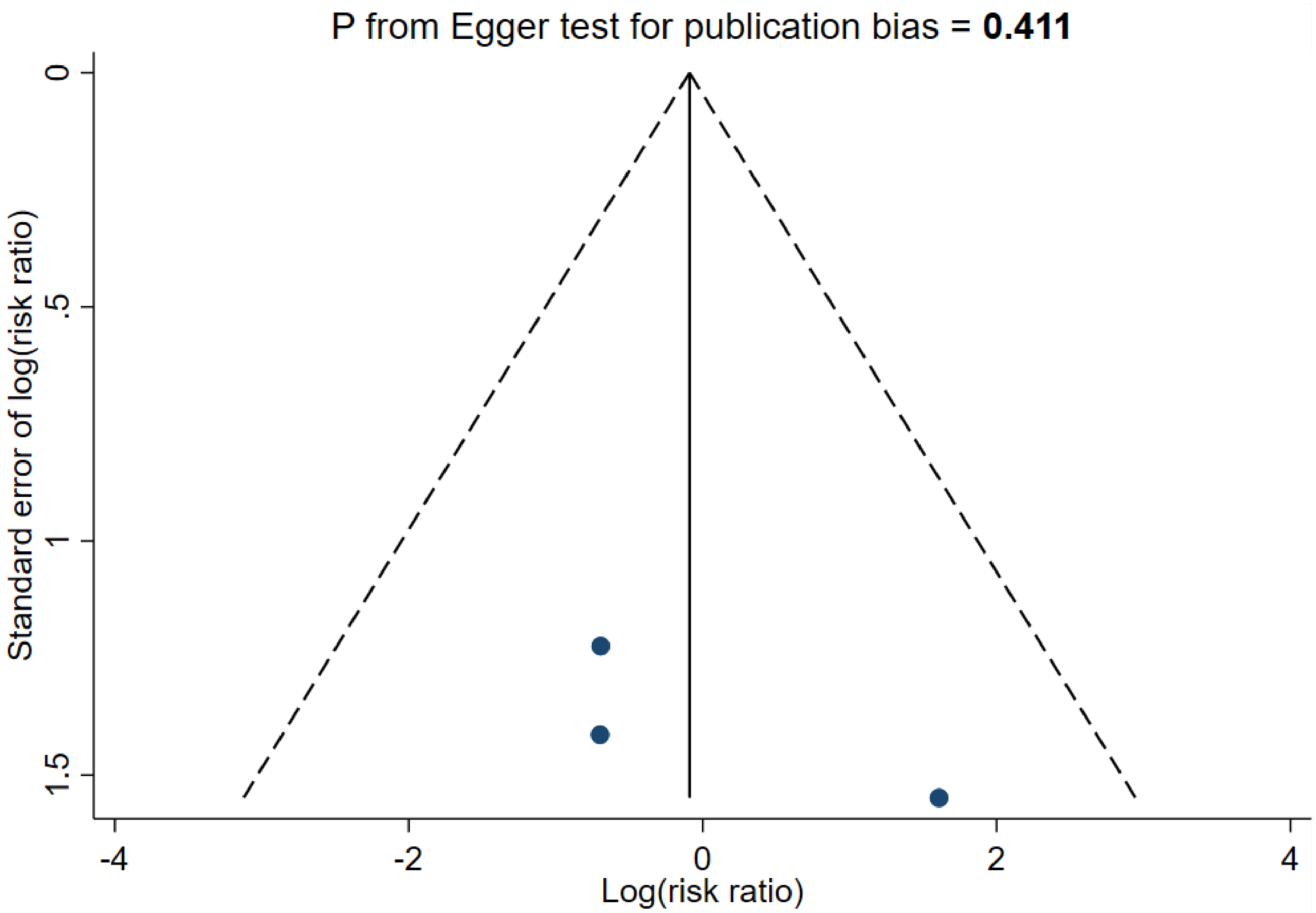

Figure S243 Funnel plot of Lung disorder

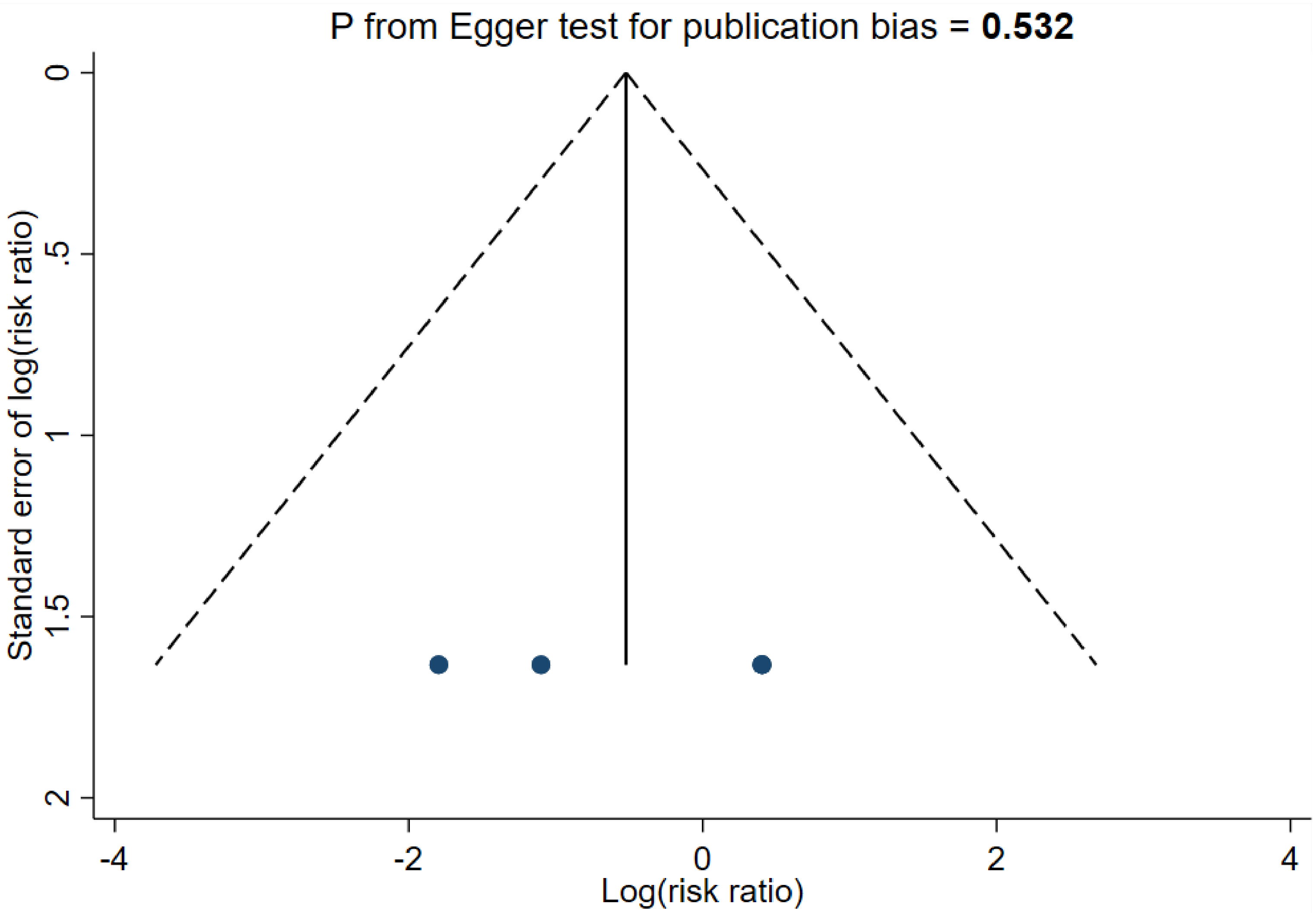

Figure S244 Funnel plot of Lung infiltration

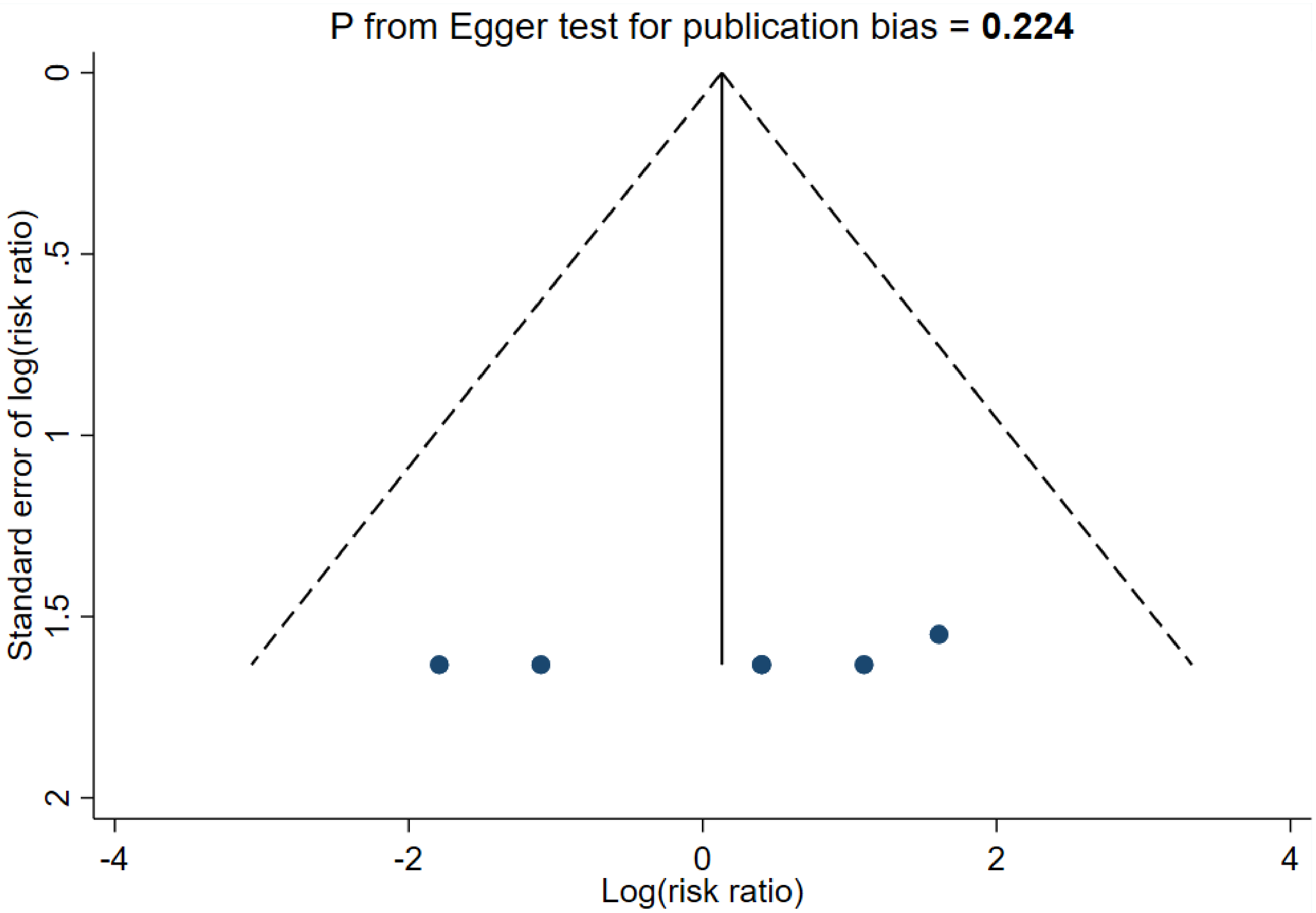

Figure S245 Funnel plot of Nasal polyps

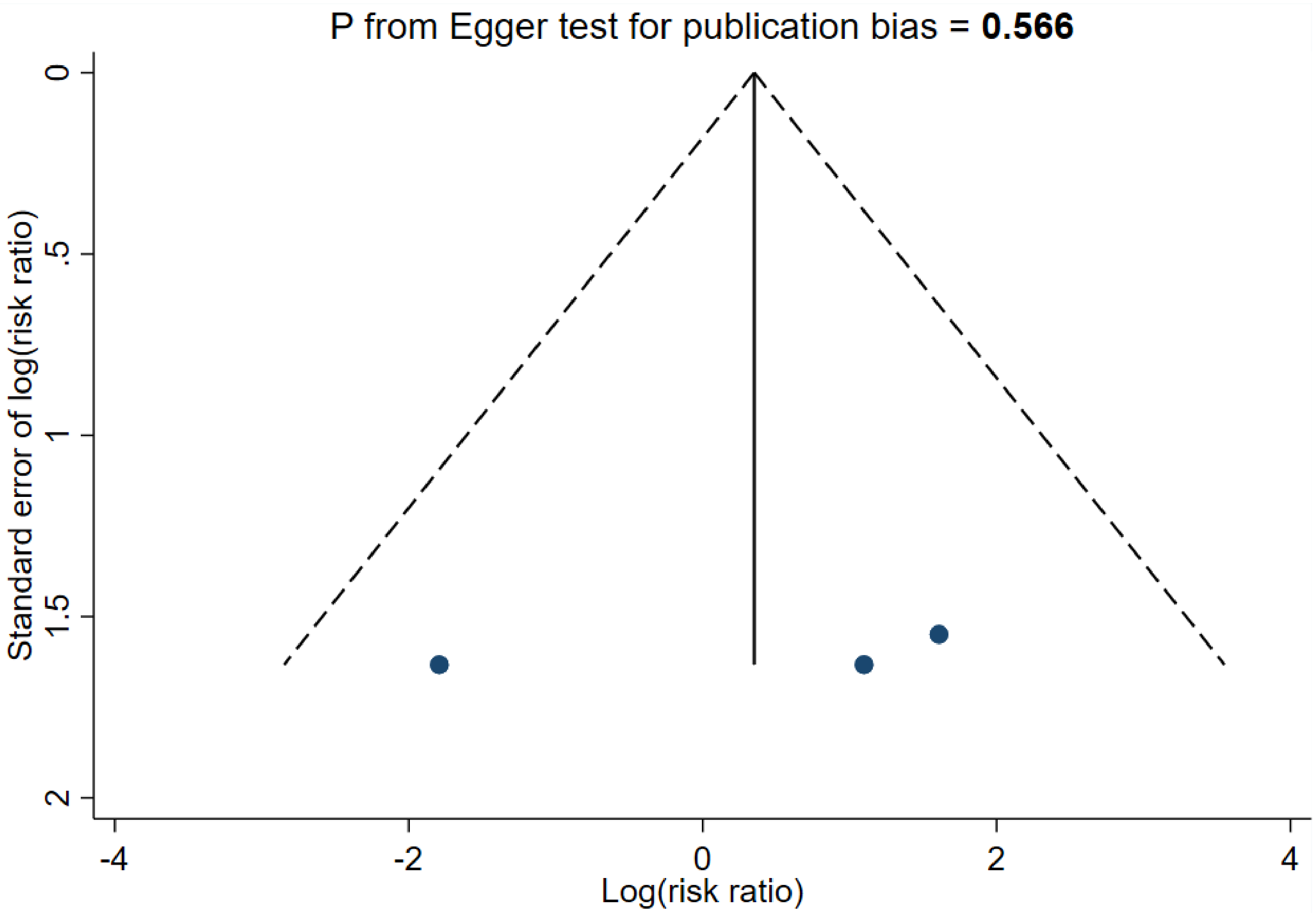

Figure S246 Funnel plot of Nasal septum deviation

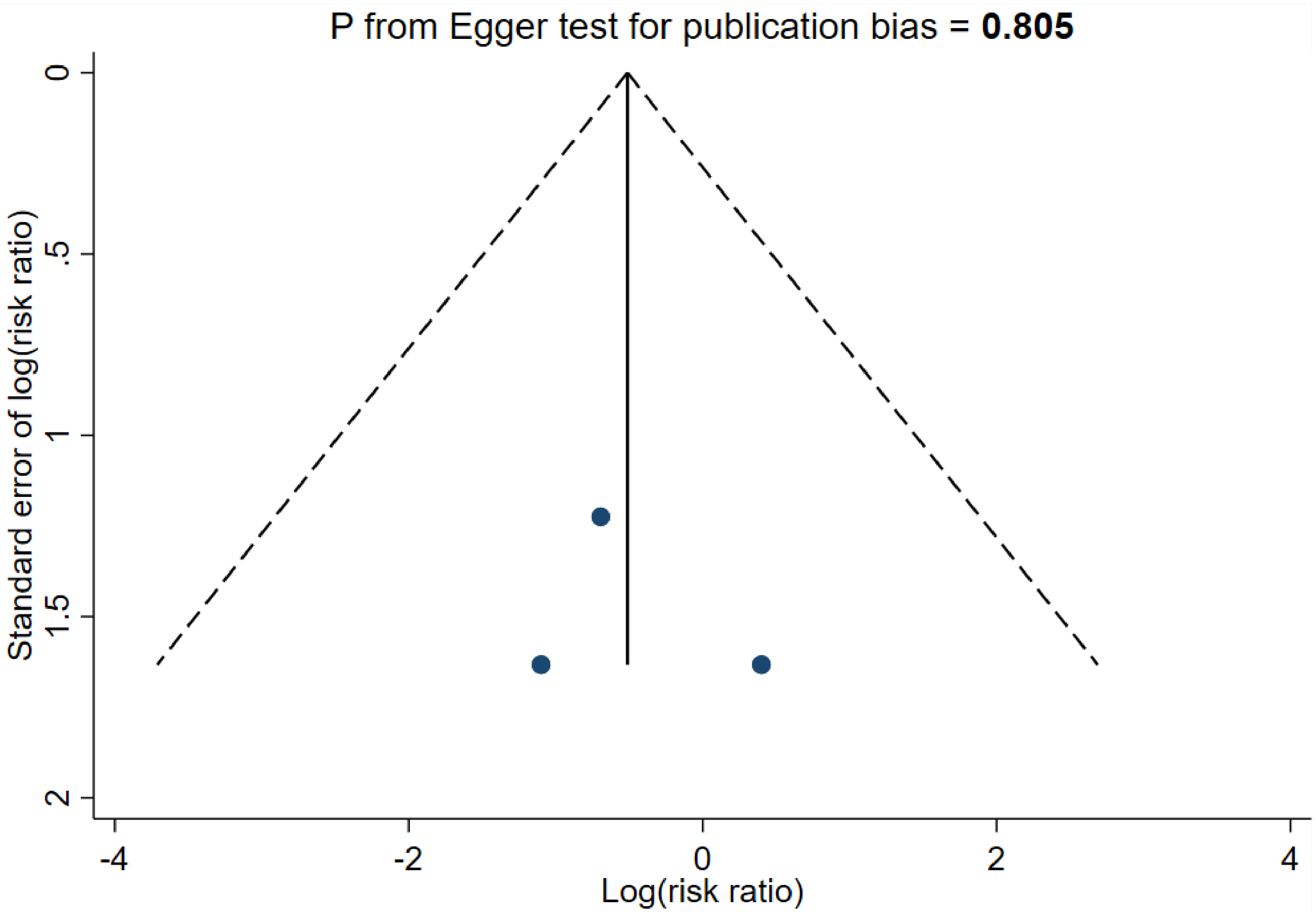

Figure S247 Funnel plot of Obstructive airways disorder

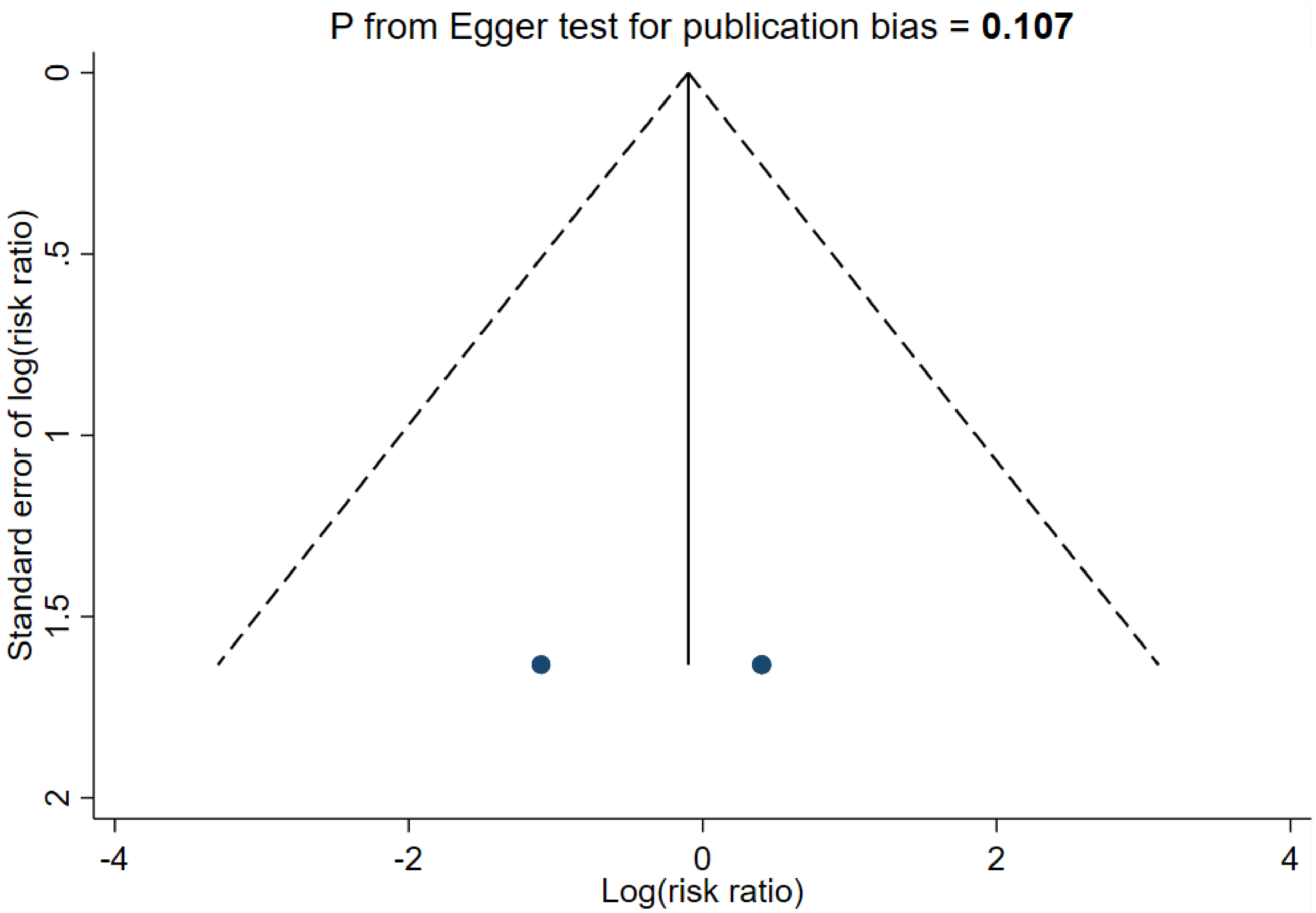

Figure S248 Funnel plot of Paranasal cyst

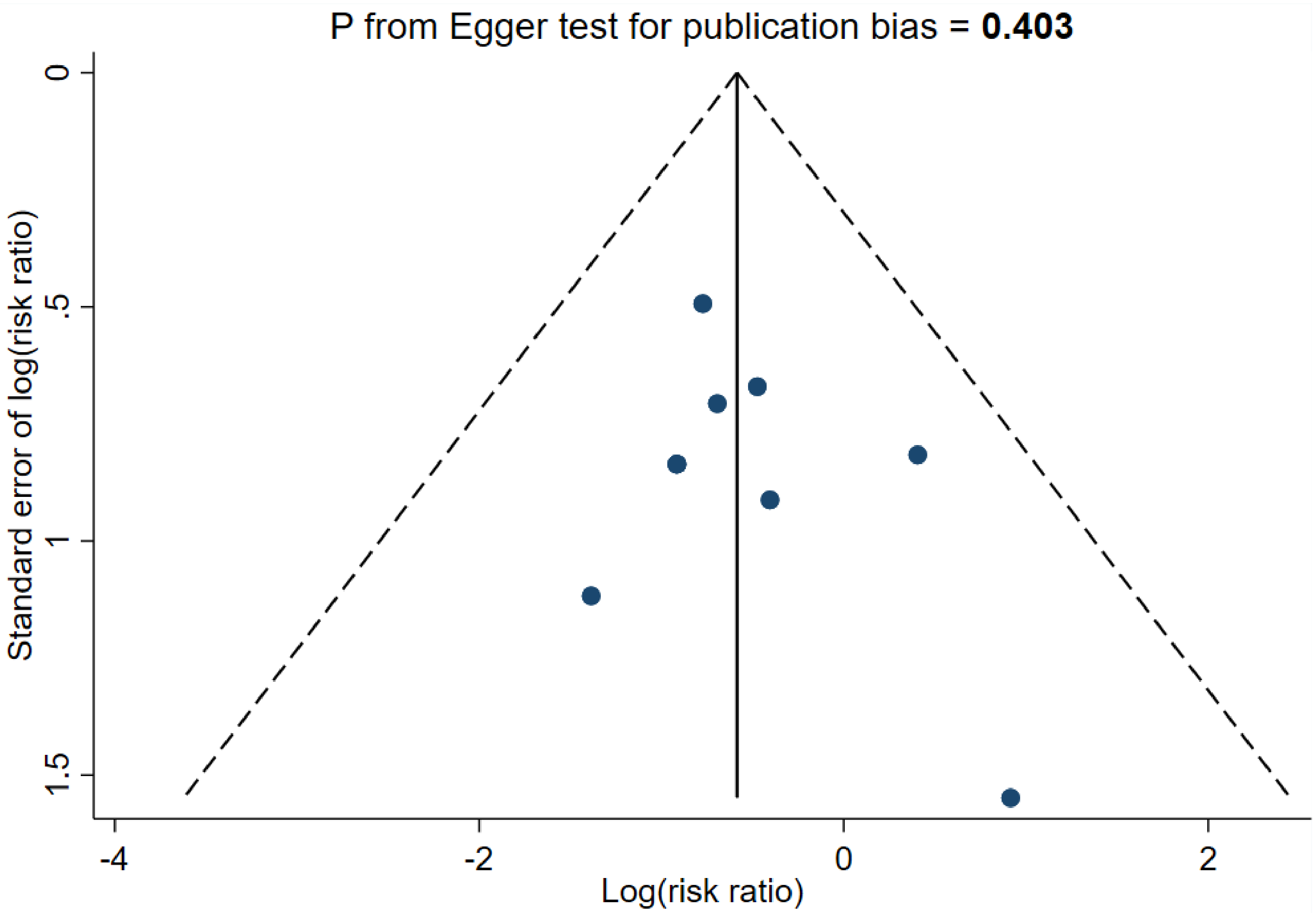

Figure S249 Funnel plot of Pleural effusion

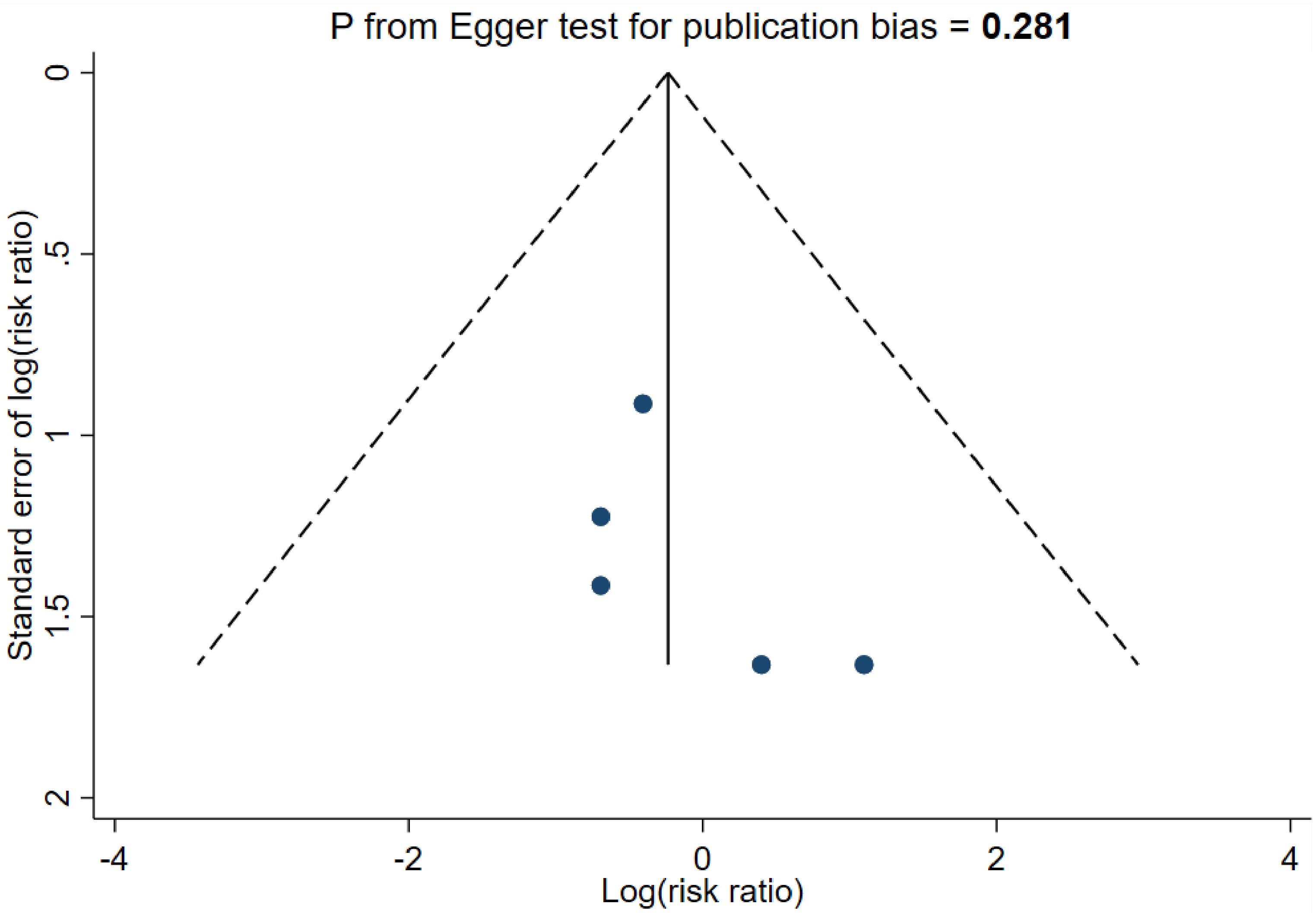

Figure S250 Funnel plot of Pleurisy

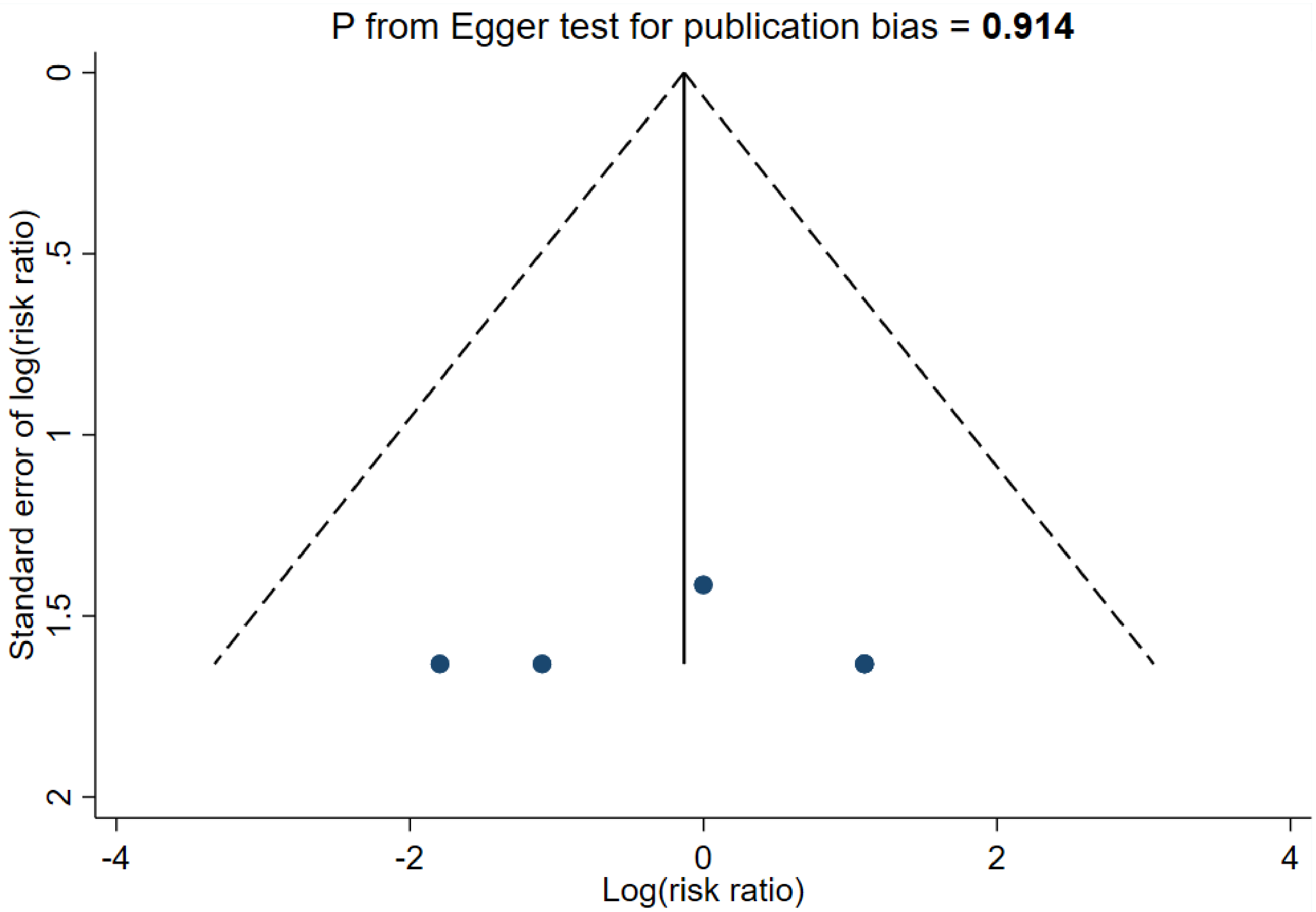

Figure S251 Funnel plot of Pleuritic pain

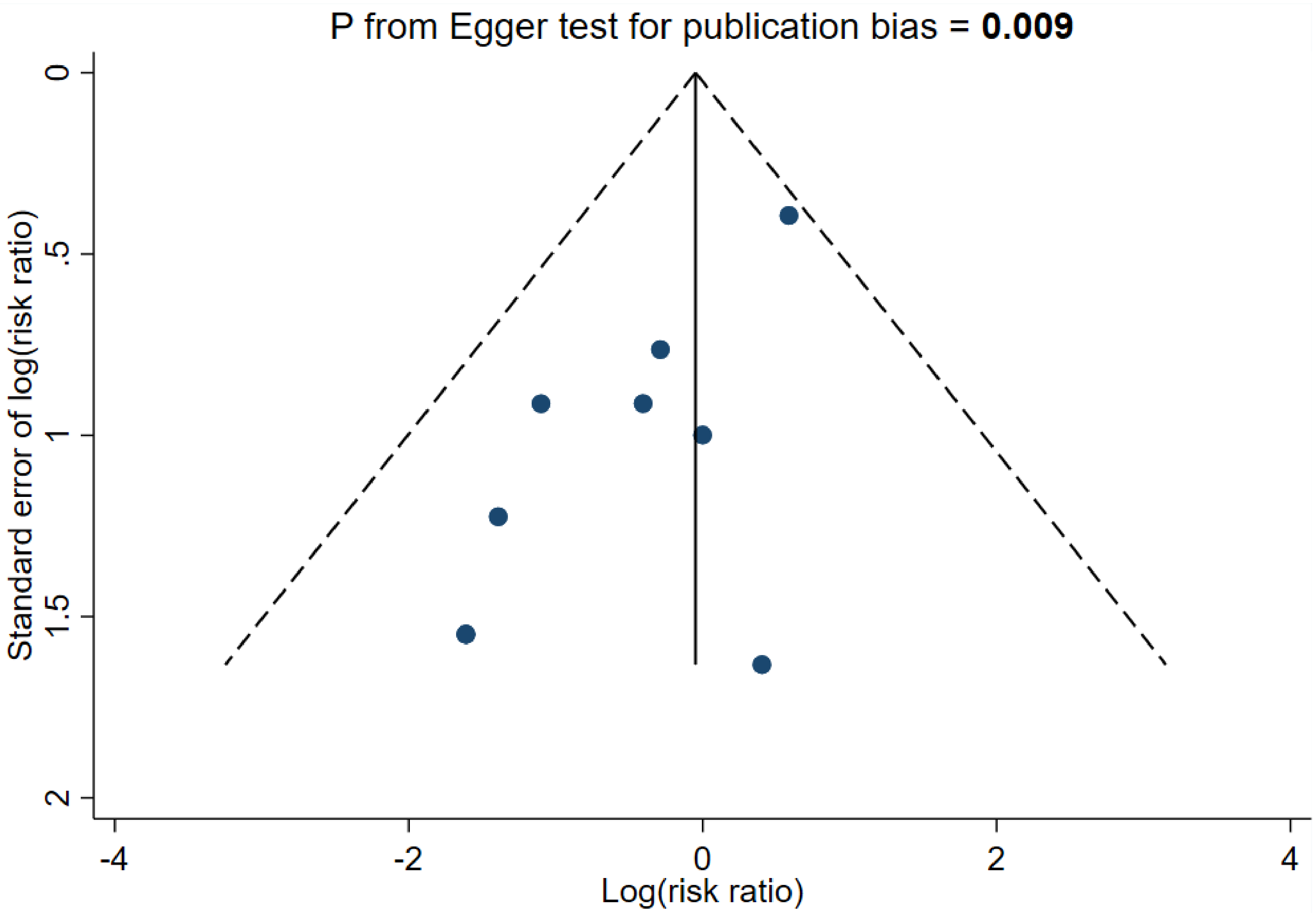

Figure S252 Funnel plot of Pneumonia aspiration

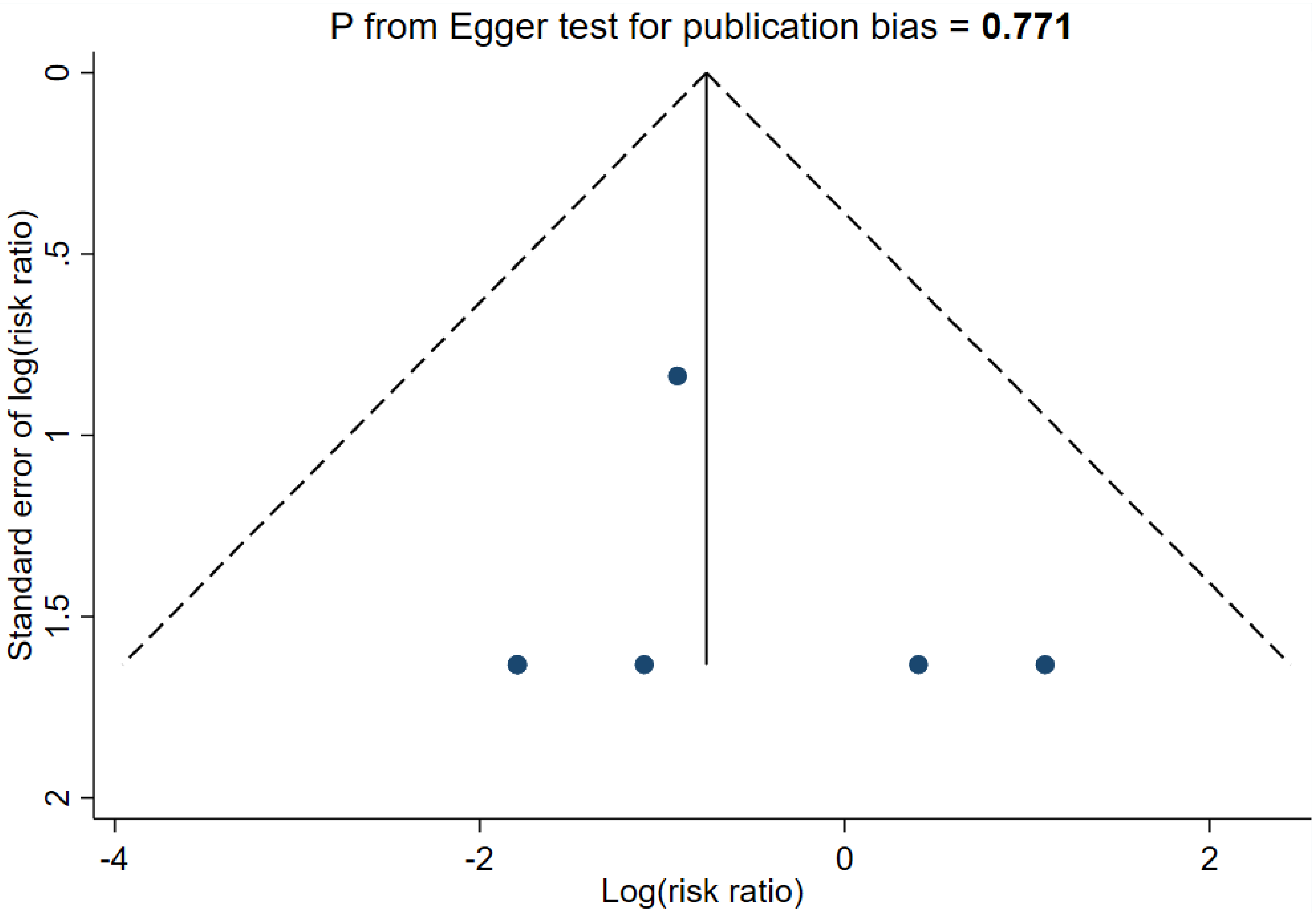

Figure S253 Funnel plot of Pneumonitis

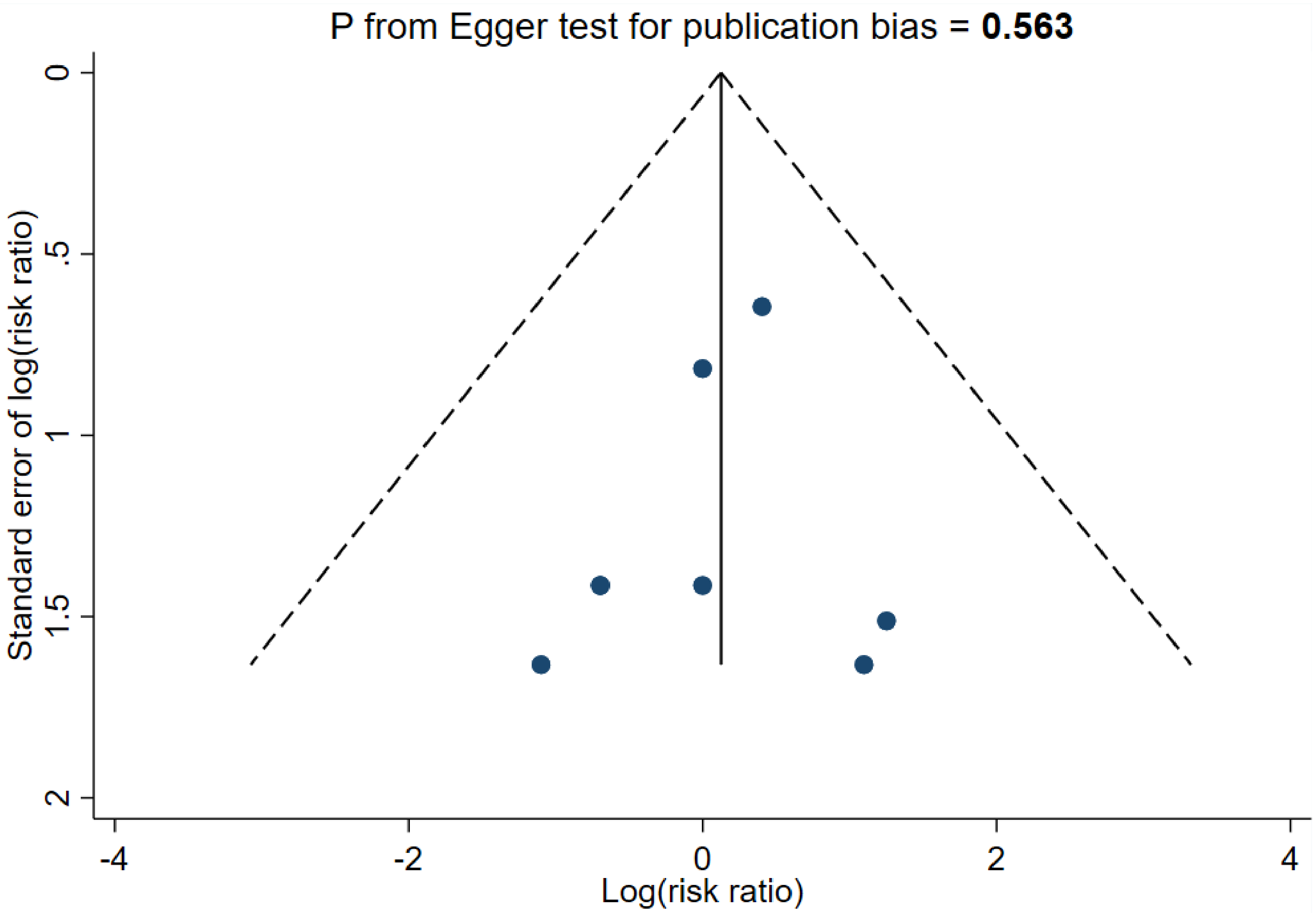

Figure S254 Funnel plot of Pneumothorax

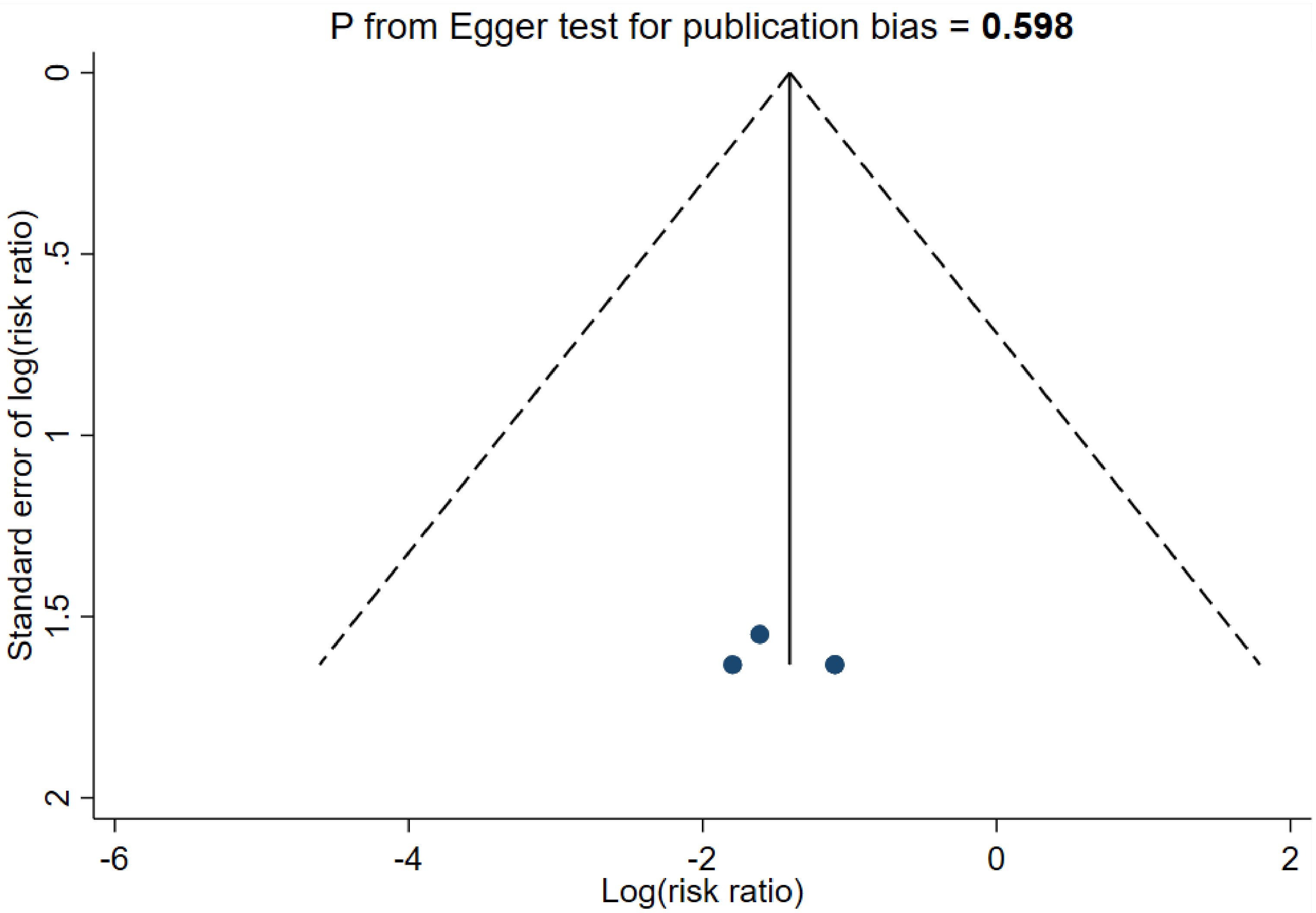

Figure S255 Funnel plot of Pulmonary arterial hypertension

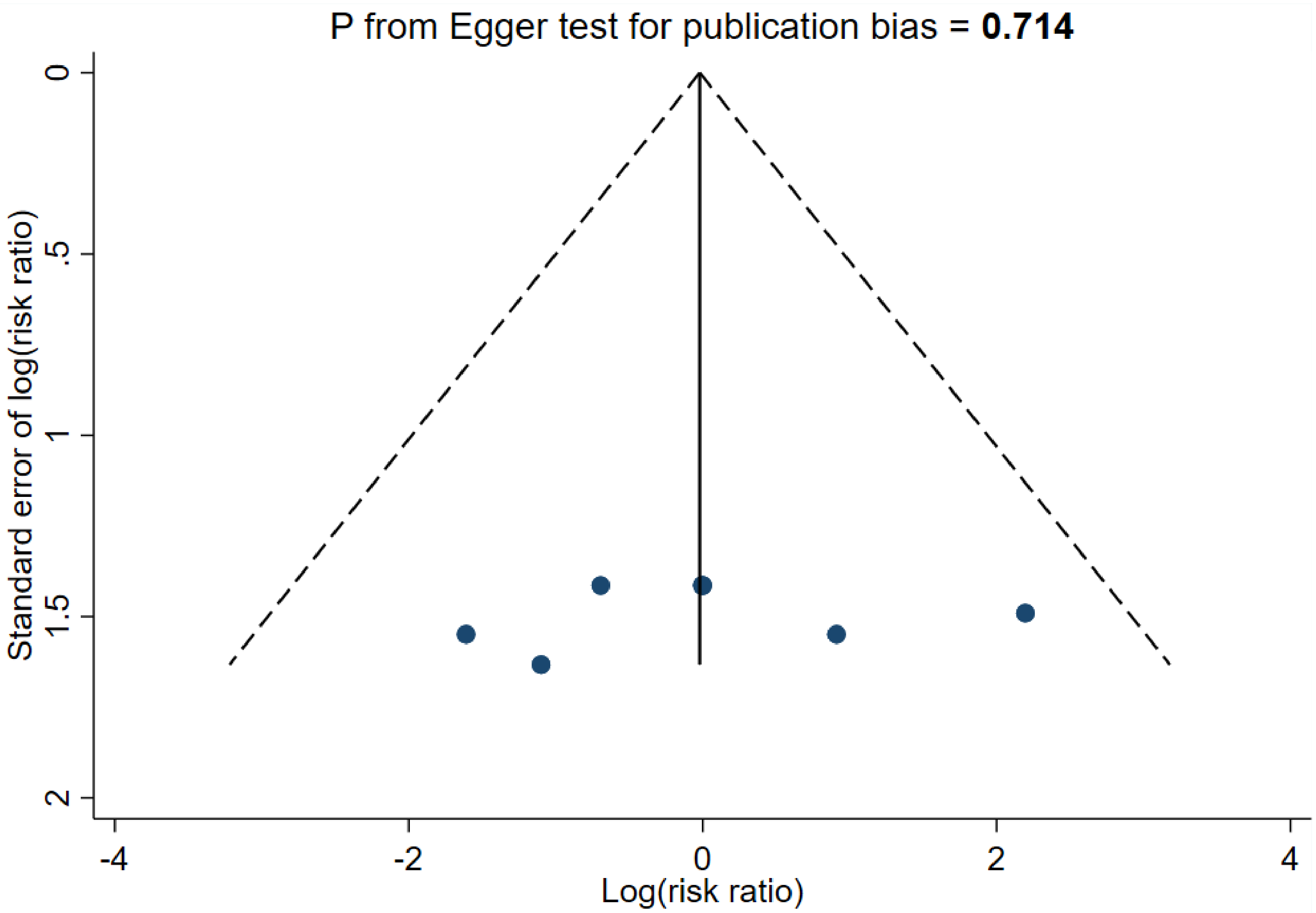

Figure S256 Funnel plot of Pulmonary congestion

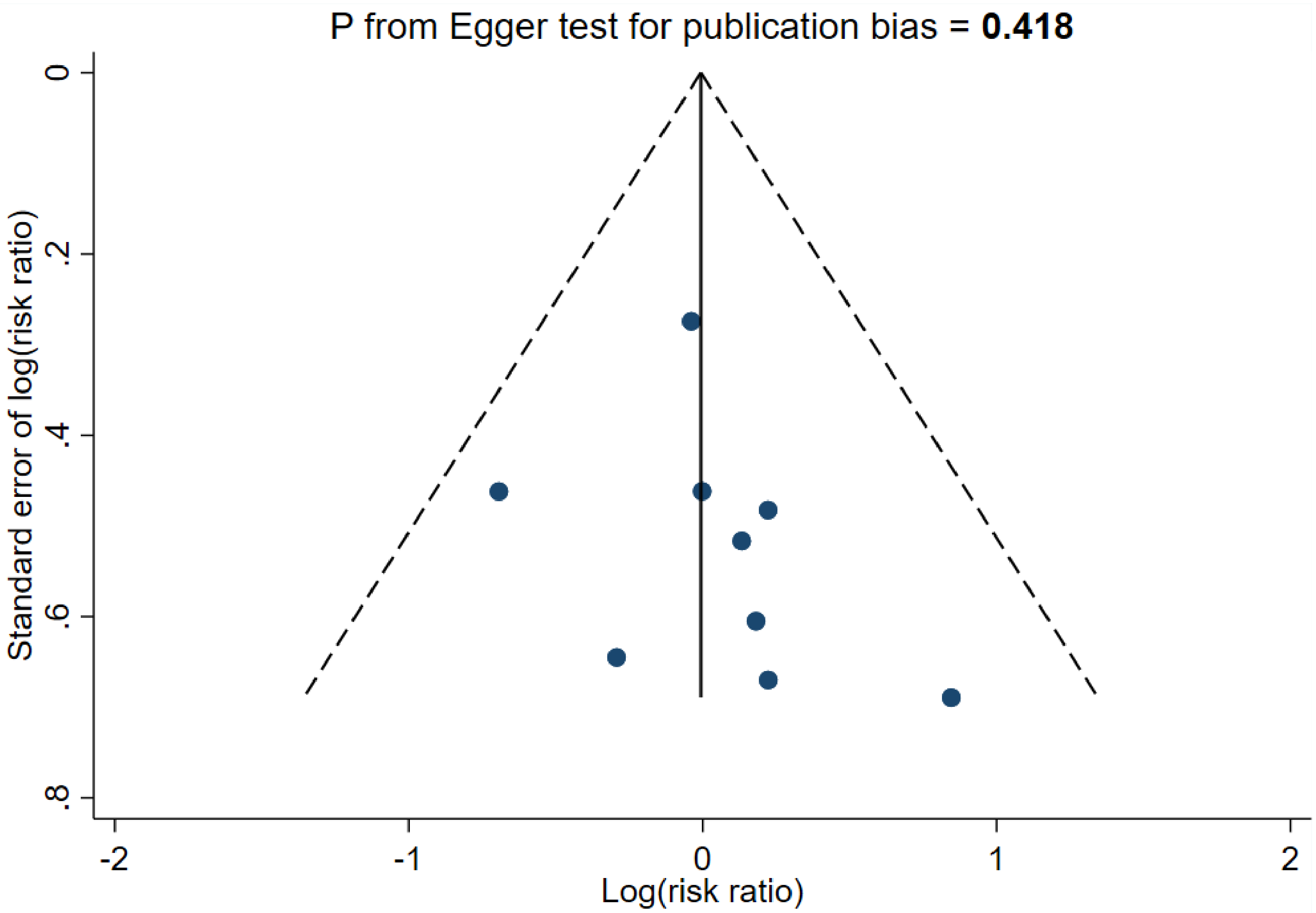

Figure S257 Funnel plot of Pulmonary embolism

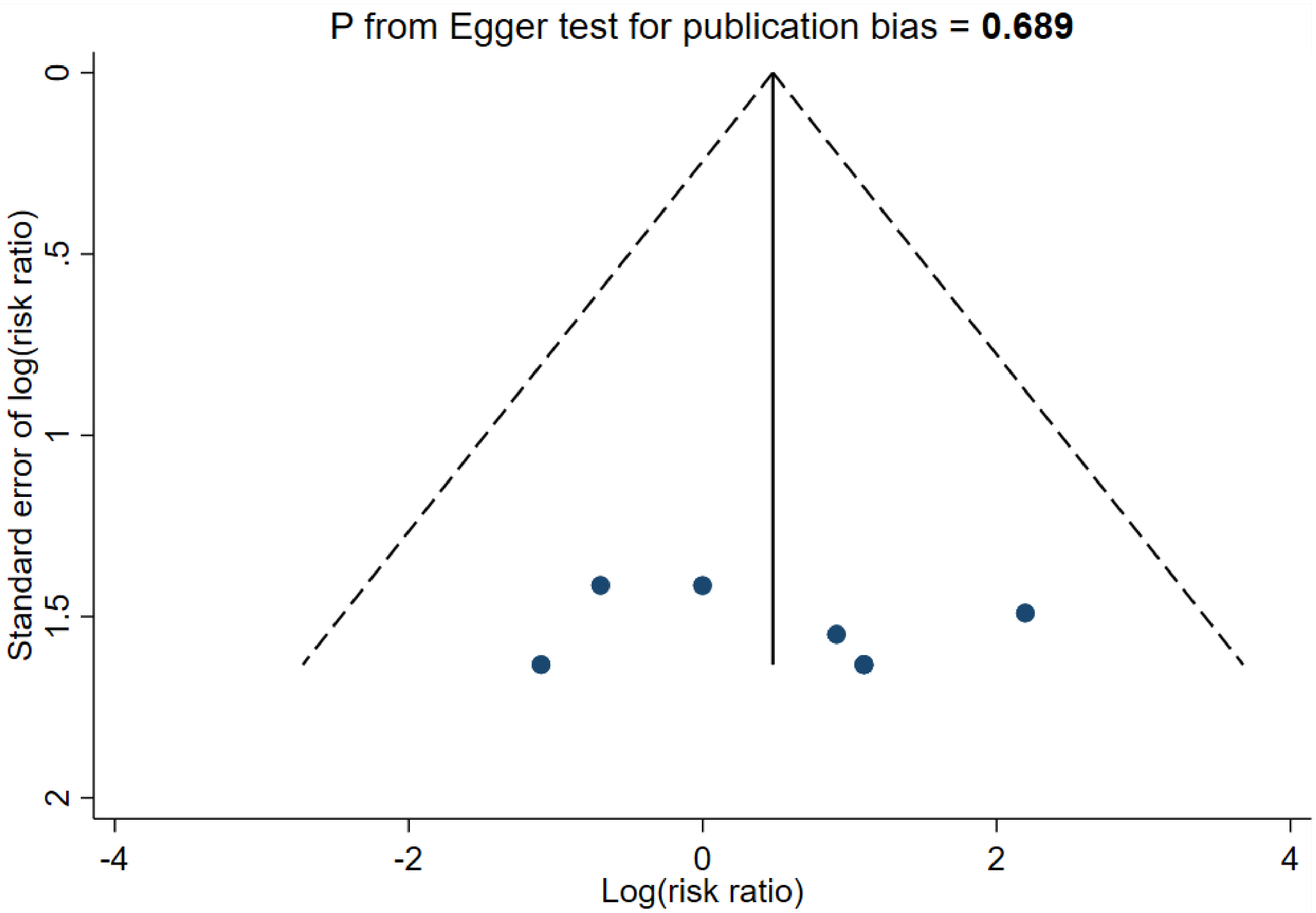

Figure S258 Funnel plot of Pulmonary fibrosis

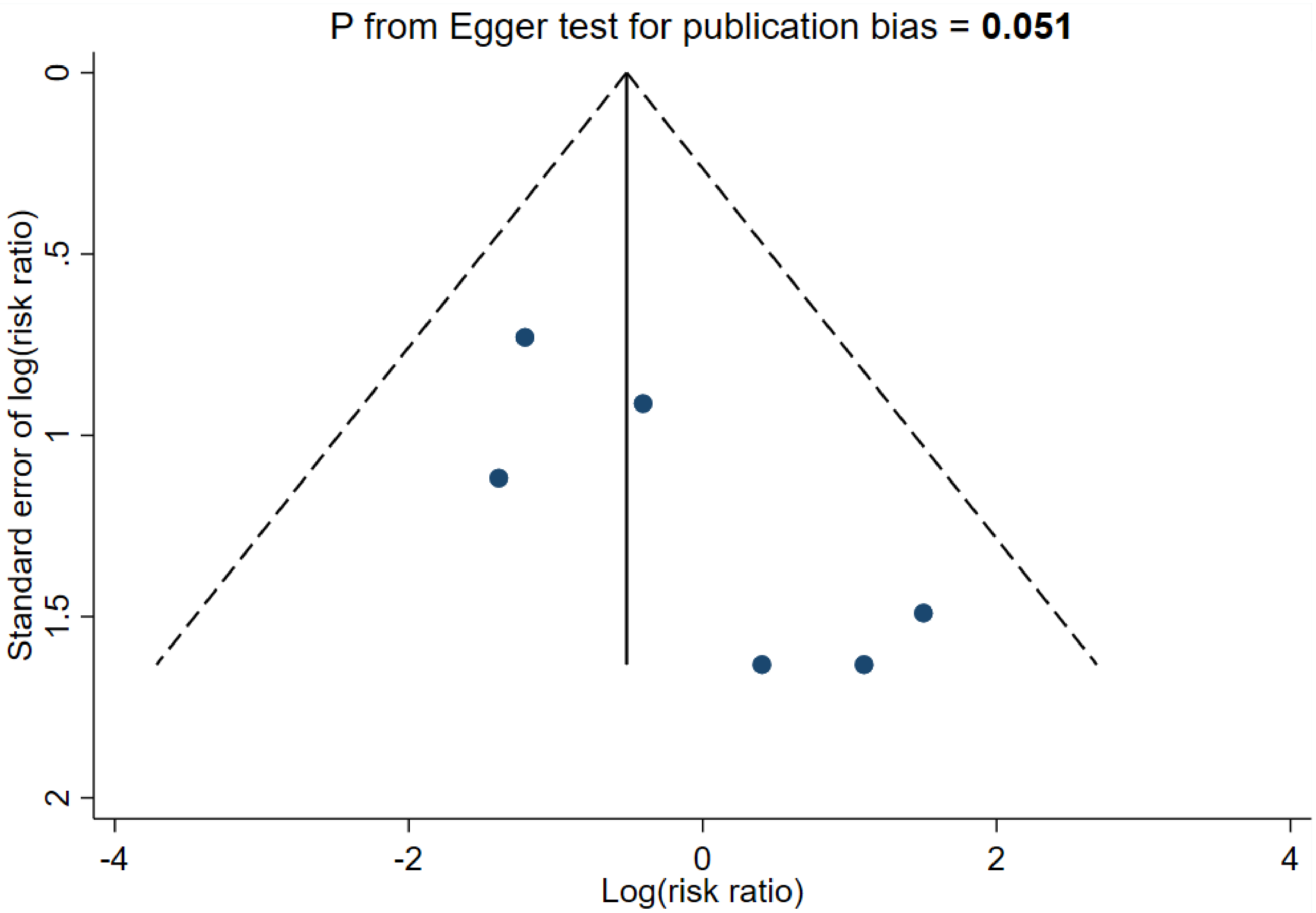

Figure S259 Funnel plot of Pulmonary hypertension

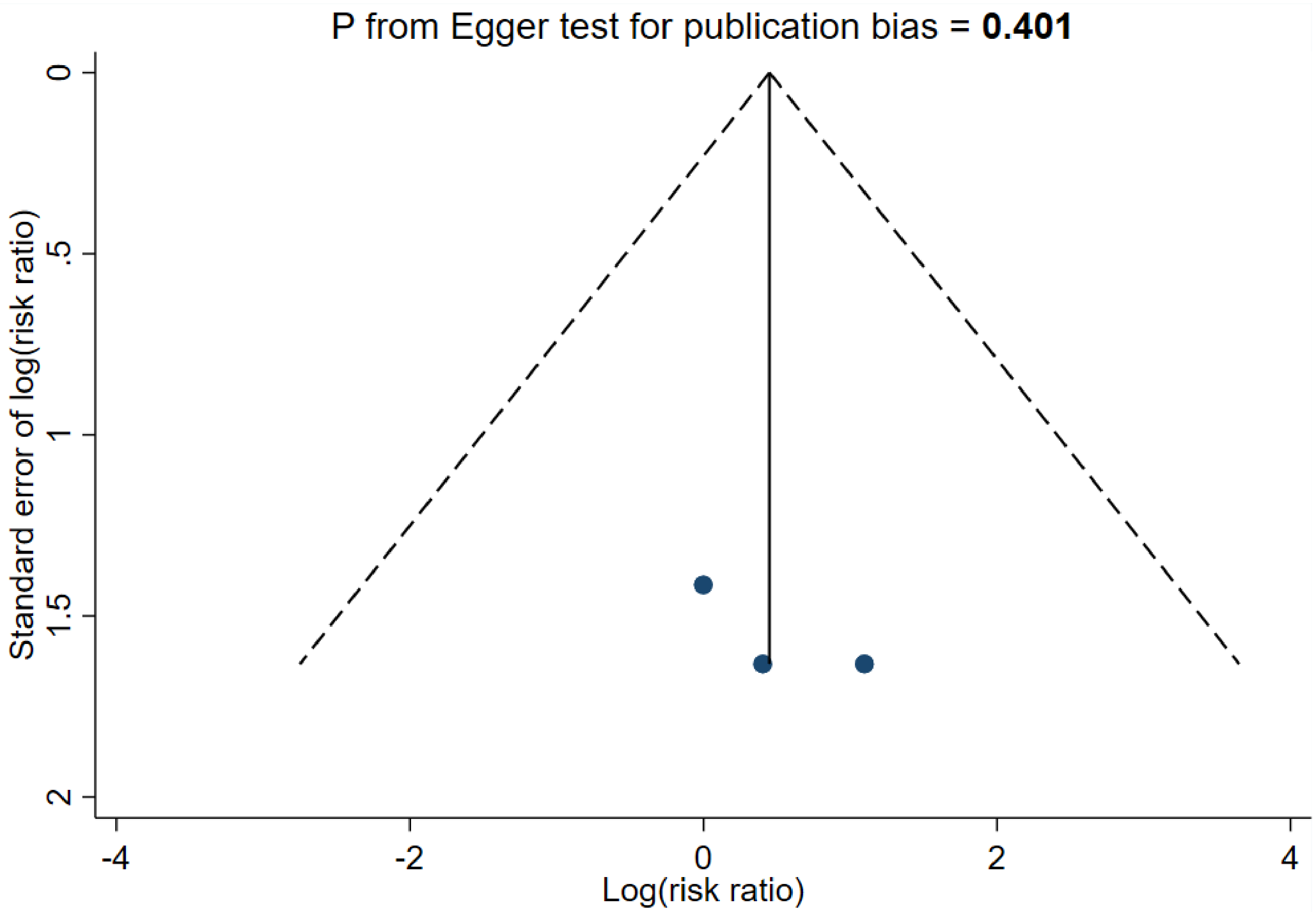

Figure S260 Funnel plot of Pulmonary infarction

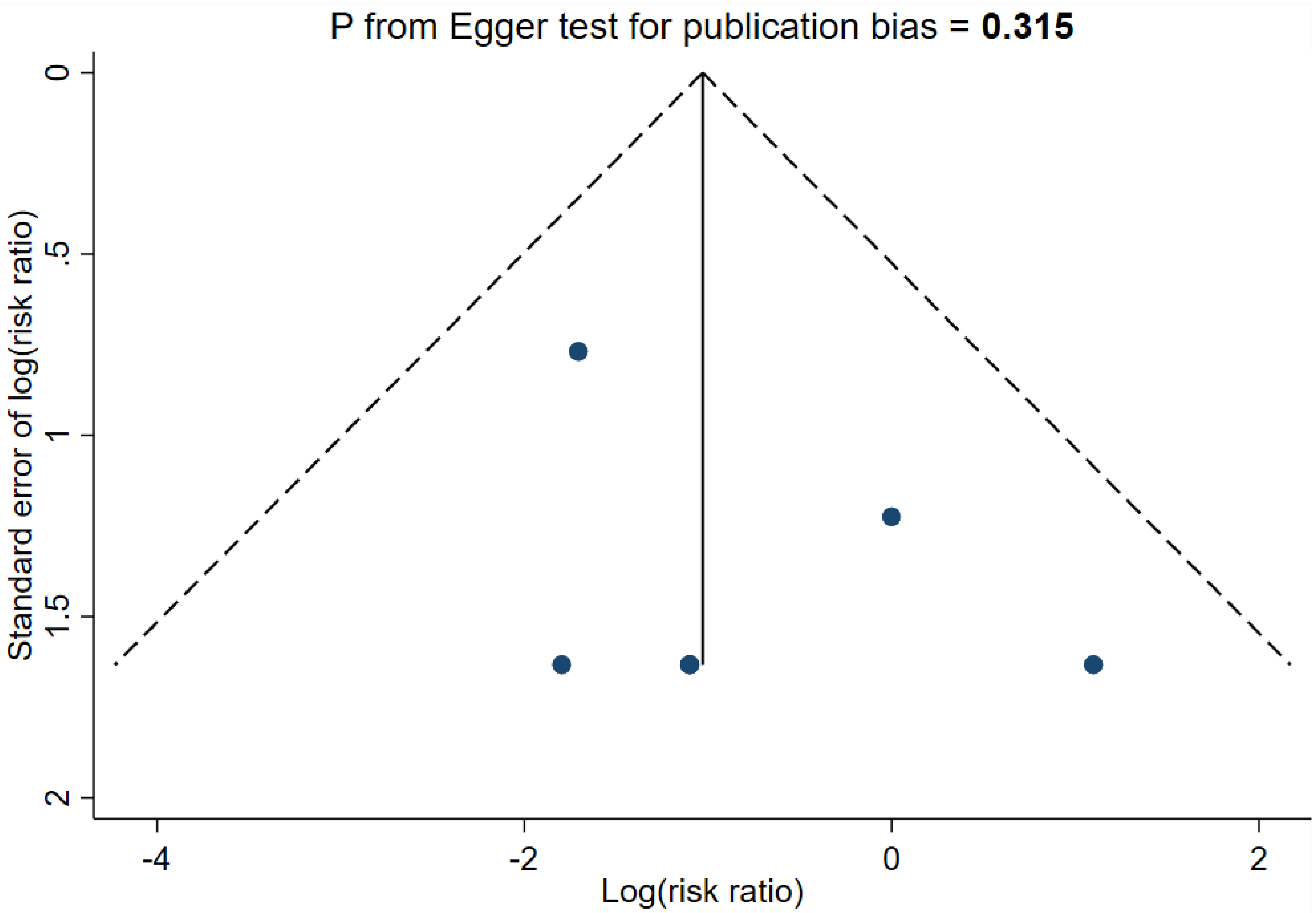

Figure S261 Funnel plot of Pulmonary mass

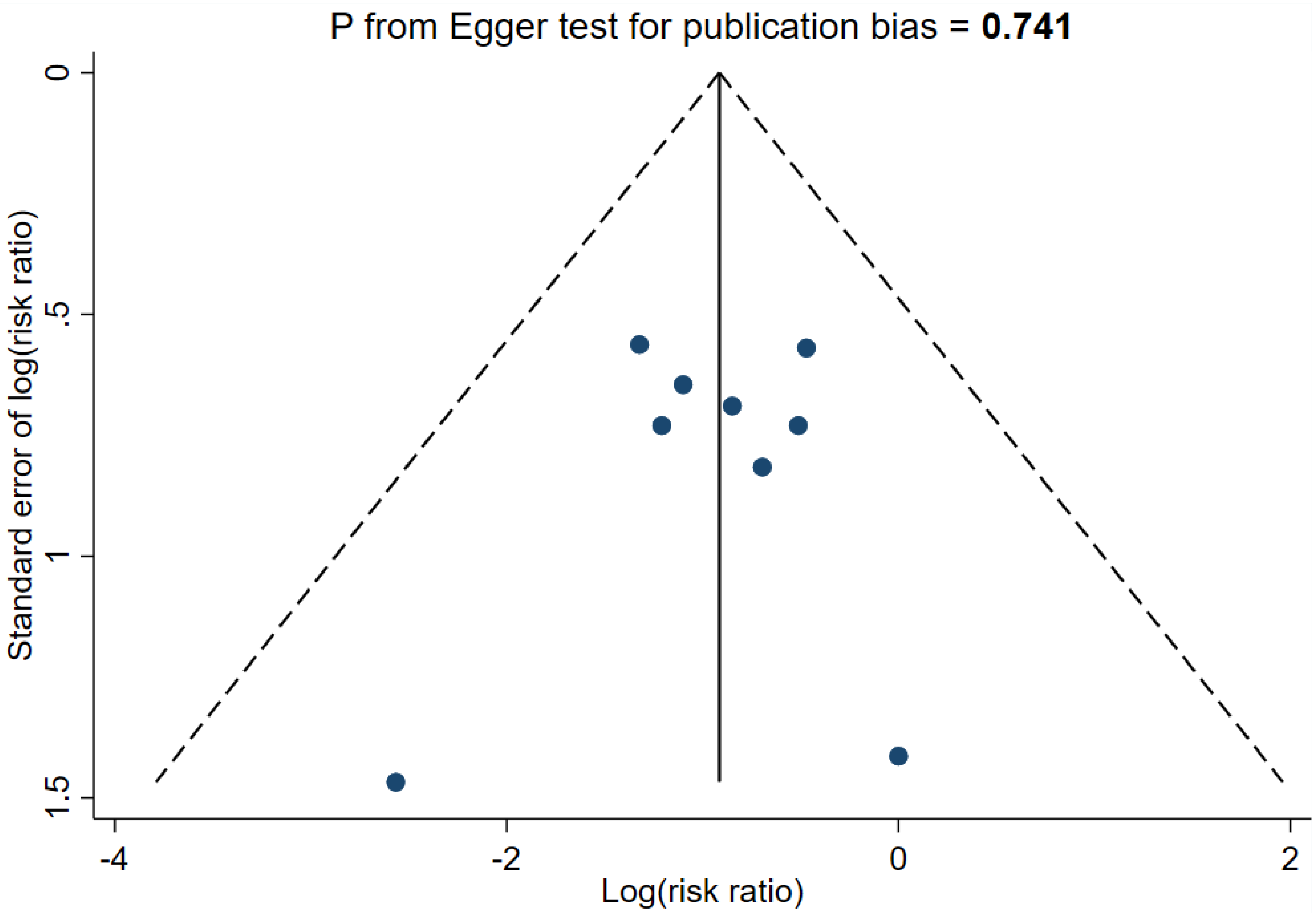

Figure S262 Funnel plot of Pulmonary oedema

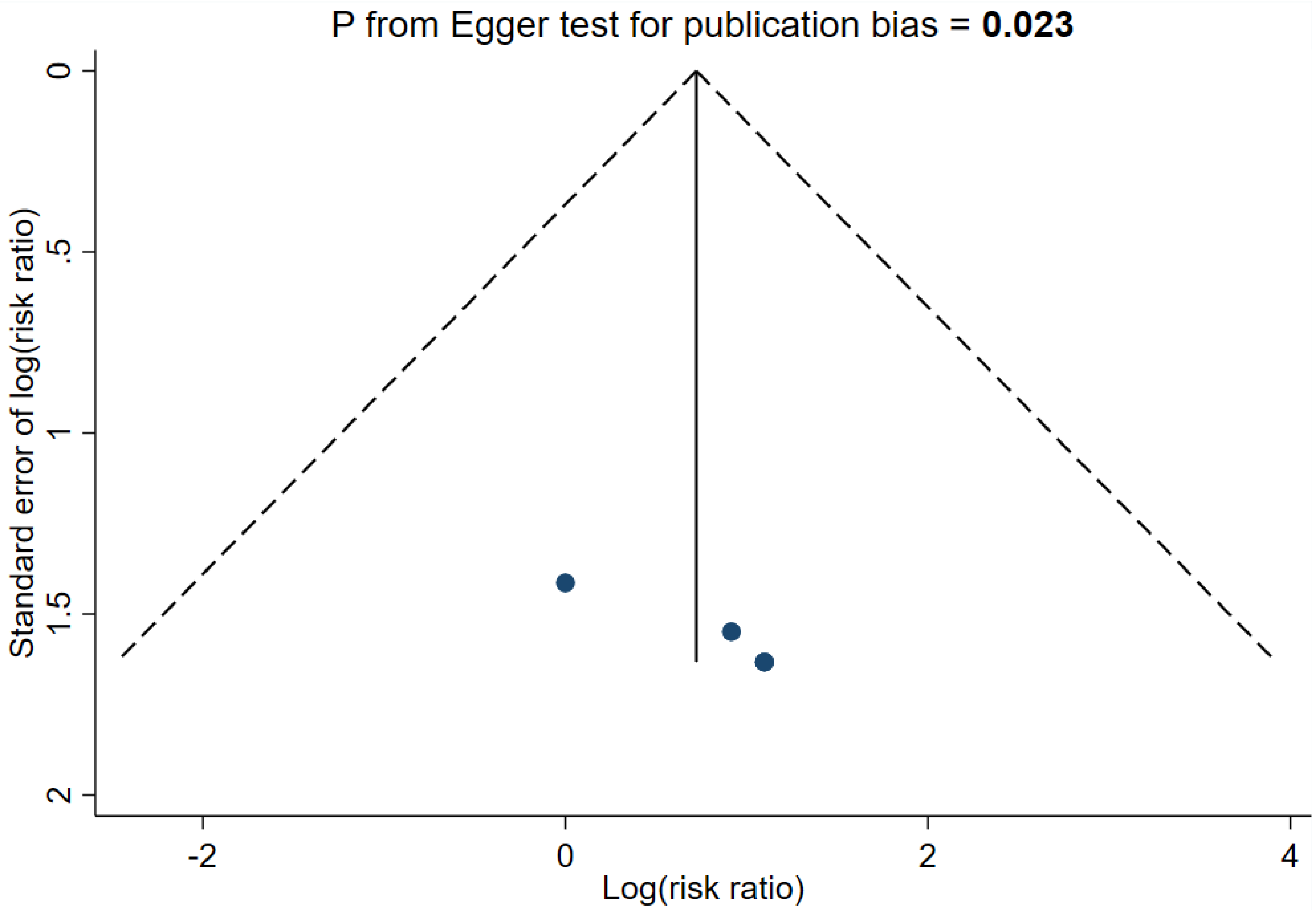

Figure S263 Funnel plot of Respiratory acidosis

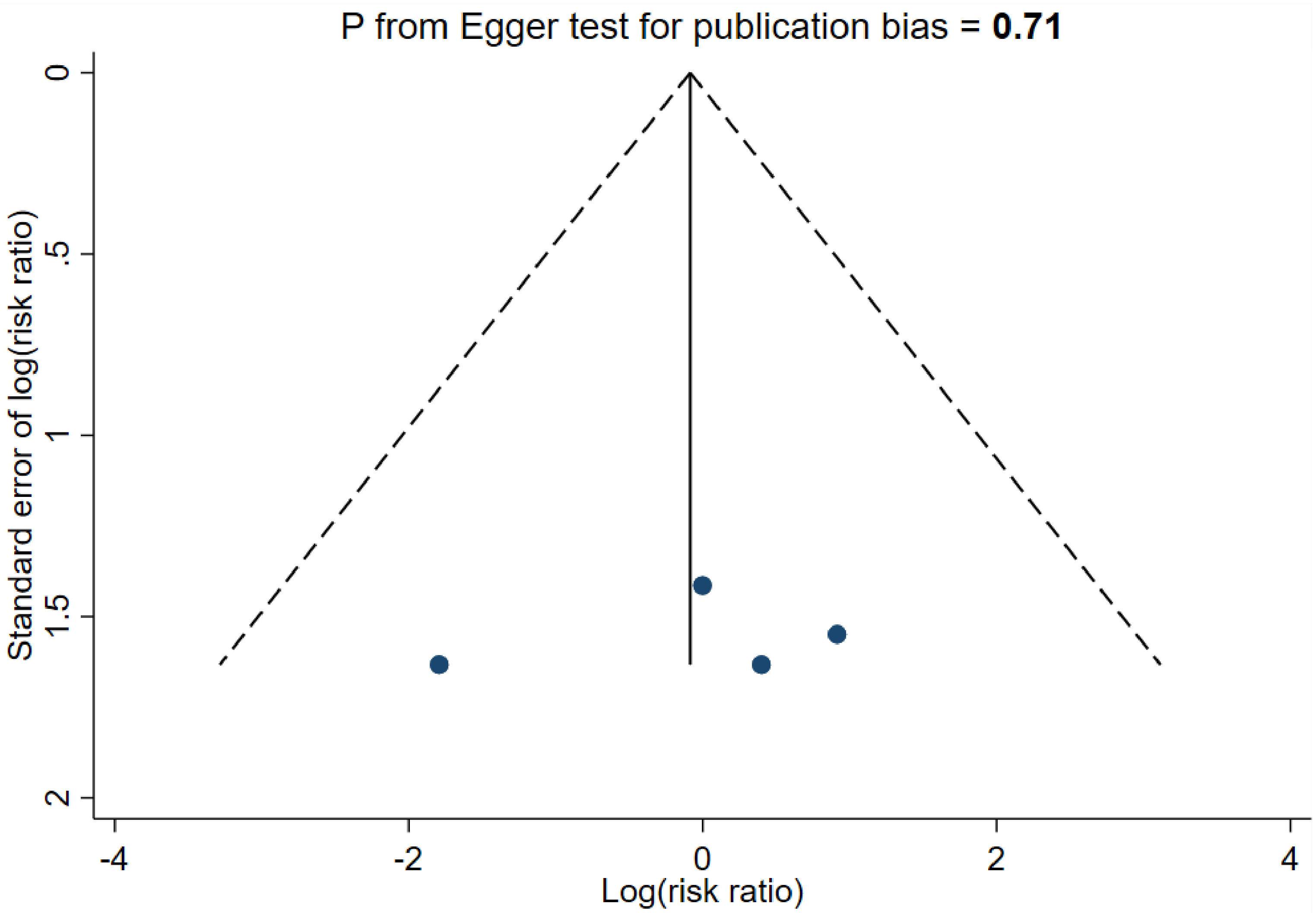

Figure S264 Funnel plot of Respiratory arrest

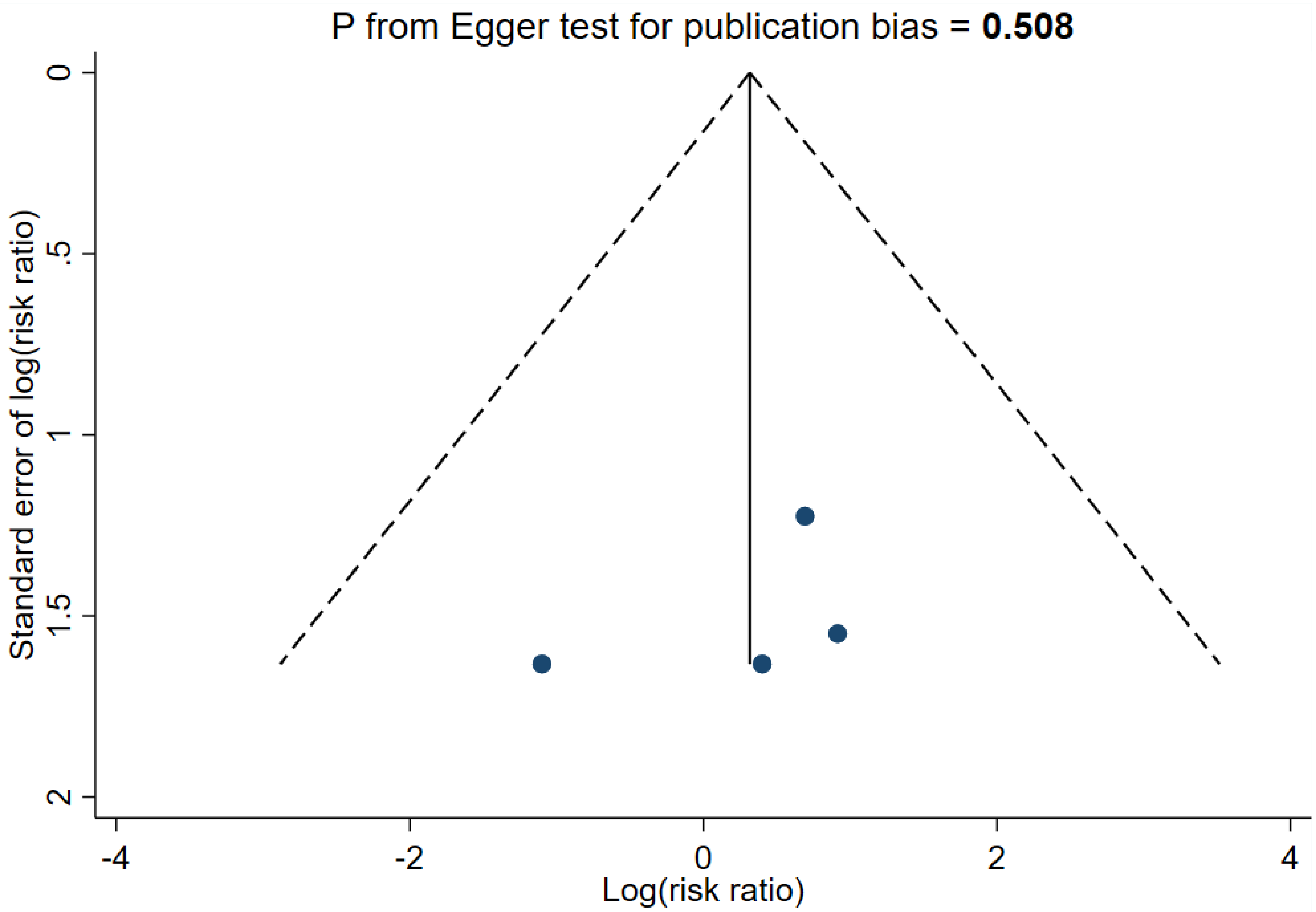

Figure S265 Funnel plot of Respiratory distress

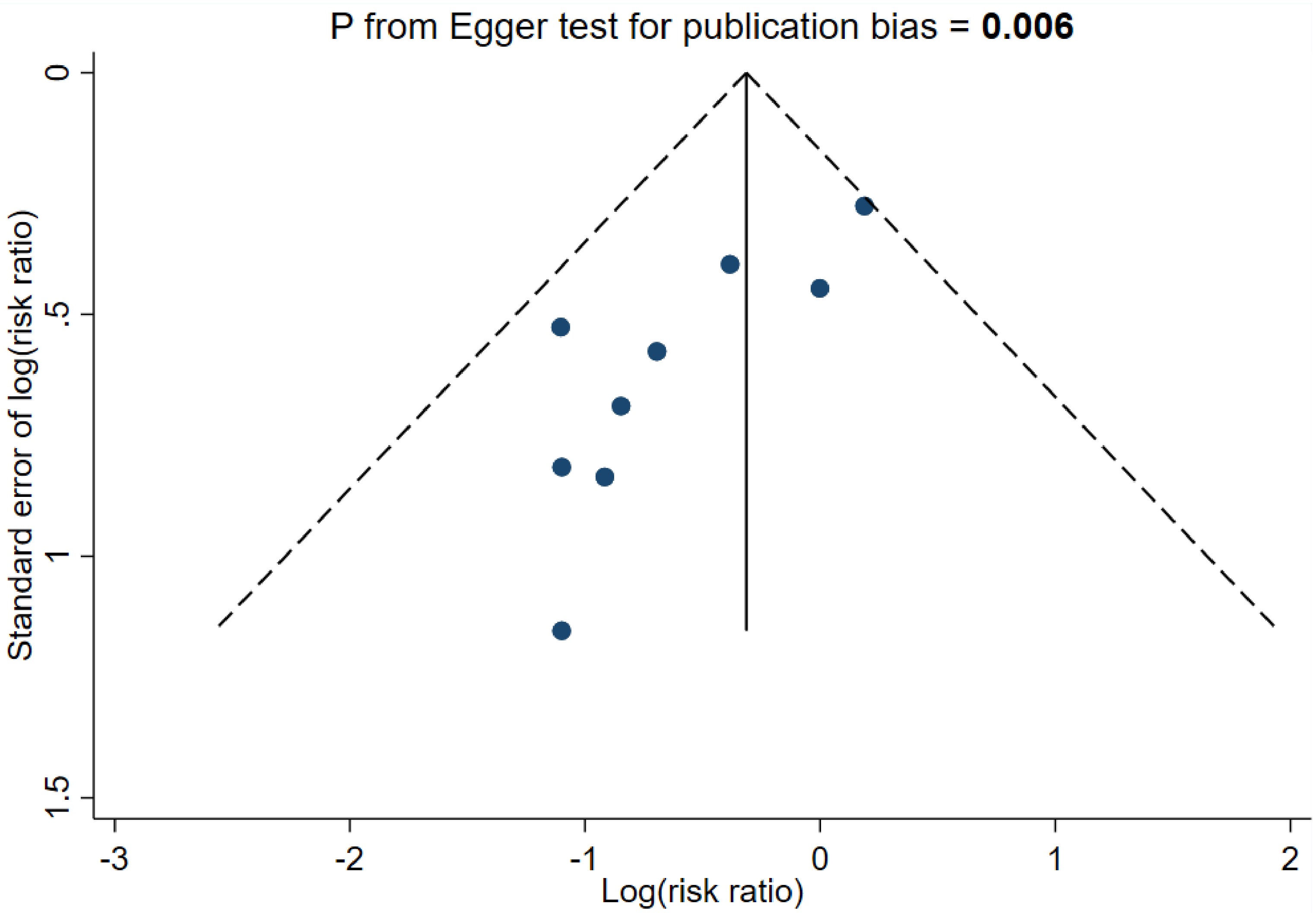

Figure S266 Funnel plot of Respiratory failure

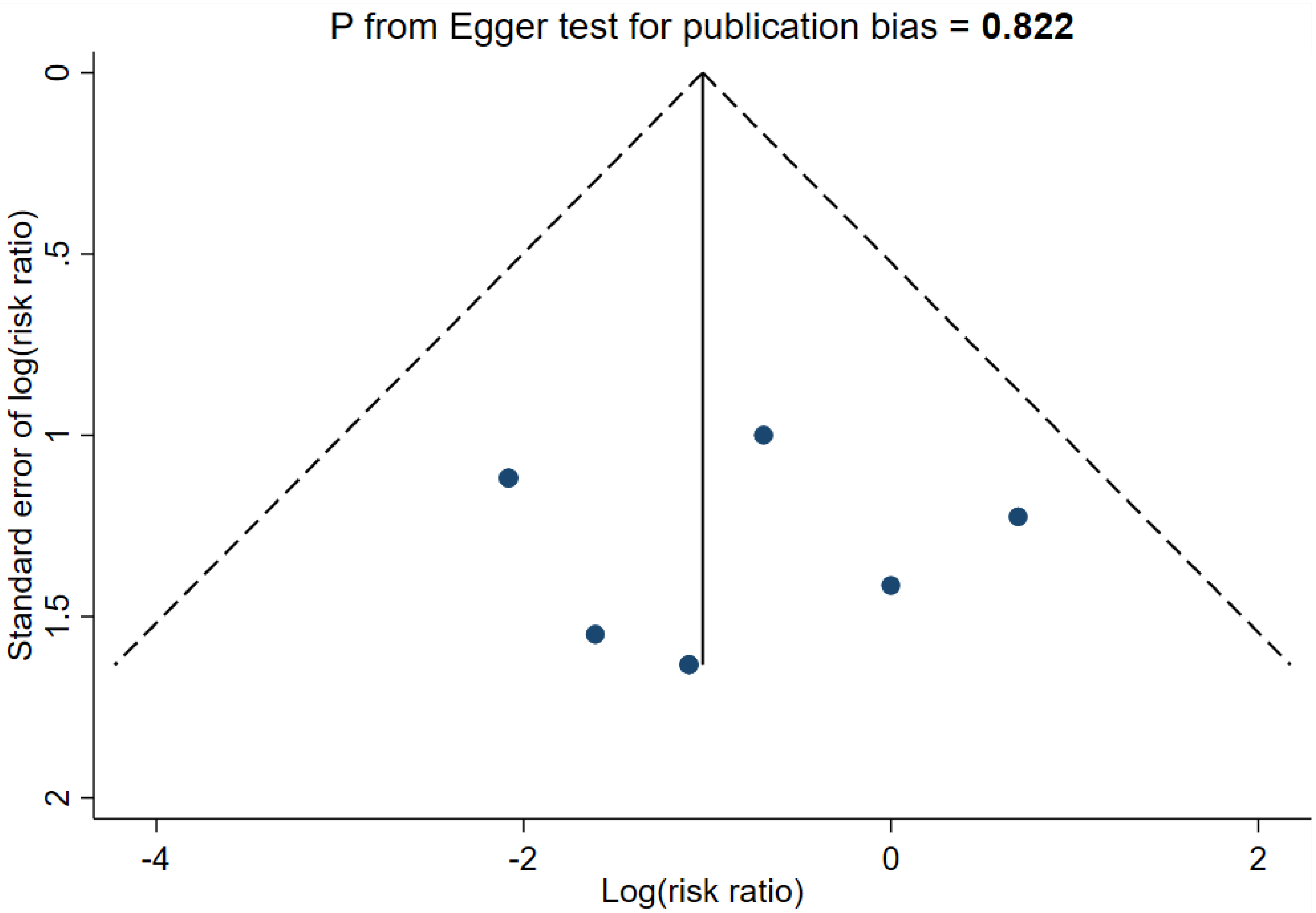

Figure S267 Funnel plot of Sleep apnoea syndrome

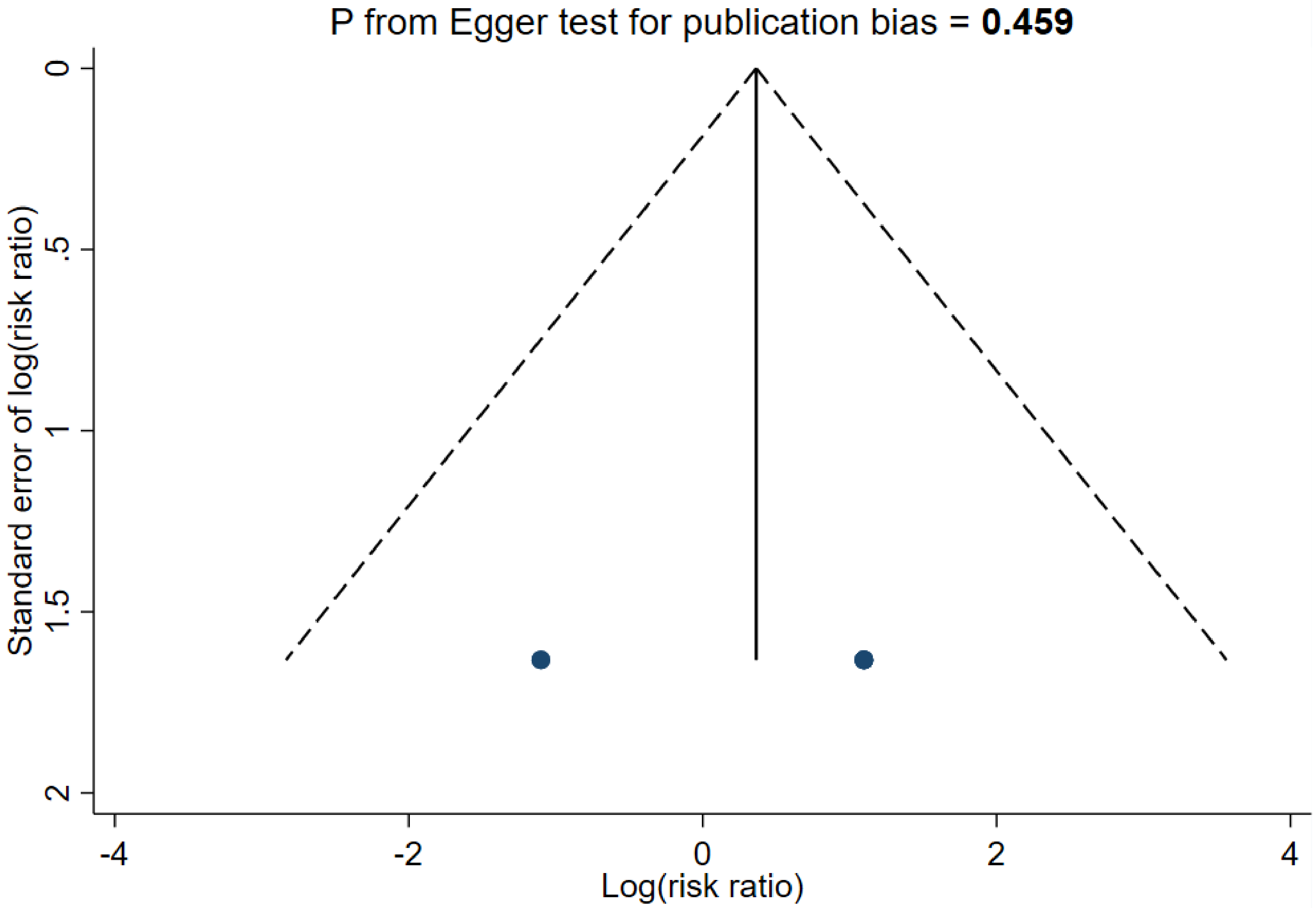

Figure S268 Funnel plot of Vocal cord leukoplakia

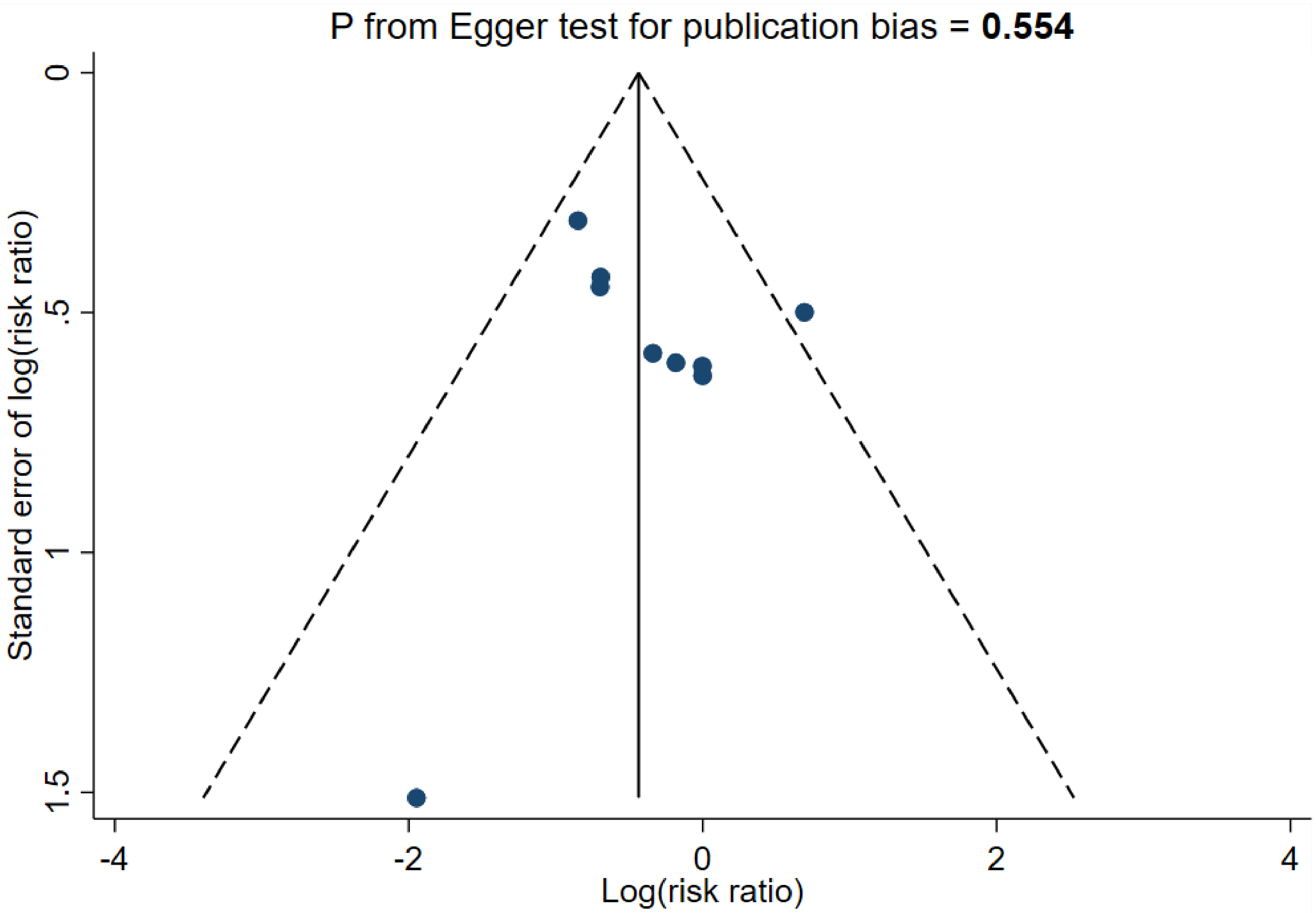

Figure S269 Funnel plot of Bronchitis

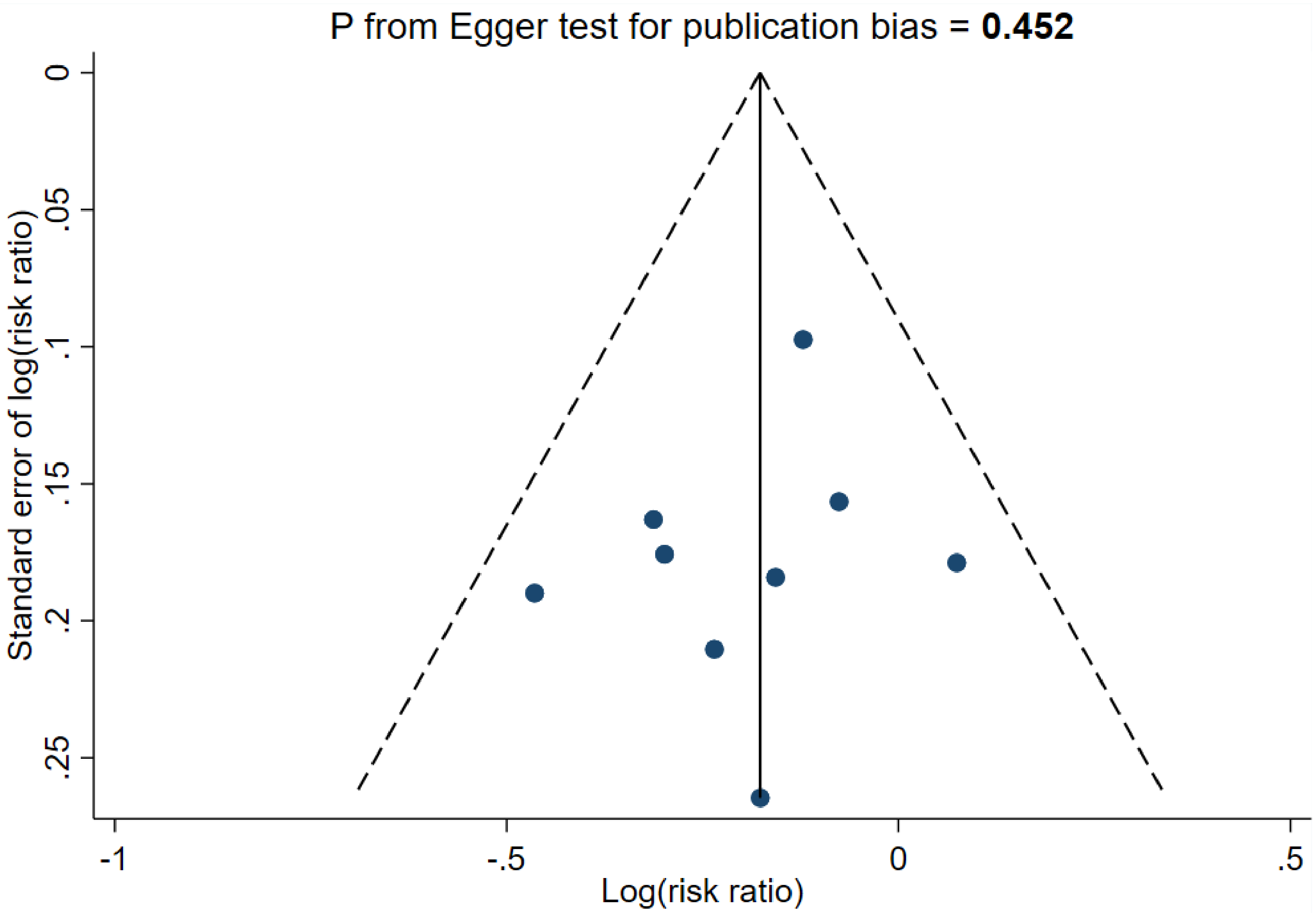

Figure S270 Funnel plot of Pneumonia

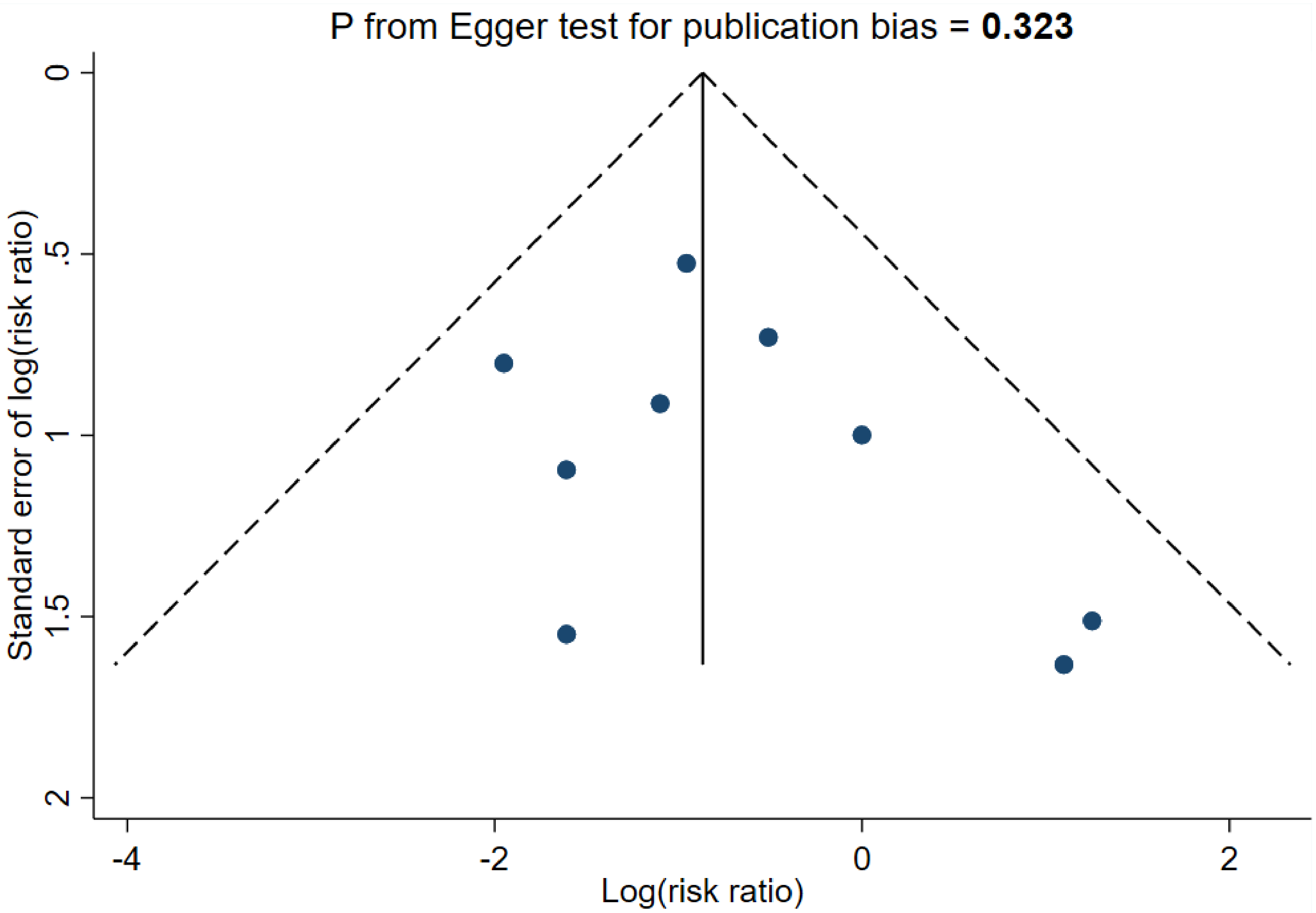

Figure S271 Funnel plot of Respiratory tract infection

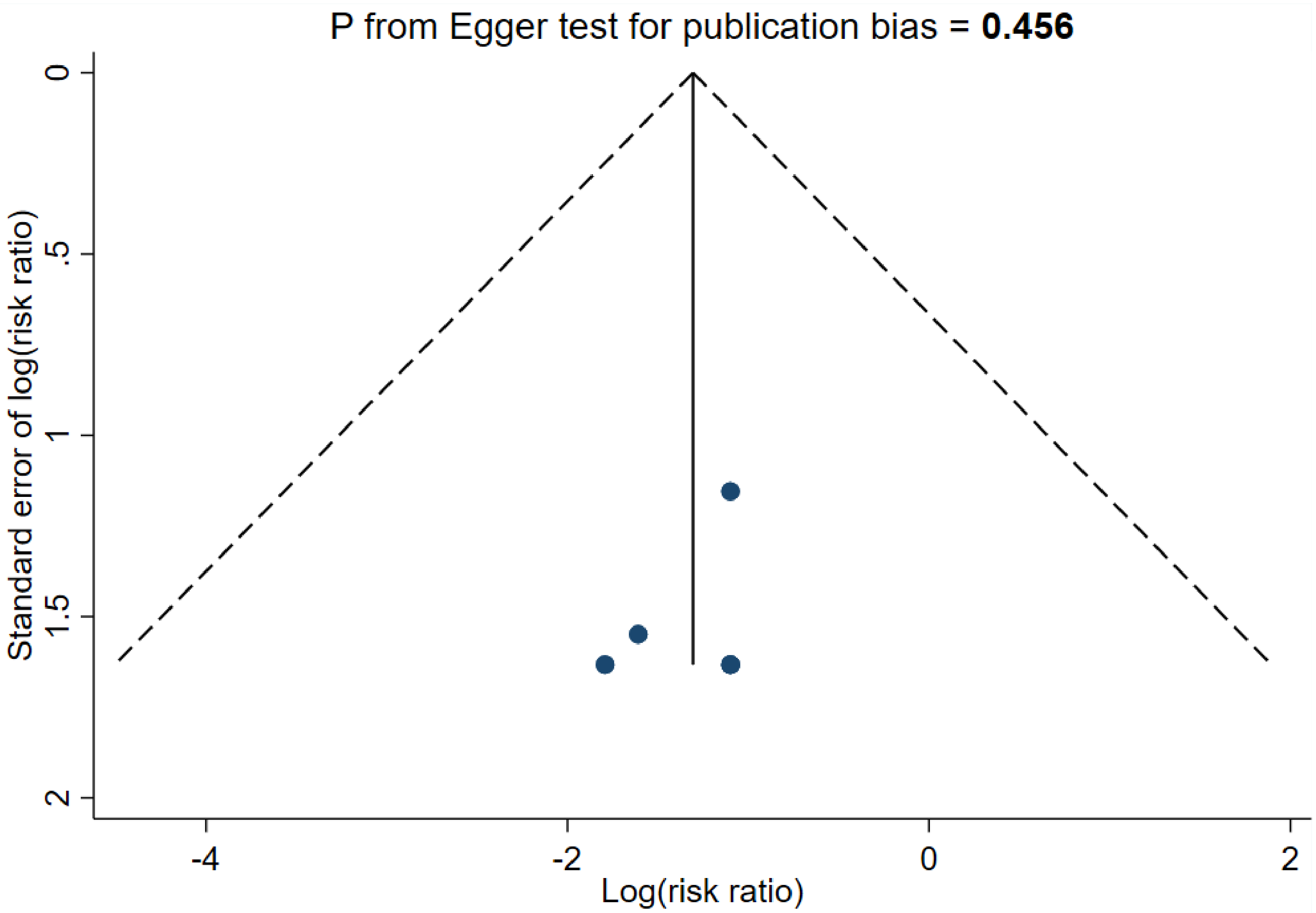

Figure S272 Funnel plot of Non-small cell lung cancer
